# Nickel-Catalyzed Enantioselective Coupling Reactions of Fluorinated Sorbamides with Aldehydes Affording *Anti*-Configured β‑Di(tri)fluoromethyl Alcohol Derivatives

**DOI:** 10.1021/jacs.6c09846

**Published:** 2026-06-24

**Authors:** Noah Richter, Gianluca Regni, Lorenzo Baldinelli, Markus Leutzsch, Giovanni Bistoni, Alois Fürstner

**Affiliations:** † Max-Planck-Institut für Kohlenforschung, 45470 Mülheim/Ruhr, Germany; ‡ Department of Chemistry, Biology, and Biotechnology, 9309 University of Perugia, I-06123 Perugia, Italy

## Abstract

A catalyst generated in situ from a bench-stable nickel stilbene complex and the axially chiral VAPOL-derived phosphoramidite ligand **L1** is capable of affecting the asymmetric reductive coupling of aldehydes with 6,6-difluorosorbamide or 6,6,6-trifluorosorbamide derivatives and related fluorinated compounds using Et_3_B as the promoter. The ligand controls the regioselective course and imposes excellent levels of asymmetric induction onto the reaction that generates di­(tri)­fluoromethylated stereogenic centers concomitant with an adjacent secondary alcohol from the prochiral substrates. Because the resulting structural motif constitutes a fluorinated bioisostere of a common type of *anti*-configured deoxypropionate unit, the new method potentially qualifies for a “fluoride scan” of numerous bioactive (natural) products. A mechanistic study revealed that one cannot extrapolate from the nickel speciation in solution and/or accessible by crystallization to the actual site of the polarized 1,3-diene at which the reductive C–C-bond formation will take place. Moreover, the only detectable complex derived from the Ni(0) precatalyst, **L1**, and a trifluoromethylated sorbamide is off the catalytic cycle; it serves as a “reservoir”, as clearly manifested in the negative nonlinear effect ((−)-NLE) between the optical purity of **L1** and that of the resulting product. A DFT study allowed these observations to be rationalized and a mechanism of the reaction to be proposed.

## Graphical abstract

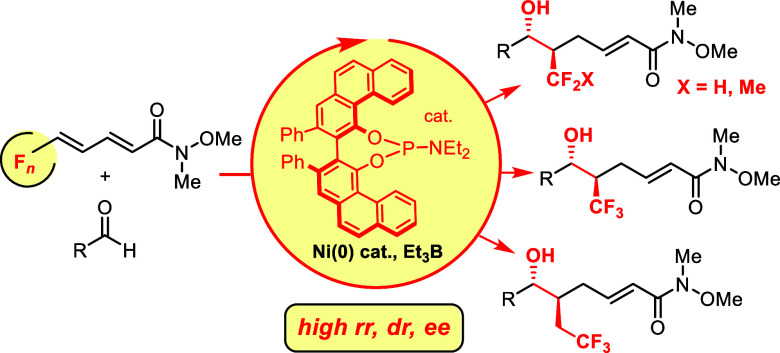

## Introduction

Our group has recently found that the axially chiral VAPOL-derived phosphoramidite ligand **L1** is capable of reverting the regiochemical course of nickel-catalyzed reductive coupling reactions of functionalized 1,3-dienes and aldehydes or certain imines; at the same time, this ligand imposes high levels of asymmetric induction on the actual bond-forming event, independent of whether the diene is electron-rich (e.g., **1**), electron-deficient (e.g., **3**), or has a push–pull character (e.g., **5**).^1–6^ These virtues allowed us to devise novel entries into optically active monoprotected 1,2-diols, methyl-branched secondary alcohols and two different routes to 1,2-aminoalcohol derivatives; representative examples (**2**, **4**, **6**) are shown in Scheme 1. In all cases, C–C bond formation occurs distal to the site that will react in the absence of **L1**.^7–10^ Asymmetric variants of nickel-catalyzed reductive coupling reactions of 1,3-dienes in general had been a real challenge for almost two decades;^11^ the few early examples were of limited scope,^12,13^ whereas more broadly applicable solutions have been forthcoming only recently.^14–22^ When seen against this backdrop, the novel transformations enabled by **L1** are deemed a significant advance.

**Scheme 1:**
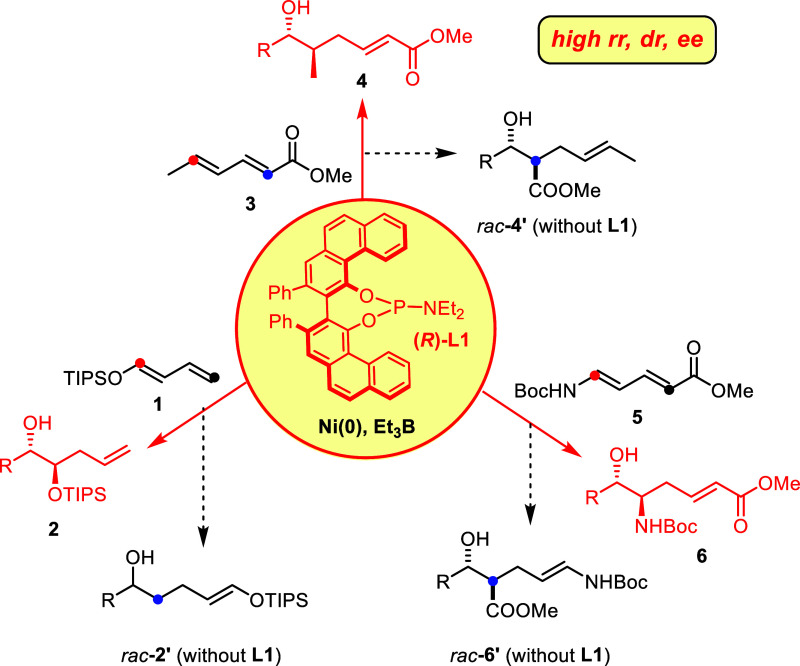
Prior Art: The VAPOL-Derived Phosphoramidite Ligand **L1** as a Game-Changer

Sorbate esters (amides) such as **3** are one particular type of functionalized 1,3-dienes that were found to be well-behaved with the Ni(0)/**L1** catalyst system, and even the analogous trifluoromethyl derivative **7** was compliant (Scheme 2).^2^ This latter result is particularly noteworthy since other nickel-catalyzed reductive coupling reactions of trifluoromethyl alkenes proceed with the loss of a fluoride atom.^23^ While the −CF_3_ substituent is faithfully preserved in our case, **7** was one of the few substrates tested in which appreciable amounts of the “ordinary” aldol-type isomer **8′** were formed in addition to the desired product **8**.^2^ This outcome was tentatively ascribed to the fact that **7** has electron-withdrawing substituents on both ends of the conjugated diene, whereas the ordinary sorbate **3** does not; the two double bonds of **7** might hence be able to ligate an active Ni(0) species similarly well, which probably impairs the regiochemical course.

**Scheme 2:**
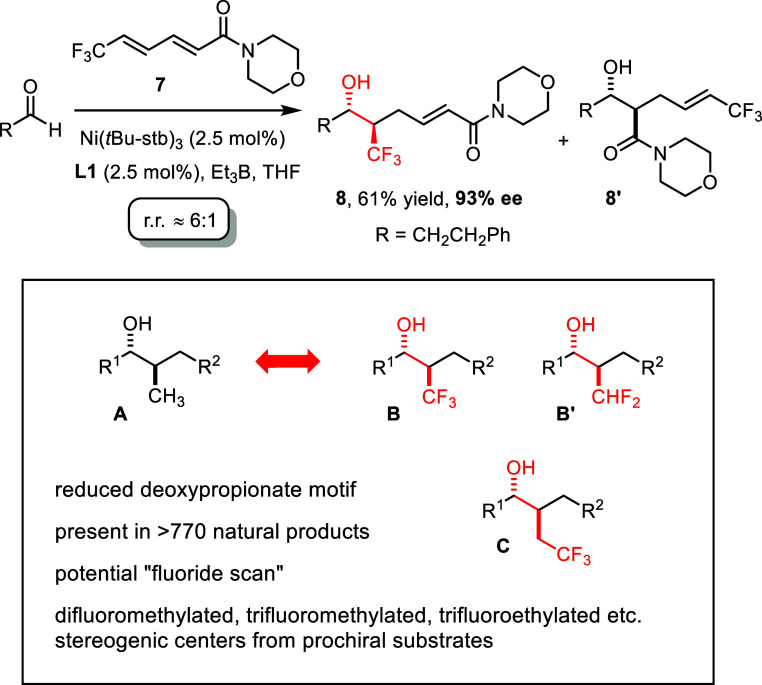
Lead Finding and Its Possible Implications

This issue notwithstanding, the ability to generate a stereogenic center carrying a −CF_3_ group concurrent with a secondary alcohol in a catalytic asymmetric manner from prochiral substrates holds considerable promise.^24–28^ Since the resulting structural motif **B** ^29–32^ can potentially be regarded as a trifluorinated (bio)­isostere of the common type of reduced deoxypropionate subunit **A**,^33^ this transformation might lend itself to a “fluoride scan” of numerous bioactive (natural) products,^34–38^ provided it can be generalized and improved. Numerous extensions to products such as **B′** and **C** can also be envisaged; in this context, it is of particular note that compounds of type **B′** comprising a −CHF_2_ substituent do not count as PFAs (in contrast to derivatives comprising a trifluoromethyl or difluoromethylene group) and might hence be particularly valuable; the synthetic repertoire allowing for their preparation in an optically active form is still rather limited.^39–41^

## Results and Discussion

### Method Development and Scope

Encouraged by this outlook, we embarked into a more systematic investigation. First, however, a number of practical issues had to be addressed. In our preliminary study, we had chosen the morpholine amide of 6,6,6-trifluorosorbic acid (**7**) as the substrate,^2^ mostly because the corresponding methyl ester is highly volatile, the products derived from it are hard to detect, and the regioisomeric product mixture formed proved difficult to separate. Although the morpholine amide **7** was found to be more suitable overall, it seemed reasonable to screen yet other acid derivatives in the hope of improving yield and selectivity of the coupling event. Moreover, the route initially chosen to make **7** by a rhodium-catalyzed, silver-mediated reaction of 4-acryloylmorpholine with α-trifluoro-methylacrylic acid had been low yielding and required extensive purification;^42^ clearly, a more productive, flexible, and better scalable route to substrates of this type was necessary.

This initial goal was achieved as shown in Scheme 3. Key to success was the finding that the aluminum alkoxide **10** formed upon reduction of commercial ester **9** with Dibal-H can be directly subjected to a Horner–Wadsworth–Emmons (HWE) reaction, thus obviating the need to isolate and handle volatile and highly hygroscopic trifluorocrotonaldehyde.^43^ This tactic worked well on a multigram scale, providing access to a set of different ester and amide derivatives for testing; the isomeric purity of the resulting diene subunit was high in all cases. When starting from difluorocrotonate **14**, which is readily accessible from the commercial β-ketoester **13** by NaBH_4_ reduction followed by dehydration,^44^ the same sequence furnished the analogous difluorinated compounds.

**Scheme 3:**
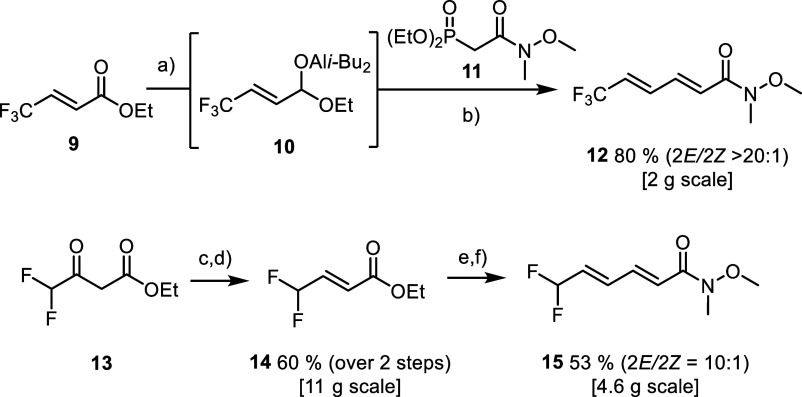
Scalable Preparation of Fluorinated Sorbamides^a^

a Reagents and Conditions: (a) Dibal-H, CH_2_Cl_2_, −78 °C; (b) **11**, NaH, Et_2_O, −78 °C to RT, 80% (over 2 steps); (c) NaBH_4_, toluene; (d) MsCl, Et_3_N, CH_2_Cl_2_, −70 °C to RT, 60% (over 2 steps); (e) Dibal-H, CH_2_Cl_2_, −78 °C; (f) **11**, NaH, Et_2_O, −78 °C to RT, 53% (over 2 steps).

A brief screening allowed us to identify the corresponding Weinreb amides **12** and **15** as the most adequate coupling partners, resulting in better yields than the morpholine amide **7** originally used, as well as an improved regioselectivity in most cases.^45^ Control experiments showed that **12** does not react at all with an aldehyde partner in the presence of Ni(0) cat/Et_3_B in the absence of **L1**; this finding stands in contrast to the results previously observed with methyl sorbate **3**, where ligand-free conditions led to the formation of the regioisomer *rac*-**4**′.^2^ Equally noteworthy is the fact that neither PPh_3_ or PCy_3_ nor the commercial phosphoramidite (*R*)-Monophos switches any reactivity on (for the control experiments, see the Supporting Information): **L1** is hence the critical reactivity determinant; at the same time, it controls the regiochemical course and imposes an appreciable level of asymmetric induction (Scheme 4). As an additional, non-negligible bonus, the resulting products **17** comprising a Weinreb amide terminus are less polar than their morpholine amide analogues, which makes their isolation in an analytically pure form far easier. Although the corresponding benzyl ester also proved highly reactive, the level of asymmetric induction reached was poor and the resulting regioisomers of product **18** could not be separated by flash chromatography. All reactions were performed with the bench stable nickel stilbene complex [Ni­(*t*Bu-stb)_3_] (**16**) as the precatalyst, thus making the handling of [Ni­(cod)_2_] unnecessary;^46,47^ a commercial solution of BEt_3_ in THF served as the promoter.

**Scheme 4:**
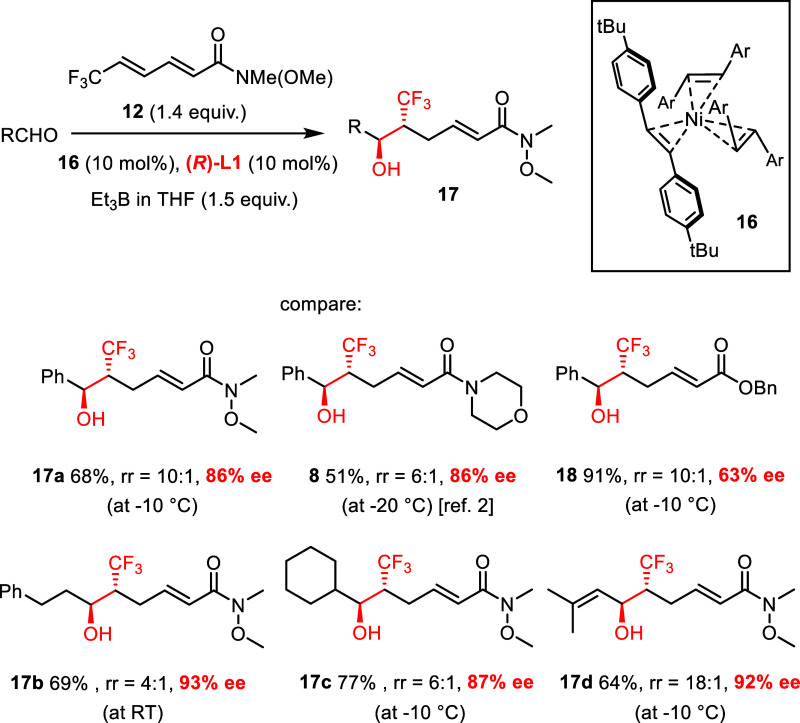
Reductive Coupling of Trifluorosorbamide **12** ^a^

a The dr was ≥20:1 in all cases; the regioisomer ratio (rr) was determined from the ^19^F NMR spectrum of the crude product; since the isomers were separable by flash chromatography (except for **18**), the yields refer to the isolated pure compound.

Gratifyingly, the difluorinated sorbamide **15** performed even better than its trifluorinated sibling **12** in that it led to higher chemical yields and rrs as well as to improved ees in most cases; the direct comparison of the pairs **17a**/**20a**, **17b**/**19d**, and **17c**/**19g** illustrates this trend, whereas the outcome for **17d**/**21c** was by and large identical. Since *anti*-configured secondary alcohols of type **B′** with a neighboring difluoro-methyl substituent are difficult to make in an optically active form,^39–41^ the scope of the new method was explored in detail. Aliphatic aldehydes were found to be good coupling partners, furnishing the desired products in generally high yields and excellent regio- and diastereoselectivity (Scheme 5); in a number of cases, the product was virtually formed as a single geometric isomer (rr ≥ 20:1, dr ≥ 20:1).^48^ Arguably most important is the remarkably high level of asymmetric induction in practically all cases investigated. This is true independent of whether the aldehyde is linear or branched at the α- or β-position. Along the same lines, respectable diastereoselectivities were observed in reactions with chiral aldehydes such as (*S*)-β-citronellal, glyceraldehyde, and the aminoaldehyde derived from N-Boc protected alanine. Somewhat unexpectedly, however, the latter two substrates reacted less regioselectively, furnishing products **19p** and **19q** with modest rrs. This shortcoming is evidently caused by the heteroatom substituent adjacent to the carbonyl group rather than the presence of the chiral center since a control experiment with 2-benzyloxyacetaldehyde leading to compound **19o** also suffered from poor regiocontrol. Apart from this unfavorable proximity effect, the method is compatible with a number of polar and apolar functional groups. Moreover, the reaction scaled well, furnishing product **19c** in high yield and excellent optical purity on a gram scale (93%, rr > 20:1, dr > 20:1, 95% ee).

**Scheme 5:**
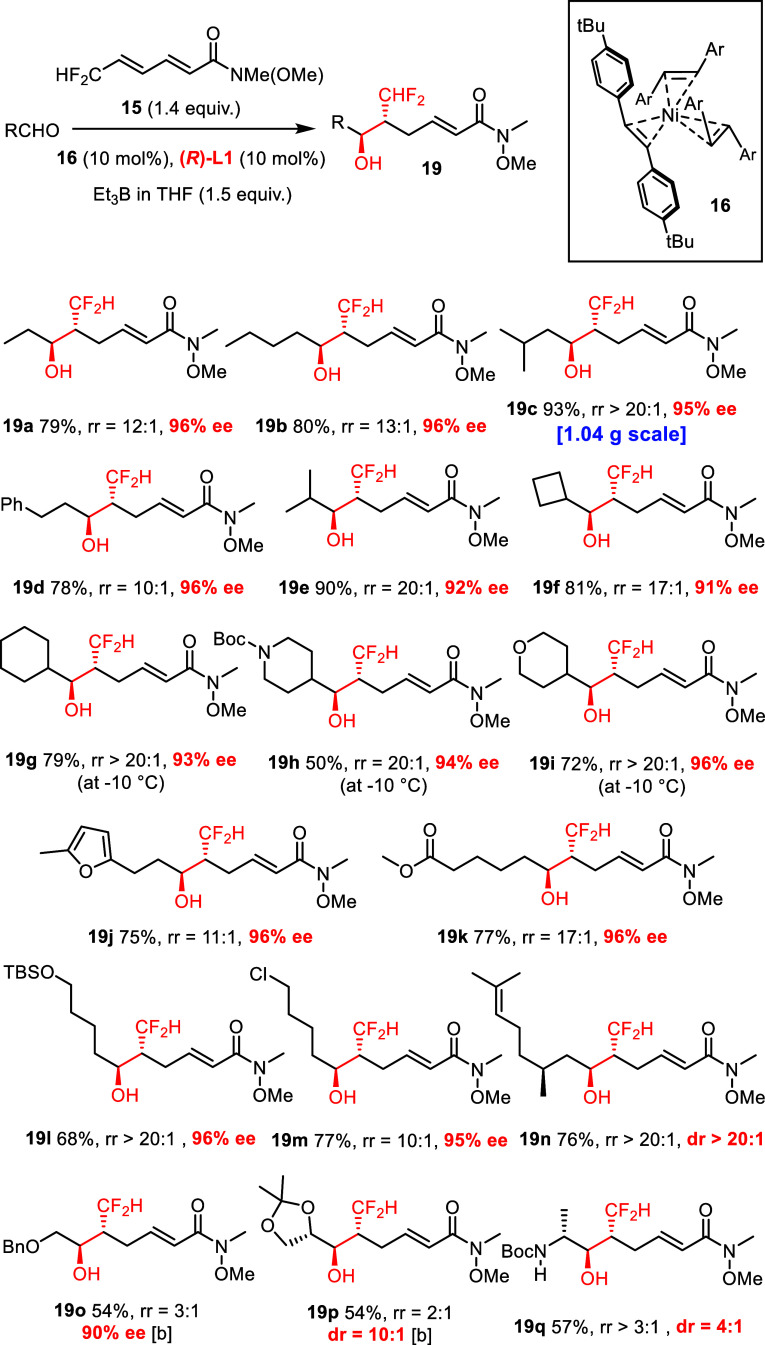
Reductive Coupling of Difluorosorbamide **15** with Aliphatic Aldehydes^a^

a All reactions were performed under the conditions shown in Scheme 4 in THF at RT unless stated otherwise.
b In toluene; the dr was ≥20:1 in all cases; the regioisomer ratio (rr) was determined from the ^19^F NMR spectrum of the crude product; since the isomers were separable by flash chromatography, the yields refer to the isolated pure compound.

An assortment of aromatic and heteroaromatic aldehydes of greatly different electronic character were also found to be well-behaved reaction partners (Scheme 6); this finding is gratifying as they had previously turned out to be no good substrates in other asymmetric reactions leading to a similar difluoromethylated motif.^30^ The optical purity attained tends to be slightly lower than those observed in the aliphatic series even when the reactions are performed at −10 °C rather than room temperature. The comparison of **20e** and **20g**,**h** suggests that more electron-rich aldehydes result in higher levels of asymmetric induction. The same trend surfaces from the heteroaromatic subset investigated as the optical purity of products **20k**,**l** derived from furan-2-carbaldehyde and a protected pyrrole-2-carbaldehyde was significantly higher than that of compound **20j** derived from pyridine-4-carbaldehyde. Despite the moderate ee, this latter example implies that the fluorinated diene derivatives used in the current study must be privileged coupling partners since pyridine-4-carbaldehyde as a potential ligand to Ni(0) had previously failed to react with the ordinary nonfluorinated sorbate ester **3**.^2^ The favorable kinetic profile may also explain why 3,5-dibromobenzaldehyde proved compatible; since bromine substituents had been borderline in our earlier studies,^49^ the successful formation of **20i** suggests that the reductive C–C bond formation is faster than the competing oxidative insertion of the low-valent nickel species into the C–Br bonds.

**Scheme 6:**
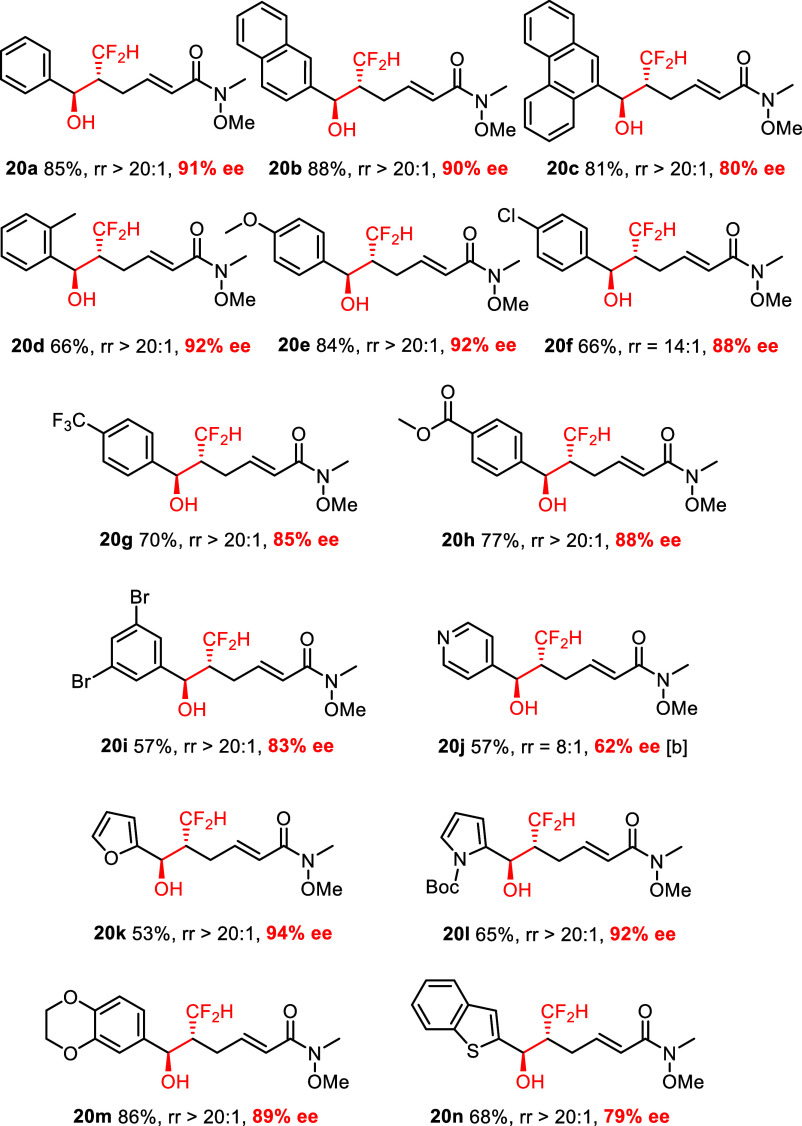
Reductive Coupling of Difluorosorbamide **15** with (Hetero)­Aromatic Aldehydes^a^

a All reactions were performed under the conditions shown in Scheme 4 in THF at −10 °C.
b Using 3 equiv of BEt_3_; the dr was ≥20:1 in all cases; the regioisomer ratio (rr) was determined from the ^19^F NMR spectrum of the crude product; since the isomers were separable by flash chromatography, the yields refer to the isolated pure compound.

Based on this rationale, we felt encouraged to test yet other substrates that had been challenging or even unsuitable in the past. α,β-Unsaturated aldehydes fall into this category; they failed to react with sorbate ester **3** as well as with 5-amino-2,4-pentadienoates **5**.^50^ It is therefore deemed remarkable that the asymmetric nickel-catalyzed coupling of **15** with various enals proceeded smoothly and with generally high levels of asymmetric induction (Scheme 7); chiral enals such as (1*R*)-myrtenal or (*S*)-perillaldehyde were also found to be adequate partners. In all cases investigated, the resulting multifunctionalized products were formed in a highly regioselective manner (rr > 20:1), thus making product isolation straightforward. However, limitations were encountered with 3-phenylpropiolaldehyde as a representative ynal, pivaldehyde (steric hindrance), Garner’s aldehyde (poorly soluble in THF), 4-methylthiazol-5-carbaldehyde (likely for the thiophilicity of Ni(0)), and—perhaps unsurprisingly−*N*-formylmorpholine (Scheme 7, bottom).

**Scheme 7:**
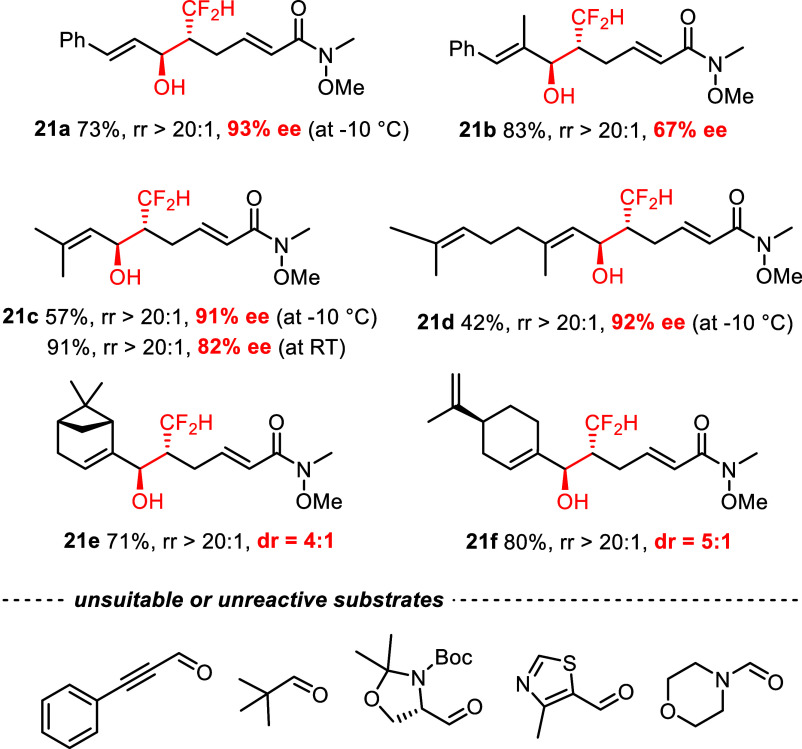
Reductive Coupling of Difluorosorbamide **15** with Enals^a^

a All reactions were performed under the conditions shown in Scheme 4 at RT, unless stated otherwise; the dr was ≥20:1 in all cases; the regioisomer ratio (rr) was determined from the ^19^F NMR spectrum of the crude product; since the isomers were separable by flash chromatography, the yields refer to the isolated pure compound.

A few additional fluorinated diene derivatives were also tested; their preparation followed the logic outlined for **12** and **15** (for details, see the Supporting Information). Even though one might imagine that a branch adjacent to the reacting olefin might be detrimental for the outcome, compound **22** proved well-behaved, furnishing excellent ees with an aliphatic, an aromatic, and an α,β-unsaturated aldehyde as the reaction partners (**23a**–**c**, Scheme 8). The same is true for dienamide **24a**, in which the F_3_C- group is formally separated from the diene system by a methylene “spacer”; once again, excellent results were obtained with all three types of model aldehydes (**25a**–**c**). A limitation, however, was marked by compound **24b** bearing a pentafluoroethyl substituent as the optical purity of the resulting product **26** was lower and the otherwise excellent regiocontrol completely lost; as the comparison with **24a** suggests, the strong electron withdrawal on both sides of the central diene unit of **24b** presumably prohibits a clear differentiation of the alkenes and hence allows the catalyst to induce coupling at both possible sites with similar ease.

**Scheme 8:**
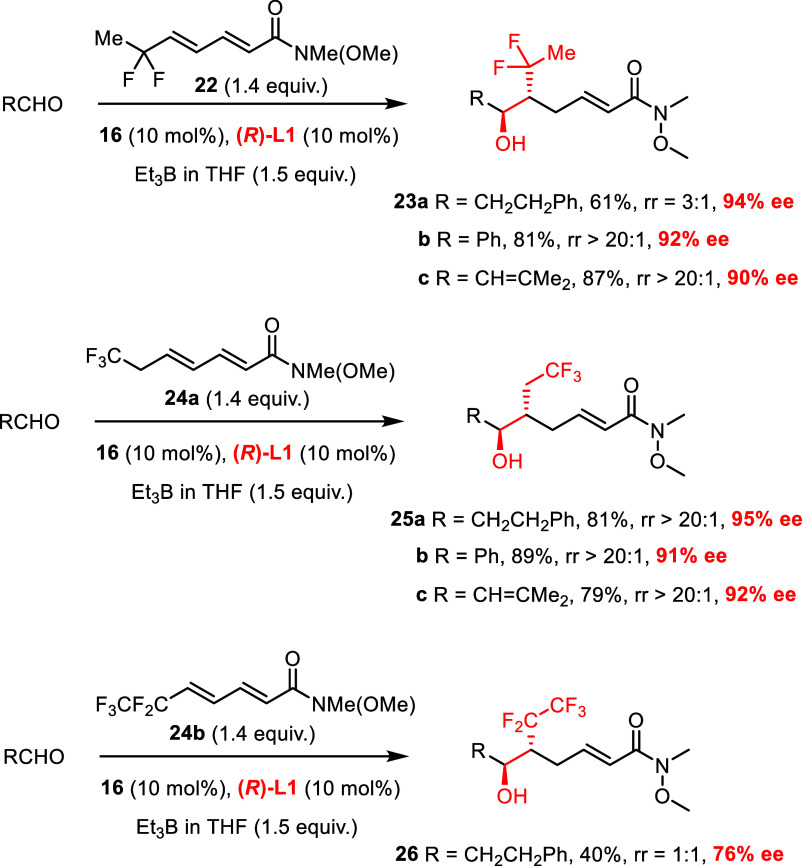
Reductive Coupling of Additional Fluorinated Dienamides^a^

a All reactions were performed at RT; the dr was ≥20:1 in all cases; the regioisomer ratio (rr) was determined from the ^19^F NMR spectrum of the crude product; since the isomers were separable by flash chromatography, the yields refer to the isolated pure compound.

The relative and absolute configurations of **20e** (Figure 1) and the silyl ether **28** derived from **19c** (the structure is contained in the Supporting Information) were determined by single-crystal X-ray diffraction analysis; these examples are representative for the aromatic and aliphatic series, respectively. Importantly, the observed configurations are mutually consistent; moreover, they correspond to the outcome previously observed with the nonfluorinated sorbate ester **3**.^2^ Therefore, all other compounds prepared during this study were confidently assigned by analogy.

**Figure 1:**
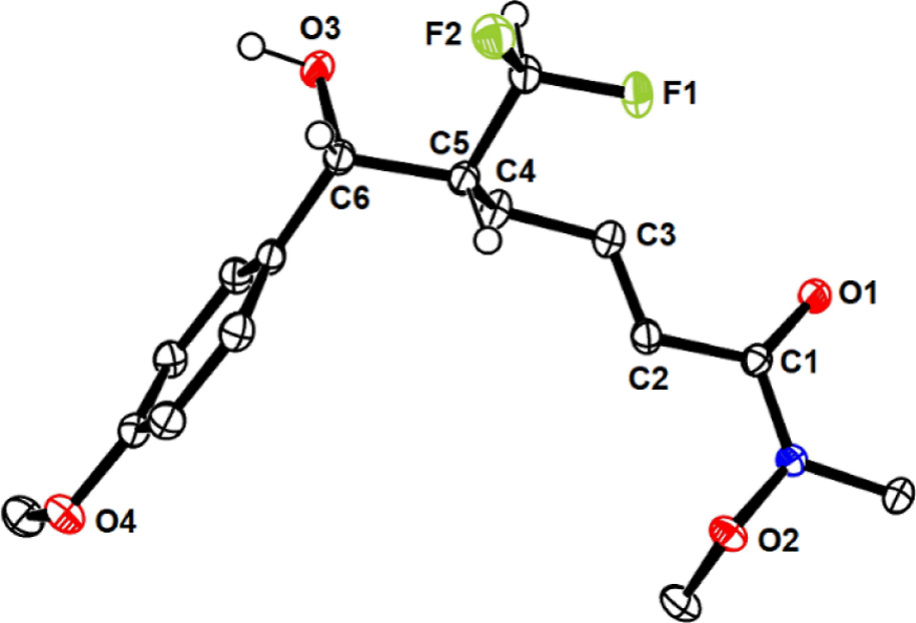
Structure of compound **20e** in the solid state; H atoms omitted for clarity, except those at the chiral centers generated by the nickel-catalyzed reductive coupling reaction.

### Applications

As briefly mentioned above, the secondary alcohols flanked by a –CF_3_ or –CHF_2_ group can potentially be regarded as fluorinated isosteres of *anti*-configured deoxypropionate units; many applications can hence be envisaged. In this context, it is emphasized that the Weinreb amide terminus with the conjugated double bond provides ample opportunity for downstream functionalization.^45^ Just a few initial forays were undertaken to illustrate this aspect (Scheme 9). Conversion of **19c** into **27** and **29** show some obvious possibilities. Furthermore, compound **19b** (ee = 96%) derived from pentanal was subjected to ozonolysis, and the resulting hemiacetal oxidized to give **30**. This product is the difluorinated analogue of *trans*-whiskey lactone (also called quercus lactone), a volatile aroma compound found in alcoholic beverages aged in oak barrels. Numerous related lactone derivatives occur in nature,^51^ which could be made in a fluorinated format for professional sensory testing by the new method. An entirely different class of natural product analogues is reached upon oxidative cleavage of the double bond of **19o**. The resulting compound **31** mimics a deoxyfuranose derivative, the 3-hydroxy group of which is formally replaced by a −CHF_2_ substituent. The electron withdrawal by this group in the σ-frame presumably stabilizes the glycosidic bond against acid-catalyzed cleavage. Indeed, related nucleoside derivatives with a −CF_3_ group at the sugar C3-position are known in the literature for their appreciable activity against hepatitis B virus while being noncytotoxic.^52^

**Scheme 9:**
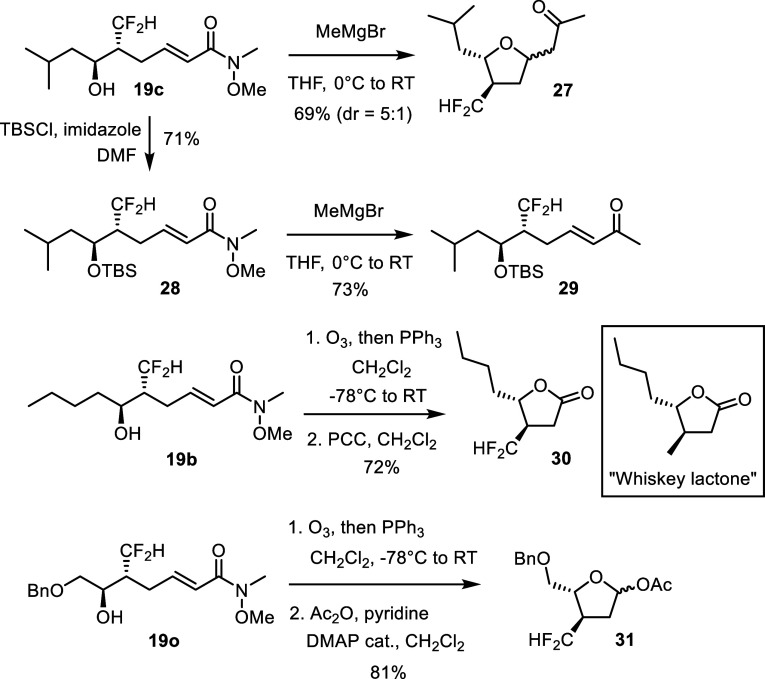
Downstream Functionalization

### Mechanistic Studies

Experimental studies into the mechanism of nickel-catalyzed reductive coupling reactions of unsaturated substrates in general and of 1,3-dienes in particular are challenging for a number of reasons.^53–55^ Therefore, the question central to the current and all our previous studies in the field had so far remained largely unanswered as to why the VAPOL-derived phosphoramidite **L1** inverts the course of the coupling reaction and, at the same time, is capable of imposing high levels of asymmetric induction on the actual C–C bond forming event.^56^ Even if the problem is simplified and the stereochemical aspect is disregarded, the phenomenon of regiodivergence itself is by no means well-understood.^57^ We had originally noticed this effect with ligands as simple as PPh_3_ and PCy_3_ when reacting sugar aldehydes with isoprene.^58,59^

Leaving these issues aside for the moment, one can say that consensus has been reached in the literature as to the elementary steps by which an unsaturated substrate reacts with carbonyl derivatives. Early mechanistic work concerning the “ligand-free” nickel-catalyzed reductive coupling of 1,3-dienes^9,60^ has later been supplemented by computational studies. Specifically, Houk and co-workers showed that the aldehyde and the PR_3_ ligand compete for the remaining two binding sites to form nickel–alkyne (or enyne) adducts such as **D** and **E**, resulting in a pre-equilibrium (Scheme 10).^61,62^ For the heteroleptic complex **E** to enter the actual catalytic cycle, the aldehyde has to switch to an η^2^ (“side on”) bonding mode, as shown in **F**. The subsequent oxidative cyclization affords an oxa-nickelacycle **G** with four low-lying d-orbitals.^53,63,64^ It was proposed that BEt_3_ comes into play only at this point; because of its oxophilicity, the transmetalation of **G** to give **H** is highly exergonic. Subsequent β-hydride elimination/reductive elimination affords the product as borinate ester **J** (prior to work up) and regenerates the propagating low-valent nickel species.^61,62^ Variations of this basic scenario have served to explain all sorts of nickel-catalyzed reductive coupling reactions developed since then.

**Scheme 10:**
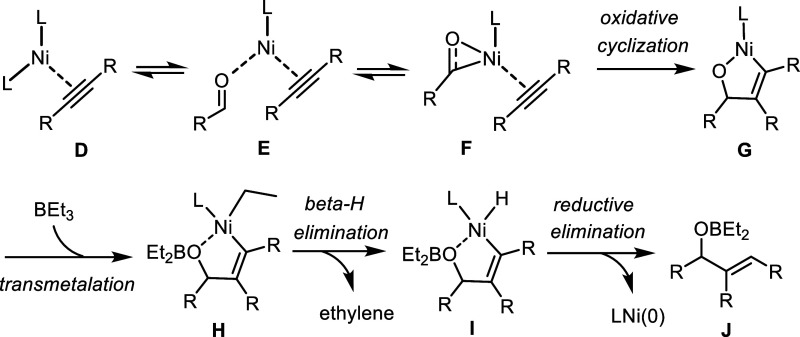
“Consensus Mechanism”^a^

a Elementary steps of nickel-catalyzed reductive coupling of unsaturated substrates and aldehydes; in the specific computed example, an alkyne (or an enyne) was chosen as the π-component, and ligand L was PPh_3_, see refs 61 and 62

Although we have no doubt that our “reversed” coupling chemistry passes through the same elementary steps and have therefore used this rationale to explain our results,^1–3^ we keep trying to consolidate the central mechanistic claims by a combined experimental/computational study. Our first objective was a better understanding for the coordination chemistry of Ni(0) with functionalized diene ligands in general and dienoate derivatives in particular, which had not been studied in detail in the past.^65,66^ First, we wondered whether or not differently ligated nickel fragments bind to the opposite double bonds of (fluorinated) sorbate esters (amides) (Scheme 11).^67^ Gratifyingly, we managed to crystallize the adduct formed on the addition of [Ni­(cod)_2_] to a solution of methyl sorbate (**3**).^68^ In the resulting complex **32**, the 1,3-diene unit is bound in the s-*cis* conformation (Figure 2). This result is at odds with earlier assumptions, which had suggested that dienes get η^4^-bound to the Ni(0) center in a rather unusual s-*trans* conformation.^9^ A closer look at the C–Ni distances in **32** shows that the contacts to C2, C3, and C4 are considerably shorter than that to C5, imparting a certain polarized character onto this diene complex. The uneven bonding is also evident from the significantly different lengths of the two double bonds, in that C2–C3 is notably elongated compared to C4–C5 as a result of stronger electron back-donation into the antibonding π* orbital. It is important to note that all structural features of **32** were well-reproduced by DFT at the PBE-D4/def2-SVP/CPCM­(toluene) level of theory^69–72^ using the ORCA quantum chemistry package (for details, see the Supporting Information).^73^ This benchmark made us confident that the computational mechanistic results reported below represent the best accuracy that could be achieved within a reasonable CPU time for this challenging 3d transition metal system.

**Scheme 11:**
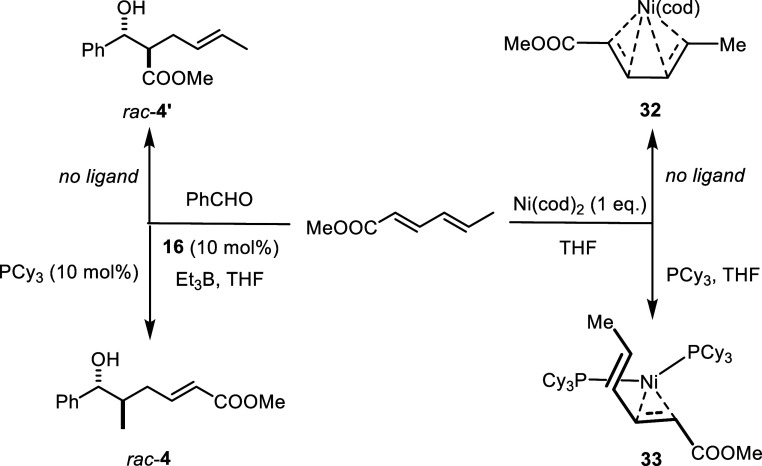
Regiodivergent Course of the Reductive Coupling of Methyl Sorbate under “Ligand-Free” Conditions and in the Presence of PCy_3_; Structure of Derived Ni(0)–Diene Complexes

**Figure 2:**
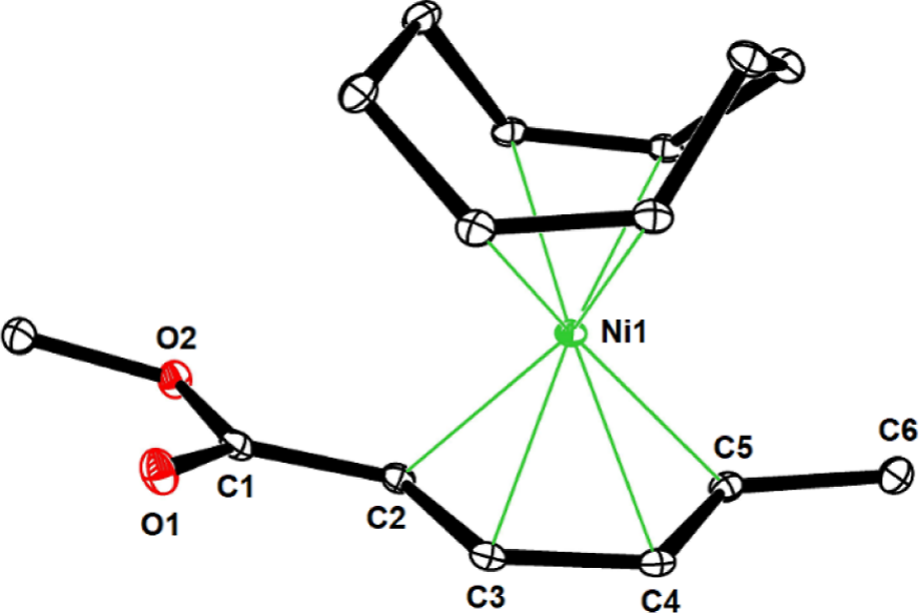
Structure of complex **32** in the solid state; H atoms omitted for clarity; chemical numbering scheme. Selected bond lengths (Å) and angles (°): Ni1–C2 2.1442 (12), Ni1–C3 2.0099 (12), Ni1–C4 2.0720 (12), Ni1–C5 2.2109 (12), C2–C3 1.4072 (17), C3–C4 1.4319 (18), C4–C5 1.3853 (18), C2–C3–C4 121.64 (11), and C3–C4–C5 121.86 (11).

The double bond conjugated to the ester remains the binding site for the Ni(0) center in the solid state when PCy_3_ is present, although this ligand reverts the course and entails coupling at the distal site; complex **33** was the only detectable species in solution (for details, see the Supporting Information). The structure in the solid state shows a more “localized” binding mode in that there is no noticeable interaction between the metal center and the distal C4–C5 olefin at which the reaction will occur (Figure 3). As expected, the formal replacement of the 1,5-cyclooctadiene ligand by the electron-donating phosphine results in stronger electron back-donation into the π* orbital, as manifested in shorter Ni–C2 and Ni–C3 contacts but an expanded C2–C3 double bond compared to those in complex **32**. The overall geometry about the Ni(0) center is close to trigonal planar.

**Figure 3:**
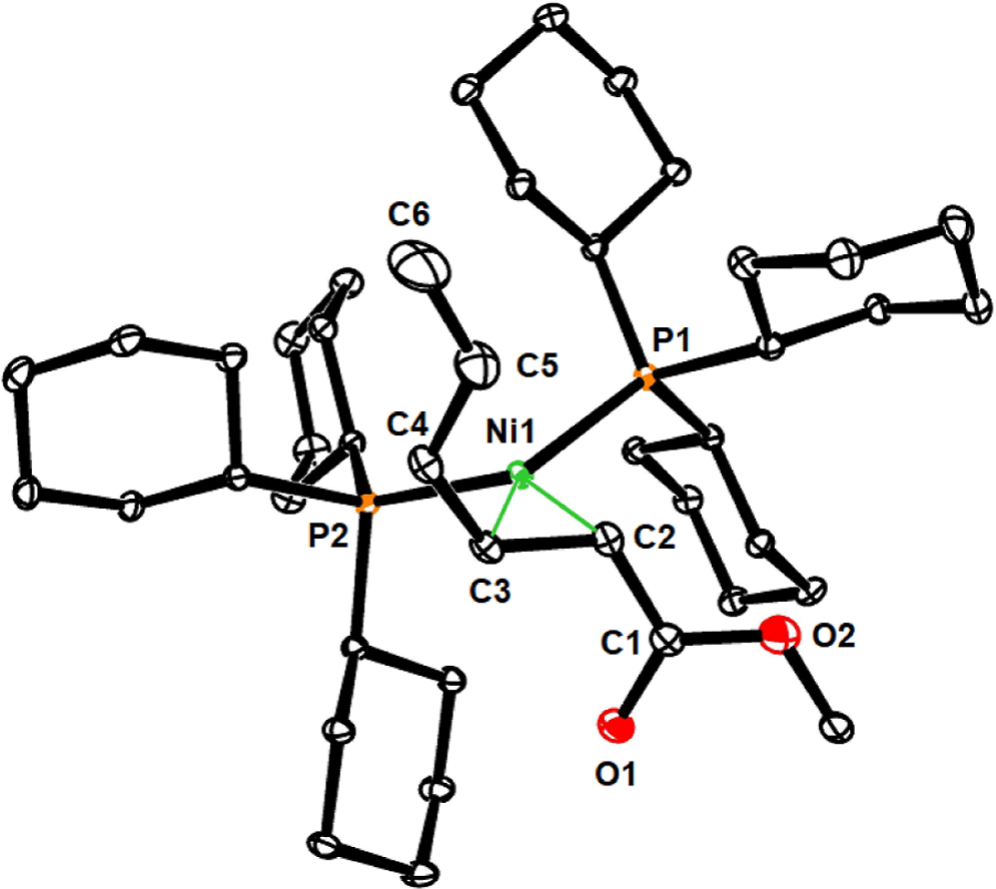
Structure of complex **33** in the solid state with a chemical numbering scheme; disorder not shown and H atoms omitted for clarity. The full structure is contained in the Supporting Information. Selected bond lengths (Å) and angles (°): Ni1–C2 1.987 (2), Ni1–C3 2.003 (2), C2–C3 1.428 (3), C4–C5 1.41 (3), P1–Ni1–C2 100.75 (6), P2–Ni1–C3 102.68 (6), and P1–Ni1–P2 117.61 (2).

As summarized in Scheme 11, the sorbate ester **3** reacts under phosphine-free conditions to give aldol-type adducts such as *rac*-**4′**, whereas PCy_3_ reverts the course and leads to product *rac*-**4**: it is the olefin distal to the ester that succumbs to C–C-bond formation, even though the [(Cy_3_P)_2_Ni­(0)] fragment is bound to the proximal double bond in the isolated complex **33**. To determine whether the binding site and the site of reaction also differ when the VAPOL-derived phosphoramidate **L1** is used, numerous attempts were made to grow crystals of the corresponding nickel complex, but to no avail.

Therefore, we sought to extract relevant information from the NMR spectra of different diene/ligand combinations in the presence of [Ni­(cod)_2_].^68^ Massive stability problems, however, thwarted our efforts: the spectra of methyl sorbate (**3**) and **L1** as well as of the fluorinated derivative **12** (or **15**) and PCy_3_ in [D_8_]-THF showed extremely broad and almost featureless lines even at low temperatures; rapid precipitation of nickel black was observed. In this context, we reiterate that PCy_3_ failed to induce a catalytic coupling reaction of **15** with benzaldehyde (see the control experiments listed in the Supporting Information).

When seen against this background, it is all the more important that the complex derived from **12**, [Ni­(cod)]_2_, and **L1** in [D_8_]-THF, which forms the basis for the asymmetric reductive coupling reactions described herein, could be characterized by spectroscopic means (Scheme 12). Even though just one equivalent of **L1** per Ni(0) was added to the mixture, the only detectable complex **34** evidently carries two such ligands.^74^ The observed ^2^ *J*_PP_ coupling of ≈37 Hz suggests that the two phosphoramidites are positioned *cis* to each other.^75^ The massively broadened signal set vaguely reminiscent of an AB-type system was then deconvoluted by a series of decoupling experiments (Figure 4). This revealed a remarkable interaction between one of the phosphorus atoms and the −CF_3_ substituent of the diene: the surprisingly large *J*_PF_ coupling constant of 12.5 Hz, which is equally manifested in the splitting of the ^19^F NMR signal, is likely due to a through-space rather than through-bond interaction.^76^ Even a cross peak in the ^19^F–^31^P HMBC spectrum was recorded (see the Supporting Information). These data show that one of the **L1** ligands must be in close proximity to the –CF_3_ group, which in turn implies that it is the distal double bond of the fluorinated sorbamide **12** that is ligated to the [(**L1**)_2_Ni­(0)] fragment. This conclusion is further supported by the fact that H4/H5 as well as C4/C5 are much more shielded than H2/H3 and C2/C3, respectively.

**Scheme 12:**
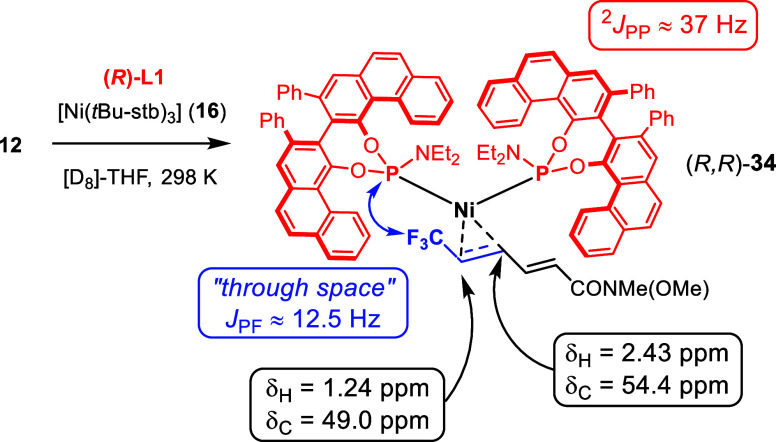
Nickel Complex Derived from Trifluorosorbamide **12** and Ligand **L1**

**Figure 4:**
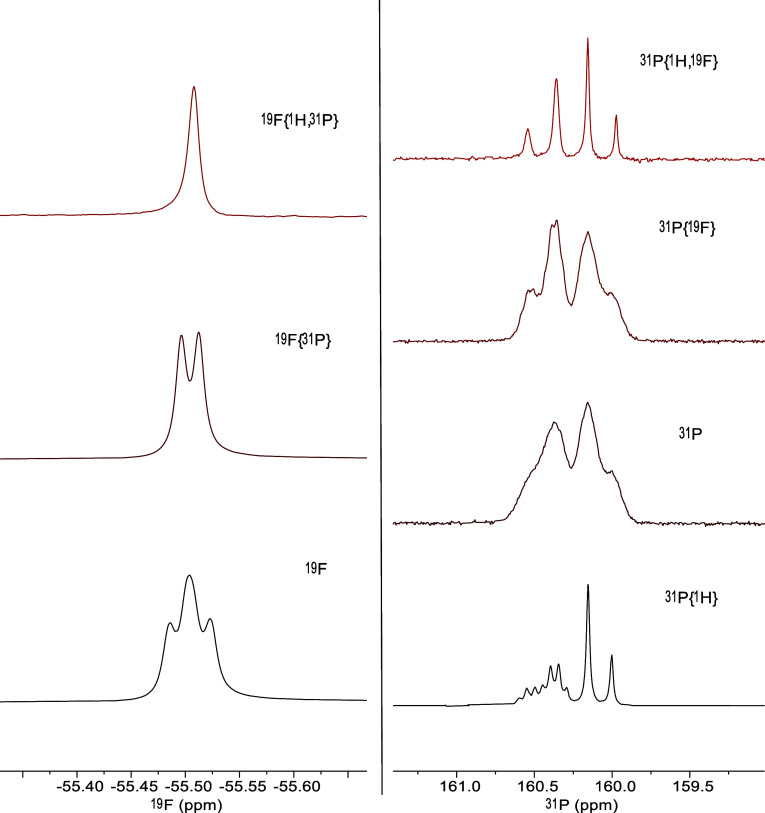
^19^F NMR (left) and ^31^P NMR spectra (right) of complex (*R,R*)-**34** recorded in differently decoupled manner as indicated.

Collectively, these crystallographic and spectroscopic results confirm the notion that Ni(0) fragments carrying different ancillary ligands can coordinate to one or the other double bond of a polarized 1,3-diene substrate. It is of utmost importance, however, to recognize that one cannot extrapolate from the binding site in the ground state to the site at which C–C bond formation will occur when an aldehyde and Et_3_B are present: while phosphine-free [Ni(0)­(cod)] and the [Ni(0)­(PCy_3_)_2_] fragment both ligate the double bond directly conjugated to the ester moiety of **3** (see complexes **32** and **33**), they induce reductive coupling with benzaldehyde at the opposite ends of the diene system, furnishing compounds *rac*-**4′** and *rac*-**4**, respectively (Scheme 11).

We can only speculate at this point as to why it is the alkene of trifluorinated sorbamide **12** distal to the amide group that interacts with [(**L1**)_2_Ni­(0)] (which is also the site where the reaction occurs). It is assumed that the fairly electron-rich metal fragment favors binding to the more electron-deficient of the two olefins, which has the largest orbital coefficients in the LUMO and hence allows for more electron back-donation from the Ni(0) center into the antibonding orbital. As the corresponding complex bearing a [(Cy_3_P)_2_Ni­(0)] fragment could not be characterized (see above), we lack further experimental evidence for whether this preference is (more) general. Since our study showed that the binding pattern in the ground state is not directly correlated with the site of catalytic bond formation, however, we have learned that this question is arguably of minor significance.

In consideration of the computational study of Houk and co-workers mentioned above,^61,62^ complex **34** is almost certainly an off-cycle intermediate; one of the ligands **L1** must be replaced by the aldehyde in order for productive C–C bond formation to take place. We computed this critically important ligand exchange and found it to be highly unfavorable (Scheme 13). Specifically, the required heteroleptic complex **K** turned out to be 5.0 kcal·mol^–1^ less stable than (*R,R*)-**M** if one takes the simultaneously generated, putative **L1**-free complex **Q** into account;^77^ the concentration of the “loaded” catalyst in the pre-equilibrium is hence marginal. NMR data strongly corroborate this conclusion in that addition of benzaldehyde to the solution of **34** does not entail any spectral change. Considering the size of **L1**, the exceptional stability of a nickel complex carrying two such ligands is striking. When the same ligand exchange was computed starting from the heterochiral complex (*S,R*)-**M** comprising two ligands **L1** of opposite configuration, the enthalpic penalty for forming **K** is only 2.1 kcal·mol^–1^. Once again, indirect evidence for this conclusion comes from NMR: the spectra recorded when *rac*-**L1** was used to generate complex **34** were superimposable with those obtained with enantiopure (≥98% ee) (*R*)-**L1**, which implies that no detectable amount of the diastereoisomeric complex (*S,R*)-**34** can be present in solution. If the homochiral complex (*R,R*)-**M** is indeed considerably more stable than its heterochiral sibling (*S,R*)-**M**, then the optical purity of the ligand **L1** passing through the catalytic cycle is deprived by the pre-equilibrium and a negative nonlinear effect ((−)-NLE) is expected to ensue.^78–81^

**Scheme 13:**
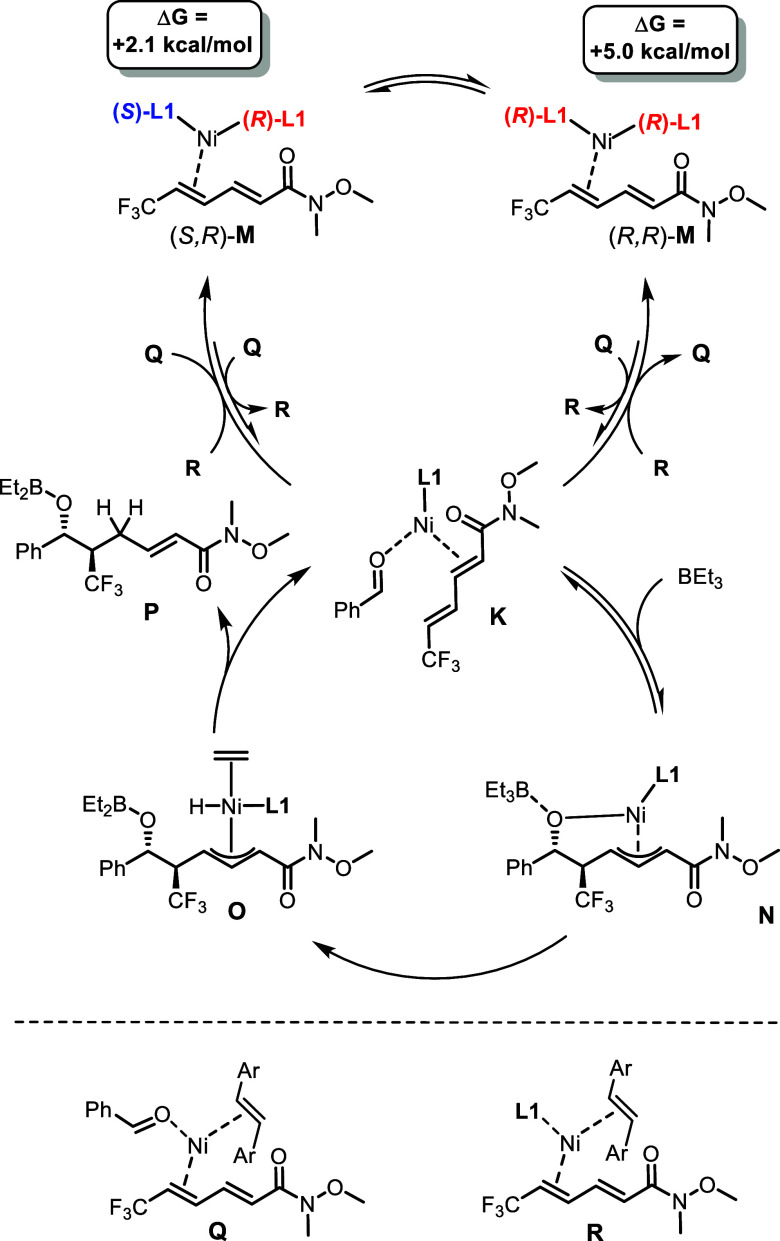
Proposed Catalytic Cycle and Pre-Equilibria

To the best of our knowledge, only a single study on nickel-catalyzed reductive coupling reactions has so far questioned if nonlinear effects are operative in such transformations but did not find any; rather, a strictly linear relationship between the ee of the specific chiral NHC ligand that was used and the product was observed.^15^ In stark contrast to this literature precedent and in perfect agreement with the conclusion drawn from our DFT study, the reaction of difluorosorbamide **15** with 3-methylbutanal catalyzed by Ni(0)/**L1** unmistakably shows a (−)-NLE (Figure 5). In this context, it is also important to remember that **L1**-free nickel complexes do not catalyze the reductive coupling of **15** at all (see above); therefore, the observed effect cannot becaused or confounded by a racemic background reaction. Moreover, varying the **L1**/Ni­(0) ratio altered the attained absolute ee values of the products, but the (−)-NLE persisted (for details, see the Supporting Information). These observations are taken as a strong argument in favor of the proposed mechanistic scenario. It confirms a central claim originally made by Houk and co-workers that nickel-catalyzed reductive coupling reactions, when performed in the presence of phosphines (and obviously also the phosphoramidite **L1** used herein), are interfaced with a pre-equilibrium that (substantially) depletes the concentration of the active species in solution.^61,62^

**Figure 5:**
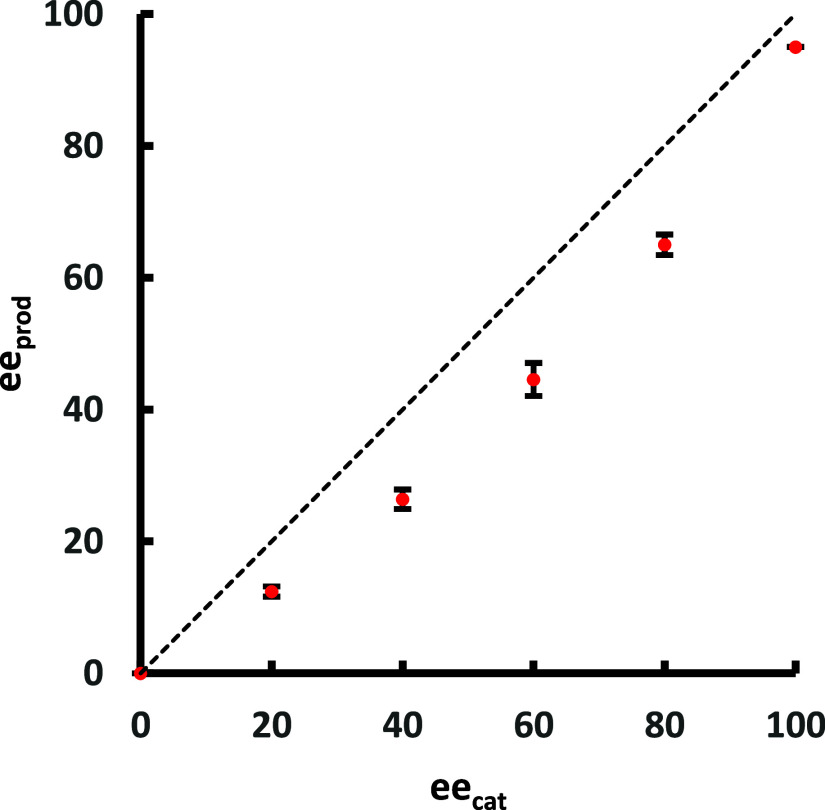
Negative nonlinear effect observed in the nickel-catalyzed coupling of difluorinated sorbamide **15** with 3-methylbutanal; each data point was determined by three independent measurements.

The excellent accord between experimental and computational data made us confident that DFT would also provide valuable insights into how **L1** exerts its effect during the actual coupling step. To this end, all pathways were computed, leading to eight possible regio- and stereoisomeric products that can be formed upon reductive coupling of trifluorinated sorbamide **12** with benzaldehyde (for the full data set, see the Supporting Information). To this end, the geometry of all stationary points was optimized at the PBE-D4/def2-SVP/CPCM­(toluene) level^69–72^ before the electronic energies of the stationary points were refined at the WB97X-D4rev/def2-QZVP/CPCM­(toluene)^82^ level of theory using the RIJCOSX approximation.^83^ In addition, the energies of the rate-determining transition states were recalculated using several combinations of functionals and basis sets (see the Supporting Information). At this point, however, we cannot help but emphasize that the results should be interpreted with caution with respect to the absolute kinetics owing to the well-known limitations of standard exchange–correlation functionals in accurately describing first-row transition metals such as nickel. Details apart, the energy difference between the competing pathways remained fairly constant across the different levels of theory, which shows that the predicted sense of asymmetric induction is fully robust and its magnitude correctly approximated.

In accord with the experimental data, the pathway leading to the (*R,R*)-configured *anti*-isomer **17a** has the lowest-lying transition state of all (Figure 6). Although the “loaded” complex in which the nickel center is bound to the proximal double bond is considerably more stable than the one where it ligates the trifluoromethylated alkene, the latter species is transiently formed as a pretransition state intermediate prior to productive C–C-bond formation. This, in turn, is consistent with our experimental observations showing that the binding site in the ground state of the nickel diene complex does not necessarily have to be the same as the site where the reaction ultimately takes place.

**Figure 6:**
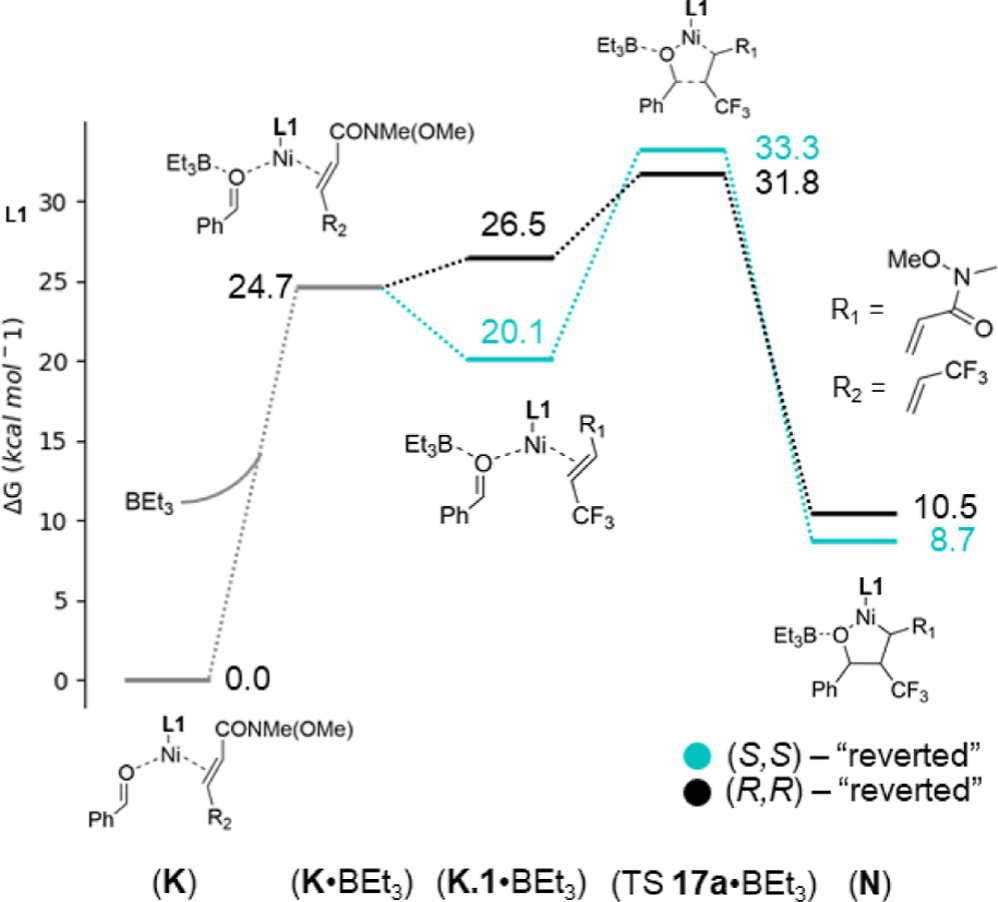
Reaction pathways of the reverted nickel-catalyzed coupling of benzaldehyde with trifluoromethylated sorbamide **12** leading to the experimentally observed product (*R,R*)-**17a** and the enantiomer thereof.

As mentioned above, the computational study by Houk and co-workers on the nickel-catalyzed reductive coupling of alkynes with aldehydes had reached the conclusion that BEt_3_ as the promoter comes into play only after the key nickelacycle intermediate has been formed. In the present case, however, a small but significant barrier lowering was observed when the pathways leading to the *anti*-configured enantiomeric products (*R,R*)-**17a** and (*S,S*)-**17a** were computed with BEt_3_ ligated to the aldehyde (Figure 6). The preference for (*R,R*)-**17a** remains unchanged and the computed ΔΔ*G* ^*⧧*^ = 1.5 kcal·mol^–1^ is in good qualitative agreement with the experimentally observed optical purity of product **17a** (86% ee).

The phosphoramidite **L1** stabilizes the transition state (TS) leading to the major product isomer (*R,R*)-**17a** through multiple π–π and σ-π contacts between the diene chain and the large surface of one phenyl-phenanthrene wing of this axially chiral ligand (Figure 7). For a more detailed understanding, an ADLD­(D4) analysis (Atomic Decomposition of London Dispersion) was performed.^84,85^ When selecting a subset of atoms of one of the ligand’s phenanthrene wings and the diene chain, attractive dispersion is markedly stronger in the TS leading to (*R,R*)-**17a** as the major product due to their proximity, to the extent that it is 1.7 kcal·mol^–1^ lower in energy (for details, see the Supporting Information). These attractive interactions are markedly reduced in the TS that connects to the minor enantiomer (*S,S*)-**17a** and almost entirely missing in all pathways leading to the regioisomeric aldol-type “Tamaru” products because the diene then points away from **L1**. The favorable stabilizing situation is the same whether BEt_3_ is included prior to nickelacycle formation or not. One can hence conclude that the extended π-surface area of the VAPOL-derived ligand **L1** is a key factor for the reversal of the regiochemical course of the coupling reaction as well as a critical determinant for achieving high levels of asymmetric induction.

**Figure 7:**
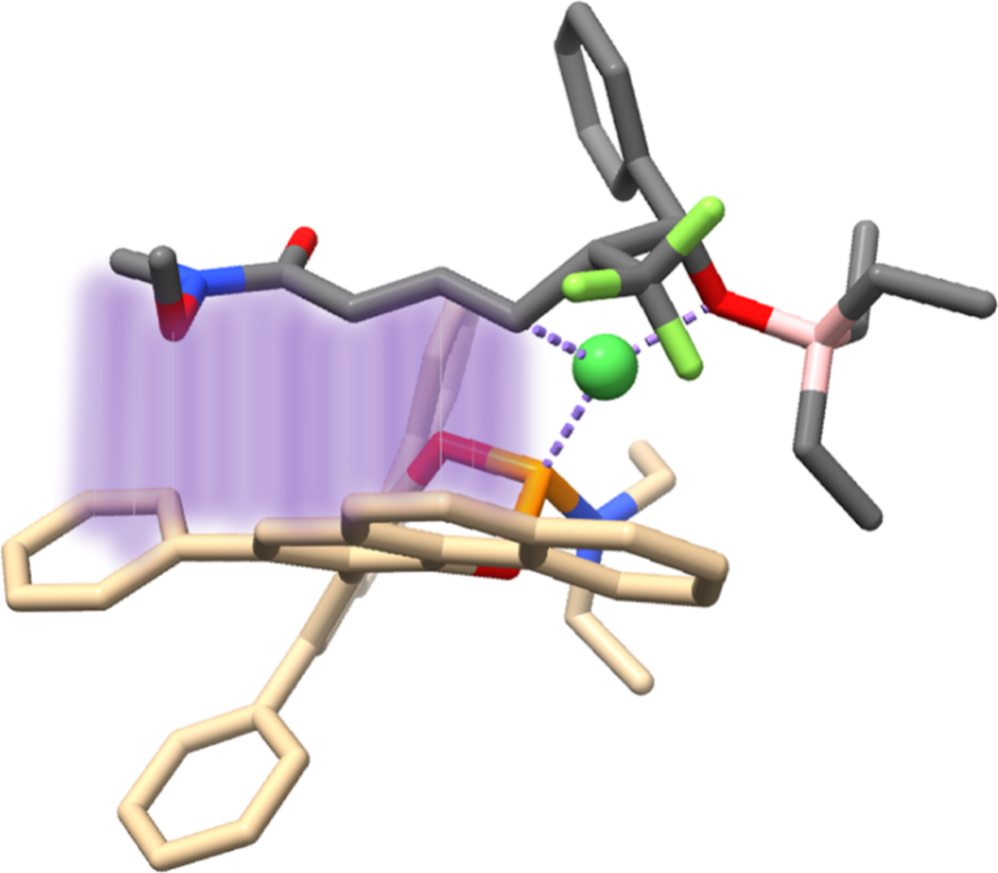
Transition state leading to the formation of the (*R,R*)-configured *anti*-isomer **17a** with BEt_3_ included, which coordinates to a lone pair of the benzaldehyde carbonyl group; the region shaded in purple features multiple π–π and σ–π contacts between the diene chain of the trifluorinated sorbamide **12** and the large surface of one of the phenyl-phenanthrene wings of the phosphoramidite ligand **L1**.

## Conclusions

The nickel-catalyzed reductive coupling of aldehydes with appropriately fluorinated sorbamide derivatives allows chiral centers with a –CF_2_H, –CF_3_, –CF_2_Me, or –CH_2_CF_3_ substituent to be generated from prochiral substrates. To meet this difficult task and ensure high levels of asymmetric induction, the use of the axially chiral, VAPOL-derived phosphoramidite ligand **L1** is quintessential. The resulting products represent valuable fluorinated (bio)­isosteres of a common type of *anti*-configured deoxypropionates and analogues; therefore, the new transformation qualifies for a “fluoride scan” of compounds comprising this important structural motif. As such, it nicely complements our efforts at developing catalytic asymmetric approaches to valuable fluorinated building blocks that are not readily accessible otherwise.^86–88^

A combined experimental/computational study has provided valuable insights into why the VAPOL-derived phosphoramidite **L1** is currently the only known ligand that is able to induce catalytic asymmetric C–C coupling reactions of functionalized 1,3-dienes following a “reverted” course. When added to the Ni(0) precatalyst and trifluorinated sorbamide, a highly stable complex of type [(**L1**)_2_Ni­(1,3-diene)] is formed, which is a “reservoir” off the actual catalytic cycle. This notion is clearly manifested in spectroscopic and computational data; it also explains the negative nonlinear effect ((−)-NLE) between the optical purity of **L1** and the resulting product. The “reverted” coupling pattern and asymmetric induction originate from multiple π–π and σ–π contacts between the diene chain of the polarized 1,3-diene substrate and the large surface of one phenyl-phenanthrene wing of the phosphoramidite ligand **L1**, which entail a favorable orientation of the coupling partner in the regio- and stereo-determining transition state. Although many details remain yet to be elucidated, these mechanistic insights are deemed a significant advance over the entirely empirical prior art that had resulted in the discovery of the remarkable functionality of **L1** and its use as a “game-changer” in nickel-catalyzed reductive diene/aldehyde coupling reactions. Ongoing projects aiming at expanding the scope of this chemistry are underway in our laboratory and will be reported in due course.

## Supplementary Material



## Notes

Experimental Section including crystallographic abstracts, characterization data, computational data, and NMR spectra of new compounds (PDF)
