# Supplementary material for: Nickel-Catalyzed Enantioselective Coupling Reactions of Fluorinated Sorbamides with Aldehydes Affording Anti-Configured β‑Di(tri)fluoromethyl Alcohol Derivatives
Source: J Am Chem Soc. 2026 Jun 24;148(26):27934–50. doi: 10.1021/jacs.6c09846 (PMC13352605; doi:10.1021/jacs.6c09846)
Supplement: Supplementary file 1 [file ja6c09846_si_001.pdf]

SUPPORTING INFORMATION

Nickel-Catalyzed Enantioselective Coupling Reactions of Fluorinated Sorbamides with Aldehydes Affording *Anti*-Configured β-Di(tri)fluoromethyl Alcohol Derivatives

Noah Richter,[a] Gianluca Regni,[b] Lorenzo Baldinelli,[b] Markus Leutzsch,[a] Giovanni Bistoni,[b] and Alois Fürstner[a],*

[a] *Max-Planck-Institut für Kohlenforschung, 45470 Mülheim/Ruhr, Germany*

[b] *Department of Chemistry, Biology, and Biotechnology, University of Perugia, I-06123 Perugia, Italy*

Email: fuerstner@kofo.mpg.de

Table of Contents

Supporting Crystallographic Information S2
Experimental S23
- General Information S23
- Substrates S24
- Reaction Optimization S29
- Control Experiments S30
- Determination of the Regioisomeric Ratio (rr) S31
- General Procedures S33
- Gram-Scale Reaction S35

Characterization Data S36
- Trifluoromethyl Derivatives S36
- Difluoromethyl Derivatives S41
- Products Derived from Other Fluorinated Dienamides S74

Applications S81
Non-linear Effect S84
Crystallization of the Nickel 1,3-Diene Complexes S86
Stoichiometric NMR-Experiments S86
Computational Study S95
Cartesian Coordinates S100
NMR Spectra S173
References S295

Supporting Crystallographic Information

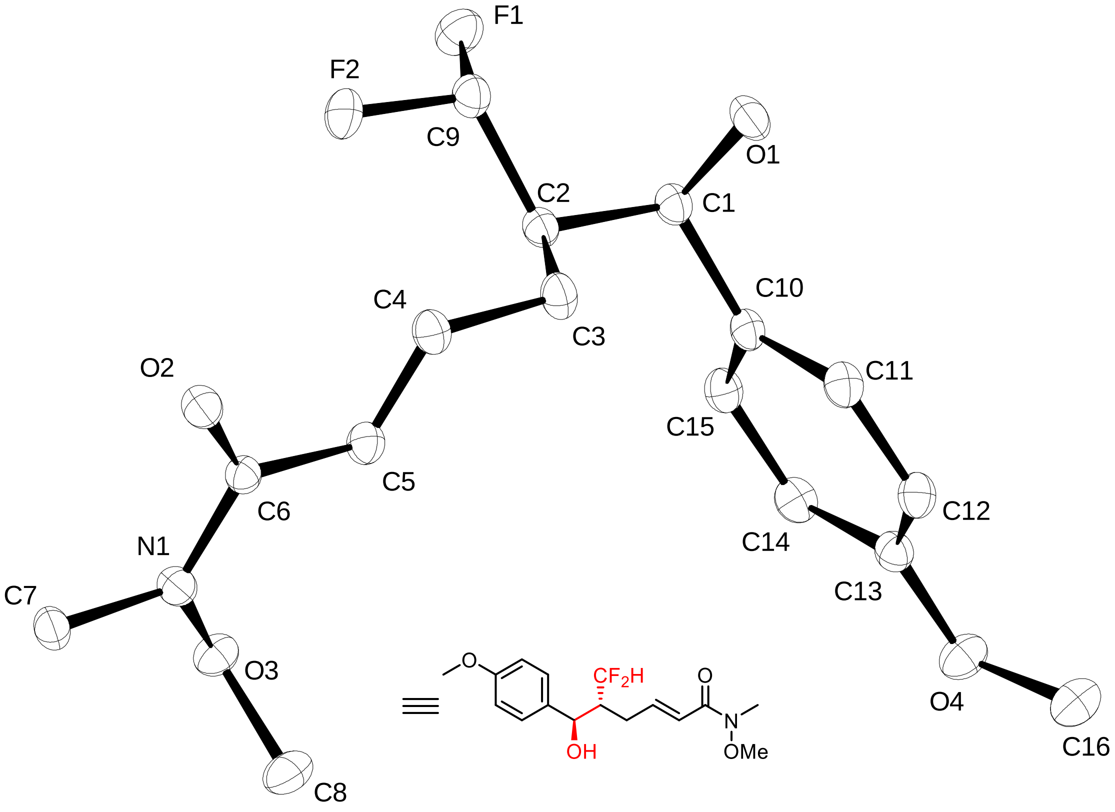

Figure S1. The asymmetric unit of compound **20e**; H-atoms have been removed for clarity.

$C_{16}H_{21}F_2NO_4$, M_r = 329.34 g·mol^{-1}, clear colorless prism, crystal size 0.194 x 0.171 x 0.121 mm^3, orthorhombic, space group *P2₁2₁2₁* [19], a = 5.395(9) Å, b = 15.32(3) Å, c = 18.73(3) Å, V = 1548(5) Å^3, T = 100(2) K, Z = 4, D_{calc} = 1.413 g·cm^3, λ = 0.71073 Å, $\mu(Mo\text{-}K_\alpha)$ = 0.116 mm^{-1}, Gaussian absorption correction (T_{min} = 0.95, T_{max} = 1.00), Bruker AXS D8-Venture diffractometer with IμS Diamond Mo-anode X-ray source and PHOTON III detector, 2.549 < θ < 31.738°, 99945 measured reflections, 5216 independent reflections, 4778 reflections with $I > 2\sigma(I)$, R_{int} = 0.0598, absolute structure parameter = -0.12(15). The structure was solved by *SHELXT* and refined by full-matrix least-squares (*SHELXL*) against F^2 to R_1 = 0.0317 [$I > 2\sigma(I)$], wR_2 = 0.0844, 227 parameters. **CCDC-2522804.**

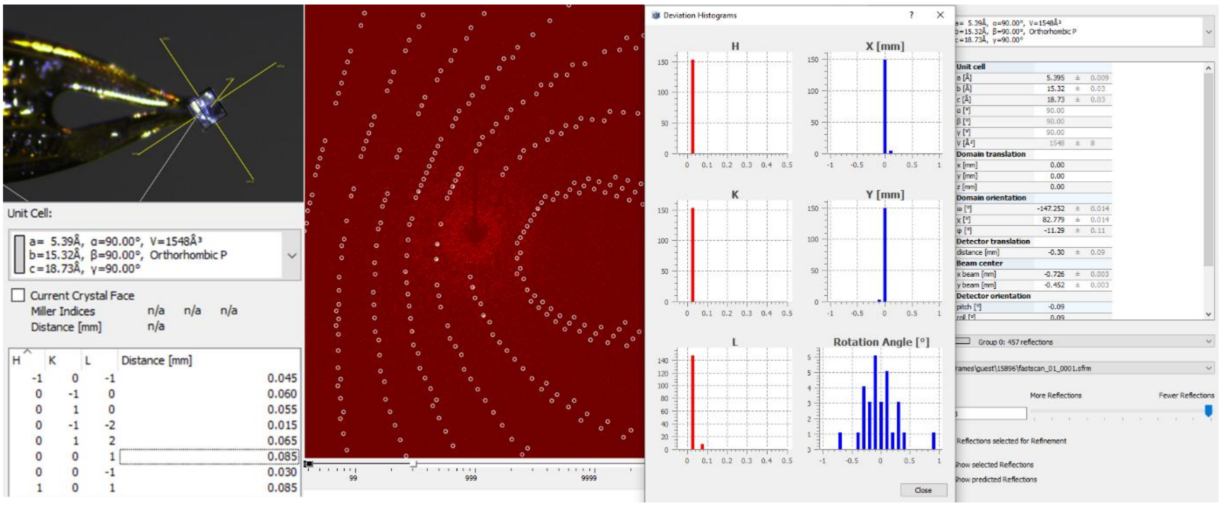

Figure S2. Crystal faces and unit cell determination/refinement of compound **20e**

INTENSITY STATISTICS FOR DATASET

Resolution	#Data	#Theory	%Complete	Redundancy	Mean I	Mean I/s	Rmerge	Rsigma
Inf - 2.69	79	83	95.2	22.14	177.16	98.42	0.0296	0.0144
2.69 - 1.85	186	186	100.0	30.72	68.64	104.53	0.0252	0.0076
1.85 - 1.47	263	263	100.0	31.43	33.78	88.37	0.0363	0.0089
1.47 - 1.28	269	269	100.0	29.66	21.31	69.56	0.0499	0.0115
1.28 - 1.16	272	272	100.0	27.05	20.33	59.39	0.0550	0.0127
1.16 - 1.07	278	278	100.0	22.81	19.47	52.03	0.0568	0.0145
1.07 - 1.01	272	272	100.0	21.58	13.53	42.18	0.0737	0.0185
1.01 - 0.96	258	258	100.0	20.39	10.04	32.11	0.0890	0.0232
0.96 - 0.92	244	244	100.0	19.26	7.06	26.68	0.1092	0.0306
0.92 - 0.88	321	321	100.0	17.62	5.69	21.79	0.1329	0.0379
0.88 - 0.85	268	268	100.0	17.68	5.52	20.34	0.1375	0.0393
0.85 - 0.83	195	195	100.0	17.30	4.91	18.28	0.1545	0.0439
0.83 - 0.80	339	339	100.0	16.03	4.10	15.58	0.1776	0.0547
0.80 - 0.78	232	232	100.0	15.80	3.91	14.78	0.1900	0.0583
0.78 - 0.76	299	299	100.0	14.99	4.04	13.97	0.1903	0.0597
0.76 - 0.75	162	162	100.0	14.37	3.92	12.80	0.1792	0.0633
0.75 - 0.73	339	339	100.0	13.77	4.04	12.01	0.1967	0.0649
0.73 - 0.71	365	365	100.0	13.70	2.75	9.48	0.2518	0.0908
0.71 - 0.70	180	180	100.0	13.02	2.95	9.37	0.2452	0.0894
0.70 - 0.69	220	220	100.0	12.95	3.37	10.23	0.2309	0.0836
0.69 - 0.68	202	205	98.5	11.86	2.99	8.26	0.2702	0.1051
0.78 - 0.68	1767	1770	99.8	13.62	3.45	10.97	0.2164	0.0764
Inf - 0.68	5243	5250	99.9	19.10	13.92	32.46	0.0588	0.0211

Table S1. Crystal data and structure refinement of compound **20e**

Empirical formula	$C_{16}H_{21}F_2NO_4$	
Color	clear colourless	
Formula weight	329.34 g · mol^{-1}	
Temperature	100(2) K	
Wavelength	0.71073 Å	
Crystal system	ORTHORHOMBIC	
Space group	**$P2_12_12_1$, (no. 19)**	
Unit cell dimensions	a = 5.395(9) Å	α= 90°.
	b = 15.32(3) Å	β= 90°.
	c = 18.73(3) Å	γ = 90°.
Volume	1548(5) Å^3	
Z	4	
Density (calculated)	1.413 Mg · m^{-3}	
Absorption coefficient	0.116 mm^{-1}	
F(000)	696 e	
Crystal size	0.194 x 0.171 x 0.121 mm^3	
θ range for data collection	2.549 to 31.738°.	
Index ranges	$-7 \le h \le 7$, $-19 \le k \le 22$, $-27 \le l \le 27$	
Reflections collected	99945	
Independent reflections	5216 [R_{int} = 0.0598]	
Reflections with I>2σ(I)	4778	
Completeness to θ = 25.242°	99.9 %	
Absorption correction	Numerical	
Max. and min. transmission	1.00 and 0.95	
Refinement method	Full-matrix least-squares on F^2	
Data / restraints / parameters	5216 / 0 / 227	
Goodness-of-fit on F^2	1.039	
Final R indices [I>2σ(I)]	R_1 = 0.0317	wR^2 = 0.0803
R indices (all data)	R_1 = 0.0370	wR^2 = 0.0844
Absolute structure parameter	-0.12(15)	
Largest diff. peak and hole	0.3 and -0.2 e · Å^{-3}	

Table S2. Bond lengths [Å] and angles [°] of compound **20e**

F(1)-C(9)	1.367(2)	F(2)-C(9)	1.361(2)
O(1)-H(1)	0.87(3)	O(1)-C(1)	1.422(3)
O(2)-C(6)	1.233(2)	O(3)-N(1)	1.393(2)
O(3)-C(8)	1.433(3)	O(4)-C(13)	1.364(2)
O(4)-C(16)	1.421(3)	N(1)-C(6)	1.349(2)
N(1)-C(7)	1.438(2)	C(1)-H(1A)	0.977(19)
C(1)-C(2)	1.533(3)	C(1)-C(10)	1.503(3)
C(2)-H(2)	1.01(2)	C(2)-C(3)	1.535(3)
C(2)-C(9)	1.502(3)	C(3)-C(4)	1.496(3)
C(4)-C(5)	1.322(3)	C(5)-C(6)	1.472(3)
C(10)-C(11)	1.384(3)	C(10)-C(15)	1.393(2)
C(11)-C(12)	1.388(3)	C(12)-C(13)	1.382(2)
C(13)-C(14)	1.387(3)	C(14)-C(15)	1.379(3)
C(1)-O(1)-H(1)	107(2)	N(1)-O(3)-C(8)	109.43(11)
C(13)-O(4)-C(16)	117.37(13)	O(3)-N(1)-C(7)	115.07(15)
C(6)-N(1)-O(3)	119.04(12)	C(6)-N(1)-C(7)	124.82(13)
O(1)-C(1)-H(1A)	109.4(12)	O(1)-C(1)-C(2)	106.73(14)
O(1)-C(1)-C(10)	112.40(12)	C(2)-C(1)-H(1A)	108.3(12)
C(10)-C(1)-H(1A)	105.5(11)	C(10)-C(1)-C(2)	114.36(13)
C(1)-C(2)-H(2)	110.1(11)	C(1)-C(2)-C(3)	112.24(14)
C(3)-C(2)-H(2)	108.7(11)	C(9)-C(2)-C(1)	108.87(13)
C(9)-C(2)-H(2)	106.2(11)	C(9)-C(2)-C(3)	110.56(16)
C(4)-C(3)-C(2)	114.53(15)	C(5)-C(4)-C(3)	123.69(14)
C(4)-C(5)-C(6)	120.83(14)	O(2)-C(6)-N(1)	119.42(13)
O(2)-C(6)-C(5)	123.36(16)	N(1)-C(6)-C(5)	117.19(13)
F(1)-C(9)-C(2)	110.84(16)	F(1)-C(9)-H(9)	108.8(11)
F(2)-C(9)-F(1)	105.01(15)	F(2)-C(9)-C(2)	110.81(13)
F(2)-C(9)-H(9)	108.1(12)	C(2)-C(9)-H(9)	113.0(11)
C(11)-C(10)-C(1)	122.50(14)	C(11)-C(10)-C(15)	118.21(14)
C(15)-C(10)-C(1)	119.22(13)	C(10)-C(11)-C(12)	121.49(14)
C(13)-C(12)-C(11)	119.23(14)	O(4)-C(13)-C(12)	124.61(14)
O(4)-C(13)-C(14)	115.12(14)	C(12)-C(13)-C(14)	120.27(15)
C(15)-C(14)-C(13)	119.75(14)	C(14)-C(15)-C(10)	121.01(14)

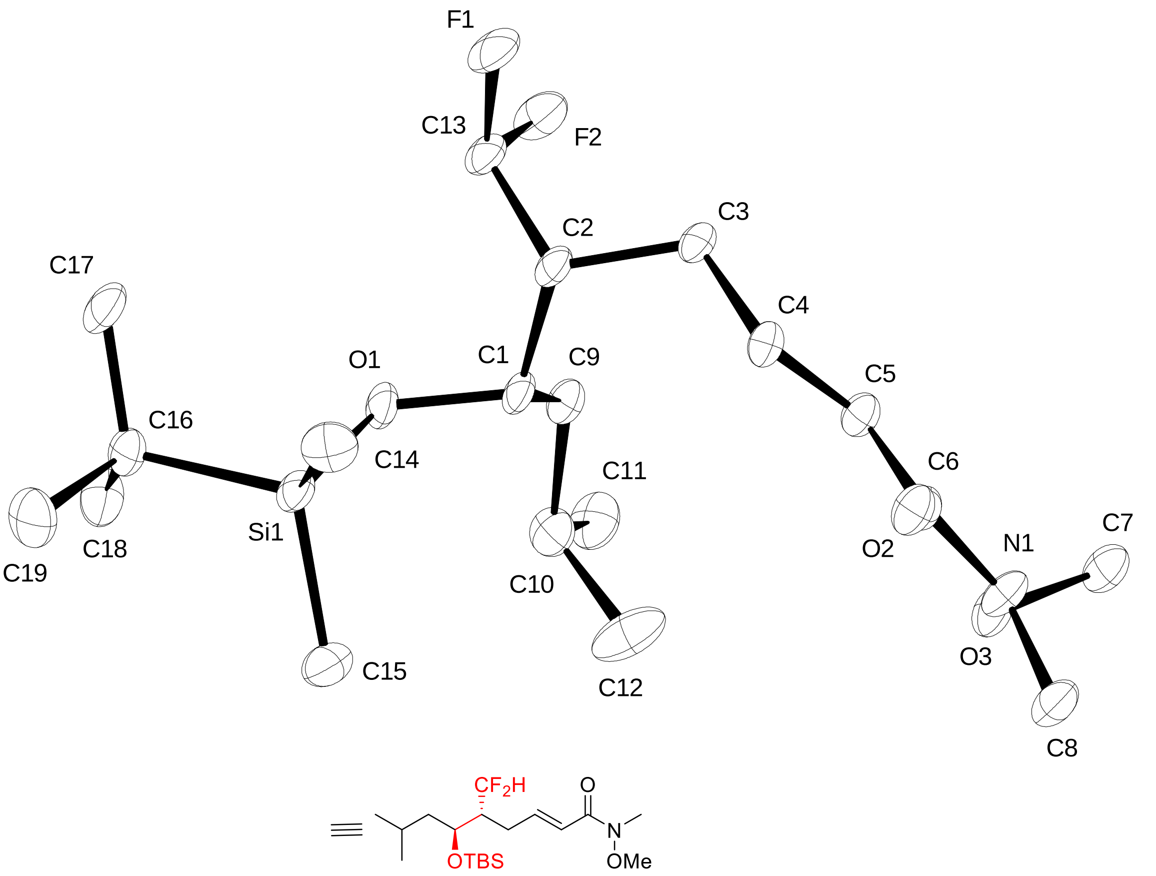

Figure S3. The asymmetric unit of compound **28**; H-atoms have been removed for clarity.

$C_{19}H_{37}F_2NO_3Si$, M_r = 393.58 g·mol^{-1}, colorless needle, crystal size 0.300 x 0.073 x 0.041 mm^3, monoclinic, space group $P2_1$ [4], a = 10.6572(4) Å, b = 6.4569(2) Å, c = 16.7040(6) Å, β = 103.488(2)°, V = 1117.74(7) Å^3, T = 100(2) K, Z = 2, D_{calc} = 1.169 g·cm^3, λ = 0.71073 Å, $\mu(Mo\text{-}K_\alpha)$ = 0.138 mm^{-1}, Gaussian absorption correction (T_{min} = 0.98, T_{max} = 1.00), Bruker AXS D8-Venture diffractometer with IµS Diamond Mo-anode X-ray source and PHOTON III detector, 1.965 < θ < 28.281°, 64258 measured reflections, 5557 independent reflections, 4864 reflections with $I > 2\sigma(I)$, R_{int} = 0.1453, absolute structure parameter = -0.13(10). The structure was solved by *SHELXT* and refined by full-matrix least-squares (*SHELXL*) against F^2 to R_1 = 0.0450 [$I > 2\sigma(I)$], wR_2 = 0.1008, 260 parameters. **CCDC-2522802**

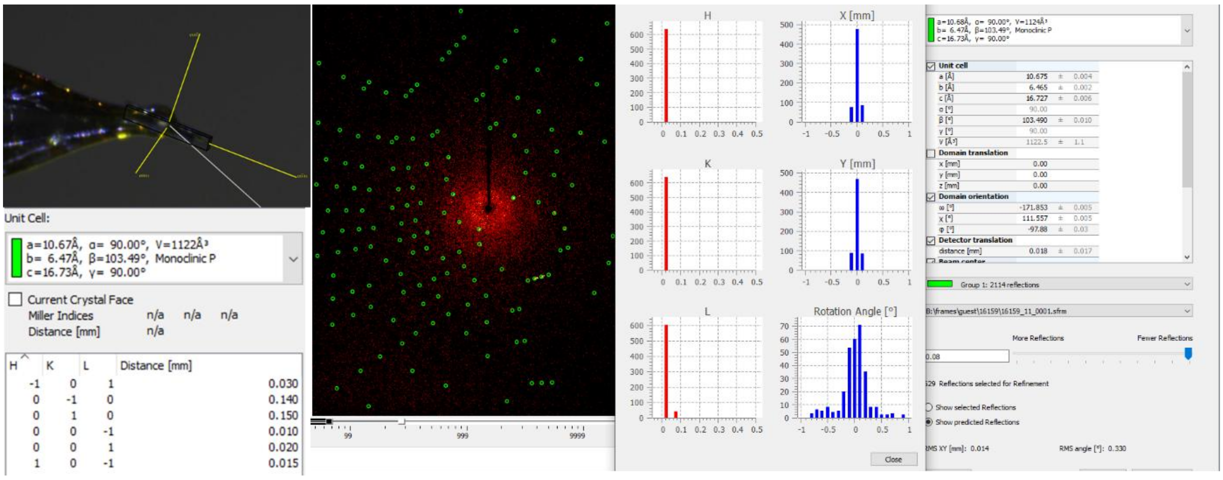

Figure S4. Crystal faces and unit cell determination/refinement of compound **28**

INTENSITY STATISTICS FOR DATASET

Resolution	#Data	#Theory	%Complete	Redundancy	Mean I	Mean I/s	Rmerge	Rsigma
Inf - 2.88	104	105	99.0	13.37	125.31	25.76	0.0404	0.0568
2.88 - 1.90	245	245	100.0	14.90	49.32	24.51	0.0622	0.0354
1.90 - 1.50	352	352	100.0	14.23	29.60	21.07	0.0923	0.0402
1.50 - 1.31	342	342	100.0	14.37	19.15	18.09	0.1293	0.0456
1.31 - 1.19	353	353	100.0	13.86	18.50	16.99	0.1409	0.0488
1.19 - 1.10	364	364	100.0	13.93	13.66	14.45	0.1691	0.0551
1.10 - 1.04	313	313	100.0	13.11	9.41	12.00	0.2134	0.0677
1.04 - 0.99	344	344	100.0	13.06	8.80	11.01	0.2270	0.0706
0.99 - 0.94	399	399	100.0	11.73	6.73	9.37	0.2641	0.0878
0.94 - 0.91	289	289	100.0	10.87	5.52	7.70	0.2976	0.1004
0.91 - 0.87	477	477	100.0	10.20	4.97	7.58	0.3234	0.1112
0.87 - 0.85	238	238	100.0	10.08	4.65	7.19	0.3323	0.1191
0.85 - 0.82	427	427	100.0	9.76	3.87	6.05	0.3621	0.1373
0.82 - 0.80	324	324	100.0	9.29	4.05	6.13	0.3743	0.1412
0.80 - 0.78	370	370	100.0	8.85	3.12	5.07	0.4238	0.1741
0.78 - 0.77	201	201	100.0	8.46	2.87	4.59	0.4168	0.1920
0.77 - 0.75	423	423	100.0	8.35	3.09	4.76	0.4067	0.1880
0.75 - 0.73	437	437	100.0	7.79	2.63	4.10	0.4581	0.2262
0.73 - 0.72	268	268	100.0	6.90	2.50	3.68	0.4626	0.2592
0.72 - 0.71	275	275	100.0	6.87	2.33	3.46	0.4572	0.2888
0.71 - 0.70	315	321	98.1	6.04	2.42	3.21	0.4581	0.3259
0.80 - 0.70	2289	2295	99.7	7.66	2.73	4.17	0.4360	0.2275
Inf - 0.70	6860	6867	99.9	10.69	11.16	9.69	0.1511	0.0721

Table S3. Crystal data and structure refinement of compound **28**

Empirical formula	$C_{19}H_{37}F_2NO_3Si$	
Color	colourless	
Formula weight	393.58 g · mol^{-1}	
Temperature	100(2) K	
Wavelength	0.71073 Å	
Crystal system	MONOCLINIC	
Space group	**$P2_1$, (no. 4)**	
Unit cell dimensions	a = 10.6572(4) Å	α= 90°.
	b = 6.4569(2) Å	β= 103.488(2)°.
	c = 16.7040(6) Å	γ = 90°.
Volume	1117.74(7) Å^3	
Z	2	
Density (calculated)	1.169 Mg · m^{-3}	
Absorption coefficient	0.138 mm^{-1}	
F(000)	428 e	
Crystal size	0.300 x 0.073 x 0.041 mm^3	
θ range for data collection	1.965 to 28.281°.	
Index ranges	-14 ≤ h ≤ 14, -8≤ k ≤ 8, -22 ≤ l ≤ 22	
Reflections collected	64258	
Independent reflections	5557 [R_{int} = 0.1453]	
Reflections with I>2σ(I)	4864	
Completeness to θ = 25.242°	100.0 %	
Absorption correction	Gaussian	
Max. and min. transmission	1.00 and 0.98	
Refinement method	Full-matrix least-squares on F^2	
Data / restraints / parameters	5557 / 1 / 260	
Goodness-of-fit on F^2	1.027	
Final R indices [I>2σ(I)]	R_1 = 0.0450	wR^2 = 0.0962
R indices (all data)	R_1 = 0.0542	wR^2 = 0.1008
Absolute structure parameter	-0.13(10)	
Largest diff. peak and hole	0.3 and -0.3 e · Å^{-3}	

Table S4. Bond lengths [Å] and angles [°] of compound **28**

Si(1)-O(1)	1.652(2)	Si(1)-C(14)	1.863(3)
Si(1)-C(15)	1.863(3)	Si(1)-C(16)	1.881(3)
F(1)-C(13)	1.376(3)	F(2)-C(13)	1.371(3)
O(1)-C(1)	1.442(3)	O(2)-C(6)	1.224(3)
O(3)-N(1)	1.407(3)	O(3)-C(7)	1.436(4)
N(1)-C(6)	1.371(3)	N(1)-C(8)	1.446(4)
C(1)-H(1)	0.94(3)	C(1)-C(2)	1.547(4)
C(1)-C(9)	1.517(4)	C(2)-H(2)	1.04(3)
C(2)-C(3)	1.534(4)	C(2)-C(13)	1.506(4)
C(3)-C(4)	1.499(4)	C(4)-C(5)	1.332(4)
C(5)-C(6)	1.480(4)	C(9)-C(10)	1.534(4)
C(10)-H(10)	0.97(4)	C(10)-C(11)	1.528(4)
C(10)-C(12)	1.516(5)	C(13)-H(13)	0.93(3)
C(16)-C(17)	1.534(4)	C(16)-C(18)	1.539(4)
C(16)-C(19)	1.538(4)		
O(1)-Si(1)-C(14)	111.02(13)	O(1)-Si(1)-C(15)	111.45(13)
O(1)-Si(1)-C(16)	103.73(12)	C(14)-Si(1)-C(15)	108.54(15)
C(14)-Si(1)-C(16)	111.40(15)	C(15)-Si(1)-C(16)	110.68(15)
C(1)-O(1)-Si(1)	126.62(17)	N(1)-O(3)-C(7)	109.5(2)
O(3)-N(1)-C(8)	113.9(2)	C(6)-N(1)-O(3)	117.8(2)
C(6)-N(1)-C(8)	122.1(2)	O(1)-C(1)-H(1)	107.9(17)
O(1)-C(1)-C(2)	108.4(2)	O(1)-C(1)-C(9)	108.6(2)
C(2)-C(1)-H(1)	105.4(17)	C(9)-C(1)-H(1)	112.4(18)
C(9)-C(1)-C(2)	113.9(2)	C(1)-C(2)-H(2)	107.4(16)
C(3)-C(2)-C(1)	113.6(2)	C(3)-C(2)-H(2)	105.5(17)
C(13)-C(2)-C(1)	111.9(2)	C(13)-C(2)-H(2)	106.6(18)
C(13)-C(2)-C(3)	111.2(2)	C(4)-C(3)-C(2)	112.8(2)
C(5)-C(4)-C(3)	124.6(3)	C(4)-C(5)-C(6)	119.6(3)
O(2)-C(6)-N(1)	119.4(3)	O(2)-C(6)-C(5)	123.5(2)
N(1)-C(6)-C(5)	117.0(2)	C(1)-C(9)-C(10)	115.9(2)
C(9)-C(10)-H(10)	110(2)	C(11)-C(10)-C(9)	108.8(3)
C(11)-C(10)-H(10)	111(2)	C(12)-C(10)-C(9)	112.3(3)
C(12)-C(10)-H(10)	104(2)	C(12)-C(10)-C(11)	109.9(3)
F(1)-C(13)-C(2)	110.2(2)	F(1)-C(13)-H(13)	108(2)
F(2)-C(13)-F(1)	105.0(2)	F(2)-C(13)-C(2)	112.3(2)
F(2)-C(13)-H(13)	103(2)	C(2)-C(13)-H(13)	117.0(19)
C(17)-C(16)-Si(1)	110.4(2)	C(17)-C(16)-C(18)	109.1(3)
C(17)-C(16)-C(19)	108.3(2)	C(18)-C(16)-Si(1)	109.2(2)
C(19)-C(16)-Si(1)	110.6(2)	C(19)-C(16)-C(18)	109.3(3)

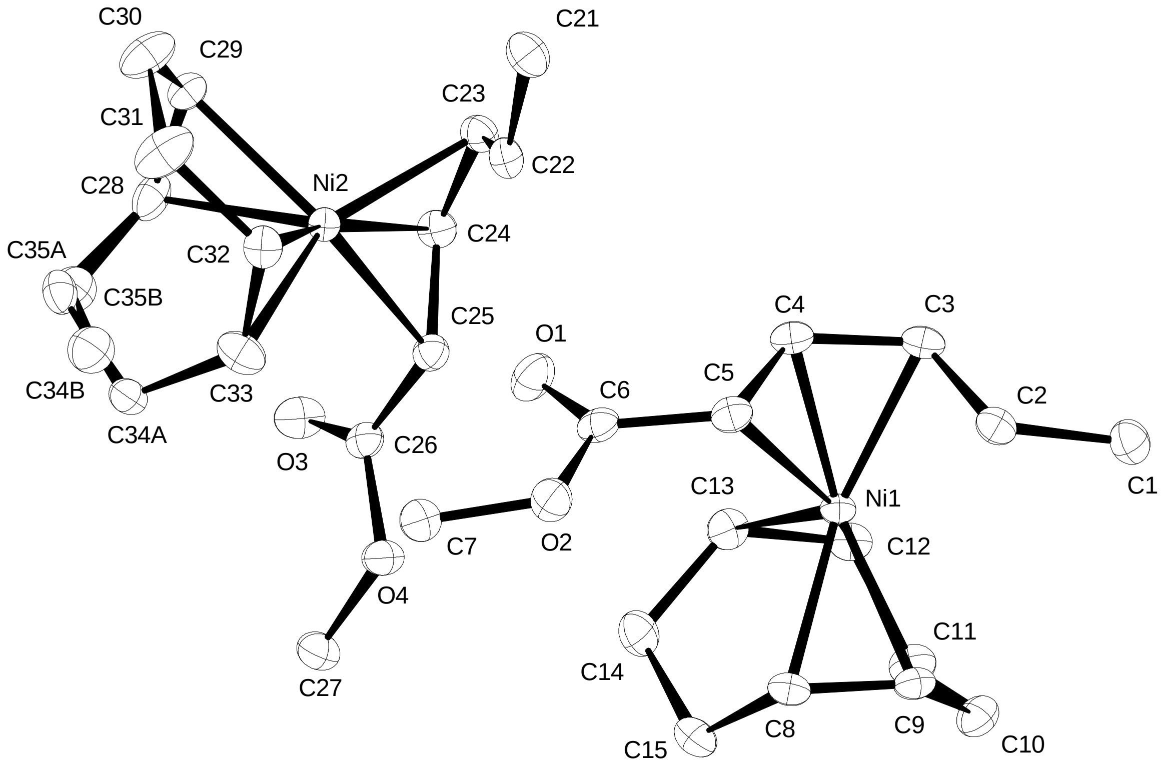

Figure S5. The asymmetric unit of complex **32**; H-atoms have been removed for clarity.

$C_{15}H_{22}NiO_2$, M_r = 293.03 g·mol^{-1}, orange prism, crystal size 0.134 x 0.093 x 0.041 mm^3, triclinic, space group $P\bar{1}$ [2], a = 9.2560(3) Å, b = 11.2593(3) Å, c = 13.3298(4) Å, α = 97.217(2)°, β = 100.427(2)°, γ = 91.179(2)°, V = 1354.08(7) Å^3, T = 100(2) K, Z = 4, D_{calc} = 1.437 g·cm^3, λ = 0.71073 Å, μ(Mo-K_α) = 1.424 mm^{-1}, Gaussian absorption correction ($T_{\min}$ = 0.88, $T_{\max}$ = 0.96), Bruker AXS D8-Venture diffractometer with IµS Diamond Mo-anode X-ray source and PHOTON III detector, 1.825 < θ < 31.581°, 230048 measured reflections, 9089 independent reflections, 8090 reflections with $I > 2\sigma(I)$, R_{int} = 0.0646. The structure was solved by *SHELXT* and refined by full-matrix least-squares (*SHELXL*) against F^2 to R_1 = 0.0265 [$I > 2\sigma(I)$], wR_2 = 0.0726, 337 parameters. **CCDC-2522803**

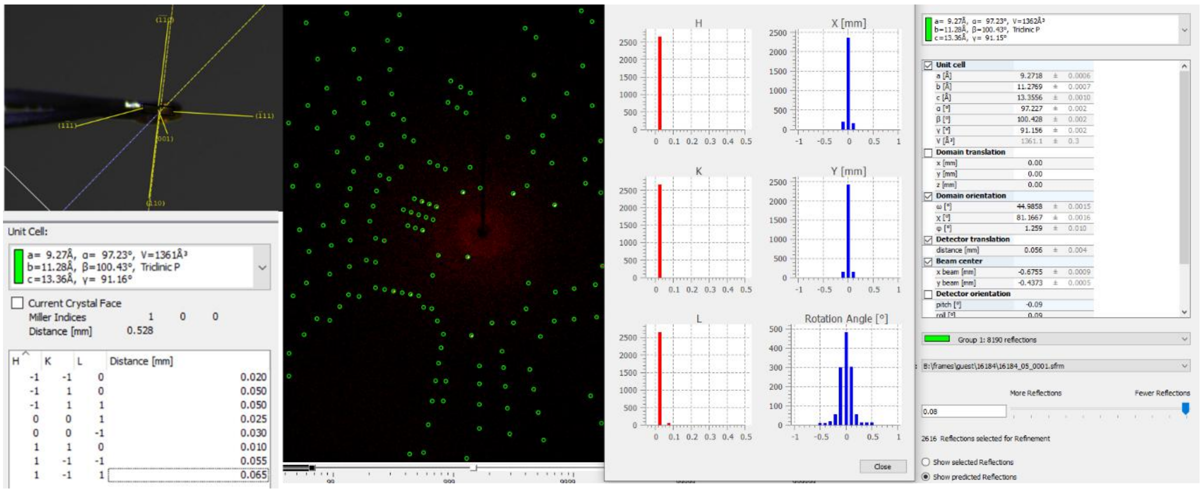

Figure S6. Crystal faces and unit cell determination/refinement of complex **32**

INTENSITY STATISTICS FOR DATASET

Resolution	#Data	#Theory	%Complete	Redundancy	Mean I	Mean I/s	Rmerge	Rsigma
Inf - 2.71	137	140	97.9	23.52	64.12	82.42	0.0306	0.0143
2.71 - 1.82	323	323	100.0	30.60	40.39	98.32	0.0299	0.0105
1.82 - 1.45	460	460	100.0	32.20	31.04	88.88	0.0315	0.0084
1.45 - 1.27	468	468	100.0	31.86	18.80	69.22	0.0417	0.0094
1.27 - 1.15	466	466	100.0	31.07	13.66	58.70	0.0528	0.0115
1.15 - 1.07	466	466	100.0	31.00	12.63	53.86	0.0569	0.0124
1.07 - 1.01	439	439	100.0	28.73	9.48	45.05	0.0689	0.0152
1.01 - 0.96	452	452	100.0	27.16	10.35	43.89	0.0666	0.0153
0.96 - 0.91	543	543	100.0	26.26	8.78	40.20	0.0792	0.0179
0.91 - 0.88	405	405	100.0	24.87	7.80	34.79	0.0850	0.0197
0.88 - 0.85	458	458	100.0	24.88	6.23	30.39	0.1007	0.0231
0.85 - 0.82	523	523	100.0	24.22	5.42	26.89	0.1128	0.0263
0.82 - 0.80	393	393	100.0	23.78	4.50	23.12	0.1255	0.0303
0.80 - 0.78	443	443	100.0	23.60	3.79	19.88	0.1377	0.0346
0.78 - 0.76	488	488	100.0	23.10	3.76	19.67	0.1416	0.0361
0.76 - 0.74	528	528	100.0	22.58	3.40	17.50	0.1519	0.0397
0.74 - 0.73	308	308	100.0	21.61	3.64	17.45	0.1539	0.0409
0.73 - 0.71	614	614	100.0	20.95	2.96	14.71	0.1755	0.0482
0.71 - 0.70	325	325	100.0	20.06	3.46	15.64	0.1710	0.0470
0.70 - 0.69	403	403	100.0	19.97	3.18	14.29	0.1723	0.0497
0.69 - 0.68	447	450	99.3	17.47	2.60	11.48	0.1933	0.0703
0.78 - 0.68	3113	3116	99.9	20.91	3.26	15.81	0.1627	0.0463
Inf - 0.68	9089	9095	99.9	25.29	10.25	37.34	0.0639	0.0173

Table S5. Crystal data and structure refinement of complex **32**

Empirical formula	$C_{15}H_{22}NiO_2$	
Color	orange	
Formula weight	293.03 g · mol^{-1}	
Temperature	100(2) K	
Wavelength	0.71073 Å	
Crystal system	TRICLINIC	
Space group	**P$\bar{1}$, (no. 2)**	
Unit cell dimensions	a = 9.2560(3) Å	α= 97.217(2)°.
	b = 11.2593(3) Å	β= 100.427(2)°.
	c = 13.3298(4) Å	γ = 91.179(2)°.
Volume	1354.08(7) Å^3	
Z	4	
Density (calculated)	1.437 Mg · m^{-3}	
Absorption coefficient	1.424 mm^{-1}	
F(000)	624 e	
Crystal size	0.134 x 0.093 x 0.041 mm^3	
θ range for data collection	1.825 to 31.581°.	
Index ranges	$-13 \le h \le 13$, $-16 \le k \le 16$, $-19 \le l \le 19$	
Reflections collected	230048	
Independent reflections	9089 [R_{int} = 0.0646]	
Reflections with I>2σ(I)	8090	
Completeness to θ = 25.242°	99.9 %	
Absorption correction	Gaussian	
Max. and min. transmission	0.96 and 0.88	
Refinement method	Full-matrix least-squares on F^2	
Data / restraints / parameters	9089 / 0 / 337	
Goodness-of-fit on F^2	1.046	
Final R indices [I>2σ(I)]	R_1 = 0.0265	wR^2 = 0.0693
R indices (all data)	R_1 = 0.0314	wR^2 = 0.0726
Largest diff. peak and hole	0.9 and -0.7 e · Å^{-3}	

Table S6. Bond lengths [Å] and angles [°] of complex **32**

Ni(1)-C(2)	2.2109(12)	Ni(1)-C(3)	2.0720(12)
Ni(1)-C(4)	2.0099(12)	Ni(1)-C(5)	2.1442(12)
Ni(1)-C(8)	2.0803(12)	Ni(1)-C(9)	2.0800(12)
Ni(1)-C(12)	2.0901(12)	Ni(1)-C(13)	2.0652(12)
O(1)-C(6)	1.2162(16)	O(2)-C(6)	1.3590(15)
O(2)-C(7)	1.4407(17)	C(1)-C(2)	1.5007(19)
C(2)-C(3)	1.3853(18)	C(3)-C(4)	1.4319(18)
C(4)-C(5)	1.4072(17)	C(5)-C(6)	1.4548(18)
C(8)-C(9)	1.3827(18)	C(8)-C(15)	1.5193(19)
C(9)-C(10)	1.5128(18)	C(10)-C(11)	1.5370(19)
C(11)-C(12)	1.5168(18)	C(12)-C(13)	1.3868(19)
C(13)-C(14)	1.5145(19)	C(14)-C(15)	1.545(2)
Ni(2)-C(22)	2.1974(12)	Ni(2)-C(23)	2.0668(12)
Ni(2)-C(24)	1.9964(12)	Ni(2)-C(25)	2.1483(12)
Ni(2)-C(28)	2.0820(13)	Ni(2)-C(29)	2.0796(12)
Ni(2)-C(32)	2.0788(12)	Ni(2)-C(33)	2.0611(13)
O(3)-C(26)	1.2177(16)	O(4)-C(26)	1.3589(15)
O(4)-C(27)	1.4366(16)	C(21)-C(22)	1.5016(18)
C(22)-C(23)	1.3849(17)	C(23)-C(24)	1.4362(17)
C(24)-C(25)	1.4057(17)	C(25)-C(26)	1.4581(17)
C(28)-C(29)	1.3839(18)	C(28)-C(35B)	1.315(7)
C(28)-C(35A)	1.578(3)	C(29)-C(30)	1.5028(19)
C(30)-C(31)	1.509(2)	C(31)-C(32)	1.516(2)
C(32)-C(33)	1.3805(19)	C(33)-C(34B)	1.730(7)
C(33)-C(34A)	1.475(2)	C(34B)-C(35B)	1.498(10)
C(34A)-C(35A)	1.545(3)		
C(3)-Ni(1)-C(2)	37.57(5)	C(3)-Ni(1)-C(5)	72.00(5)
C(3)-Ni(1)-C(8)	153.09(5)	C(3)-Ni(1)-C(9)	126.56(5)
C(3)-Ni(1)-C(12)	107.50(5)	C(4)-Ni(1)-C(2)	71.20(5)
C(4)-Ni(1)-C(3)	41.04(5)	C(4)-Ni(1)-C(5)	39.43(5)
C(4)-Ni(1)-C(8)	137.91(5)	C(4)-Ni(1)-C(9)	155.28(5)
C(4)-Ni(1)-C(12)	116.70(5)	C(4)-Ni(1)-C(13)	100.55(5)
C(5)-Ni(1)-C(2)	83.56(5)	C(8)-Ni(1)-C(2)	117.90(5)
C(8)-Ni(1)-C(5)	98.71(5)	C(8)-Ni(1)-C(12)	95.07(5)
C(9)-Ni(1)-C(2)	90.22(5)	C(9)-Ni(1)-C(5)	124.67(5)
C(9)-Ni(1)-C(8)	38.83(5)	C(9)-Ni(1)-C(12)	86.35(5)
C(12)-Ni(1)-C(2)	119.35(5)	C(12)-Ni(1)-C(5)	143.01(5)
C(13)-Ni(1)-C(2)	151.54(5)	C(13)-Ni(1)-C(3)	119.91(5)
C(13)-Ni(1)-C(5)	107.68(5)	C(13)-Ni(1)-C(8)	86.87(5)
C(13)-Ni(1)-C(9)	103.35(5)	C(13)-Ni(1)-C(12)	38.99(5)
C(6)-O(2)-C(7)	115.05(11)	C(1)-C(2)-Ni(1)	126.20(9)
C(3)-C(2)-Ni(1)	65.77(7)	C(3)-C(2)-C(1)	120.95(12)
C(2)-C(3)-Ni(1)	76.66(7)	C(2)-C(3)-C(4)	121.86(11)
C(4)-C(3)-Ni(1)	67.15(7)	C(3)-C(4)-Ni(1)	71.81(7)

C(5)-C(4)-Ni(1)	75.44(7)	C(5)-C(4)-C(3)	121.64(11)
C(4)-C(5)-Ni(1)	65.13(7)	C(4)-C(5)-C(6)	117.50(11)
C(6)-C(5)-Ni(1)	116.36(8)	O(1)-C(6)-O(2)	121.61(13)
O(1)-C(6)-C(5)	126.73(12)	O(2)-C(6)-C(5)	111.65(11)
C(9)-C(8)-Ni(1)	70.58(7)	C(9)-C(8)-C(15)	123.81(12)
C(15)-C(8)-Ni(1)	109.54(9)	C(8)-C(9)-Ni(1)	70.60(7)
C(8)-C(9)-C(10)	125.83(12)	C(10)-C(9)-Ni(1)	107.03(8)
C(9)-C(10)-C(11)	113.87(11)	C(12)-C(11)-C(10)	113.01(10)
C(11)-C(12)-Ni(1)	109.76(8)	C(13)-C(12)-Ni(1)	69.54(7)
C(13)-C(12)-C(11)	123.84(12)	C(12)-C(13)-Ni(1)	71.48(7)
C(12)-C(13)-C(14)	126.11(12)	C(14)-C(13)-Ni(1)	106.83(9)
C(13)-C(14)-C(15)	113.41(11)	C(8)-C(15)-C(14)	112.77(11)
C(23)-Ni(2)-C(22)	37.75(5)	C(23)-Ni(2)-C(25)	71.91(5)
C(23)-Ni(2)-C(28)	121.75(6)	C(23)-Ni(2)-C(29)	102.73(5)
C(23)-Ni(2)-C(32)	129.09(5)	C(24)-Ni(2)-C(22)	71.71(5)
C(24)-Ni(2)-C(23)	41.35(5)	C(24)-Ni(2)-C(25)	39.44(5)
C(24)-Ni(2)-C(28)	103.21(5)	C(24)-Ni(2)-C(29)	112.51(5)
C(24)-Ni(2)-C(32)	159.30(5)	C(24)-Ni(2)-C(33)	136.03(6)
C(25)-Ni(2)-C(22)	83.38(5)	C(28)-Ni(2)-C(22)	151.86(5)
C(28)-Ni(2)-C(25)	110.70(5)	C(29)-Ni(2)-C(22)	116.16(5)
C(29)-Ni(2)-C(25)	141.66(5)	C(29)-Ni(2)-C(28)	38.85(5)
C(32)-Ni(2)-C(22)	93.08(5)	C(32)-Ni(2)-C(25)	127.07(5)
C(32)-Ni(2)-C(28)	96.76(5)	C(32)-Ni(2)-C(29)	86.55(5)
C(33)-Ni(2)-C(22)	116.29(5)	C(33)-Ni(2)-C(23)	151.11(6)
C(33)-Ni(2)-C(25)	96.79(5)	C(33)-Ni(2)-C(28)	87.01(6)
C(33)-Ni(2)-C(29)	102.18(6)	C(33)-Ni(2)-C(32)	38.96(5)
C(26)-O(4)-C(27)	115.07(10)	C(21)-C(22)-Ni(2)	124.96(9)
C(23)-C(22)-Ni(2)	66.01(7)	C(23)-C(22)-C(21)	120.94(12)
C(22)-C(23)-Ni(2)	76.24(7)	C(22)-C(23)-C(24)	121.53(11)
C(24)-C(23)-Ni(2)	66.70(7)	C(23)-C(24)-Ni(2)	71.95(7)
C(25)-C(24)-Ni(2)	76.12(7)	C(25)-C(24)-C(23)	121.20(11)
C(24)-C(25)-Ni(2)	64.44(7)	C(24)-C(25)-C(26)	118.58(11)
C(26)-C(25)-Ni(2)	117.87(8)	O(3)-C(26)-O(4)	121.83(12)
O(3)-C(26)-C(25)	127.11(12)	O(4)-C(26)-C(25)	111.07(11)
C(29)-C(28)-Ni(2)	70.49(7)	C(29)-C(28)-C(35A)	120.74(13)
C(35B)-C(28)-Ni(2)	114.6(3)	C(35B)-C(28)-C(29)	138.1(4)
C(35A)-C(28)-Ni(2)	107.33(10)	C(28)-C(29)-Ni(2)	70.67(7)
C(28)-C(29)-C(30)	125.73(13)	C(30)-C(29)-Ni(2)	107.69(9)
C(29)-C(30)-C(31)	115.43(12)	C(30)-C(31)-C(32)	114.85(12)
C(31)-C(32)-Ni(2)	109.44(9)	C(33)-C(32)-Ni(2)	69.83(7)
C(33)-C(32)-C(31)	123.31(13)	C(32)-C(33)-Ni(2)	71.21(7)
C(32)-C(33)-C(34B)	108.9(3)	C(32)-C(33)-C(34A)	130.51(15)
C(34B)-C(33)-Ni(2)	101.4(2)	C(34A)-C(33)-Ni(2)	108.18(10)
C(35B)-C(34B)-C(33)	115.2(5)	C(28)-C(35B)-C(34B)	111.6(6)
C(33)-C(34A)-C(35A)	112.30(14)	H(34C)-C(34A)-H(34D)	107.9
C(34A)-C(35A)-C(28)	112.53(13)		

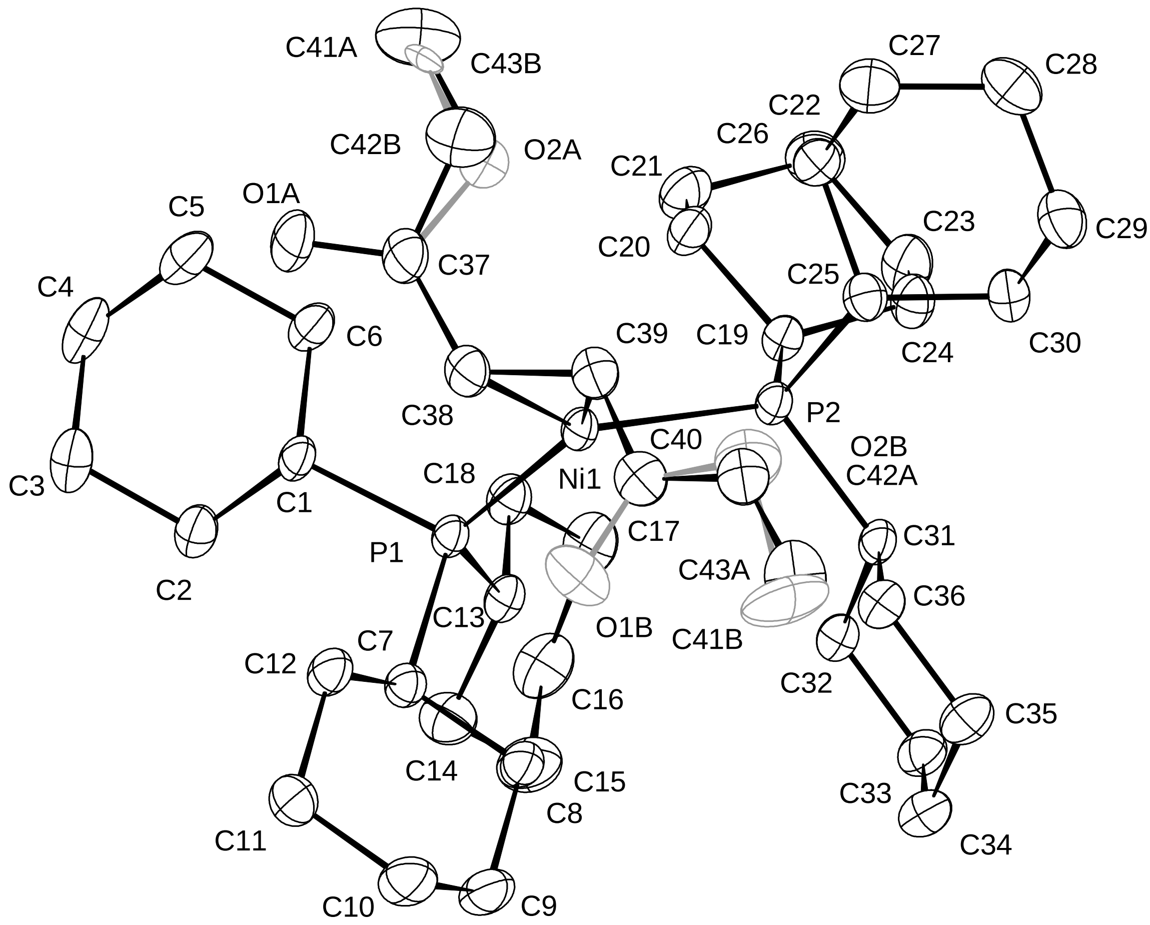

Figure S7. The asymmetric unit of complex **33**; H-atoms have been removed for clarity.

$C_{50.50}$ H_{94} Ni O_2 P_2, M_r = 853.90 g·mol^{-1}, orange prism, crystal size 0.24 x 0.178 x 0.134 mm^3, monoclinic, space group $P2_1/n$ [14], a = 10.3582(4) Å, b = 14.3833(6) Å, c = 33.9534(15) Å, β = 90.275(2)°, V = 5058.5(4) Å^3, T = 150(2) K, Z = 4, D_{calc} = 1.121 g·cm^3, λ = 0.71073 Å, $\mu(Mo\text{-}K_\alpha)$ = 0.482 mm^{-1}, Gaussian absorption correction ($T_{\min}$ = 0.93, $T_{\max}$ = 0.96), Bruker-AXS Kappa Mach3 with APEX-II detector and IµS microfocus source, 1.199 < θ < 29.129°, 125218 measured reflections, 13595 independent reflections, 9255 reflections with $I > 2\sigma(I)$, R_{int} = 0.0832. The structure was solved by *SHELXT* and refined by full-matrix least-squares (*SHELXL*) against F^2 to R_1 = 0.0466 [$I > 2\sigma(I)$], wR_2 = 0.1158, 483 parameters. **CCDC- 2522805**

The structure contains disordered pentane in two positions. Despite intensive efforts, this could not be adequately refined and a solvent mask was used.

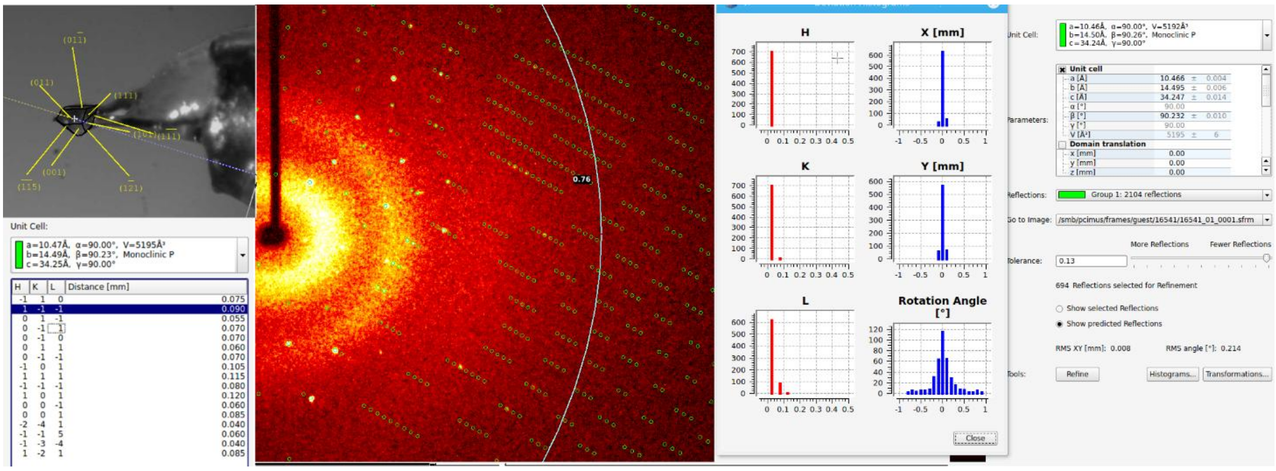

Figure S8. Crystal faces and unit cell determination/refinement of complex **33**

INTENSITY STATISTICS FOR DATASET

Resolution	#Data	#Theory	%Complete	Redundancy	Mean I	Mean I/s	Rmerge	Rsigma
Inf - 2.91	249	251	99.2	14.29	61.05	80.76	0.0217	0.0084
2.91 - 1.91	585	585	100.0	15.59	26.83	63.51	0.0317	0.0108
1.91 - 1.51	830	830	100.0	15.81	17.63	49.08	0.0430	0.0140
1.51 - 1.31	847	847	100.0	15.78	11.75	37.60	0.0611	0.0191
1.31 - 1.19	827	827	100.0	15.31	8.64	28.80	0.0817	0.0258
1.19 - 1.10	880	880	100.0	14.07	6.84	21.90	0.1046	0.0354
1.10 - 1.03	886	886	100.0	10.40	5.77	15.19	0.1282	0.0530
1.03 - 0.98	814	814	100.0	8.57	4.88	11.82	0.1552	0.0713
0.98 - 0.94	762	762	100.0	7.57	4.39	9.78	0.1711	0.0871
0.94 - 0.90	922	922	100.0	6.48	3.93	8.00	0.1898	0.1073
0.90 - 0.87	809	809	100.0	6.19	3.33	6.70	0.2198	0.1307
0.87 - 0.84	933	933	100.0	5.77	2.59	5.07	0.2735	0.1786
0.84 - 0.82	699	699	100.0	5.70	2.55	4.72	0.2921	0.1882
0.82 - 0.80	723	723	100.0	5.57	2.18	3.97	0.3344	0.2272
0.80 - 0.78	866	869	99.7	5.37	1.79	3.23	0.3917	0.2887
0.78 - 0.76	916	919	99.7	5.18	1.61	2.72	0.4399	0.3374
0.76 - 0.74	987	996	99.1	5.17	1.36	2.37	0.4881	0.4027
0.74 - 0.73	585	593	98.7	4.84	1.35	2.18	0.5152	0.4391
0.73 - 0.71	1190	1208	98.5	4.79	1.16	1.84	0.5907	0.5269
0.71 - 0.70	660	674	97.9	4.70	1.00	1.54	0.6576	0.6217
0.70 - 0.69	561	628	89.3	3.34	0.82	1.18	0.6651	1.0029
0.79 - 0.69	5336	5457	97.8	4.80	1.28	2.12	0.5228	0.4686
Inf - 0.69	16531	16655	99.3	8.35	6.10	14.52	0.0836	0.0717

Table S7. Crystal data and structure refinement of complex **33**

Empirical formula	$C_{50.50}$ H_{94} Ni O_2 P_2	
Color	orange	
Formula weight	853.90 g·mol^{-1}	
Temperature	150(2) K	
Wavelength	0.71073 Å	
Crystal system	Monoclinic	
Space group	$P2_1/n$, (no. 14)	
Unit cell dimensions	a = 10.3582(4) Å	α= 90°.
	b = 14.3833(6) Å	β= 90.275(2)°.
	c = 33.9534(15) Å	γ = 90°.
Volume	5058.5(4) Å^3	
Z	4	
Density (calculated)	1.121 Mg·m^{-3}	
Absorption coefficient	0.482 mm^{-1}	
F(000)	1884 e	
Crystal size	0.24 x 0.178 x 0.134 mm^3	
θ range for data collection	1.199 to 29.129°.	
Index ranges	-14 ≤ h ≤ 14, -19≤ k ≤ 19, -46 ≤ l ≤ 46	
Reflections collected	125218	
Independent reflections	13595 [R_{int} = 0.0832]	
Reflections with I>2σ(I)	9255	
Completeness to θ = 25.242°	100.0 %	
Absorption correction	Gaussian	
Max. and min. transmission	0.95956 and 0.93029	
Refinement method	Full-matrix least-squares on F^2	
Data / restraints / parameters	13595 / 25 / 483	
Goodness-of-fit on F^2	1.050	
Final R indices [I>2σ(I)]	R_1 = 0.0466	wR^2 = 0.1057
R indices (all data)	R_1 = 0.0796	wR^2 = 0.1158
Largest diff. peak and hole	0.372 and -0.443 e·Å^{-3}	

Table S8. Bond lengths [Å] and angles [°] of complex **33**

Ni(1)-P(1)	2.2208(5)	Ni(1)-P(2)	2.2103(5)
Ni(1)-C(38)	2.003(2)	Ni(1)-C(39)	1.987(2)
P(1)-C(1)	1.8567(18)	P(1)-C(7)	1.8607(19)
P(1)-C(13)	1.875(2)	P(2)-C(19)	1.857(2)
P(2)-C(25)	1.870(2)	P(2)-C(31)	1.8571(18)
C(1)-H(1)	1.0000	C(1)-C(2)	1.528(3)
C(1)-C(6)	1.529(3)	C(2)-H(2A)	0.9900
C(2)-H(2B)	0.9900	C(2)-C(3)	1.534(3)
C(3)-H(3A)	0.9900	C(3)-H(3B)	0.9900
C(3)-C(4)	1.508(3)	C(4)-H(4A)	0.9900
C(4)-H(4B)	0.9900	C(4)-C(5)	1.514(3)
C(5)-H(5A)	0.9900	C(5)-H(5B)	0.9900
C(5)-C(6)	1.528(3)	C(6)-H(6A)	0.9900
C(6)-H(6B)	0.9900	C(7)-H(7)	1.0000
C(7)-C(8)	1.531(2)	C(7)-C(12)	1.525(3)
C(8)-H(8A)	0.9900	C(8)-H(8B)	0.9900
C(8)-C(9)	1.517(3)	C(9)-H(9A)	0.9900
C(9)-H(9B)	0.9900	C(9)-C(10)	1.519(3)
C(10)-H(10A)	0.9900	C(10)-H(10B)	0.9900
C(10)-C(11)	1.517(3)	C(11)-H(11A)	0.9900
C(11)-H(11B)	0.9900	C(11)-C(12)	1.528(3)
C(12)-H(12A)	0.9900	C(12)-H(12B)	0.9900
C(13)-H(13)	1.0000	C(13)-C(14)	1.536(3)
C(13)-C(18)	1.526(3)	C(14)-H(14A)	0.9900
C(14)-H(14B)	0.9900	C(14)-C(15)	1.534(3)
C(15)-H(15A)	0.9900	C(15)-H(15B)	0.9900
C(15)-C(16)	1.520(3)	C(16)-H(16A)	0.9900
C(16)-H(16B)	0.9900	C(16)-C(17)	1.517(3)
C(17)-H(17A)	0.9900	C(17)-H(17B)	0.9900
C(17)-C(18)	1.526(3)	C(18)-H(18A)	0.9900
C(18)-H(18B)	0.9900	C(19)-H(19)	1.0000
C(19)-C(20)	1.539(2)	C(19)-C(24)	1.533(3)
C(20)-H(20A)	0.9900	C(20)-H(20B)	0.9900
C(20)-C(21)	1.522(3)	C(21)-H(21A)	0.9900
C(21)-H(21B)	0.9900	C(21)-C(22)	1.524(3)
C(22)-H(22A)	0.9900	C(22)-H(22B)	0.9900
C(22)-C(23)	1.520(3)	C(23)-H(23A)	0.9900
C(23)-H(23B)	0.9900	C(23)-C(24)	1.521(3)
C(24)-H(24A)	0.9900	C(24)-H(24B)	0.9900
C(25)-H(25)	1.0000	C(25)-C(26)	1.534(3)
C(25)-C(30)	1.538(3)	C(26)-H(26A)	0.9900
C(26)-H(26B)	0.9900	C(26)-C(27)	1.522(3)
C(27)-H(27A)	0.9900	C(27)-H(27B)	0.9900
C(27)-C(28)	1.519(3)	C(28)-H(28A)	0.9900
C(28)-H(28B)	0.9900	C(28)-C(29)	1.520(3)

C(29)-H(29A)	0.9900	C(29)-H(29B)	0.9900
C(29)-C(30)	1.522(3)	C(30)-H(30A)	0.9900
C(30)-H(30B)	0.9900	C(31)-H(31)	1.0000
C(31)-C(32)	1.527(3)	C(31)-C(36)	1.526(3)
C(32)-H(32A)	0.9900	C(32)-H(32B)	0.9900
C(32)-C(33)	1.530(3)	C(33)-H(33A)	0.9900
C(33)-H(33B)	0.9900	C(33)-C(34)	1.522(3)
C(34)-H(34A)	0.9900	C(34)-H(34B)	0.9900
C(34)-C(35)	1.513(3)	C(35)-H(35A)	0.9900
C(35)-H(35B)	0.9900	C(35)-C(36)	1.529(3)
C(36)-H(36A)	0.9900	C(36)-H(36B)	0.9900
C(37)-H(37)	0.9500	C(37)-C(38)	1.450(3)
C(37)-O(1A)	1.216(3)	C(37)-O(2A)	1.340(6)
C(37)-C(42B)	1.41(3)	C(38)-H(38)	1.0000
C(38)-C(39)	1.428(3)	C(39)-H(39)	1.0000
C(39)-C(40)	1.462(3)	C(40)-H(40)	0.9500
C(40)-C(42A)	1.332(10)	C(40)-O(1B)	1.250(7)
C(40)-O(2B)	1.31(3)	O(2A)-C(41A)	1.577(12)
C(41A)-H(41A)	0.9800	C(41A)-H(41B)	0.9800
C(41A)-H(41C)	0.9800	C(42A)-H(42A)	0.9500
C(42A)-C(43A)	1.500(8)	C(43A)-H(43A)	0.9800
C(43A)-H(43B)	0.9800	C(43A)-H(43C)	0.9800
O(2B)-C(41B)	1.429(10)	C(41B)-H(41D)	0.9800
C(41B)-H(41E)	0.9800	C(41B)-H(41F)	0.9800
C(42B)-H(42B)	0.9500	C(42B)-C(43B)	1.11(4)
C(43B)-H(43D)	0.9800	C(43B)-H(43E)	0.9800
C(43B)-H(43F)	0.9800		

P(2)-Ni(1)-P(1)	117.61(2)	C(38)-Ni(1)-P(1)	102.68(6)
C(38)-Ni(1)-P(2)	139.43(6)	C(39)-Ni(1)-P(1)	139.90(6)
C(39)-Ni(1)-P(2)	100.75(6)	C(39)-Ni(1)-C(38)	41.93(8)
C(1)-P(1)-Ni(1)	113.50(6)	C(1)-P(1)-C(7)	102.80(8)
C(1)-P(1)-C(13)	107.91(9)	C(7)-P(1)-Ni(1)	109.91(6)
C(7)-P(1)-C(13)	99.89(9)	C(13)-P(1)-Ni(1)	120.59(6)
C(19)-P(2)-Ni(1)	113.01(6)	C(19)-P(2)-C(25)	107.45(9)
C(25)-P(2)-Ni(1)	112.08(7)	C(31)-P(2)-Ni(1)	118.17(6)
C(31)-P(2)-C(19)	103.68(9)	C(31)-P(2)-C(25)	101.26(8)
P(1)-C(1)-H(1)	105.1	C(2)-C(1)-P(1)	117.44(13)
C(2)-C(1)-H(1)	105.1	C(2)-C(1)-C(6)	110.78(15)
C(6)-C(1)-P(1)	112.25(13)	C(6)-C(1)-H(1)	105.1
C(1)-C(2)-H(2A)	109.5	C(1)-C(2)-H(2B)	109.5
C(1)-C(2)-C(3)	110.76(17)	H(2A)-C(2)-H(2B)	108.1
C(3)-C(2)-H(2A)	109.5	C(3)-C(2)-H(2B)	109.5
C(2)-C(3)-H(3A)	109.3	C(2)-C(3)-H(3B)	109.3
H(3A)-C(3)-H(3B)	108.0	C(4)-C(3)-C(2)	111.44(18)
C(4)-C(3)-H(3A)	109.3	C(4)-C(3)-H(3B)	109.3
C(3)-C(4)-H(4A)	109.3	C(3)-C(4)-H(4B)	109.3

C(3)-C(4)-C(5)	111.57(17)	H(4A)-C(4)-H(4B)	108.0
C(5)-C(4)-H(4A)	109.3	C(5)-C(4)-H(4B)	109.3
C(4)-C(5)-H(5A)	109.3	C(4)-C(5)-H(5B)	109.3
C(4)-C(5)-C(6)	111.54(17)	H(5A)-C(5)-H(5B)	108.0
C(6)-C(5)-H(5A)	109.3	C(6)-C(5)-H(5B)	109.3
C(1)-C(6)-H(6A)	109.5	C(1)-C(6)-H(6B)	109.5
C(5)-C(6)-C(1)	110.76(17)	C(5)-C(6)-H(6A)	109.5
C(5)-C(6)-H(6B)	109.5	H(6A)-C(6)-H(6B)	108.1
P(1)-C(7)-H(7)	107.1	C(8)-C(7)-P(1)	110.10(13)
C(8)-C(7)-H(7)	107.1	C(12)-C(7)-P(1)	115.80(13)
C(12)-C(7)-H(7)	107.1	C(12)-C(7)-C(8)	109.28(16)
C(7)-C(8)-H(8A)	109.4	C(7)-C(8)-H(8B)	109.4
H(8A)-C(8)-H(8B)	108.0	C(9)-C(8)-C(7)	110.95(16)
C(9)-C(8)-H(8A)	109.4	C(9)-C(8)-H(8B)	109.4
C(8)-C(9)-H(9A)	109.3	C(8)-C(9)-H(9B)	109.3
C(8)-C(9)-C(10)	111.55(18)	H(9A)-C(9)-H(9B)	108.0
C(10)-C(9)-H(9A)	109.3	C(10)-C(9)-H(9B)	109.3
C(9)-C(10)-H(10A)	109.4	C(9)-C(10)-H(10B)	109.4
H(10A)-C(10)-H(10B)	108.0	C(11)-C(10)-C(9)	111.14(18)
C(11)-C(10)-H(10A)	109.4	C(11)-C(10)-H(10B)	109.4
C(10)-C(11)-H(11A)	109.4	C(10)-C(11)-H(11B)	109.4
C(10)-C(11)-C(12)	111.24(17)	H(11A)-C(11)-H(11B)	108.0
C(12)-C(11)-H(11A)	109.4	C(12)-C(11)-H(11B)	109.4
C(7)-C(12)-C(11)	110.87(17)	C(7)-C(12)-H(12A)	109.5
C(7)-C(12)-H(12B)	109.5	C(11)-C(12)-H(12A)	109.5
C(11)-C(12)-H(12B)	109.5	H(12A)-C(12)-H(12B)	108.1
P(1)-C(13)-H(13)	104.0	C(14)-C(13)-P(1)	120.77(14)
C(14)-C(13)-H(13)	104.0	C(18)-C(13)-P(1)	113.50(13)
C(18)-C(13)-H(13)	104.0	C(18)-C(13)-C(14)	108.70(16)
C(13)-C(14)-H(14A)	109.7	C(13)-C(14)-H(14B)	109.7
H(14A)-C(14)-H(14B)	108.2	C(15)-C(14)-C(13)	109.62(18)
C(15)-C(14)-H(14A)	109.7	C(15)-C(14)-H(14B)	109.7
C(14)-C(15)-H(15A)	109.2	C(14)-C(15)-H(15B)	109.2
H(15A)-C(15)-H(15B)	107.9	C(16)-C(15)-C(14)	112.04(19)
C(16)-C(15)-H(15A)	109.2	C(16)-C(15)-H(15B)	109.2
C(15)-C(16)-H(16A)	109.4	C(15)-C(16)-H(16B)	109.4
H(16A)-C(16)-H(16B)	108.0	C(17)-C(16)-C(15)	111.27(19)
C(17)-C(16)-H(16A)	109.4	C(17)-C(16)-H(16B)	109.4
C(16)-C(17)-H(17A)	109.3	C(16)-C(17)-H(17B)	109.3
C(16)-C(17)-C(18)	111.48(19)	H(17A)-C(17)-H(17B)	108.0
C(18)-C(17)-H(17A)	109.3	C(18)-C(17)-H(17B)	109.3
C(13)-C(18)-C(17)	110.45(16)	C(13)-C(18)-H(18A)	109.6
C(13)-C(18)-H(18B)	109.6	C(17)-C(18)-H(18A)	109.6
C(17)-C(18)-H(18B)	109.6	H(18A)-C(18)-H(18B)	108.1
P(2)-C(19)-H(19)	105.2	C(20)-C(19)-P(2)	112.75(13)
C(20)-C(19)-H(19)	105.2	C(24)-C(19)-P(2)	117.30(13)
C(24)-C(19)-H(19)	105.2	C(24)-C(19)-C(20)	110.11(16)

C(19)-C(20)-H(20A)	109.4	C(19)-C(20)-H(20B)	109.4
H(20A)-C(20)-H(20B)	108.0	C(21)-C(20)-C(19)	111.38(17)
C(21)-C(20)-H(20A)	109.4	C(21)-C(20)-H(20B)	109.4
C(20)-C(21)-H(21A)	109.4	C(20)-C(21)-H(21B)	109.4
C(20)-C(21)-C(22)	111.35(17)	H(21A)-C(21)-H(21B)	108.0
C(22)-C(21)-H(21A)	109.4	C(22)-C(21)-H(21B)	109.4
C(21)-C(22)-H(22A)	109.4	C(21)-C(22)-H(22B)	109.4
H(22A)-C(22)-H(22B)	108.0	C(23)-C(22)-C(21)	110.97(18)
C(23)-C(22)-H(22A)	109.4	C(23)-C(22)-H(22B)	109.4
C(22)-C(23)-H(23A)	109.4	C(22)-C(23)-H(23B)	109.4
C(22)-C(23)-C(24)	111.10(18)	H(23A)-C(23)-H(23B)	108.0
C(24)-C(23)-H(23A)	109.4	C(24)-C(23)-H(23B)	109.4
C(19)-C(24)-H(24A)	109.4	C(19)-C(24)-H(24B)	109.4
C(23)-C(24)-C(19)	111.07(16)	C(23)-C(24)-H(24A)	109.4
C(23)-C(24)-H(24B)	109.4	H(24A)-C(24)-H(24B)	108.0
P(2)-C(25)-H(25)	104.5	C(26)-C(25)-P(2)	112.47(13)
C(26)-C(25)-H(25)	104.5	C(26)-C(25)-C(30)	109.00(16)
C(30)-C(25)-P(2)	120.34(14)	C(30)-C(25)-H(25)	104.5
C(25)-C(26)-H(26A)	109.3	C(25)-C(26)-H(26B)	109.3
H(26A)-C(26)-H(26B)	108.0	C(27)-C(26)-C(25)	111.40(17)
C(27)-C(26)-H(26A)	109.3	C(27)-C(26)-H(26B)	109.3
C(26)-C(27)-H(27A)	109.5	C(26)-C(27)-H(27B)	109.5
H(27A)-C(27)-H(27B)	108.1	C(28)-C(27)-C(26)	110.78(19)
C(28)-C(27)-H(27A)	109.5	C(28)-C(27)-H(27B)	109.5
C(27)-C(28)-H(28A)	109.5	C(27)-C(28)-H(28B)	109.5
C(27)-C(28)-C(29)	110.92(19)	H(28A)-C(28)-H(28B)	108.0
C(29)-C(28)-H(28A)	109.5	C(29)-C(28)-H(28B)	109.5
C(28)-C(29)-H(29A)	109.3	C(28)-C(29)-H(29B)	109.3
C(28)-C(29)-C(30)	111.78(18)	H(29A)-C(29)-H(29B)	107.9
C(30)-C(29)-H(29A)	109.3	C(30)-C(29)-H(29B)	109.3
C(25)-C(30)-H(30A)	109.6	C(25)-C(30)-H(30B)	109.6
C(29)-C(30)-C(25)	110.41(17)	C(29)-C(30)-H(30A)	109.6
C(29)-C(30)-H(30B)	109.6	H(30A)-C(30)-H(30B)	108.1
P(2)-C(31)-H(31)	108.1	C(32)-C(31)-P(2)	113.22(12)
C(32)-C(31)-H(31)	108.1	C(36)-C(31)-P(2)	109.54(13)
C(36)-C(31)-H(31)	108.1	C(36)-C(31)-C(32)	109.59(16)
C(31)-C(32)-H(32A)	109.3	C(31)-C(32)-H(32B)	109.3
C(31)-C(32)-C(33)	111.58(15)	H(32A)-C(32)-H(32B)	108.0
C(33)-C(32)-H(32A)	109.3	C(33)-C(32)-H(32B)	109.3
C(32)-C(33)-H(33A)	109.4	C(32)-C(33)-H(33B)	109.4
H(33A)-C(33)-H(33B)	108.0	C(34)-C(33)-C(32)	111.28(16)
C(34)-C(33)-H(33A)	109.4	C(34)-C(33)-H(33B)	109.4
C(33)-C(34)-H(34A)	109.7	C(33)-C(34)-H(34B)	109.7
H(34A)-C(34)-H(34B)	108.2	C(35)-C(34)-C(33)	110.05(17)
C(35)-C(34)-H(34A)	109.7	C(35)-C(34)-H(34B)	109.7
C(34)-C(35)-H(35A)	109.4	C(34)-C(35)-H(35B)	109.4
C(34)-C(35)-C(36)	110.95(17)	H(35A)-C(35)-H(35B)	108.0

C(36)-C(35)-H(35A)	109.4	C(36)-C(35)-H(35B)	109.4
C(31)-C(36)-C(35)	111.80(16)	C(31)-C(36)-H(36A)	109.3
C(31)-C(36)-H(36B)	109.3	C(35)-C(36)-H(36A)	109.3
C(35)-C(36)-H(36B)	109.3	H(36A)-C(36)-H(36B)	107.9
C(38)-C(37)-H(37)	117.0	O(1A)-C(37)-C(38)	125.0(2)
O(1A)-C(37)-O(2A)	121.1(3)	O(2A)-C(37)-C(38)	113.9(3)
C(42B)-C(37)-H(37)	117.0	C(42B)-C(37)-C(38)	125.9(12)
Ni(1)-C(38)-H(38)	115.7	C(37)-C(38)-Ni(1)	109.96(15)
C(37)-C(38)-H(38)	115.7	C(39)-C(38)-Ni(1)	68.43(11)
C(39)-C(38)-C(37)	121.91(19)	C(39)-C(38)-H(38)	115.7
Ni(1)-C(39)-H(39)	116.9	C(38)-C(39)-Ni(1)	69.65(11)
C(38)-C(39)-H(39)	116.9	C(38)-C(39)-C(40)	119.23(19)
C(40)-C(39)-Ni(1)	107.83(13)	C(40)-C(39)-H(39)	116.9
C(39)-C(40)-H(40)	117.6	C(42A)-C(40)-C(39)	124.8(5)
C(42A)-C(40)-H(40)	117.6	O(1B)-C(40)-C(39)	122.5(4)
O(1B)-C(40)-O(2B)	122.4(12)	O(2B)-C(40)-C(39)	114.7(11)
C(37)-O(2A)-C(41A)	114.5(5)	O(2A)-C(41A)-H(41A)	109.5
O(2A)-C(41A)-H(41B)	109.5	O(2A)-C(41A)-H(41C)	109.5
H(41A)-C(41A)-H(41B)	109.5	H(41A)-C(41A)-H(41C)	109.5
H(41B)-C(41A)-H(41C)	109.5	C(40)-C(42A)-H(42A)	118.0
C(40)-C(42A)-C(43A)	123.9(9)	C(43A)-C(42A)-H(42A)	118.0
C(42A)-C(43A)-H(43A)	109.5	C(42A)-C(43A)-H(43B)	109.5
C(42A)-C(43A)-H(43C)	109.5	H(43A)-C(43A)-H(43B)	109.5
H(43A)-C(43A)-H(43C)	109.5	H(43B)-C(43A)-H(43C)	109.5
C(40)-O(2B)-C(41B)	116(2)	O(2B)-C(41B)-H(41D)	109.5
O(2B)-C(41B)-H(41E)	109.5	O(2B)-C(41B)-H(41F)	109.5
H(41D)-C(41B)-H(41E)	109.5	H(41D)-C(41B)-H(41F)	109.5
H(41E)-C(41B)-H(41F)	109.5	C(37)-C(42B)-H(42B)	118.3
C(43B)-C(42B)-C(37)	123(3)	C(43B)-C(42B)-H(42B)	118.3
C(42B)-C(43B)-H(43D)	109.5	C(42B)-C(43B)-H(43E)	109.5
C(42B)-C(43B)-H(43F)	109.5	H(43D)-C(43B)-H(43E)	109.5
H(43D)-C(43B)-H(43F)	109.5	H(43E)-C(43B)-H(43F)	109.5

Experimental

General Information. Unless stated otherwise, all reactions were carried out under argon atmosphere in flame-dried Schlenk glassware. Solvents were purified by distillation over the indicated drying agents under argon: toluene ($NaAlEt_4$), THF (Mg/anthracene), Et_2O (Mg/anthracene), *tert*-butyl methyl ether (CaH_2), pentane (Na/K), CH_2Cl_2 (CaH_2), $CHCl_3$ ($CaCl_2$), methanol (Mg). DMF and NEt_3 were dried by an absorption solvent purification system based on molecular sieves. *tert*-Butanol was dried over 3 Å molecular sieves.

Flash chromatography: Merck silica gel 60 (40-63 µm or 15-40 µm) with the indicated eluent mixture. Automated flash chromatography was performed on a Biotage® Isolera™ One flash chromatography instrument. In all cases, Biotage® pre-packed silica cartridges (Biotage® Sfär Silica HC D Duo 20 µm manufacturer numbers FSUD-0443-0XXX; [the last three digits describe the size of column e.g. FSRD-0445-0010 for a 10 g column]) were used with the eluent gradient given in the procedure.

Thin layer chromatography (TLC): Macherey-Nagel precoated plates (POLYGRAM®SIL/UV254); visualization by UV light (λ = 254 nm) or by staining with solutions of *p*-anisaldeyhde, ninhydrin, ceric ammonium molybdate, or $KMnO_4$.

High performance liquid chromatography (HPLC): Analyses for the determination of the enantiomeric excess (ee) of the individual compounds were performed on a Shimadzu LC 2020 instrument equipped with a Shimadzu SPD-M20A UV/VIS detector. Solvents (HPLC grade) were purchased and used as received. The exact conditions are stated separately for each compound.

Nuclear magnetic resonance (NMR): Spectra were recorded on *Bruker* Avance III 300, 400 MHz, 500 MHz (equipped with a Bruker TBO (H/F/X) probe, or an Avance Neo 600 MHz (equipped with a *Bruker* BBO CryoProbe) NMR spectrometers in the solvents indicated; chemical shifts are given in ppm relative to TMS, coupling constants (*J*) in Hz. The solvent signals were used as references and the chemical shifts converted to the TMS scale ($CDCl_3$: δ_C = 77.16 ppm; residual $CHCl_3$ in $CDCl_3$: δ_H = 7.26 ppm; [D_6]-DMSO: δ_C = 39.52 ppm; residual D_2HSOCD_3 in [D_6]-DMSO: δ_H = 2.50 ppm; CD_2Cl_2: δ_C = 53.84 ppm; residual $CDHCl_2$ in CD_2Cl_2: δ_H = 5.32 ppm). All spectra were recorded at 298 K unless stated otherwise. Peak multiplicities are indicated by the following abbreviations: s = singlet, d = doublet, t = triplet, q = quartet, p = pentet, h = hextet, hept = heptet, m = multiplet, dd = double doublet, dt = double triplet; “br” indicates a broad peak, where the width is large enough to eclipse small coupling constants, which might otherwise be expected. ^{19}F, ^{31}P and ^{13}C NMR spectra were recorded with broadband ^{1}H decoupling, unless stated otherwise.

Infrared spectra (IR): Spectra were recorded on an Alpha Platinum ATR instrument (Bruker); Absorption bands are reported in wavenumbers (cm^{-1}).

High-resolution mass spectrometry (HRMS): MS (EI): Finnigan MAT 8200 (70 eV), ESI-MS: ESQ3000 (Bruker), Thermo Scientific LTQ-FT, or Thermo Scientific Exactive. HRMS: Bruker APEX III FT-MS (7T magnet), MAT 95 (Finnigan), Thermo Scientific LTQ-FT, or Thermo Scientific Exactive. GC-MS: Shimadzu GCMS-QP2010 Ultra instrument, GC-MS (CI): Q Exactive GC Orbitrap.

Optical rotations were measured with an A-Krüss Otronic Model P8000-t polarimeter at a wavelength of 589 nm. The values are given as specific optical rotation with exact temperature, concentration (c (10 mg/mL)) and solvent.

Melting points (m.p.) were determined using a BÜCHI B-540 melting point apparatus.

Substrates

Aldehydes were purchased from commercial suppliers with the exception of geranial, 5-*tert*-butyldimethylsilyloxypentanal and (*S*)-glyceraldehyde acetonide, which were prepared by following literature procedures.[1,2,3] Liquid aldehydes were distilled and solid aldehydes were recrystallized and their purity confirmed by ^{1}H NMR.

(Ni(*t*Bu-stb)$_3$) (**16**) and Ni(cod)$_2$ were purchased from STREM chemicals.

The ligand precursor VAPOL[4] and the phosphoramidite ligand **L1**[5] were prepared following previously established synthetic routes.

Triethylborane solutions were prepared from neat triethylborane and the respective solvent.

Unless stated otherwise, all other commercially available compounds (TCI Chemicals, Fisher Scientific, Sigma Aldrich, abcr, BLDpharm) were used as received.

(2*E*,4*E*)-6,6,6-Trifluoro-*N*-methoxy-*N*-methylhexa-2,4-dienamide (12). Dibal-H (1 M in hexane, 13.1 mL, 13.09 mmol) was added dropwise at –78 °C (the temperature was constantly monitored with internal thermometer) to a solution of ethyl (*E*)-4,4,4-trifluorobut-2-enoate (**9**) (2.0 g, 11.90 mmol) in CH_2Cl_2 (22.8 mL). The resulting yellow solution was stirred for 1h.

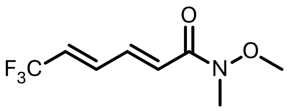

Diethyl (2-(methoxy(methyl)amino)-2-oxoethyl)phosphonate (**11**) (4.2 mL, 20.2 mmol) was carefully added to a suspension of NaH (485.3 mg, 20.2 mmol) in Et_2O (22.8 mL) at 0 °C to give a colorless solution after stirring for 30 min. This solution was then added dropwise to the solution containing the reduced ester at –78 °C. Stirring was continued for 15 min at –78 °C and then for 3 h at room temperature. *tert*-Butyl methyl ether (500 mL) and a saturated solution of Rochelle's salt (300 mL) was carefully added and the mixture was vigorously stirred until clear phase separation was reached. The organic phase was washed with HCl (2 M, 2 x 100 mL) and brine (100 mL) before it was dried over $MgSO_4$. After concentration *in vacuo*, the crude product was purified by flash chromatography (fine silica; hexane/ethyl acetate, 9:1 then 8:2) to obtain the title compound as a white crystalline solid (1.98 g, 80 %). m.p. = 41-42 °C. ^{1}H NMR (400 MHz, CD_2Cl_2, 298 K): δ [ppm] = 7.26 (ddq, *J* = 15.4, 11.2, 1.1 Hz, 1H), 6.98 – 6.88 (m, 1H), 6.78 (d, *J* = 15.2 Hz, 1H), 6.12 – 6.01 (m, 1H), 3.71 (s, 3H), 3.23 (s, 3H). ^{13}C NMR (101 MHz, CD_2Cl_2, 298 K): δ [ppm] = 165.7, 138.2, 136.0 (q, $^3J_{CF}$ = 6.9 Hz), 126.9, 124.9 (q, $^2J_{CF}$ = 34.0 Hz), 123.4 (d, $^1J_{CF}$ = 269.06 Hz), 62.4, 32.6. ^{19}F NMR (282 MHz, CD_2Cl_2, 298 K): δ [ppm] = –64.6 (s). IR (ATR): ṽ = 1666, 1643, 1614, 1422, 1386, 1321, 1268, 1218, 1102, 994, 863, 682, 569, 417 cm^{-1}. HRMS (GC-EI): *m/z*: calcd. for [$C_8H_{10}F_3NO_2$][M^+]: 209.0658, found: 209.0659.

Ethyl (*E*)-4,4-difluorobut-2-enoate (14). $NaBH_4$ (4.85 g, 128.1 mmol) was added in portions to a solution of ethyl 4,4-difluoro-3-oxobutanoate (16.2 mL, 122 mmol) in toluene (813 mL) at 0 °C. The resulting slurry was allowed to warm to room temperature. After stirring for 6 h, the reaction was carefully quenched with aq. HCl (10 % *w/w*) at 0 °C and the aqueous layer was extracted with *tert*-butyl methyl ether. The phases were separated and the aqueous layer was extracted with *tert*-butyl methyl ether (3 x 200 mL). The combined organic phases were dried over $MgSO_4$ and concentrated *in vacuo*.

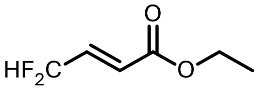

The crude alcohol product was dissolved in CH_2Cl_2 (206 mL) and the solution was cooled to –78 °C. Methanesulfonyl chloride (8.8 mL, 113.8 mmol) and triethylamine (43.3 mL, 310.5 mmol) were added, the cooling bath was removed and the mixture stirred at room temperature for 1 h. After cooling to 0 °C, water was added and the solution was again warmed to room temperature and stirred for another 1 h. The organic layer was separated and the aqueous phase extracted with

dichloromethane (2 x 200 mL). The combined organic layers were washed with conc. HCl (15 mL) and then evaporated in *vacuo*. Kugelrohr distillation (80 °C, 14 mbar) of the residue afforded the title compound as a colorless oil (36.8 g, 38 %). ^{1}H NMR (400 MHz, $CDCl_3$, 298 K): δ [ppm] = 6.87 – 6.72 (m, 1H), 6.41 – 6.00 (m, 1H), 6.31 – 6.22 (m, 1H), 4.23 (q, *J* = 7.1 Hz, 2H), 1.30 (t, *J* = 7.1 Hz, 3H). ^{19}F NMR (282 MHz, $CDCl_3$, 298 K): δ [ppm] = –115.9 (s).

(2*E*,4*E*)-6,6-Difluoro-*N*-methoxy-*N*-methylhexa-2,4-dienamide (15). Dibal-H (1 M in hexane, 50.0 mL, 50.0 mmol) was added dropwise at –78 °C (monitored with an internal thermometer) to a solution of ethyl (*E*)-4,4-difluorobut-2-enoate (**14**) (6.83 g, 45.49 mmol) in CH_2Cl_2 (91 mL). The resulting yellow solution was stirred for 1h.

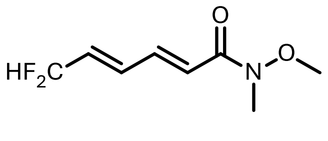

In parallel, diethyl (2-(methoxy(methyl)amino)-2-oxoethyl)phosphonate (**11**) (15.9 mL, 77.3 mmol) was carefully added to a suspension of NaH (1.75 g, 72.8 mmol) in Et_2O (91 mL) at 0 °C to give a colorless solution after stirring for 30 min. This solution was added dropwise to the solution of the reduced ester at –78 °C. The mixture was rapidly stirred for 15 min at –78 °C and for another 3 h at room temperature. *tert*-Butyl methyl ether (900 mL) and a saturated solution of Rochelle's salt (500 mL) were carefully added and the mixture was stirred until a clear phase separation was reached. The organic phase was washed with HCl (2 M, 2 x 180 mL) and brine (150 mL) before it was dried over $MgSO_4$. After concentration *in vacuo*, the crude product was purified by flash chromatography (fine silica; hexane/ethyl acetate, 9:1 then 7:3) to obtain the title product as a white crystalline solid (4.64 g, 53 %). m.p. = 51-53 °C. ^{1}H NMR (400 MHz, CD_2Cl_2, 298 K): δ [ppm] = 7.35 – 7.26 (m, 1H), 6.72 – 6.62 (m, 2H), 6.33 – 6.02 (m, 2H), 3.72 (s, 3H), 3.27 (s, 3H). ^{13}C NMR (101 MHz, CD_2Cl_2, 298 K): δ [ppm] = 166.1, 139.8 (t, $^4J_{CF}$ = 2.1 Hz), 134.3 (t, $^3J_{CF}$ = 11.7 Hz), 130.4 (t, $^2J_{CF}$ = 23.3 Hz), 124.2 (t, $^5J_{CF}$ = 2.4 Hz), 113.9 (t, $^1J_{CF}$ = 325.0 Hz), 62.1, 32.6. ^{19}F NMR (282 MHz, CD_2Cl_2, 298 K): δ [ppm] = –112.7 (s). IR (ATR): ṽ = 1637, 1612, 1422, 1375, 1210, 1179, 1130, 1092, 991, 877, 821, 599, 566, 426 cm^{-1}. HRMS (ESI$^+$): *m/z*: calcd. for $[C_8H_{11}F_2NO_2Na]^+$ $[M+Na]^+$: 214.0650, found: 214.0650.

Ethyl 4,4-difluoro-3-hydroxypentanoate (S1). Ethyl 2,2-difluoropropionate (10.0 g, 72.4 mmol) and ethyl acetate (9.9 mL, 101.4 mmol) were added to a suspension of NaH (2.26 g, 94.1 mmol) in THF (144.8 mL) at room temperature. The resulting slurry was stirred at 40 °C (bath temperature) overnight. The reaction was carefully quenched with aq. sat. NH_4Cl solution (50 mL), followed by addition of HCl (2 M) to adjust pH to 3-4. The aqueous phase was extracted with ethyl acetate (2 x 200 mL) before the combined organic fractions were dried over $MgSO_4$. After concentration in *vacuo*, the residue was purified by flash chromatography (hexane/*tert*-butyl methyl ether, 7:3) to obtain the title compound as a clear oil (10.8 g, 73 %, 1.2:1 mixture of keto and enol form).^{1}H NMR (400 MHz, $CDCl_3$, 298 K): δ [ppm] = 12.00 (s, 1H, enol), 5.52 (s, 1H, enol), 4.26 (dq, *J* = 12.3, 7.1 Hz, 4H, keto + enol), 3.73 (t, *J* = 1.3 Hz, 2H, keto), 1.88 – 1.69 (m, 6H, keto + enol), 1.34 (t, *J* = 7.2, 3H, enol), 1.31 (t, *J* = 7.2, 3H, keto). ^{19}F NMR (282 MHz, $CDCl_3$, 298 K): δ [ppm] = –99.4 (q, *J* = 19.2), –99.5 (q, *J* = 18.9).

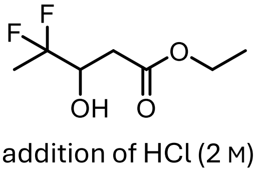

Ethyl (*E*)-4,4-difluoropent-2-enoate (S2). $NaBH_4$ (993.1 mg, 26.3 mmol) was added in portions to a solution of ester **S1** (5.18 g, 25 mmol) in toluene (170 mL) at 0 °C (bath temperature). The slurry was then stirred at room temperature for 6 h before the reaction was carefully quenched with aq. HCl (10 % *w/w*) at 0 °C. The aqueous layer was extracted with *tert*-butyl methyl ether (3 x 200 mL), and the combined organic phases were dried over $MgSO_4$ and concentrated in *vacuo*.

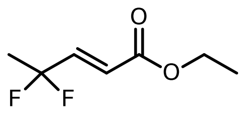

The crude alcohol product was dissolved in CH_2Cl_2 (44.9 mL) and the solution cooled to −78 °C (bath temperature). Methanesulfonyl chloride (2.1 mL, 27.5 mmol) and triethylamine (10.5 mL, 75

mmol) were added, the cooling bath was removed and the mixture stirred at room temperature for 1 h. After cooling to 0 °C, water was added and the solution was again warmed to room temperature and stirred for another 1 h. The organic layer was separated and the aqueous phase extracted with CH_2Cl_2 (2 x 80 mL). The combined organic layers were washed with conc. HCl (10 mL) and then evaporated in *vacuo*. Kugelrohr distillation (100 °C, 14 mbar) of the residue afforded the title compound as a colorless oil (2.3 g, 56 %). ^{1}H NMR (400 MHz, $CDCl_3$, 298 K): δ [ppm] = 6.86 (dt, *J* = 15.8, 10.6 Hz, 1H), 6.28 (dt, *J* = 15.8, 2.4 Hz, 1H), 4.27 (q, *J* = 7.1 Hz, 2H), 1.77 (t, *J* = 18.2 Hz, 3H), 1.34 (t, *J* = 7.1 Hz, 3H). ^{13}C NMR (101 MHz, CD_2Cl_2, 298 K): δ [ppm] = 165.4, 140.0 (t, $^2J_{CF}$ = 28.3 Hz), 125.0 (t, $^3J_{CF}$ = 8.7 Hz), 120.2 (t, $^1J_{CF}$ = 236.0 Hz), 61.5, 24.0 (t, $^2J_{CF}$ = 28.1 Hz), 14.3. ^{19}F NMR (282 MHz, $CDCl_3$, 298 K): δ [ppm] = –91.5 (m). IR (ATR): ṽ = 2987, 1725, 1665, 1387, 1369, 1310, 1290, 1244, 1193, 1167, 1127, 1031, 918, 874, 721, 577 cm^{-1}. HRMS (GC-CI, Ammonia): *m/z*: calcd. for [$C_7H_{11}F_2O_2$] [M+H$^+$]: 165.0722, found: 165.0722.

(2*E*,4*E*)-6,6-difluoro-*N*-methoxy-*N*-methylhepta-2,4-dienamide (22). Dibal-H (1 M in hexane, 13.4 mL, 13.40 mmol) was added dropwise at −78 °C (the temperature was constantly monitored with an internal thermometer) to a solution of ethyl (*E*)-4,4-difluoropent-2-enoate (**S2**) (2.0 g, 12.18 mmol) in CH_2Cl_2 (22.6 mL). The resulting yellow solution was stirred at this temperature for 1h.

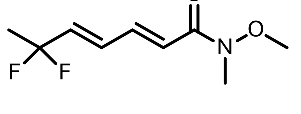

Diethyl (2-(methoxy(methyl)amino)-2-oxoethyl)phosphonate (**11**) (4.3 mL, 20.7 mmol) was carefully added to a suspension of NaH (497 mg, 20.7 mmol) in Et_2O (23.0 mL) at 0 °C (bath temperature) to give a colorless solution after stirring for 30 min. This solution was then added dropwise to the solution containing the reduced ester at −78 °C. Stirring was continued for 15 min at −78 °C and then for 3 h at room temperature. *tert*-Butyl methyl ether (500 mL) and sat. aq. Rochelle's salt solution (300 mL) were carefully added and the mixture was vigorously stirred until clear phase separation was reached. The organic phase was washed with HCl (2 M, 2 x 100 mL) and brine (100 mL) before it was dried over $MgSO_4$. After concentration in *vacuo*, the crude product was purified by flash chromatography (hexane/ethyl acetate, 9:1 then 8:2) to obtain the title compound as a clear oil (1.97 g, 79 %). ^{1}H NMR (400 MHz, CD_2Cl_2, 298 K): δ [ppm] = 7.30 – 7.19 (m, 1H), 6.74 – 6.63 (m, 2H), 6.16 – 6.03 (m, 1H), 3.70 (s, 3H), 3.22 (s, 3H), 1.74 (t, *J* = 18.2 Hz, 3H). ^{13}C NMR (101 MHz, CD_2Cl_2, 298 K): δ [ppm] = 166.3, 140.1 (t, $^4J_{CF}$ = 1.9 Hz), 134.0 (t, $^2J_{CF}$ = 27.4 Hz), 131.6 (t, $^3J_{CF}$ = 9.6 Hz), 124.1, 120.8 (t, $^1J_{CF}$ = 234.6 Hz), 62.3, 32.6, 24.2 (t, $^2J_{CF}$ = 28.7 Hz). ^{19}F NMR (282 MHz, CD_2Cl_2, 298 K): δ [ppm] = –89.0 (qdq, *J* = 18.2, 10.7, 2.6 Hz). IR (ATR): ṽ = 2972, 2942, 1641, 1613, 1418, 1384, 1312, 1250, 1219, 1114, 1000, 912, 815, 555 cm^{-1}. HRMS (GC-EI): *m/z*: calcd. for [$C_9H_{13}F_2NO_2$] [M]: 205.0909, found: 205.0907.

Ethyl (*E*)-5,5,5-trifluoropent-2-enoate (S3). Ethyl (diethoxyphosphoryl)acetate (9.73 mL, 49.1 mmol) was carefully added to a suspension of NaH (1.29 g, 53.5 mmol) in THF (23.0 mL) at 0 °C to give a colorless solution after stirring for 30 min. This solution was then added dropwise to a solution of 3,3,3-trifluoropropanal in THF at −78 °C. Stirring was continued for 15 min at −78 °C and then for 3 h at room temperature. The reaction was carefully quenched by the addition of aq. sat. NH_4Cl solution (100 mL). The aqueous layer was extracted with ethyl acetate (3 x 200 mL) before the combined organic layers were dried over $MgSO_4$. After concentration in *vacuo*, the residue was purified by flash chromatography (hexane/ethyl acetate, 98:2) to obtain the title compound as a clear oil (2.97 g, 37 %). ^{1}H NMR (400 MHz, $CDCl_3$, 298 K): δ [ppm] = 6.84 (dt, *J* = 15.7, 7.2 Hz, 1H), 6.06 (d, *J* = 16.1 Hz, 1H), 4.24 (q, *J* = 7.1 Hz, 2H), 3.07 – 2.96 (qdd, *J* = 10.4, 7.2, 1.5 Hz, 2H), 1.32 (t, *J* = 7.1 Hz, 3H). ^{19}F NMR (282 MHz, $CDCl_3$, 298 K): δ [ppm] = –65.5 (t, *J* = 10.4 Hz).

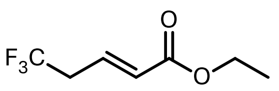

(2*E*,4*E*)-7,7,7-trifluoro-*N*-methoxy-*N*-methylhepta-2,4-dienamide (24a). Dibal-H (1 M in hexane, 13.4 mL, 13.40 mmol) was added dropwise at −78 °C (the temperature was constantly monitored with internal thermometer) to a solution of ethyl (*E*)-5,5,5-trifluoropent-2-enoate (**S3**) (2.2 g, 12.08 mmol) in CH_2Cl_2 (22.5 mL). The resulting yellow solution was stirred for 1h.

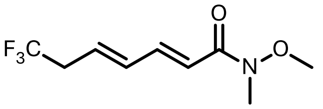

Diethyl (2-(methoxy(methyl)amino)-2-oxoethyl)phosphonate (**11**) (4.4 mL, 20.7 mmol) was carefully added to a suspension of NaH (492.8 mg, 20.5 mmol) in Et_2O (23.0 mL) at 0 °C to give a colorless solution after stirring for 30 min. This solution was then added dropwise to the solution containing the reduced ester at −78 °C. Stirring was continued for 15 min at −78 °C and then for 3 h at room temperature. *tert*-Butyl methyl ether (500 mL) and a sat. aq. Rochelle's salt solution (300 mL) were carefully added and the mixture was vigorously stirred until clear phase separation was reached. The organic layer was washed with HCl (2 M, 2 x 100 mL) and brine (100 mL) before it was dried over $MgSO_4$. After concentration in *vacuo*, the crude product was purified by flash chromatography (CH_2Cl_2/MeOH, 98:2) to obtain the title compound as a clear oil (0.57 g, 21 %).^{1}H NMR (400 MHz, CD_2Cl_2, 298 K): δ [ppm] = 7.25 (dd, *J* = 15.2, 10.1 Hz, 1H), 6.53 (d, *J* = 15.2 Hz, 1H), 6.50 – 6.42 (m, 1H), 6.03 – 5.93 (m, 1H), 3.70 (s, 3H), 3.21 (s, 3H), 2.98 (qdd, *J* = 10.7, 7.5, 1.4 Hz, 2H). ^{13}C NMR (101 MHz, CD_2Cl_2, 298 K): δ [ppm] = 166.8, 141.4, 135.4, 128.4 (q, $^3J_{CF}$ = 3.7 Hz), 126.2 (q, $^1J_{CF}$ = 276.6 Hz), 121.2, 62.2, 37.7 (q, $^2J_{CF}$ = 30.0 Hz), 32.6. ^{19}F NMR (282 MHz, CD_2Cl_2, 298 K): δ [ppm] = –66.4 (t, *J* = 10.7 Hz). IR (ATR): ṽ = 2971, 2941, 1663, 1634, 1416, 1383, 1252, 1135, 1001, 847, 620 cm^{-1}. HRMS (GC-EI): *m/z*: calcd. for [$C_9H_{12}F_3NO_2$] [M]: 223.0814, found: 223.0815.

Benzyl (*E*)-4,4,5,5,5-pentafluoropent-2-enoate (S4). A solution of ethyl 2,2,3,3,3-pentafluoropropanoate (10.0 mL, 68.0 mmol) in Et_2O (15 mL) was added dropwise over 15 min to a suspension of $LiAlH_4$ (860 mg, 22.7 mmol) in Et_2O (40 mL) at –78 °C (bath temperature). The resulting mixture was stirred for 3 h at this temperature before the reaction was carefully quenched with H_2SO_4 (1 M, 50 mL) and allowed to warm to room temperature. The biphasic mixture was separated and the aqueous layer was extracted with Et_2O (3 x 20 mL). The combined organic extracts were sequentially washed with H_2SO_4 (1 M) and brine, dried over anhydrous Na_2SO_4, filtered and carefully evaporated in vacuo to provide a colorless oil which was further purified by distillation (bath temperature: 130 °C). The fraction boiling at ≈95°C was collected and found to consist of a mixture of the hydrate and the ethyl hemiacetal of 2,2,3,3,3-pentafluoropropanal (8.99 g).

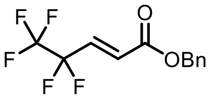

Benzyl dimethylphosphonoacetate (2.3 mL, 10.8 mmol) and Et_3N (1.7 mL, 12.0 mmol) was added to a solution of LiBr (1.15 g, 13.2 mmol) in THF (8.0 mL) at 0 °C, causing the formation of a white precipitate. After stirring for 30 min, the mixture of 2,2,3,3,3-pentafluoropropanal hydrate and hemiacetal (2.0 g) in THF (2 mL) was added slowly. The mixture was stirred for 1 h at ambient temperature before the reaction was quenched with HCl (2 M, 6 mL) and pH 7 phosphate buffer (10 mL). The aqueous layer was extracted with, the combined organic phases were dried over Na_2SO_4 and concentrated under reduced pressure. The residue was purified by flash chromatography (silica; *tert*-butyl methyl ether /hexanes, 1:10) to give the title compound as a colorless oil (2.40 g, 57% over 2 steps). ^{1}H NMR (400 MHz, $CDCl_3$, 298 K): δ [ppm] = 7.46 – 7.32 (m, 5H), 6.85 (dtd, *J* = 15.8, 11.7, 0.9 Hz, 1H), 6.60 (dt, *J* = 15.8, 2.1 Hz, 1H), 5.26 (s, 2H). ^{13}C NMR (101 MHz, $CDCl_3$) δ 163.6, 135.0, 131.0 (t, J_{CF} = 24.1 Hz), 130.8 (t, J_{CF} = 8.3 Hz), 128.9, 128.7, 118.6 (qt, J_{CF} = 285.7, 36.6 Hz), 111.7 (tq, J_{CF} = 252.0, 39.3 Hz), 67.7 (one sp^2 carbon missing due to signal overlap). ^{19}F NMR (282 MHz, $CDCl_3$) δ –84.6 (t, *J* = 3.0 Hz), -117.2 (q, *J* = 2.8 Hz). IR (ATR): ṽ = 1731, 1306, 1282, 1196, 1119, 1042, 972, 738, 696, 663, 592, 528, 480 cm^{-1}.

(2*E*,4*E*)-6,6,7,7,7-Pentafluoro-*N*-methoxy-*N*-methylhepta-2,4-dienamide (24b). Dibal-H (1 M in hexane, 3.93 mL, 3.89 mmol) was added dropwise at −78 °C (the temperature was constantly monitored with internal thermometer) to a solution of benzyl (*E*)-4,4,5,5,5-pentafluoropent-2-enoate (**S4**) (1.0 g, 3.57 mmol) in CH_2Cl_2 (7.5 mL). The resulting solution was stirred for 1h.

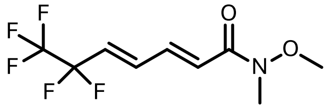

Diethyl (2-(methoxy(methyl)amino)-2-oxoethyl)phosphonate (**11**) (1.25 mL, 6.00 mmol) was carefully added to a suspension of NaH (144.2 mg, 6.00 mmol) in Et_2O (6.7 mL) at 0 °C to give a colorless solution after stirring for 30 min. This solution was then added dropwise to the solution containing the reduced ester at −78 °C. Stirring was continued for 15 min at −78 °C and then for 3 h at room temperature. *tert*-Butyl methyl ether (200 mL) and sat. aq. Rochelle salt solution (100 mL) were carefully added and the mixture was vigorously stirred until a clear phase separation was reached. The organic phase was washed with HCl (2 M, 2 x 40 mL) and brine (40 mL) before it was dried over $MgSO_4$. After concentration in *vacuo*, the crude product was purified by flash chromatography (hexane/ethyl acetate, 7:3) to obtain the title compound as a clear oil (0.47 g, 65 %).^{1}H NMR (400 MHz, CD_2Cl_2, 298 K): δ [ppm] = 7.34 – 7.23 (m, 1H), 7.03 – 6.93 (m, 1H), 6.80 (d, J = 15.2 Hz, 1H), 6.11 – 5.98 (m, 1H), 3.71 (s, 3H), 3.24 (s, 3H). ^{13}C NMR (101 MHz, CD_2Cl_2, 298 K): δ [ppm] = 165.6, 138.3 (t, $^4J_{CF}$ = 2.0 Hz), 138.0 (t, $^3J_{CF}$ = 9.3 Hz), 127.1 (t, $^4J_{CF}$ = 2.0 Hz), 123.1 (t, $^2J_{CF}$ = 23.1 Hz), 119.27 (dt, $^1J_{CF}$ = 285.6, $^2J_{CF}$ = 37.9 Hz), 113.2 – 111.9 (m), 62.4, 32.6. ^{19}F NMR (282 MHz, CD_2Cl_2, 298 K): δ [ppm] = –85.4, –116.0. IR (ATR): ṽ = 1660, 1638, 1613, 1423, 1388, 1306, 1195, 1101, 1032, 997, 737 cm^{-1}. HRMS (GC-EI): *m/z*: calcd. for [$C_9H_{10}F_5NO_2$] [M]: 259.0626, found: 259.0625.

Reaction Optimization

Table S9. Aliphatic Aldehydes

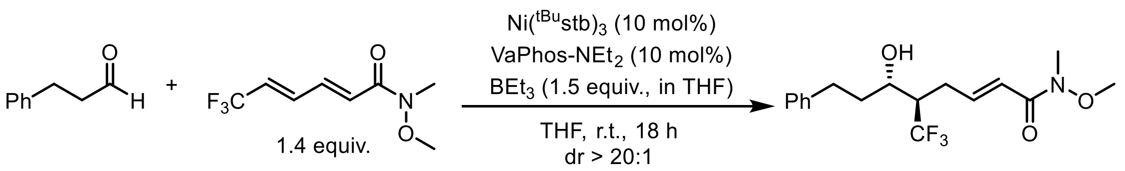

Entry	Solvent	Yield (NMR)	Regioisomer (NMR Yield)	rr	ee
**1**	**THF**	**72 % (77 %)**	**18 %**	**4:1**	**93 %**
2	toluene	83 % (96 %)	3 %	> 20:1	85 %
3	pentane	70 % (81 %)	3%	> 20:1	84 %
4	Et_2O	83 % (95 %)	4 %	> 20:1	88 %
5	1,4-dioxane	84 % (96 %)	3 %	> 20:1	88 %
6	trifluorotoluene	82 % (90 %)	3 %	> 20:1	84 %

Table S10. Aromatic Aldehydes

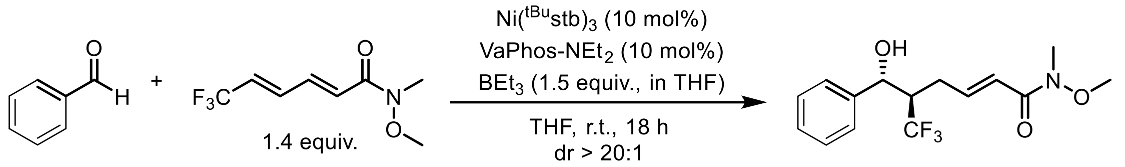

Entry	Variation	Yield (NMR)	Regioisomer (NMR Yield)	rr	ee
1	none	79 % (91 %)	9 %	10:1	81 %
2	in Et_2O, –10 °C	82 % (94 %)	4 %	> 20:1	66 %
**3**	**–10 °C**	**68 %**	**n.d.**	**10:1**	**86 %**
4	in toluene	87 % (95 %)	4 %	> 20:1	56 %

The highest enantioselectivity was obtained when running the reaction in THF for both aromatic and aliphatic aldehydes, although other solvents led to improved regioselectivity. Attempts were made to increase the ee by lowering the reaction temperature with Et_2O as a solvent. However, the yield dropped significantly at temperatures ≤ –10 °C, especially for aliphatic aldehydes (the rr remained unchanged).
In case of aromatic- and α/β-unsaturated aldehydes, a reaction temperature of –10 °C was found to be the best compromise concerning ee and yield.

Control Experiments: Ligands

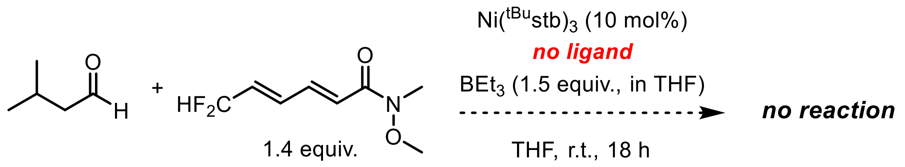

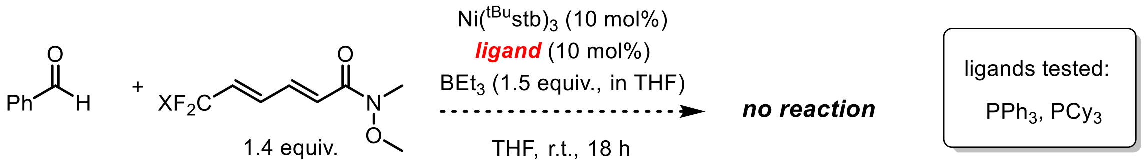

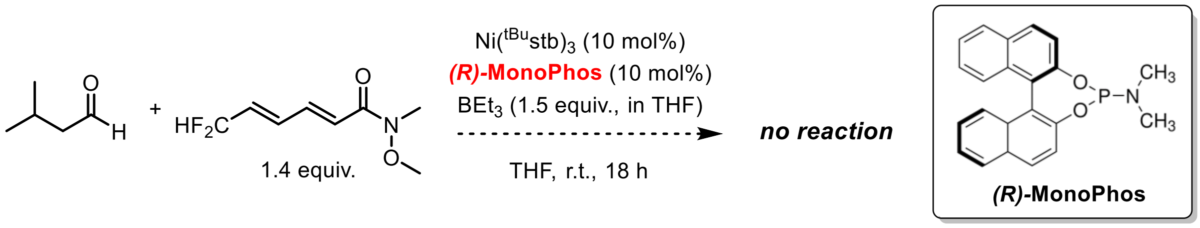

Control Experiments: Nickel Sources

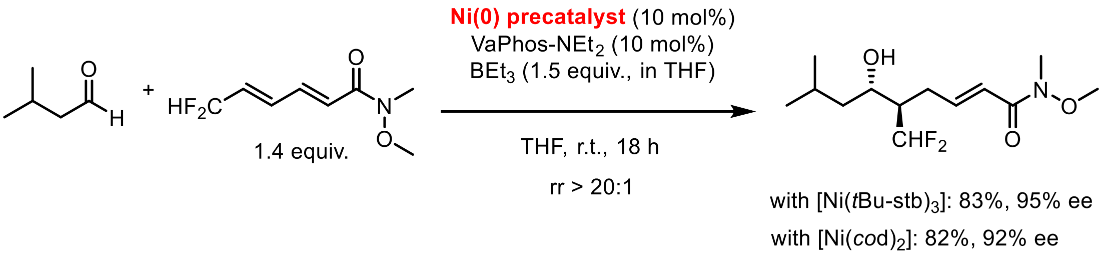

Determination of the Regioisomeric Ratio (rr)

The regioisomeric ratio (rr) was determined by ^{19}F NMR analysis of the crude products after aqueous workup. Figure S9 shows the ^{19}F NMR spectrum of the crude mixture formed upon reductive coupling of benzaldehyde with diene **12** under standard reaction conditions, as well as the ^{19}F NMR spectrum of **12** itself and of the purified product **17a**. As these compounds have significantly different chemical shifts, the crude spectrum allows for accurate determination of the rr (Figure S10). This fact also applies to the products derived from the difluorosorbamide **15** (Figures S11 and S12).

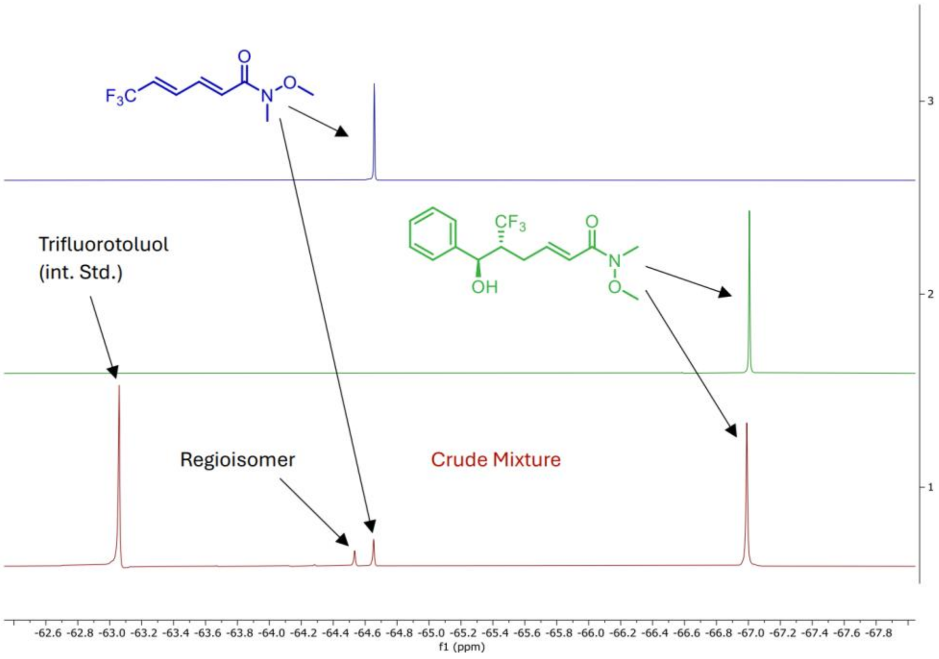

Figure S9. ^{19}F NMR spectrum of the crude mixture formed upon reductive coupling of trifluorodiene **12** and benzaldehyde (red), stacked with the spectrum of diene **12** (blue) and the purified product **17a** (green).

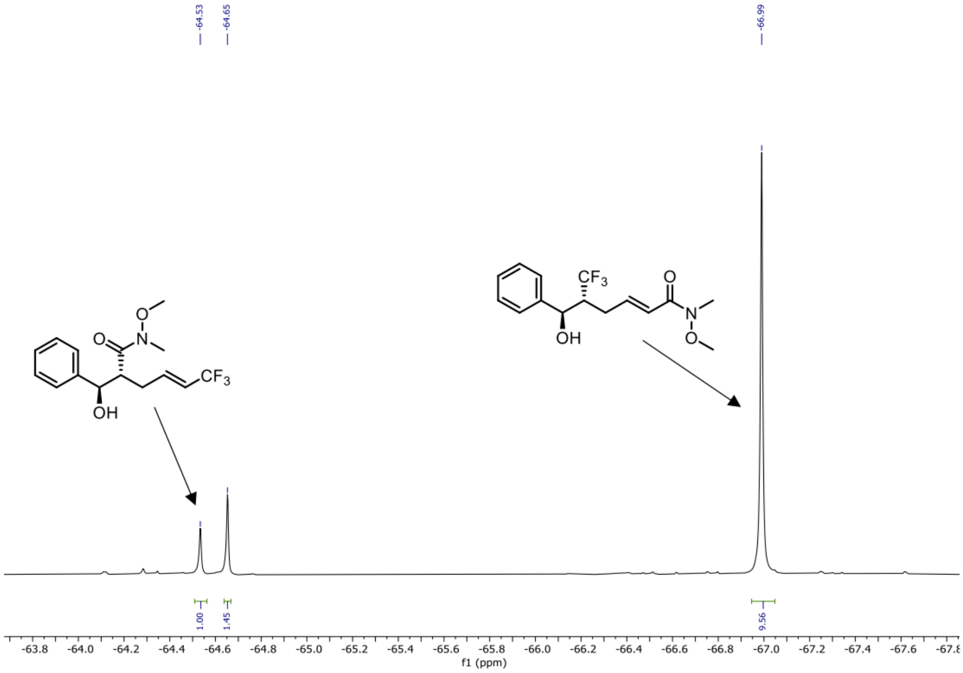

Figure S10. Determination of the rr from the ^{19}F NMR of the crude product **17a**; rr ≈ 10:1.

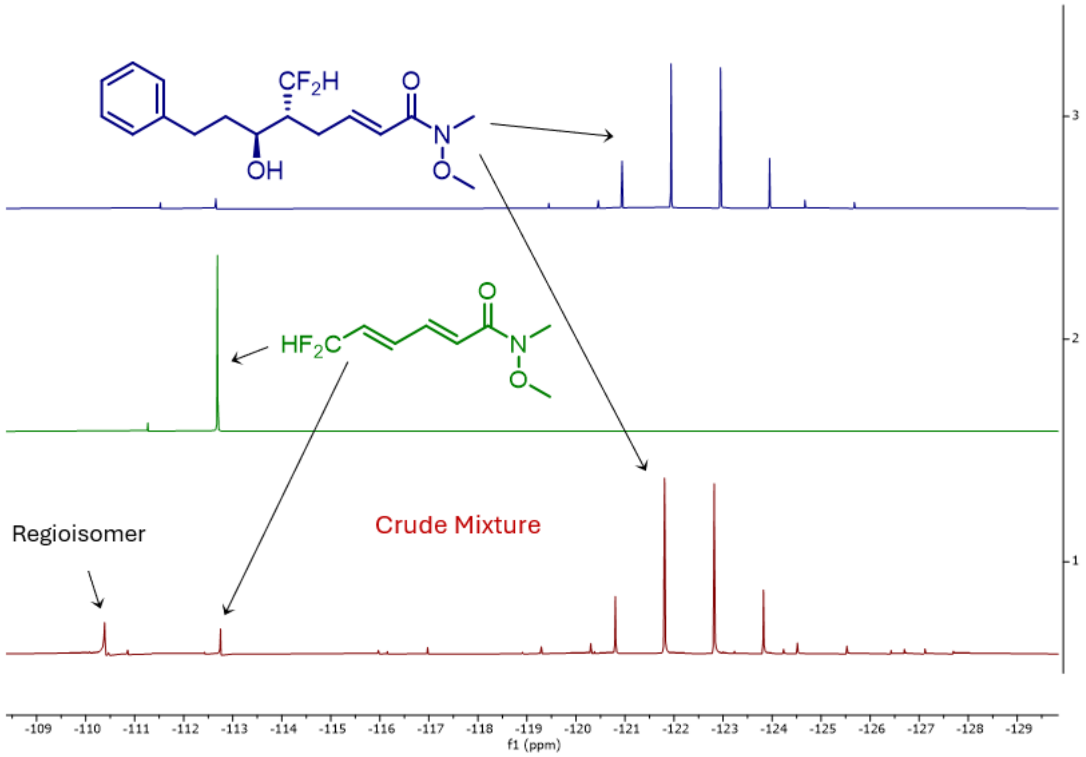

Figure S11. ^{19}F NMR spectrum of the crude mixture formed upon reductive coupling of difluorodiene **15** and hydrocinnamaldehyde (red), stacked with the spectrum of **15** (green) and the purified product **19d**.

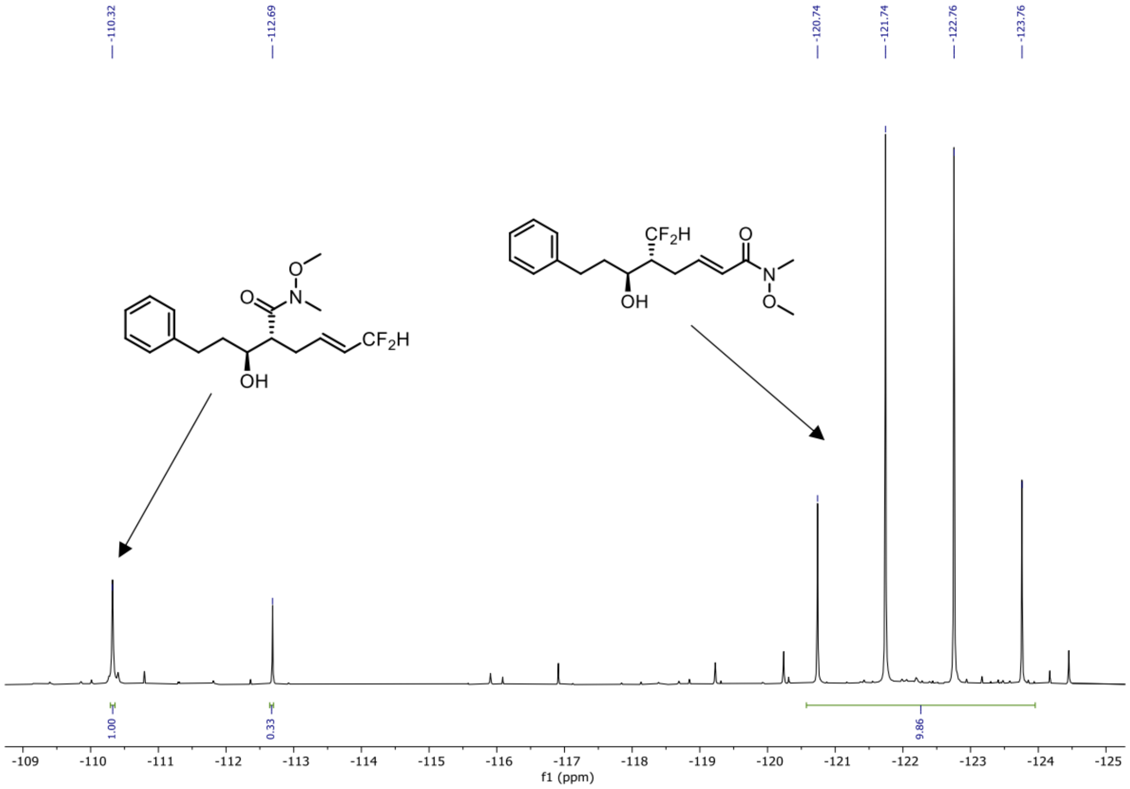

Figure S12. Determination of the rr from the ^{19}F NMR of the crude product **19d**; rr ≈10:1.

General Procedures

General Procedure A (For the Reductive Coupling of Aromatic or α,β-Unsaturated Aldehydes). A flame-dried Schlenk flask was charged with tris(*trans*-1,2-bis(4-*tert*-butylphenyl)ethene)nickel(0) (Ni(*t*Bu-stb)$_3$, **16**) (18.7 mg, 0.02 mmol), phosphoramidite (*R*)-**L1** (≥98% ee, 12.8 mg, 0.02 mmol) and the respective diene (0.28 mmol). The Schlenk flask was evacuated and refilled with argon (3 cycles). THF (0.4 mL) and triethylborane (1 M in THF, 0.30 mL, 0.30 mmol) were added and the resulting solution was stirred for 5 min. The respective aldehyde (0.20 mmol) was added under an argon counterflow, the flask was sealed and the mixture stirred at –10 °C for 18 h. The reaction was quenched *via* the oxidative work-up procedure (see below). Purification of the crude material by flash chromatography furnished the product in analytically pure form.

General Procedure B (For Aliphatic Aldehydes). A flame-dried Schlenk flask was charged with tris(*trans*-1,2-bis(4-*tert*-butylphenyl)ethene)nickel(0) (Ni(*t*Bu-stb)$_3$, **16**) (18.7 mg, 0.02 mmol), phosphoramidite (*R*)-**L1** (≥98% ee, 12.8 mg, 0.02 mmol) and the respective diene (0.28 mmol). The Schlenk flask was evacuated and refilled with argon (3 cycles). THF (0.4 mL) and triethylborane (1 M in THF, 0.30 mL, 0.30 mmol) were added and the resulting solution was stirred for 5 min. The respective aldehyde (0.20 mmol) was added under an argon counterflow, the flask was sealed and the mixture stirred at room temperature for 18 h. The reaction was quenched *via* the oxidative work-up procedure (see below). Purification of the crude material by flash chromatography furnished the product in analytically pure form.

Oxidative Work-up Procedure. ***Note:*** *The product initially formed is the corresponding -OBEt$_2$ adduct. The O-B bond is fairly stable towards hydrolysis but can be rapidly cleaved under oxidative conditions to give the desired free alcohol.*

The reaction mixture was cooled to 0 °C and pH 7 phosphate buffer solution (1.5 mL) and aq. H_2O_2 (30% *w/w* in water, ≈0.3 mL) was added. The mixture was diluted with ethyl acetate (2 mL) and vigorously stirred for 1 h at 0 °C. Additional ethyl acetate (10 mL) and water (5 mL) were added, the organic phase was separated, and the aqueous layer was extracted with ethyl acetate (2 × 10 mL). The combined organic layers were washed with sat. aq. $Na_2S_2O_3$ (15 mL) and brine (15 mL), dried over $MgSO_4$, filtered, and concentrated *in vacuo*.

Notes

1. THF was distilled under argon and stored in Schlenk flasks under argon for long- term use. Karl-Fischer-titration was used to check that the water content did not exceed 20 ppm.
2. The presence of residual chlorinated solvents ($CHCl_3$, CH_2Cl_2 etc.) in any of the reaction partners can lead to catalyst deactivation.
3. Triethylborane solutions were prepared from neat triethylborane and the respective solvent.

   Note: *Triethylborane is pyrophoric and must be handled carefully under rigorously oxygen-free and water-free conditions.*
4. The CF_2H-/CF_3-substituted dienes showed good stability and were handled and weighed in air. They were kept in the freezer at –18 °C for long-term storage.
5. Tris(*trans*-1,2-bis(4-*tert*-butylphenyl)ethene)nickel(0) (**16**) can be weighed in air, yet was stored in the freezer at –18 °C.

6. Although tris(*trans*-1,2-bis(4-*tert*-butylphenyl)ethene)nickel(0) (**16**) is air-stable in the solid state, care must be taken once it has been dissolved. Therefore, the ligand was always added *before* the solvent as it helps stabilize the Ni(0) complex should any residual oxygen be present in solution. Likewise, the diene seems to stabilize the nickel species, while triethylborane quenches any remaining oxygen.

9. Small-scale reactions were carried out in sealed Schlenk flasks. However, ethylene gas is released from reduction of the nickel catalyst by triethylborane and care must be taken to use reaction vessels that can withstand the increase in pressure caused by this gas evolution.

   Note: *For larger scale reactions, it is essential to use a gas bubbler to prevent build-up of overpressure and possible explosion of the flask.*

10. The regioisomeric ratio was determined by ^{19}F NMR analysis of the crude material after the aqueous workup. Unless stated otherwise, the dr was > 20:1.

11. Due to the high viscosity of some of the products, remaining solvent traces were seen in some of the recorded NMR spectra. In all these cases, a comment is added to the procedure of the respective compound and the yield was calculated taking the added mass into account

Gram-Scale Reaction

(5*R*,6*S*,*E*)-5-(Difluoromethyl)-6-hydroxy-*N*-methoxy-*N*,8-dimethyl-non-2-enamide (19c). A flame-dried Schlenk flask equiped with a gas inlet connected to an argon/vacuum manifold was charged with tris(*trans*-1,2-bis(4-*tert*-butylphenyl)ethene)nickel(0) (Ni(*t*Bu-stb)$_3$, **16**) (374.4 mg, 0.4 mmol), phosphoramidite (*R*)-**L1** (≥98% ee, 255.9 mg, 0.4 mmol) and diene **15** (1.07 g, 5.6 mmol). The Schlenk flask was evacuated and refilled with argon (3 cycles). THF (8.1 mL) and triethylborane (1 M in THF, 6.0 mL, 6.0 mmol) were added and the resulting solution was stirred for 5 min. Isovaleraldehyde (0.43 m, 4.0 mmol) was added under an argon counterflow and the mixture stirred under a gentle flow of argon at room temperature for the 18 h. The reaction mixture was cooled to 0 °C before pH 7 phosphate buffer solution (15 mL) and aq. H_2O_2 (30% *w/w* in water, 5 mL) were added. The mixture was diluted with ethyl acetate (20 mL) and vigorously stirred for 1 h at 0 °C. Additional ethyl acetate (150 mL) and water (50 mL) were added, the organic phase was separated, and the aqueous layer was extracted with ethyl acetate (2 × 10 mL). The combined organic layers were washed with sat. aq. $Na_2S_2O_3$ (150 mL) and brine (200 mL), dried over $MgSO_4$, filtered and concentrated *in vacuo*. Purification of the crude material (rr > 20:1) by automated flash chromatography (25 g column, 5-60 % gradient of cyclohexane/ethyl acetate) gave the title compound as a colorless oil (1.04 g, 93 % yield, dr > 20:1, 95 % ee).

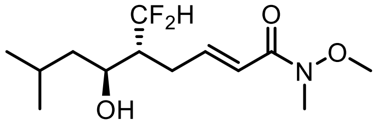

$[\alpha]_D^{20}$ = –14.6 (c = 0.8, CH_2Cl_2). ^{1}H NMR (400 MHz, CD_2Cl_2, 298 K): δ [ppm] = 6.97 – 6.85 (m, 1H), 6.48 (d, *J* = 15.3 Hz, 1H), 5.94 (td, *J* = 56.4, 4.2 Hz, 1H), 4.03 – 3.93 (m, 1H), 3.68 (s, 3H), 3.19 (s, 3H), 2.53 – 2.44 (m, 3H), 2.12 – 1.98 (m, 1H), 1.80 – 1.68 (m, 1H), 1.52 – 1.41 (m, 1H), 1.28 – 1.20 (m, 1H), 0.92 (dd, *J* = 15.3, 6.6 Hz, 6H). ^{19}F NMR (282 MHz, CD_2Cl_2, 298 K): δ [ppm] = –121.3 (d, *J* = 282.3 Hz), –123.5 (d, *J* = 282.3 Hz). ^{13}C NMR (101 MHz, CD_2Cl_2, 298 K): δ [ppm] = 166.7, 145.2, 121.0, 118.5 (t, $^1J_{CF}$ = 241.4 Hz), 67.6 (t, $^3J_{CF}$ = 5.0 Hz), 62.1, 48.0 (t, $^2J_{CF}$ = 17.6 Hz), 43.6, 32.5, 26.8 (t, $^3J_{CF}$ = 4.7 Hz), 25.0, 23.6, 21.9. IR (ATR): ṽ = 3415, 2957, 2937, 2871, 1660, 1618, 1467, 1425, 1386, 1178, 1109, 1090, 1040, 997, 913, 690, 620, 528 cm^{-1}. HRMS (ESI$^+$): *m/z*: calcd. for $[C_{13}H_{23}F_2NO_3Na]^+$ [M+Na]$^+$: 302.1538, found: 320.1537.

The ee was determined by HPLC analysis: Chiralpak 150 mm IG-3, 3 µm, 4.6 mm ∅, CH_3CN/H_2O = 40:60, v = 1.0 mL/min, λ = 215 nm, t(minor) = 10.18 min, t(major) = 12.73 min (racemate: left, enantioenriched sample: right).

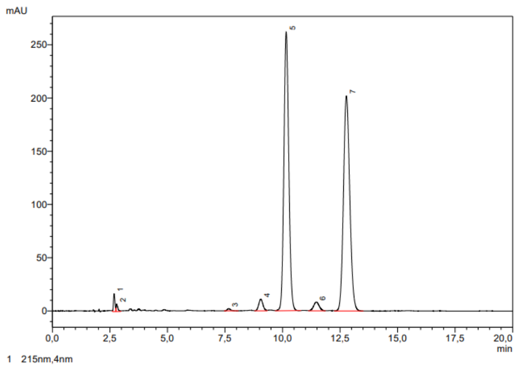

PDA Ch1 215nm

Peak #	Ret. Time	Area %	Name
1	2,68	0,95	
2	2,79	0,46	
3	7,66	0,26	
4	9,06	1,73	
5	10,16	47,43	1. Enantiomer
6	11,47	1,77	
7	12,78	47,40	2. Enantiomer
Total		100,00	

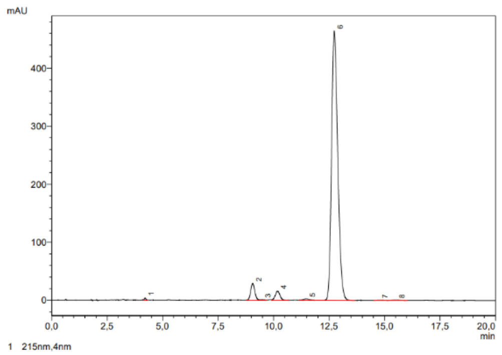

PDA Ch1 215nm

Peak #	Ret. Time	Area %	Name
1	4,21	0,21	
2	9,06	3,90	
3	9,48	0,18	
4	10,18	2,51	1. Enantiomer
5	11,48	0,56	
6	12,73	92,31	2. Enantiomer 94.7 % ee
7	14,75	0,12	
8	15,51	0,19	
Total		100,00	

Characterization Data

Trifluoromethyl Derivatives

(*R*,*E*)-6,6,6-Trifluoro-5-((*R*)-hydroxy(phenyl)methyl)-*N*-methoxy-*N*-methylhex-2-enamide

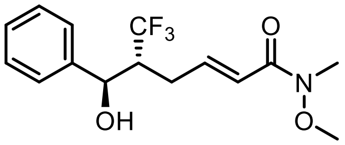

(17a). The title compound was prepared from benzaldehyde (20.2 µL, 0.20 mmol) and diene **12** (58.6 mg, 0.28 mmol) according to the general procedure **A**. The crude material (rr ≈10:1) was purified by automated flash chromatography (10 g column, 5–60 % gradient of cyclohexane/ethyl acetate) to afford the product as a colorless oil (43.3 mg, 68 % yield, dr > 20:1, 86 % ee).

$[\alpha]_D^{20}$ = +10.8 (c = 1.1, CH_2Cl_2). ^{1}H NMR (400 MHz, CD_2Cl_2, 298 K): δ [ppm] = 7.40 – 7.27 (m, 5H), 6.72 – 6.62 (m, 1H), 6.32 (dt, *J* = 15.5, 1.5 Hz, 1H), 5.25 – 5.21 (m, 1H), 3.62 (s, 3H), 3.13 (s, 3H), 2.81 – 2.48 (m, 4H). ^{13}C NMR (101 MHz, CD_2Cl_2, 298 K): δ [ppm] = 166.5, 143.8, 141.9, 128.9, 128.2, 127.6 (q, $^1J_{CF}$ = 281.5 Hz), 126.2, 121.2, 70.6, 62.0, 50.4 (q, $^2J_{CF}$ = 23.1 Hz), 32.4, 26.1. ^{19}F NMR (282 MHz, CD_2Cl_2, 298 K): δ [ppm] = –67.0 (s). IR (ATR): ṽ = 3371, 2931, 1661, 1619, 1384, 1263, 1194, 1152, 1127, 1073, 1046, 1000, 766, 704, 613 cm^{-1}. HRMS (ESI$^+$) : *m/z*: calcd. for $[C_{15}H_{19}F_3NO_3]^+$ $[M+H]^+$: 318.1311, found: 318.1315.

The ee was determined by HPLC analysis: Chiralpak 150 mm IB-N-3, 3 µm, 4.6 mm ∅, *n*-heptane/2-propanol = 90:10, v = 1.0 mL/min, λ = 210 nm, t(minor) = 8.12 min, t(major) = 11.21 min (racemate: left, enantioenriched sample: right).

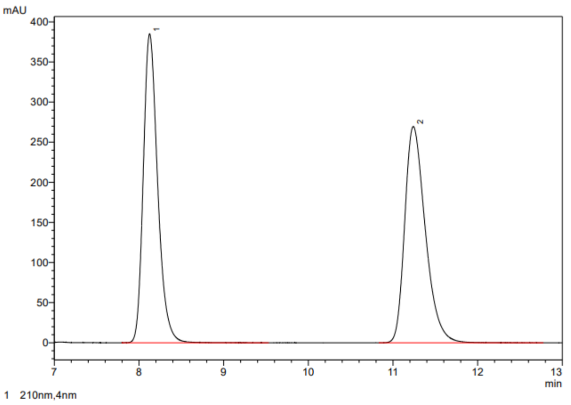

PDA Ch1 210nm

Peak #	Ret. Time	Area %	Name
1	8,13	49,84	1. Enantiomer
2	11,24	50,16	2. Enantiomer
Total		100,00	

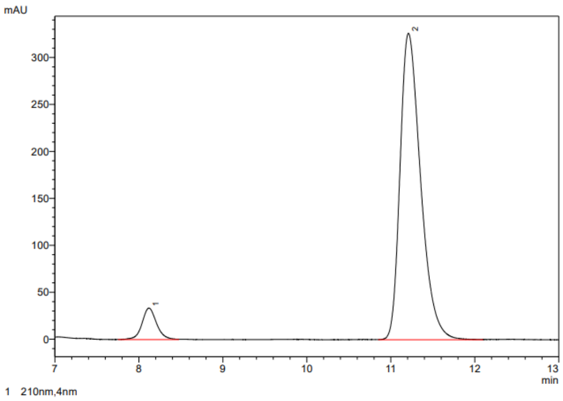

PDA Ch1 210nm

Peak #	Ret. Time	Area %	Name
1	8,12	6,82	1. Enantiomer
2	11,21	93,18	2. Enantiomer 86.4 % ee
Total		100,00	

(5*R*,6*S*,*E*)-6-Hydroxy-*N*-methoxy-*N*-methyl-8-phenyl-5-(trifluoromethyl)oct-2-enamide

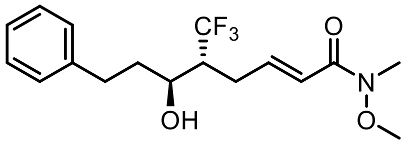

(17b). The title compound was prepared from 3-phenylpropanal (26.3 µL, 0.20 mmol) and diene **12** (58.6 mg, 0.28 mmol) according to the general procedure **B**. The crude material (rr ≈ 4:1) was purified by automated flash chromatography (10 g column, 5–60 % gradient of cyclohexane/ethyl acetate) to afford the product as a colorless oil (47.5 mg, 69 % yield, dr > 20:1, 93 % ee).

$[\alpha]_D^{20}$ = –6.7 (c = 0.7, CH_2Cl_2). ^{1}H NMR (400 MHz, CD_2Cl_2, 298 K): δ [ppm] = 7.33 – 7.15 (m, 5H), 6.97 – 6.82 (m, 1H), 6.49 (d, *J* = 15.3, 1.5 Hz, 1H), 4.09 – 3.99 (m, 1H), 3.65 (s, 3H), 3.19 (s, 3H), 2.89 – 2.77 (m, 1H), 2.72 – 2.56 (m, 3H), 2.45 – 2.29 (m, 1H), 2.27– 2.12 (br, 1H),1.99 – 1.71 (m, 2H). ^{13}C NMR (101 MHz, CD_2Cl_2, 298 K): δ [ppm] = 166.5, 144.1, 141.9, 128.8 (d, $^4J_{CF}$ = 4.0 Hz), 128.1 (q, $^1J_{CF}$ = 281.3 Hz), 126.4, 121.4, 68.5 (q, $^3J_{CF}$ = 2.8 Hz), 62.1, 48.4 (q, $^2J_{CF}$ = 23.1 Hz), 37.2, 32.6, 32.5, 27.3, 26.8 (q, $^3J_{CF}$ = 2.5 Hz). ^{19}F NMR (282 MHz, CD_2Cl_2, 298 K): δ [ppm] = –67.6 (s). IR (ATR): ṽ = 3417, 2937, 1662, 1618, 1426, 1386, 1268, 1157, 1127, 998, 927, 750, 701 cm^{-1}. HRMS (ESI$^+$): *m/z*: calcd. for $[C_{17}H_{22}F_3NO_3Na]^+$ [M+Na]$^+$: 368.1444 , found: 368.1445.

The ee was determined by HPLC analysis: Chiralpak 150 mm IB-N-3, 3 µm, 4.6 mm ∅, *n*-heptane/2-propanol = 90:10, v = 1.0 mL/min, λ = 210 nm, t(minor) = 9.54 min, t(major) = 16.54 min (racemate: left, enantioenriched sample: right).

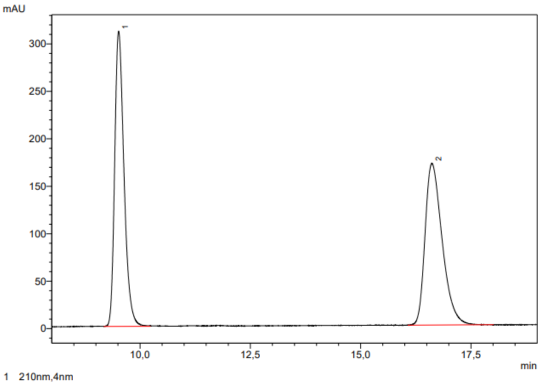

PDA Ch1 210nm

Peak #	Ret. Time	Area %	Name
1	9,51	49,84	1. Enantiomer
2	16,61	50,16	2. Enantiomer
Total		100,00	

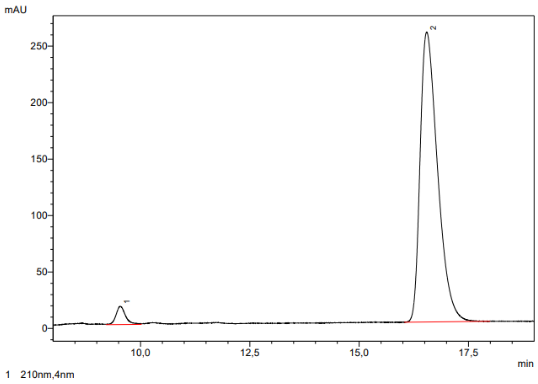

PDA Ch1 210nm

Peak #	Ret. Time	Area %	Name
1	9,54	3,34	1. Enantiomer
2	16,54	96,66	2. Enantiomer 93.3 % ee
Total		100,00	

(*R*,*E*)-5-((*S*)-Cyclohexyl(hydroxy)methyl)-6,6,6-trifluoro-*N*-methoxy-*N*-methylhex-2-enamide (17c). The title compound was prepared from cyclohexyl carbaldehyde (24.2 µL, 0.20 mmol) and diene **12** (83.7 mg, 0.40 mmol) according to the general procedure **A**. The crude material (rr ≈ 6:1) was purified by automated flash chromatography (10 g column, 5–60 % gradient of cyclohexane/ethyl acetate) to afford the product as a colorless oil (49.7 mg, 77 % yield, dr > 20:1, 87 % ee).

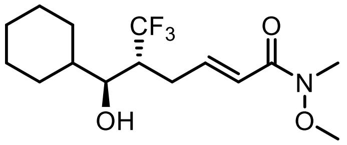

$[\alpha]_D^{20}$ = –19.6 (c = 1.1, CH_2Cl_2). ^{1}H NMR (400 MHz, CD_2Cl_2, 298 K): δ [ppm] = 6.97 - 6.84 (m, 1H), 6.50 (dt, *J* = 15.3, 1.4 Hz, 1H), 3.68 (s, 3H), 3.19 (s, 3H), 2.67 - 2.45 (m, 3H), 2.05 - 1.91 (m, 2H), 1.84 - 1.55 (m, 5H), 1.30 - 1.14 (m, 3H), 1.05 -0.79 (m, 3H). ^{13}C NMR (101 MHz, CD_2Cl_2, 298 K): δ [ppm] = 166.6, 144.3, 128.6 (q, $^1J_{CF}$ = 281.3 Hz), 121.2, 73.2 (q, $^3J_{CF}$ = 2.8 Hz), 62.1, 44.9 (q, $^2J_{CF}$ = 23.1 Hz), 41.3 (2C), 32.5, 29.6, 29.4, 26.6, 26.3, 26.2. ^{19}F NMR (282 MHz, CD_2Cl_2, 298 K): δ [ppm] = –67.9 (s). IR (ATR): ṽ = 3419, 2926, 2853, 1661, 1619, 1448, 1386, 1265, 1253, 1156, 1126, 1074, 998, 961, 938, 688, 530 cm^{-1}. HRMS (ESI$^+$): *m/z*: calcd. for $[C_{15}H_{24}F_3NO_3Na]^+$ $[M+Na]^+$: 346.1600, found: 346.1597.

The ee was determined by HPLC analysis: Chiralpak 150 mm IB-N-3, 3 µm, 4.6 mm ∅, *n*-heptane/2-propanol = 98:2, v = 1.0 mL/min, λ = 215 nm, t(minor) = 13.64 min, t(major) = 12.07 min (racemate: left, enantioenriched sample: right).

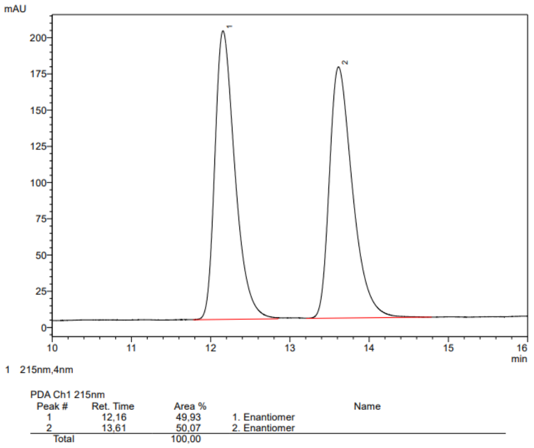

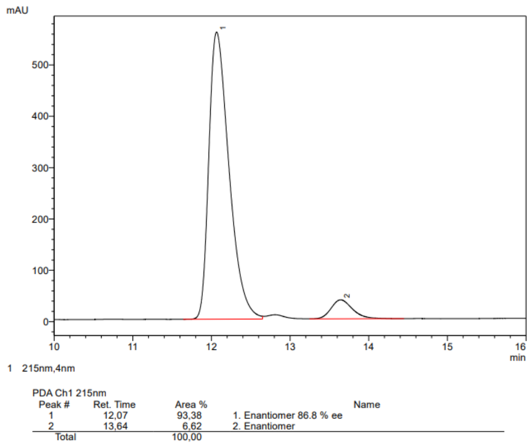

(5*R*,6*S*,*E*)-6-Hydroxy-*N*-methoxy-*N*,8-dimethyl-5-(trifluoromethyl)nona-2,7-dienamide (17d).

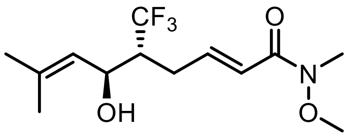

The title compound was prepared from 3-methylbut-2-enal (19.3 µL, 0.20 mmol) and diene **12** (58.6 mg, 0.28 mmol) according to the general procedure **A**. The crude material (rr > 20:1) was purified by automated flash chromatography (10 g column, 5–60 % gradient of cyclohexane/ethyl acetate) to afford the product as a colorless oil (37.8 mg, 64 % yield, dr > 20:1, 92 % ee).

$[\alpha]_D^{20}$ = + 6.7 (c = 0.8, CH_2Cl_2). ^{1}H NMR (400 MHz, CD_2Cl_2, 298 K): δ [ppm] = 6.96 - 6.82 (m, 1H), 6.49 (d, J = 15.3 Hz, 1H), 5.28 (d, J = 8.7, 1H), 4.70 (dd, J = 8.7, 4.0 Hz, 1H), 3.68 (s, 3H), 3.19 (s, 3H), 2.62 (app. t, J = 6.8 Hz, 2H), 2.49 - 2.31 (m, 1H), 2.00 - 1.85 (m, 1H), 1.74 (s, 3H), 1.68 (s, 3H). ^{13}C NMR (101 MHz, CD_2Cl_2, 298 K): δ [ppm] = 166.6, 144.0, 137.2, 127.7 (q, $^1J_{CF}$ = 281.1), 124.9, 121.4, 66.3 (q, $^3J_{CF}$ = 3.0 Hz), 62.1, 49.1 (q, $^1J_{CF}$ = 22.8 Hz), 32.5, 27.4 (q, $^3J_{CF}$ = 2.5 Hz), 25.9, 18.3. ^{19}F NMR (282 MHz, CD_2Cl_2, 298 K): δ [ppm] = –67.0 (s). IR (ATR): ṽ = 3407, 2936, 1662, 1621, 1441, 1382, 1262, 1158, 1101, 1034, 999, 962 cm^{-1}. HRMS (ESI$^+$): *m/z*: calcd. for $[C_{13}H_{20}F_3NO_3Na]^+$ $[M+Na]^+$: 318.1287, found: 318.1290.

The ee was determined by HPLC analysis: Chiralpak 150 mm IB-N-3, 3 µm, 4.6 mm ∅, *n*-heptane/2-propanol = 95:5, v = 1.0 mL/min, λ = 210 nm, t(minor) = 9.33 min, t(major) = 8.11 min (racemate: left, enantioenriched sample: right).

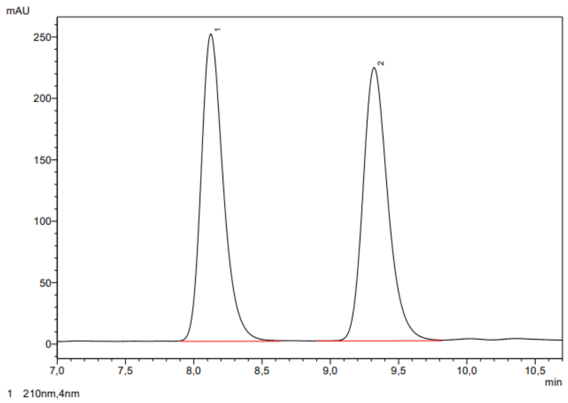

PDA Ch1 210nm

Peak #	Ret. Time	Area %	Name
1	8,12	49,97	1. Enantiomer
2	9,32	50,03	2. Enantiomer
Total		100,00	

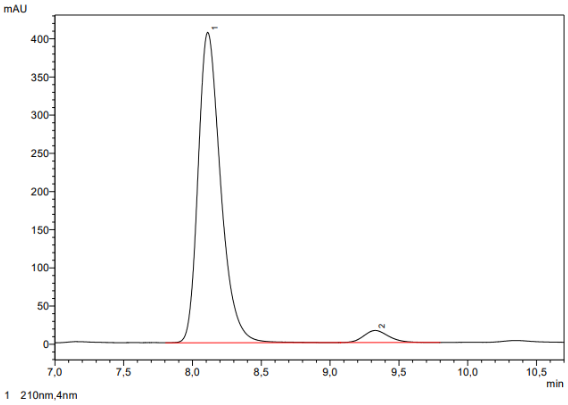

PDA Ch1 210nm

Peak #	Ret. Time	Area %	Name
1	8,11	95,83	1. Enantiomer 91.7 % ee
2	9,33	4,17	2. Enantiomer
Total		100,00	

Benzyl (*R*,*E*)-6,6,6-trifluoro-5-((*R*)-hydroxy(phenyl)methyl)hex-2-enoate (18). The title compound was prepared from benzaldehyde (15.2 µL, 0.15 mmol) and benzyl 6,6,6-trifluorosorbate (53.8 mg, 0.21 mmol) according to the general procedure **A**. The crude material (rr ≈ 10:1) was purified by automated flash chromatography (10 g column, 5–60 % gradient of cyclohexane/ethyl acetate) to afford the product as a colorless oil (49.6 mg, 91 % yield, dr > 20:1, 63 % ee).

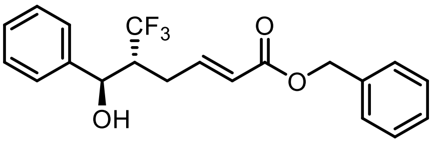

^{1}H NMR (400 MHz, CD_2Cl_2, 298 K): δ [ppm] = 7.42 – 7.25 (m, 11H), 6.82 – 6.68 (m, 1H), 5.78 (dt, *J* = 15.6, 1.5 Hz, 1H), 5.26 (d, *J* = 2.8 Hz, 1H), 5.10 (s, 2H), 2.69 – 2.52 (m, 3H). ^{13}C NMR (101 MHz, CD_2Cl_2, 298 K): δ [ppm] = 166.1, 146.2, 141.6, 136.7, 129.0, 128.9, 128.5, 128.5, 128.4, 126.1, 123.1, 70.5 (q, $^{3}J_{CF}$ = 3.0 Hz), 66.4, 50.3 (q, $^{2}J_{CF}$ = 23.4 Hz), 25.80 (q, $^{3}J_{CF}$ = 2.2 Hz). ^{19}F NMR (282 MHz, CD_2Cl_2, 298 K): δ [ppm] = -67.3 (s). HRMS (ESI^{+}) : *m/z*: calcd. for $[C_{20}H_{19}F_3O_3Na]^+$ $[M+Na]^+$: 387.1179, found: 387.1178.

The ee was determined by HPLC analysis: Chiralpak 150 mm IB-N-3, 3 µm, 4.6 mm ∅, acetonitrile/H_2O = 50:50, v = 1.0 mL/min, λ = 220 nm, t(minor) = 16.41 min, t(major) = 17.68 min (racemate: left, enantioenriched sample: right).

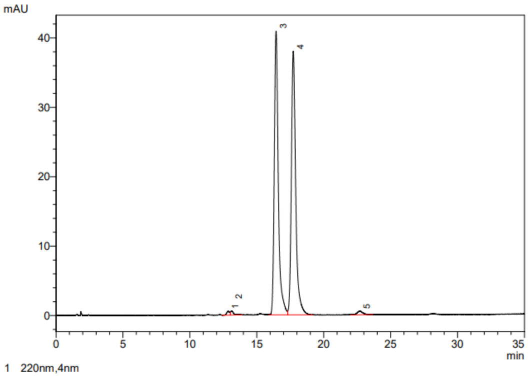

1 220nm,4nm

PDA Ch1 220nm

Peak #	Ret. Time	Area %	Name
1	12.86	0.50	
2	13.13	0.56	
3	16.44	48.95	1. enantiomer
4	17.73	49.03	2. enantiomer
5	22.69	0.95	
Total		100.00	

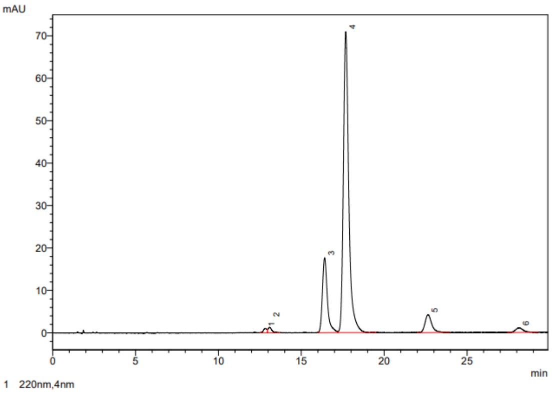

1 220nm,4nm

PDA Ch1 220nm

Peak #	Ret. Time	Area %	Name
1	12.81	0.67	
2	13.09	0.94	
3	16.41	17.00	1. enantiomer
4	17.68	74.24	2. enantiomer
5	22.65	5.52	
6	28.15	1.64	
Total		100.00	

ee = 62.7 %

Difluoromethyl Derivatives

(5*R*,6*S*,*E*)-5-(Difluoromethyl)-6-hydroxy-*N*-methoxy-*N*-methyloct-2-enamide (19a). The title compound was prepared from propionaldehyde (14.4 µL, 0.20 mmol) and diene **15** (53.5 mg, 0.28 mmol,) according to the general procedure **B**. The crude material (rr ≈ 12:1) was purified by automated flash chromatography (10 g column, 5–60 % gradient of cyclohexane/ethyl acetate) to afford the product as a colorless oil (39.7 mg, 79 % yield, dr > 20:1, 96 % ee).

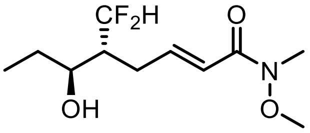

$[\alpha]_D^{20}$ = –2.9 (c = 0.8, CH_2Cl_2). ^1H NMR (400 MHz, CD_2Cl_2, 298 K): δ [ppm] = 6.99 – 6.85 (m, 1H), 6.54 – 6.42 (m, 1H), 5.92 (td, *J* = 56.4, 4.3 Hz, 1H), 3.86 – 3.75 (m, 1H), 3.68 (s, 3H), 3.19 (s, 3H), 2.53 – 2.46 (m, 2H), 2.35 (br. s, 1H), 2.17 – 1.98 (m, 1H), 1.59 – 1.47 (m, 2H), 0.96 (t, *J* = 7.4 Hz, 3H). ^{13}C NMR (101 MHz, CD_2Cl_2, 298 K): δ [ppm] = 166.7, 145.1, 121.0, 118.5 (t, $^1J_{CF}$ = 242.0 Hz), 71.3 (t, $^3J_{CF}$ = 5.0 Hz), 62.1, 47.4 (t, $^2J_{CF}$ = 17.8 Hz), 32.5, 27.8, 26.7 (t, $^3J_{CF}$ = 4.7 Hz), 10.6. ^{19}F NMR (282 MHz, CD_2Cl_2, 298 K): δ [ppm] = –121.5 (d, *J* = 282.3 Hz), –123.6 (d, *J* = 282.3 Hz). IR (ATR): ṽ = 3114, 2967, 2939, 1660, 1617, 1462, 1427, 1388, 1260, 1100, 1032, 915, 800, 542, 460 cm^{-1}. HRMS (ESI^+): *m/z*: calcd. for $[C_{11}H_{19}F_2NO_3Na]^+$ $[M+Na]^+$: 274.1225, found: 274.1223.

The ee was determined by HPLC analysis: Chiralpak 150 mm IG-3, 3 µm, 4.6 mm ∅, CH_3OH/H_2O = 80:20, v = 1.0 mL/min, λ = 215 nm, t(minor) = 5.79 min, t(major) = 6.77 min (racemate: left, enantioenriched sample: right).

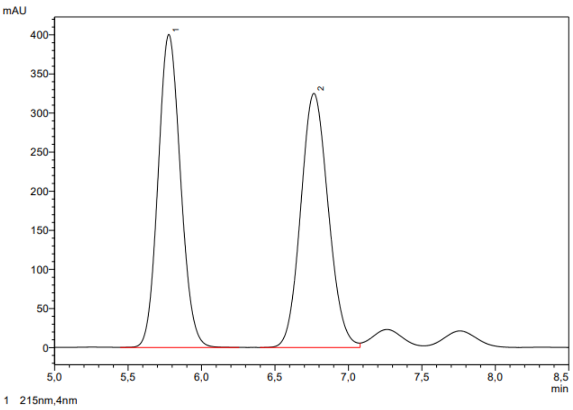

1 215nm,4nm

PDA Ch1 215nm

Peak #	Ret. Time	Area %	Name
1	5,78	50,08	1. Enantiomer
2	6,77	49,92	2. Enantiomer
Total		100,00	

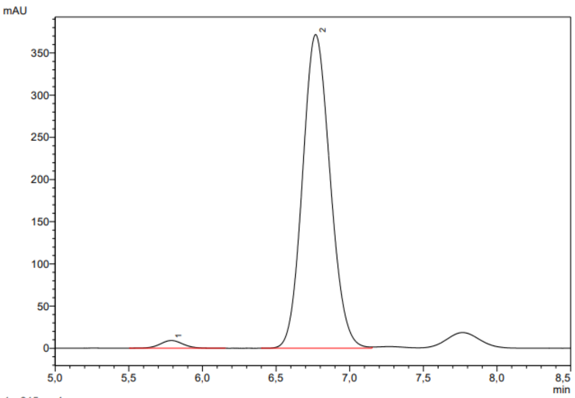

1 215nm,4nm

PDA Ch1 215nm

Peak #	Ret. Time	Area %	Name
1	5,79	2,00	1. Enantiomer
2	6,77	98,00	2. Enantiomer 96.0 % ee
Total		100,00	

(5*R*,6*S*,*E*)-5-(Difluoromethyl)-6-hydroxy-*N*-methoxy-*N*-methyldec-2-enamide (19b). The title compound was prepared from valeraldehyde (53.4 µL, 0.25 mmol) and diene **15** (133.8 mg, 0.7 mmol) according to the general procedure **B**. The crude material (rr ≈ 20:1) was purified by automated flash chromatography (10 g column, 5–60 % gradient of cyclohexane/ethyl acetate) to afford the product (111.1 mg, 80 % yield, dr > 20:1, 96 % ee) as a colorless oil.

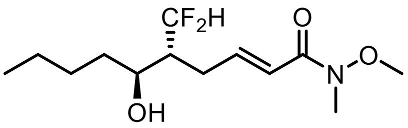

$[\alpha]_D^{20}$ = –8.7 (c = 1.3, CH_2Cl_2). ^{1}H NMR (400 MHz, $CDCl_3$, 298 K): δ [ppm] = 7.01 – 6.91 (m, 1H), 6.47 (d, *J* = 15.5 Hz, 1H), 5.92 (td, *J* = 56.3, 4.0 Hz, 1H), 3.97 – 3.88 (m, 1H), 3.69 (s, 3H), 3.23 (s, 3H), 2.53 – 2.47 (m, 2H), 2.13 – 1.98 (m, 2H), 1.58 – 1.19 (m, 6H), 0.91 (t, *J* = 7.0 Hz, 3H). ^{19}F NMR (282 MHz, $CDCl_3$, 298 K): δ [ppm] = –121.5 (d, *J* = 282.3 Hz), –123.6 (d, *J* = 282.3 Hz). ^{13}C NMR (101 MHz, $CDCl_3$, 298 K): δ [ppm] = 166.6, 144.9, 120.8, 117.9 (t, $^1J_{CF}$ = 241.9 Hz), 69.5 (t, $^3J_{CF}$ = 4.8 Hz), 61.9, 47.3 (t, $^2J_{CF}$ = 17.9 Hz), 34.2, 32.5, 28.3, 26.5 (t, $^3J_{CF}$ = 4.6 Hz), 22.6, 14.1. IR (ATR): ṽ = 3407, 2956, 2934, 2872, 1659, 1616, 1462, 1423, 1385, 1196, 1180, 1103, 1034, 997, 913, 688, 622, 529, 440 cm^{-1}. HRMS (ESI$^+$): *m/z*: calcd. for $[C_{13}H_{23}F_2NO_3Na]^+$ $[M+Na]^+$: 302.1538, found: 302.1535.

The ee was determined by HPLC analysis: Chiralpak 150 mm IG-3, 3 µm, 4.6 mm ∅, MeOH/H_2O = 80:20, v = 1.0 mL/min, λ = 215 nm, t(minor) = 6.53 min, t(major) = 8.79 min (racemate: left, enantioenriched sample: right).

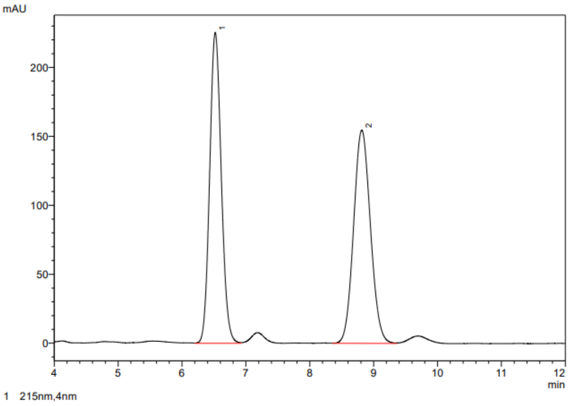

PDA Ch1 215nm

Peak #	Ret. Time	Area %	Name
1	6,52	50,06	1. Enantiomer
2	8,81	49,94	2. Enantiomer
Total		100,00	

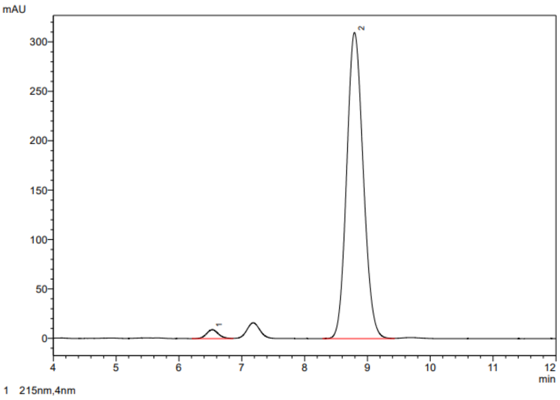

PDA Ch1 215nm

Peak #	Ret. Time	Area %	Name
1	6,53	2,08	1. Enantiomer
2	8,79	97,92	2. Enantiomer 95.8 % ee
Total		100,00	

(5*R*,6*S*,*E*)-5-(Difluoromethyl)-6-hydroxy-*N*-methoxy-*N*-methyl-8-phenyloct-2-enamide (19d).

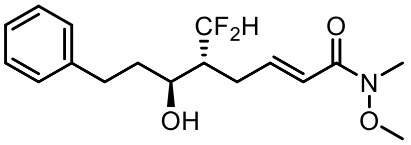

The title compound was prepared from 3-phenylpropanal (26.3 µL, 0.20 mmol) and diene **15** (53.5 mg, 0.28 mmol) according to the general procedure **B**. The crude material (rr ≈ 10:1) was purified by automated flash chromatography (10 g column, 5–60 % gradient of cyclohexane/ethyl acetate) to afford the product as a colorless oil (51.8 mg, 78 % yield, dr > 20:1, 96 % ee).

$[\alpha]_D^{20}$ = –11.1 (c = 0.9, CH_2Cl_2). ^{1}H NMR (400 MHz, CD_2Cl_2, 298 K): δ [ppm] = 7.32 – 7.15 (m, 5H), 6.97 – 6.85 (m, 1H), 6.52 – 6.43 (m, 1H), 5.94 (td, *J* = 56.3, 4.0 Hz, 1H), 3.99 – 3.89 (m, 1H), 3.66 (s, 3H), 3.19 (s, 3H), 2.89 – 2.76 (m, 1H), 2.70 – 2.57 (m, 1H), 2.55 – 2.46 (m, 2H), 2.34 (br. s, 1H), 2.20 – 2.02 (m, 1H), 1.88 – 1.75 (m, 2H). ^{13}C NMR (101 MHz, CD_2Cl_2, 298 K): δ [ppm] = 166.6, 144.8, 142.1, 128.8, 128.8, 126.3, 121.2, 118.4 (t, $^1J_{CF}$ = 241.7 Hz), 69.5 – 68.9 (m), 62.1, 47.9 (t, $^2J_{CF}$ = 17.8 Hz), 36.5, 32.7, 32.5, 26.8 (t, $^3J_{CF}$ =4.7 Hz). ^{19}F NMR (282 MHz, CD_2Cl_2, 298 K): δ [ppm] = –121.4 (d, *J* = 282.4 Hz), –123.5 (d, *J* = 282.6 Hz). IR (ATR): ṽ = 3406, 2936, 1659, 1613, 1425, 1386, 1178, 1133, 1092, 1030, 996, 913, 750, 701, 523, 444 cm^{-1}. HRMS (ESI$^+$): *m/z*: calcd. for $[C_{17}H_{23}F_2NO_3Na]^+$ $[M+Na]^+$: 350.1535, found: 350.1538.

The ee was determined by HPLC analysis: Chiralpak 150 mm IG-3, 3 µm, 4.6 mm ∅, *n*-heptane/ethanol = 92:8, v = 1.0 mL/min, λ = 210 nm, t(minor) = 21.38 min, t(major) = 22.81 min (racemate: left, enantioenriched sample: right).

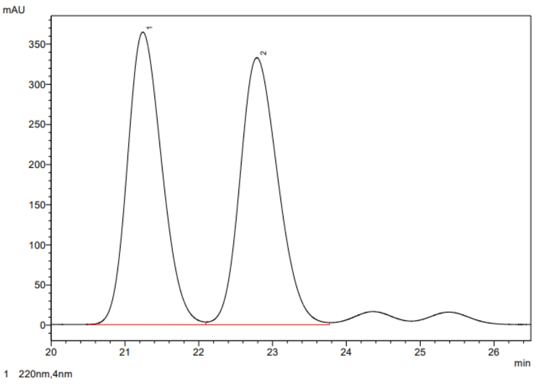

PDA Ch1 220nm

Peak #	Ret. Time	Area %	Name
1	21,24	49,88	1. Enantiomer
2	22,79	50,12	2. Enantiomer
Total		100,00	

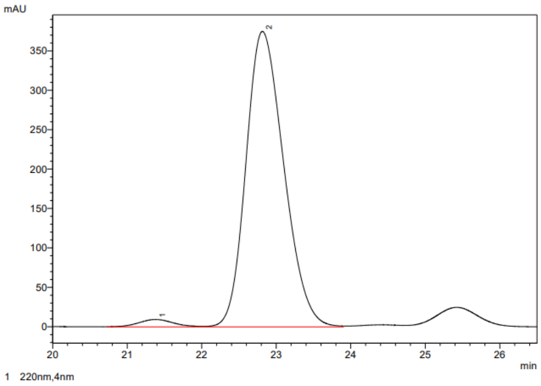

PDA Ch1 220nm

Peak #	Ret. Time	Area %	Name
1	21,38	2,22	1. Enantiomer
2	22,81	97,78	2. Enantiomer 95.6 % ee
Total		100,00	

(5*R*,6*S*,*E*)-5-(Difluoromethyl)-6-hydroxy-*N*-methoxy-*N*,7-dimethyloct-2-enamide (19e). The title compound was prepared from isobutyraldehyde (18.3 µL, 0.20 mmol) and diene **15** (53.5 mg, 0.28 mmol) according to the general procedure **B**. The crude material (rr > 20:1) was purified by automated flash chromatography (10 g column, 5–60 % gradient of cyclohexane/ethyl acetate) to afford the product as a colorless oil (47.8 mg, 90 % yield, dr > 20:1, 92 % ee).

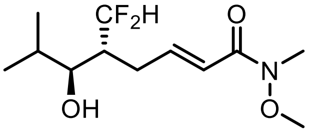

$[\alpha]_D^{20}$ = –5.2 (c = 1.3, CH_2Cl_2). ^{1}H NMR (400 MHz, CD_2Cl_2, 298 K): δ [ppm] = 6.99 – 6.90 (m, 1H), 6.48 (d, *J* = 15.3 Hz, 1H), 5.89 (td, *J* = 56.6, 5.1 Hz, 1H), 3.68 (s, 3H), 3.53 (dd, *J* = 8.2, 3.1 Hz, 1H), 3.19 (s, 3H), 2.59 – 2.41 (m, 2H), 2.34 – 2.10 (m, 2H), 1.85 – 1.72 (m, 1H), 0.99 (d, *J* = 6.6 Hz, 3H), 0.89 (d, *J* = 6.7 Hz, 3H). ^{13}C NMR (101 MHz, CD_2Cl_2, 298 K): δ [ppm] = 166.7, 145.3, 120.9, 119.1 (t, $^1J_{CF}$ = 241.7 Hz), 75.0 (t, $^3J_{CF}$ = 5.0 Hz), 62.1, 44.9 (t, $^2J_{CF}$ = 17.7 Hz), 32.5, 31.6, 26.8 – 26.6 (m), 19.3, 18.7. ^{19}F NMR (282 MHz, CD_2Cl_2, 298 K): δ [ppm] = –121.6 (d, *J* = 281.2 Hz), –123.8 (d, *J* = 281.1 Hz).
IR (ATR): ṽ = 3411, 2964, 2938, 2876, 1660, 1618, 1465, 1425, 1387, 1179, 1150, 1102, 1068, 1041, 998, 965, 969, 574, 443 cm^{-1}. HRMS (CI$^+$): *m/z*: calcd. for $[C_{12}H_{22}F_2NO_3]^+$ $[M+H]^+$: 266.1562, found: 266.1565.

The ee was determined by HPLC analysis: Chiralpak 150 mm IG-3, 3 µm, 4.6 mm ∅, *n*-heptane/ethanol = 90:10, v = 1.0 mL/min, λ = 215 nm, t(minor) = 5.79 min, t(major) = 6.77 min (racemate: left, enantioenriched sample: right).

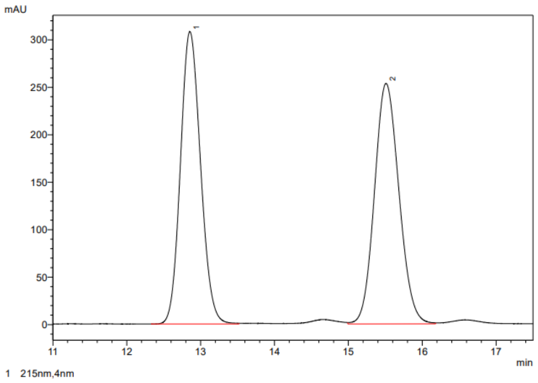

PDA Ch1 215nm

Peak #	Ret. Time	Area %	Name
1	12,85	49,90	1. Enantiomer
2	15,51	50,10	2. Enantiomer
Total		100,00	

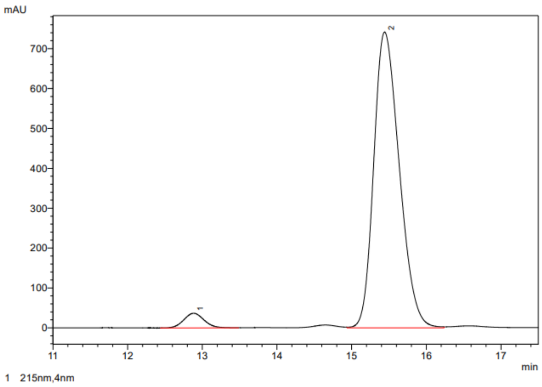

PDA Ch1 215nm

Peak #	Ret. Time	Area %	Name
1	12,88	3,81	1. Enantiomer
2	15,44	96,19	2. Enantiomer 92.4 % ee
Total		100,00	

(5*R*,6*S*,*E*)-6-Cyclobutyl-5-(difluoromethyl)-6-hydroxy-*N*-methoxy-*N*-methylhex-2-enamide

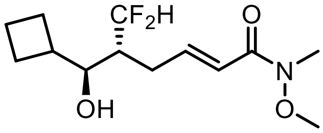

(19f). The title compound was prepared from cyclobutanecarbaldehyde (18.0 µL, 0.20 mmol) and diene **15** (53.5 mg, 0.28 mmol) according to the general procedure **B**. The crude material (rr ≈ 17:1) was purified by automated flash chromatography (10 g column, 5–60 % gradient of cyclohexane/ethyl acetate) to afford the product as a colorless oil (45.0 mg, 81 % yield, dr > 20:1, 91 % ee).

$[\alpha]_D^{20}$ = –2.8 (c = 1.1, CH_2Cl_2). ^1H NMR (400 MHz, CD_2Cl_2, 298 K): δ [ppm] = 6.95 – 6.85 (m, 1H), 6.49 – 6.42 (m, 1H), 5.87 (td, J = 56.5, 4.7 Hz, 1H), 3.86 – 3.80 (m, 1H), 3.68 (s, 3H), 3.19 (s, 3H), 2.54 – 2.42 (m, 3H), 2.30 (br. s, 1H), 2.09 – 1.84 (m, 5H), 1.82 – 1.69 (m, 2H). ^{13}C NMR (101 MHz, CD_2Cl_2, 298 K): δ [ppm] = 166.7, 145.3, 120.9, 118.6 (t, $^1J_{CF}$ = 241.8 Hz), 74.0 (t, $^3J_{CF}$ = 5.0 Hz), 62.1, 45.4 (t, $^2J_{CF}$ = 17.8 Hz), 39.1, 32.5, 26.6 (t, $^3J_{CF}$ = 4.7 Hz), 25.6, 24.8, 18.1. ^{19}F NMR (282 MHz, CD_2Cl_2, 298 K): δ [ppm] = –121.4 (d, J = 281.1 Hz), –123.8 (d, J = 281.2 Hz). IR (ATR): ṽ = 3411, 2967, 2938, 2864, 1659, 1617, 1462, 1424, 1387, 1245, 1179, 1094, 1037, 997, 916, 694, 620, 524, 441 cm^{-1}. HRMS (ESI$^+$): *m/z*: calcd. for $[C_{13}H_{21}F_2NO_3Na]^+$ $[M+Na]^+$: 300.1381, found: 300.1377.

The ee was determined by HPLC analysis: Chiralpak 150 mm IG-3, 3 µm, 4.6 mm ∅, *n*-heptane/2-propanol = 95:5, v = 1.0 mL/min, λ = 220 nm, t(minor) = 22.49 min, t(major) = 27.43 min (racemate: left, enantioenriched sample: right).

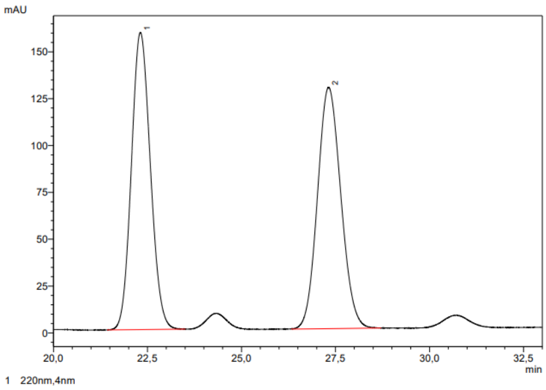

PDA Ch1 220nm

Peak #	Ret. Time	Area %	Name
1	22,31	50,07	1. Enantiomer
2	27,31	49,93	2. Enantiomer
Total		100,00	

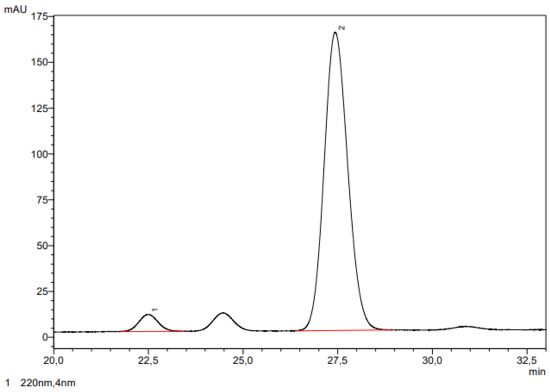

PDA Ch1 220nm

Peak #	Ret. Time	Area %	Name
1	22,49	4,45	1. Enantiomer
2	27,43	95,55	2. Enantiomer 91.1 % ee
Total		100,00	

(5*R*,6*S*,*E*)-6-Cyclohexyl-5-(difluoromethyl)-6-hydroxy-*N*-methoxy-*N*-methylhex-2-enamide

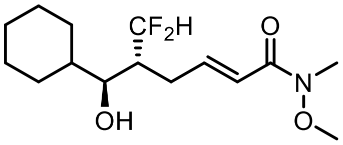

(19g). The title compound was prepared from cyclohexylcarbaldehyde (24.2 µL, 0.20 mmol) and diene **15** (53.5 mg, 0.28 mmol) according to the general procedure **A**. The crude material (rr > 20:1) was purified by automated flash chromatography (10 g column, 5–60 % gradient of cyclohexane/ethyl acetate) to afford the product as a colorless oil (48.8 mg, 79 % yield, dr > 20:1, 93 % ee).

$[\alpha]_D^{20}$ = –1.8 (c = 1.2, CH_2Cl_2). ^{1}H NMR (400 MHz, CD_2Cl_2, 298 K): δ [ppm] = 6.99 – 6.88 (m, 1H), 6.51 – 6.43 (m, 1H), 5.89 (td, *J* = 56.8, 5.3 Hz, 1H), 3.68 (s, 3H), 3.61 – 3.55 (m, 1H), 3.19 (s, 3H), 2.58 – 2.39 (m, 2H), 2.32 – 2.10 (m, 2H), 2.03 – 1.93 (m, 1H), 1.81 – 1.55 (m, 4H), 1.52 - 1.40 (m, 1H), 1.31 – 1.10 (m, 4H), 1.02 – 0.89 (m, 2H). ^{13}C NMR (101 MHz, CD_2Cl_2, 298 K): δ [ppm] = 166.7, 145.4, 120.8, 119.2 (t, $^1J_{CF}$ = 241.9 Hz), 74.2 – 73.7 (m), 62.1, 44.4 (t, $^2J_{CF}$ = 17.6 Hz), 41.0, 32.5, 29.5, 29.4, 26.7, 26.7, 26.3, 26.2. ^{19}F NMR (282 MHz, CD_2Cl_2, 298 K): δ [ppm] = –121.3 (d, *J* = 280.3 Hz), –123.8 (d, *J* = 280.6 Hz). IR (ATR): ṽ = 3416, 2925, 2852, 1660, 1618, 1447, 1387, 1311, 1181, 1095, 1035, 996, 968, 892, 694, 620, 536, 435 cm^{-1}. HRMS (EI): *m/z*: calcd. for $[C_{15}H_{25}F_2NO_3]^+$ $[M]^+$: 305.1797, found: 305.1798.

The ee was determined by HPLC analysis: Chiralpak 150 mm IG-3, 3 µm, 4.6 mm ∅, CH_3CN/H_2O = 40:60, v = 1.0 mL/min, λ = 215 nm, t(minor) = 22.82 min, t(major) = 32.70 min (racemate: left, enantioenriched sample: right).

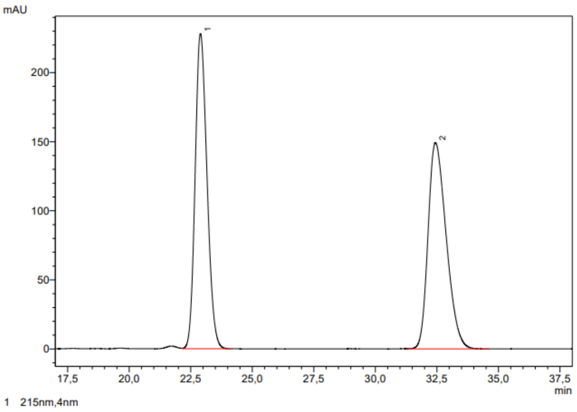

PDA Ch1 215nm

Peak #	Ret. Time	Area %	Name
1	22,90	49,95	1. Enantiomer
2	32,44	50,05	2. Enantiomer
Total		100,00	

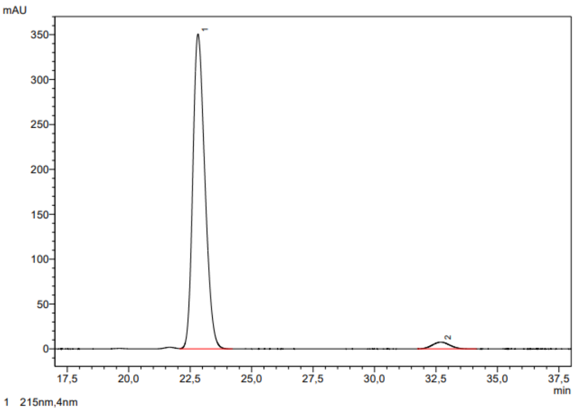

PDA Ch1 215nm

Peak #	Ret. Time	Area %	Name
1	22,82	96,93	1. Enantiomer 93.9 % ee
2	32,70	3,07	2. Enantiomer
Total		100,00	

tert-Butyl 4-((1S,2R,E)-2-(difluoromethyl)-1-hydroxy-6-(methoxy(methyl)amino)-6-oxohex-4-en-1-yl)piperidine-1-carboxylate (19h).

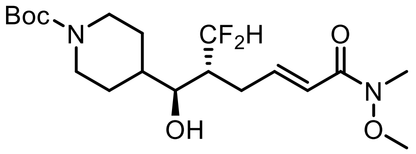

The title compound was prepared from 1-*tert*-butoxycarbonyl-4-piperidinecarboxaldehyde (42.7 mg, 0.20 mmol) and diene **15** (53.5 mg, 0.28 mmol) according to the general procedure **A**. The crude material (rr ≈ 20:1) was purified by automated flash chromatography (10 g column, 5–60 % gradient of cyclohexane/ethyl acetate) to afford the product as a colorless oil (40.6 mg, 50 % yield, dr > 20:1, 94 % ee).

$[\alpha]_D^{20}$ = +4.0 (c = 1.2, CH_2Cl_2). ^1H NMR (400 MHz, CD_2Cl_2, 298 K): δ [ppm] = 6.99 – 6.87 (m, 1H), 6.48 (d, *J* = 15.3 Hz, 1H), 5.90 (td, *J* = 56.6, 5.1 Hz, 1H), 4.16 – 4.08 (m, 2H), 3.68 (s, 3H), 3.68 – 3.59 (m, 1H), 3.19 (s, 3H), 2.78 – 2.42 (m, 5H), 2.23 – 2.09 (m, 1H), 1.97 – 1.89 (m, 1H), 1.68 – 1.56 (m, 1H), 1.55 – 1.46 (m, 1H), 1.42 (s, 9H), 1.18 – 1.10 (m, 2H). ^{13}C NMR (101 MHz, CD_2Cl_2, 298 K): δ [ppm] = 166.7, 155.0, 145.0, 121.1, 119.0 (t, $^1J_{CF}$ = 241.9 Hz), 79.5, 73.1 (t, $^3J_{CF}$ = 5.0 Hz), 62.1, 44.4 (t, $^2J_{CF}$ = 17.8 Hz), 44.0 (br. s), 39.6, 32.5, 28.7, 28.5, 26.7 (t, $^3J_{CF}$ = 4.6 Hz). ^{19}F NMR (282 MHz, CD_2Cl_2, 298 K): δ [ppm] = –121.9 (d, *J* = 281.2 Hz), –123.6 (d, *J* = 281.2 Hz). IR (ATR): ṽ = 3414, 2975, 2933, 1688, 1664, 1630, 1426, 1390, 1366, 1283, 1252, 1224, 1171, 1127, 1102, 1038, 998, 969, 870, 538 cm^{-1}. HRMS (ESI$^+$): *m/z*: calcd. for $[C_{19}H_{32}F_2N_2O_5Na]^+$ $[M+Na]^+$: 429.2172, found: 429.2171.

The ee was determined by HPLC analysis: Chiralpak 150 mm IB-N-3, 3 µm, 4.6 mm ∅, *n*-heptane/2-propanol = 90:10, v = 1.0 mL/min, λ = 215 nm, t(minor) = 10.98 min, t(major) = 9.53 min (racemate: left, enantioenriched sample: right).

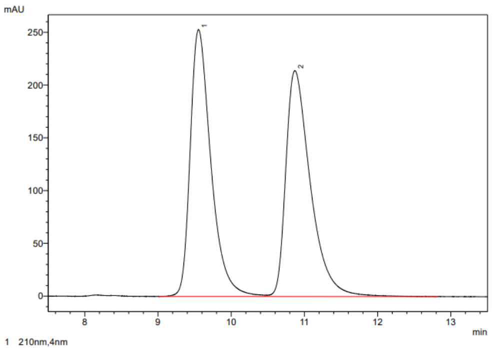

PDA Ch1 210nm

Peak #	Ret. Time	Area %	Name
1	9,55	49,88	1. Enantiomer
2	10,87	50,12	2. Enantiomer
Total		100,00	

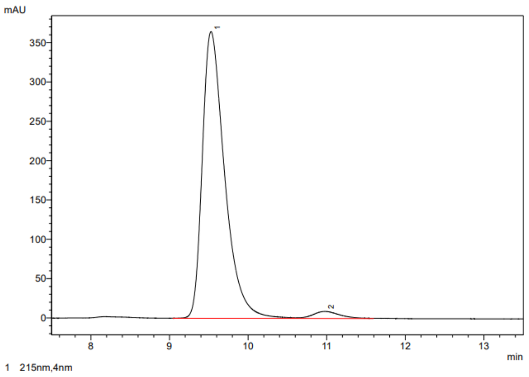

PDA Ch1 215nm

Peak #	Ret. Time	Area %	Name
1	9,53	96,97	1. Enantiomer 93.9 % ee
2	10,98	3,03	2. Enantiomer
Total		100,00	

(5*R*,6*S*,*E*)-5-(Difluoromethyl)-6-hydroxy-*N*-methoxy-*N*-methyl-6-(tetrahydro-2*H*-pyran-4-yl)hex-2-enamide (19i).

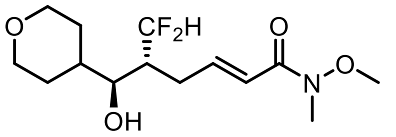

The title compound was prepared from tetrahydropyran-4-carbaldehyde (21.1 µL, 0.20 mmol) and diene **15** (53.5 mg, 0.28 mmol) according to the general procedure **A**. The crude material (rr > 20:1) was purified by automated flash chromatography (10 g column, 5–60 % gradient of cyclohexane/ethyl acetate) to afford the product as a colorless oil (44.3 mg, 72 % yield, dr > 20:1, 96 % ee).

$[\alpha]_D^{20}$ = –12.2 (c = 0.8, CH_2Cl_2). ^{1}H NMR (400 MHz, CD_2Cl_2, 298 K): δ [ppm] = 6.99 – 6.87 (m, 1H), 6.48 (d, *J* = 15.3 Hz, 1H), 5.90 (td, *J* = 56.6, 5.2 Hz, 1H), 4.01 – 3.87 (m, 2H), 3.68 (s, 3H), 3.65 – 3.59 (m, 1H), 3.38 – 3.28 (m, 2H), 3.19 (s, 3H), 2.80 (d, *J* = 5.3 Hz, 1H), 2.59 – 2.42 (m, 2H), 2.20 – 2.09 (m, 1H), 1.88 – 1.80 (m, 1H), 1.78 – 1.65 (m, 1H), 1.46 – 1.38 (m, 1H), 1.37 – 1.25 (m, 2H). ^{13}C NMR (101 MHz, CD_2Cl_2, 298 K): δ [ppm] = 166.7, 145.1, 121.0, 119.0 (t, $^1J_{CF}$ = 241.9 Hz), 73.4 – 73.2 (m), 68.1, 67.5, 62.1, 44.1 (t, $^2J_{CF}$ = 17.7 Hz), 38.6, 32.5, 29.7, 29.5, 26.6 (t, $^3J_{CF}$ = 4.6 Hz). ^{19}F NMR (282 MHz, CD_2Cl_2, 298 K): δ [ppm] = –121.5 (d, *J* = 281.2 Hz), –123.7 (d, *J* = 281.2 Hz). IR (ATR): ṽ = 3417, 2941, 2848, 1660, 1617, 1442, 1388, 1307, 1242, 1179, 1150, 1092, 1037, 986, 877, 823, 527 cm^{-1}. HRMS (ESI$^+$): *m/z*: calcd. for $[C_{14}H_{23}F_2NO_4Na]^+$ $[M+Na]^+$: 330.1487, found: 330.1488.

The ee was determined by HPLC analysis: Chiralpak 150 mm IG-3, 3 µm, 4.6 mm ∅, CH_3CN/H_2O = 40:60, v = 1.0 mL/min, λ = 215 nm, t(minor) = 3.85 min, t(major) = 5.45 min (racemate: left, enantioenriched sample: right).

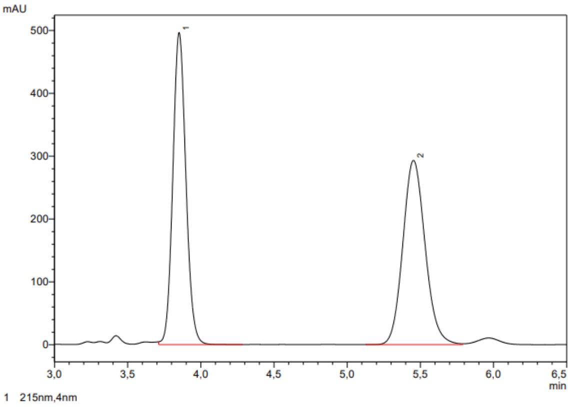

PDA Ch1 215nm

Peak #	Ret. Time	Area %	Name
1	3,85	49,74	1. Enantiomer
2	5,45	50,26	2. Enantiomer
Total		100,00	

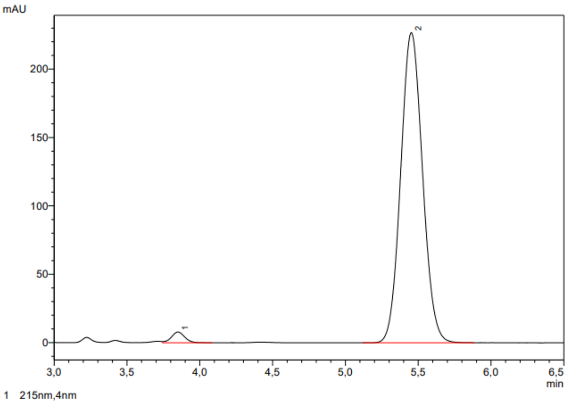

PDA Ch1 215nm

Peak #	Ret. Time	Area %	Name
1	3,85	2,04	1. Enantiomer
2	5,45	97,96	2. Enantiomer 95.9 % ee
Total		100,00	

(5*R*,6*S*,*E*)-5-(Difluoromethyl)-6-hydroxy-*N*-methoxy-*N*-methyl-8-(5-methylfuran-2-yl)oct-2-enamide (19j).

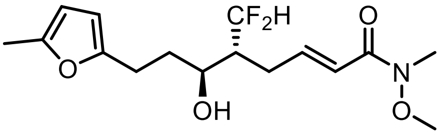

The title compound was prepared from 3-(5-methyl-2-furyl)-propionaldehyde (26.7 µL, 0.20 mmol) and diene **15** (53.5 mg, 0.28 mmol) according to the general procedure **B**. The crude material (rr ≈ 11:1) was purified by automated flash chromatography (10 g column, 5–60 % gradient of cyclohexane/ethyl acetate) to afford the product as a colorless oil (49.6 mg, 75 % yield, dr > 20:1, 96 % ee).

$[\alpha]_D^{20}$ = –8.0 (c = 0.7, CH_2Cl_2). ^1H NMR (400 MHz, CD_2Cl_2, 298 K): δ [ppm] = 6.95 – 6.86 (m, 1H), 6.52 – 6.44 (m, 1H), 6.11 – 5.77 (m, 3H), 3.97 – 3.90 (m, 1H), 3.67 (s, 3H), 3.19 (s, 3H), 2.81 – 2.71 (m, 1H), 2.68 – 2.58 (m, 1H), 2.54 – 2.47 (m, 2H), 2.39 (br. s, 1H), 2.23 (s, 3H), 2.17 – 2.01 (m, 1H), 1.85 – 1.77 (m, 2H). ^{13}C NMR (101 MHz, CD_2Cl_2, 298 K): δ [ppm] = 166.6, 153.7, 151.0, 144.8, 121.2, 118.3 (t, $^1J_{CF}$ = 241.4 Hz), 106.2, 69.1 (t, $^3J_{CF}$ = 4.8 Hz), 62.1, 47.9 (t, $^2J_{CF}$ = 17.9 Hz), 33.3, 32.5, 26.8 (t, $^3J_{CF}$ = 4.6 Hz), 25.1, 13.6. ^{19}F NMR (282 MHz, CD_2Cl_2, 298 K): δ [ppm] = –121.7 (d, *J* = 282.8 Hz), –123.5 (d, *J* = 282.8 Hz). IR (ATR): ṽ = 3410, 2925, 1659, 1615, 1571, 1437, 1386, 1218, 1179, 1091, 1021, 997, 960, 785, 693, 621, 577, 528, 446 cm^{-1}. HRMS (GC-EI): *m/z*: calcd. for $[C_{16}H_{23}F_2NO_4]$[M] : 331.1589, found: 331.1591.

The ee was determined by HPLC analysis: Chiralpak 150 mm IG-3, 3 µm, 4.6 mm ∅, *n*-heptane/2-propanol = 95:5, v = 1.0 mL/min, λ = 215 nm, t(minor) = 22.68 min, t(major) = 24.21 min (racemate: left, enantioenriched sample: right).

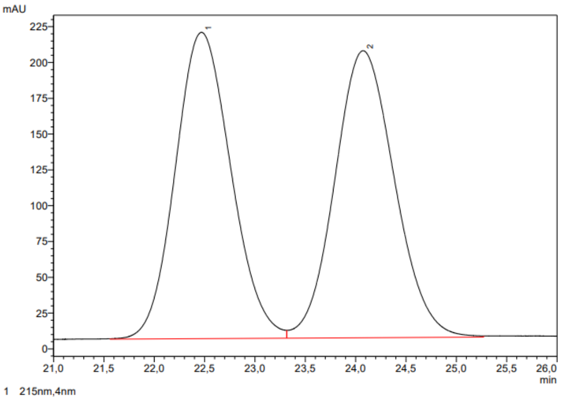

PDA Ch1 215nm

Peak #	Ret. Time	Area %	Name
1	22,47	49,85	1. Enantiomer
2	24,08	50,15	2. Enantiomer
Total		100,00	

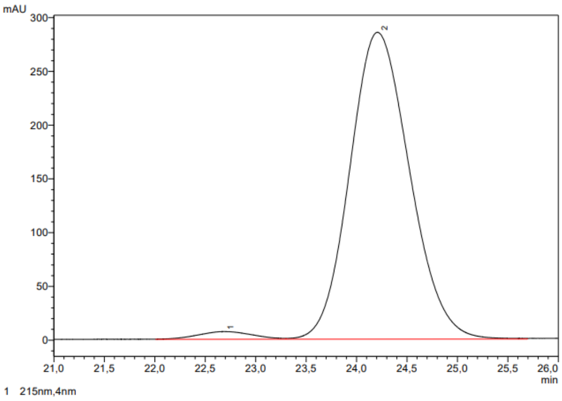

PDA Ch1 215nm

Peak #	Ret. Time	Area %	Name
1	22,68	2,22	1. Enantiomer
2	24,21	97,78	2. Enantiomer 95.6 % ee
Total		100,00	

Methyl (6*S*,7*R*,*E*)-7-(difluoromethyl)-6-hydroxy-11-(methoxy(methyl)amino)-11-oxoundec-9-enoate (19k). The title compound was prepared from methyl-6-oxohexanoate (27.5 µL, 0.20 mmol) and diene **15** (53.5 mg, 0.28 mmol) according to the general procedure **B**. The crude material (rr ≈ 17:1) was purified by automated flash chromatography (10 g column, 5–60 % gradient of cyclohexane/ethyl acetate) to afford the product as a colorless oil (51.9 mg, 77 % yield, dr > 20:1, 96 % ee).

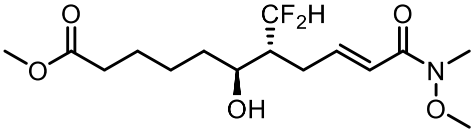

$[\alpha]_D^{20}$ = –8.7 (c = 1.1, CH_2Cl_2). ^{1}H NMR (400 MHz, CD_2Cl_2, 298 K): δ [ppm] = 6.96 – 6.86 (m, 1H), 6.51 - 6.43 (m, 1H), 5.92 (td, *J* = 56.4, 4.2 Hz, 1H), 3.93 – 3.86 (m, 1H), 3.68 (s, 3H), 3.63 (s, 3H), 3.19 (s, 3H), 2.52 – 2.46 (m, 2H), 2.31 (t, *J* = 7.4 Hz, 2H), 2.14 – 1.99 (m, 2H), 1.68 – 1.45 (m, 5H), 1.38 – 1.27 (m, 1H). ^{13}C NMR (101 MHz, CD_2Cl_2, 298 K): δ [ppm] = 174.3, 166.7, 145.0, 121.1, 118.4 (t, $^1J_{CF}$ = 241.6 Hz), 69.4 (t, $^3J_{CF}$ = 5.0 Hz), 62.1, 51.7, 47.8 (t, $^2J_{CF}$ = 17.8 Hz), 34.3, 34.2, 32.5, 26.8 (t, $^3J_{CF}$ = 4.7 Hz), 25.9, 25.0. ^{19}F NMR (282 MHz, CD_2Cl_2, 298 K): δ [ppm] = –121.5 (d, *J* = 282.3 Hz), –123.5 (d, *J* = 282.3 Hz). IR (ATR): ṽ = 3428, 2943, 2866, 1736, 1660, 1620, 1439, 1385, 1198, 1178, 1098, 1032, 997, 535 cm^{-1}. HRMS (ESI$^+$): *m/z*: calcd. for $[C_{15}H_{25}F_2NO_5Na]^+$ $[M+Na]^+$: 360.1593, found: 360.1590.

The ee was determined by HPLC analysis: Chiralpak 150 mm IG-3, 3 µm, 4.6 mm ∅, *n*-heptane/EtOH = 80:20, v = 1.0 mL/min, λ = 215 nm, t(minor) = 19.21 min, t(major) = 27.95 min (racemate: left, enantioenriched sample: right).

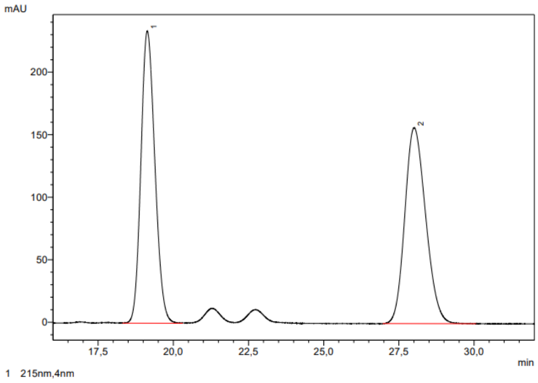

PDA Ch1 215nm

Peak #	Ret. Time	Area %	Name
1	19,13	49,98	1. Enantiomer
2	28,01	50,02	2. Enantiomer
Total		100,00	

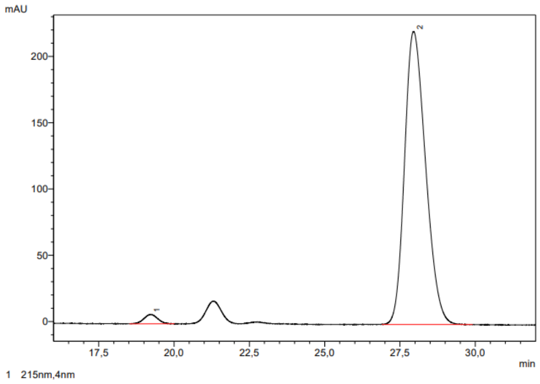

PDA Ch1 215nm

Peak #	Ret. Time	Area %	Name
1	19,21	2,04	1. Enantiomer
2	27,95	97,96	2. Enantiomer 95.9 % ee
Total		100,00	

(5R,6S,E)-10-((*tert*-Butyldimethylsilyl)oxy)-5-(difluoromethyl)-6-hydroxy-N-methoxy-N-methyldec-2-enamide (19l). The title compound was prepared from 5-((*tert*-butyldimethylsilyl)oxy)pentanal (71.3 µL, 0.20 mmol, 70 % purity) and diene **15** (53.5 mg, 0.28 mmol) according to the general procedure **B**. The crude material (rr > 20:1) was purified by automated flash chromatography (10 g column, 5–60 % gradient of cyclohexane/ethyl acetate) to afford the product as a colorless oil (55.8 mg, 68 % yield, dr > 20:1, 96 % ee).

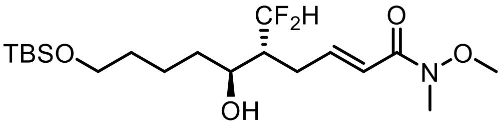

$[\alpha]_D^{20}$ = –4.1 (c = 0.9, CH_2Cl_2). ^{1}H NMR (400 MHz, CD_2Cl_2, 298 K): δ [ppm] = 6.98 – 6.85 (m, 1H), 6.48 (d, *J* = 15.3 Hz, 1H), 5.93 (td, *J* = 56.4, 4.2 Hz, 1H), 3.96 – 3.86 (m, 1H), 3.68 (s, 3H), 3.62 (t, *J* = 5.9 Hz, 2H), 3.19 (s, 3H), 2.54 – 2.44 (m, 2H), 2.23 – 1.93 (m, 2H), 1.60 – 1.45 (m, 5H), 1.41 – 1.32 (m, 1H), 0.89 (s, 9H), 0.04 (s, 6H). ^{13}C NMR (101 MHz, CD_2Cl_2, 298 K): δ [ppm] = 166.7, 145.0, 121.1, 118.5 (t, $^1J_{CF}$ = 241.5 Hz), 69.8 (t, $^3J_{CF}$ = 5.0 Hz), 63.4, 62.1, 47.8 (t, $^2J_{CF}$ = 17.8 Hz), 34.5, 32.8, 32.5, 26.8 (t, $^3J_{CF}$ = 4.6 Hz), 26.1, 22.8, 18.6, –5.2. ^{19}F NMR (282 MHz, CD_2Cl_2, 298 K): δ [ppm] = –121.6 (d, *J* = 282.3 Hz), –123.5 (d, *J* = 282.3 Hz). IR (ATR): ṽ = 3420, 2933, 2858, 1661, 1620, 1463, 1422, 1387, 1255, 1180, 1098, 1038, 1004, 836, 777, 662, 428 cm^{-1}. HRMS (ESI$^+$): *m/z*: calcd. for $[C_{19}H_{37}F_2NO_4SiNa]^+$ $[M+Na]^+$: 432.2352, found: 432.2354.

The ee was determined by HPLC analysis: Chiralpak 150 mm IG-3, 3 µm, 4.6 mm ∅, *n*-heptane/2-Propanol = 98:2, v = 1.0 mL/min, λ = 215 nm, t(minor) = 24.64 min, t(major) = 28.61 in (racemate: left, enantioenriched sample: right).

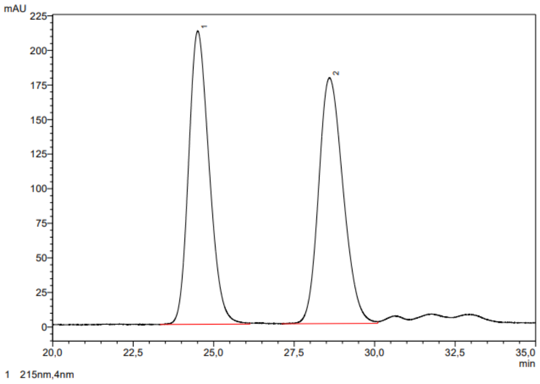

PDA Ch1 215nm

Peak #	Ret. Time	Area %	Name
1	24,51	49,96	1. Enantiomer
2	28,60	50,04	2. Enantiomer
Total		100,00	

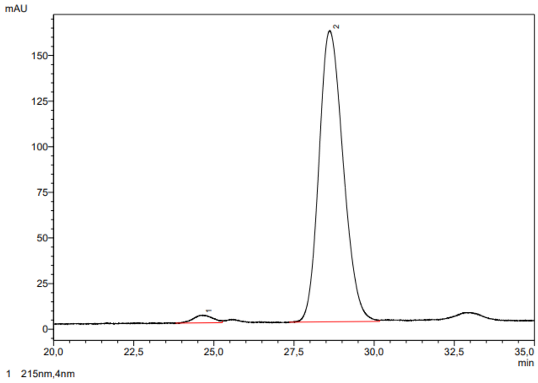

PDA Ch1 215nm

Peak #	Ret. Time	Area %	Name
1	24,64	2,14	1. Enantiomer
2	28,61	97,86	2. Enantiomer 95.7 % ee
Total		100,00	

(5*R*,6*S*,*E*)-10-Chloro-5-(difluoromethyl)-6-hydroxy-*N*-methoxy-*N*-methyldec-2-enamide (19m). The title compound was prepared from 5-chloropentenal (24.1 µL, 0.20 mmol) and diene **15** (53.5 mg, 0.28 mmol) according to the general procedure **B**. The crude material (rr ≈ 10:1) was purified by automated flash chromatography (10 g column, 5–60 % gradient of cyclohexane/ethyl acetate) to afford the product as a colorless oil (48.1 mg, 77 % yield, dr > 20:1, 95 % ee).

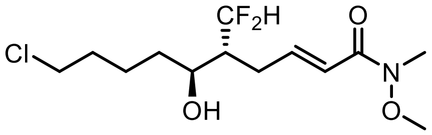

$[\alpha]_D^{20}$ = –8.9 (c = 1.2, CH_2Cl_2). ^{1}H NMR (400 MHz, CD_2Cl_2, 298 K): δ [ppm] = 6.96 – 6.86 (m, 1H), 6.52 – 6.45 (m, 1H), 5.93 (td, *J* = 56.3, 4.1 Hz, 1H), 3.93 – 3.87 (m, 1H), 3.68 (s, 3H), 3.55 (t, *J* = 6.6 Hz, 2H), 3.19 (s, 3H), 2.68 – 2.45 (m, 3H), 2.15 – 2.00 (m, 1H), 1.86 – 1.74 (m, 2H), 1.69 – 1.40 (m, 4H). ^{13}C NMR (101 MHz, CD_2Cl_2, 298 K): δ [ppm] = 166.7, 145.0, 121.1, 118.4 (t, $^1J_{CF}$ = 241.7 Hz), 69.5 (t, $^3J_{CF}$ = 4.8 Hz), 62.1, 47.8 (t, $^2J_{CF}$ = 17.8 Hz), 45.4, 33.9, 32.7, 32.5, 26.8 (t, $^3J_{CF}$ = 4.7 Hz), 23.8. ^{19}F NMR (282 MHz, CD_2Cl_2, 298 K): δ [ppm] = –121.6 (d, *J* = 282.8 Hz), –123.5 (d, *J* = 282.8 Hz). IR (ATR): ṽ = 3405, 2939, 1660, 1616, 1433, 1387, 1311, 1180, 1094, 1033, 998, 912, 692, 648, 528, 442 cm^{-1}. HRMS (ESI$^+$): *m/z*: calcd. for $[C_{13}H_{22}F_2NO_3Na]^+$ $[M+Na]^+$: 336.1148, found: 336.1146.

The ee was determined by HPLC analysis: Chiralpak 150 mm IG-3, 3 µm, 4.6 mm ∅, *n*-heptane/EtOH = 80:20, v = 1.0 mL/min, λ = 215 nm, t(minor) = 9.30 min, t(major) = 15.86 min (racemate: left, enantioenriched sample: right).

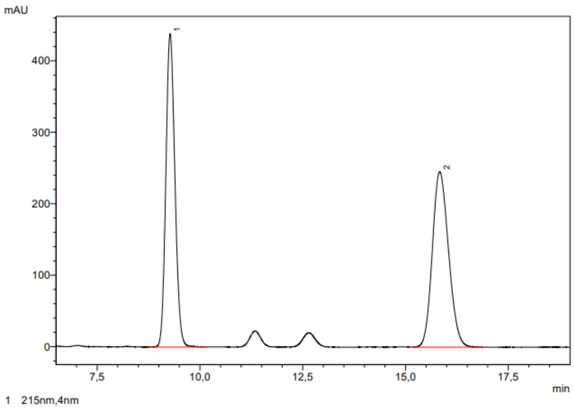

PDA Ch1 215nm

Peak #	Ret. Time	Area %	Name
1	9,27	49,87	1. Enantiomer
2	15,83	50,13	2. Enantiomer
Total		100,00	

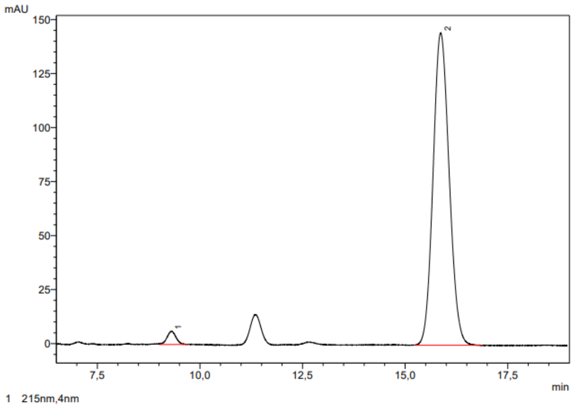

PDA Ch1 215nm

Peak #	Ret. Time	Area %	Name
1	9,30	2,32	1. Enantiomer
2	15,86	97,68	2. Enantiomer 95.4 % ee
Total		100,00	

(5*R*,6*R*,*E*)-7-(Benzyloxy)-5-(difluoromethyl)-6-hydroxy-*N*-methoxy-*N*-methylhept-2-enamide

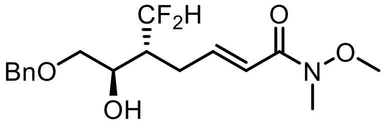

(19o). The title compound was prepared from benzyloxyacetaldehyde (28.1 µL, 0.20 mmol) and diene **15** (53.5 mg, 0.28 mmol) according to the general procedure **B**. The crude material (rr ≈ 3:1) was purified by automated flash chromatography (10 g column, 5–60 % gradient of cyclohexane/ethyl acetate) to afford the product as a colorless oil (37.0 mg, 54 % yield, dr > 20:1, 90 % ee).

$[\alpha]_D^{20}$ = –4.4 (c = 1.1, CH_2Cl_2). ^{1}H NMR (400 MHz, CD_2Cl_2, 298 K): δ [ppm] = 7.39 – 7.27 (m, 5H), 6.94 – 6.84 (m, 1H), 6.48 (d, *J* = 15.4 Hz, 1H), 5.93 (td, *J* = 56.2, 3.9 Hz, 1H), 4.55 (s, 2H), 4.11 – 4.02 (m, 1H), 3.67 (s, 3H), 3.59 – 3.47 (m, 2H), 3.19 (s, 3H), 2.63 – 2.55 (m, 1H), 2.55 – 2.49 (m, 2H), 2.29 – 2.15 (m, 1H). ^{13}C NMR (101 MHz, CD_2Cl_2, 298 K): δ [ppm] = 166.6, 144.3, 138.3, 128.81, 128.2, 128.2, 121.4, 117.8 (t, $^1J_{CF}$ = 241.9 Hz), 73.8, 72.4 – 72.3 (m), 68.9 – 68.2 (m), 62.1, 45.2 (t, $^2J_{CF}$ = 18.5 Hz), 32.5, 27.0 (t, $^3J_{CF}$ = 4.8 Hz). ^{19}F NMR (282 MHz, CD_2Cl_2, 298 K): δ [ppm] = –123.9 (d, *J* = 282.3 Hz), –123.2 (d, *J* = 282.3 Hz). IR (ATR): ṽ = 3410, 2935, 2868, 1660, 1619, 1454, 1386, 1200, 1179, 1100, 1027, 998, 910, 740, 699 cm^{-1}. HRMS (ESI$^+$): *m/z*: calcd. for $[C_{17}H_{23}F_2NO_4Na]^+$ $[M+Na]^+$: 366.1487, found: 366.1489.

The ee was determined by HPLC analysis: Chiralpak 150 mm IA-3, 3 µm, 4.6 mm ∅, *n*-heptane/ 2-propanol = 95:5, v = 1.0 mL/min, λ = 254 nm, t(minor) = 20.7 min, t(major) = 24.8 min (racemate: left, enantioenriched sample: right).

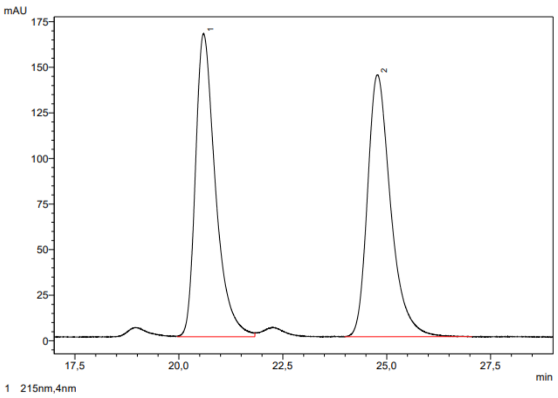

PDA Ch1 215nm

Peak #	Ret. Time	Area %	Name
1	20,60	49,98	1. Enantiomer
2	24,78	50,02	2. Enantiomer
Total		100,00	

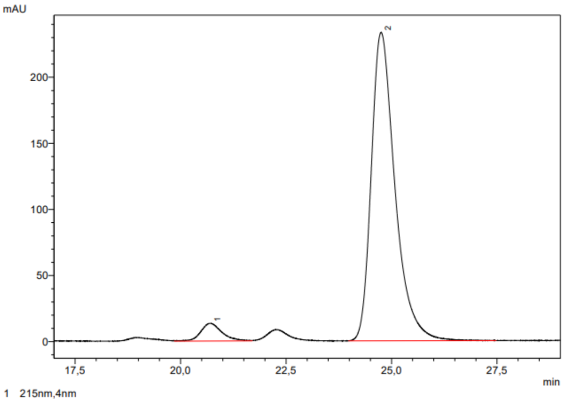

PDA Ch1 215nm

Peak #	Ret. Time	Area %	Name
1	20,71	4,89	1. Enantiomer
2	24,75	95,11	2. Enantiomer 90.2 % ee
Total		100,00	

(5*R*,6*S*,8*S*,*E*)-5-(Difluoromethyl)-6-hydroxy-*N*-methoxy-*N*,8,12-trimethyltrideca-2,11-dien-

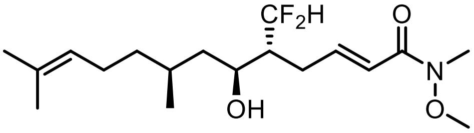

amide (19n). The title compound was prepared from (*R*)-(+)-citronellal (36.3 µL, 0.20 mmol) and diene **15** (53.5 mg, 0.28 mmol) according to the general procedure **B**. The crude material (rr > 20:1) was purified by automated flash chromatography (10 g column, 5–60 % gradient of cyclohexane/ethyl acetate) to afford the product as a colorless oil (53.1 mg, 76 % yield, dr > 20:1).

$[\alpha]_D^{20}$ = –13.8 (c = 0.8, CH_2Cl_2). ^{1}H NMR (400 MHz, CD_2Cl_2, 298 K): δ [ppm] = 6.96 – 6.86 (m, 1H), 6.52 – 6.44 (m, 1H), 5.94 (td, *J* = 56.3, 4.0 Hz, 1H), 5.13 – 5.07 (m, 1H), 4.05 – 3.95 (m, 1H), 3.68 (s, 3H), 3.19 (s, 3H), 2.53 – 2.45 (m, 2H), 2.28 (br. s, 1H), 2.12 – 1.91 (m, 3H), 1.70 – 1.66 (m, 3H), 1.63 – 1.56 (m, 4H), 1.37 – 1.13 (m, 4H), 0.90 (d, *J* = 6.6 Hz, 3H). ^{13}C NMR (101 MHz, CD_2Cl_2, 298 K): δ [ppm] = 166.3, 144.7, 131.2, 124.6, 120.6, 118.0 (t, $^1J_{CF}$ = 241.4 Hz), 67.2 – 66.8 (m), 61.7, 47.9 (t,

$^2J_{CF}$ = 17.8 Hz), 41.4, 37.8, 32.1, 28.9, 26.5 (t, $^3J_{CF}$ = 4.5 Hz), 25.4, 18.6, 17.3. ^{19}F NMR (282 MHz, CD_2Cl_2, 298 K): δ [ppm] = –121.3 (d, *J* = 282.7 Hz), –123.5 (d, *J* = 282.6 Hz). IR (ATR): ṽ = 3411, 2962, 2925, 1660, 1618, 1441, 1380, 1178, 1092, 1040, 1040, 998, 914, 841, 823, 694, 620, 531, 445 cm^{-1}. HRMS (ESI$^+$): *m/z*: calcd. for $[C_{18}H_{31}F_2NO_3Na]^+$ $[M+Na]^+$: 370.2164, found: 370.2162.

(5*R*,6*R*,*E*)-5-(Difluoromethyl)-6-((*S*)-2,2-dimethyl-1,3-dioxolan-4-yl)-6-hydroxy-*N*-methoxy-*N*-methylhex-2-enamide (19p).

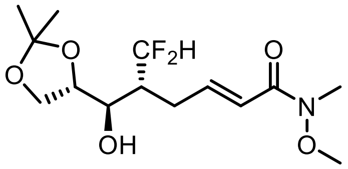

The title compound was prepared from (*S*)-2,2-dimethyl-1,3-dioxolan-4-carboxaldehyd (39.4 µL, 0.20 mmol) and diene **15** (53.5 mg, 0.28 mmol) according to the general procedure **B**. The crude material (rr ≈ 2:1) was purified by automated flash chromatography (10 g column, 5–60 % gradient of cyclohexane/ethyl acetate) to afford the product as a colorless oil (34.7 mg, 54 % yield, dr ≈ 10:1).

$[\alpha]_D^{20}$ = –1.8 (c = 1.1, CH_2Cl_2). ^{1}H NMR (400 MHz, CD_2Cl_2, 298 K): δ [ppm] = 6.95 – 6.85 (m, 1H), 6.54 – 6.46 (m, 1H), 5.92 (td, *J* = 56.2, 4.8 Hz, 1H), 4.21 – 4.15 (m, 1H), 4.05 – 3.99 (m, 1H), 3.78 – 3.70 (m, 2H), 3.68 (s, 3H), 3.19 (s, 3H), 2.69 – 2.44 (m, 3H), 2.14 – 2.01 (m, 1H), 1.42 (s, 3H), 1.35 (s, 3H). ^{13}C NMR (101 MHz, CD_2Cl_2, 298 K): δ [ppm] = 166.6, 144.3, 121.4, 117.9 (t, $^1J_{CF}$ = 242.3 Hz), 110.2, 77.0, 69.8 (t, $^3J_{CF}$ = 4.9 Hz), 66.5, 62.1, 46.2 (t, $^2J_{CF}$ = 18.3 Hz), 32.5, 27.3 (t, $^3J_{CF}$ = 4.7 Hz), 26.7, 25.4. ^{19}F NMR (282 MHz, CD_2Cl_2, 298 K): δ [ppm] = –121.5 (d, *J* = 282.3 Hz), –123.2 (d, *J* = 282.3 Hz). IR (ATR): ṽ = 2956, 2931, 2858, 1697, 1631, 1470, 1361, 1253, 1088, 1064, 979, 940, 897, 836, 807, 775 cm^{-1}. HRMS (ESI$^+$): *m/z*: calcd. for $[C_{14}H_{23}F_2NO_5Na]^+$ $[M+Na]^+$: 346.1436, found: 346.1435.

***tert*-Butyl ((2*R*,3*R*,4*R*,*E*)-4-(difluoromethyl)-3-hydroxy-8-(methoxy(methyl)amino)-8-oxooct-6-en-2-yl)carbamate (19q).**

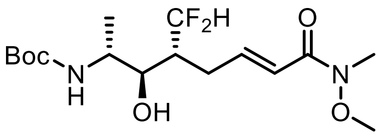

The title compound was prepared from *tert*-butyl (*R*)-(1-oxopropan-2-yl)carbamate (34.6 mg, 0.20 mmol) and diene **15** (53.5 mg, 0.28 mmol) according to the general procedure **B**. The crude material (rr ≈ 3:1) was purified by automated flash chromatography (10 g column, 5–60 % gradient of cyclohexane/ethyl acetate) to afford the product as a colorless oil (41.6 mg, 57 % yield, dr ≈ 4:1). The diastereomers were inseparable by flash chromatography but are clearly visible in obtained spectra.

$[\alpha]_D^{20}$ = +1.9 (c = 0.6, CH_2Cl_2). ^{1}H NMR (400 MHz, CD_2Cl_2, 298 K): δ [ppm] = 7.03 – 6.89 (m, 1H), 6.49 (d, *J* = 15.3 Hz, 1H), 6.20 – 5.67 (m, 1H), 5.09 – 4.87 (m, 1H), 3.84 – 3.71 (m, 2H), 3.68 (s, 3H), 3.67 – 3.61 (m, 1H), 3.20 (s, 3H), 2.70 – 2.46 (m, 2H), 2.27 – 2.09 (m, 1H), 1.42 (s, 9H), 1.21 – 1.12 (m, 3H). ^{13}C NMR (101 MHz, CD_2Cl_2, 298 K): δ [ppm] = 166.9, 156.8, 145.5, 120.8, 118.0 (t, $^1J_{CF}$ = 241.8 Hz), 80.0, 73.3, 62.1, 49.2, 45.7 (t, $^2J_{CF}$ = 17.8 Hz), 32.6, 28.5 (d, $^3J_{CF}$ = 2.9 Hz), 27.6 – 27.1 (m), 18.5. ^{19}F NMR (282 MHz, CD_2Cl_2, 298 K): δ [ppm] = –121.8 (d, *J* = 392.3 Hz), –124.9 (d, *J* = 283.9 Hz). IR (ATR): ṽ = 3369, 2976, 2935, 1703, 1660, 1620, 1511, 1453, 1391, 1367, 1247, 1167, 1104, 1047, 1027, 999 cm^{-1}. HRMS (ESI$^+$): *m/z*: calcd. for $[C_{16}H_{28}F_2N_2O_5Na]^+$ $[M+Na]^+$: 389.1859, found: 389.1855.

(5*R*,6*R*,*E*)-5-(Difluoromethyl)-6-hydroxy-*N*-methoxy-*N*-methyl-6-phenylhex-2-enamide

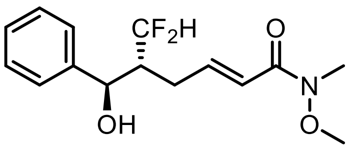

(20a). The title compound was prepared from benzaldehyde (20.2 μL, 0.20 mmol) and diene **15** (53.5 mg, 0.28 mmol) according to the general procedure **A**. The crude material (rr > 20:1) was purified by automated flash chromatography (10 g column, 5–60 % gradient of cyclohexane/ethyl acetate) to afford the product as a colorless oil (55.2 mg, 85 % yield, dr > 20:1, 91 % ee).

$[\alpha]_D^{20}$ = +28.2 (c = 0.7, CH_2Cl_2). ^{1}H NMR (400 MHz, CD_2Cl_2, 298 K): δ [ppm] = 7.42 – 7.25 (m, 5H), 6.90 – 6.77 (m, 1H), 6.36 (d, *J* = 15.3 Hz, 1H), 5.78 (td, *J* = 56.2, 4.2 Hz, 1H), 5.03 – 4.97 (m, 1H), 3.63 (s, 3H), 3.24 – 3.19 (m, 1H), 3.13 (s, 3H), 2.58 – 2.43 (m, 2H), 2.41 – 2.23 (m, 1H). ^{13}C NMR (101 MHz, CD_2Cl_2, 298 K): δ [ppm] = 166.7, 144.9, 141.7, 128.8, 128.1, 126.1, 120.6, 117.5 (t, $^1J_{CF}$ = 242.4 Hz), 72.0 – 71.5 (m) , 61.8, 49.4 (t, $^2J_{CF}$ = 18.2 Hz), 32.4, 26.5 (t, $^3J_{CF}$ = 4.1 Hz). ^{19}F NMR (282 MHz, CD_2Cl_2, 298 K): δ [ppm] = –120.9 (d, *J* = 283.4 Hz), –124.8 (d, *J* = 283.9 Hz). IR (ATR): ṽ = 3383, 2938, 1659, 1615, 1428, 1388, 1178, 1134, 1107, 1069, 1042, 998, 918, 823, 766, 704, 626, 538, 447 cm^{-1}. HRMS (ESI$^+$): *m/z*: calcd. for $[C_{15}H_{19}F_2NO_3Na]^+$ [M+Na]$^+$: 322.1222, found: 322.1222.

The ee was determined by HPLC analysis: Chiralpak 150 mm IG-3, 3 µm, 4.6 mm ∅, *n*- heptane/2-propanol = 90:10, v = 1.0 mL/min, λ = 210 nm, t(minor) = 12.07 min, t(major) = 16.49 min (racemate: left, enantioenriched sample: right).

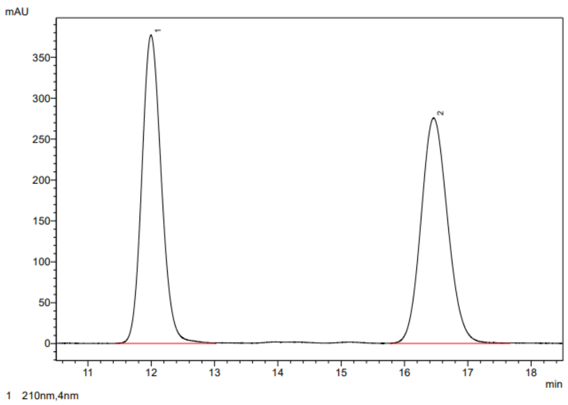

1 210nm,4nm

PDA Ch1 210nm

Peak #	Ret. Time	Area %	Name
1	12,00	49,99	1. Enantiomer
2	16,46	50,01	2. Enantiomer
Total		100,00	

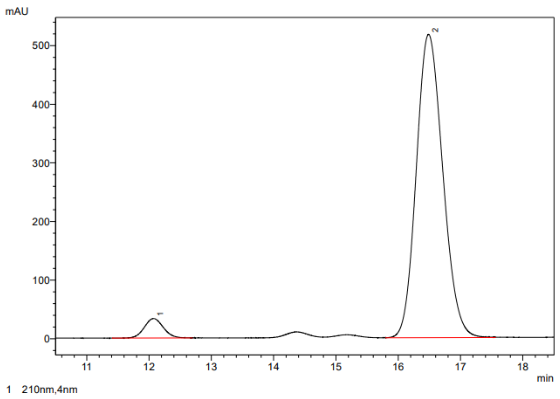

1 210nm,4nm

PDA Ch1 210nm

Peak #	Ret. Time	Area %	Name
1	12,07	4,42	1. Enantiomer
2	16,49	95,58	2. Enantiomer 91.2 % ee
Total		100,00	

(5*R*,6*R*,*E*)-5-(Difluoromethyl)-6-hydroxy-*N*-methoxy-*N*-methyl-6-(naphthalen-2-yl)hex-2-enamide (20b). The title compound was prepared from 2-naphthaldehyde (69.9 mg, 0.20 mmol) and diene **15** (53.5 mg, 0.28 mmol) according to the general procedure **A**. The crude material (rr > 20:1) was purified by automated flash chromatography (10 g column, 5–60 % gradient of cyclohexane/ethyl acetate) to afford the product as a colorless oil (61.5 mg, 88 % yield, dr > 20:1, 90 % ee).

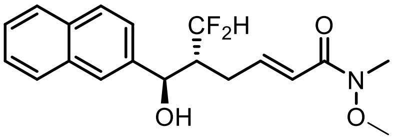

$[\alpha]_D^{20}$ = +10.7 (c = 0.9, CH_2Cl_2). ^1H NMR (400 MHz, CD_2Cl_2, 298 K): δ [ppm] = 7.89 – 7.81 (m, 4H), 7.53 – 7.42 (m, 3H), 6.92 – 6.80 (m, 1H), 6.33 (d, *J* = 15.4 Hz, 1H), 5.84 (td, *J* = 56.1, 4.2 Hz, 1H), 5.18 (dd, *J* = 5.2, 2.3 Hz, 1H), 3.55 (s, 3H), 3.16 – 3.11 (m, 1H), 3.09 (s, 3H), 2.60 – 2.37 (m, 3H). ^{13}C NMR (101 MHz, CD_2Cl_2, 298 K): δ [ppm] = 166.7, 145.0, 139.7, 133.6, 133.4, 128.7, 128.3, 128.0, 126.7, 126.5, 125.4, 124.3, 120.8, 118.3 (t, $^1J_{CF}$ = 242.1 Hz), 72.0 (t, $^3J_{CF}$ = 5.6 Hz), 61.9, 49.6 (t, $^2J_{CF}$ = 18.0 Hz), 32.4, 26.8 (t, $^3J_{CF}$ = 4.2 Hz). ^{19}F NMR (282 MHz, CD_2Cl_2, 298 K): δ [ppm] = –121.1 (d, *J* = 283.4 Hz), –124.6 (d, *J* = 283.4 Hz). IR (ATR): ṽ = 3371, 2937, 1658, 1613, 1508, 1426, 1386, 1306, 1271, 1177, 1125, 1040, 998, 961, 860, 822, 749, 665, 623, 479 cm^{-1}. HRMS (EI^+): *m/z*: calcd. for $[C_{19}H_{21}F_2NO_3]^+$ $[M]^+$: 349.1484, found: 349.1486.

The ee was determined by HPLC analysis: Chiralpak 150 mm IG-3, 3 µm, 4.6 mm ∅, CH_3CN/H_2O = 70:30, v = 1.0 mL/min, λ = 225 nm, t(minor) = 13.80 min, t(major) = 8.35 min (racemate: left, enantioenriched sample: right).

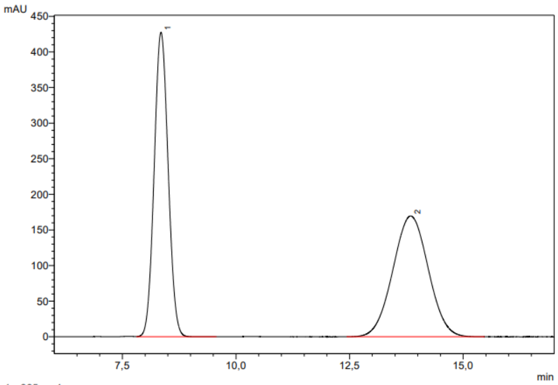

1 225nm,4nm

PDA Ch1 225nm

Peak #	Ret. Time	Area %	Name
1	8,35	49,84	1. Enantiomer
2	13,84	50,16	2. Enantiomer
Total		100,00	

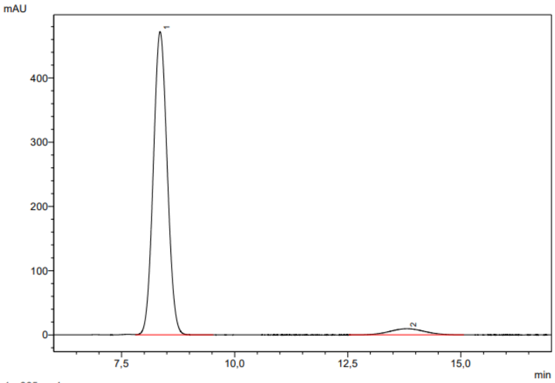

1 225nm,4nm

PDA Ch1 225nm

Peak #	Ret. Time	Area %	Name
1	8,35	95,11	1. Enantiomer 90.2 % ee
2	13,80	4,89	2. Enantiomer
Total		100,00	

(5*R*,6*R*,*E*)-5-(Difluoromethyl)-6-hydroxy-N-methoxy-N-methyl-6-(phenanthren-9-yl)hex-2-enamide (20c).

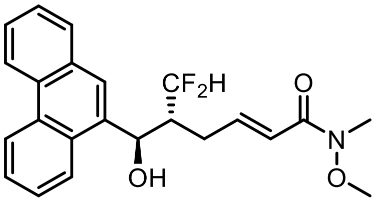

The title compound was prepared from phenanthrene-9-carbaldehyde (41.2 mg, 0.20 mmol) and diene **15** (53.5 mg, 0.28 mmol) according to the general procedure **A**. The crude material (rr > 20:1) was purified by automated flash chromatography (10 g column, 5–60 % gradient of cyclohexane/ethyl acetate) to afford the product (64.9 mg, 81 % yield, dr > 20:1, 80 % ee) as a colorless oil.

Note: due to high viscosity of the isolated compound, residual cyclohexane (≈ 5 %) is visible in the ^{1}H NMR spectrum. $[\alpha]_D^{20}$ = +36.8 (c = 0.8, CH_2Cl_2). ^{1}H NMR (400 MHz, CD_2Cl_2, 298 K): δ [ppm] = 8.79 – 8.72 (m, 1H), 8.66 (d, *J* = 7.4 Hz, 1H), 8.06 – 7.98 (m, 2H), 7.95 – 7.89 (m, 1H), 7.73 – 7.55 (m, 4H), 6.76 – 6.59 (m, 1H), 6.26 – 5.84 (m, 3H), 3.43 (s, 3H), 3.08 (s, 1H), 3.01 (s, 3H), 2.68 – 2.43 (m, 3H). ^{13}C NMR (101 MHz, CD_2Cl_2, 298 K): δ [ppm] = 166.5, 145.0, 135.7, 131.54, 131.1, 130.5, 129.2, 127.4, 127.3, 127.2, 126.9, 125.5, 123.9, 123.6, 122.8, 120.7, 119.0 (t, $^{1}J_{CF}$ = 242.2 Hz), 68.4 – 68.2 (m), 61.8, 47.5 (t, $^{2}J_{CF}$ = 17.9 Hz), 32.3, 26.6 (t, $^{3}J_{CF}$ = 4.3 Hz). ^{19}F NMR (282 MHz, CD_2Cl_2, 298 K): δ [ppm] = –121.7 (d, *J* = 281.7 Hz), –123.3 (d, *J* = 282.3 Hz). IR (ATR): ṽ = 3374, 2929, 2855, 1659, 1613, 1427, 1387, 1248, 1178, 1112, 1042, 996, 957, 892, 759, 752, 730, 703, 618 cm^{-1}. HRMS (ESI^{+}): *m/z*: calcd. for $[C_{23}H_{23}F_2NO_3Na]^+$ $[M+Na]^+$: 422.1538, found: 422.1541.

The ee was determined by HPLC analysis: Chiralpak 150 mm IB-N-3, 3 µm, 4.6 mm ∅, CH_3CN/H_2O = 60:40, v = 1.0 mL/min, λ = 254 nm, t(minor) = 8.28 min, t(major) = 16.04 min (racemate: left, enantioenriched sample: right).

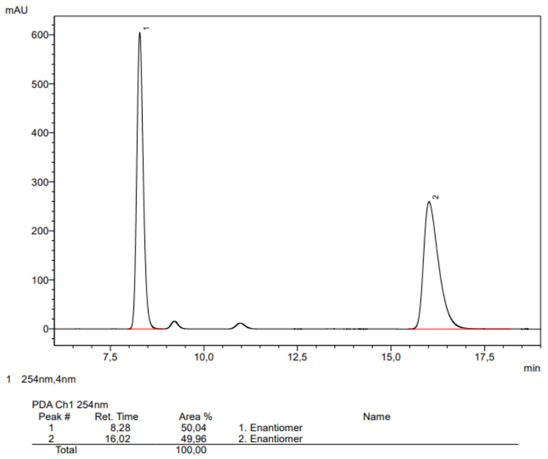

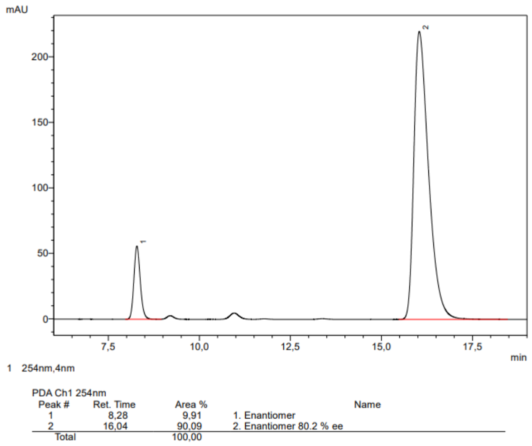

(5*R*,6*R*,*E*)-5-(Difluoromethyl)-6-hydroxy-*N*-methoxy-*N*-methyl-6-(o-tolyl)hex-2-enamide (20d). The title compound was prepared from 2-methylbenzaldehyde (23.1 µL, 0.20 mmol) and diene **15** (53.5 mg, 0.28 mmol) according to the general procedure **A**. The crude material (rr > 20:1) was purified by automated flash chromatography (10 g column, 5–60 % gradient of cyclohexane/ethyl acetate) to afford the product as a colorless oil. (41.3 mg, 66 % yield, dr > 20:1, 92 % ee)

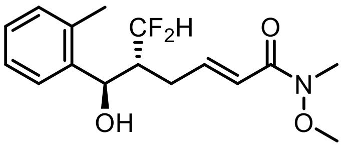

Note: due to high viscosity of the isolated compound, residual cyclohexane (≈ 5 %) is visible in the ^{1}H NMR spectrum. $[\alpha]_D^{20}$ = +42.2 (c = 0.9, CH_2Cl_2). ^{1}H NMR (400 MHz, CD_2Cl_2, 298 K): δ [ppm] = 7.56 – 7.50 (m, 1H), 7.27 – 7.12 (m, 3H), 6.85 – 6.73 (m, 1H), 6.34 (d, *J* = 15.4 Hz, 1H), 5.82 (td, *J* = 56.3, 4.7 Hz, 1H), 5.27 (d, *J* = 4.3 Hz, 1H), 3.63 (s, 3H), 3.13 (s, 3H), 2.87 (br. s, 1H), 2.57 – 2.51 (m, 2H), 2.32 – 2.19 (m, 4H). ^{13}C NMR (101 MHz, CD_2Cl_2, 298 K): δ [ppm] = 166.8, 145.3, 140.4, 134.7, 131.0, 128.0, 126.6, 126.5, 120.6, 118.6 (t, $^1J_{CF}$ = 241.9 Hz), 68.4 (t, $^3J_{CF}$ = 5.7 Hz), 62.0, 47.8 (t, $^2J_{CF}$ = 18.0 Hz), 32.5, 26.6 (t, $^3J_{CF}$ = 4.2 Hz), 19.0. ^{19}F NMR (282 MHz, CD_2Cl_2, 298 K): δ [ppm] = –121.8 (d, *J* = 282.8 Hz), –123.9 (d, *J* = 282.8 Hz). IR (ATR): ṽ = 3384, 2969, 2937, 1659, 1617, 1462, 1427, 1387, 1180, 1132, 1039, 998, 924, 888, 763, 733, 688, 625, 578, 439 cm^{-1}. HRMS (ESI$^+$): *m/z*: calcd. for $[C_{16}H_{21}F_2NO_3Na]^+$ $[M+Na]^+$: 336.1381, found: 336.1379.

The ee was determined by HPLC analysis: Chiralpak 150 mm IG-3, 3 µm, 4.6 mm ∅, CH_3CN/H_2O = 40:60, v = 1.0 mL/min, λ = 215 nm, t(minor) = 12.68 min, t(major) = 27.12 min (racemate: left, enantioenriched sample: right).

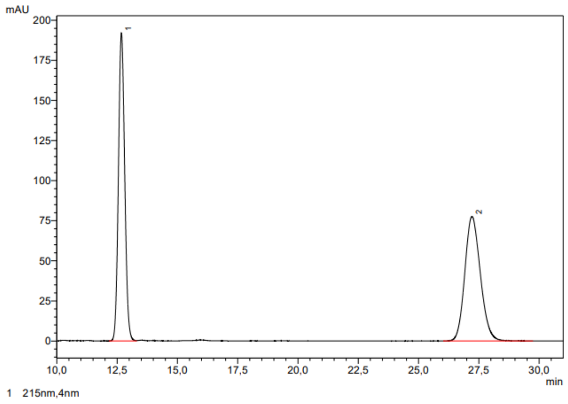

PDA Ch1 215nm

Peak #	Ret. Time	Area %	Name
1	12,68	50,02	1. Enantiomer
2	27,22	49,98	2. Enantiomer
Total		100,00	

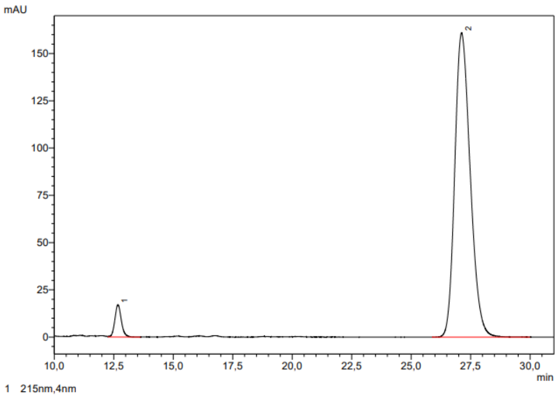

PDA Ch1 215nm

Peak #	Ret. Time	Area %	Name
1	12,68	4,26	1. Enantiomer
2	27,12	95,74	2. Enantiomer 91.5 % ee
Total		100,00	

(5*R*,6*R*,*E*)-5-(Difluoromethyl)-6-hydroxy-*N*-methoxy-6-(4-methoxyphenyl)-*N*-methylhex-2-enamide (20e).

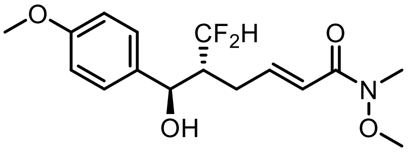

The title compound was prepared from 4-methoxybenzaldehyde (24.3 µL, 0.20 mmol) and diene **15** (53.5 mg, 0.28 mmol) according to the general procedure **A**. The crude material (rr > 20:1) was purified by automated flash chromatography (10 g column, 5–60 % gradient of cyclohexane/ethyl acetate) to afford the product as a white crystalline solid (55.1 mg, 84 % yield, dr > 20:1, 92 % ee).

m.p. = 81-82 °C. $[\alpha]_D^{20}$ = +23.9 (c = 0.6, CH_2Cl_2). ^{1}H NMR (400 MHz, CD_2Cl_2, 298 K): δ [ppm] = 7.34 – 7.29 (m, 2H), 6.96 – 6.85 (m, 3H), 6.42 (d, *J* = 15.4 Hz, 1H), 5.77 (td, *J* = 56.1, 4.0 Hz, 1H), 4.95 (d, *J* = 5.6 Hz, 1H), 3.83 (s, 3H), 3.69 (s, 3H), 3.19 (s, 3H), 2.86 (br. s, 1H), 2.60 – 2.51 (m, 2H), 2.41 – 2.26 (m, 1H). ^{13}C NMR (101 MHz, CD_2Cl_2, 298 K): δ [ppm] = 166.8, 159.7, 145.2, 134.2, 127.6, 120.7, 118.2 (t, $^1J_{CF}$ = 241.9 Hz), 114.3, 71.8 – 71.6 (m), 62.0, 55.6, 49.9 (t, $^2J_{CF}$ = 18.0 Hz), 32.5, 26.9 (t, $^3J_{CF}$ = 4.1 Hz). ^{19}F NMR (282 MHz, CD_2Cl_2, 298 K): δ [ppm] = –121.1 (d, *J* = 283.9 Hz), –125.0 (d, *J* = 282.7 Hz). IR (ATR): ṽ = 3391, 2938, 1659, 1612, 1512, 1441, 1387, 1330, 1176, 1134, 1093, 1033, 998, 923, 891, 834, 688, 541, 441 cm^{-1}. HRMS (ESI$^+$): *m/z*: calcd. for $[C_{16}H_{21}F_2NO_4Na]^+$ $[M+Na]^+$: 352.1331, found: 352.1332.

The ee was determined by HPLC analysis: Chiralpak 150 mm IG-3, 3 µm, 4.6 mm ∅, CH_3CN/H_2O = 70:30, v = 1.0 mL/min, λ = 225 nm, t(minor) = 5.25 min, t(major) = 9.17 min (racemate: left, enantioenriched sample: right).

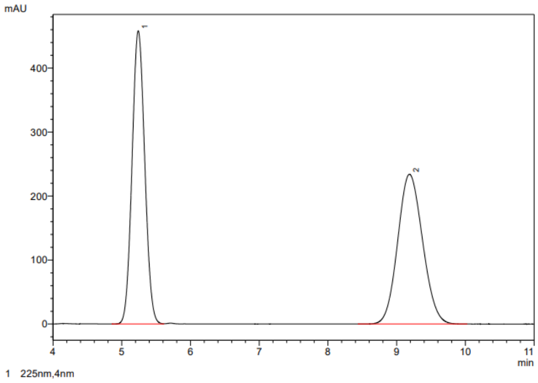

PDA Ch1 225nm

Peak #	Ret. Time	Area %	Name
1	5,24	49,96	1. Enantiomer
2	9,19	50,04	2. Enantiomer
Total		100,00	

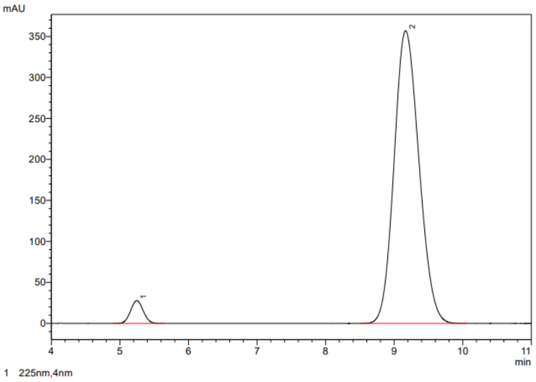

PDA Ch1 225nm

Peak #	Ret. Time	Area %	Name
1	5,25	3,80	1. Enantiomer
2	9,17	96,20	2. Enantiomer 92.4 % ee
Total		100,00	

(5*R*,6*R*,*E*)-6-(4-Chlorophenyl)-5-(difluoromethyl)-6-hydroxy-*N*-methoxy-*N*-methyl-hex-2-enamide (20f). The title compound was prepared from 4-chlorobenzaldehyde (23.5 µL, 0.20 mmol) and diene **15** (53.5 mg, 0.28 mmol) according to the general procedure **A**. The crude material (rr ≈ 14:1) was purified by automated flash chromatography (10 g column, 5–60 % gradient of cyclohexane/ethyl acetate) to afford the product as a colorless oil (43.9 mg, 66 % yield, dr > 20:1, 88 % ee).

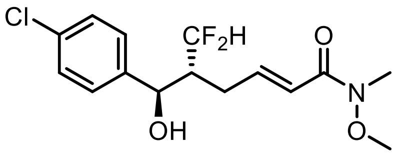

$[\alpha]_D^{20}$ = +7.0 (c = 1.2, CH_2Cl_2). ^{1}H NMR (400 MHz, CD_2Cl_2, 298 K): δ [ppm] = 7.37 – 7.29 (m, 4H), 6.86 – 6.77 (m, 1H), 6.34 (d, *J* = 15.3 Hz, 1H), 5.81 (td, *J* = 56.1, 4.3 Hz, 1H), 5.03 (d, *J* = 4.8 Hz, 1H), 3.64 (s, 3H), 3.14 (s, 3H), 3.08 (br. s, 1H), 2.55 – 2.39 (m, 2H), 2.37 – 2.22 (m, 1H). ^{13}C NMR (101 MHz, CD_2Cl_2, 298 K): δ [ppm] = 166.7, 144.7, 141.0, 133.7, 129.0, 127.9, 120.9, 118.1 (t, $^1J_{CF}$ = 242.2 Hz), 71.2 (t, $^3J_{CF}$ = 5.6 Hz), 62.1, 49.7 (t, $^2J_{CF}$ = 18.1 Hz), 32.5, 26.7 (t, $^3J_{CF}$ = 4.1 Hz). ^{19}F NMR (282 MHz, CD_2Cl_2, 298 K): δ [ppm] = –121.5 (d, *J* = 283.9 Hz), –124.5 (d, *J* = 283.9 Hz). IR (ATR): ṽ = 3379, 2968, 2938, 1659, 1614, 1490, 1426, 1389, 1180, 1118, 1090, 1042, 1014, 999, 923, 832, 680, 535 cm^{-1}. HRMS (EI$^+$): *m/z*: calcd. for $[C_{15}H_{18}F_2NO_3]^+$ $[M]^+$: 333.0937, found: 333.0936.

The ee was determined by HPLC analysis: Chiralpak 150 mm IG-3, 3 µm, 4.6 mm ∅, *n*-heptane/2-propanol = 95:5, v = 1.0 mL/min, λ = 220 nm, t(minor) = 25.59 min, t(major) = 27.92 min (racemate: left, enantioenriched sample: right).

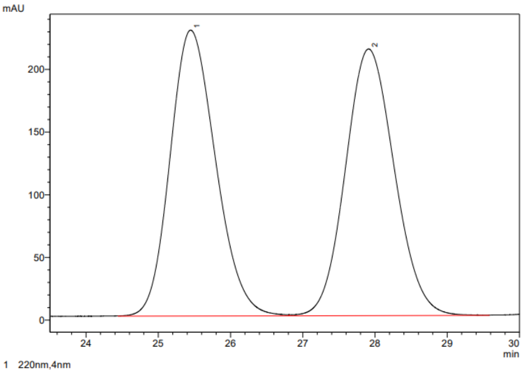

PDA Ch1 220nm

Peak #	Ret. Time	Area %	Name
1	25,45	49,95	1. Enantiomer
2	27,91	50,05	2. Enantiomer
Total		100,00	

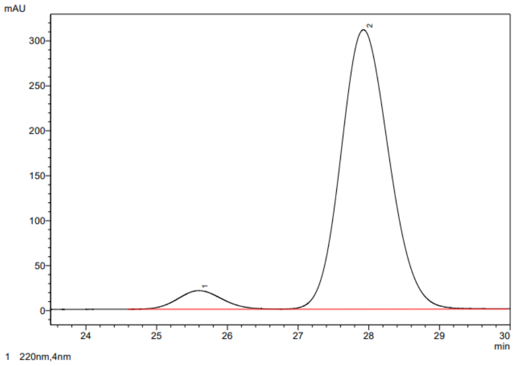

PDA Ch1 220nm

Peak #	Ret. Time	Area %	Name
1	25,59	5,83	1. Enantiomer
2	27,92	94,17	2. Enantiomer 88.3 % ee
Total		100,00	

(5*R*,6*R*,*E*)-5-(Difluoromethyl)-6-hydroxy-*N*-methoxy-*N*-methyl-6-(4-(trifluoromethyl)phenyl)-hex-2-enamide (20g). The title compound was prepared from 4-trifluoromethylbenzaldehyde (27.3 µL, 0.20 mmol) and diene **15** (53.5 mg, 0.28 mmol) according to the general procedure **A**. The crude material (rr > 20:1) was purified by automated flash chromatography (10 g column, 5–60 % gradient of cyclohexane/ethyl acetate) to afford the product as a colorless oil (51.5 mg, 70 % yield, dr > 20:1, 85 % ee).

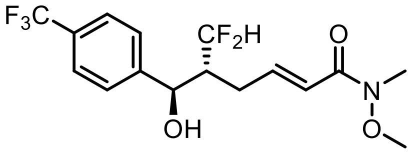

$[\alpha]_D^{20}$ = +19.6 (c = 0.7, CH_2Cl_2). ^{1}H NMR (400 MHz, CD_2Cl_2, 298 K): δ [ppm] = 7.64 (d, *J* = 8.1 Hz, 2H), 7.52 (d, *J* = 8.8 Hz, 2H), 6.87 – 6.75 (m, 1H), 6.32 (d, *J* = 15.3 Hz, 1H), 5.85 (td, *J* = 56.0, 4.4 Hz, 1H), 5.14 (d, *J* = 4.4 Hz, 1H), 3.62 (s, 3H), 3.35 (s, 1H), 3.11 (s, 3H), 2.53 – 2.28 (m, 3H). ^{13}C NMR (101 MHz, CD_2Cl_2, 298 K): δ [ppm] = 166.7, 146.8, 144.8, 130.0 (q, $^2J_{CF3}$ = 32.3 Hz), 126.9, 125.8 (q, $^3J_{CF3}$ = 3.8 Hz), 124.7 (q, $^1J_{CF3}$ = 271.5 Hz), 120.9, 118.1 (t, $^1J_{CF2H}$ = 242.2 Hz), 71.1 (t, $^3J_{CF2H}$ = 5.6 Hz), 62.0, 49.6 (t, $^2J_{CF2H}$ = 18.1 Hz), 32.4, 26.7 (t, $^3J_{CF2H}$ = 4.2 Hz). ^{19}F NMR (282 MHz, CD_2Cl_2, 298 K): δ [ppm] = –62.8 (s, 3F), –121.7 (d, *J* = 283.9 Hz), –124.3 (d, *J* = 283.9 Hz). IR (ATR): ṽ = 3377, 2940, 1660, 1618, 1419, 1390, 1326, 1165, 1124, 1067, 1017, 1000, 875, 690, 609, 519 cm^{-1}. HRMS (EI$^+$): *m/z*: calcd. for $[C_{16}H_{18}F_5NO_3]^+$ $[M]^+$: 367.1201, found: 367.1203.

The ee was determined by HPLC analysis: Chiralpak 150 mm IG-3, 3 µm, 4.6 mm ∅, CH_3CN/H_2O = 50:50, v = 1.0 mL/min, λ = 215 nm, t(minor) = 8.22 min, t(major) = 6.81 min (racemate: left, enantioenriched sample: right).

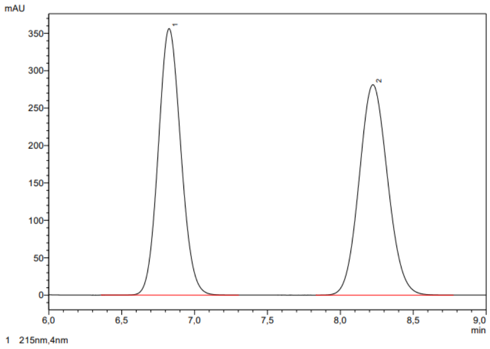

PDA Ch1 215nm

Peak #	Ret. Time	Area %	Name
1	6,83	50,02	1. Enantiomer
2	8,22	49,98	2. Enantiomer
Total		100,00	

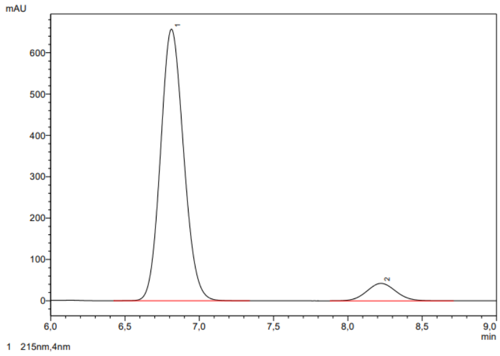

PDA Ch1 215nm

Peak #	Ret. Time	Area %	Name
1	6,81	92,51	1. Enantiomer 85.0 % ee
2	8,22	7,49	2. Enantiomer
Total		100,00	

Methyl 4-((1*R*,2*R*,*E*)-2-(difluoromethyl)-1-hydroxy-6 (methoxy(methyl)amino)-6-oxohex-4-en-1-yl)benzoate (20h). The title compound was prepared from methyl-4-formylbenzoate (32.8 mg, 0.20 mmol) and diene **15** (53.5 mg, 0.28 mmol) according to the general procedure **A**. The crude material (rr > 20:1) was purified by automated flash chromatography (10 g column, 5–60 % gradient of cyclohexane/ethyl acetate) to afford the product as a colorless oil (55.1 mg, 77 % yield, dr > 20:1, 88 % ee).

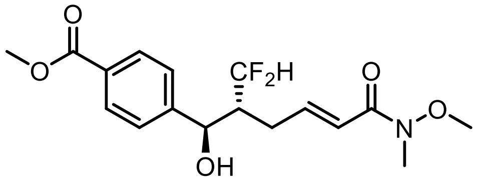

Note: due to high viscosity of the isolated compound, residual cyclohexane (≈ 5 %) is visible in the ^{1}H NMR spectrum. $[\alpha]_D^{20}$ = +21.8 (c = 1.3, CH_2Cl_2). ^{1}H NMR (400 MHz, CD_2Cl_2, 298 K): δ [ppm] = 8.03 – 7.98 (m, 2H), 7.48 – 7.43 (m, 2H), 6.84 – 6.74 (m, 1H), 6.32 (d, *J* = 15.4 Hz, 1H), 5.84 (td, *J* = 56.1, 4.5 Hz, 1H), 5.10 (d, *J* = 4.4 Hz, 1H), 3.88 (s, 3H), 3.62 (s, 3H), 3.55 – 3.33 (m, 1H), 3.12 (s, 3H), 2.54 – 2.27 (m, 3H). ^{13}C NMR (101 MHz, CD_2Cl_2, 298 K): δ [ppm] = 167.1, 166.6, 147.6, 144.8, 130.1, 130.0, 126.5, 120.9, 118.1 (t, $^1J_{CF}$ = 242.2 Hz), 71.4 (t, $^3J_{CF}$ = 5.5 Hz), 62.0, 52.4, 49.6 (t, $^2J_{CF}$ = 18.1 Hz), 32.5, 26.7 (t, $^3J_{CF}$ = 4.1 Hz). ^{19}F NMR (282 MHz, CD_2Cl_2, 298 K): δ [ppm] = –121.4 (d, *J* = 283.9 Hz), –124.3 (d, *J* = 283.4 Hz). IR (ATR): ṽ = 3400, 2954, 1720, 1660, 1612, 1437, 1389, 1281, 1180, 1113, 1043, 1019, 999, 963, 867, 774, 719 cm^{-1}. HRMS (ESI^+): *m/z*: calcd. for $[C_{17}H_{21}F_2NO_5Na]^+$ $[M+Na]^+$: 380.1280, found: 380.1280.

The ee was determined by HPLC analysis: Chiralpak 150 mm IG-3, 3 µm, 4.6 mm ∅, CH_3CN/H_2O = 70:30, v = 1.0 mL/min, λ = 235 nm, t(minor) = 8.00 min, t(major) = 6.69 min (racemate: left, enantioenriched sample: right).

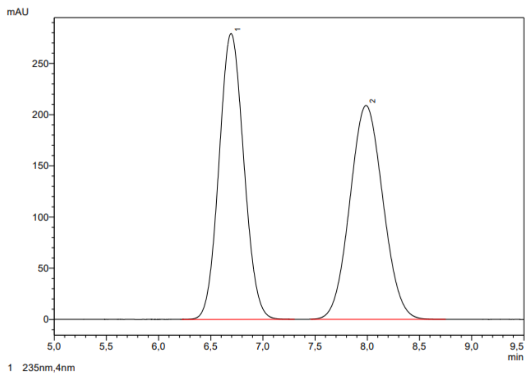

PDA Ch1 235nm

Peak #	Ret. Time	Area %	Name
1	6,69	49,98	1. Enantiomer
2	7,99	50,02	2. Enantiomer
Total		100,00	

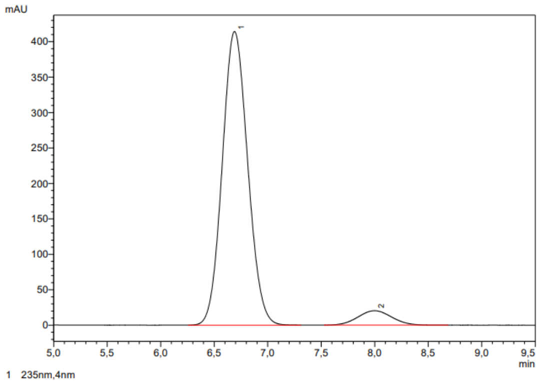

PDA Ch1 235nm

Peak #	Ret. Time	Area %	Name
1	6,69	93,83	1. Enantiomer 87.7 % ee
2	8,00	6,17	2. Enantiomer
Total		100,00	

(5*R*,6*R*,*E*)-6-(3,5-Dibromophenyl)-5-(difluoromethyl)-6-hydroxy-*N*-methoxy-*N*-methylhex-2-enamide (20i). The title compound was prepared from 3,5-dibromobenzaldehyde (52.8 mg, 0.20 mmol) and diene **15** (53.5 mg, 0.28 mmol) according to the general procedure **A**. The crude material (rr > 20:1) was purified by automated flash chromatography (10 g column, 5–60 % gradient of cyclohexane/ethyl acetate) to afford the product as a colorless oil (52.1 mg, 57 % yield, dr > 20:1, 83 % ee).

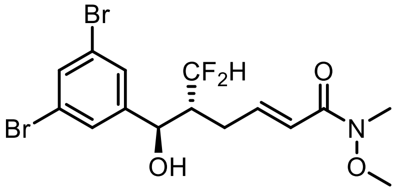

Note: due to high viscosity of the isolated compound, residual cyclohexane (≈ 5 %) is visible in the ^{1}H NMR spectrum. $[\alpha]_D^{20}$ = +4.0 (c = 0.6, CH_2Cl_2). ^{1}H NMR (400 MHz, CD_2Cl_2, 298 K): δ [ppm] = 7.62 – 7.59 (m, 1H), 7.49 (d, *J* = 1.8 Hz, 2H), 6.87 – 6.74 (m, 1H), 6.37 (d, *J* = 15.3 Hz, 1H), 5.86 (td, *J* = 56.0, 4.4 Hz, 1H), 5.03 (d, *J* = 4.0 Hz, 1H), 3.66 (s, 3H), 3.15 (s, 3H), 2.57 – 2.22 (m, 4H). ^{13}C NMR (101 MHz, CD_2Cl_2, 298 K): δ [ppm] = 166.6, 146.7, 144.5, 133.6, 128.5, 123.4, 121.1, 117.9 (t, $^1J_{CF}$ = 242.3 Hz), 70.5 (t, $^3J_{CF}$ = 5.6 Hz), 62.2, 49.5 (t, $^2J_{CF}$ = 18.2 Hz), 32.5, 26.7 (t, $^3J_{CF}$ = 4.0 Hz). ^{19}F NMR (282 MHz, CD_2Cl_2, 298 K): δ [ppm] = –121.8 (d, *J* = 283.9 Hz), –124.1 (d, *J* = 283.9 Hz). IR (ATR): ṽ = 3371, 2966, 2935, 1659, 1613, 1557, 1425, 1389, 1194, 1116, 1045, 999, 858, 742, 690 cm^{-1}. HRMS (ESI$^+$): *m/z*: calcd. for $[C_{15}H_{17}F_2NO_3Br_2Na]^+$ $[M+Na]^+$: 477.9435, found: 477.9434.

The ee was determined by HPLC analysis: Chiralpak 150 mm IG-3, 3 µm, 4.6 mm ∅, CH_3CN/H_2O = 50:50, v = 1.0 mL/min, λ = 220 nm, t(minor) = 8.09 min, t(major) = 14.35 min (racemate: left, enantioenriched sample: right).

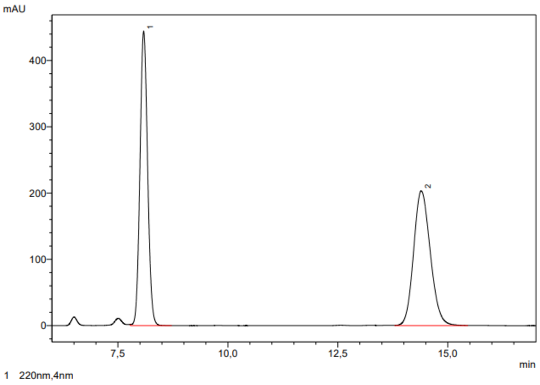

PDA Ch1 220nm

Peak #	Ret. Time	Area %	Name
1	8,09	49,87	1. Enantiomer
2	14,39	50,13	2. Enantiomer
Total		100,00	

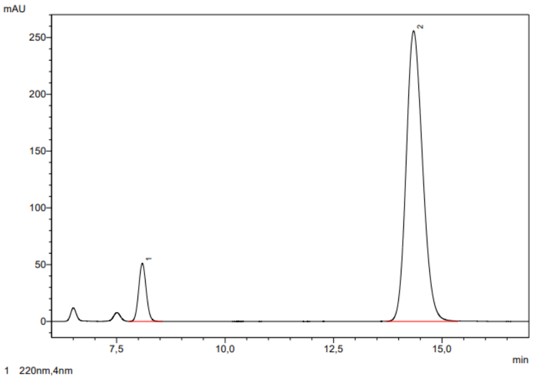

PDA Ch1 220nm

Peak #	Ret. Time	Area %	Name
1	8,09	8,41	1. Enantiomer
2	14,35	91,59	2. Enantiomer 83.2 % ee
Total		100,00	

(5*R*,6*R*,*E*)-5-(Difluoromethyl)-6-hydroxy-*N*-methoxy-*N*-methyl-6-(pyridin-4-yl)hex-2-en-amide (20j).

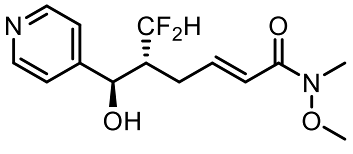

The title compound was prepared from pyridine-4-carbaldehyde (18.8 µL, 0.20 mmol) and diene **15** (53.5 mg, 0.28 mmol) according to the general procedure **A** but using three equivalents of BEt_3 to promote the conversion. The crude material (rr ≈ 8:1) was purified by automated flash chromatography (10 g column, 5–60 % gradient of cyclohexane/ethyl acetate) to afford the product as a colorless oil (34.0 mg, 57 % yield, dr > 20:1, 62 % ee).

$[\alpha]_D^{20}$ = +17.7 (c = 0.5, CH_2Cl_2). ^{1}H NMR (400 MHz, CD_2Cl_2, 298 K): δ [ppm] = 8.51 (d, *J* = 6.1 Hz, 2H), 7.32 (d, *J* = 6.1 Hz, 2H), 6.82 – 6.70 (m, 1H), 6.33 (d, *J* = 15.4 Hz, 1H), 5.92 (td, *J* = 56.1, 4.6 Hz, 1H), 5.11 (d, *J* = 3.6 Hz, 1H), 3.63 (s, 3H), 3.14 (s, 3H), 2.53 – 2.27 (m, 3H), 1.26 (br. s, 1H). ^{13}C NMR (101 MHz, CD_2Cl_2, 298 K): δ [ppm] = 166.5, 151.8, 150.0, 144.2, 121.6, 121.2, 118.1 (t, $^1J_{CF}$ = 242.3 Hz), 70.2 (t, $^3J_{CF}$ = 5.6 Hz), 62.1, 49.2 (t, $^2J_{CF}$ = 18.2 Hz), 32.5, 26.5 (t, $^3J_{CF}$ = 4.3 Hz). ^{19}F NMR (282 MHz, CD_2Cl_2, 298 K): δ [ppm] = –121.6 (d, *J* = 283.4 Hz), –123.9 (d, *J* = 283.4 Hz). IR (ATR): ṽ = 3323, 2923, 2854, 1660, 1605, 1415, 1387, 1180, 1114, 1041, 999, 959, 820, 694, 630 cm^{-1}. HRMS (ESI$^+$): *m/z*: calcd. for $[C_{14}H_{18}F_2N_2ONa]^+$ $[M+Na]^+$: 323.1177, found: 323.1177.

The ee was determined by HPLC analysis: Chiralpak 150 mm IG-3, 3 µm, 4.6 mm ∅, *n*-heptane/2-propanol = 90:10, v = 1.0 mL/min, λ = 215 nm, t(minor) = 24.20 min, t(major) = 27.58 min (racemate: left, enantioenriched sample: right).

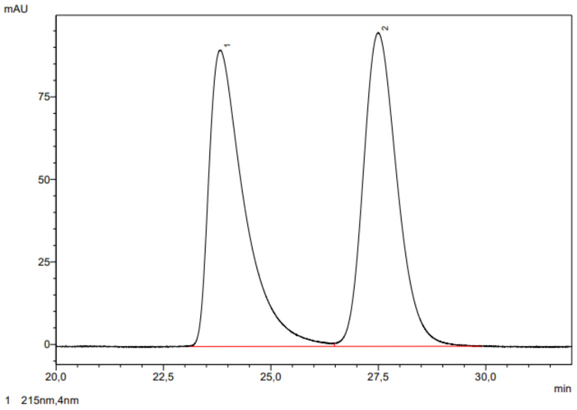

PDA Ch1 215nm

Peak #	Ret. Time	Area %	Name
1	23,82	49,79	1. Enantiomer
2	27,49	50,21	2. Enantiomer
Total		100,00	

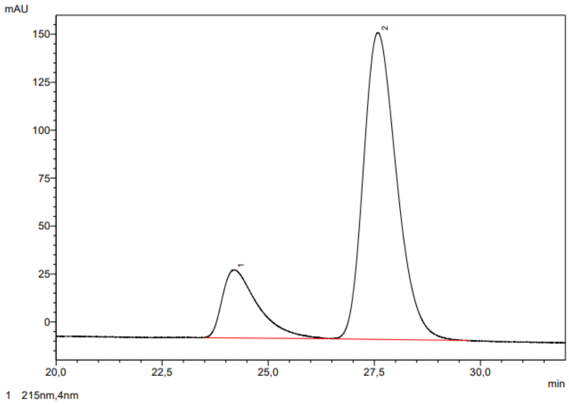

PDA Ch1 215nm

Peak #	Ret. Time	Area %	Name
1	24,20	19,18	1. Enantiomer
2	27,58	80,82	2. Enantiomer 61.6 % ee
Total		100,00	

(5*R*,6*R*,*E*)-5-(Difluoromethyl)-6-(furan-2-yl)-6-hydroxy-*N*-methoxy-*N*-methylhex-2-enamide

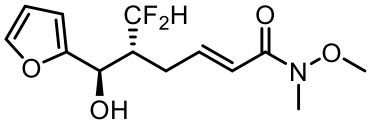

(20k). The title compound was prepared from furfural (16.6 µL, 0.20 mmol) and diene **15** (53.5 mg, 0.28 mmol) according to the general procedure **A**. The crude material (rr > 20:1) was purified by automated flash chromatography (10 g column, 5–60 % gradient of cyclohexane/ethyl acetate) to afford the product as a colorless oil (30.7 mg, 53 % yield, dr > 20:1, 94 % ee).

$[\alpha]_D^{20}$ = +5.0 (c = 1.1, CH_2Cl_2). ^{1}H NMR (400 MHz, CD_2Cl_2, 298 K): δ [ppm] = 7.41 (dd, J = 1.9, 0.9 Hz, 1H), 6.92 – 6.82 (m, 1H), 6.42 (d, J = 15.4 Hz, 1H), 6.46 – 6.31 (m, 2H), 5.83 (td, J = 56.2, 3.9 Hz, 1H), 4.96 (t, J = 4.3 Hz, 1H), 3.67 (s, 3H), 3.17 (s, 3H), 3.03 (d, J = 4.8 Hz, 1H), 2.59 – 2.50 (m, 3H). ^{13}C NMR (101 MHz, CD_2Cl_2, 298 K): δ [ppm] = 166.7, 154.5, 144.5, 142.7, 121.1, 117.7 (t, $^{1}J_{CF}$ = 242.0 Hz), 110.7, 107.7, 66.7 – 66.1 (m), 62.1, 47.5 (t, $^{2}J_{CF}$ = 18.4 Hz), 32.5, 27.2 (t, $^{3}J_{CF}$ = 4.3 Hz). ^{19}F NMR (282 MHz, CD_2Cl_2, 298 K): δ [ppm] = –120.9 (d, J = 285.0 Hz), –124.8 (d, J = 284.4 Hz). IR (ATR): ṽ = 3370, 2972, 2939, 1660, 1616, 1427, 1388, 1181, 1147, 1113, 1049, 1005, 884, 819, 742, 599 cm^{-1}. HRMS (ESI$^+$): *m/z*: calcd. for $[C_{13}H_{17}F_2NO_4Na]^+$ $[M+Na]^+$: 312.1018, found: 312.1018.

The ee was determined by HPLC analysis: Chiralpak 150 mm IG-3, 3 µm, 4.6 mm ∅, *n*-heptane/2-propanol = 90:10, v = 1.0 mL/min, λ = 215 nm, t(minor) = 13.44 min, t(major) = 14.56 min (racemate: left, enantioenriched sample: right).

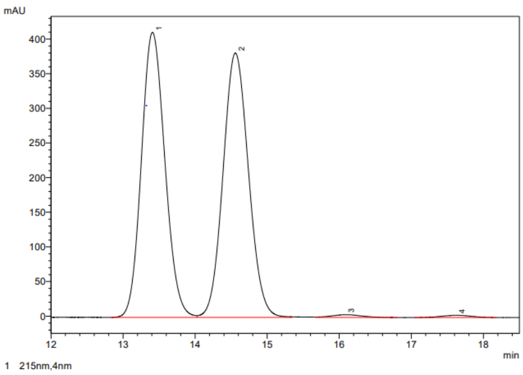

PDA Ch1 215nm

Peak #	Ret. Time	Area %	Name
1	13,41	49,33	1. Enantiomer
2	14,56	49,58	2. Enantiomer
3	16,08	0,60	
4	17,61	0,49	
Total		100,00	

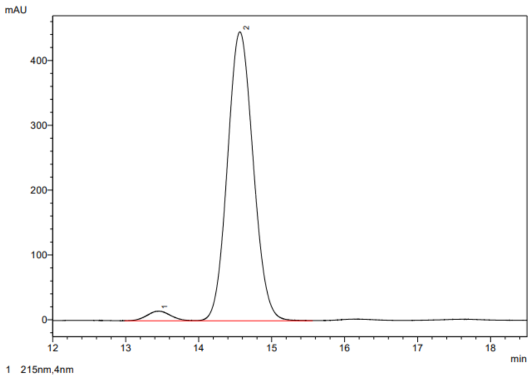

PDA Ch1 215nm

Peak #	Ret. Time	Area %	Name
1	13,44	2,98	1. Enantiomer
2	14,56	97,02	2. Enantiomer 94.0 % ee
Total		100,00	

tert-Butyl 2-((1_R_,2_R_,_E_)-2-(difluoromethyl)-1-hydroxy-6-(methoxy(methyl)amino)-6-oxohex-4-en-1-yl)-1_H_-pyrrole-1-carboxylate (20l). The title compound was prepared from 1-_tert_-butoxycarbonyl-pyrrol-2-carboxaldehyde (39.0 mg, 0.20 mmol) and diene **15** (53.5 mg, 0.28 mmol) according to the general procedure **A**. The crude material (rr > 20:1) was purified by automated flash chromatography (10 g column, 5–60 % gradient of cyclohexane/ethyl acetate) to afford the product as a colorless oil (50.5 mg, 65 % yield, dr > 20:1, 92 % ee).

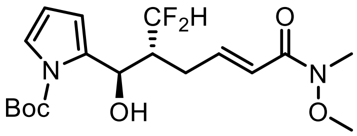

$[\alpha]_D^{20}$ = +3.2 (c = 1.5, CH_2Cl_2). ^{1}H NMR (400 MHz, CD_2Cl_2, 298 K): δ [ppm] = 7.23 (dd, J = 3.3, 1.8 Hz, 1H), 6.96 – 6.86 (m, 1H), 6.45 (d, J = 15.3 Hz, 1H), 6.26 – 6.22 (m, 1H), 6.13 (t, J = 3.4 Hz, 1H), 5.77 (td, J = 56.2, 3.9 Hz, 1H), 5.11 (t, J = 5.9 Hz, 1H), 3.95 (d, J = 6.8 Hz, 1H), 3.68 (s, 3H), 3.18 (s, 3H), 2.73 – 2.51 (m, 3H), 1.60 (s, 9H). ^{13}C NMR (101 MHz, CD_2Cl_2, 298 K): δ [ppm] = 166.8, 150.7, 145.1, 135.3, 123.1, 120.8, 118.3 (t, $^1J_{CF}$ = 242.4 Hz), 113.8, 110.7, 85.6, 66.4 (t, $^3J_{CF}$ = 6.2 Hz), 62.1, 46.6 (t, $^2J_{CF}$ = 18.1 Hz), 32.5, 28.1, 27.6 (t, $^3J_{CF}$ = 4.2 Hz). ^{19}F NMR (282 MHz, CD_2Cl_2, 298 K): δ [ppm] = –120.3 (d, J = 283.4 Hz), –125.2 (d, J = 282.8 Hz). IR (ATR): ṽ = 3391, 2978, 2935, 1737, 1660, 1618, 1414, 1372, 1328, 1257, 1163, 1130, 1059, 1010, 921, 894, 846, 773, 728, 570 cm^{-1}. HRMS (ESI$^+$): _m/z_: calcd. for $[C_{18}H_{26}F_2N_2O_5Na]^+$ $[M+Na]^+$: 411.1702, found: 411.1702.

The ee was determined by HPLC analysis: Chiralpak 150 mm IG-3, 3 µm, 4.6 mm ∅, _n_-heptane/2-propanol = 90:10, v = 1.0 mL/min, λ = 220 nm, t(minor) = 14.23 min, t(major) = 15.60 min (racemate: left, enantioenriched sample: right).

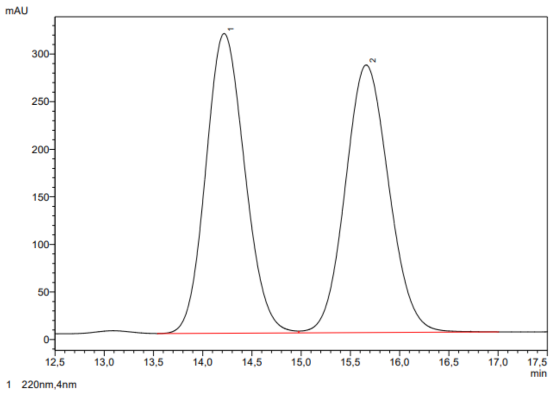

1 220nm,4nm

PDA Ch1 220nm

Peak #	Ret. Time	Area %	Name
1	14,22	50,03	1. Enantiomer
2	15,66	49,97	2. Enantiomer
Total		100,00	

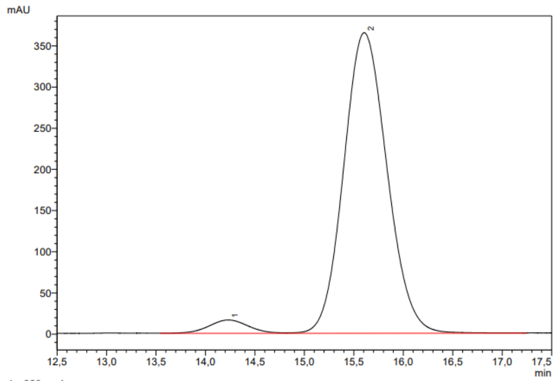

1 220nm,4nm

PDA Ch1 220nm

Peak #	Ret. Time	Area %	Name
1	14,23	3,90	1. Enantiomer
2	15,60	96,10	2. Enantiomer 92.2 % ee
Total		100,00	

(5*R*,6*R*,*E*)-5-(Difluoromethyl)-6-(2,3-dihydrobenzo[b][1,4]dioxin-6-yl)-6-hydroxy-*N*-methoxy-*N*-methylhex-2-enamide (20m). The title compound was prepared from 2,3-dihydrobenzo[b][1,4]dioxin-6-carbaldehyde (32.8 mg, 0.20 mmol) and diene **15** (53.5 mg, 0.28 mmol) according to the general procedure **A**. The crude material (rr > 20:1) was purified by automated flash chromatography (10 g column, 5–60 % gradient of cyclohexane/ethyl acetate) to afford the product as a colorless oil (61.6 mg, 86 % yield, dr > 20:1, 89 % ee).

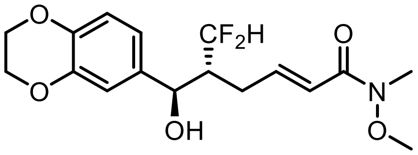

$[\alpha]_D^{20}$ = +19.0 (c = 1.3, CH_2Cl_2). ^{1}H NMR (400 MHz, CD_2Cl_2, 298 K): δ [ppm] = 6.89 – 6.78 (m, 4H), 6.37 (d, *J* = 15.3 Hz, 1H), 5.74 (td, *J* = 56.1, 4.1 Hz, 1H), 4.88 – 4.82 (m, 1H), 4.23 (s, 4H), 3.65 (s, 3H), 3.15 (s, 3H), 3.04 (d, *J* = 3.7 Hz, 1H), 2.54 – 2.46 (m, 2H), 2.34 – 2.19 (m, 1H). ^{13}C NMR (101 MHz, CD_2Cl_2, 298 K): δ [ppm] = 166.8, 145.1, 144.1, 143.7, 135.4, 120.7, 119.3, 118.2 (t, $^1J_{CF}$ = 241.3 Hz), 117.6, 115.3, 71.8 – 71.5 (m), 64.9, 64.8, 62.1, 49.8 (t, $^2J_{CF}$ = 17.9 Hz), 32.5, 26.9 (t, $^3J_{CF}$ = 4.2 Hz). ^{19}F NMR (282 MHz, CD_2Cl_2, 298 K): δ [ppm] = –121.0 (d, *J* = 283.4 Hz), –125.0 (d, *J* = 283.4 Hz). IR (ATR): ṽ = 3401, 2977, 2936, 2879, 1659, 1614, 1592, 1507, 1433, 1387, 1285, 1258, 1202, 1126, 1067, 998, 920, 888, 820, 751, 530, 444 cm^{-1}. HRMS (EI): *m/z*: calcd. for $[C_{17}H_{21}F_2NO_5]^+$ $[M]^+$: 357.1382, found: 357.1383.

The ee was determined by HPLC analysis: Chiralpak 150 mm IG-3, 3 µm, 4.6 mm ∅, *n*-heptane/2-propanol = 60:40, v = 1.0 mL/min, λ = 220 nm, t(minor) = 5.11 min, t(major) = 8.70 min (racemate: left, enantioenriched sample: right).

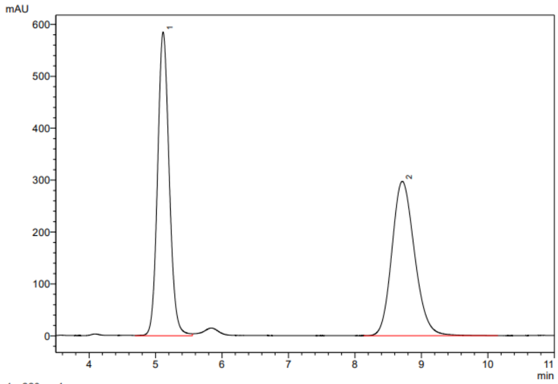

1 220nm,4nm

PDA Ch1 220nm

Peak #	Ret. Time	Area %	Name
1	5,11	50,24	1. Enantiomer
2	8,71	49,76	2. Enantiomer
Total		100,00	

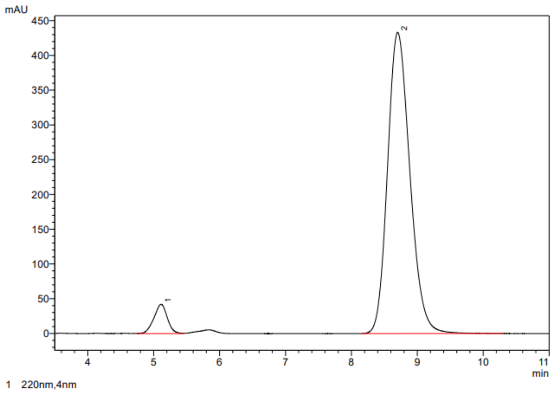

1 220nm,4nm

PDA Ch1 220nm

Peak #	Ret. Time	Area %	Name
1	5,11	5,50	1. Enantiomer
2	8,70	94,50	2. Enantiomer 89.0 % ee
Total		100,00	

(5*R*,6*R*,*E*)-6-(Benzo[b]thiophen-2-yl)-5-(difluoromethyl)-6-hydroxy-*N*-methoxy-*N*-methyl-hex-2-enamide (20n). The title compound was prepared from benzo[b]thiophen-2-carboxaldehyd (32.4 mg, 0.20 mmol) and diene **15** (53.5 mg, 0.28 mmol) according to the general procedure **A**. The crude material (rr > 20:1) was purified by automated flash chromatography (10 g column, 5–60 % gradient of cyclohexane/ethyl acetate) to afford the product as a colorless oil (48.1 mg, 68 % yield, dr > 20:1, 79 % ee).

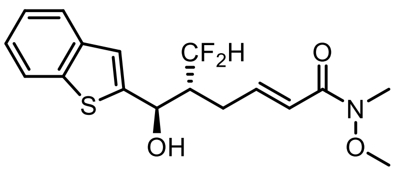

Note: due to high viscosity of the isolated compound, residual cyclohexane (≈ 5 %) is visible in the ^{1}H NMR spectrum. $[\alpha]_D^{20}$ = +5.1 (c = 0.8, CH_2Cl_2). ^{1}H NMR (400 MHz, CD_2Cl_2, 298 K): δ [ppm] = 7.86 – 7.70 (m, 2H), 7.39 – 7.27 (m, 2H), 7.26 (s, 1H), 6.99 – 6.87 (m, 1H), 6.41 (d, *J* = 15.3 Hz, 1H), 5.89 (td, *J* = 56.0, 4.2 Hz, 1H), 5.36 – 5.33 (m, 1H), 3.59 (s, 3H), 3.53 – 3.47 (m, 1H), 3.12 (s, 3H), 2.65 – 2.58 (m, 2H), 2.56 – 2.42 (m, 1H). ^{13}C NMR (101 MHz, CD_2Cl_2, 298 K): δ [ppm] = 166.7, 147.0, 144.6, 139.9, 139.7, 124.8, 124.7, 123.9, 122.8, 121.3, 121.1, 117.9 (t, $^1J_{CF}$ = 242.4 Hz), 69.1 (t, $^3J_{CF}$ = 6.0 Hz), 62.0, 49.8 (t, $^2J_{CF}$ = 18.3 Hz), 32.5, 27.1 (t, $^3J_{CF}$ = 4.2 Hz). ^{19}F NMR (282 MHz, CD_2Cl_2, 298 K): δ [ppm] = –121.4 (d, *J* = 284.4 Hz), –124.8 (d, *J* = 284.5 Hz). IR (ATR): ṽ = 3359, 2966, 2934, 1659, 1613, 1460, 1435, 1389, 1309, 1196, 1180, 1129, 1039, 997, 922, 864, 834, 750, 728, 692 cm^{-1}. HRMS (EI): *m/z*: calcd. for $[C_{17}H_{19}F_2NO_3S]^+$ $[M]^+$: 355.1048, found: 355.1049.

The ee was determined by HPLC analysis: Chiralpak 150 mm IG-3, 3 µm, 4.6 mm ∅, *n*-heptane/EtOH = 80:20, v = 1.0 mL/min, λ = 230 nm, t(minor) = 11.59 min, t(major) = 16.88 min (racemate: left, enantioenriched sample: right).

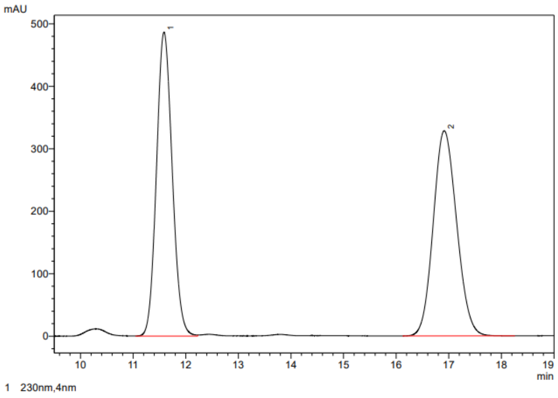

PDA Ch1 230nm

Peak #	Ret. Time	Area %	Name
1	11,59	49,92	1. Enantiomer
2	16,91	50,08	2. Enantiomer
Total		100,00	

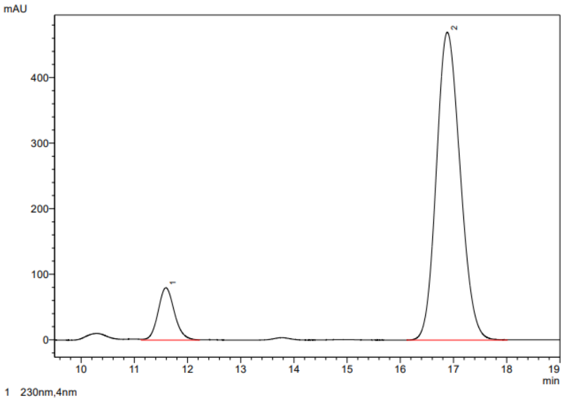

PDA Ch1 230nm

Peak #	Ret. Time	Area %	Name
1	11,59	10,52	1. Enantiomer
2	16,88	89,48	2. Enantiomer 79.0 % ee
Total		100,00	

(2*E*,5*R*,6*S*,7*E*)-5-(Difluoromethyl)-6-hydroxy-*N*-methoxy-*N*-methyl-8-phenylocta-2,7-dien-amide (21a). The title compound was prepared from cinnamaldehyde (25.2 µL, 0.20 mmol) and diene **15** (53.5 mg, 0.28 mmol) according to the general procedure **A.** The crude material (rr > 20:1) was purified by automated flash chromatography (10 g column, 5 – 60 % gradient of cyclohexane/ethyl acetate) to afford the product as a colorless oil (47.7 mg, 73 % yield, dr > 20:1, 93 % ee).

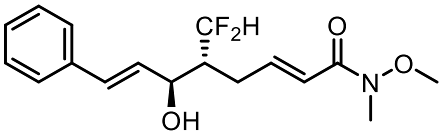

$[\alpha]_D^{20}$ = –26.3 (c = 0.6, CH_2Cl_2). ^{1}H NMR (400 MHz, CD_2Cl_2, 298 K): δ [ppm] = 7.43 – 7.22 (m, 5H), 7.01 – 6.88 (m, 1H), 6.64 (d, *J* = 15.9 Hz, 1H), 6.46 (d, *J* = 15.4 Hz, 1H), 6.27 – 5.82 (m, 2H), 4.56 (t, *J* = 5.6 Hz, 1H), 3.62 (s, 3H), 3.18 (s, 3H), 3.03 (br. s, 1H), 2.63 – 2.44 (m, 2H), 2.38 – 2.19 (m, 1H). ^{13}C NMR (101 MHz, CD_2Cl_2, 298 K): δ [ppm] = 166.8, 145.0, 136.8, 132.2, 129.5, 129.0, 128.3, 126.9, 121.1, 117.9 (t, $^1J_{CF}$ = 241.8 Hz), 71.20 – 70.91 (m), 62.0, 48.2 (t, $^2J_{CF}$ = 18.0 Hz), 32.5, 27.0 (t, $^3J_{CF}$ = 4.4 Hz). ^{19}F NMR (282 MHz, CD_2Cl_2, 298 K): δ [ppm] = –121.1 (d, *J* = 283.4 Hz), –124.2 (d, *J* = 283.4 Hz). IR (ATR): ṽ = 3394, 2971, 2936, 1660, 1616, 1425, 1388, 1304, 1180, 1120, 1044, 997, 970, 917, 754, 695, 525 cm^{-1}. HRMS (ESI$^+$): *m/z*: calcd. for $[C_{17}H_{21}F_2NO_3Na]^+$ $[M+Na]^+$: 348.1382, found: 348.1381.

The ee was determined by HPLC analysis: Chiralpak 150 mm IB-N-3, 3 µm, 4.6 mm ∅, *n*-heptane/2-propanol = 90:10, v = 1.0 mL/min, λ = 250 nm, t(minor) = 18.35 min, t(major) = 12.73 min (racemate: left, enantioenriched sample: right).

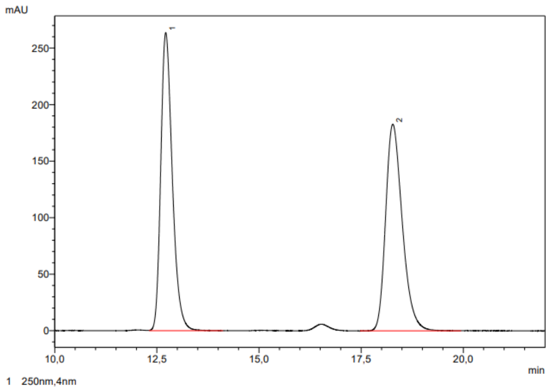

PDA Ch1 250nm

Peak #	Ret. Time	Area %	Name
1	12,72	49,89	1. Enantiomer
2	18,27	50,11	2. Enantiomer
Total		100,00	

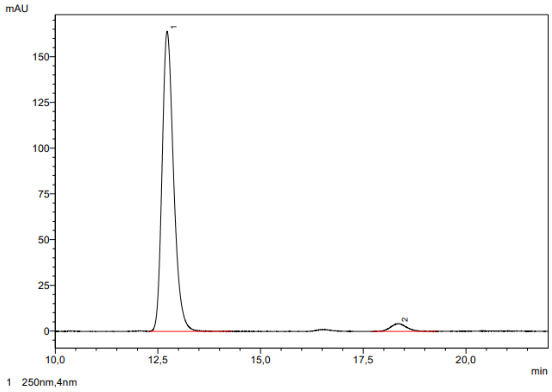

PDA Ch1 250nm

Peak #	Ret. Time	Area %	Name
1	12,73	96,51	1. Enantiomer 93.0 % ee
2	18,35	3,49	2. Enantiomer
Total		100,00	

(2*E*,5*R*,6*R*,7*E*)-5-(Difluoromethyl)-6-hydroxy-*N*-methoxy-*N*,7-dimethyl-8-phenylocta-2,7-dienamide (21b).

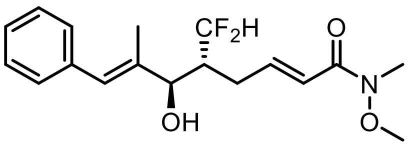

The title compound was prepared from (*E*)-2-methyl-3-phenylacrylaldehyde (27.8 µL, 0.20 mmol) and diene **15** (53.5 mg, 0.28 mmol) according to the general procedure **A**. The crude material (rr > 20:1) was purified by automated flash chromatography (10 g column, 5–60 % gradient of cyclohexane/ethyl acetate) to afford the product as a colorless oil (56.7 mg, 83 % yield, dr > 20:1, 67 % ee).

$[\alpha]_D^{20}$ = +13.2 (c = 0.9, CH_2Cl_2). ^{1}H NMR (400 MHz, CD_2Cl_2, 298 K): δ [ppm] = 7.37 – 7.19 (m, 5H), 7.00 – 6.90 (m, 1H), 6.65 (s, 1H), 6.48 – 6.40 (m, 1H), 5.92 (td, *J* = 56.3, 4.7 Hz, 1H), 4.43 (d, *J* = 4.9 Hz, 1H), 3.61 (s, 3H), 3.17 (s, 3H), 2.66 – 2.42 (m, 3H), 2.38 – 2.23 (m, 1H), 1.85 (d, *J* = 1.4 Hz, 3H). ^{13}C NMR (101 MHz, CD_2Cl_2, 298 K): δ [ppm] = 166.8, 145.5, 137.9, 137.7, 129.4, 128.5, 127.0, 126.9, 120.8, 118.6 (t, $^1J_{CF}$ = 242.1 Hz), 74.7 (t, $^3J_{CF}$ = 5.6 Hz), 62.0, 45.6 (t, $^2J_{CF}$ = 18.0 Hz), 32.5, 26.9 – 26.7 (m), 14.9. ^{19}F NMR (282 MHz, CD_2Cl_2, 298 K): δ [ppm] = –120.8 (d, *J* = 283.3 Hz), –124.4 (d, *J* = 282.3 Hz). IR (ATR): ṽ = 3400, 2927, 1659, 1614, 1442, 1386, 1179, 1133, 1100, 1041, 999, 922, 820, 751, 700, 575, 517, 444 cm^{-1}. HRMS (EI): *m/z*: calcd. for $[C_{18}H_{23}F_2NO_3]^+$ $[M]^+$: 339.1640, found: 339.1640.

The ee was determined by HPLC analysis: Chiralpak 150 mm IG-3, 3 µm, 4.6 mm ∅, CH_3CN/H_2O = 50:50, v = 1.0 mL/min, λ = 242 nm, t(minor) = 10.68 min, t(major) = 23.01 min (racemate: left, enantioenriched sample: right).

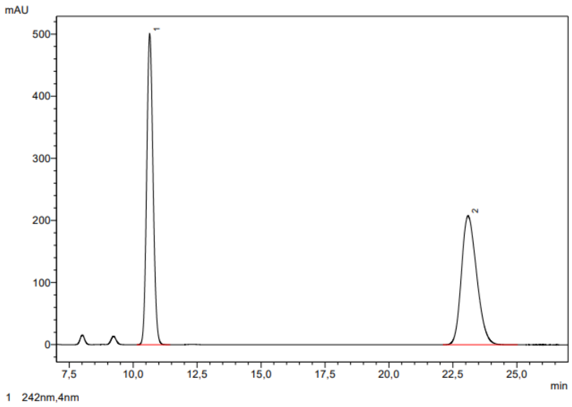

PDA Ch1 242nm

Peak #	Ret. Time	Area %	Name
1	10,65	50,00	1. Enantiomer
2	23,09	50,00	2. Enantiomer
Total		100,00	

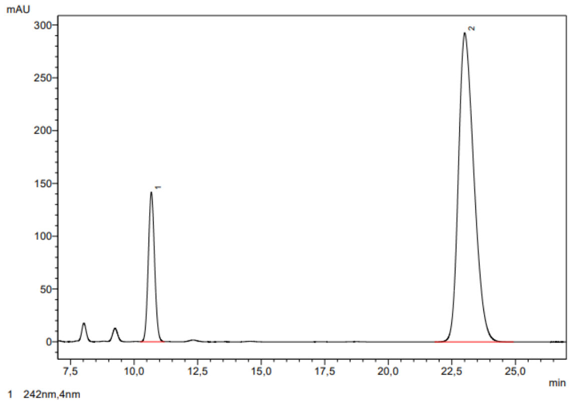

PDA Ch1 242nm

Peak #	Ret. Time	Area %	Name
1	10,68	16,43	1. Enantiomer
2	23,01	83,57	2. Enantiomer 67.1 % ee
Total		100,00	

(5*R*,6*S*,*E*)-5-(Difluoromethyl)-6-hydroxy-*N*-methoxy-*N*,8-dimethylnona-2,7-dienamide (21c). The title compound was prepared from 3-methyl-2-butenal (19.3 µL, 0.20 mmol) and diene **15** (53.5 mg, 0.28 mmol) according to the general procedure **A**. The crude material (rr > 20:1) was purified by automated flash chromatography (10 g column, 5–60 % gradient of cyclohexane/ethyl acetate) to afford the product as a colorless oil (31.7 mg, 57 % yield, dr > 20:1, 91 % ee).

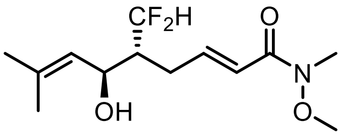

$[\alpha]_D^{20}$ = –2.8 (c = 1.2, CH_2Cl_2). ^{1}H NMR (400 MHz, CD_2Cl_2, 298 K): δ [ppm] = 6.98 – 6.87 (m, 1H), 6.51 – 6.42 (m, 1H), 5.92 (td, *J* =56.1, 3.4 Hz, 1H), 5.27 – 5.20 (m, 1H), 4.56 – 4.50 (m, 1H), 3.68 (s, 3H), 3.19 (s, 3H), 2.59 – 2.43 (m, 2H), 2.27 – 2.06 (m, 2H), 1.74 (d, *J* = 1.5 Hz, 3H), 1.67 (d, *J* = 1.5 Hz, 3H). ^{13}C NMR (101 MHz, CD_2Cl_2, 298 K): δ [ppm] = 166.8, 145.3, 137.6, 124.9, 120.9, 118.0 (t, $^1J_{CF}$ = 241.5 Hz), 67.3 – 67.1 (m), 62.1, 48.5 (t, $^2J_{CF}$ = 17.9 Hz), 32.5, 27.2 (t, $^3J_{CF}$ = 4.3 Hz), 26.0, 18.3. ^{19}F NMR (282 MHz, CD_2Cl_2, 298 K): δ [ppm] = –120.8 (d, *J* = 284.4 Hz), –124.7 (d, *J* = 284.4 Hz). IR (ATR): ṽ = 3397, 2937, 1660, 1620, 1442, 1385, 1180, 1122, 1036, 995, 918, 689, 621, 526, 444 cm^{-1}. HRMS (ESI$^+$): *m/z*: calcd. for $[C_{13}H_{21}F_2NO_3Na]^+$ $[M+Na]^+$: 300.1381, found: 300.1379.

The ee was determined by HPLC analysis: Chiralpak 150 mm IG-3, 3 µm, 4.6 mm ∅, *n*-heptane/EtOH = 90:10, v = 1.0 mL/min, λ = 215 nm, t(minor) = 13.42 min, t(major) = 16.50 min (racemate: left, enantioenriched sample: right).

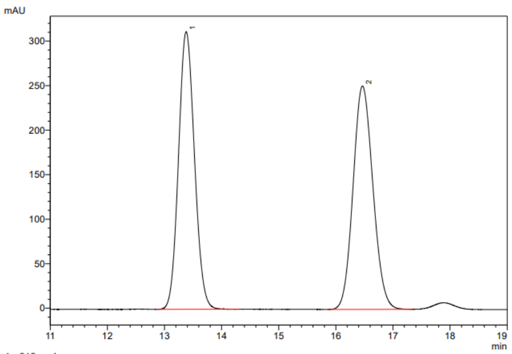

1 215nm,4nm

PDA Ch1 215nm

Peak #	Ret. Time	Area %	Name
1	13,38	49,98	1. Enantiomer
2	16,47	50,02	2. Enantiomer
Total		100,00	

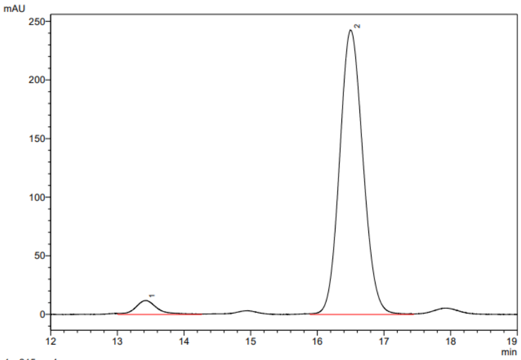

1 215nm,4nm

PDA Ch1 215nm

Peak #	Ret. Time	Area %	Name
1	13,42	4,40	1. Enantiomer
2	16,50	95,60	2. Enantiomer 91.2 % ee
Total		100,00	

(2*E*,5*R*,6*S*,7*E*)-5-(Difluoromethyl)-6-hydroxy-*N*-methoxy-*N*,8,12-trimethyltrideca-2,7,11-trienamide (21d).

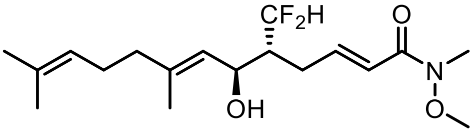

The title compound was prepared from (*E*)-3,7-dimethylocta-2,6-dienal (62.3 µL, 0.20 mmol) and diene **15** (53.5 mg, 0.28 mmol) according to the general procedure **A**. The crude material (rr > 20:1) was purified by automated flash chromatography (10 g column, 5–60 % gradient of cyclohexane/ethyl acetate) to afford the product as a colorless oil (29.1 mg, 42 % yield, dr > 20:1, 92 % ee).

$[\alpha]_D^{20}$ = –4.2 (c = 0.9, CH_2Cl_2). ^1H NMR (400 MHz, CD_2Cl_2, 298 K): δ [ppm] = 6.98 – 6.88 (m, 1H), 6.46 (d, *J* = 15.3 Hz, 1H), 5.91 (td, *J* = 56.2, 3.2 Hz, 1H), 5.27 – 5.19 (m, 1H), 5.11 – 5.04 (m, 1H), 4.54 (dd, *J* = 9.1, 5.8 Hz, 1H), 3.68 (s, 3H), 3.19 (s, 3H), 2.61 – 2.40 (m, 2H), 2.17 – 2.00 (m, 5H), 1.94 (br. s, 1H), 1.70 – 1.66 (m, 6H), 1.61 (s, 3H). ^{13}C NMR (101 MHz, CD_2Cl_2, 298 K): δ [ppm] = 166.8, 145.3, 140.9, 132.3, 124.8, 124.2, 120.9, 118.0 (t, $^1J_{CF}$ = 241.7 Hz), 67.3 – 67.1(m), 62.06, 48.52 (t, $^2J_{CF}$ = 17.9 Hz), 40.03, 32.51, 27.18 (t, $^3J_{CF}$ = 4.2 Hz), 26.60, 25.77, 17.78, 16.69. ^{19}F NMR (282 MHz, CD_2Cl_2, 298 K): δ [ppm] = –120.7 (d, *J* = 284.4 Hz), –124.7 (d, *J* = 284.5 Hz). IR (ATR): ṽ = 3403, 2962, 2855, 1660, 1630, 1441, 1384, 1178, 1120, 1046, 997, 917, 697, 521, 449 cm^{-1}. HRMS (ESI$^+$): *m/z*: calcd. for $[C_{18}H_{29}F_2NO_3Na]^+$ $[M+Na]^+$: 368.2008, found: 368.2009.

The ee was determined by HPLC analysis: Chiralpak 150 mm IG-3, 3 µm, 4.6 mm ∅, CH_3CN/H_2O = 45:55, v = 1.0 mL/min, λ = 254 nm, t(minor) = 19.34 min, t(major) = 31.40 min (racemate: left, enantioenriched sample: right).

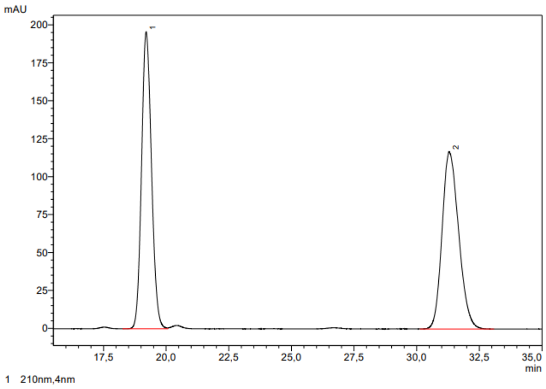

PDA Ch1 210nm

Peak #	Ret. Time	Area %	Name
1	19,20	50,05	1. Enantiomer
2	31,29	49,95	2. Enantiomer
Total		100,00	

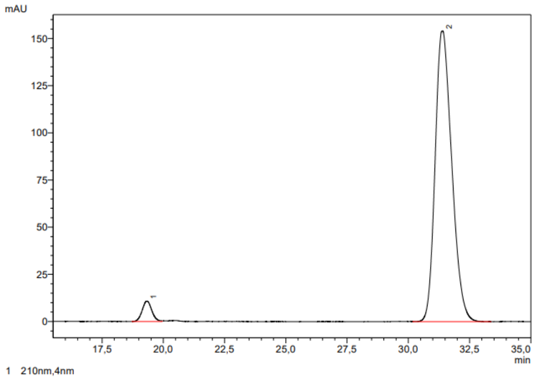

PDA Ch1 210nm

Peak #	Ret. Time	Area %	Name
1	19,34	3,92	1. Enantiomer
2	31,40	96,08	2. Enantiomer 92.2 % ee
Total		100,00	

(5*R*,6*R*,*E*)-5-(Difluoromethyl)-6-((1*R*,5*S*)-6,6-dimethylbicyclo[3.1.1]hept-2-en-2-yl)-6-hydroxy-*N*-methoxy-*N*-methylhex-2-enamide (21e).

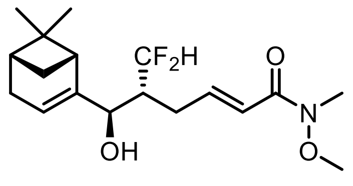

The title compound was prepared from (–)-myrtenal (30.4 µL, 0.20 mmol) and diene **15** (53.5 mg, 0.28 mmol) according to the general procedure **B**. The crude material (rr > 20:1) was purified by automated flash chromatography (10 g column, 5–60 % gradient of cyclohexane/ethyl acetate) to afford the product as a colorless oil (49.0 mg, 71 % yield, dr ≈ 4:1). The diastereomers were inserparable by flash chromatography but are clearly visible in obtained spectra.

Note: due to high viscosity of the isolated compound, residual cyclohexane (≈ 6 %) is visible in the ^{1}H NMR spectrum. $[\alpha]_D^{20}$ = +2.1 (c = 1.3, CH_2Cl_2). ^{1}H NMR (400 MHz, CD_2Cl_2, 298 K): δ [ppm] = 6.93 – 6.84 (m, 1H), 6.49 – 6.37 (m, 1H), 6.06 – 5.69 (m, 1H), 5.61 – 5.54 (m, 1H), 4.26 (s, 1H), 3.67 (s, 3H), 3.18 (s, 3H), 2.55 – 2.28 (m, 5H), 2.16 – 2.01 (m, 3H), 1.96 (br. s, 1H), 1.68 (br. s, 1H), 1.29 (s, 3H), 0.84 (s, 3H). ^{13}C NMR (101 MHz, CD_2Cl_2, 298 K): δ [ppm] = 166.8, 148.2, 145.4, 120.8, 118.9 (t, $^1J_{CF}$ = 242.2 Hz), 118.7, 72.3 – 72.1 (m), 62.1, 45.1 (t, $^2J_{CF}$ = 17.8 Hz), 43.5, 41.5, 38.4, 32.5, 31.8, 31.5, 26.6 – 26.4 (m), 26.3, 21.2. ^{19}F NMR (282 MHz, CD_2Cl_2, 298 K): δ [ppm] = –120.37 (d, *J* = 281.7 Hz), –123.7 (d, *J* = 281.7 Hz). IR (ATR): ṽ = 3394, 2918, 1660, 1620, 1464, 1421, 1384, 1132, 1046, 1032, 997, 960 cm^{-1}. HRMS (ESI$^+$): *m/z*: calcd. for $[C_{18}H_{27}F_2NO_3Na]^+$ $[M+Na]^+$: 366.1851, found: 366.1854.

(5*R*,6*R*,*E*)-5-(Difluoromethyl)-6-hydroxy-*N*-methoxy-*N*-methyl-6-((*S*)-4-(prop-1-en-2-yl)-cyclo-hex-1-en-1-yl)hex-2-enamide (21f).

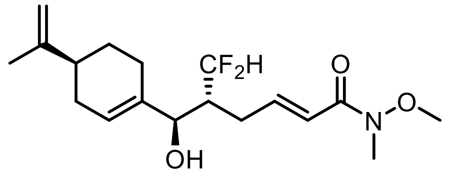

The title compound was prepared from (–)-perillaldehyde (34.4 µL, 0.20 mmol) and diene **15** (53.5 mg, 0.28 mmol) according to the general procedure **B**. The crude material (rr > 20:1) was purified by automated flash chromatography (10 g column, 5–60 % gradient of cyclohexane/ethyl acetate) to afford the product as a colorless oil (54.7 mg, 80 % yield, dr ≈ 4:1). The diastereomers were inserparable by flash chromatography but are clearly visible in the recorded spectra.

$[\alpha]_D^{20}$ = +14.3 (c = 0.9, CH_2Cl_2). ^{1}H NMR (400 MHz, CD_2Cl_2, 298 K): δ [ppm] = 6.98 – 6.84 (m, 1H), 6.43 (d, *J* = 15.4 Hz, 1H), 6.07 – 5.62 (m, 2H), 4.77 – 4.67 (m, 2H), 4.27 – 4.17 (m, 1H), 3.68 (s, 3H), 3.18 (s, 3H), 2.61 – 2.37 (m, 2H), 2.27 – 1.78 (m, 9H), 1.74 (s, 3H). ^{13}C NMR (101 MHz, CD_2Cl_2, 298 K): δ [ppm] = 166.9, 150.1, 145.6, 137.6, 124.3, 120.6, 118.5 (t, $^1J_{CF}$ = 241.9 Hz), 108.9, 73.4 – 73.2 (m), 62.1, 45.6 (t, $^2J_{CF}$ = 17.9 Hz), 41.4, 32.5, 30.7, 27.7, 26.9 (t, $^3J_{CF}$ = 4.2 Hz), 24.9, 20.9. ^{19}F NMR (282 MHz, CD_2Cl_2, 298 K): δ [ppm] = –120.5 (d, *J* = 282.3 Hz), –124.9 (d, *J* = 282.3 Hz). IR (ATR): ṽ = 3395, 2966, 2923, 1660, 1618, 1436, 1385, 1179, 1134, 1039, 999, 919, 889 cm^{-1}. HRMS (EI$^+$): *m/z*: calcd. for $[C_{18}H_{27}F_2NO_3]^+$ $[M]^+$: 343.1953, found: 343.1950.

Products Derived from Other Fluorinated Dienamides

(5*R*,6*S*,*E*)-5-(1,1-Difluoroethyl)-6-hydroxy-*N*-methoxy-*N*-methyl-8-phenyloct-2-enamide

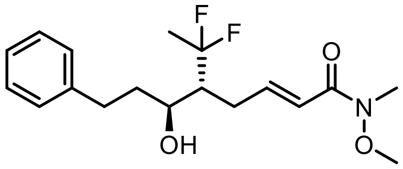

(23a). The title compound was prepared from phenylpropanal (26.3 µL, 0.20 mmol) and diene **22** (57.5 mg, 0.28 mmol) according to the general procedure **B**. The crude material (rr ≈ 3:1) was purified by automated flash chromatography (10 g column, 5–60 % gradient of cyclohexane/ethylacetate) to afford the product as a colorless oil (42.1 mg, 61 % yield, dr > 20:1, 94 % ee).

$[\alpha]_D^{20}$ = –9.4 (c = 0.8, CH_2Cl_2). ^{1}H NMR (400 MHz, CD_2Cl_2, 298 K): δ [ppm] = 7.31 – 7.14 (m, 5H), 6.97 – 6.87 (m, 1H), 6.46 (d, *J* = 15.3 Hz, 1H), 4.06 – 4.00 (m, 1H), 3.65 (s, 3H), 3.19 (s, 3H), 2.84 – 2.74 (m, 1H), 2.69 – 2.55 (m, 2H), 2.52 – 2.43 (m, 1H), 2.16 – 2.00 (m, 1H), 1.94 – 1.84 (m, 2H), 1.80 – 1.71 (m, 1H), 1.64 (t, *J* = 19.6 Hz, 3H). ^{13}C NMR (101 MHz, CD_2Cl_2, 298 K): δ [ppm] = 166.7, 146.2, 142.1, 128.8 (2C), 126.6 (t, $^1J_{CF}$ = 241.4 Hz), 126.3, 120.6, 69.5 (t, $^3J_{CF}$ = 5.0 Hz), 62.1, 50.7 (t, $^2J_{CF}$ = 21.4 Hz), 37.7, 32.8, 32.5, 27.2 (t, $^1J_{CF}$ = 4.3 Hz), 22.5 (t, $^2J_{CF}$ = 27.5 Hz). ^{19}F NMR (282 MHz, CD_2Cl_2, 298 K): δ [ppm] = –90.2 (qd, *J* = 19.5, 14.0 Hz), –90.9 (dqd, *J* = 244.8, 19.5, 13.8 Hz). IR (ATR): ṽ = 3417, 2939, 1660, 1616, 1391, 1143, 997, 913, 749, 701 cm^{-1}. HRMS (ESI$^+$) : *m/z*: calcd. for $[C_{18}H_{25}F_2NO_3Na]^+$ $[M+Na]^+$: 364.1695, found: 364.1694.

The ee was determined by HPLC analysis: Chiralpak 150 mm IG-3, 3 µm, 4.6 mm ∅, CH_3CN/H_2O = 40:60, v = 1.0 mL/min, λ = 220 nm, t(minor) = 15.16 min, t(major) = 16.34 min (racemate: left, enantioenriched sample: right).

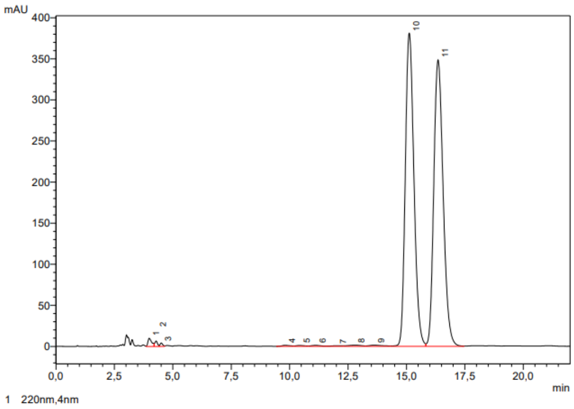

PDA Ch1 220nm

Peak #	Ret. Time	Area %	Name
1	3,99	0,57	
2	4,29	0,26	
3	4,51	0,17	
4	9,81	0,14	
5	10,44	0,13	
6	11,11	0,16	
7	11,99	0,11	
8	12,78	0,28	
9	13,62	0,28	
10	15,12	49,00	1. Enantiomer
11	16,35	48,91	2. Enantiomer
Total		100,00	

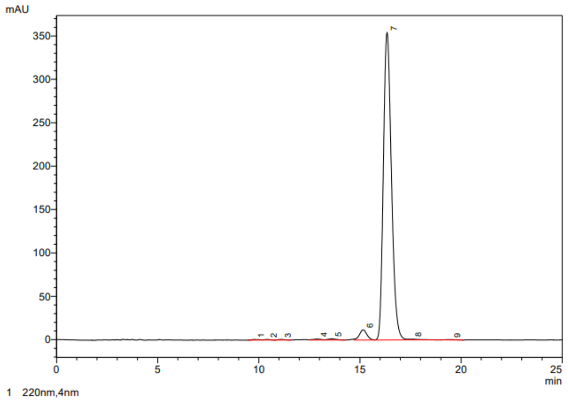

PDA Ch1 220nm

Peak #	Ret. Time	Area %	Name
1	9,78	0,17	
2	10,40	0,14	
3	11,11	0,16	
4	12,89	0,32	
5	13,60	0,38	
6	15,16	2,94	1. Enantiomer
7	16,34	95,39	2. Enantiomer 94.0 % ee
8	17,55	0,37	
9	19,48	0,13	
Total		100,00	

(*R*,*E*)-6,6-Difluoro-5-((*R*)-hydroxy(phenyl)methyl)-*N*-methoxy-*N*-methylhept-2-enamide (23b). The title compound was prepared from benzaldehyde (20.2 µL, 0.20 mmol) and diene **22** (57.5 mg, 0.28 mmol) according to the general procedure **B**. The crude material (rr > 20:1) was purified by automated flash chromatography (10 g column, 5–60 % gradient of cyclohexane/ethylacetate) to afford the product as a colorless oil (51.0 mg, 81 % yield, dr > 20:1, 92 % ee).

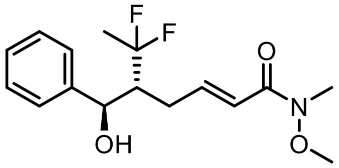

$[\alpha]_D^{20}$ = +45.5 (c = 0.9, CH_2Cl_2). ^{1}H NMR (400 MHz, CD_2Cl_2, 298 K): δ [ppm] = 7.39 – 7.32 (m, 4H), 7.29 – 7.24 (m, 1H), 6.65 – 6.54 (m, 1H), 6.21 (d, *J* = 15.3 Hz, 1H), 5.26 (s, 1H), 3.60 (s, 3H), 3.11 (s, 3H), 2.54 – 2.40 (m, 3H), 2.40 – 2.28 (m, 1H), 1.72 (t, *J* = 19.7 Hz, 3H). ^{13}C NMR (101 MHz, CD_2Cl_2, 298 K): δ [ppm] = 166.7, 146.0, 143.1, 128.7, 127.8, 126.4, 126.0, 120.1, 71.1 (dd, *J* = 6.8, 4.5 Hz), 62.0, 53.1 (t, $^2J_{CF}$ = 21.6 Hz), 32.4, 26.4 (dd, *J* = 4.9, 3.1 Hz), 22.54 (t, $^2J_{CF}$ = 27.3 Hz). ^{19}F NMR (282 MHz, CD_2Cl_2, 298 K): δ [ppm] = –87.8 (dqd, *J* = 245.0, 19.8, 12.9 Hz), –90.1 (dqd, *J* = 244.7, 19.6, 13.0 Hz). IR (ATR): ṽ = 3392, 2939, 1658, 1613, 1390, 1193, 1135, 999, 917, 767, 705, 527 cm^{-1}. HRMS (ESI^+) : *m/z*: calcd. for $[C_{16}H_{21}F_2NO_3Na]^+$ $[M+Na]^+$: 336.1382, found: 336.1381.

The ee was determined by HPLC analysis: Chiralpak 150 mm IG-3, 3 µm, 4.6 mm ∅, CH_3CN/H_2O = 40:60, v = 1.0 mL/min, λ = 220 nm, t(minor) = 8.11 min, t(major) = 15.23 min (racemate: left, enantioenriched sample: right).

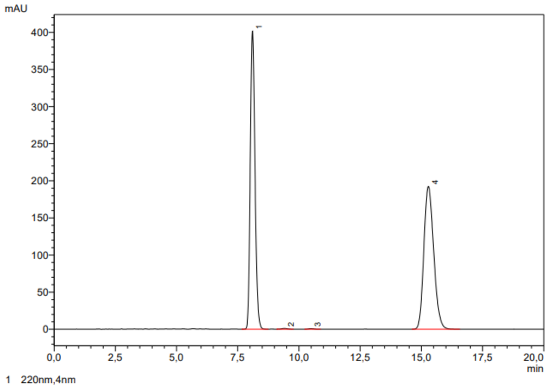

PDA Ch1 220nm

Peak #	Ret. Time	Area %	Name
1	8,10	49,90	1. Enantiomer
2	9,41	0,14	
3	10,49	0,11	
4	15,29	49,85	2. Enantiomer
Total		100,00	

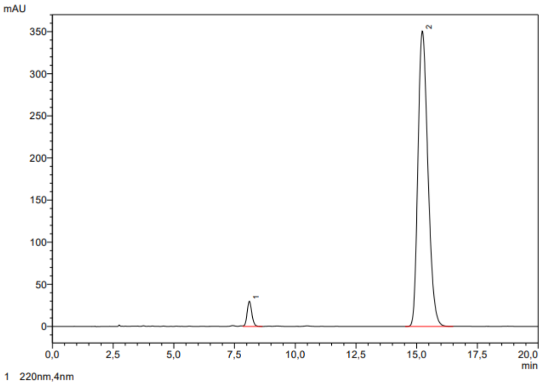

PDA Ch1 220nm

Peak #	Ret. Time	Area %	Name
1	8,11	3,89	1. Enantiomer
2	15,23	96,11	2. Enantiomer 92.2 % ee
Total		100,00	

(5*R*,6*S*,*E*)-5-(1,1-Difluoroethyl)-6-hydroxy-*N*-methoxy-*N*,8-dimethylnona-2,7-dienamide

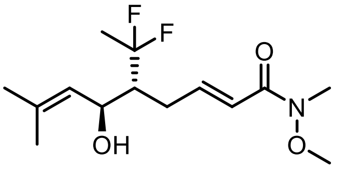

(23c). The title compound was prepared from 3-methylbut-2-enal (19.3 μL, 0.20 mmol) and diene **22** (57.5 mg, 0.28 mmol) according to the general procedure **B**. The crude material (rr > 20:1) was purified by automated flash chromatography (10 g column, 5–60 % gradient of cyclohexane/ethylacetate) to afford the product as a colorless oil (51.2 mg, 87 % yield, dr > 20:1, 90 % ee).

$[\alpha]_D^{20}$ = +22.6 (c = 1.2, CH_2Cl_2). ^{1}H NMR (400 MHz, $CDCl_3$, 298 K): δ [ppm] = 7.02 – 6.93 (m, 1H), 6.46 (d, *J* = 15.3 Hz, 1H), 5.32 – 5.28 (m, 1H), 4.75 (dd, *J* = 8.5, 2.6 Hz, 1H), 3.69 (s, 3H), 3.24 (s, 3H), 2.65 – 2.45 (m, 2H), 2.17 – 2.05 (m, 1H), 1.74 – 1.60 (m, 10H). ^{13}C NMR (101 MHz, $CDCl_3$, 298 K): δ [ppm] = 166.8, 146.4, 136.1, 125.8 (t, $^1J_{CF}$ = 241.7 Hz), 125.6, 120.0, 66.9 (t, $^3J_{CF}$ = 5.3 Hz), 61.9, 51.3 (t, $^2J_{CF}$ = 21.6 Hz), 32.5, 27.5 (t, $^3J_{CF}$ = 4.4 Hz), 26.0, 22.6 (t, $^2J_{CF}$ = 27.4 Hz), 18.4. ^{19}F NMR (282 MHz, $CDCl_3$, 298 K): δ [ppm] –89.6 (dqd, *J* = 39.3, 19.6, 13.4 Hz). IR (ATR): ṽ = 3416, 2937, 1659, 1616, 1423, 1388, 1180, 1147, 1105, 998, 911, 843, 526 cm^{-1}. HRMS (ESI$^+$) : *m/z*: calcd. for $[C_{14}H_{23}F_2NO_3Na]^+$ $[M+Na]^+$: 314.1538, found: 314.1539.

The ee was determined by HPLC analysis: Chiralpak 150 mm IG-3, 3 μm, 4.6 mm ∅, n-heptan/2-propanol = 90:10, v = 1.0 mL/min, λ = 215 nm, t(minor) = 12.48 min, t(major) = 15.79 min (racemate: left, enantioenriched sample: right).

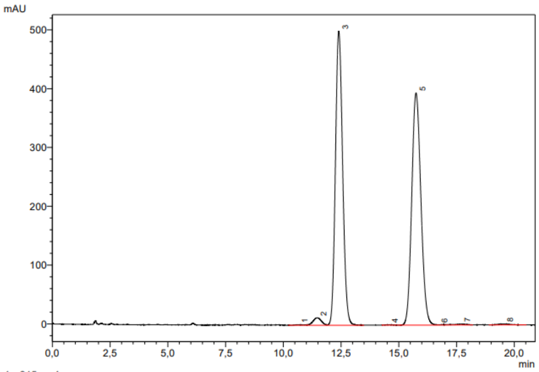

1 215nm,4nm

PDA Ch1 215nm

Peak #	Ret. Time	Area %	Name
1	10,64	0,14	
2	11,47	1,45	
3	12,40	48,72	1. Enantiomer
4	14,56	0,08	
5	15,75	48,96	2. Enantiomer
6	16,69	0,15	
7	17,69	0,21	
8	19,54	0,30	
Total		100,00	

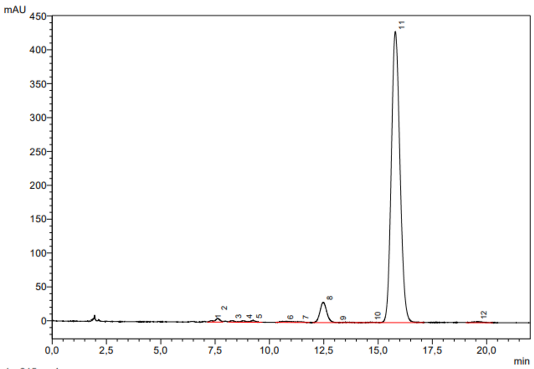

1 215nm,4nm

PDA Ch1 215nm

Peak #	Ret. Time	Area %	Name
1	7,34	0,19	
2	7,63	0,60	
3	8,28	0,25	
4	8,78	0,25	
5	9,23	0,30	
6	10,67	0,45	
7	11,38	0,21	
8	12,48	5,04	1. Enantiomer
9	13,10	0,12	
10	14,68	0,11	
11	15,79	92,11	2. Enantiomer 89.6 % ee
12	19,57	0,37	
Total		100,00	

(5*S*,6*S*,*E*)-6-hydroxy-*N*-methoxy-*N*-methyl-8-phenyl-5-(2,2,2-trifluoroethyl)oct-2-enamide

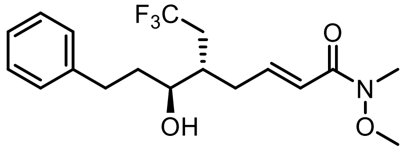

(25a). The title comound was prepared from phenylpropanal (26.3 µL, 0.20 mmol) and diene **24a** (62.5 mg, 0.28 mmol) according to the general procedure **B**. The crude material (rr > 20:1) was purified by automated flash chromatography (10 g column, 5–60 % gradient of cyclohexane/ethylacetate) to afford the product as a colorless oil (58.2 mg, 81 % yield, dr > 20:1, 95 % ee).

$[\alpha]_D^{20}$ = –31.6 (c = 1.3, CH_2Cl_2). ^{1}H NMR (400 MHz, CD_2Cl_2, 298 K): δ [ppm] = 7.31 – 7.14 (m, 5H), 6.91 – 6.81 (m, 1H), 6.45 (d, *J* = 15.3 Hz, 1H), 3.82 – 3.73 (m, 1H), 3.67 (s, 3H), 3.20 (s, 3H), 2.87 – 2.77 (m, 1H), 2.69 – 2.59 (m, 1H), 2.44 – 2.36 (m, 3H), 2.13 – 2.01 (m, 2H), 1.90 (br. s, 1H), 1.82 – 1.71 (m, 2H). ^{13}C NMR (101 MHz, CD_2Cl_2, 298 K): δ [ppm] = 166.5, 144.5, 142.2, 128.8, 128.7, 127.9 (q, $^1J_{CF}$ = 123.8 Hz), 126.4, 121.6, 72.0, 62.1, 38.2, 35.9, 34.1 (q, $^2J_{CF}$ = 27.5 Hz), 32.92, 32.5, 32.2. ^{19}F NMR (282 MHz, CD_2Cl_2, 298 K): δ [ppm] –63.8 (t, *J* = 11.3 Hz). IR (ATR): ṽ = 3416, 2940, 1658, 1616, 1440, 1385, 1254, 1136, 1052, 750, 700 cm^{-1}. HRMS (ESI$^+$) : *m/z*: calcd. for $[C_{18}H_{24}F_3NO_3Na]^+$ $[M+Na]^+$: 382.1600, found: 382.1599.

The ee was determined by HPLC analysis: Chiralpak 150 mm IG-3, 3 µm, 4.6 mm ∅, CH_3CN/H_2O = 35:65, v = 1.0 mL/min, λ = 220 nm, t(minor) = 33.95 min, t(major) = 37.30 min (racemate: left, enantioenriched sample: right).

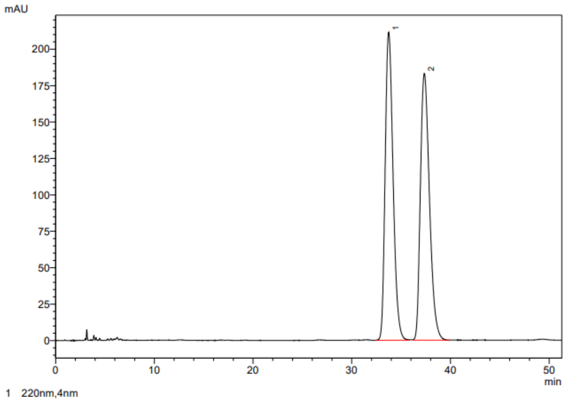

PDA Ch1 220nm

Peak #	Ret. Time	Area %	Name
1	33,74	50,10	
2	37,36	49,90	
Total		100,00	

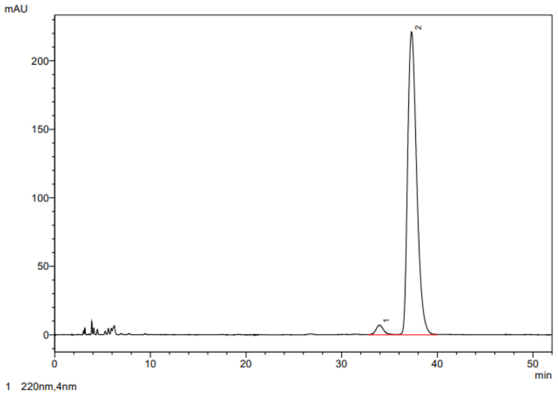

PDA Ch1 220nm

Peak #	Ret. Time	Area %	Name
1	33,95	2,75	1. Enantiomer
2	37,30	97,25	2. Enantiomer 94.5 % ee
Total		100,00	

(*S*,*E*)-7,7,7-Trifluoro-5-((*R*)-hydroxy(phenyl)methyl)-*N*-methoxy-*N*-methylhept-2-enamide

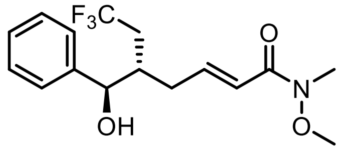

(25b). The title compound was prepared from benzaldehyde (15.2 μL, 0.15 mmol) and diene **24a** (46.9 mg, 0.21 mmol) according to the general procedure **B**. The crude material (rr > 20:1) was purified by automated flash chromatography (10 g column, 5–60 % gradient of cyclohexane/ethylacetate) to afford the product as a colorless oil (44.4 mg, 89 % yield, dr > 20:1, 91 % ee).

$[\alpha]_D^{20}$ = +4.6 (c = 1.1, CH_2Cl_2). ^{1}H NMR (400 MHz, CD_2Cl_2, 298 K): δ [ppm] = 7.40 – 7.26 (m, 5H), 6.90 – 6.78 (m, 1H), 6.42 (d, *J* = 15.3 Hz, 1H), 4.86 (s, 1H), 3.66 (s, 3H), 3.16 (s, 3H), 2.65 (d, *J* = 3.6 Hz, 1H), 2.52 – 2.38 (m, 1H), 2.39 – 2.25 (m, 3H), 2.13 – 1.98 (m, 1H). ^{13}C NMR (101 MHz, CD_2Cl_2, 298 K): δ [ppm] = 166.6, 144.5, 142.7, 129.3, 128.8, 128.1, 126.6, 121.8, 74.4, 62.1, 39.7 (q, $^3J_{CF}$ = 2.1 Hz), 33.8 (q, $^2J_{CF}$ = 27.6 Hz), 32.5, 31.3. ^{19}F NMR (282 MHz, CD_2Cl_2, 298 K): δ [ppm] –63.5 (t, *J* = 11.3 Hz). IR (ATR): ṽ = 3395, 2940, 1658, 1615, 1438, 1386, 1253, 1140, 1048, 752, 704 cm^{-1}. HRMS (ESI$^+$) : *m/z*: calcd. for $[C_{16}H_{20}F_3NO_3Na]^+$ $[M+Na]^+$: 354.1288, found: 354.1286.

The ee was determined by HPLC analysis: Chiralpak 150 mm IG-3, 3 μm, 4.6 mm ∅, CH_3CN/H_2O = 40:60, v = 1.0 mL/min, λ = 220 nm, t(minor) = 9.81 min, t(major) = 11.03 min (racemate: left, enantioenriched sample: right).

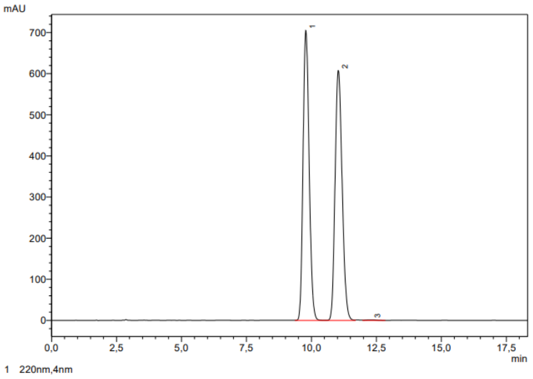

PDA Ch1 220nm

Peak #	Ret. Time	Area %	Name
1	9,79	49,96	1. Enantiomer
2	11,04	49,88	2. Enantiomer
3	12,29	0,16	
Total		100,00	

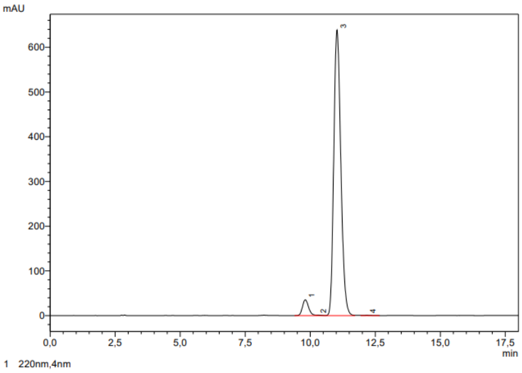

PDA Ch1 220nm

Peak #	Ret. Time	Area %	Name
1	9,81	4,60	1. Enantiomer
2	10,26	0,10	
3	11,03	95,18	2. Enantiomer 90.8 % ee
4	12,17	0,11	
Total		100,00	

(5*S*,6*R*,*E*)-6-hydroxy-*N*-methoxy-*N*,8-dimethyl-5-(2,2,2-trifluoroethyl)nona-2,7-dienamide

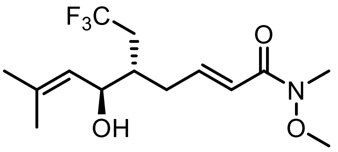

(25c). The title comound was prepared from 3-methylbut-2-enal (19.3, 0.20 mmol) and diene **24a** (62.5 mg, 0.28 mmol) according to the general procedure **B**. The crude material (rr > 20:1) was purified by automated flash chromatography (10 g column, 5–60 % gradient of cyclohexane/ethylacetate) to afford the product as a colorless oil (49.1 mg, 79 % yield, dr > 20:1, 92 % ee).

$[\alpha]_D^{20}$ = –22.1 (c = 0.8, CH_2Cl_2). ^{1}H NMR (400 MHz, $CDCl_3$, 298 K): δ [ppm] = 7.00 – 6.90 (m, 1H), 6.45 (d, *J* = 15.3 Hz, 1H), 5.17 (dp, *J* = 8.9, 1.4 Hz, 1H), 4.40 (dd, *J* = 8.9, 5.6 Hz, 1H), 3.69 (s, 3H), 3.23 (s, 3H), 2.57 – 2.48 (m, 1H), 2.42 – 2.25 (m, 2H), 2.11 – 1.92 (m, 2H), 1.75 (d, *J* = 1.4 Hz, 3H), 1.73 (br. s, 1H), 1.66 (d, *J* = 1.4 Hz, 3H). ^{13}C NMR (101 MHz, $CDCl_3$, 298 K): δ [ppm] = 166.6, 144.5, 137.9, 127.3 (q, $^1J_{CF}$ = 277.0 Hz), 124.7, 121.3, 68.8, 61.8, 38.6 (q, $^4J_{CF}$ = 2.0 Hz), 33.2 (q, $^2J_{CF}$ = 27.8 Hz), 32.4, 32.1, 26.1, 18.6. ^{19}F NMR (282 MHz, $CDCl_3$, 298 K): δ [ppm] = –63.3 (t, *J* = 11.2 Hz). IR (ATR): ṽ = 3416, 2939, 1660, 1619, 1441, 1385, 1256, 1140, 1043, 839 cm^{-1}. HRMS (ESI$^+$) : *m/z*: calcd. for $[C_{14}H_{22}F_3NO_3Na]^+$ $[M+Na]^+$: 332.1440, found: 332.1444.

The ee was determined by HPLC analysis: Chiralpak 150 mm IG-3, 3 µm, 4.6 mm ∅, CH_3CN/H_2O = 35:65, v = 1.0 mL/min, λ = 215 nm, t(major) = 10.80 min, t(minor) = 12.58 min (racemate: left, enantioenriched sample: right).

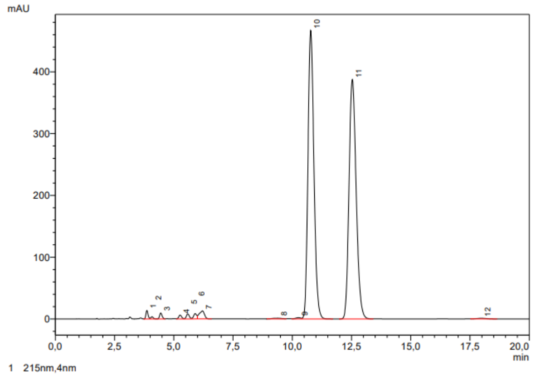

PDA Ch1 215nm

Peak #	Ret. Time	Area %	Name
1	3,86	0,50	
2	4,08	0,14	
3	4,45	0,42	
4	5,26	0,31	
5	5,59	0,43	
6	5,90	0,47	
7	6,21	1,16	
8	9,39	0,19	
9	10,26	0,18	
10	10,78	48,09	1. Enantiomer
11	12,53	47,96	2. Enantiomer
12	17,98	0,15	
Total		100,00	

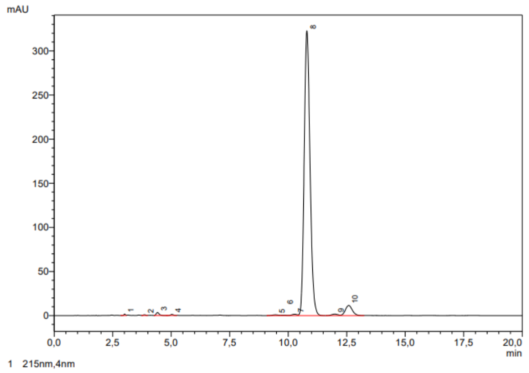

PDA Ch1 215nm

Peak #	Ret. Time	Area %	Name
1	3,01	0,13	
2	3,86	0,11	
3	4,41	0,53	
4	5,03	0,19	
5	9,46	0,26	
6	9,82	0,12	
7	10,27	0,37	
8	10,80	93,77	1. Enantiomer 91.8 % ee
9	11,98	0,51	
10	12,58	4,00	2. Enantiomer
Total		100,00	

(5*R*,6*S*,*E*)-6-Hydroxy-*N*-methoxy-*N*-methyl-5-(perfluoroethyl)-8-phenyloct-2-enamide (26).

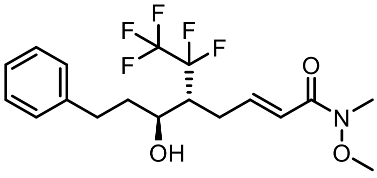

The title compound was prepared from phenylpropanal (26.3 µL, 0.20 mmol) and diene **24a** (72.6 mg, 0.28 mmol) according to the general procedure **B**. The crude material (rr = 1:1) was purified by automated flash column chromatography (10 g column, 5–60 % gradient of cyclohexane/ethylacetate) to afford the product as a colorless oil (31.6 mg, 40 % yield, 76 % ee).

$[\alpha]_D^{20}$ = –5.2 (c = 1.2, CH_2Cl_2). ^1H NMR (400 MHz, CD_2Cl_2, 298 K): δ [ppm] = 7.32 – 7.15 (m, 5H), 6.97 – 6.81 (m, 1H), 6.48 (dt, *J* = 15.4, 1.6 Hz, 1H), 4.15 (ddd, *J* = 9.2, 4.2, 1.8 Hz, 1H), 3.64 (s, 3H), 3.19 (s, 3H), 2.87 – 2.76 (m, 1H), 2.72 – 2.58 (m, 3H), 2.47 – 2.30 (m, 1H), 2.05 – 1.86 (m, 2H), 1.83 – 1.70 (m, 1H). ^{13}C NMR (101 MHz, CD_2Cl_2, 298 K): δ [ppm] = 166.5, 144.6, 141.7, 128.9, 128.8, 126.4, 121.3, 119.7 (dt, $^1J_{CF}$ = 287.1, $^2J_{CF}$ = 37.1 Hz), 117.3 (q, $^2J_{CF}$ = 36.6 Hz), 68.2 (t, $^3J_{CF}$ = 4.1 Hz), 62.1, 46.0 (t, $^2J_{CF}$ = 18.5 Hz), 37.5, 32.7, 27.3, 25.9. ^{19}F NMR (282 MHz, CD_2Cl_2, 298 K): δ [ppm] = –82.5, –115.4 (d, *J* = 274.5 Hz), -117.1 (d, *J* = 275.1 Hz). IR (ATR): ṽ = 3408, 2939, 1662, 1619, 1387, 1196, 1123, 1001, 922, 701 cm^{-1}. HRMS (GC-EI): *m/z*: calcd. for $[C_{18}H_{22}F_5NO_3]$ [M]: 395.1514, found: 395.1516.

The ee was determined by HPLC analysis: Chiralpak 150 mm IG-3, 3 µm, 4.6 mm ∅, CH_3CN/H_2O = 40:60, v = 1.0 mL/min, λ = 210 nm, t(minor) = 15.77 min, t(major) = 17.39 min (racemate: left, enantioenriched sample: right).

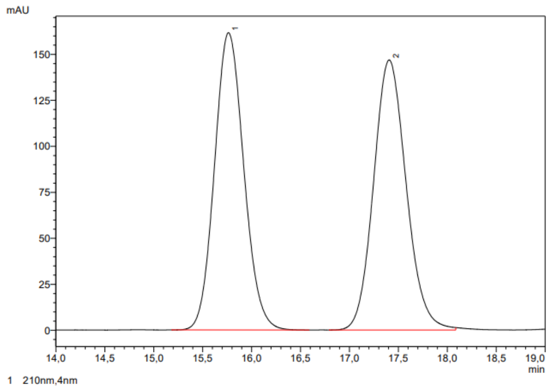

PDA Ch1 210nm

Peak #	Ret. Time	Area %	Name
1	15,76	49,77	1. Enantiomer
2	17,41	50,23	2. Enantiomer
Total		100,00	

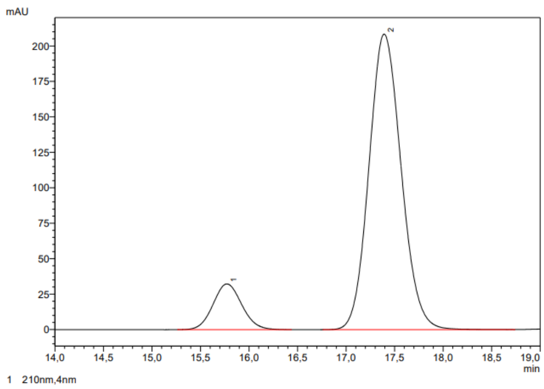

PDA Ch1 210nm

Peak #	Ret. Time	Area %	Name
1	15,77	12,14	1. Enantiomer
2	17,39	87,86	2. Enantiomer 75.7 % ee
Total		100,00	

Applications

(5*R*,6*S*,*E*)-6-((*tert*-Butyldimethylsilyl)oxy)-5-(difluoromethyl)-*N*-methoxy-*N*,8-dimethylnon-2-enamide (28).

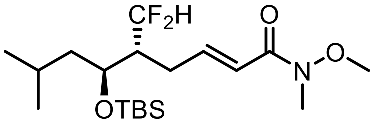

tert-Butyldimethylchlorosilane (105.2 mg, 0.70 mmol) and imidazole (91.4 mg, 1.34 mmol) were added to a solution of alcohol **19c** (150.0 mg, 0.54 mmol) in DMF (0.5 mL), and the resulting mixture was stirred at room temperature for 40 h. The reaction was quenched with hexane (20 mL), the organic phase was washed with brine (2 x 10 mL), dried over Mg_2SO_4 and concentrated in *vacuo*. The crude material was purified by automated flash chromatography (10 g column, 1-10 % gradient of cyclohexane/ethyl acetate) to give the title compound as a white crystalline solid (50.1 mg, 71 % yield).

m.p. = 36-38 °C. $[\alpha]_D^{20}$ = –3.9 (c = 0.6, CH_2Cl_2). ^{1}H NMR (400 MHz, $CDCl_3$, 298 K): δ [ppm] = 7.01 – 6.90 (m, 1H), 6.44 (d, *J* = 15.3 Hz, 1H), 5.85 (td, *J* = 56.7, 4.5 Hz, 1H), 3.99 – 3.91 (m, 1H), 3.68 (s, 3H), 3.23 (s, 3H), 2.46 – 2.37 (m, 2H), 2.13 – 1.99 (m, 1H), 1.62 – 1.52 (m, 1H), 1.42 – 1.27 (m, 2H), 0.91 – 0.84 (m, 15H), 0.07 – 0.03 (m, 6H). ^{13}C NMR (101 MHz, $CDCl_3$, 298 K): δ [ppm] = 166.6, 145.1, 120.6, 117.7 (t, $^1J_{CF}$ = 241.5 Hz), 68.8 – 68.4 (m), 61.8, 47.1 (t, $^2J_{CF}$ = 18.0 Hz), 43.2 – 43.0 (m), 32.4, 26.4 (t, $^3J_{CF}$ = 4.6 Hz), 25.9, 24.5, 22.8 (d, $^3J_{CF}$ = 5.0 Hz), 18.1, –4.2, –4.6. ^{19}F NMR (282 MHz, $CDCl_3$, 298 K): δ [ppm] = –119.8 (d, *J* = 282.8 Hz), –123.8 (d, *J* = 282.8 Hz). IR (ATR): ṽ = 2956, 2931, 2858, 1667, 1637, 1465, 1410, 1380, 1255, 1089, 1061, 1002, 895, 836, 776 cm^{-1}. HRMS (ESI$^+$): *m/z*: calcd. for $[C_{19}H_{37}F_2NO_3SiNa]^+$ $[M+Na]^+$: 416.2403, found: 416.2404.

(6*R*,7*S*,*E*)-7-((*tert*-Butyldimethylsilyl)oxy)-6-(difluoromethyl)-9-methyldec-3-en-2-one (29).

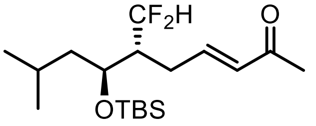

Compound **28** (50 mg, 0.13 mmol) was dissolved in THF (2 mL) and the resulting solution was cooled to 0 °C. A solution of methylmagnesium bromide (3 M, 0.11 mL, 0.32 mmol) was added dropwise and the resulting mixture was stirred for 2 h while reaching ambient temperature. After quenching of the reaction with sat. aq. NH_4Cl solution (5 mL), the aqueous layer was extracted with EtOAc (3 x 10 mL). The combined organic layers were washed with brine, dried over Mg_2SO_4 and concentrated in *vacuo*. The residue was purified by automated flash chromatography (10 g column, 1-5 % gradient of cyclohexane/ethyl acetate), affording the title compound as a colorless oil (32 mg, 73 % yield).

$[\alpha]_D^{20}$ = -0.7 (c = 0.8, CH_2Cl_2). ^{1}H NMR (400 MHz, $CDCl_3$, 298 K): δ [ppm] = 6.86 – 6.76 (m, 1H), 6.11 (dt, *J* = 15.9, 1.4 Hz, 1H), 5.83 (td, *J* = 56.7, 5.0 Hz, 1H), 4.01 – 3.94 (m, 1H), 2.46 – 2.36 (m, 2H), 2.24 (s, 3H), 2.10 – 1.98 (m, 1H), 1.61 – 1.49 (m, 1H), 1.43 – 1.29 (m, 2H), 0.91 – 0.86 (m, 15H), 0.05 (d, *J* = 4.3 Hz, 6H). ^{13}C NMR (101 MHz, $CDCl_3$, 298 K): δ [ppm] = 198.3, 145.9, 132.8, 117.8 (t, $^1J_{CF}$ = 241.7 Hz), 68.7 (t, $^3J_{CF}$ = 5.5 Hz), 47.0 (t, $^2J_{CF}$ = 17.9 Hz), 43.3, 27.0, 26.6 (t, $^3J_{CF}$ = 4.5 Hz), 25.9, 24.6, 22.8 (d, *J* = 35.6 Hz), 18.1, –4.2, –4.7. ^{19}F NMR (282 MHz, $CDCl_3$, 298 K): δ [ppm] = –119.3 (d, *J* = 282.8 Hz), –123.6 (d, *J* = 282.8 Hz). IR (ATR): ṽ = 2957, 2931, 2859, 1701, 1679, 1631, 1470, 1362, 1254, 1142, 1094, 1066, 981, 940, 897, 837, 808, 776 cm^{-1}. HRMS (ESI$^+$): *m/z*: calcd. for $[C_{18}H_{34}F_2O_2SiNa]^+$ $[M+Na]^+$: 371.2188, found: 371.2187.

1-((4*R*,5*S*)-4-(Difluoromethyl)-5-isobutyltetrahydrofuran-2-yl)propan-2-one (27a).

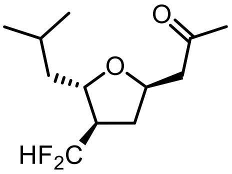

Alcohol **19c** (43.0 mg, 0.15 mmol) was dissolved in THF (2 mL) and the resulting solution cooled to 0 °C. A solution of methylmagnesium bromide (3 M in Et_2O, 0.17 mL, 0.51 mmol) was added dropwise. The mixture was then stirred at room temperature for 2 h before the reaction was quenched with sat. aq. NH_4Cl (2 mL) and the aqueous phase extracted with EtOAc (3 x 20 mL). The combined organic layers were dried over Mg_2SO_4 and then concentrated under *vacuo*. The residue was purified by automated flash chromatography (10 g column, 5-40 %

gradient of cyclohexane/ethyl acetate) to give the title compound as a colorless oil (32.4 mg, 69 % yield, dr ≈ 5:1).

Data of the major isomer: ^{1}H NMR (400 MHz, $CDCl_3$, 298 K): δ [ppm] = 5.71 (td, *J* = 56.6, 5.0 Hz, 1H), 4.32 (dq, *J* = 8.8, 6.2 Hz, 1H), 4.07 (ddd, *J* = 9.4, 5.4, 3.9 Hz, 1H), 2.80 (dd, *J* = 16.2, 6.7 Hz, 1H), 2.57 (dd, *J* = 16.2, 6.2 Hz, 1H), 2.37 – 2.27 (m, 2H), 2.18 (s, 3H), 1.78 – 1.70 (m, 1H), 1.54 – 1.45 (m, 2H), 1.26 (ddd, *J* = 13.9, 8.5, 3.9 Hz, 1H), 0.91 (d, *J* = 6.9 Hz, 3H), 0.90 (d, *J* = 6.4 Hz, 3H). ^{13}C NMR (101 MHz, $CDCl_3$, 298 K): δ [ppm] = 207.0, 117.4 (t, $^{1}J_{CF}$ = 241.4 Hz), 76.5 (dd, $^{3}J_{CF}$ = 5.3, 3.3 Hz), 73.4, 49.6 (t, $^{2}J_{CF}$ = 20.0 Hz), 49.2, 44.3, 32.7 (dd, $^{3}J_{CF}$ = 5.0, 2.9 Hz), 30.9, 25.3, 23.4, 22.0. ^{19}F NMR (282 MHz, $CDCl_3$, 298 K): δ [ppm] = –118.1 (d, *J* = 281.8 Hz), –119.6 (d, *J* = 281.9 Hz). IR (ATR): ṽ = 3418, 2956, 2937, 2872, 1660, 1620, 1467, 1420, 1385, 1178, 1090, 1038, 998, 913 cm^{-1}. HRMS (ESI^{+}): *m/z*: calcd. for $[C_{12}H_{20}F_2O_2Na]^+$ $[M+Na]^+$: 257.1324, found: 257.1324.

Data of minor isomer: ^{1}H NMR (400 MHz, $CDCl_3$, 298 K): δ [ppm] = 5.75 (td, *J* = 56.4, 4.9 Hz, 1H), 4.18 (dq, *J* = 9.0, 6.3 Hz, 1H), 3.88 (ddd, *J* = 9.0, 6.6, 4.0 Hz, 1H), 2.78 (dd, *J* = 15.8, 6.9 Hz, 1H), 2.56 (dd, *J* = 15.8, 6.0 Hz, 1H), 2.28 – 2.22 (m, 1H), 2.18 (s, 3H), 2.16 – 2.12 (m, 1H), 1.78 – 1.70 (m, 1H), 1.69 – 1.63 (m, 1H), 1.54 – 1.45 (m, 1H), 1.38 (ddd, *J* = 13.8, 8.4, 4.0 Hz, 1H), 0.91 (d, *J* = 6.9 Hz, 3H), 0.90 (d, *J* = 6.4 Hz, 3H). ^{13}C NMR (101 MHz, $CDCl_3$, 298 K): δ [ppm] = 206.9, 117.2 (t, $^{1}J_{CF}$ = 241.4 Hz), 77.0 (dd, $^{3}J_{CF}$ = 4.9, 3.8 Hz), 74.6, 49.5, 48.9 (t, $^{2}J_{CF}$ = 19.5 Hz), 45.1, 32.3 (dd, $^{3}J_{CF}$ = 4.8, 3.4 Hz), 30.9, 25.4, 23.5, 22.1. ^{19}F NMR (282 MHz, $CDCl_3$, 298 K): δ [ppm] = –119.2 (d, *J* = 283.1 Hz), –120.3 (d, *J* = 283.0 Hz).

IR (ATR): ṽ = 3418, 2956, 2937, 2872, 1660, 1620, 1467, 1420, 1385, 1178, 1090, 1038, 998, 913 cm^{-1}. HRMS (ESI^{+}): *m/z*: calcd. for $[C_{12}H_{20}F_2O_2Na]^+$ $[M+Na]^+$: 257.1324, found: 257.1324.

The diastereomers were assigned based on NOESY data:

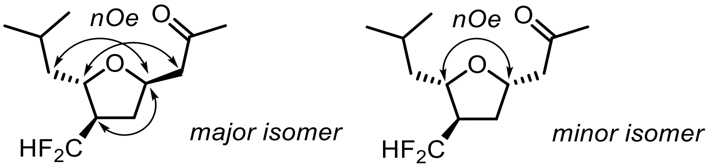

(4*R*,5*R*)-5-((Benzyloxy)methyl)-4-(difluoromethyl)tetrahydrofuran-2-yl acetate (31). Alcohol **19o** (24.0 mg, 0.07 mmol) was dissolved in dichloromethane (1 mL) and the resulting solution was cooled to –78 °C. Ozone was bubbled through the solution until a deep blue color persisted. The mixture was purged with argon until the blue color had completely disappeared. Triphenylphosphine (22.0 mg, 0.08 mmol) was then added and the mixture allowed to reach room temperature. After stirring for 30 min, the mixture was concentrated in *vacuo*.

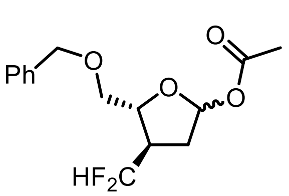

The residue was dissolved in hexane/EtOAc (1:1, 2 mL) and the mixture filtered through a plug of silica. The plug was rinsed with an additional 4 mL of the same solvent mixture and the combined filtrates were again concentrated. The residue was dissolved in dichloromethane (1 mL). Pyridine (12.4 µL, 0.15 mmol), DMAP (0.5 mol%) and acetic anhydride (13.2 µL, 13.9 mmol) were added and the solution was stirred for 1 h at room temperature. The reaction was quenched with water (1 mL) and the organic phase was washed with aq. HCl (1 M, 1 mL), sat. aq. $NaHCO_3$ (1 mL) and dried over Mg_2SO_4. The solution was concentrated in *vacuo* and the residue purified by automated flash chromatography (10 g column, 5-40 % gradient of cyclohexane/ethyl acetate), affording the title compound as a colorless oil (16.9 mg, 81 % yield over 2 steps, dr ≈ 1:1).

Note: *since the diastereomeric ratio is 1:1, the signals of both compounds are listed below and annotated A and B*. ^{1}H NMR (400 MHz, $CDCl_3$, 298 K): δ [ppm] = 7.32 (m, 10H, A,B), 6.38 (dt, *J* = 5.5, 1.1 Hz, 1H, A), 6.31 (dd, *J* = 4.1, 1.6 Hz, 1H, B), 5.89 (td, *J* = 56.9, 6.7 Hz, 1H, A), 5.87 (td, *J* = 56.3, 4.2 Hz, 1H, B), 4.61 – 4.53 (m, 4H, A,B), 4.36 (q, *J* = 4.4 Hz, 1H, A), 4.30 (dt, *J* = 7.8, 5.3 Hz, 1H, B), 3.68 – 3.54 (m, 4H, A,B), 2.89 – 2.78 (m, 1H, B), 2.77 – 2.67 (m, 1H, A), 2.42 (dddd, *J* = 14.4, 10.5, 5.3, 1.6 Hz, 1H, A), 2.24 – 2.16 (m, 2H, B), 2.10 – 2.06 (m, 1H, A), 2.06 (s, 3H, B), 1.95 (s, 3H, A). ^{13}C NMR (101 MHz, $CDCl_3$, 298 K): δ [ppm] = 170.2 (A), 170.1 (B), 138.1 (A), 138.0 (B), 128.6 (B), 128.6 (A), 127.9 (A), 127.9 (B), 127.8 (A), 127.7 (B), 119.2 – 115.4 (m, B), 118.4 – 114.6 (m, A), 98.7 (A), 98.4 (B), 79.5 (t, *J* = 4.5 Hz, B), 79.3 (dd, *J* = 6.8, 2.7 Hz, A), 73.8 (A), 73.5 (B), 72.3 (B), 71.0 (A), 43.7 (t, *J* = 21.3 Hz, A), 43.6 (t, *J* = 21.3 Hz, B), 33.1 – 33.0 (m, A), 32.70 (t, *J* = 4.2 Hz, B), 21.4 (B), 21.4 (A). ^{19}F NMR (282 MHz, $CDCl_3$, 298 K): δ [ppm] = –118.0 (d, *J* = 284.2 Hz, A), –118.6 (d, *J* = 284.4 Hz, B), –119.4 (d, *J* = 284.2 Hz, A), –121.9 (d, *J* = 284.5 Hz, B). IR (ATR): ṽ = 2924, 2864, 1739, 1453, 1365, 1232, 1096, 1029, 1008, 1029, 1008, 973, 934, 858, 737, 699, 605, 513 cm^{-1}. HRMS (ESI$^+$): *m/z*: calcd. for $[C_{15}H_{18}F_2O_4Na]^+$ $[M+Na]^+$: 323.1065, found: 323.1063.

(4*R*,5*S*)-5-Butyl-4-(difluoromethyl)dihydrofuran-2(3*H*)-one (30). A solution of alcohol **19b** (14.8 mg, 0.053 mmol) in dichloromethane (0.6 mL) was cooled to –78 °C. Ozone was bubbled through the solution until a blue color persisted. The mixture was purged with argon until the blue color had completely disappeared. Triphenylphosphine (16.7 mg, 0.064 mmol) was added and the mixture was allowed to reach ambient temperature. After stirring for 30 min, the mixture was concentrated under *vacuo*.

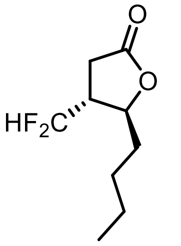

The residue was dissolved in hexane/EtOAc (1:1, 2 mL) and the somewhat turbid solution filtered through a plug of silica, which was carefully rinsed with the same solvent mixture (4 mL). The combined filtrates were evaporated and the residue as dissolved in dichloromethane (0.5 mL). This solution was slowly added to a suspension of pyridinium chlorochromate (15.8 mg, 0.073 mmol) and Celite (30 mg) in CH_2Cl_2 (0.5 mL) and the resulting mixture stirred at room temperature for 6 h. The suspension was concentrated under *vacuo* and the title compound was obtained after purification of the residue by automated flash chromatography (10 g column, 5-40 % gradient of cyclohexane/ethyl acetate) as a colorless oil (7.4 mg, 72 % yield over 2 steps).

$[\alpha]_D^{20}$ = –15.0 (c = 1.1, CH_2Cl_2). ^{1}H NMR (400 MHz, $CDCl_3$, 298 K): δ [ppm] = 5.85 (td, *J* = 55.8, 3.7 Hz, 1H), 4.57 – 4.47 (m, 1H), 2.78 – 2.57 (m, 3H), 1.75 – 1.68 (m, 2H), 1.51 – 1.33 (m, 4H), 0.92 (t, *J* = 7.2 Hz, 3H). ^{13}C NMR (101 MHz, $CDCl_3$, 298 K): δ [ppm] = 174.3, 115.5 (t, *J* = 242.9 Hz), 79.1 (t, *J* = 4.6 Hz), 44.3 (t, *J* = 21.5 Hz), 35.4, 28.6 (t, *J* = 4.8 Hz), 27.3, 22.4, 14.0. ^{19}F NMR (282 MHz, $CDCl_3$, 298 K): δ [ppm] = –122.6 (d, *J* = 11.2 Hz). IR (ATR): ṽ = 2957, 2924, 2855, 1776, 1463, 1425, 1379, 1361, 1260, 1179, 1110, 1044, 973, 927, 800, 685 cm^{-1}. HRMS (ESI$^+$): *m/z*: calcd. for $[C_9H_{14}F_2O_2Na]^+$ $[M+Na]^+$: 215.0854, found: 215.0855.

Non-linear Effect

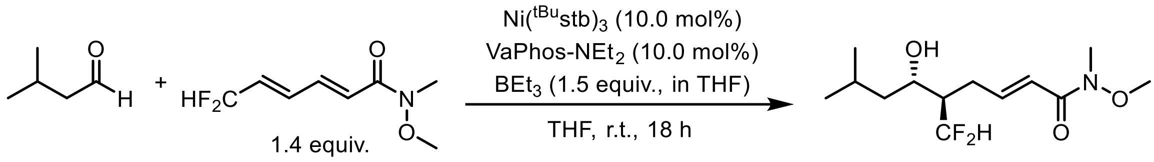

The reactions to determine the non-linear effect were carried out according to general procedure **B** using 3-methylbutanal (21.5 µL, 0.20 mmol), difluorosorbamide **15** (53.5 mg, 0.28 mmol) and ligand (*R*)-**L1** of different optical purity as indicated below. All reaction mixtures seemed to be homogeneous solutions without any visible precipitates. In all cases, complete conversion of the substrate was observed. The crude material (rr > 20:1) was purified by automated flash chromatography (10 g column, 5–60 % gradient of cyclohexane/ethyl acetate); the isolated yields were invariably >90%. The ee was determined by HPLC analysis (Chiralpak 150 mm IG-3, 3 µm, 4.6 mm ∅, CH_3CN/H_2O = 40:60, v = 1.0 mL/min, λ = 215 nm) for each reaction.

For each selected optical purity of (*R*)-**L1**, the corresponding measurement was performed in triplicate (indicated [1/2/3]). Error bars indicate ±1 standard deviation.

ee_{cat} = 0%, ee_{prod} = 0%
ee_{cat} = 20%, ee_{prod} = 12.8%[1], 12.9%[2], 11.5%[3]
ee_{cat} = 40%, ee_{prod} = 24.8%[1], 27.7%[2], 26.7%[3]
ee_{cat} = 60%, ee_{prod} = 42.1%[1], 44.5%[2], 47.1%[3]
ee_{cat} = 80%, ee_{prod} = 64.2%[1], 66.8%[2], 64.0%[3]
ee_{cat} = 98%, ee_{prod} = 95%

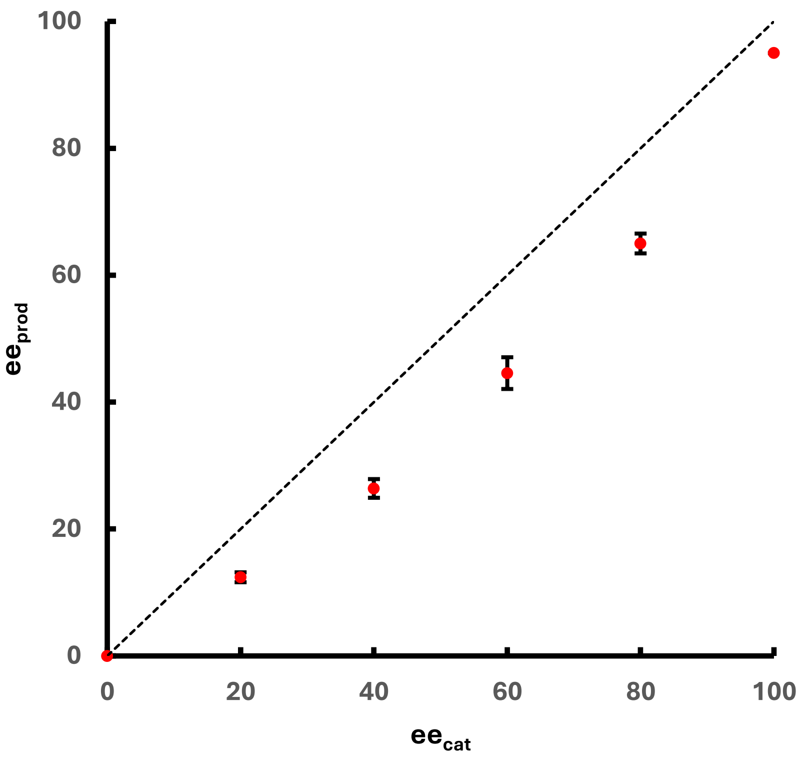

Figure S13. (–)-Non linear effect in the nickel catalyzed reductive coupling of 3-methylbutenal and diene **15** in the presence of the VAPOL-derived phosphoramidite ligand (*R*)-**L1** of different optical purity.

In additional control experiments, the ratio of **L1**:Ni(0) was changed from 1:1 to 2:1 using **L1** of two different optical purities; once again, a significant (–)-non linear effect was observed. Under the premise that the homochiral complex of type (*R,R*)-**M** is energetically (much) more stable than its heterochiral analogue (*S,R*)-**M**, a higher concentration of **L1** will favor the formation of (*R,R*)-**M** acting as reservoir and hence lower the optical purity of the ligand passing through the catalytic cycle. In line with this notion, the ee's of the resulting products were lower than those obtained with a **L1**:Ni(0) ratio of 1:1.

ee of L1	ee of product	
	L1:Ni(0) = 1:1	L1:Ni(0) = 2:1
40%	26.4%	20.9%
98%	95.0%	89.5%

Crystallization of the Nickel 1,3-Diene Complexes

[Ni(cod)$_2$] was chosen instead of [Ni(*t*Bu-stb)$_3$] because the substituted stilbene ligands of the latter are the least soluble component and crystallize before any complex could be obtained. In the NMR studies, they obscure the aromatic/olefinic region of the spectra and hence render the interpretation even more challenging.

Complex 32. [Ni(cod)$_2$] (218.0 mg, 0.79 mmol) and methyl (2*E*,4*E*)-hexa-2,4-dienoatein (**3**) (103.3 µL, 0.79 mmol) were dissolved in 2 mL of toluene at room temperature to give a dark red solution. The mixture was stirred for 2 h followed by filtration under argon. A single crystal for X-ray diffraction analysis was prepared by recrystallization from toluene at −60 °C.

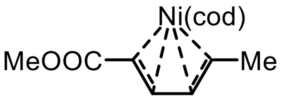

Complex 33. [Ni(cod)$_2$] (55.0 mg, 0.20 mmol), PCy$_3$ (112.2 mg, 0.40 mmol) and methyl (2*E*,4*E*)-hexa-2,4-dienoatein (**3**) (26.1 µL, 0.2 mmol) were dissolved in 5 mL of THF at room temperature to give a dark red solution. The mixture was stirred for 2 h followed by filtration under argon and concentration under high vacuum (10^{-3} mbar) to give a viscous dark red oil. A single crystal for X-ray diffraction analysis was prepared by recrystallization from pentane at −40 °C.

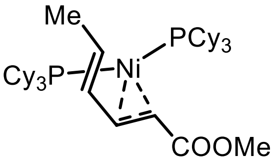

For the characterization by NMR, see the following Section.

Stoichiometric NMR-Experiments

Complex 33. A flame-dried Schlenk flask was charged with [Ni(cod)$_2$] (13.8 mg, 0.05 mmol), PCy$_3$ (28.0 mg, 0.1 mmol) and methyl sorbate (**3**) (6.5 µL, 0.05 mmol). The Schlenk flask was evacuated and refilled with argon (3 cycles). [D$_8$]-THF (1.0 mL) was added and the resulting deep red solution was stirred for 1 h before the solution was canula-filtered and transferred to a J-Young NMR tube.

The experiment described above yields almost exclusively one species in solution, besides traces of oxidized phosphine ligand and uncoordinated ligand and diene. The observed species is a nickel complex to which we assign structure **33**; it is comprised of two *cis*-bound PCy$_3$-ligands and one methyl sorbate diene. The diene is coordinated via the double bond vicinal to the carbonyl group (Figure S15-S17). This matches the obtained X-ray structure of complex **33**.

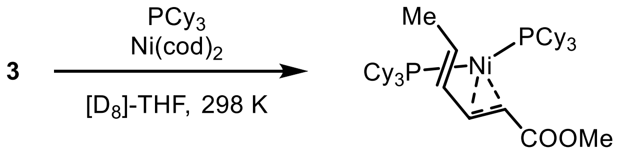

33

- The $^{31}P\{^{1}H\}$-NMR shows a defined species featuring a doublet (*J* = 36.2 Hz) at 31.4 ppm and another doublet (*J* = 36.2 Hz) at 30.7 ppm (Figure S14). The signals correspond to one ligand each. The $^{3}J_{PP}$ coupling (*J* = 36.2 Hz) between the two ^{31}P nuclei suggests that the two ligands occupy a *cis* arrangement.[6]

- The coordination of the diene *via* the double bond vicinal to the carbonyl group was deducted from the observed shifts of the corresponding ^{1}H/^{13}C signals (3.27 ppm and 2.39 ppm in the ^{1}H NMR and 50.5 ppm and 44.5 ppm in the ^{13}C NMR spectrum). A 1D selective

TOCSY NMR spectrum exciting the proton in the 4-position of the diene was conducted to extract the chemical shifts from the crowded regions (Figure S16). The $^{3}J_{H4,H5}$ coupling constant is closer to an aliphatic *trans* coupling (9 Hz), which also indicates substantial loss of double bond character upon binding to the Ni center.

- The sample was characterized at 283 K due to significant broadening of the ^{1}H, ^{13}C and ^{31}P signals at temperatures above and below (Figure S18). 2D ^{31}P-^{31}P-EXSY spectra indicate that the line broadening in the ^{31}P NMR spectrum at room temperature is due to *intramolecular* exchange between the two phosphine ligands (potentially via a Berry-pseudorotation mechanism) (Figure S19).

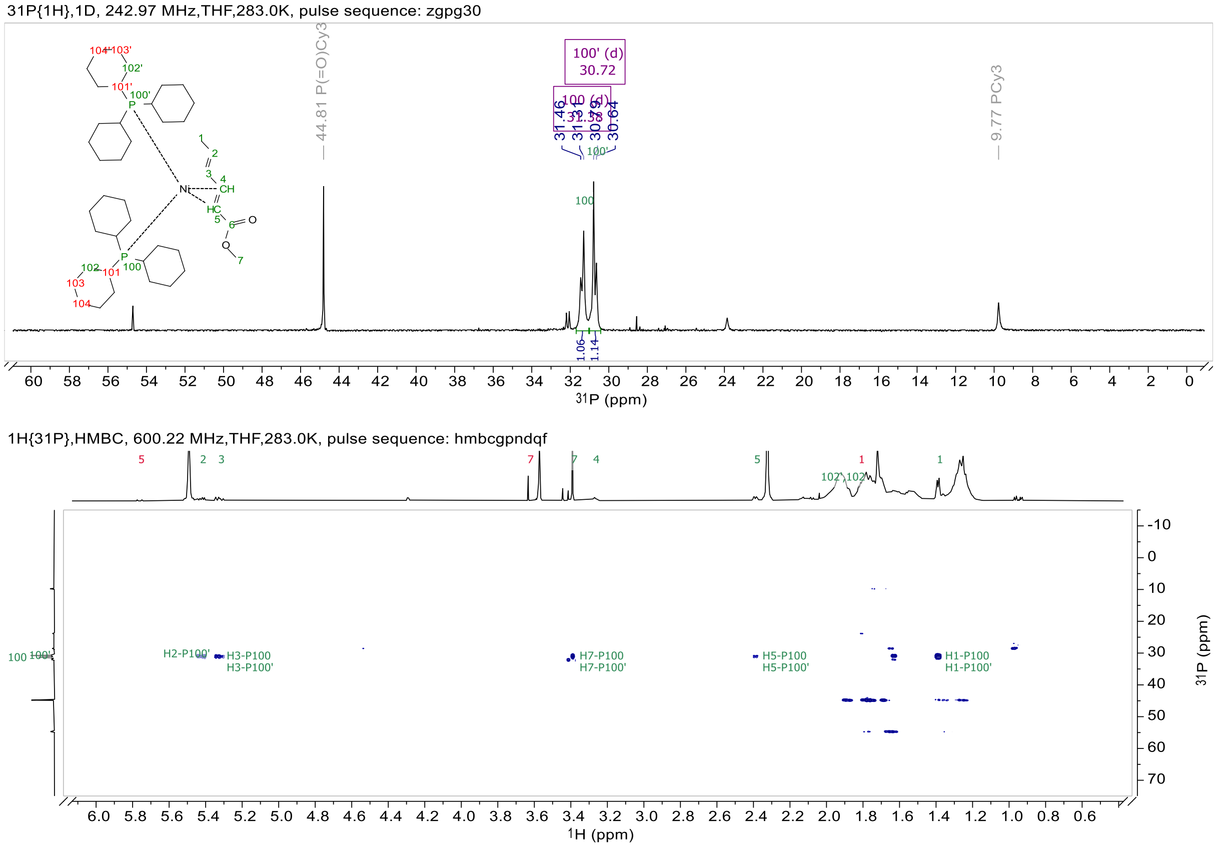

Figure S14. Top: $^{31}P\{^{1}H\}$ NMR spectrum showing the nickel complex **33** comprised of two PCy_3 ligands and one diene **3**, together with uncoordinated and some oxidized ligand; bottom: $^{1}H\{^{31}P\}$ HMBC NMR spectrum.

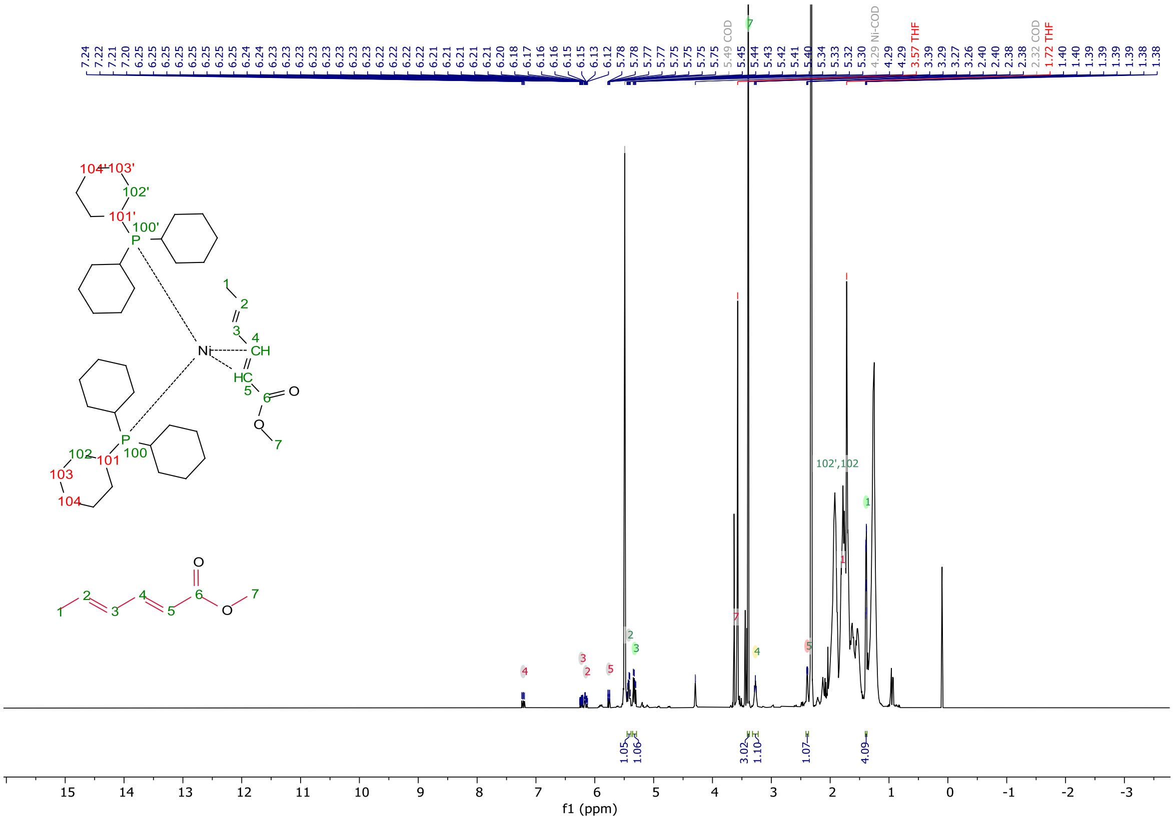

Figure S15. ^{1}H NMR spectrum showing the nickel complex **33**; the inserted numbers show characteristic signals.

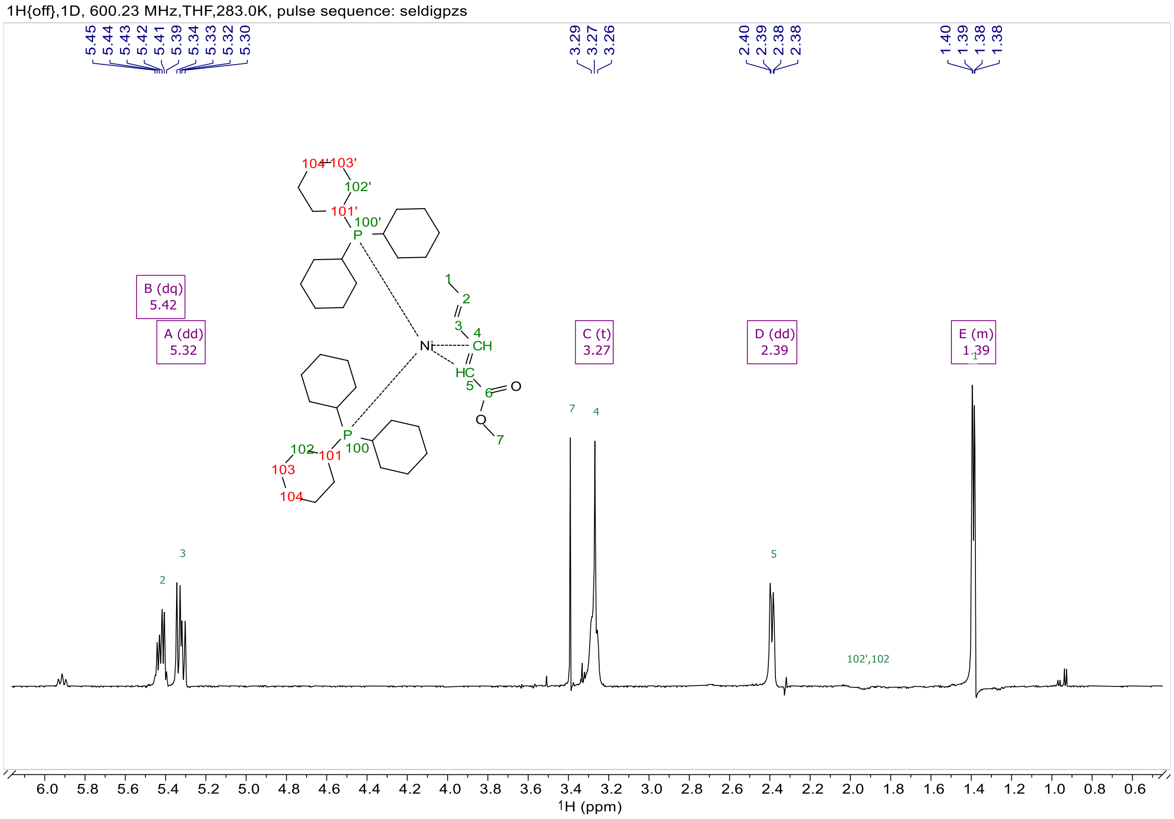

Figure S16. 1D selective TOCSY NMR spectrum obtained after excitation of the proton at the C4 position of the ligated diene **3,** allowing the olefinic signals to be isolated and assigned.

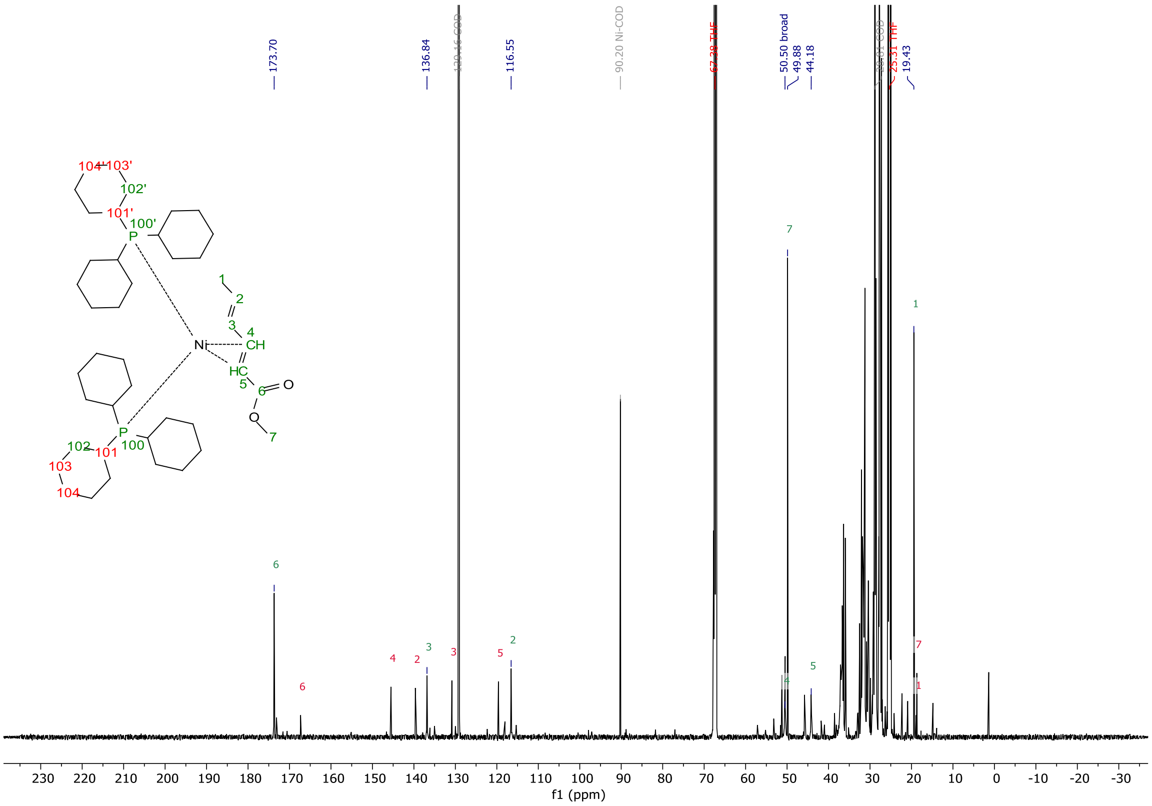

Figure S17. $^{13}C\{^1H\}$ NMR spectrum showing the nickel complex **33**; the inserted numbers show characteristic signals.

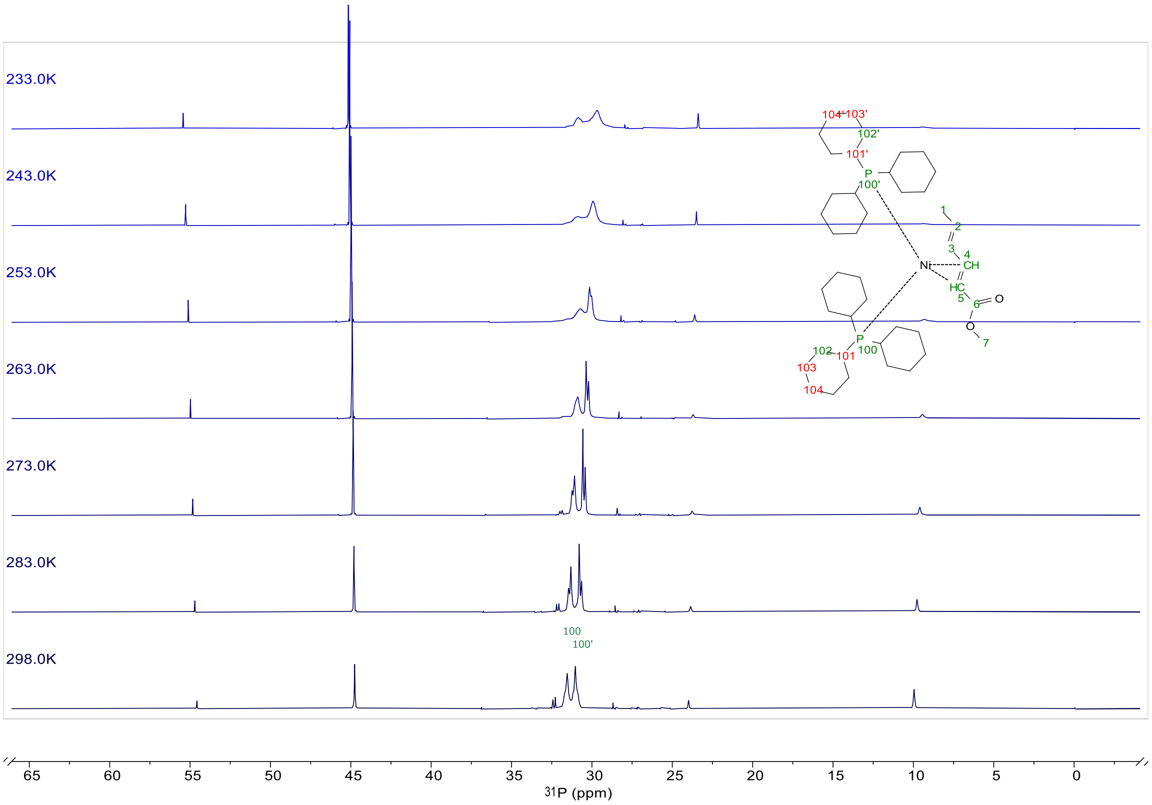

Figure S18. Variable temperature $^{31}P\{^1H\}$ NMR experiments highlighting 283 K as optimum temperature for obtaining defined signals

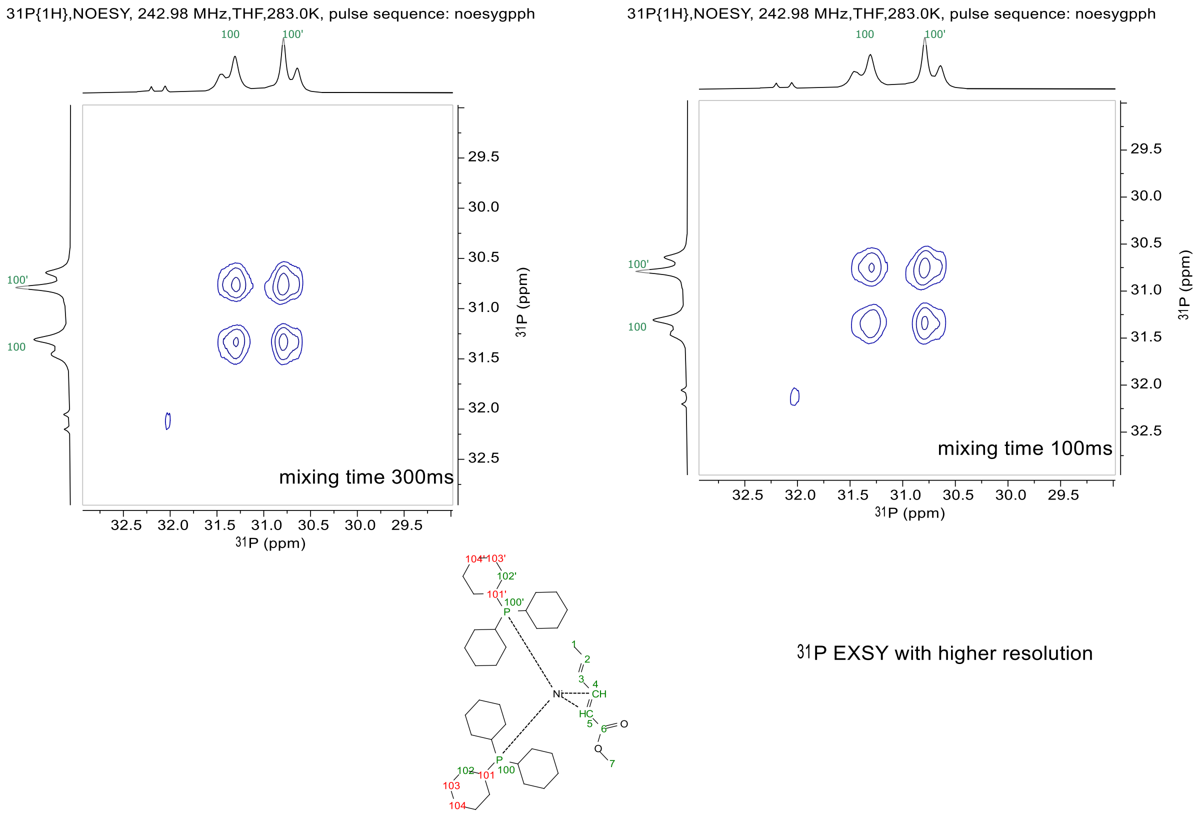

Figure S19. ^{31}P-^{31}P-EXSY spectra of complex **33** indicating intramolecular ligand exchange between the two phosphine ligands

Complex 34. A flame-dried Schlenk flask was charged with tris(*trans*-1,2-bis(4-*tert*-butylphenyl)ethene) nickel(0) (Ni(*t*Bu-stb)$_3$, **16**) (23.4 mg, 0.025 mmol), phosphoramidite (*R*)-**L1** (16.0 mg, 0.025 mmol, 98% ee) and trifluorinated diene **12** (0.05 mmol, 10.5 mg). The Schlenk flask was evacuated and refilled with argon (3 cycles). [D$_8$]-THF (1.0 mL) was added and the resulting deep red solution was stirred for 1 h before the solution was filtered and transferred to a J-Young NMR tube.

Inspection of the solution by MS (ESI+) shows a signal at *m/z* = 1336.40, corresponding to [**L1**$_2$Ni(0)] (*calcd.* for $C_{88}H_{68}N_2NiO_4P_2$: 1336.40).

The experiment described above yielded almost exclusively a single species in solution, besides traces of oxidized phosphoramidite ligand and uncoordinated ligand (Figues S14, S15, S17). The observed species is a nickel complex to which we assign structure **34**; it is comprised of two *cis*-bound VAPOL-ligands **L1** and one CF_3-subsituted diene. The diene is coordinated via the double bond vicinal to the CF_3-group.

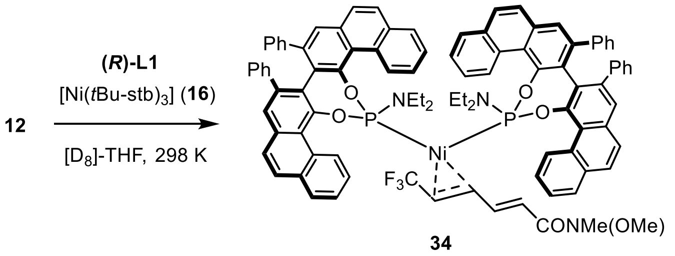

- The $^{31}P\{^1H\}$-NMR shows a defined species consisting of a dq (*J* = 36.9, 12.5 Hz) at 160.5 ppm and a d (*J* = 36.9 Hz) at 160.1 ppm (Figure S20). The signals correspond to one ligand each. The signal at 160.5 ppm shows a complex splitting pattern which is likely due to through-space coupling of the P-atom with the CF_3-group rather than a ^4J coupling through the bonds.[7] A high J_{PF} coupling constant of 12.5 Hz for the signal at 160.5 ppm and a cross-peak in the ^{19}F-^{31}P-HMBC (Figure S24) can be observed, further supporting this assignment.

- The coordination of the diene *via* the double bond vicinal to the CF_3-group was deducted by the observed shift of the corresponding $^1H/^{13}C$ signals compared to the uncoordinated diene (signals at 2.4 ppm and 1.3 ppm in the ^1H and 54.4 ppm and 49.0 ppm in the ^{13}C NMR spectrum). A 1D selective TOCSY NMR spectrum exciting the proton in the 3-position of the diene was conducted to extract the chemical shifts from the crowded regions (Figure S22).

- A series of ^{19}F and ^{31}P NMR spectra with varying $^1H/^{19}F/^{31}P$-decoupling were recorded, delivering further proof for the interaction of the CF_3-group with one of the phosphoramidite ligands (Figure S25).

- The corresponding stoichiometric experiment using *rac*-**L1** resulted in an identical spectra. This shows that the homochiral complexes (*R,R*)-**34** and (*S,S*)-**34** are thermodynamically much more stable than the corresponding heterochiral complex (*R,S*)-**34** in solution. This conclusion is in excellent accord with the results of the DFT study.

- Addition of benzaldehyde to the solution comprising (*R,R*)-**34** did not visibly change the spectrum. Therefore, replacement of an **L1** ligand from the nickel center by the aldehyde evidently faces a substantial energetic barrier. Once again, this is perfectly in line with the computational results.

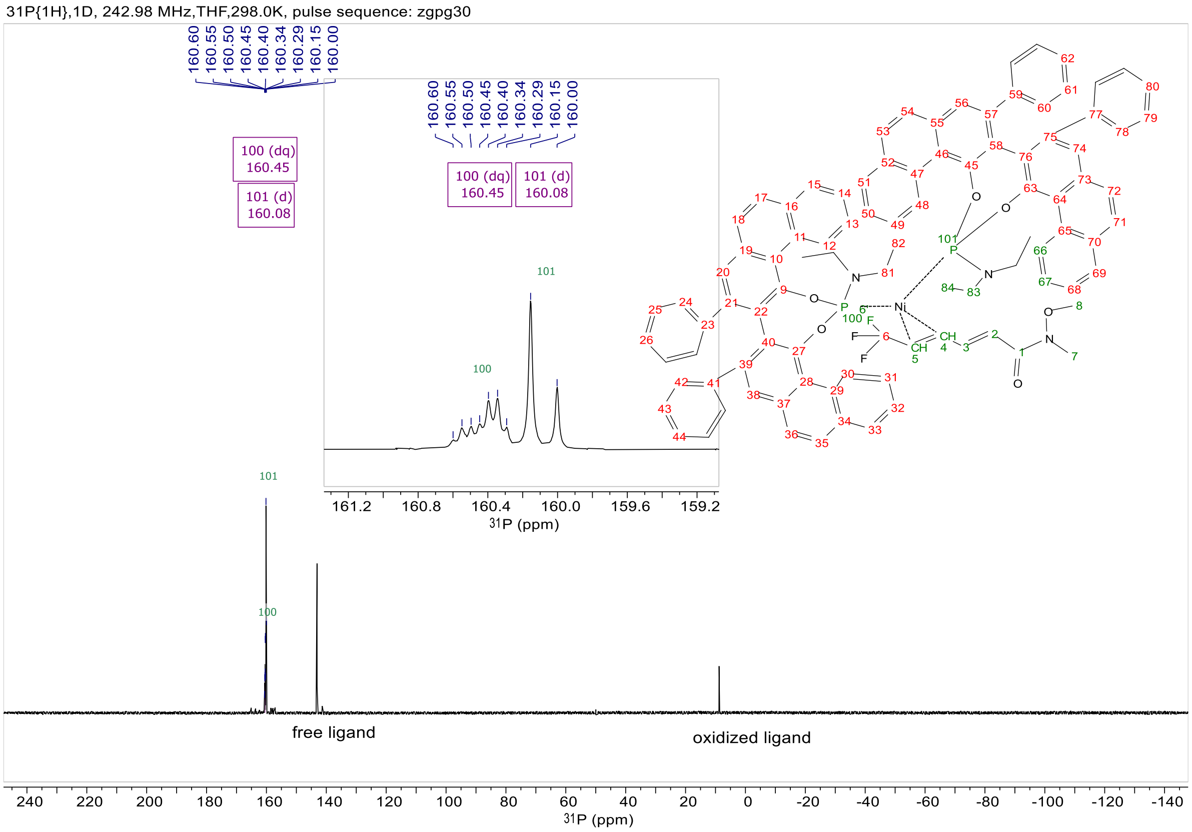

Figure S20. $^{31}P\{^1H\}$ NMR spectrum showing the nickel complex **34** comprised of two VAPOL-derived phosphoramidite ligands (*R*)-**L1** and one CF_3-diene **12**, together with uncoordinated and some oxidized ligand (*R*)-**L1**.

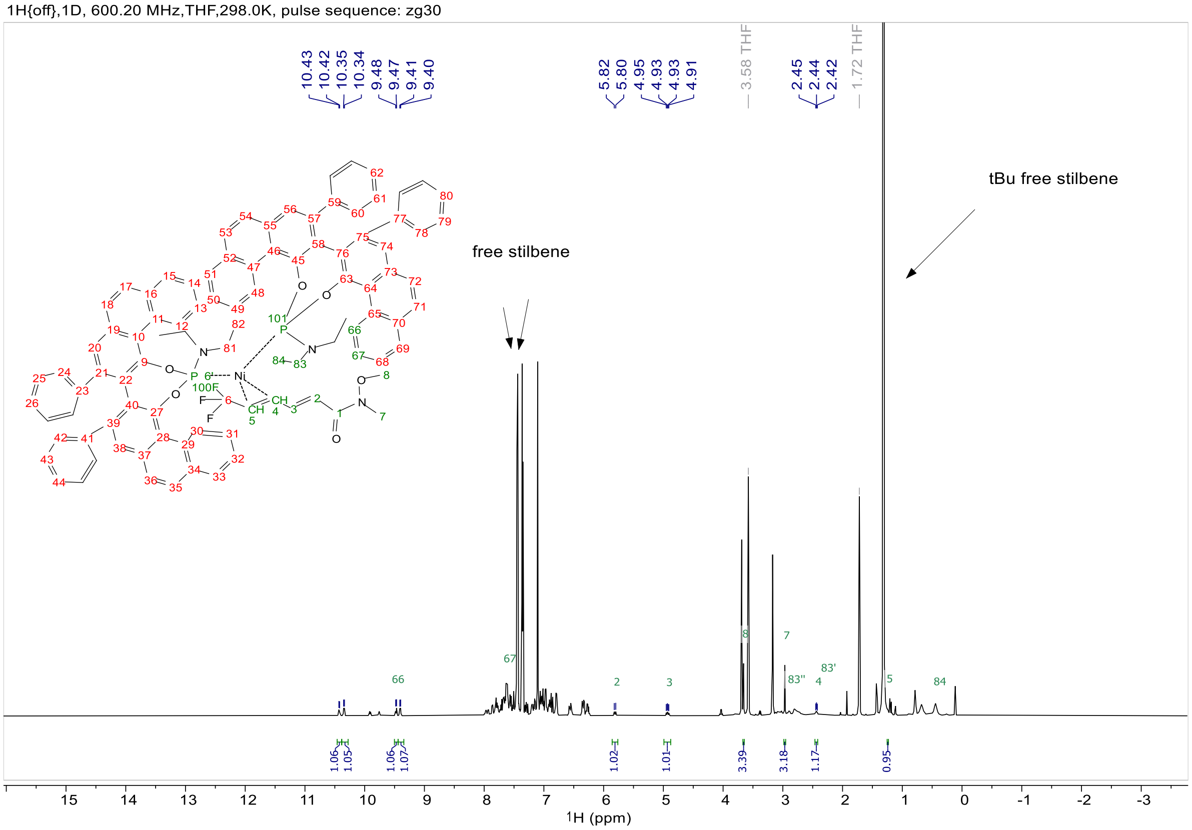

Figure S21. ^1H NMR spectrum showing of the homochiral nickel complex **34** described above; the inserted numbers show characteristic signals.

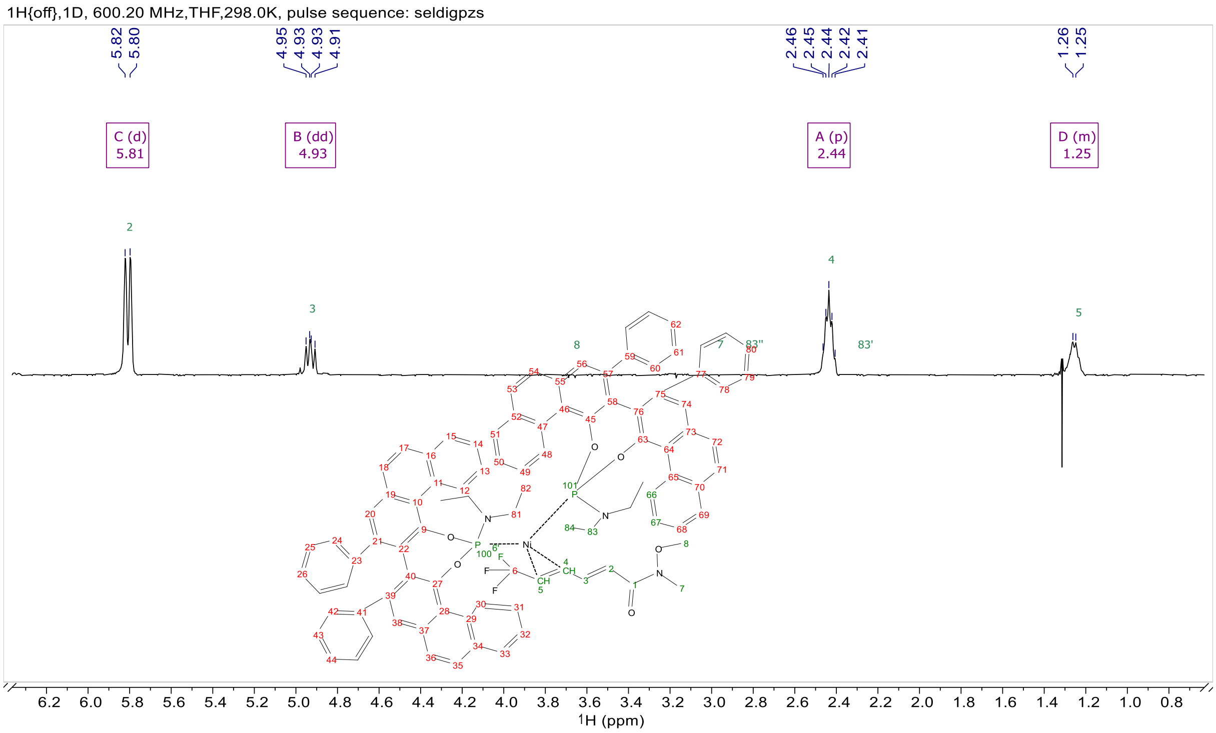

Figure S22. 1D selective TOCSY NMR spectrum obtained after excitation of the proton at the C3 position of the ligated diene in **34**, allowing the olefinic signals to be isolated and assigned.

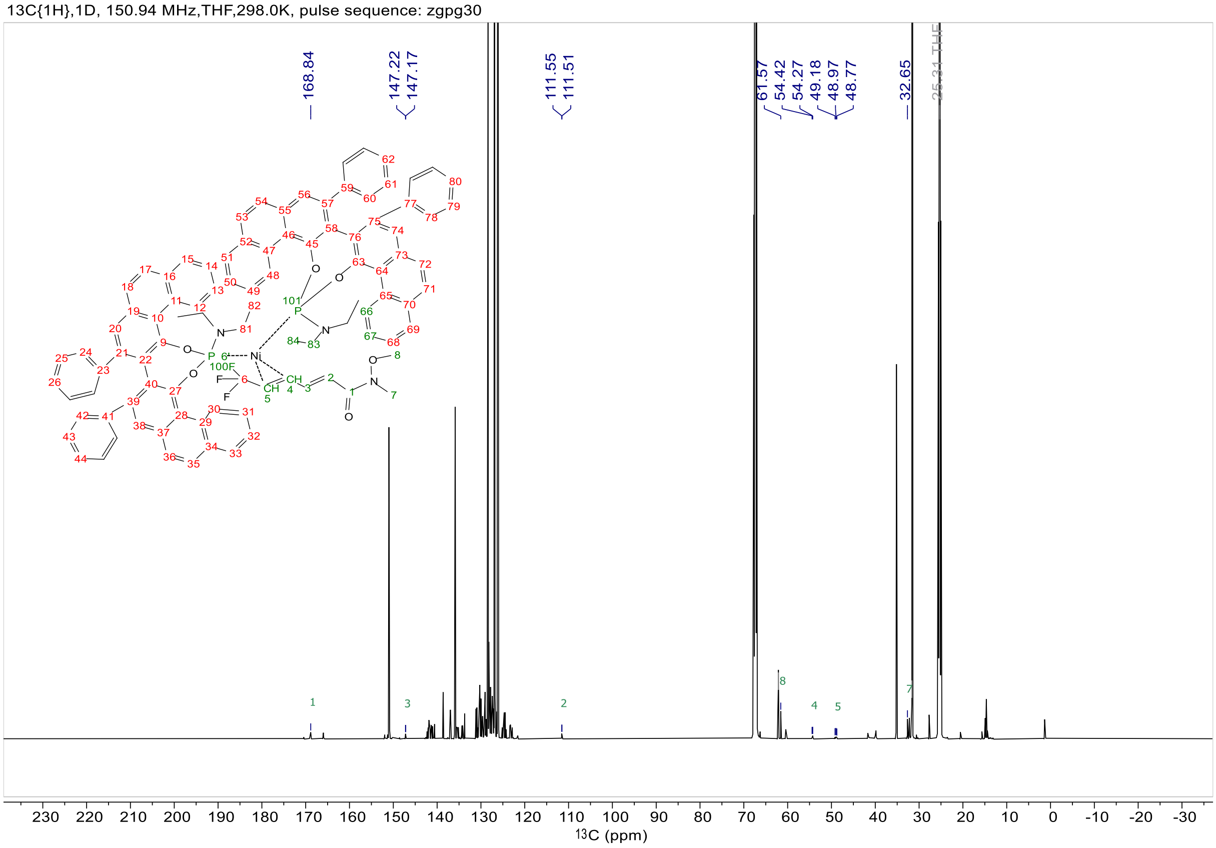

Figure S23. $^{13}C\{^{1}H\}$ NMR spectrum showing of the homochiral nickel complex **34** described above; the inserted numbers show characteristic signals.

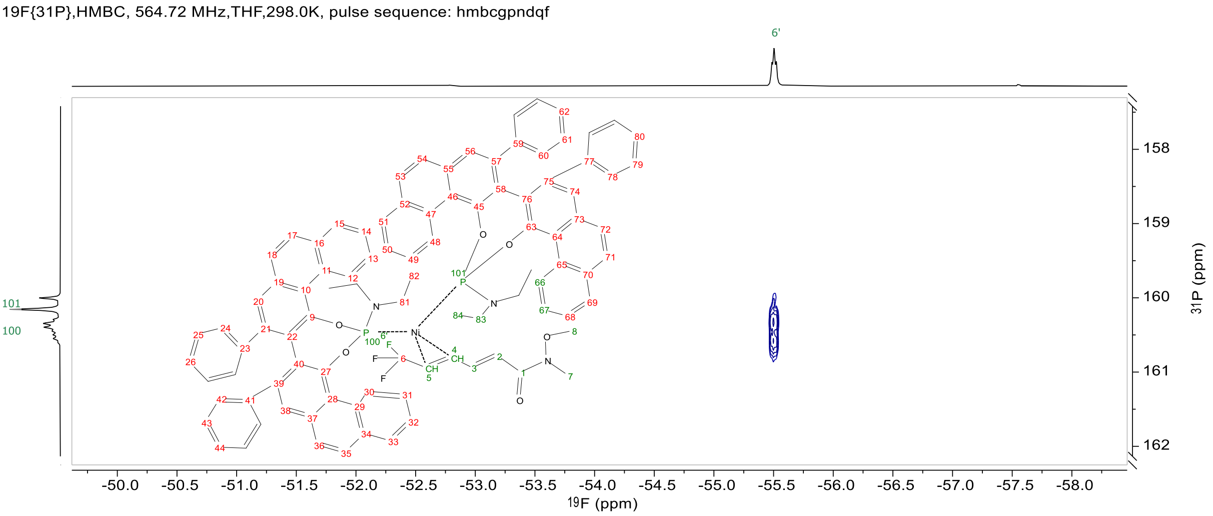

Figure S24. ^{19}F-^{31}P HMBC NMR spectrum of complex **34** showing a cross peak between the P-atom of one of the phosphoramidite ligands and the CF_3-substituent of the diene.

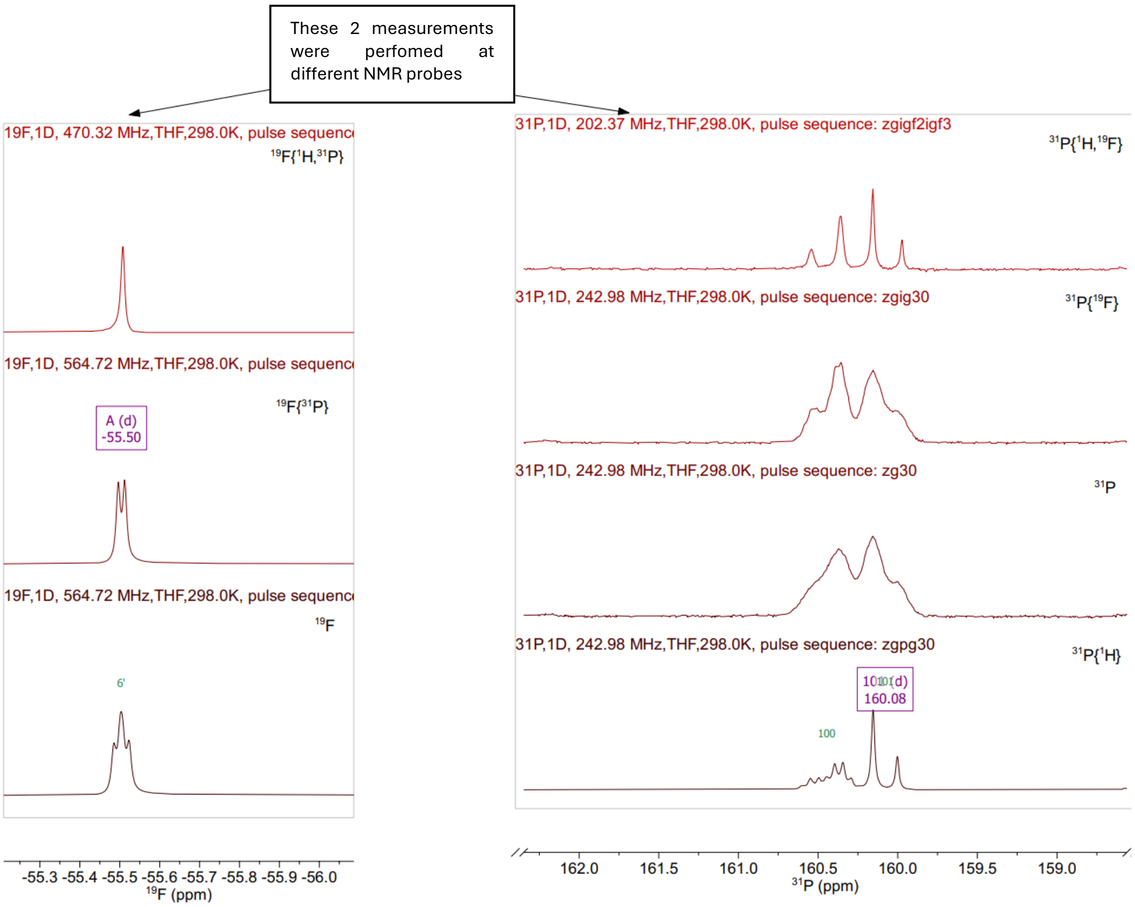

Figure S25. Series of ^{19}F and ^{31}P decoupling experiments proving the coupling between the CF_3-substituent and the P-atoms of the phosphoramidite ligands of complex **34**

Computational Study

Computational Details. All calculations were performed using the ORCA quantum chemistry package (stable versions 6.0 and 6.1).[8] The conformational space of all key intermediates as well as all transition states leading to the *anti*-isomers, was explored with the CREST program[9] using the GFN2-xTB[10] and GFN-FF[11] methods. The resulting conformer ensembles were filtered stepwise based on relative energy and RMSD. At each step, among the structures differing by at least 2.0 Å RMSD from the current reference, the lowest-energy conformer was selected for DFT re-optimization and then used as the new reference. For all stationary points, the geometry was optimized at the PBE-D4/def2-SVP/CPCM(toluene) level.[12,13,14,15] Frequency calculations were used to confirm the nature of stationary points and obtain thermochemical corrections at 298.15 K. Electronic energies were refined at the WB97X-D4rev/def2-QZVP/CPCM(toluene)[16] level using the RIJCOSX approximation.[17]

Calibration Study. At the PBE-D4/def2-SVP/CPCM(toluene) level of theory, an excellent agreement was obtained between the computed and the experimental Ni–C bond lengths (X-ray structure analysis) of complex **32**. This level of theory was therefore used for the study of the reaction coordinates leading to the different regio- and stereoisomers that can be formed upon coupling of trifluorosorbamide **12** and benzaldehyde (Scheme S1).

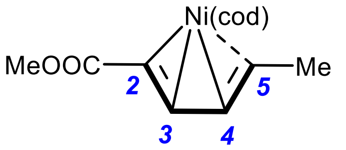

32

	C2-Ni (Å)	C3-Ni (Å)	C4-Ni (Å)	C5-Ni (Å)	RMSD
Experimental	2.1442	2.0099	2.072	2.211	0.000
PBE-D4/def2-SVP	2.1485	2.015	2.062	2.198	0.009

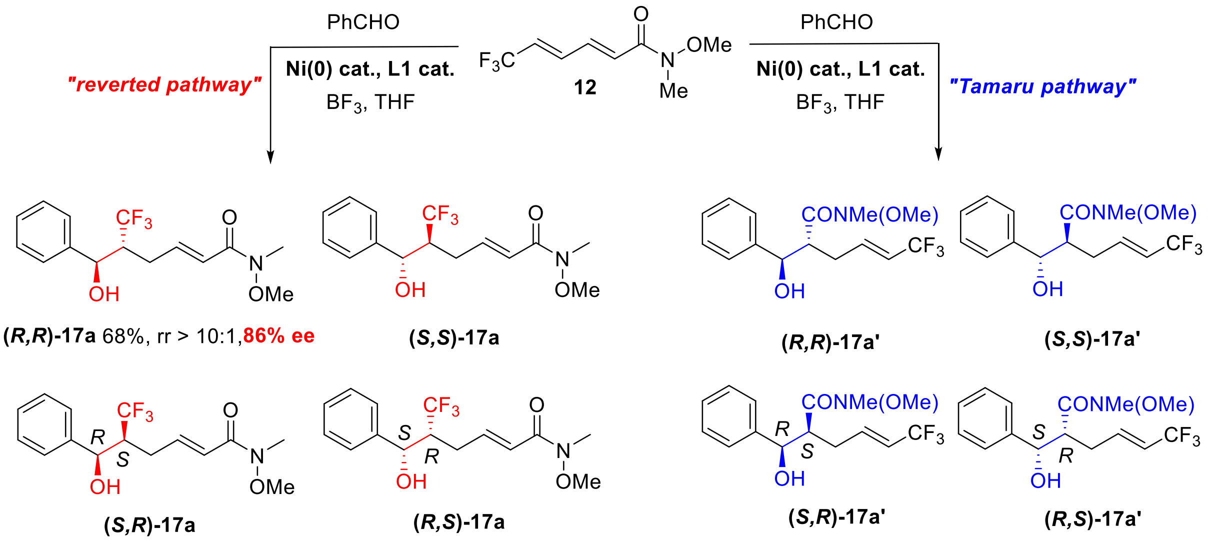

Scheme S1. Full set of possible reaction pathways for the Ni-catalyzed coupling of trifluorinated sorbamide **12** and benzaldehyde

Reaction Pathways. For both the "Tamaru" and the "reverted" pathway, the *anti*-configured transition states (TSs) are consistently lower in energy than the *syn* counterparts. Among all of them, the pathway leading to (*R*,*R*)-**17a** has the lowest barrier. This matches the experimental result, thus confirming the reliability of the computed selectivity.

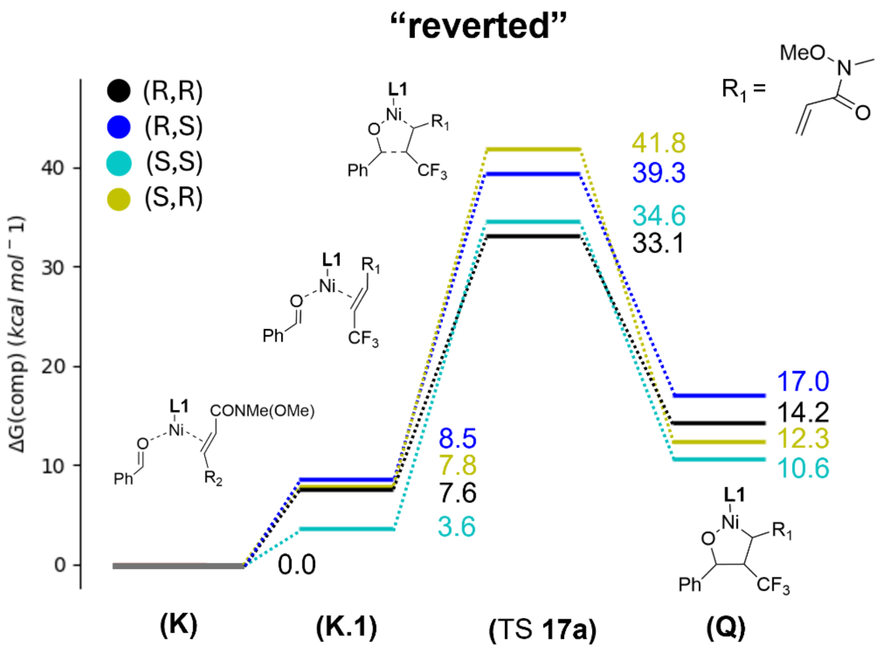

Figure S26. Pathways leading to the different stereomers of the „inverted" coupling product **17a**

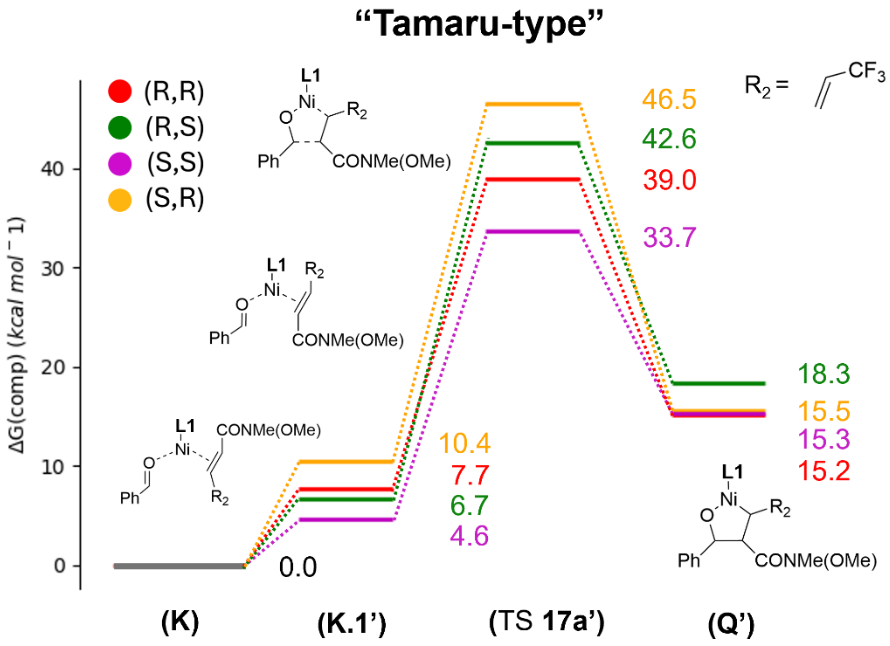

Figure S27. Pathways leading to the different stereomers of the „Tamaru-type" coupling product **17a'**

To investigate the dependence of our results on the nature of the exchange-correlation functional in more detail, we recalculated the energies of the rate-determining transition states using several combinations of functionals and basis sets. For this analysis, we did not repeat the full conformational sampling; rather, the lowest-energy structure previously identified within the established conformational ensemble were used. Note that the DFT reoptimizations after the conformational sampling were carried out at the PBE-D4/def2-SVP level and the nature of the most stable conformer does not change upon single point refinement with WB97X-D4rev/def2-QZVP.

	*ΔG ((R,R)-**17a**•BEt_3)*	*ΔG ((S,S)-**17a**•BEt_3)*
PBE0-D4/def2-QZVP//PBE-D4/def2-SVP	31.5 kcal/mol	33.4 kcal/mol
WB97X-D4rev/def2-QZVP//PBE-D4/def2-SVP	31.8 kcal/mol	33.4 kcal/mol
PBE-D4/def2-QZVP//PBE-D4/def2-SVP	37.0 kcal/mol	38.2 kcal/mol
PBE-D4/def2-QZVP//PBE-D4/def2-QZVP	36.8 kcal/mol	38.5 kcal/mol

It is interesting to note that the computed energy barriers do not decrease when using simpler GGA functionals. On the contrary, the PBE functional yields the highest barriers among those tested. Importantly, in all cases the energy difference between the competing pathways remains fairly constant across the different levels of theory. This indicates that the predicted stereoselectivity is robust and not an artifact of the specific choice of functional or basis set, supporting the reliability of the stereochemical conclusions drawn in this work.

Effect of BEt_3. We also investigated the potential impact of BEt_3 on the barrier of the enantio-selectivity-determining step. To this end, the two lowest-energy TSs within the *anti* manifold were computed with BEt_3 explicitly included prior to metallacycle formation, *i.e.* the pathways leading to ***(R,R)*-17a** and ***(S,S)*-17a**, respectively. Ligation of BEt_3 to benzaldehyde resulted in a small but significant barrier-lowering but left the computed selectivity unchanged. The favorable substrate/**L1** contact that stabilizes the TS leading to the experimentally observed “reverted” product ***(R,R)*-17a** is hence fully preserved upon inclusion of the Lewis acid.

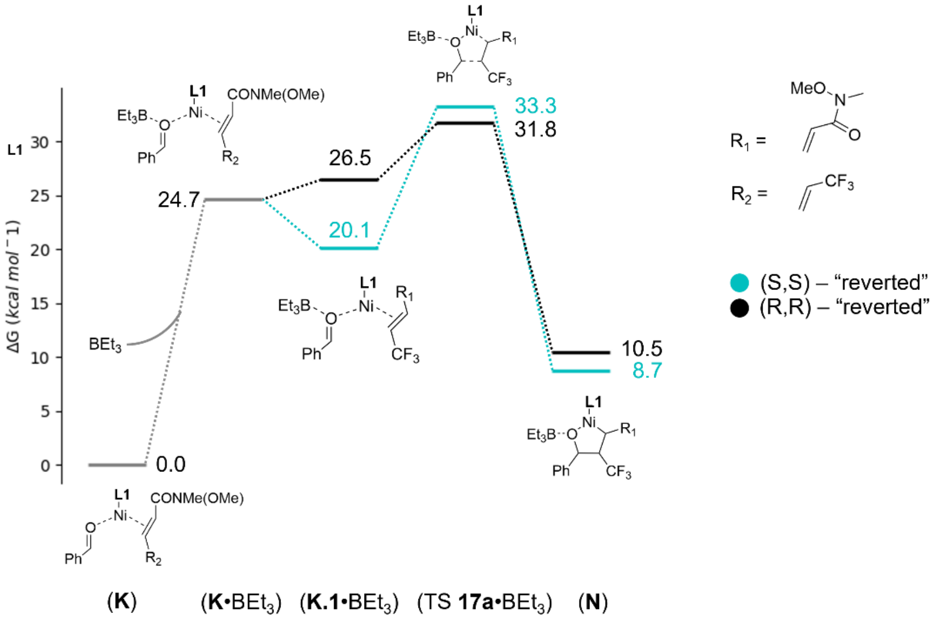

Figure S28. Pathways leading to the enantiomers of the „inverted“ coupling product **17a** with BEt_3 explicitly included prior to metallacycle formation

Ligand/Substrate Interactions and Their Impact of Selectivity. In the favored “reverted” pathway leading to (*R,R*)-**17a**, **L1** stabilizes the TS through extended π-π and σ-π contacts with the chain of the 1,3-diene substrate, as highlighted in blue Figures S29 and S30. In the analogous pathway leading to the enantiomeric product (*S,S*)-**17a**, this interaction is markedly reduced.

In the “Tamaru-type” pathways, the diene chain is oriented away from **L1**, removing any meaningful interaction. The ability of **L1** to engage this substrate chain in attractive non-covalent interactions therefore emerges as a key factor leading to the experimentally observed product.

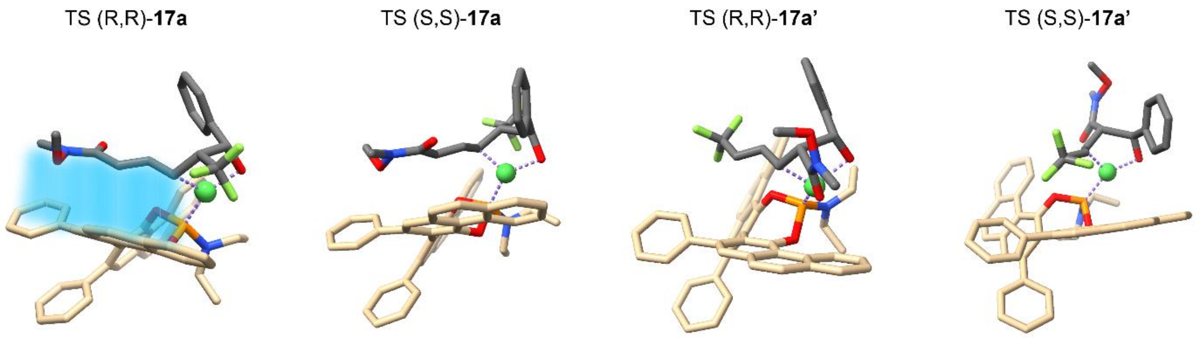

Figure S29. Geometry of the TSs leading to four different isomers, which show the distinctly different orientation of the 1,3-diene relative to the ligand **L1**

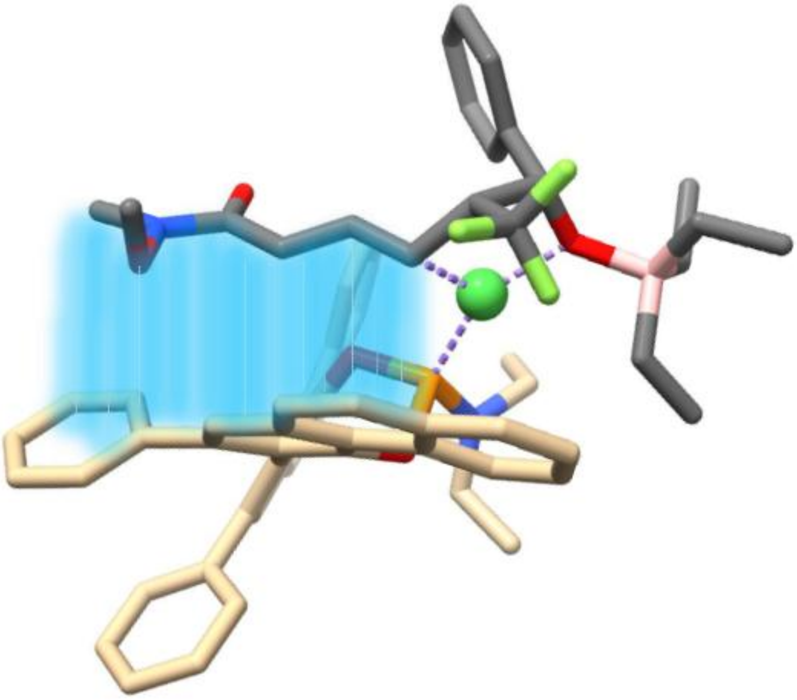

Figure S30. Geometry of the TSs leading to ***(R,R)-17a*** with BEt_3 explicitly included

To gain further insights, an ADLD(D4) analysis on the two transition states leading to **(*R,R*)-17a** and **(*S,S*)-17a** was performed to better understand the possible role of dispersion.[18,19] The analysis confirms that the major TS is stabilized through multiple π-π and σ-π contacts between the diene chain and the large surface of one phenyl-phenanthrene wing of this axially chiral

ligand. To isolate this effect, we summed the atomic dispersion contributions for a selected subset of atoms involved in these interactions (see Figure S31 and S32). The difference of these contributions in the two systems is 1.7 kcal/mol in favor of the major TS, confirming that these particular atoms are much more involved in the major transition state than in the minor one. Interestingly, when extending the sum to include the atomic dispersion contributions of all atoms in the ligand wing, the difference between the two transition states reduces to 0.1 kcal/mol (still favoring the major TS). This observation suggests that the origin of the overall stabilization is not trivial and likely arises from a delicate equilibrium between attractive dispersion forces and steric repulsion.

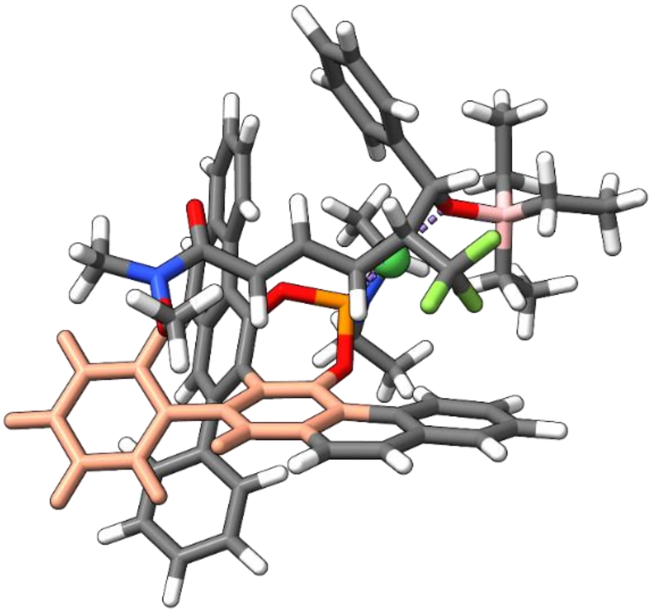

Figure S31. Atoms selected for ADLD(D4) analysis in (*R,R*)-**17a**•BEt_3

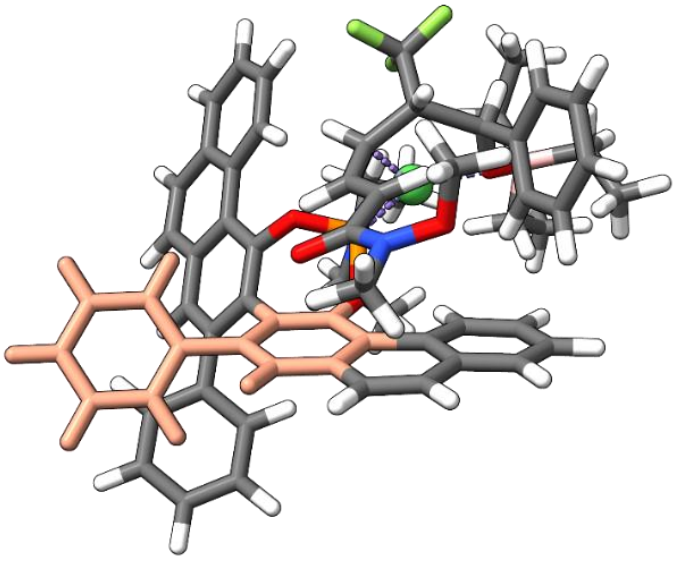

Figure S32. Atoms selected for ADLD(D4) analysis in (*S,S*)-**17a**•BEt_3.

Cartesian Coordinates

K

Electronic energy: -4915.9046 Eh
Enthalpy: -4906.0369 Eh
Gibbs free energy: -4915.0642 Eh

P	0.56514277684927	1.45833398512728	-0.79252793393869
O	-0.69649685867675	1.21239644128934	0.33133756271567
O	0.03222861997553	0.32583963240133	-1.93152291473927
N	0.25940391022892	2.83301352383867	-1.69010415688467
C	-2.37769482293939	-0.07479273967353	-0.74018552973685
C	-1.38786822978548	-1.18777305426985	-0.79598001346756
C	-1.99894578950541	1.12584338956225	-0.10424971460157
C	-0.14902561080316	-0.95009639528927	-1.41602726698316
C	-2.88834123934549	2.22259430119533	0.11160818955022
C	0.88801868955604	-1.92320216209913	-1.53453739050030
C	-3.69712332355257	-0.19434528074790	-1.27433585363139
C	-1.65090604313823	-2.47689163737889	-0.23814156848122
C	-4.20840413709732	2.06917954732834	-0.43691150373532
C	0.60334597423206	-3.20051339475610	-0.93936756221538
C	-4.57295471281731	0.87977337509653	-1.11171173152455
C	-0.64955814819247	-3.44396933922136	-0.32754328879929
C	-2.54715212573443	3.46423665191563	0.80995427836986
C	2.19428222972445	-1.70433041248844	-2.15866092956229
C	-3.53024089364245	4.51932216929042	0.85113183582572
C	3.17571446698450	-2.75852401056140	-2.08116739387176
C	-4.15215387642284	-1.40008023250990	-2.01180238905278
C	-2.92318561677773	-2.81659900560437	0.44885478240788
C	-5.15668398332645	3.14487066011696	-0.34494080144671
C	1.60691756116435	-4.22863640847998	-0.92389492707127
C	-4.82480380112032	4.32864086786399	0.25787475452934
C	2.84867016213399	-4.01085651547346	-1.45800929508553
C	-1.31436097647404	3.70635772438409	1.48381046828581
C	2.57937494553174	-0.51504363550478	-2.84339572313663
C	-3.23201709701079	5.74791850544108	1.49979405880594
C	4.47564772157039	-2.56590803565268	-2.62012729920164
C	-5.43260939703480	-1.94033236515614	-1.76015634212013
C	-3.56601549749362	-4.04137947296165	0.16327670281261
C	-3.33930123139178	-2.01923271928188	-2.98761150419914
C	-3.50372624449842	-1.95618196480073	1.40760359941786
C	-1.05322836184859	4.91642415292732	2.12389159637414
C	3.86015881624773	-0.35455841717260	-3.36848489770750
C	-2.00829290391906	5.95573219157955	2.12604375903115
C	4.82642476934811	-1.37512043796866	-3.24484203401691
C	-5.87983399337730	-3.07702582000908	-2.44633135213277
C	-4.76391514567559	-4.39001386603892	0.80114668349231
C	-3.78670782138073	-3.15688078845402	-3.67307754835957
C	-4.70271971711985	-2.30573727883713	2.04372414898537
C	-5.05594832528962	-3.69457601100279	-3.40106032201503
C	-5.34139308000284	-3.51978994411477	1.73958367592341
H	-5.57944189078088	0.82274282305387	-1.55258998865040
H	-0.81590465110402	-4.42989117512852	0.13219970096411
H	-6.15470782461586	2.99619912454324	-0.78501125268039
H	1.35607337604890	-5.19219319510901	-0.45438740134238
H	-5.55103409861659	5.15464285173534	0.31182551479162
H	3.62309049845841	-4.79145818245688	-1.41877452303186
H	-0.54829589367288	2.92985727927601	1.52611882409150
H	1.85754980604485	0.29005305531374	-2.99196200756158
H	-4.00292344673183	6.53450609405487	1.50389659495930
H	5.20723023720405	-3.38148090362062	-2.52113214476839
H	-6.06697733285101	-1.48322061847914	-0.98608018364132
H	-3.13658257564910	-4.70917152398217	-0.59866056941098
H	-2.35264041463398	-1.59435851957342	-3.22087726136502
H	-2.99029381546423	-1.02250997850914	1.67284831383507
H	-0.08661159256369	5.04700548847081	2.63437634972574
H	4.11229071439804	0.58890158529976	-3.87512609722659
H	-1.79292900297236	6.91199653324970	2.62666374375522

H	5.84117221315745	-1.23530090477381	-3.64708483995188
H	-6.87419234758191	-3.49282823531771	-2.22235455539724
H	-5.25762516485832	-5.34172131097738	0.55157534930434
H	-3.14012618998016	-3.62358892923712	-4.43209748473697
H	-5.13927442863167	-1.62549368850346	2.79143854516562
H	-5.40349496376871	-4.59159621231034	-3.93631610708737
H	-6.28587782497741	-3.78885092899583	2.23720809184087
C	-0.78616236475186	2.93365509792784	-2.72455976693776
C	0.90329618585953	4.07257147199502	-1.22728626660663
H	-1.60567844528919	2.22776393852839	-2.48620398207418
H	-1.22464903947594	3.95179211341880	-2.64374621838788
C	-0.28645358177763	2.66561474760359	-4.14454829955884
H	0.09687421823245	1.63013876183584	-4.23202744434662
H	-1.11980684115469	2.78388095687722	-4.86729589663210
H	0.52392053794386	3.36098787130385	-4.43686342775675
C	1.73087543031561	4.79012776323690	-2.28813659844507
H	0.11868994225171	4.74726918007906	-0.81543706870411
H	1.55907788666680	3.79659450737514	-0.37543633617709
H	2.51862860347496	4.11924363361560	-2.68194466873448
H	1.10638444731387	5.15100486105737	-3.13018018091738
H	2.22252835310611	5.67626553835804	-1.83743098821093
Ni	2.50485984316837	1.07485104948090	0.05534363834656
H	-1.86229443593022	1.29772241526897	3.21120659020427
H	-0.46699408804272	1.92189594790504	4.17247016054375
C	-0.98626153960907	1.00899246268897	3.83116280737698
H	-1.33284627839092	0.42903517947496	4.71026563913369
N	-0.04042466522524	0.23737543513346	3.05543910709358
O	-0.57874927020453	-0.91996189170544	2.48828525221339
H	3.73045854030075	0.64525901071297	2.26209927345436
H	-0.82941145151676	-2.90600398473536	2.78853303259952
C	5.35442908216196	-1.34763662411769	1.39013973747991
C	3.24856731500874	-0.05032682381590	1.55145720519961
C	4.05167584665298	-1.15925495730374	1.06356304302725
C	-0.30921024811279	-2.07064757177239	3.29472847178479
C	6.20197576024742	-2.41546600676904	0.79709326101880
C	1.08898940034753	0.78370264087559	2.48363903419494
C	1.82371211463605	-0.07960638558163	1.51087726014830
H	0.78101837423908	-2.28268612556165	3.34184796305796
H	3.56202787098842	-1.87497551886074	0.38186079133071
H	1.31422548680656	-0.98187649463112	1.13908161613692
O	1.48672407905393	1.91247319119895	2.81813373121178
H	-0.71279998989616	-1.95476951154296	4.32396371916110
C	6.73571301949111	3.61405416904027	2.40909489790899
H	7.34185717870032	4.11952153916294	3.17650985877290
C	5.48660560036219	4.14554131022105	2.03429719684371
C	7.20966944173945	2.44284252731158	1.79586657273876
H	5.11715000895485	5.06703971469371	2.51005041588156
H	8.19202878041127	2.03186262565070	2.07554069323539
C	4.71253405003218	3.50703420380113	1.05993866872592
C	6.43569432492150	1.79977167484030	0.81826561590346
C	5.17535176565023	2.31884258912788	0.44920281248242
H	3.73505496464620	3.91507847288299	0.76245604351395
H	6.80686173074087	0.89008989542558	0.32188573994126
C	4.37657829191531	1.61564063429726	-0.57682376952859
O	3.40384107841626	2.19565223938804	-1.21147688188858
H	4.85430883203170	0.71286117167702	-1.02966117617383
H	5.88258029257013	-0.66522421065644	2.07341746781464
F	5.48881569054969	-3.43761271511956	0.25721742884708
F	7.04806102939496	-2.95554120675756	1.71349268943744
F	6.99960479366336	-1.92918631351349	-0.20951832267412

(R,R)-K.1

Electronic energy: -4915.8912 Eh

Enthalpy: -4906.0273 Eh

Gibbs free energy: -4915.0520 Eh

P	0.85130826968556	1.20831030092164	-1.067420792
O	-0.45972541603718	1.50507916104410	-0.031106706
O	0.16547907586894	-0.08285550863667	-1.930913714
N	0.85992069147343	2.28795093196083	-2.341609684
C	-2.35649089632238	0.32753860201253	-0.813835569
C	-1.61434649126212	-0.95027325723784	-0.614832085
C	-1.75963621985622	1.55973873300032	-0.494915977
C	-0.39439342708864	-1.16420717930995	-1.282649397
C	-2.43218752310310	2.81816893181956	-0.586443968
C	0.22736397284917	-2.44602858102581	-1.398663216
C	-3.69270955201072	0.31226723730627	-1.326461044
C	-2.19000509986269	-2.02110652306272	0.137705217
C	-3.78695185626338	2.76179575977109	-1.065064709
C	-0.43205571041205	-3.52050894756028	-0.706758283
C	-4.37207331105899	1.52521262621253	-1.427037788
C	-1.60389593391831	-3.28067701981563	0.047568027
C	-1.85523549463300	4.12283053915215	-0.251923294
C	1.46165070693961	-2.72998557395727	-2.135994004
C	-2.66568298137736	5.30238935562557	-0.436272124
C	2.01574661835680	-4.06019866085230	-2.058714819
C	-4.36593668468528	-0.92846601884362	-1.791236244
C	-3.33892969623712	-1.81178434966585	1.05151681
C	-4.55268691871726	3.96747894390419	-1.219006059
C	0.14940230840078	-4.83375891011079	-0.68836024
C	-4.01272661937105	5.18962840663486	-0.920950078
C	1.33722949468228	-5.08845270773976	-1.319939687
C	-0.53783078566492	4.32178230207498	0.251194531
C	2.15967112649621	-1.78825581512391	-2.947579561
C	-2.13717846721537	6.58653857355080	-0.133937033
C	3.22890390832854	-4.36721992055814	-2.731056738
C	-5.70032471302180	-1.19190647547169	-1.411425143
C	-4.36332398521427	-2.77793719838611	1.158082446
C	-3.71634458679260	-1.84471402254837	-2.648486222
C	-3.39676340888443	-0.67097226653276	1.883394262
C	-0.04371583250314	5.59210299603545	0.539271102
C	3.34298944600018	-2.11970340073823	-3.603960692
C	-0.84146639745438	6.74014178575236	0.345541443
C	3.89566790263054	-3.41275912255212	-3.490297146
C	-6.36304116847931	-2.34123884186062	-1.863768519
C	-5.42232255990201	-2.60392197154656	2.059577299
C	-4.37792497158348	-2.99510221123772	-3.098758286
C	-4.45668467590949	-0.49700166364603	2.783283196
C	-5.70253850382367	-3.25083479849038	-2.705288767
C	-5.47633012998670	-1.45932722421500	2.872563093
H	-5.39151638050350	1.53557963580596	-1.840857701
H	-2.01139098812111	-4.10921266202917	0.641828454
H	-5.58556665834310	3.88192800994699	-1.589982626
H	-0.37709026241529	-5.62436709280617	-0.132407044
H	-4.60301185761154	6.11078823567979	-1.046074055
H	1.78971692388041	-6.09173231005227	-1.286008923
H	0.12264631275805	3.47243386535466	0.428412091
H	1.75942832258645	-0.77911928946198	-3.067901411
H	-2.78433415253515	7.46436574415739	-0.288079767
H	3.62841333162882	-5.38979869050281	-2.643244811
H	-6.20914190856412	-0.49996026984876	-0.723307841
H	-4.34324367796780	-3.66009882912226	0.500659534
H	-2.68568258946363	-1.64526351601628	-2.97577203
H	-2.58122355492507	0.06494509149083	1.85391324
H	0.98390986168059	5.68081912498923	0.923232124
H	3.84986861404780	-1.35503108825916	-4.21207417
H	-0.44609524404727	7.74169160051657	0.573965085
H	4.83603505752810	-3.66506938350350	-4.003344464
H	-7.39898399069236	-2.53439130170944	-1.545232702

H	-6.21849852368071	-3.36203442549146	2.118250922
H	-3.85565532646941	-3.69516281901745	-3.768901125
H	-4.47783471743488	0.39316842658234	3.430472807
H	-6.21957904886710	-4.15665269912029	-3.057593235
H	-6.30969067724810	-1.32014764655339	3.578328816
C	-0.23171227850606	2.39291774255332	-3.324208416
C	2.05483670713453	3.13209795941829	-2.549705475
H	-1.09972461026077	1.82596699535219	-2.94056875
H	-0.54876545301818	3.45900042282633	-3.364739834
C	0.12353749053564	1.88814323141748	-4.722096485
H	0.43082299593715	0.82357917455321	-4.690747287
H	-0.76048028900998	1.97424900205095	-5.386186923
H	0.94315228006667	2.47564454469401	-5.181885116
C	3.23018210682059	2.40047914687736	-3.198964395
H	1.72758163958648	3.98974978238713	-3.173592432
H	2.37117604814038	3.55388419553100	-1.576328922
H	3.57443093496249	1.56656803827445	-2.553200279
H	2.96587157195830	1.97725885639760	-4.188202788
H	4.08611419656432	3.09316055983040	-3.335214684
Ni	2.55491570782061	1.25731473714691	0.304916245
H	-2.86309671690443	-3.16735573980908	3.775100152
H	-2.11426343548111	-1.83993164623331	4.74658697
C	-1.95834670105702	-2.85448030402842	4.338536174
H	-1.78119117901234	-3.56373131194760	5.174670792
N	-0.81560193065939	-2.78193404427524	3.459860069
O	-0.51614133820351	-3.95632400100940	2.772399384
H	1.91805382200976	-0.03738734176149	2.699285921
H	0.63809948479699	-5.62370425361454	2.838864836
C	3.96382674985969	0.00471518356655	0.916055364
C	1.81380500123854	-0.78668412370929	1.894628591
C	2.73552935967992	-0.69374122307717	0.769259191
C	0.50390590724786	-4.70610250419462	3.443253419
C	5.08415796011650	-0.18059678378176	-0.054549594
C	-0.15100213022376	-1.61304564406782	3.135891644
C	0.82868894806846	-1.71389060591280	2.027099131
H	1.46142466232527	-4.14254899636956	3.477030439
H	2.59167961483424	-1.37811722877418	-0.083796444
H	0.71279498001580	-2.52825684630116	1.299446805
O	-0.39326008734906	-0.55938954062226	3.74547071
H	0.19606206586176	-4.98501803800280	4.474545568
C	2.21958588978556	1.96124180425398	5.506770527
H	1.93199845882619	1.73221316027434	6.544271254
C	1.23810785635584	2.32872183563782	4.568803481
C	3.56783696262759	1.88460275904041	5.117011021
H	0.18078016843699	2.37644385927838	4.868069237
H	4.33914913749490	1.60433707858091	5.850819159
C	1.59787717573294	2.61207070572815	3.246703323
C	3.93174427547640	2.16386780467379	3.79248827
C	2.94971873751159	2.52174037138945	2.841797824
H	0.83380391181827	2.88626025635265	2.505318471
H	4.98856605224924	2.10886879251946	3.483989802
C	3.33429896470930	2.73297349406652	1.429807219
O	2.47490177300103	3.16932991896180	0.552571274
H	4.42396430957636	2.82873717377951	1.214185186
H	4.32414505387042	0.25551890919688	1.928004932
F	4.65323753839093	-0.41221425935686	-1.325278866
F	5.88728729540406	-1.22851748865316	0.27883126
F	5.88960067115680	0.91755860000876	-0.100672173

(R,S)-K.1
Electronic energy: -4915.8916 Eh
Enthalpy: -4906.0262 Eh
Gibbs free energy: -4915.0506 Eh

P	0.71042250602773	1.42835342878756	-0.17963916
O	-0.66178752601582	1.22057488390493	0.811484335
O	0.43995043691499	0.18560042134155	-1.295019308
N	0.39431727929129	2.70143549259490	-1.237781366

C	-2.24797729186652	0.20007385101739	-0.623605506
C	-1.43736861542027	-1.04609917074480	-0.518880813
C	-1.88328477855406	1.29889870514159	0.171287976
C	-0.07627751025216	-1.03129427431771	-0.87416691
C	-2.69208841078315	2.46145134748751	0.319999665
C	0.74442744999638	-2.20070521211105	-0.884176528
C	-3.41923427379218	0.28306270328927	-1.437118454
C	-2.03930069415835	-2.26050413264105	-0.070877568
C	-3.83591263918233	2.54352115510535	-0.545135403
C	0.10343376434018	-3.41095179845848	-0.440040747
C	-4.16490305539657	1.46339044910767	-1.398427814
C	-1.25625099195806	-3.41221180124164	-0.055375519
C	-2.42270294452124	3.54930298303967	1.255746134
C	2.15615434962702	-2.25432729077862	-1.269938446
C	-3.21081009856670	4.74994537525732	1.155625328
C	2.87334640385808	-3.49299109931265	-1.087577056
C	-3.84146556719318	-0.82120457679263	-2.334745938
C	-3.44560006091671	-2.33008457805249	0.400981234
C	-4.61773013674647	3.74973496760440	-0.576243525
C	0.85844834785659	-4.62621886754677	-0.309615356
C	-4.29474683432408	4.82224047341263	0.213842192
C	2.19591191483744	-4.66055784573194	-0.597802078
C	-1.45398372283110	3.48117188666450	2.297469423
C	2.88609480615667	-1.16431764892005	-1.825043515
C	-2.94084352425989	5.84592601843853	2.018638083
C	4.25457550669896	-3.57301620743893	-1.409781059
C	-5.20290520860448	-1.19014643286827	-2.405886214
C	-4.27345166352184	-3.39632694274487	-0.013912498
C	-2.91252788787975	-1.51283316313407	-3.144072484
C	-3.97952619481667	-1.36858768257995	1.288102101
C	-1.22393291692921	4.56431822921385	3.143055938
C	4.23680030012286	-1.27495208926172	-2.145143392
C	-1.95131164492648	5.76617942775939	2.992456741
C	4.93678566678278	-2.47949953346261	-1.927339027
C	-5.62286597901817	-2.22800303856030	-3.248639348
C	-5.59983599194326	-3.49245503520230	0.428528434
C	-3.33245928019606	-2.55155958629323	-3.986113803
C	-5.30731606532176	-1.46300888728053	1.726917988
C	-4.68819776085477	-2.91719283403094	-4.038197217
C	-6.12540687844906	-2.52093576813862	1.295885584
H	-5.03125386320960	1.57425768634507	-2.067691649
H	-1.68368846681819	-4.34669824426229	0.333506591
H	-5.47431258193844	3.79660680218896	-1.266220781
H	0.33318736493855	-5.52464741299582	0.048955977
H	-4.88314799316357	5.75180595400700	0.164783161
H	2.77596891515677	-5.58835598917315	-0.474780886
H	-0.89832950344627	2.55104529833964	2.45483399
H	2.37956408027916	-0.21552899395702	-2.016669637
H	-3.54771969053584	6.75871511399472	1.911109582
H	4.77243434882416	-4.53122571903429	-1.246369933
H	-5.93251492239656	-0.67797111853891	-1.760633447
H	-3.87799321990762	-4.13915991272296	-0.723130159
H	-1.85232988571595	-1.22271238324536	-3.122453698
H	-3.34219116724835	-0.55778299664606	1.667181428
H	-0.47048700198564	4.47399096669061	3.94049901
H	4.75857408285241	-0.40396131204409	-2.563560831
H	-1.75365216368336	6.62199582437827	3.655770319
H	6.00787893394422	-2.55151172603488	-2.169801238
H	-6.68652233200595	-2.51032069617902	-3.277858247
H	-6.23181444664393	-4.32407154710196	0.080703945
H	-2.59436070898266	-3.07651590290343	-4.611866659
H	-5.70163000405459	-0.70717326187758	2.423482458
H	-5.01554471590159	-3.73679293940373	-4.696242994
H	-7.16912580766352	-2.59007801281059	1.639270365
C	-0.29518663051495	2.55942923012280	-2.534108803
C	0.45666091566702	4.05528749864693	-0.657043012
H	-1.01284578603472	1.71777153234144	-2.487930491

H	-0.90644652694055	3.47637793564291	-2.673720859
C	0.65599566771393	2.36121103351392	-3.713879629
H	1.24522702634637	1.43134925450727	-3.587341565
H	0.08192157827222	2.27525995977876	-4.659412571
H	1.36372675478080	3.20787938669386	-3.816659677
C	1.30433817287615	5.05036792598052	-1.441190079
H	-0.58398743247933	4.43787071223870	-0.555149434
H	0.85118002389820	3.96623078546668	0.374147339
H	2.36289693046864	4.72994376813913	-1.476780809
H	0.93463898787728	5.18820790208311	-2.477678755
H	1.26340145515319	6.04005639062399	-0.942565912
Ni	2.54898908374378	1.35420244540738	0.97889927
H	-3.66717207590381	-3.25372203541189	3.495391637
H	-3.26300049136870	-1.81972427678578	4.520057907
C	-2.93126040761518	-2.82602530720182	4.207398336
H	-2.86070284396292	-3.48108844239764	5.102222227
N	-1.64530866166710	-2.66743806297197	3.571934298
O	-1.10809285229287	-3.82865851339773	3.020729988
H	0.91457964355337	0.37041468257766	3.321165227
H	0.08589179574379	-5.41808686968747	3.429045486
C	3.52132475209538	0.29258076385536	2.312415829
C	1.13718585218520	-0.47710897934440	2.648322543
C	2.37120222700839	-0.43660854947387	1.873428026
C	-0.25079441937305	-4.50175483581420	3.950779544
C	4.88051876016952	-0.28346493042102	2.073711795
C	-1.00896243892661	-1.45355964946730	3.376335321
C	0.25652352901818	-1.51007028144368	2.611775749
H	0.63187146281021	-3.87758471836322	4.21008727
H	2.53403041266012	-1.28110338151193	1.17984804
H	0.45509009992501	-2.39302074717503	1.988757864
O	-1.49721193997304	-0.40620375544225	3.833200923
H	-0.79421754713326	-4.78572617474436	4.878142846
C	6.06077275469887	1.78312268787464	-3.170216406
H	6.55423961327964	1.60698852354502	-4.138321774
C	4.70505427330678	2.15960555969741	-3.127016385
C	6.78092413477362	1.62154800525289	-1.973384854
H	4.13274910930801	2.27250476606634	-4.060829514
H	7.83867528783215	1.31853267728491	-2.004045851
C	4.07911862539908	2.38712947952834	-1.896873898
C	6.15820853762754	1.85407349979619	-0.739580992
C	4.80457839184606	2.25308830780120	-0.689617355
H	3.02019511155910	2.67706632255412	-1.848379766
H	6.72184704398406	1.73352878076957	0.197180294
C	4.16166085098393	2.59323546645418	0.599306982
O	3.02249340650607	3.20230720975803	0.629764913
H	4.81984567164478	2.61304787902362	1.496556049
H	3.47236231614369	0.82792874851523	3.280070793
F	5.04206574876320	-0.77975030606551	0.821932991
F	5.14472648806755	-1.31065933507402	2.932824204
F	5.87578230759124	0.63136187401806	2.278884553

(S,S)-K.1
Electronic energy: -4915.8981 Eh
Enthalpy: -4906.0362 Eh
Gibbs free energy: -4915.0584 Eh

P	0.75203230168943	1.07146344642988	-0.974235645
O	-0.56334330179783	1.41410528013880	0.065448878
O	0.02808262782557	-0.18974264056297	-1.84239419
N	0.79264274888113	2.20172923644469	-2.202756594
C	-2.46293349771781	0.31693506414635	-0.817593497
C	-1.77719215444567	-0.97175868463248	-0.517620954
C	-1.83195236938273	1.52662154114882	-0.471899309
C	-0.53140449509169	-1.23063543147469	-1.117170272
C	-2.44777867237267	2.80839417329609	-0.619821636
C	0.11787395864664	-2.49964350094684	-1.069276496
C	-3.75670253426642	0.34344866124602	-1.422751032
C	-2.39153247533168	-1.97763018592968	0.287674355

C	-3.75599670415262	2.79943776800682	-1.219845496
C	-0.51745118042531	-3.48797758658293	-0.240087313
C	-4.36561881275546	1.58380742349568	-1.608449054
C	-1.73883499005940	-3.20285045236516	0.41107786
C	-1.86173235738136	4.09353258210801	-0.228576073
C	1.33276008870792	-2.86444076006851	-1.799520189
C	-2.60008257333030	5.30144681896741	-0.507308253
C	1.91750375560718	-4.16031772624349	-1.555180035
C	-4.45813640263149	-0.88461901225644	-1.877139768
C	-3.66570548734165	-1.75313834526279	1.013405778
C	-4.44984865710272	4.03311992135809	-1.466133812
C	0.10566424918280	-4.76631443232049	-0.03724958
C	-3.89131413718263	5.23697370720674	-1.132105493
C	1.28918435999010	-5.08001249093413	-0.648226366
C	-0.60120140660960	4.24655411127917	0.416021599
C	1.96418442521325	-2.04135342693029	-2.775052289
C	-2.05635288810945	6.56795297273478	-0.162761051
C	3.10710486162149	-4.54175307670339	-2.230499198
C	-3.79187993754726	-1.88762720190059	-2.61626386
C	-3.91965181049669	-0.54803611343824	1.705236306
C	-5.83143637595714	-1.05563473636415	-1.595485393
C	-4.64493926942200	-2.76886717023777	1.053866769
C	-0.09150713587059	5.50121509760525	0.746338965
C	3.12255031264582	-2.44727225899546	-3.434329011
C	-0.81564094089938	6.67653320948074	0.453141126
C	3.71279947950871	-3.69798450215793	-3.154958717
C	-4.47654678478716	-3.03300123174742	-3.044839732
C	-5.11922735398533	-0.36302706114504	2.405879081
C	-6.51617204480070	-2.20020732953605	-2.025259515
C	-5.84438668257191	-2.58582950179140	1.755058109
C	-5.83993785297872	-3.19697573373353	-2.746997999
C	-6.08915967138532	-1.38004855421647	2.431726098
H	-5.34720874252780	1.63170109557707	-2.103113603
H	-2.17403882771501	-3.97640367959026	1.059342933
H	-5.44355083052817	3.98311609247015	-1.937073964
H	-0.38961151423970	-5.48311261907247	0.634778667
H	-4.42480653463603	6.17964767891786	-1.329978313
H	1.77010182082398	-6.05530063549817	-0.475971133
H	-0.01137446229806	3.36290340018291	0.664843378
H	1.53526544569010	-1.06648853211129	-3.017749034
H	-2.64729481117266	7.46800288362662	-0.394444463
H	3.53724625060383	-5.53128155149918	-2.010292061
H	-2.72948271928836	-1.76155276238472	-2.869084189
H	-3.15949686027955	0.24561611654924	1.707647434
H	-6.35628304412648	-0.29527936947200	-0.997616369
H	-4.47443872843763	-3.70196131246347	0.496136579
H	0.88794905444870	5.56369002365539	1.241550178
H	3.58219116805546	-1.77358018264491	-4.173062096
H	-0.40534695819096	7.66401212072301	0.713688061
H	4.63630852914350	-4.00560963142191	-3.668475182
H	-3.94042991083005	-3.80202060933587	-3.621942118
H	-5.29602514404275	0.58270081445854	2.941272138
H	-7.58290677217711	-2.32109716322839	-1.781653025
H	-6.60006384744536	-3.38641273440242	1.761486843
H	-6.37477789748842	-4.09957319224365	-3.080469404
H	-7.03303829485004	-1.23195559717314	2.978653369
C	-0.12004374487638	2.23378429944889	-3.359038495
C	1.88994661552862	3.18031086600073	-2.144817777
H	0.41837383645184	1.84930385748212	-4.255271088
H	-0.93758421383736	1.51587045631166	-3.162124064
C	-0.70021202165221	3.61382733836789	-3.659018064
H	-1.41921564145771	3.53154186481737	-4.498463397
H	-1.23950586959683	4.02653132188130	-2.785073437
H	0.07861168237253	4.34241273117049	-3.960952496
C	2.89748854599425	3.04755062821282	-3.281511358
H	1.46030473857486	4.20481105687362	-2.100718516
H	2.40215708340147	3.02900526252700	-1.16909496

H	3.69041022704498	3.81489838037739	-3.172110082
H	3.37297769117134	2.04746528551741	-3.250024758
H	2.42761410752205	3.19456606254363	-4.275086143
Ni	2.62834136800064	0.85147047815377	0.097292986
H	0.28971677616510	-4.93647707940550	3.634008085
H	-1.42264229653368	-4.93879837431443	3.046262155
C	-0.70010920845618	-4.45714042062948	3.740331095
H	-1.06279596597030	-4.58878767183523	4.781189745
N	-0.52925914936602	-3.06008400290288	3.421127612
O	-1.70042955363623	-2.30545314289360	3.410177704
H	2.68035923024038	-1.05230258029892	2.338407152
H	-2.06059810407234	-2.47251878565103	5.486016998
C	3.19347487885188	1.53364301947260	1.883307945
C	1.75517728132413	-0.45383941568763	2.258839849
C	1.89517650422907	0.96030020879712	1.945631807
C	-1.95530216592496	-1.70783686801131	4.685833185
C	3.41217048556806	3.00691782261915	1.9657836
C	0.62240327286783	-2.54165611341878	2.85127265
C	0.58078436793014	-1.10400448097117	2.486984087
H	-1.15416369364261	-0.98788535419174	4.961567067
H	0.99891057395846	1.59782326476968	2.017181587
H	-0.38523009450879	-0.58257434679306	2.432773004
O	1.63175573204031	-3.25200540553867	2.726874752
H	-2.91542396778858	-1.17068916286279	4.564514538
C	5.62624018198597	-3.02021670084451	1.925059663
H	5.88857237118345	-3.88958010428777	2.547174007
C	4.61471343172047	-3.12753424532391	0.953540214
C	6.29832986410591	-1.79842633455009	2.10424617
H	4.07269929494732	-4.07504496508787	0.817366822
H	7.09308216174890	-1.71237389943710	2.861280093
C	4.27476054739428	-2.02194103039223	0.167349594
C	5.95585689939794	-0.68662456746710	1.321526832
C	4.93711952587406	-0.78694876926872	0.347763357
H	3.47970264477239	-2.09108664362215	-0.585991067
H	6.47879325053859	0.27381730694400	1.460713708
C	4.52852064758837	0.41084092031031	-0.418478229
O	3.69455242921808	0.34127274699490	-1.41573463
H	5.17543557498613	1.31385851203110	-0.310698888
H	4.05344200244101	0.96232365635869	2.272909502
F	4.57078548656819	3.37734235198658	1.355835453
F	3.49633838149431	3.44989523682533	3.249498206
F	2.40993004379644	3.72388654085840	1.376435092

(S,R)-K.1
Electronic energy: -4915.8921 Eh
Enthalpy: -4906.0300 Eh
Gibbs free energy: -4915.0518 Eh

P	0.71014802924125	0.74512999687709	-1.19357710588087
O	-0.45752752021996	1.13572546961588	-0.00964011161417
O	-0.07437783751062	-0.61008967406231	-1.83632645187584
N	0.53933116023928	1.76366274932147	-2.50749774767327
C	-2.49773796529370	0.19951960950605	-0.75638988051062
C	-1.92620893673369	-1.14168277415680	-0.44654726203797
C	-1.75010456365820	1.35075056340624	-0.44723802697825
C	-0.69916960757669	-1.53537248283933	-1.01323988497913
C	-2.25725903415152	2.68095545973631	-0.55406005606366
C	-0.13866021018395	-2.84120893940061	-0.84713982553510
C	-3.81137772534815	0.34542066883756	-1.30075161660026
C	-2.64788866604803	-2.05916096855672	0.37711563273274
C	-3.59834789354638	2.79049299406036	-1.06409064111852
C	-0.93782786228630	-3.76492131130363	-0.08583812219109
C	-4.32547772240310	1.63516100221166	-1.43349325126380
C	-2.15577416733515	-3.35458586535504	0.49997661578401
C	-1.52636976756645	3.90480800628832	-0.21373014207482
C	1.15410176889089	-3.29825102020445	-1.36231979737451
C	-2.17189495523246	5.17498098202071	-0.44076166145510
C	1.59555238784233	-4.63293918299517	-1.03683487747797

C	-4.62291793545697	-0.80918479115040	-1.76220054449897
C	-3.86341119735507	-1.67399841831658	1.13528287242416
C	-4.19549102708131	4.08231373187275	-1.25910122306850
C	-0.47767164384532	-5.10422731009684	0.15112838761392
C	-3.50723028680286	5.22896730228656	-0.96721901734817
C	0.74925591847088	-5.51697004737267	-0.28805028678214
C	-0.21047676285331	3.94258270500154	0.33183520233289
C	2.02853197536409	-2.51208900428821	-2.16302690443081
C	-1.49265842792839	6.38612503001393	-0.14011238529896
C	2.86782668260095	-5.08980363047364	-1.47108647362138
C	-4.05134173906819	-1.85244351570217	-2.52466677527013
C	-3.92019961989172	-0.46221115241384	1.85938676899736
C	-6.00459242373076	-0.86473576665303	-1.47470552392869
C	-4.97470686296315	-2.54228755859933	1.19231504157708
C	0.43289223824045	5.14480662501963	0.62078304449201
C	3.27474449693712	-2.98244869693759	-2.57382658159189
C	-0.20487024657294	6.38098005253653	0.38214238374669
C	3.70948468405616	-4.27746757027516	-2.22261207666924
C	-4.83625972663341	-2.92394351373930	-2.97180092302152
C	-5.05788004184136	-0.12595039471716	2.60583291978230
C	-6.78990338929485	-1.93501961511190	-1.92331240879567
C	-6.11236345431352	-2.20775964040100	1.93948688167869
C	-6.20767355528674	-2.97256062527294	-2.66943648935457
C	-6.16131129959949	-0.99564043429691	2.64662153488992
H	-5.32259260234583	1.77314174413945	-1.87743045701329
H	-2.69620824913722	-4.07508468632738	1.13070934849099
H	-5.21970999359571	4.12464207990951	-1.66016189973059
H	-1.12836171956240	-5.78084112008469	0.72528451610512
H	-3.96623293271597	6.21706230176223	-1.12615732047599
H	1.11226454251379	-6.53446368304325	-0.07609592096793
H	0.31852816400380	3.01038439563399	0.53707756964675
H	1.72924903449330	-1.51430248032403	-2.48279629672142
H	-2.01625155851473	7.33653311630818	-0.32879381958332
H	3.17443258112414	-6.11174478449353	-1.19788647433212
H	-2.98277220726758	-1.81492601801543	-2.78093294076288
H	-3.05515416814165	0.21619993024247	1.84971196736976
H	-6.45830223043485	-0.07380136799397	-0.85858500481054
H	-4.95739370183953	-3.47745135632525	0.61240853839130
H	1.45009399861983	5.11397073208053	1.03706023003582
H	3.91426192175932	-2.32231316569114	-3.17808004592809
H	0.30942123370175	7.32697115316697	0.61009625026455
H	4.69545522186334	-4.64372501587821	-2.54729316600157
H	-4.37321806107268	-3.72543751844334	-3.56767629512056
H	-5.08069752640151	0.82223993882790	3.16501376904758
H	-7.86212397456384	-1.96608619346743	-1.67577941341320
H	-6.97336959555691	-2.89358582637591	1.95784402562987
H	-6.82180376829346	-3.81707046804113	-3.01808377255505
H	-7.05609502222436	-0.72880843443469	3.22981648977707
C	-0.51278150054844	1.65936757698339	-3.53523554789866
C	1.57699240990977	2.79191877151795	-2.67887743974942
H	-0.07799858236365	1.19615961163089	-4.44999202064092
H	-1.27439831130987	0.94939077807824	-3.16565946285870
C	-1.16833606455499	2.99114095481242	-3.89438015558497
H	-2.00414882431799	2.80547592530279	-4.59813684535536
H	-1.58172881904117	3.49803891259202	-3.00156456140339
H	-0.46478513965325	3.68739281387684	-4.39304989393733
C	2.38682236159754	2.64368171470922	-3.96311279176165
H	1.10600087333837	3.79713512429280	-2.61556716805465
H	2.25168287046883	2.72320416134837	-1.79878556684991
H	3.15689949617765	3.43988553067514	-4.01338146380980
H	2.89860499640528	1.66191650016893	-3.98005614999566
H	1.75665503352854	2.73752757451770	-4.87042800546264
Ni	2.72025257582262	0.55416257712147	-0.37015539522212
H	0.20732332678663	-5.14381376223983	3.52119261323927
H	-1.55494177698455	-4.99494981971531	3.13560553900770
C	-0.71375610125591	-4.56766975747709	3.72180402596468
H	-0.95989017408859	-4.63235949163800	4.80262173667956

N	-0.45339591556218	-3.20468708856869	3.32557260043692
O	-1.54921659147952	-2.34544906071030	3.39331678282353
H	2.78910344839640	-1.53037879625437	1.80319771941836
H	-1.73647374461295	-2.43524175046001	5.49680466283158
C	3.46372092227462	1.05709551506912	1.40214818973682
C	1.92074236166556	-0.84834635143256	1.84819169679141
C	2.14110550558316	0.55457620742201	1.53577441331518
C	-1.63503461175397	-1.70052175124725	4.66864121450147
C	3.71494201051005	2.52681215891058	1.57598796965317
C	0.68154252511418	-2.80935797416573	2.63609206309327
C	0.73168461195339	-1.38193637719897	2.23703076372492
H	-0.75143567220117	-1.05115905097096	4.85332463747509
H	1.30642071350611	1.24914296881493	1.72836222724776
H	-0.17601742919052	-0.76627001087276	2.30317990692864
O	1.61077728764779	-3.60704579682495	2.44193498331514
H	-2.55040006581348	-1.08034006436509	4.61651019367388
C	7.68392144521905	2.91381694436703	-0.65085903763467
H	8.49653556222659	3.64320592001170	-0.51137599415278
C	6.59597597786709	3.22105136601384	-1.48819529760713
C	7.73335725756867	1.67043448063300	0.00114535821042
H	6.55975720152532	4.19146851825088	-2.00723963819370
H	8.58654761505503	1.42185815571550	0.65099889609456
C	5.56032664781469	2.29751189579929	-1.66779711707457
C	6.69719627599607	0.74437583621841	-0.17672428519007
C	5.59598870590926	1.05128819663910	-1.00415858205909
H	4.70980614272007	2.52592339481749	-2.32540746750546
H	6.73460088284547	-0.23417700833663	0.32933402012034
C	4.50943832539833	0.05954542873869	-1.16668759308322
O	3.56560701993337	0.20378791440533	-2.05314506942951
H	4.74106886861152	-0.97257385493514	-0.79843633743693
H	4.32214725394916	0.43343020883965	1.70288727262883
F	5.02584270295100	2.83564550504153	1.49500783011466
F	3.27406543132690	2.99035522551538	2.78021739738814
F	3.06629174294788	3.28574643871013	0.63214879672921

TS (R,R)-17a
Electronic energy: -4915.8522 Eh
Enthalpy: -4905.9714 Eh
Gibbs free energy: -4915.0114 Eh

P	0.92202293693325	0.79867317381790	-1.38411035309410
O	-0.29973685781843	1.36310012408947	-0.35179901028945
O	0.15190136761643	-0.62474938188995	-1.88407752253548
N	0.86326241626727	1.60206843676933	-2.84996203478245
C	-2.29599697189649	0.24685158367677	-0.96582081726266
C	-1.68541078130186	-0.99964479573554	-0.42463138643885
C	-1.57926012120632	1.45174457627687	-0.86479372575359
C	-0.45455502469457	-1.44001483156567	-0.94486043865923
C	-2.09678268545087	2.71868958243474	-1.26462154805032
C	0.15662236340928	-2.67993905166701	-0.58930662404323
C	-3.59466130305977	0.25599827399908	-1.56237867246780
C	-2.34022996631357	-1.77814520097350	0.57768608859705
C	-3.38747894705885	2.69146988415846	-1.89541185630086
C	-0.53719205734859	-3.44838896958909	0.40877360404092
C	-4.09826314576675	1.47390245282985	-2.02110467616044
C	-1.75783997684323	-2.98738138499907	0.95436494586059
C	-1.40678242693166	3.99831815005869	-1.10662391646610
C	1.42203771915310	-3.18871866904311	-1.12433011399454
C	-1.98628411154147	5.16733935178107	-1.71878391101431
C	1.98062823498727	-4.38408088803101	-0.54240026242837
C	-4.40172462515130	-0.97828419517423	-1.73768896108374
C	-3.57819120175589	-1.32265573414772	1.25793751123296
C	-3.94066387454477	3.89638809670197	-2.44952545843180
C	0.04828546272522	-4.65390327120487	0.92659166565399
C	-3.25282357833408	5.07945279891107	-2.39225700625357
C	1.27107951940991	-5.08853088519613	0.48914006323231
C	-0.20936741749236	4.18136126875609	-0.35575910829907
C	2.14643152366743	-2.59352656581415	-2.19790232772555

C	-1.32327502116205	6.42065659783100	-1.62691776921355
C	3.22668160884325	-4.88566016085305	-1.00385635530047
C	-5.77412511952491	-0.97326315709067	-1.40489702565075
C	-3.72851274795845	0.01338807002606	1.69342608026890
C	-3.83668180885177	-2.16668566924910	-2.25186003956394
C	-4.62002164917940	-2.23880946948230	1.52132788853753
C	0.40868182677491	5.42737909050256	-0.26675700569394
C	3.35849560406278	-3.11730288599949	-2.64153545861266
C	-0.13413894413356	6.55531236458034	-0.92015900430985
C	3.91729226644759	-4.26218192528369	-2.03570787137532
C	-6.55618937517814	-2.12507087172378	-1.56470812917401
C	-4.89561357024214	0.42139417668898	2.35331147519056
C	-4.61918017160339	-3.31838573308287	-2.41204765238282
C	-5.78624715507191	-1.83015531647548	2.18229961420189
C	-5.98048365892220	-3.30447036389503	-2.06396323375814
C	-5.93214478105176	-0.49558324383302	2.59532583666840
H	-5.07195846195157	1.49185972379167	-2.53345062552284
H	-2.21582459574698	-3.57464956584976	1.76170617818669
H	-4.92470879521050	3.83888428389109	-2.93951476458900
H	-0.50378833448445	-5.20554538864714	1.70287905713155
H	-3.67179670165138	5.99417716235250	-2.83993956066932
H	1.72755731367317	-5.99893881695725	0.90781322713289
H	0.23256926720913	3.33292516739006	0.17463571851042
H	1.74103020241515	-1.71230272904672	-2.70094293017568
H	-1.78406525446660	7.29227870238118	-2.11776612029658
H	3.63148783500719	-5.79256252009521	-0.52791378385019
H	-6.22024651558347	-0.06191818759031	-0.97882751489321
H	-2.90961074316482	0.73119041096550	1.54930100704931
H	-2.77620735101737	-2.18294508068493	-2.54164021352049
H	-4.52655078791807	-3.27670678544655	1.16799332175483
H	1.33347446896052	5.52809550681691	0.32079335700803
H	3.88471452624332	-2.62201047175786	-3.47159200569347
H	0.36899167636495	7.53187332407259	-0.85486860329138
H	4.88259377113214	-4.66135782379234	-2.38206572369429
H	-7.62011324315227	-2.10511417218922	-1.28274698707606
H	-4.98835781182509	1.46422262323849	2.69353313295081
H	-4.16210523249616	-4.23393480941852	-2.81799377769485
H	-6.59384203492280	-2.55631366674336	2.36254549043248
H	-6.59197348602238	-4.21184729636280	-2.18514020337001
H	-6.84992763392173	-0.17167305029171	3.10986616468492
C	2.00356927823074	2.47536173618465	-3.19048883140261
C	-0.17670811639931	1.39467835990183	-3.86966338699493
H	2.65661834709212	2.52521090402343	-2.29138600315323
H	2.61506228865548	1.99255945782819	-3.98529663517461
C	1.60469475011180	3.88602644741161	-3.61194470551630
H	0.99855029976369	4.38197657186308	-2.82894349988893
H	2.51960187151486	4.49012256344127	-3.77604268186247
H	1.02894999499834	3.89580351048476	-4.55958582720137
C	0.33241969155952	0.67832041997642	-5.11915085714345
H	-0.99514599335478	0.80968000241606	-3.41336880903604
H	-0.61236690197714	2.38276871056638	-4.13260810515634
H	1.12141405952653	1.25492215508015	-5.64291764966721
H	0.74531889209694	-0.31786144962405	-4.86018713596284
H	-0.50162733261710	0.53069943874398	-5.83444689695337
Ni	2.71510924830219	0.92959689534597	-0.43129517977475
H	-2.27330382047722	-1.06372834053371	5.87582652353714
H	-3.31150463058591	-1.23696438002347	4.39997648975935
C	-2.41357497552867	-0.73940694982952	4.82285514589624
H	-2.55225280183484	0.35610682223083	4.79306484343394
N	-1.24923602034822	-1.03977471772722	4.02427382440278
O	-0.97896124937970	-2.39572933788336	3.85717578665268
H	1.59274591425847	1.15807048818907	2.31781429825721
H	0.96511033795392	-2.36215381724767	4.67148853612570
C	3.97860685571475	-0.06338353412989	1.22123432579443
C	1.51452162336770	0.12616434280851	1.93454852799871
C	2.49213257648077	-0.34113927349840	0.95339574755239
C	-0.01826929432106	-2.86077583260293	4.81338783334744

C	4.86314119266125	-1.08789562159004	0.52298806746224
C	-0.53586824603000	-0.10585100645289	3.29556561620912
C	0.50438097056985	-0.65744832140588	2.39354294167692
H	-0.37039645314878	-2.70642749911164	5.85635193033119
H	2.29545410369069	-1.36799246654550	0.59261207735918
H	0.42020901104287	-1.70587338373390	2.07428778085519
O	-0.77430168737681	1.10637902628215	3.40425304838605
H	0.08641366677624	-3.94502096017809	4.61708492379085
C	2.91329017270079	4.02908849028312	4.06520518524700
H	2.48720536459901	4.66199421094059	4.85867502166675
C	2.55148449499469	4.23621658282634	2.72483018484073
C	3.83032785978311	3.01352501290216	4.38780881275956
H	1.84106470781322	5.03630903900768	2.46491085649147
H	4.13066071562873	2.85398548083833	5.43507806843058
C	3.09381219770781	3.42618243357610	1.71413366938533
C	4.36393276706731	2.20148414221530	3.37638063196328
C	3.98929797602484	2.39009300964053	2.03198670373922
H	2.83986885773828	3.57722783045067	0.65404671339214
H	5.08545983035856	1.40908599211567	3.63857891707423
C	4.53185043415625	1.49321723135153	0.91408065699639
O	4.30836991831595	1.97275189149514	-0.35642871762815
H	5.62473259599822	1.33375614465163	1.09941046999527
H	4.16799491093085	-0.18304652297098	2.30779736880186
F	4.78550078470153	-0.99828997893647	-0.83047466204929
F	4.51594976809634	-2.35967766484170	0.85637010246196
F	6.16663999701867	-0.93796175692319	0.86068747927264

TS (R,S)-17a
Electronic energy: -4915.8440 Eh
Enthalpy: -4905.9714 Eh
Gibbs free energy: -4915.0015 Eh

P	0.67791688068184	1.47028295578461	-0.31860139669613
O	-0.65248408058977	1.23327736856355	0.71092863906669
O	0.31620393425753	0.25302575683489	-1.43076138486384
N	0.41082707638229	2.76528298044262	-1.34741087388658
C	-2.31515858160078	0.14234917497482	-0.57134819186885
C	-1.44708316646801	-1.06846859736837	-0.52967763779726
C	-1.90081986477325	1.29126358017185	0.12527628033073
C	-0.11794622674776	-0.98634513828895	-0.98712969156045
C	-2.67305657056355	2.48478036464718	0.22921387270145
C	0.76960060961774	-2.10379001426338	-1.04690257135429
C	-3.56106410176573	0.16737437407339	-1.27111698898507
C	-1.94427101756458	-2.32028704814842	-0.05284823659088
C	-3.90890405719596	2.48839298109757	-0.50343219044009
C	0.22832648044596	-3.35089454363524	-0.57961894633811
C	-4.31768289928506	1.33995876346685	-1.22326385156680
C	-1.10434214976680	-3.42986588917632	-0.11578032139180
C	-2.27728715758478	3.68009022379066	0.97416431794273
C	2.17382109225806	-2.05510973268161	-1.46126203188185
C	-3.09496452762039	4.86108320505604	0.85341681751525
C	2.98980634258120	-3.22705416134409	-1.25558059279130
C	-4.05726638570669	-0.98445135927553	-2.06623508141993
C	-3.28965910031015	-2.47046916787722	0.55561025780296
C	-4.70951902483769	3.68067820525189	-0.55214890962163
C	1.06906410932147	-4.51018848699990	-0.46780444540395
C	-4.30508681805209	4.82736292285240	0.07871278091860
C	2.40447939613207	-4.44163070365911	-0.76038490832350
C	-1.14525092245131	3.75933973500588	1.83711321917617
C	2.81009285786513	-0.92357323124974	-2.04913407583805
C	-2.72086204943325	6.05654502218588	1.52426465813362
C	4.37737203187832	-3.19388565360386	-1.55828374312474
C	-5.40972473027878	-1.37814658597902	-1.96444496760030
C	-4.08942967772226	-3.58814440430068	0.23098311993406
C	-3.21298495392320	-1.69410876153127	-2.94915363354713
C	-3.77615638454052	-1.54297953150306	1.50370583762643
C	-0.80662650959936	4.94253811981132	2.49030132701774
C	4.16936070577894	-0.92535455220420	-2.35354031214712

C	-1.58546043977101	6.10854843811204	2.32504062391585
C	4.97121765302124	-2.05508742797146	-2.08766725694278
C	-5.90104471517352	-2.45895850520778	-2.70871607439492
C	-5.34762776327694	-3.76665274572302	0.82141096735251
C	-3.70439973128089	-2.77613385068194	-3.69226434391903
C	-5.03563991513313	-1.72198564748059	2.09235932476664
C	-5.04867214323549	-3.16671200738445	-3.57144313962891
C	-5.82921987255281	-2.82984732182611	1.75061116994807
H	-5.25470720655739	1.39513577900088	-1.79746802285119
H	-1.45020853412496	-4.38919766565129	0.29330147532547
H	-5.64798927575024	3.65456770486463	-1.12700741169016
H	0.61719453156771	-5.44263019787068	-0.09725899279989
H	-4.91154132791689	5.74452376318822	0.01756117377561
H	3.05543126526995	-5.32062502274031	-0.63344586209622
H	-0.52844934667590	2.87230435720855	2.00610477289224
H	2.22502307200337	-0.03026758471616	-2.28083713460228
H	-3.36086346952355	6.94466175152561	1.40320766187318
H	4.97316562859470	-4.10034105604477	-1.36802442295664
H	-6.07153628351813	-0.85040527892789	-1.26127211432403
H	-3.73082618761082	-4.30589157851391	-0.52215849716854
H	-2.16384741910682	-1.38571816248178	-3.06335908929471
H	-3.14553932348185	-0.69940249260031	1.81629678800971
H	0.07947136294789	4.95778928883913	3.14332017359644
H	4.62167013095757	-0.02619144087853	-2.79388029763792
H	-1.30707441096804	7.04190020329089	2.83756376019117
H	6.04888532129791	-2.03454938284627	-2.30940334861888
H	-6.95451997100285	-2.76014674333692	-2.60241258430783
H	-5.96249886731885	-4.63658678471446	0.54364707327653
H	-3.03196224831243	-3.31491427996088	-4.37746250988528
H	-5.39406023383086	-0.99334353842645	2.83571823035276
H	-5.43156268580527	-4.01988284017640	-4.15232037590297
H	-6.81953468262631	-2.96551072541980	2.21206806728273
C	-0.48817743725805	2.73919975394636	-2.51675170529416
C	0.98254813906387	4.06209978027585	-0.94802226045753
H	-1.32212423600702	2.03804515910942	-2.32261155643122
H	-0.94747724702738	3.74893121607568	-2.58545811730227
C	0.19446571174219	2.36209656795064	-3.83189859639905
H	0.60426076582518	1.33453955121047	-3.77762146479977
H	-0.54348513332763	2.39154800396697	-4.65972851927088
H	1.02129548858560	3.05292387045521	-4.08551492714786
C	1.87862942753204	4.71461814955336	-1.99635878211561
H	0.15290320985334	4.74901450402453	-0.66581016343104
H	1.57004672150209	3.88665272309193	-0.01973132047956
H	2.70193013057475	4.03801278470276	-2.30237683723761
H	1.31539791147510	5.00698943180617	-2.90511581613480
H	2.32904524491414	5.63529228411210	-1.57400966708859
Ni	2.34504426953366	1.61964680838783	0.84835818888939
H	-2.85464677383099	-3.96256633648310	3.22749534896231
H	-2.67353077069327	-2.57206133463658	4.36613326467651
C	-2.19129359676145	-3.48862833027133	3.98207989551677
H	-2.02147843435981	-4.19637708700852	4.82060249756351
N	-0.94205561505238	-3.08859871085728	3.38021871896693
O	-0.21934298162296	-4.11802689406519	2.77934786595887
H	1.06702520473456	0.32872892424564	3.15635714909370
H	1.28116493868441	-5.44605486796638	3.09963860561927
C	3.75118479199330	0.46180859617733	2.22397625370791
C	1.37026474142493	-0.42979123156092	2.41198021255293
C	2.50142346030726	-0.12506394393474	1.54555734947375
C	0.82346668122872	-4.59513158774971	3.63962739720929
C	4.74080398047438	-0.67160420013620	2.45858719823657
C	-0.54877706546782	-1.77714020686505	3.18782166062263
C	0.66789893838434	-1.59175712422141	2.36412058420732
H	1.59405242060369	-3.81207263669189	3.81241236551056
H	2.72420536608464	-0.89775215339235	0.78578620904649
H	0.96919839814744	-2.39970136384562	1.68407056746672
O	-1.19106135272184	-0.83826242990680	3.68438876817348
H	0.41983289752393	-4.94804597590674	4.61325973732189

C	6.87210570175703	1.63396792618516	-2.05332863887708
H	7.48398433171385	1.61279319380615	-2.96838174812634
C	5.62623371526955	2.28119347892186	-2.04691046681905
C	7.34107355939140	1.03016502236717	-0.87441217047180
H	5.25387110004931	2.77660434868195	-2.95754504174743
H	8.32583633720504	0.53770696284478	-0.86201826014882
C	4.85615343711855	2.31002789317232	-0.87553263276540
C	6.57230159228207	1.07143337172667	0.29858785790280
C	5.30901348273088	1.69811450604317	0.30992730664863
H	3.89389462962319	2.83976153117684	-0.85016630438073
H	6.97406933919042	0.62560142310784	1.21898675274473
C	4.47898083754823	1.86280167081403	1.58771706088235
O	3.57186739735156	2.90579821374899	1.50929966308901
H	5.19326339713453	2.02511745587004	2.43327866335455
H	3.46749562880328	0.81950721370355	3.23470079200534
F	5.11571158828359	-1.29761996090974	1.31827217498651
F	4.19710828504193	-1.62111980863208	3.26653245696921
F	5.87188397351847	-0.24069331159031	3.08054650290317

TS (S,S)-17a
Electronic energy: -4915.8499 Eh
Enthalpy: -4905.9821 Eh
Gibbs free energy: -4915.0091 Eh

P	0.93534195626708	1.15208775866752	-1.12271548217969
O	-0.27128721681773	1.45552251897005	0.03602603332750
O	0.11346509841814	-0.07727712033398	-1.93894196547465
N	0.89093137167448	2.32075795874468	-2.31675340697602
C	-2.27855918608624	0.45280308312975	-0.74236408600483
C	-1.61875846344994	-0.86843120123300	-0.53441336294732
C	-1.57540507647007	1.62254760029945	-0.38817998799503
C	-0.38021311851066	-1.10900457039639	-1.15523702957256
C	-2.13512356648597	2.93541889707878	-0.43033023813312
C	0.35407409808028	-2.32512267769448	-1.04039363009823
C	-3.60820709878610	0.57312546319637	-1.25378728807127
C	-2.20641627537058	-1.89973178621439	0.26079095546707
C	-3.47739465092790	3.01760312234189	-0.93893831782529
C	-0.27014309766644	-3.34584300196056	-0.24417133575433
C	-4.16704211228240	1.84851273319729	-1.33913389573772
C	-1.52622324717287	-3.11265366948285	0.36295368638138
C	-1.44976966334852	4.16698141579069	-0.02791990572055
C	1.66901303685950	-2.58518975174256	-1.62927669998172
C	-2.13137366885836	5.42343426730767	-0.22144652388367
C	2.32340409888056	-3.83053487065825	-1.31379898310118
C	-4.39506906362166	-0.59606571463680	-1.72115865000693
C	-3.47717255866266	-1.71744956716839	1.00436652830461
C	-4.11561591150517	4.29584633432809	-1.09009568993519
C	0.40512514176627	-4.59086960945323	-0.00940289729288
C	-3.46386221653367	5.45296244204043	-0.75728551817003
C	1.66180979936282	-4.81334660129202	-0.50237105333215
C	-0.14766092218346	4.22526399987261	0.54889639844180
C	2.35856227458615	-1.70032373128968	-2.51071390052540
C	-1.49087762567677	6.64402045681740	0.12408595806244
C	3.61855755028789	-4.10585813577018	-1.82913398228826
C	-3.81740875987614	-1.59833272651329	-2.53216619030055
C	-3.73406182620341	-0.54609062409825	1.75082594232972
C	-5.76006686961325	-0.71063355512072	-1.37745123683545
C	-4.44744102966085	-2.74222547583582	1.00628885678415
C	0.45612336714687	5.43524205765027	0.88409754203688
C	3.62846560281767	-1.99627418569833	-3.00304272866645
C	-0.21057124198300	6.66003968215845	0.66431916251880
C	4.27662644088903	-3.19920853778366	-2.65233416589593
C	-4.57915936292442	-2.69095881860577	-2.96812211467272
C	-4.93182777477344	-0.40056237545085	2.46363302140454
C	-6.52138943253270	-1.80274615985375	-1.81393451753099
C	-5.64457965185992	-2.59844013548494	1.72059454636574
C	-5.93229778947960	-2.80129760208110	-2.60614927356060
C	-5.89463607623654	-1.42451040862522	2.44894179896064

H	-5.17425166197795	1.96425846113400	-1.76644948179560
H	-1.95144745686412	-3.91013907079272	0.98956227358136
H	-5.13999029174537	4.31992536846460	-1.49229061396766
H	-0.10068420461408	-5.34841199831556	0.60782269831850
H	-3.95239801748959	6.43110048396766	-0.88724360653491
H	2.19041648717350	-5.75507595950550	-0.28925211626288
H	0.39922888271484	3.30212464302502	0.74531070003844
H	1.87167278762013	-0.78482959908113	-2.85274570460258
H	-2.03925104432720	7.58444021863531	-0.04289543662463
H	4.09135154137581	-5.06399428470378	-1.56228759724639
H	-2.76413298318176	-1.51177617410270	-2.83489505850491
H	-2.97830661287902	0.25117943347243	1.78481654939562
H	-6.21743719355024	0.04963135485145	-0.72664410409478
H	-4.27251138293598	-3.64906734176450	0.40809358663422
H	1.46270597583358	5.42077163637913	1.32682078703478
H	4.12414301445398	-1.27639920225705	-3.67170856232158
H	0.27378460667271	7.61331669889721	0.92497576609292
H	5.28423791943882	-3.42147787615811	-3.03452035736848
H	-4.11278077573054	-3.46033535463533	-3.60256913476462
H	-5.11290779969299	0.51943884918071	3.04091303915731
H	-7.57936595181143	-1.88199316058532	-1.52035839274076
H	-6.39451069607751	-3.40402118492990	1.69624551984488
H	-6.52735960559019	-3.66319884003607	-2.94518517884419
H	-6.83715075965189	-1.30662835480325	3.00545957589136
C	-0.17563419136399	2.41724412194818	-3.33131980724476
C	2.12448999011430	3.09770718457038	-2.52634324627062
H	0.17229654082173	1.94446654193165	-4.27766960083537
H	-1.03073236307773	1.80954321813223	-2.98379677854158
C	-0.63231393269061	3.84897595217129	-3.59855608699350
H	-1.47887831135707	3.83433438098519	-4.31380936617419
H	-0.97543165973352	4.34312314110412	-2.66879225677171
H	0.16901221009612	4.46968571623479	-4.04711997298904
C	2.89697752509498	2.69696570972970	-3.77955597685862
H	1.87162232224769	4.17936776783904	-2.53390777088365
H	2.76431090379899	2.93175075444233	-1.63599525628318
H	3.82877710313211	3.29238055990300	-3.85485131887637
H	3.17941441577159	1.62487929545807	-3.73043742380784
H	2.31529907444720	2.86454096175043	-4.70859511015466
Ni	2.76392422459670	0.76738662871923	-0.31564440998733
H	0.51591294368220	-4.81411799620002	3.61236774350648
H	-1.20535611687506	-4.73001411997757	3.05453077702301
C	-0.44530504866123	-4.28268726970659	3.73149061395897
H	-0.79524755825417	-4.38377384792459	4.77983605481420
N	-0.20833012884438	-2.89933929515409	3.39200438991043
O	-1.34698924333628	-2.09475432263346	3.36693580965634
H	2.99896313337600	-1.19164802714583	1.84812129387061
H	-1.71138314990219	-2.23697598513387	5.44360982833909
C	3.72586142525159	1.45899519767356	1.64954450663572
C	2.13261197646410	-0.50951115634406	1.88828722122076
C	2.32703279975864	0.88256631502517	1.51237733300188
C	-1.58171229049602	-1.48122295623614	4.63872102698144
C	3.72524972239285	2.97634677359208	1.72414505393331
C	0.94196084444378	-2.46320846110441	2.75187477512062
C	0.95988548210599	-1.04053117865010	2.33735536943221
H	-0.75722706531013	-0.78629405823570	4.91033691120421
H	1.50041589860789	1.56474321253321	1.77549591394213
H	0.04834078080360	-0.43270449149939	2.42154559955301
O	1.90730477609720	-3.22824011025945	2.60727108144310
H	-2.52466061814283	-0.91502618549787	4.51580844181405
C	6.34946011402047	-2.83899222631578	2.24554492437579
H	6.69101622111324	-3.78656498308022	2.68979133573369
C	5.55778134452901	-2.84578324308755	1.08611993339516
C	6.70959369424173	-1.61396073113924	2.83343526937851
H	5.27073563123478	-3.79833274313666	0.61519056126838
H	7.34208505227867	-1.59847746044553	3.73466417470888
C	5.12437989250827	-1.63761156157813	0.51900403946191
C	6.26395871931876	-0.40805793329287	2.27211298687481

C	5.45669181663033	-0.41034859202021	1.11843873953785
H	4.51336118731467	-1.63575801327213	-0.39468600194262
H	6.54734360035315	0.55210655703442	2.73597261404188
C	4.94513251132550	0.90402500425329	0.52732927834074
O	4.60076983225416	0.82196049495781	-0.79389472170127
H	5.70912575650892	1.69825790733440	0.71937738396975
H	4.17380031704096	1.10459192983891	2.60041574755681
F	4.94361336209090	3.45667943073818	2.07009400494013
F	2.84296450618395	3.43964566267495	2.65297149559566
F	3.38278396735267	3.56964060504634	0.54722134954482

TS (S,R)-17a
Electronic energy: -4915.8388 Eh
Enthalpy: -4905.9760 Eh
Gibbs free energy: -4914.9975 Eh

P	0.70111906968422	0.79810518236952	-1.17039058242319
O	-0.41587047523266	1.12197858534850	0.07598105038084
O	-0.21654705516185	-0.38571273592975	-1.94492061570386
N	0.62046396559096	1.98552081348625	-2.34796445175947
C	-2.47495555011294	0.09017563675975	-0.51494065622587
C	-1.77607045652967	-1.21899727890682	-0.37051943117110
C	-1.75249249249841	1.26843448540319	-0.23165641693738
C	-0.60412920557252	-1.43123624730138	-1.11582752855843
C	-2.33358033517311	2.57258624699714	-0.21954560731239
C	0.18867618379140	-2.61263888411454	-1.05729675846871
C	-3.85233262280469	0.19070937249832	-0.88272852937145
C	-2.23750667050268	-2.25674900038123	0.49922286591274
C	-3.72228070731064	2.63655926214894	-0.58767428881733
C	-0.27758957625231	-3.61827918541779	-0.14522585941720
C	-4.43618779717723	1.45749370551547	-0.90835624221164
C	-1.47841611154296	-3.42569458337681	0.57603249951806
C	-1.62399747260519	3.81442595365384	0.10123909312848
C	1.43220384769612	-2.83654933581505	-1.79570860715478
C	-2.32966228346448	5.06346092020109	-0.04402262675180
C	2.21378028718743	-4.00194764044908	-1.46964802677239
C	-4.66461975762121	-0.99617971795115	-1.25191360959599
C	-3.45870468736506	-2.13919904987422	1.33574060407503
C	-4.38380518128287	3.90837489260590	-0.68583380837023
C	0.50853365277872	-4.79596938696024	0.09011683565702
C	-3.71036206869274	5.07537962746801	-0.44120337748777
C	1.72114345757057	-4.96322722207793	-0.52220366689578
C	-0.27356901982097	3.88871751451257	0.55081573801385
C	1.93039193603040	-1.98994648056363	-2.82913553614024
C	-1.66469710042992	6.29286009335234	0.21318595085786
C	3.46343724487076	-4.22051962510135	-2.10938772614909
C	-4.16165263215120	-1.99445749760811	-2.11559519287491
C	-3.76422695336494	-0.96527030407323	2.05864835034135
C	-5.97730465313996	-1.13655371991824	-0.75042137272513
C	-4.33450053118988	-3.24237531718546	1.44632114030581
C	0.35535949116037	5.10700014262705	0.79923824479495
C	3.15141695264870	-2.23750923453438	-3.45107276608290
C	-0.33614925047607	6.32480584349497	0.62050767116042
C	3.94011893276839	-3.34556221479542	-3.07817311233143
C	-4.94290364890182	-3.10926827140382	-2.44918562525562
C	-4.91619345138015	-0.89422925315162	2.85267069764696
C	-6.75780670495458	-2.25100038315860	-1.08427392951081
C	-5.48611485098213	-3.17209950232196	2.24160001648735
C	-6.24125016581424	-3.24573122384270	-1.93009481424426
C	-5.78478442716594	-1.99424838735352	2.94549279858744
H	-5.48519024322841	1.55827479873468	-1.22487161150097
H	-1.78390233677354	-4.21448986452358	1.27857411844799
H	-5.44459516970069	3.91982306528559	-0.97972742721387
H	0.13118266703737	-5.53829990525301	0.80855068574834
H	-4.21774970568916	6.04795641673604	-0.53603801315231
H	2.34072934726327	-5.84793751553406	-0.30830389028694
H	0.28992881496711	2.96938650894297	0.71760537016927
H	1.33472390066738	-1.13987781859743	-3.17018235998911

H	-2.23251647271550	7.22784637405336	0.08494633921432
H	4.04540404214578	-5.11160305961916	-1.82589362496896
H	-3.15243979025330	-1.88883675585555	-2.53846038887925
H	-3.07843597032692	-0.10950106133503	2.01806481075182
H	-6.37120881765990	-0.37992445576136	-0.05558072599079
H	-4.12483462037684	-4.15465420861892	0.86802160872888
H	1.40163595974516	5.10444232980487	1.14012925308835
H	3.50210471946685	-1.55090446367380	-4.23635595939052
H	0.16615074981945	7.28520269813649	0.81191810199499
H	4.91344997966377	-3.52365049694854	-3.55990706712270
H	-4.53526340982102	-3.87623541083878	-3.12564423270450
H	-5.13247891050874	0.02846166941978	3.41319721013653
H	-7.77188321615557	-2.35095294965929	-0.66780782468791
H	-6.16297877828551	-4.03838002356505	2.29969575181198
H	-6.85060931658286	-4.12566176119594	-2.18769220217385
H	-6.69153775736387	-1.93401652429524	3.56699608830008
C	-0.36097169952262	2.04038009239683	-3.44591801684471
C	1.78933248076376	2.87977062823475	-2.44362962322609
H	0.18115508227803	1.88366088912850	-4.40607135032025
H	-1.04623060393489	1.18009454040028	-3.33587180127754
C	-1.16175367694703	3.34036249123513	-3.51053451489230
H	-1.84762611294781	3.30409167025727	-4.38104928855076
H	-1.77059274046516	3.48805936029097	-2.59911850824618
H	-0.51284812598954	4.22999114650949	-3.63952147323884
C	2.90382318193200	2.34511629750628	-3.34160357436887
H	1.42627148671337	3.86608739949036	-2.79932725587634
H	2.17867085891936	3.06133014945150	-1.42291002249302
H	3.72429612854916	3.08692187673697	-3.42130608956338
H	3.32850241696132	1.41247803541161	-2.91498794427433
H	2.53956933129706	2.13102189368852	-4.36714903833870
Ni	2.53486459799362	0.25297812960089	-0.45102441006241
H	-0.67514433626300	-4.39332834494826	3.99215014954600
H	-2.34635367906977	-3.71521503064497	3.79501417315827
C	-1.35661558261182	-3.57268691670723	4.28021768742111
H	-1.48958294363285	-3.58025700692439	5.38255548385415
N	-0.74682757544284	-2.34273161338590	3.83460842061180
O	-1.50041360079944	-1.19174453678268	4.05271006627839
H	2.61691701959373	-1.57665664103985	1.80981662197094
H	-1.31827542589389	-1.28153153965108	6.15537933071250
C	3.79504743269596	0.90382018459272	1.30930344793046
C	1.92424367600079	-0.72044057244715	1.90220437407980
C	2.32435680904114	0.58250164655459	1.39163294786517
C	-1.16518272250010	-0.58244179455567	5.30490580082852
C	4.08673356895141	2.38777906777829	1.11206122671031
C	0.38365832444231	-2.28171648574089	3.03630861773183
C	0.72917079142194	-0.93837347447851	2.52095805707932
H	-0.11532265522807	-0.21555235899343	5.30824611325410
H	1.65902930494009	1.41798363910004	1.66322285918231
H	0.01142083641113	-0.11586517969176	2.64500518873278
O	1.05444643095665	-3.30050537404941	2.81207009925185
H	-1.85880584636329	0.27386635950168	5.40820167380209
C	8.79556712819391	1.38761688153116	0.17366571437268
H	9.82595795674623	1.77549173014256	0.16728910754616
C	7.97062246285894	1.55619887874629	-0.95089787208736
C	8.30400342025079	0.71250505450150	1.30458295564458
H	8.35597540089271	2.07439546271707	-1.84289505580507
H	8.94999757000076	0.56568115123609	2.18400532461766
C	6.65828627423143	1.06109636159671	-0.94328430016537
C	6.99148610738361	0.22026102440806	1.31006123707867
C	6.15825714065886	0.39683035057816	0.18961852120045
H	6.00036302557653	1.16635741201478	-1.81809520799936
H	6.60938140961883	-0.31600764543605	2.19503569652492
C	4.74746942793253	-0.16131378296405	0.17078640515288
O	4.23459953154892	-0.35682514996555	-1.06361826059179
H	4.70961916848633	-1.07679847364439	0.81685179937522
H	4.31073824570036	0.57936498988140	2.23461044615667
F	5.25071880608512	2.75297291794250	1.68771742626001

F	3.11993983383619	3.18808746222656	1.65704698533316
F	4.16198786731010	2.74293256594786	-0.20215715151997

(R,R)-Q
Electronic energy: -4915.8843 Eh
Enthalpy: -4906.0050 Eh
Gibbs free energy: -4915.0415 Eh

P	0.47100825180723	0.91052398714604	-1.07761637196124
O	-0.78463222106201	0.75934341355674	0.03704971903584
O	-0.11186569761891	-0.20481498765198	-2.20648025885651
N	0.27147281958655	2.30337239387575	-1.98076046851788
C	-2.55345368389014	-0.41734169360911	-1.00079879035631
C	-1.63716093939812	-1.59199406803819	-1.03525903887649
C	-2.09193353088532	0.77099572313105	-0.40836419873219
C	-0.40522758472126	-1.46534646218827	-1.70163421762481
C	-2.89507607321983	1.93513845671403	-0.22448945923426
C	0.50803049280936	-2.54219002911659	-1.90363546131063
C	-3.88506849603761	-0.46886625421522	-1.51581208296919
C	-1.98308404292057	-2.84358099622191	-0.43923420435542
C	-4.22886133260580	1.85355183809044	-0.75494674038860
C	0.12536457168407	-3.79273738858327	-1.30384889620077
C	-4.68241633704143	0.66852144463582	-1.38252000485736
C	-1.08989474078767	-3.90509054936932	-0.58670663157910
C	-2.46537346189443	3.16760136444073	0.43800627623151
C	1.76193003978723	-2.46670912479024	-2.65472485346158
C	-3.38029026145478	4.28169360892220	0.47486339910295
C	2.59390445109954	-3.64176747055075	-2.71616817672499
C	-4.43397630830202	-1.67112700376952	-2.19240160266385
C	-3.23394917364645	-3.03641833406298	0.33700341002781
C	-5.10247793388950	2.99221483237826	-0.68219363105994
C	0.99084089984712	-4.93434435122647	-1.40444772468604
C	-4.68959925491698	4.16345184997696	-0.10483100937776
C	2.18135235076218	-4.85841540610226	-2.07579291027468
C	-1.21107887915349	3.34108010023167	1.08960771382602
C	2.22369553354350	-1.31122951079124	-3.34970618312876
C	-2.99859510931043	5.49525341620198	1.10825137183051
C	3.83015109936245	-3.60372539626458	-3.41378715067327
C	-5.74057973155511	-2.11346471993437	-1.88951767696963
C	-3.99774308203984	-4.21149343632498	0.16281040100715
C	-3.68694013137111	-2.38531265932836	-3.15588628291879
C	-3.68210168941663	-2.07384458063801	1.27022297576662
C	-0.86830904192341	4.53447391448123	1.72144556369933
C	3.44128550396830	-1.30160960247494	-4.02648081951476
C	-1.75910419226787	5.62956672870018	1.72394438371740
C	4.26143826294749	-2.44960283364712	-4.05578854954285
C	-6.27956692511135	-3.24435492085087	-2.51699703289994
C	-5.18110115151960	-4.41430192093462	0.88542950524101
C	-4.22639438449621	-3.51658160872473	-3.78319450427172
C	-4.86836373764771	-2.27599177772549	1.98965930879704
C	-5.52248477118952	-3.95435195590976	-3.46276110614123
C	-5.62557449359540	-3.44404594829177	1.79795408172808
H	-5.69771902655138	0.66308813420693	-1.80646074359400
H	-1.31798942203657	-4.86851786971395	-0.10652674639764
H	-6.11248679171032	2.89988430887317	-1.11020522812855
H	0.67319838062908	-5.87102318299605	-0.92140412901995
H	-5.36170128605828	5.03461320797471	-0.05972520363916
H	2.84598922183174	-5.73371671335215	-2.14148766372092
H	-0.50154611151918	2.51273886565195	1.12996527345482
H	1.60614216961823	-0.41069221759149	-3.37553034474013
H	-3.71856322980330	6.32878857728844	1.11267687443573
H	4.44562568390011	-4.51682045085395	-3.43393249662396
H	-6.32399474804071	-1.58318050319729	-1.12193650192821
H	-3.67570677861247	-4.95743541334690	-0.57919518473553
H	-2.67817966128783	-2.04146352237707	-3.42534392283116
H	-3.09216945449026	-1.16305041609791	1.44627212261953
H	0.09767280743099	4.59352012564828	2.24445695423362
H	3.75937716931936	-0.38412514557216	-4.54529003440197

H	-1.48235115411942	6.57238835626953	2.21992295072526
H	5.22555534686867	-2.43294679219226	-4.58628931507794
H	-7.29390995317239	-3.58211878623098	-2.25455194976722
H	-5.76906830452647	-5.33061421309292	0.72265679519853
H	-3.63023187096243	-4.05766620396186	-4.53401703458811
H	-5.20012016751430	-1.51263975670855	2.71056848186773
H	-5.94234151773289	-4.84627702235699	-3.95276883586620
H	-6.55928712722270	-3.59776094186374	2.36047956500196
C	-0.76323951953558	2.53226253658152	-2.99717977336512
C	1.26856987007459	3.35510528078193	-1.70947510825360
H	-1.50850206316705	1.71889226595247	-2.91503716219567
H	-1.29950904725513	3.47464309094318	-2.74639611272496
C	-0.21178139981529	2.58447182416224	-4.42092247165986
H	0.28094657025547	1.62723877431540	-4.68643796371405
H	-1.03460261414188	2.76272848219301	-5.14271835907040
H	0.52651357572392	3.40251022362919	-4.54870755990222
C	2.67746258107289	2.97723500150039	-2.16701139105623
H	0.93181423670048	4.27188820864042	-2.23459478768275
H	1.26640022763083	3.61035512277535	-0.62669972737658
H	3.01260949688776	1.96630896182254	-1.80232559351490
H	2.73771254085684	2.89478974912586	-3.27104093325090
H	3.43559960350932	3.70170470383580	-1.81049763541545
Ni	2.41539277238188	0.89064910194449	-0.27544333606184
H	-2.80773327100855	2.89197780171551	3.87986384670403
H	-1.35612586824402	3.21097075969338	4.90977407820033
C	-2.08701149970666	2.44355819333796	4.59817247772181
H	-2.63736271441351	2.07604123999003	5.48886403651591
N	-1.34009998627267	1.37476142914998	3.97718798315809
O	-2.12251706929100	0.32192454956153	3.49323180209865
H	2.38403047231363	1.31161306717795	2.54047467539320
H	-2.84472316231869	-1.50711853036529	4.01132616140651
C	3.70513619504539	-0.91473727662338	1.37238156310404
C	1.73293931537215	0.51609792594334	2.14310147755466
C	2.26742070659887	-0.40952378849410	1.14564341145539
C	-2.17086979188304	-0.74578097028813	4.44813444866294
C	4.06893331544344	-2.06802866577506	0.44925631157808
C	-0.03315759339352	1.51424517606010	3.53037585465309
C	0.46303159766716	0.44860339740390	2.63848456681981
H	-1.16364997636582	-1.18859140240801	4.60702785218909
H	1.56402573038808	-1.22789593843267	0.89578505604789
H	-0.22682164401447	-0.33943338458018	2.31119093060689
O	0.63971379012112	2.50200100983135	3.87172483260944
H	-2.58367935337833	-0.40238193485357	5.42145276954315
C	5.36838129759528	2.53373249610492	4.83696160764944
H	5.53553108066637	3.10494713353716	5.76309524721836
C	4.88558498301689	3.17619806262494	3.68602187109723
C	5.63982143002779	1.15501720152854	4.79850803285750
H	4.67262768466351	4.25643065402654	3.70624755244383
H	6.02560604385517	0.64367596509275	5.69430574682045
C	4.66977629291056	2.44499328974102	2.50707351882785
C	5.41872785809984	0.42605079181540	3.62059218312407
C	4.91830160868710	1.06201288181860	2.46747198967375
H	4.28859307536713	2.92770384743035	1.59512285774113
H	5.64057745974851	-0.65367324420432	3.59698262710211
C	4.67729097389762	0.27374274582786	1.17792378006449
O	4.16613054286048	1.06013041871170	0.13562176672468
H	5.67701530683890	-0.12840789025779	0.87588598372009
H	3.77162814555064	-1.36202232702292	2.39024563367188
F	4.21902584808790	-1.70085950385609	-0.84382026665195
F	3.12885433393358	-3.05157956172797	0.48114658598113
F	5.24429064040311	-2.63460055479201	0.83556709749795

(R,S)-Q

Electronic energy: -4915.8795 Eh
Enthalpy: -4905.9984 Eh
Gibbs free energy: -4915.0370 Eh

P	0.27461180892190	1.55589338137672	-0.51590806066683
O	-1.04484691900236	1.33198584099932	0.51979335274067
O	-0.14622856166867	0.37396582400389	-1.64120228071296
N	0.04320207255673	2.90318576549439	-1.48553639157468
C	-2.72817745564251	0.18670682572860	-0.69646663806958
C	-1.82927751016626	-1.00171751272797	-0.67553465754820
C	-2.31154460604334	1.35215687734362	-0.02738111230498
C	-0.51840549716279	-0.87772013463216	-1.16779646315206
C	-3.10659832014893	2.53009930199008	0.09491837475611
C	0.42566411749467	-1.94565301182460	-1.19299910210350
C	-4.00213041208996	0.17782264420888	-1.34475754005475
C	-2.26050403781716	-2.26495981937155	-0.16424320656015
C	-4.37322863858522	2.49624238834104	-0.58207382370654
C	-0.03928058072648	-3.19975039605919	-0.66972169453681
C	-4.78517609397620	1.33128258706785	-1.27292558600481
C	-1.36681320637350	-3.33332772883951	-0.20258898975513
C	-2.70332479416564	3.74809664243682	0.79897037780667
C	1.82473633091012	-1.82198018556028	-1.60178322840275
C	-3.55073178282085	4.90959282471999	0.69465032924964
C	2.72340062873720	-2.90641659189328	-1.29615112674991
C	-4.50312514012313	-0.99210425715943	-2.10958980284669
C	-3.58983862200062	-2.46016052430860	0.46553266503907
C	-5.20292659737046	3.66910974679172	-0.61087317295121
C	0.87915157876569	-4.28922165445969	-0.48820118542753
C	-4.79575292321801	4.83550601347990	-0.01941005806601
C	2.21531081022113	-4.13175250108713	-0.74362376086377
C	-1.53381815869988	3.86888502282322	1.60594012353148
C	2.37305748917978	-0.69928178037868	-2.28655051257732
C	-3.17010071237219	6.12690059277486	1.32144673001451
C	4.11175173003376	-2.77773478589105	-1.57006434443458
C	-5.84363743013324	-1.40972378854972	-1.95700771920145
C	-4.35648962803016	-3.60432871124525	0.15553554776749
C	-3.67611661957942	-1.69655700517939	-3.01280493987100
C	-4.09107498714285	-1.54501508229175	1.41796205696758
C	-1.18836658376007	5.07336251469697	2.21551108659545
C	3.73273331672200	-0.61560468155147	-2.57391233316100
C	-1.99792484326620	6.22042964079211	2.06284571722028
C	4.62139931256196	-1.64048587921210	-2.18453716634825
C	-6.33913145551259	-2.50886365054866	-2.67077280956825
C	-5.60011831296532	-3.82049153273560	0.76404270221772
C	-4.17183534227030	-2.79677692734849	-3.72565846476466
C	-5.33606480928150	-1.76202896449042	2.02451531017201
C	-5.50325975869186	-3.21130062420044	-3.55373209965157
C	-6.09835576709392	-2.89593278070327	1.69653887801836
H	-5.74683118969185	1.35899108744018	-1.80681732305931
H	-1.66230518941255	-4.29539786424334	0.23960839053290
H	-6.16595666262870	3.61223979733154	-1.14115775519850
H	0.48836918535793	-5.23293473331255	-0.07949414446898
H	-5.42492643198916	5.73802483824091	-0.06726206352932
H	2.92623069921954	-4.94949330073151	-0.54750517596939
H	-0.90080627927355	2.99330155034867	1.77451600544949
H	1.71499839733316	0.10863147818916	-2.61733700848017
H	-3.83484787675890	6.99848491096378	1.21433375427664
H	4.77736197239346	-3.61062912336016	-1.29434029864252
H	-6.49088930198323	-0.88662353601632	-1.23711718737957
H	-3.98401958652077	-4.31309842245535	-0.59943455024985
H	-2.63771651400922	-1.36986069600740	-3.16656183943311
H	-3.48222597932785	-0.68010903149351	1.71629668791875
H	-0.27596159030419	5.11991401779361	2.82994445395034
H	4.11549493763613	0.26545316803902	-3.10736807729741
H	-1.71501397145990	7.17028284452422	2.54150201787855
H	5.69954679754386	-1.54196558524251	-2.38021348455207
H	-7.38227497903482	-2.82862019758526	-2.52446413001766

H	-6.19100847149275	-4.71032665338403	0.49754780249388
H	-3.51294502715481	-3.33153771680558	-4.42699654783064
H	-5.70814821978216	-1.04293333806624	2.77051812393471
H	-5.88916325876979	-4.07893244215782	-4.11071128056089
H	-7.07753435117601	-3.06138711705534	2.17186648434082
C	-0.91381833493458	3.03756635945683	-2.59094223175669
C	0.90558379070590	4.03563773245109	-1.12294167551216
H	-1.79031593151312	2.39595184909052	-2.37708273288741
H	-1.28772323465034	4.08482818483137	-2.57331335285815
C	-0.35782526871955	2.68007197319735	-3.97097458253475
H	-0.00785116132173	1.62916862929059	-3.98620623906567
H	-1.15472067194451	2.78670077661000	-4.73504993499406
H	0.48603434838389	3.33237208054945	-4.26757738606583
C	2.05281240212209	4.33995303850689	-2.08141056684671
H	0.26960488631268	4.93350061029709	-0.95422493134326
H	1.31853878328249	3.81584629240895	-0.09659935927053
H	2.64333595579664	3.42374781640962	-2.28404053053500
H	1.68791337441272	4.74275457604718	-3.04653388523397
H	2.72903904893968	5.09413334773801	-1.63134457753841
Ni	2.09504659201130	1.94654081088032	0.48267489183530
H	-2.73661049382353	-3.74550031128437	3.10498213480557
H	-2.61702201412077	-2.27450073636121	4.14651753247019
C	-2.08002249238627	-3.17324843001298	3.79401237226705
H	-1.81938599271735	-3.81056524916525	4.66563407636398
N	-0.89221371317838	-2.71815232631443	3.11336572478766
O	-0.08785730626618	-3.72615841940395	2.58577018812881
H	1.00240587464402	0.70974850334574	2.95792810993654
H	1.50583790462058	-4.91381640585683	2.99510607163224
C	3.76501643798684	0.72125830503039	2.31062282367369
C	1.34269436481176	-0.00771356254040	2.18850698391171
C	2.54891714681635	0.34089624516197	1.43370879875029
C	0.98798663898178	-4.06093748129943	3.47414438240186
C	4.47932005584021	-0.52695592169630	2.80682874106260
C	-0.58387339486036	-1.39378701547724	2.86536654323269
C	0.64052550667988	-1.16415606270669	2.06119961822080
H	1.69888885888513	-3.21402547136503	3.58445017957833
H	2.81002462007364	-0.38988188026456	0.63981382777736
H	0.97950835386890	-1.95649271945368	1.38045299173028
O	-1.28996403399128	-0.47985560137420	3.32130087626619
H	0.61057840581089	-4.37135205925206	4.47250179461035
C	7.40692247302969	0.89173247494117	-1.59225511351634
H	8.13293902701629	0.68737901362319	-2.39443432778242
C	6.23300644099136	1.60949197440381	-1.86253062051139
C	7.65828343904824	0.45431813891343	-0.27981937719620
H	6.03361813114457	1.97776821672595	-2.88145085207792
H	8.58477024992176	-0.09469678250025	-0.04971932329188
C	5.31016861271527	1.86791009410694	-0.83601252306163
C	6.74488215071378	0.73235275280489	0.74664520894637
C	5.54644870830681	1.42834880414330	0.47872068643510
H	4.39609057977477	2.44566713413632	-1.03146671609254
H	6.97905745392250	0.41606805500371	1.77291263622409
C	4.57821072101711	1.83136399746280	1.59679678132721
O	3.57865965867845	2.71542249749179	1.14415218507491
H	5.20206110137586	2.35143997602378	2.36406246006668
H	3.37808853315616	1.20289751986516	3.23312401307262
F	4.87042282913785	-1.35333524262198	1.80487855973066
F	3.67433004160814	-1.26259235383453	3.62382645454365
F	5.58834693006530	-0.22166398403892	3.53730697364126

(S,S)-Q
Electronic energy: -4915.8913 Eh
Enthalpy: -4906.0190 Eh
Gibbs free energy: -4915.0473 Eh

P	0.52800022659778	0.85108860004753	-1.60250731919001
O	-0.59223181338159	1.13534233215043	-0.35311427706883
O	-0.48631532643622	-0.16767243629081	-2.50098683519963
N	0.57539339829656	2.17841975706901	-2.60792715717922

C	-2.66545719425032	0.10139082423166	-0.95187695499042
C	-1.93383844407770	-1.19603035442882	-0.94229134218049
C	-1.94577598287076	1.26282068354547	-0.58294738630064
C	-0.76049826167830	-1.27413037664903	-1.70252523289495
C	-2.55966909725565	2.53991117814341	-0.39430083227757
C	0.20970244650820	-2.30767416350044	-1.57792107607713
C	-4.05716332589572	0.21007151153143	-1.25337401095840
C	-2.29440397802920	-2.31139038413159	-0.12253101903515
C	-3.96417415828260	2.60933775991370	-0.69428653237276
C	-0.15278327429826	-3.39303660790324	-0.71505713788912
C	-4.66748369836720	1.45724240599854	-1.12053560764276
C	-1.40925148457531	-3.39025863692602	-0.06351135745497
C	-1.86704783295889	3.75284529519990	0.04987062015051
C	1.55531778520529	-2.26291728714439	-2.15876710637263
C	-2.60626691455718	4.99001530652851	0.09166146535818
C	2.46946255774903	-3.33419424789396	-1.83503041336084
C	-4.85221815727921	-0.95324996163610	-1.72017411299138
C	-3.53286109542313	-2.34989402693156	0.69548827060474
C	-4.65817117620329	3.86465856047068	-0.60550553637963
C	0.78933662136132	-4.44251230257217	-0.44692922205569
C	-4.00300002903841	5.01122939183487	-0.24304352221396
C	2.05151533780148	-4.40677356501161	-0.97621118094445
C	-0.49958474284238	3.80787413060797	0.44923435320498
C	2.02155769324273	-1.27384253565691	-3.09961240256981
C	-1.95611786495557	6.19649543597940	0.46865695170073
C	3.77909024165112	-3.33269426558139	-2.37264023810084
C	-4.34585460390490	-1.84654827714279	-2.69041748867669
C	-3.91648588416135	-1.26660888984871	1.51500453175293
C	-6.14894471782771	-1.17735644097397	-1.20774331453471
C	-4.33401889688893	-3.51294208389205	0.69327516290120
C	0.11489094569977	5.00337694739583	0.81448894445413
C	3.32720014359942	-1.31930477213506	-3.61732174115570
C	-0.61017113233553	6.21511622726515	0.81602787003579
C	4.21892274733238	-2.33261011235856	-3.23792121449965
C	-5.10702662110376	-2.94329097421416	-3.11730164311217
C	-5.07298248496452	-1.34388097243134	2.30222726601011
C	-6.90885333466055	-2.27414414094485	-1.63428875424996
C	-5.49352060686807	-3.58722625876461	1.47674985348496
C	-6.38848443488098	-3.16554733602926	-2.58649078373213
C	-5.87003496256338	-2.50035110609618	2.28264535061820
H	-5.73003583640973	1.56709364665467	-1.38428076831505
H	-1.64279775022967	-4.23436788828484	0.60123604009035
H	-5.73119872943121	3.88197606936379	-0.85074806418043
H	0.48253690616034	-5.25537701671311	0.22697681948978
H	-4.53772265926334	5.97249735468867	-0.19359702810397
H	2.77848143368100	-5.20143769631755	-0.74862080157274
H	0.08919508355419	2.88978770161708	0.47124608619800
H	1.30708922891457	-0.60017321193219	-3.58276764277699
H	-2.54902296502489	7.12457303595033	0.48123628211991
H	4.45555447185595	-4.15376728802036	-2.08863246820619
H	-3.35078299319842	-1.66924783278933	-3.12278970191466
H	-3.28132415367044	-0.37398703509476	1.57236727574467
H	-6.54478472133404	-0.50476360308678	-0.43243990457591
H	-4.05494275027265	-4.35637290616167	0.04394182133130
H	1.17733545104642	4.98865954338669	1.10274273985901
H	3.63266929971219	-0.55577183167329	-4.34853387804096
H	-0.11990720774331	7.15896154297171	1.09902997708856
H	5.24466185069600	-2.35140899211557	-3.63429934373960
H	-4.69717670940407	-3.62752279238914	-3.87615579570249
H	-5.34600571639539	-0.49471198417473	2.94748943587961
H	-7.90992670350940	-2.44253827142064	-1.20857542783413
H	-6.11253278104528	-4.49716761372887	1.45028426208371
H	-6.98210186431769	-4.03143782761782	-2.91780700651850
H	-6.78114770089294	-2.55656544521344	2.89815728403155
C	-0.55842764618264	2.58770642796387	-3.45784124071940
C	1.84963086848645	2.90027561188553	-2.72480333283947
H	-0.34567925863223	2.29106188583999	-4.51010769197133

H	-1.44098916810872	1.99612580933399	-3.14909882492286
C	-0.87654263544320	4.07877004213752	-3.39121947576288
H	-1.78792092900361	4.28492755343133	-3.98718776477676
H	-1.06573336432526	4.40495361805502	-2.35064338630601
H	-0.05989896810872	4.70086857625628	-3.80917105552424
C	2.50169501570668	2.77769512488974	-4.09886847225438
H	1.69057505215081	3.96733933824618	-2.45630839452525
H	2.52692584284501	2.48767678119452	-1.95192936808658
H	3.47509826918768	3.30793433160807	-4.10292774937725
H	2.68836034411639	1.71359072172403	-4.34908492053441
H	1.87708356735631	3.21841134024389	-4.90218941945898
Ni	2.28304879111608	-0.15798771830357	-1.00693984404707
H	-1.21639335411784	-3.83020456923229	3.64182572076899
H	-2.83219315254839	-3.01507659125147	3.52394750685758
C	-1.79843322409704	-2.93340037465242	3.92120551112108
H	-1.83904110381707	-2.86212315300678	5.02968663691023
N	-1.11774824470081	-1.80134709096426	3.34130479219902
O	-1.72395287479497	-0.56224250428357	3.54648118312132
H	2.33611126483560	-1.49255096302061	1.43907696360034
H	-1.38939212624836	-0.55246460837100	5.63230471372424
C	3.82225795063876	0.84837443459932	1.07367243595267
C	1.73362327531329	-0.57285401261910	1.38753968932578
C	2.33603241929069	0.61428181870375	0.77654240156694
C	-1.21999406063439	0.07205525368750	4.72829142379025
C	4.34049337203668	2.15833155904684	0.50720295173575
C	0.02313085818293	-1.89208332856844	2.55629155567253
C	0.49256971886393	-0.62830402886409	1.95703142374109
H	-0.13657170946902	0.30257150200736	4.63141142380058
H	1.73584859999011	1.53001946691961	0.93338121446641
H	-0.16239564808807	0.25197583025159	1.98464679541000
O	0.58857237576951	-2.98949175644215	2.40985038888594
H	-1.79247539947330	1.01462493278587	4.82372862143451
C	5.42442884012504	-3.40359079749085	3.52104174959686
H	5.62210760196357	-4.17276191102262	4.28360392224273
C	4.73188074513825	-3.73239493795491	2.34499588830271
C	5.86658613115917	-2.08352395320082	3.71790092899667
H	4.37966357371646	-4.76297737455805	2.18096075151636
H	6.41734042183259	-1.81734388920721	4.63378993968687
C	4.48003340268916	-2.74883907318451	1.37558457761991
C	5.60745872562712	-1.10161681313309	2.74973296971171
C	4.89830032602087	-1.42233095536532	1.57505795015681
H	3.93599896034322	-2.98363183035944	0.45068901687010
H	5.96528886681324	-0.07041894112284	2.90700687339528
C	4.62243496504398	-0.35760582971535	0.50913488759618
O	3.94152185075970	-0.84740661609604	-0.60657457540384
H	5.63437856974562	0.01035926981445	0.19597690389372
H	3.95821519098532	0.97109155387466	2.17290081681114
F	5.57052674200961	2.44544336982227	1.00582309577624
F	3.53187818262179	3.20939112762373	0.82209791046602
F	4.46705371710611	2.16040638352151	-0.84909350436263

(S,R)-Q
Electronic energy: -4915.8879 Eh
Enthalpy: -4906.0192 Eh
Gibbs free energy: -4915.0446 Eh

P	0.35690003692124	0.77657611141985	-1.62776003598621
O	-0.73314238242158	1.10339163471742	-0.35546494419508
O	-0.64612429403132	-0.31268955481053	-2.44227029717865
N	0.35259957613490	2.02969143130654	-2.73089142302040
C	-2.80308062289680	0.02389187136774	-0.87788051080265
C	-2.05812580721950	-1.26479635899372	-0.80647094200314
C	-2.09023747239189	1.20900913465138	-0.57410978307049
C	-0.89357790052052	-1.37379424103246	-1.57568730343364
C	-2.71663790393161	2.48593763025463	-0.43500189165863
C	0.09103046588163	-2.38637434943558	-1.40856938693759
C	-4.19918132852192	0.10330562638837	-1.16584539395531
C	-2.39415353987510	-2.33652132654101	0.07970155460313

C	-4.12522849775814	2.52701862063543	-0.72231153381732
C	-0.24525507007418	-3.42418553433773	-0.47925231965668
C	-4.82114906571671	1.34942454533547	-1.08513551679387
C	-1.49378348450249	-3.39950414716919	0.18713489919844
C	-2.03256281174407	3.72582773990217	-0.05486008818130
C	1.42839082212602	-2.35553079159294	-2.00855835491081
C	-2.78417323058016	4.95605798554350	-0.06408280828381
C	2.36703180037467	-3.38475462817921	-1.62286141139981
C	-4.98769118422472	-1.09014867271108	-1.56267267086700
C	-3.62483170020743	-2.34812619881239	0.91049120066602
C	-4.83059485569892	3.77883345046897	-0.68897240520403
C	0.71717525381185	-4.44018980301890	-0.15875587645903
C	-4.18376068580930	4.94779583769519	-0.38848336657643
C	1.97697764058178	-4.40783621683231	-0.69380313280138
C	-0.66248304529750	3.81322232459034	0.32776902106214
C	1.86177834635116	-1.42803577883246	-3.02438419128088
C	-2.14452121595988	6.18478329304724	0.25370275086941
C	3.66781593214774	-3.39878921537706	-2.18082089764070
C	-4.48491590137432	-2.02566247214667	-2.49403731679563
C	-4.02328631005451	-1.22722725484022	1.67069649712459
C	-6.27456429855061	-1.30196245738088	-1.02095020017160
C	-4.40568897122975	-3.52313935436041	0.97913043500046
C	-0.05792284714006	5.03041773200709	0.63425579916827
C	3.15774283738745	-1.49162060597795	-3.56370092797040
C	-0.79628402733622	6.23300290246071	0.58924804352297
C	4.07288188241924	-2.46127556716637	-3.12919012918184
C	-5.23969932757139	-3.15029771568870	-2.85485067359597
C	-5.17263534622870	-1.28136128514090	2.47024839997302
C	-7.02775425039135	-2.42690522199846	-1.38077776751323
C	-5.55840072409196	-3.57414841612285	1.77441830265536
C	-6.51091262613147	-3.35924106838649	-2.29493191516687
C	-5.94834751620839	-2.45109329288691	2.52217409931618
H	-5.88797379481720	1.43607516450666	-1.34024644640643
H	-1.70846718358376	-4.20779972469100	0.90125186839519
H	-5.90562470949175	3.77397987562371	-0.92575026861690
H	0.43025106700146	-5.21737640884108	0.56404705498231
H	-4.72721614052422	5.90539495077528	-0.38154201054437
H	2.72229540394534	-5.17071574277690	-0.42171428299537
H	-0.06572771445739	2.90194407179272	0.39249326863683
H	1.13033117541445	-0.79506903581382	-3.53629477990699
H	-2.74749939840013	7.10615607879874	0.23019933192338
H	4.36428168561982	-4.18439062902033	-1.84884453910931
H	-3.49806177487722	-1.86046777421571	-2.94944619577489
H	-3.40554914448905	-0.32100477371146	1.67109094829874
H	-6.66719132085089	-0.59545915268065	-0.27477090844340
H	-4.11698387744522	-4.39606966891989	0.37455276751185
H	1.00663616912306	5.03984817958005	0.91464963997489
H	3.43601847725955	-0.77747999970246	-4.35335702600443
H	-0.31431625725798	7.19350412227192	0.82687041656738
H	5.09200226820563	-2.49270592664223	-3.54155595128502
H	-4.83295264988232	-3.86743294518857	-3.58443861459319
H	-5.45806456542118	-0.40191526718377	3.06793314218572
H	-8.02065612881450	-2.58447954172964	-0.93236440291022
H	-6.16168136180019	-4.49452936638838	1.80261425842999
H	-7.09942423533992	-4.24684659463410	-2.57373374192802
H	-6.85389645339910	-2.48878519504206	3.14723622476042
C	-0.68502281950628	2.25178780033899	-3.75409618653525
C	1.52552829055836	2.92114796588445	-2.73039315890182
H	-0.20384233777535	2.16842845788698	-4.75476983822802
H	-1.40518683537879	1.41518971136383	-3.68970337382157
C	-1.42018257477097	3.58590045926284	-3.64141137680677
H	-2.18061965897320	3.65318544641688	-4.44537679580895
H	-1.93821628923173	3.68030262831621	-2.66857368121322
H	-0.74071922795249	4.45435907561139	-3.75684778598305
C	2.55255351234447	2.58617296333906	-3.80883957864116
H	1.16417885820452	3.96589649905951	-2.83249131270693
H	2.00070995843975	2.86959861410849	-1.73231437540533

H	3.39513085330277	3.30522247427675	-3.77184445420932
H	2.96427515592934	1.57015252866734	-3.64400091266192
H	2.11401747545771	2.62753414446742	-4.82655533040518
Ni	2.14649394463176	-0.15906672411685	-1.02120052145188
H	-1.11818920167151	-3.72160692084932	3.88118521088153
H	-2.76027247488608	-2.99889483952463	3.62264858545438
C	-1.75492598054068	-2.83762879804739	4.06683544062719
H	-1.86101538629500	-2.69390516157837	5.16347586922278
N	-1.09272977164737	-1.71397962715216	3.44995652610119
O	-1.74836844826500	-0.48873440385235	3.56586720275530
H	2.33750381554451	-1.41851640287766	1.45803957382314
H	-1.47430818707159	-0.34936899803158	5.65567602103386
C	3.74361128907233	0.97364848138791	0.93641164783373
C	1.71315341935496	-0.51013034871064	1.40157797167137
C	2.25175832589273	0.67669802744656	0.73483515762199
C	-1.30279849491280	0.22961224811452	4.72221804385310
C	4.17071631742638	2.24845215150819	0.21840029798330
C	0.05248466102427	-1.81385011583247	2.67141583864627
C	0.49099345887917	-0.57061729797649	2.01264337356068
H	-0.22642338767695	0.49663845423397	4.64121009820505
H	1.62103517433586	1.57174032665984	0.88332708259339
H	-0.17817517228234	0.29934724584609	2.01928730897524
O	0.64744516835329	-2.90200396682483	2.58302306840535
H	-1.91269289025401	1.15302401913742	4.74846846941336
C	8.76222611383449	0.17447362703715	-0.16382366086095
H	9.84439923715068	0.29934808998417	-0.32394644768460
C	7.89085568782488	0.07694023521350	-1.26060875329451
C	8.24519324409926	0.10301563704478	1.14171986259047
H	8.29016485396355	0.12452807432266	-2.28610567757467
H	8.92245915273336	0.17108826566765	2.00742216254539
C	6.51116869938570	-0.08209814320184	-1.05568134987469
C	6.86754310725151	-0.06310000756100	1.34361737867134
C	5.98755761897524	-0.14885111172136	0.24755123392636
H	5.81108463699419	-0.16294172110956	-1.89918370202931
H	6.46634683514578	-0.12961007831919	2.36846816819698
C	4.48851490336746	-0.30607146095484	0.46676755495393
O	3.84401728182817	-0.76742379267827	-0.67883806685600
H	4.38033714024517	-1.02833879500528	1.32100685691207
H	3.94258873737970	1.19542922278753	2.01086947884964
F	5.36694844858610	2.69862455945613	0.66091765095840
F	3.27366216323359	3.26352881935088	0.43898218512803
F	4.27021943267925	2.12249678074100	-1.13055661003634

(R,R)-K.1’
Electronic energy: -4915.8915 Eh
Enthalpy: -4906.0275 Eh
Gibbs free energy: -4915.0519 Eh

P	-0.07658018840688	1.68108031808093	-0.28865161072023
O	-1.34638995929850	0.76828232347433	0.39449678827888
O	0.25024981991043	0.68515736702808	-1.61463582073652
N	-0.70041514152134	2.95401493674235	-1.18098801268131
C	-2.12480420548756	-0.67907127541072	-1.30513520535901
C	-0.81936729725461	-1.39710447017345	-1.21615459154757
C	-2.38008363485488	0.41042381538341	-0.45169536221547
C	0.37306401206041	-0.68113986889460	-1.43782355872890
C	-3.60425477437681	1.14646957875333	-0.44064403291679
C	1.64610879075852	-1.29957801037939	-1.61936023031422
C	-3.12135991184069	-1.06733465585240	-2.25419079436388
C	-0.75700832728952	-2.81428622442788	-1.03929095997444
C	-4.58662753477587	0.72514162915736	-1.40235961220838
C	1.64790635416263	-2.73538785690020	-1.56765054893767
C	-4.32451724145568	-0.36146134317454	-2.26929130408074
C	0.47089585945536	-3.44448857398302	-1.23620176343612
C	-3.90299462661783	2.29416717016840	0.41959204156341
C	2.90919179204457	-0.60208939937103	-1.86428498059554
C	-5.13558954408553	3.01180668276380	0.20348909365161
C	4.07752825245438	-1.38540028651612	-2.17948693701734

C	-2.91312381224895	-2.15011314057188	-3.25008804749676
C	-1.94827663641405	-3.61921418082552	-0.67215469237836
C	-5.82362179995519	1.44397010638871	-1.53613669237049
C	2.85184951729244	-3.46769565469766	-1.84432665208723
C	-6.07887264218271	2.55558907963479	-0.77921648033042
C	4.01168358503685	-2.82011474836644	-2.17553771194526
C	-3.06130048778758	2.76274010566373	1.46820738744486
C	3.09147885534904	0.80725251665649	-1.77601822206402
C	-5.43323818579752	4.16622087287775	0.97611971484073
C	5.31829985475440	-0.74517629501022	-2.44340841453069
C	-3.92993573512382	-3.10333538144518	-3.47673813291784
C	-2.17333517722938	-4.87853958700592	-1.27125298318007
C	-1.72822766766328	-2.23248096072125	-4.01491422081265
C	-2.86941637698941	-3.16223774535918	0.29725645173925
C	-3.37670196468672	3.89826002757751	2.21223196782840
C	4.32934319071907	1.40921848114452	-1.99356409210834
C	-4.56289400029620	4.61943943836770	1.96091966396108
C	5.45394319454743	0.63571782106161	-2.35109886910427
C	-3.76355317051239	-4.11841761968703	-4.42840666266632
C	-3.28931834666943	-5.65099741415739	-0.92377079914613
C	-1.56147806201707	-3.24859914164603	-4.96570830215359
C	-3.98782709154345	-3.93319094619565	0.64237817101311
C	-2.57599398665887	-4.19846982632605	-5.17334659989921
C	-4.20541475527708	-5.17902384629929	0.03033461985471
H	-5.08803452680882	-0.62152743513879	-3.01765493318539
H	0.54095896532008	-4.53628044532055	-1.12019826348266
H	-6.55123580115662	1.08971463352962	-2.28233432285350
H	2.81065327748827	-4.56697517813749	-1.80318974438409
H	-7.01741694113686	3.11795184834764	-0.90319396142313
H	4.92490564031036	-3.38696502795107	-2.41507134926030
H	-2.14487999353200	2.21969533714931	1.71153951166023
H	2.25886051279617	1.46115859325324	-1.51713936405483
H	-6.37946716179064	4.69471805460503	0.78004372619204
H	6.18456278559426	-1.37612782301675	-2.69804148868550
H	-4.84643825693127	-3.06636253878900	-2.86854049220032
H	-1.48320822608071	-5.23644228958774	-2.04986809466128
H	-0.93519247179992	-1.48447889553235	-3.87153127168097
H	-2.69649948938723	-2.19848174963023	0.79719887934417
H	-2.68955132502921	4.22516874412084	3.00619022526022
H	4.41056239055661	2.50107444430102	-1.88239349563391
H	-4.80399837221281	5.51840645849700	2.54836844505173
H	6.42566664001600	1.11617679799844	-2.54188318026408
H	-4.56205515302279	-4.86115215563007	-4.57883707474096
H	-3.45351915294694	-6.62283389008487	-1.41421957286176
H	-0.63217426970765	-3.29500779324616	-5.55405544206834
H	-4.69195419027522	-3.56046156427511	1.40215717029903
H	-2.44152822250884	-4.99832710224811	-5.91775940707762
H	-5.08614967284834	-5.78219191258913	0.29950602725118
C	-1.28412398584759	2.82247126243615	-2.52965667144756
C	-0.82454880976055	4.25020865988511	-0.50208229452387
H	-1.75105772238525	1.82351088502583	-2.62764636605031
H	-2.11480370136839	3.55833318960369	-2.59272653184137
C	-0.28422064531584	3.02832223233532	-3.66868994858699
H	0.52566854789222	2.27575563279407	-3.61207280179475
H	-0.79682176814692	2.91103440713225	-4.64567747581432
H	0.17627667225501	4.03475797804565	-3.64186154513561
C	-0.02113074389334	5.38593915599542	-1.12963435884522
H	-1.90389335841332	4.51582941303050	-0.44817360980929
H	-0.50451401062522	4.10144556146459	0.55034685750146
H	1.05353511937834	5.12333798270165	-1.16528265518361
H	-0.37183627342856	5.62380211261443	-2.15353113794360
H	-0.13814617388070	6.30438527427399	-0.51848551811548
Ni	1.59118045545305	1.96550225327590	1.05907270389859
H	5.79697601653888	-0.96071398317648	0.24734190880544
H	4.58936738139187	-2.20793017531681	0.74279794087237
C	5.27146526303712	-1.42012118320925	1.10963855427350
H	6.01559967347150	-1.87110699714471	1.79950601184492

N	4.45716377287235	-0.43709301578497	1.78710925059030
O	5.12141913705682	0.73508953713937	2.15546404022077
H	0.45882813070989	-0.17408196537198	2.09239472907471
H	6.27853489288478	1.54615892552011	3.61414791214066
C	-0.96903976900179	1.28504283228061	3.91255874225414
C	0.98830015218054	0.70369021275469	2.49816740118435
C	0.29302515632201	1.52414820515012	3.48134288819505
C	5.74223920271143	0.59397300383009	3.43871075555136
C	-1.61816657317125	2.09391739041316	4.98511583769834
C	3.07494644613328	-0.43983739181079	1.76427803709549
C	2.40428371109927	0.72314851971620	2.41269878126659
H	4.98724691603558	0.43529116937200	4.23942433406318
H	0.84965368546396	2.37429079517948	3.91318820186430
H	2.98265785710347	1.31765315987543	3.13395649209433
O	2.45361860105570	-1.38999843529289	1.26842883510126
H	6.47541970163459	-0.24090808763939	3.44954143450755
C	1.99600554574363	5.24297912385607	4.89871127112772
H	1.68920475606566	5.70100988105626	5.85120883401641
C	1.24757619028072	5.47309030341492	3.73027814259999
C	3.14406357125017	4.43321197814566	4.84233833695421
H	0.35725154518360	6.11953677571533	3.76560445318703
H	3.74384854938183	4.26527947025104	5.75017033609799
C	1.62734937445691	4.87790683869078	2.52182206814696
C	3.52774227152855	3.83687165493976	3.63329575824595
C	2.76037510764893	4.03154966931198	2.46174190097886
H	1.05603232119729	5.05701429342196	1.60037252129318
H	4.42797563713146	3.20509294166804	3.59007495601939
C	3.08228701707059	3.29408850251756	1.21839015120642
O	2.37760174971290	3.47357384447514	0.13737658392267
H	4.07337126953715	2.78985321495336	1.15862748694808
H	-1.55930830818184	0.43387676299157	3.53882088405475
F	-1.13121122975428	3.36514111468058	5.05493263199847
F	-1.44067873090991	1.54595776429997	6.22104824888064
F	-2.96068024961884	2.18484574435967	4.80074645122436

(R,S)-K.1’
Electronic energy: -4915.8923 Eh
Enthalpy: -4906.0241 Eh
Gibbs free energy: -4915.0534 Eh

P	0.19524245757210	1.87975237304352	-0.56603356103837
O	-1.13254703292297	1.16309875456071	0.20958692653480
O	0.22633300867236	0.87125474640985	-1.92554459987892
N	-0.32041366382771	3.26451344570452	-1.35050874345211
C	-2.09310827474913	-0.42676706770691	-1.25098961278202
C	-0.78971609627365	-1.15046591648046	-1.20758619003866
C	-2.24211141936982	0.74371501212890	-0.48411014752830
C	0.37200128132064	-0.47824467477773	-1.63246662637103
C	-3.46990214955472	1.46130672833418	-0.36328391097300
C	1.64019052257866	-1.10786300848832	-1.80159479734446
C	-3.19846767273102	-0.90185243212352	-2.02171022335300
C	-0.69229419260462	-2.51550198453786	-0.79450646168553
C	-4.55327791151584	0.97756796657471	-1.17381022257510
C	1.70823541442111	-2.47769899406709	-1.37174366547917
C	-4.39158369068954	-0.17918598509081	-1.97325418369242
C	0.55822728792369	-3.13116988360377	-0.86904859712489
C	-3.68762408323109	2.62703371909592	0.49308968536875
C	2.83122895893065	-0.48336480092814	-2.37818935129147
C	-4.95082373268104	3.31624026098383	0.40600121330547
C	4.05068753682758	-1.24851173855297	-2.42340437713245
C	-3.12128848357545	-2.11947729754206	-2.86874317203992
C	-1.85398945199706	-3.28545245257122	-0.28033892408159
C	-5.80120506362878	1.68955586176405	-1.20486817140653
C	2.95467946976866	-3.18914769095206	-1.42921128250846
C	-5.98383625889780	2.82728671158740	-0.46484012529910
C	4.08350084778429	-2.59332703884154	-1.92223589602177
C	-2.74876130907188	3.11523967384912	1.44689030318690
C	2.86511634144845	0.82172036744111	-2.94750240048027

C	-5.19222011141031	4.46455558970809	1.20730120963779
C	5.22526312418394	-0.68471316314210	-2.98753088274832
C	-4.17294897264413	-3.06173479547057	-2.83405804086581
C	-2.03747470569273	-4.62835964410584	-0.67915209507697
C	-2.02533639690976	-2.36395868969804	-3.72619242025606
C	-2.78250544073681	-2.72264395411354	0.62464374273035
C	-3.01810255221452	4.23293784820723	2.23362045367539
C	4.02695923363403	1.34409875516375	-3.51415728385032
C	-4.24042086794853	4.92851924834472	2.10806538569166
C	5.22316105528174	0.59476350685219	-3.53117166516662
C	-4.12483722039132	-4.22159922422524	-3.61850849892489
C	-3.11728271452514	-5.38113616785694	-0.19897487610659
C	-1.97713439941980	-3.52502891529599	-4.50998991323752
C	-3.86063207831011	-3.47652876628001	1.10749982750020
C	-3.02323184666859	-4.46142366018626	-4.45542142418616
C	-4.03636955545087	-4.80768656361931	0.69472450286368
H	-5.23841231924494	-0.50040477585099	-2.59797569019992
H	0.66855447888493	-4.16619801273732	-0.51229024781333
H	-6.60414316556559	1.30162738748681	-1.85040941183744
H	2.97549625136178	-4.22840335243996	-1.06684130145412
H	-6.93688387439077	3.37741403154813	-0.50552905852940
H	5.03742546937816	-3.14184623038843	-1.96218345148669
H	-1.80279618074544	2.58781279501106	1.58568302505731
H	1.96084938689733	1.43471714933139	-2.95503557565149
H	-6.16386865871370	4.97375984863784	1.10888313162017
H	6.14277044828075	-1.29399989419345	-2.99690376752087
H	-5.01992188574337	-2.89695063003250	-2.15120249186382
H	-1.34286533028021	-5.07259805958186	-1.40765235212603
H	-1.20887826149799	-1.63027525754002	-3.78814279626213
H	-2.66043056586111	-1.68633236669177	0.96498492378246
H	-2.26665474716863	4.56594360844634	2.96592844336606
H	4.00338104863486	2.35588634233867	-3.94692256902077
H	-4.44351595882560	5.81592227709161	2.72667890096149
H	6.13901338405786	1.01604829000385	-3.97313882515150
H	-4.94728925021621	-4.95136551389900	-3.56437307751830
H	-3.24934887185456	-6.42069585675088	-0.53635100477737
H	-1.11587612922565	-3.69683134321918	-5.17385709618898
H	-4.56339524435828	-3.01664031509298	1.81924407324321
H	-2.98023107475582	-5.37553723914804	-5.06741969438285
H	-4.88674531429569	-5.39718872223533	1.07064845269178
C	-1.17800721147935	3.24584545990556	-2.55062060556381
C	-0.20011657144139	4.53184195861195	-0.61375960293512
H	-1.77679996885784	2.31378021899298	-2.56023701327691
H	-1.90800876968556	4.07680556219623	-2.44060576644047
C	-0.41363524529371	3.36886994218397	-3.86871470864042
H	0.26108424239816	2.50237817165676	-4.01164666038907
H	-1.12668834617072	3.38764466087983	-4.71843994455246
H	0.19444619396260	4.29339157318333	-3.90935563479215
C	0.53473411376565	5.62996699495968	-1.37523796809445
H	-1.21969497141717	4.87069710135206	-0.31848472763890
H	0.34610377098834	4.31005131280819	0.32610400809678
H	1.55218103309752	5.28993203853709	-1.64890574881930
H	-0.00660177291676	5.93160126340109	-2.29449985156540
H	0.62743334975839	6.53082852091086	-0.73493667813349
Ni	1.92336417447009	1.94731813365082	0.74866757176965
H	7.05579529097160	0.16304293618727	2.18448257098128
H	6.08983571719347	-0.98138049250021	1.16965721340005
C	6.22800016374268	-0.57512327084010	2.18775376062304
H	6.47445382254381	-1.40622869771771	2.88231565592728
N	4.98697206946674	0.06508913052168	2.55620221876752
O	4.99498619240406	0.73552055951544	3.77928823005039
H	1.39928145222628	-0.51435860370999	1.28827627048028
H	4.68985723753219	0.47094970055782	5.76923424835324
C	-0.78525009824296	-0.19276942439173	2.83368662410988
C	1.43054786656873	0.40016150030914	1.90873337876480
C	0.27489482387958	0.64253264175131	2.76756515459844
C	4.58900758907678	-0.13164803783544	4.84634632708239

C	-2.01560205073332	0.08510883080423	3.62087083900573
C	3.89132259064642	0.16040871196771	1.72921752393554
C	2.71788616672034	0.91356458934660	2.25359023743002
H	3.53181466742572	-0.45511569479581	4.72691420959992
H	0.27934687842618	1.55651311391400	3.38639259053331
H	2.84559955734559	1.50417058634629	3.17387298809397
O	3.88346331318464	-0.40134013478922	0.62214879437247
H	5.24553477047761	-1.02541083212074	4.92044581424747
C	7.56979578086211	2.42154116992542	0.66574273780948
H	8.64630503370476	2.20080042007411	0.59727062554499
C	6.72393723882193	2.15649832123725	-0.42732484527540
C	7.03640476393084	2.96624684305468	1.84654784808191
H	7.13372127141786	1.72073253951905	-1.35160271699817
H	7.69225032541570	3.17080064208364	2.70667707919276
C	5.35779695116075	2.44589850499451	-0.34822733953308
C	5.66651028497395	3.24924502827765	1.93013046434898
C	4.81699411742903	2.99989729085036	0.83221750500207
H	4.69116153211405	2.24934758828896	-1.19926544514735
H	5.24427160761183	3.67413795810937	2.85443933713407
C	3.38887365024800	3.37809670275893	0.91284419671039
O	2.64730141418081	3.48116882431218	-0.14818627649231
H	3.11099174780113	3.96824051805541	1.82399038641152
H	-0.82476490943333	-1.11637885414891	2.23490891618220
F	-1.98770642748154	1.27968196242899	4.26083172032700
F	-2.25647996133203	-0.86864289395037	4.56158688093783
F	-3.11937511341672	0.09500278848716	2.80690227224790

(S,S)-K.1’
Electronic energy: -4915.8957 Eh
Enthalpy: -4906.0323 Eh
Gibbs free energy: -4915.0569 Eh

P	0.94035914215296	0.51750691935629	-0.94735515708588
O	-0.36771036426602	0.91103670026743	0.07313383072981
O	0.12007590223565	-0.56751480803781	-1.95955179676937
N	1.16534281611901	1.73388471283426	-2.06905577040249
C	-2.34582537225081	0.08373129180085	-0.92020188507123
C	-1.78853406316770	-1.28787945119798	-0.74296667211634
C	-1.60548508556970	1.19257009913735	-0.46981375754206
C	-0.56826055152666	-1.61221750295433	-1.36495458807102
C	-2.08961213514495	2.53709876600373	-0.50560947391707
C	-0.06750992386236	-2.94509527395866	-1.46610455101547
C	-3.63020619622793	0.28956875381045	-1.51192245247250
C	-2.50410706971186	-2.30167622205139	-0.03531520494158
C	-3.39415625657164	2.70686811855713	-1.08881414180614
C	-0.80722660007013	-3.94085175043893	-0.73871959039995
C	-4.11810575272911	1.59399739376684	-1.57708224682439
C	-1.98479564624402	-3.59506913253639	-0.03911638922193
C	-1.37483705671267	3.72062480679364	-0.01769038881233
C	1.09838792537802	-3.36117793470014	-2.24751848071330
C	-1.99467974550515	5.01377345457081	-0.18133522863402
C	1.52521274956631	-4.73704871493033	-2.16581461087737
C	-4.44663708041305	-0.81821115822225	-2.07096459675941
C	-3.74277528398830	-2.01750354070232	0.73041388731793
C	-3.96819446668603	4.01789384206998	-1.21443733895337
C	-0.33532667469529	-5.29665520246199	-0.68837588177580
C	-3.29304245988210	5.12782825667326	-0.78417558344033
C	0.79569805189980	-5.67618374264289	-1.35970546303824
C	-0.09472839185601	3.69947523125857	0.60592774987452
C	1.83787463508380	-2.50370016969167	-3.11106785940573
C	-1.32366970511716	6.18884480013760	0.25211360121763
C	2.66969636043036	-5.17081639698503	-2.88528855662723
C	-3.88063796133493	-1.81084531853571	-2.90209753222416
C	-3.85693168510223	-0.87254888827807	1.55017574532942
C	-5.83084147281856	-0.87882467799541	-1.79696887889350
C	-4.82868581511564	-2.91838841305626	0.67898331752921
C	0.54400134737088	4.86653624364444	1.02077927460364
C	2.95052703911902	-2.95906430222018	-3.81492238306862

C	-0.06706984147837	6.12612643009114	0.84258322811115
C	3.38449376750858	-4.29628084899787	-3.69608889960583
C	-4.67375286838821	-2.83985236896955	-3.42788200536168
C	-5.02732941574367	-0.62936438289074	2.28115069700863
C	-6.62398935538113	-1.90671098391288	-2.32410487597718
C	-5.99891692811955	-2.67664811850959	1.41096009348026
C	-6.04743191647901	-2.89540789371544	-3.13757101136051
C	-6.10552369457279	-1.52776116954710	2.21133392830025
H	-5.09197567731462	1.77951868433583	-2.05436090750103
H	-2.48550940572387	-4.37690172174986	0.54974090766746
H	-4.96506995463388	4.10497993439103	-1.67308471959537
H	-0.90758454164040	-6.02177796095152	-0.09006262403513
H	-3.73432489529238	6.13119391613793	-0.88894967824138
H	1.15738509385680	-6.71513331889366	-1.31179874932657
H	0.42396384231394	2.75488336284338	0.77442456978535
H	1.53326132352912	-1.46086571287544	-3.22469188877490
H	-1.82732765803814	7.15779468367567	0.10822894582081
H	2.97792770793505	-6.22370005447823	-2.78928898339083
H	-2.81018521334241	-1.76808213682533	-3.14900735371780
H	-3.01303938656918	-0.17237796944872	1.62449755355748
H	-6.28006519999168	-0.12828399567589	-1.12918969654904
H	-4.76402544824401	-3.80116807829807	0.02539234127833
H	1.53667900222385	4.77402873440490	1.48410531079628
H	3.49834259461097	-2.25755616913981	-4.46215944966372
H	0.44223321492334	7.04559099142458	1.16960062532900
H	4.27249602053574	-4.64494058935755	-4.24495017512720
H	-4.21480709666282	-3.60240585213246	-4.07583179483641
H	-5.09505524883516	0.26646020121807	2.91741107135682
H	-7.69795725015717	-1.94395856642124	-2.08515664785506
H	-6.83947138787336	-3.38449197938250	1.34485201742509
H	-6.66749120581686	-3.70731260953126	-3.54780634892463
H	-7.02589885034363	-1.33382384334918	2.78341481557208
C	0.31473811316225	1.95943676262457	-3.25170725830204
C	2.35867006691032	2.57573351267391	-1.88859038682150
H	0.85010396530955	1.59643778381021	-4.15893018160067
H	-0.57950121160065	1.31788079865046	-3.14695730939726
C	-0.11084482341992	3.41246865510037	-3.44791372776525
H	-0.81375070742675	3.47290084797890	-4.30277316957564
H	-0.62623664839677	3.80684374439187	-2.55094532755123
H	0.74554417772780	4.07760288311118	-3.67722096037930
C	3.45203575254075	2.32441675903938	-2.92249520982256
H	2.04353757332621	3.64109808786952	-1.88194434500497
H	2.74411234998297	2.38751740757267	-0.86378658649287
H	4.31001207371622	3.00311186320934	-2.73901622281572
H	3.81071694680800	1.27820837989032	-2.84771070596409
H	3.09845226435412	2.50897932395187	-3.95751608045019
Ni	2.70930284342530	-0.03060077294264	0.18728825228972
H	4.97438783047918	4.16314738489968	1.48574227761082
H	6.34587965559357	3.26090595131430	0.72420567333297
C	5.76277851488434	3.40930879705007	1.65905408137125
H	6.44324777974725	3.76871668403363	2.45766393417271
N	5.10772457418983	2.18259934968247	2.05610360652513
O	5.97019938905543	1.10096030229992	2.25117996097760
H	6.85167388226979	1.89764417696772	3.99701864489169
C	0.15966279846443	-1.39915857053687	2.64356399114255
C	1.92602593732239	0.24045398493591	2.00912957135561
C	1.45302418040466	-1.08499049635609	2.38659890184579
C	6.33697592364558	0.98288089344402	3.63161604382867
C	-0.24372012999989	-2.77242045133473	3.07355733036674
C	3.76425523037654	1.93431702999969	1.84221581278275
C	3.31478570199409	0.53264530916188	2.02001834712815
H	5.44997227395618	0.77426522499300	4.26796343454525
O	3.00318224684514	2.86380499679347	1.51635802277963
H	7.03705468780564	0.12695653078257	3.67900527556895
C	4.42502369583208	-4.47894908014402	2.06785893401914
H	4.38395537444762	-5.39056809272221	2.68349059021963
C	5.37907821411698	-3.48359435865724	2.34619396391007

C	3.52791148719617	-4.30810055485395	0.99762554791494
H	6.08989853657637	-3.62043967123356	3.17594600932204
H	2.77817524897545	-5.08107400616680	0.76974586931705
C	5.42610685137382	-2.31596957619836	1.57178974594021
C	3.57585832509828	-3.14608886330062	0.21974765107555
C	4.51554706213749	-2.13017889304585	0.50723657049801
H	6.16574070141127	-1.52947432538899	1.78886766163274
H	2.88116574804339	-2.99860368805341	-0.61673547767171
C	4.49225535722803	-0.85645082322010	-0.24536801960446
F	-1.57983697571326	-2.96625663866670	2.94230628001694
F	0.05055965540032	-3.00925776498844	4.38593494437352
F	0.38490726061322	-3.75206833158352	2.36589898289174
O	3.74028186944417	-0.69819222939949	-1.29632807099794
H	-0.64405353246290	-0.64855164830378	2.60300713299862
H	2.21576569119740	-1.88055018229100	2.46241959766580
H	4.02359812656118	-0.20149790651609	2.42662181055700
H	1.20363219406163	1.07171138677103	2.01967703267012
H	5.33325104452641	-0.14806317285268	-0.05836075358739

(S,R)-K.1’
Electronic energy: -4915.8863 Eh
Enthalpy: -4906.0217 Eh
Gibbs free energy: -4915.0476 Eh

P	0.73236224413803	0.42297252098539	-0.87022691100944
O	-0.64694376022962	0.99968522309883	-0.08587153524623
O	-0.05294886498523	-0.42139878818060	-2.12306371229358
N	1.34057451531209	1.69265697111797	-1.77278113391114
C	-2.57233888104950	-0.05833098076669	-1.00492897750989
C	-1.88894058679134	-1.38374265269085	-0.96581247977435
C	-1.92479647507668	1.11642521798390	-0.57529965912407
C	-0.70505414026930	-1.56730501584965	-1.70053836306149
C	-2.57816870156336	2.38834797473568	-0.48232823293942
C	-0.16803939868391	-2.84506790128815	-2.02634790154187
C	-3.92940364812720	0.01988120310739	-1.45828932262753
C	-2.47232084281117	-2.51432437502608	-0.30766188990182
C	-3.96718006640885	2.40497950254376	-0.85028245396310
C	-0.78896700763228	-3.96357483710635	-1.37675754854981
C	-4.59295831118876	1.23961835877809	-1.35092008653520
C	-1.89877514012273	-3.76774748158942	-0.51931616257277
C	-1.96224832512724	3.63437394120864	-0.01693301203393
C	0.96408726368621	-3.07388183108796	-2.91953037204269
C	-2.79391016548099	4.80426022096651	0.12913583283350
C	1.50400453993341	-4.40477852832009	-3.01520249826121
C	-4.65136997245960	-1.14083256619829	-2.04241819489400
C	-3.60345707145909	-2.37053838610975	0.64114371160570
C	-4.74117448381415	3.60773372974246	-0.71876248180337
C	-0.23081349824010	-5.27852031180838	-1.53275597518054
C	-4.18393902970514	4.75685165746298	-0.22673901346967
C	0.88867698502808	-5.48556004974148	-2.29512097402191
C	-0.58015013112666	3.79396902112254	0.28968877389235
C	1.55463601797848	-2.07104548079254	-3.74071670482189
C	-2.23869068512793	6.02309498359836	0.60509687637224
C	2.62821734481245	-4.65654344367853	-3.84696261497349
C	-4.05448447441328	-1.96755099514697	-3.02067342019806
C	-3.64029875440911	-1.29273409916396	1.55313867252377
C	-5.97954578444241	-1.41690254971864	-1.64919186048578
C	-4.64288556842368	-3.32546422128645	0.68518875485746
C	-0.05756278383243	5.00322456688973	0.74529414365603
C	2.64264087721598	-2.34986408670509	-4.56625847847536
C	-0.88958432471407	6.13007156731083	0.91995293982225
C	3.20200779615013	-3.64594476353757	-4.61009105461364
C	-4.76038296019237	-3.04305408195378	-3.57634671273783
C	-4.68881923896243	-1.16431291619779	2.47270199596675
C	-6.68680321230145	-2.49062348081452	-2.20699677204292
C	-5.69286205496282	-3.19823919925775	1.60503490755334
C	-6.07821506611261	-3.31214308858794	-3.16947685997453
C	-5.72339318185378	-2.11466994263715	2.49914219434196

H	-5.63729154141397	1.31646348666341	-1.68833793508307
H	-2.29725348453595	-4.63950810885567	0.02093888349314
H	-5.80205830972228	3.57403211637831	-1.01055461413195
H	-0.71489322913575	-6.11421189950900	-1.00431590721236
H	-4.78641802504247	5.67079871982999	-0.10855211236685
H	1.32504369789064	-6.49211950432013	-2.39001216765163
H	0.10095109752583	2.95430565676694	0.14845387132407
H	1.14335767718951	-1.05750227565503	-3.72838205438235
H	-2.90792490397226	6.89126649719203	0.71197758779993
H	3.02939905485440	-5.68155769456532	-3.88542002739032
H	-3.02895963063524	-1.75809879870438	-3.35703865055599
H	-2.82728261414662	-0.55636309041563	1.55344261661434
H	-6.44615180220143	-0.80095037950211	-0.86575644806004
H	-4.64345684487926	-4.15654986356229	-0.03614258265384
H	1.01946518928389	5.07198779099567	0.96367855523236
H	3.06758808045218	-1.54542304165327	-5.18645596570338
H	-0.47427832766113	7.08093369070551	1.28682882219745
H	4.07138258308079	-3.85677062776511	-5.25125542234012
H	-4.27730985363411	-3.67402316267894	-4.33822060931715
H	-4.68532936492433	-0.31854661496741	3.17663667207957
H	-7.71601863192880	-2.69655854867970	-1.87490039290156
H	-6.50172335385129	-3.94510316806938	1.61474326060757
H	-6.62977744254962	-4.16020691929615	-3.60370315658125
H	-6.55048440988870	-2.01412961542457	3.21884768577426
C	0.57362178447393	2.28485378708295	-2.88335413043174
C	2.77921286706925	1.97740076537657	-1.67182686176339
H	0.84167070461933	1.77511007621934	-3.83720470805764
H	-0.49825180490359	2.06219668214967	-2.71265959512049
C	0.76901526711951	3.79181025391665	-3.02739719928562
H	0.10100003425329	4.17125203633703	-3.82614958485184
H	0.51810009767878	4.32403714025415	-2.08916111270731
H	1.80733240978080	4.05452769273033	-3.31231243393783
C	3.59403820801680	1.50837385923087	-2.87487754612470
H	2.91705552553448	3.06849337382074	-1.50973178561309
H	3.15637915581045	1.49273671489110	-0.74603329741548
H	4.67308545889488	1.67751378601043	-2.68528934288274
H	3.44559177650302	0.42426102969593	-3.05298222518521
H	3.32570889248719	2.04924677627012	-3.80566851652105
Ni	2.04031811730974	-0.53656659087415	0.55207711015575
H	6.87303666532514	1.17047892435853	1.45359193431447
H	7.44585811105445	-0.46925881935035	1.95562780271204
C	6.87800717974852	0.42954863583010	2.27296828073641
H	7.36394815854255	0.87580872226797	3.16597376887076
N	5.49929680800078	0.09184587489305	2.54084030745049
O	5.29669619608751	-0.90088609901948	3.49856864849460
H	6.09788197878490	0.23152839143254	5.09335688780556
C	-0.23392121968969	1.56785872747977	2.92450215761164
C	1.95394438415982	0.88615705925899	1.95070858952775
C	0.72228863480793	0.63869454730571	2.69849422017449
C	5.18736360727566	-0.34138178564734	4.81482737987760
C	-1.50607002057602	1.28641489687019	3.64306867027680
C	4.43290067067426	0.56684972006333	1.80583992788285
C	3.08831396398025	0.04971162440264	2.17243753954082
H	4.29283101168189	0.31284751633342	4.90200399006932
O	4.62053247988303	1.41108171773510	0.91323967856123
H	5.08099184489030	-1.20868344513913	5.49377136637749
C	6.89366869739452	-2.09944512982029	-1.26743199820181
H	7.88429754755984	-2.04570273032966	-1.74468645514965
C	6.78468646153249	-2.44129196906367	0.09191006364439
C	5.73297057411403	-1.83582454465921	-2.01779259181273
H	7.69065807828886	-2.66244710070742	0.67752882144431
H	5.81129256449930	-1.57914559386290	-3.08572999656380
C	5.52464501823459	-2.50976289777340	0.70262470555509
C	4.47370783089861	-1.90983989193592	-1.41346238721870
C	4.35844067151706	-2.24282470984657	-0.04535551391099
H	5.43775128316969	-2.76499006072899	1.77013843456338
H	3.55596734525967	-1.72974352772654	-1.99114468178840

C	3.02290645235889	-2.34321658294161	0.58218133666280
F	-2.58909495304201	1.56541697321130	2.85028967380007
F	-1.64728489200047	2.06243847555463	4.75179908155538
F	-1.63222503225519	-0.00405911662580	4.03810009847850
O	1.94383218462605	-2.31869254040474	-0.13356907178006
H	-0.14840494443425	2.59790720652582	2.54614624231121
H	0.57958811818552	-0.37804810964567	3.10443682794347
H	3.03772586035469	-0.72059301497009	2.95513047672846
H	2.12110137881363	1.90844319737411	1.56767523202673
H	2.98952287803719	-2.77486617322822	1.61143039864748

TS (R,R)-17a'
Electronic energy: -4915.8418 Eh
Enthalpy: -4905.9772 Eh
Gibbs free energy: -4915.0020 Eh

P	-0.56866969613043	-0.39650035575844	-1.47374463311754
O	0.54529150246354	-1.17293041868681	-0.44946275217182
O	0.47962592583537	0.89025192019634	-1.91693664125158
N	-0.57901136831277	-1.19446002521682	-2.94476115021413
C	2.58303686257551	0.06425680100569	-0.43867058489429
C	1.81173427794848	1.26262215123823	-0.00533756204755
C	1.90996748727277	-1.16174572160810	-0.62256663381637
C	0.76001335665330	1.69611180313061	-0.82996266605492
C	2.58663395673187	-2.38831637359553	-0.91539934819523
C	-0.00354431072176	2.87860515608343	-0.59634754399181
C	3.99404782079890	0.13031267555251	-0.66222676019398
C	2.12940793313708	2.00855572914572	1.17334436767235
C	4.00074575602180	-2.28377249672921	-1.14826279878309
C	0.32610723191687	3.59867548863179	0.60245764576734
C	4.66102916262517	-1.03863967933498	-1.02427344516836
C	1.37005675766835	3.14808065717849	1.44599530683087
C	1.95652402114323	-3.70803239182704	-0.98617223305352
C	-1.08205029659072	3.36894414029056	-1.45193232769649
C	2.76043840442198	-4.83423062486999	-1.39399794006279
C	-1.81473425983574	4.53401621022521	-1.02857057499521
C	4.76571291628943	1.39498525227760	-0.55034974728309
C	3.21211547577422	1.61819081598789	2.11205220850204
C	4.75649993144467	-3.43996955102694	-1.54391468400962
C	-0.42678096735379	4.76654829995225	0.96811238217032
C	4.15603531583765	-4.66205446852261	-1.68546241971594
C	-1.46064208225030	5.21104441761625	0.18711657296034
C	0.60528714034165	-3.97805971497521	-0.62551291939439
C	-1.47862139658599	2.76644072719701	-2.68156517455716
C	2.18555835646161	-6.13130445321983	-1.47403743202454
C	-2.89955182594677	5.01706635371956	-1.80764961811352
C	4.30733478922698	2.59969658997947	-1.12884025414459
C	4.05953204193842	2.61029890049370	2.65464816058082
C	6.00502472268193	1.40208968638948	0.12702850674852
C	3.41443333160620	0.27589386091512	2.50408754167175
C	0.07562171568311	-5.26486722332602	-0.68603439180944
C	-2.55487517282701	3.25398110562893	-3.42064876812905
C	0.85829070047477	-6.35312600887192	-1.12796050621711
C	-3.28222408215712	4.38132339868225	-2.98327692083355
C	5.05833403188666	3.77773778527707	-1.01558700056402
C	5.08260439723255	2.27255263800458	3.55022998837195
C	6.75588044678283	2.57964319455280	0.23944021504662
C	4.43601195171377	-0.06121893599799	3.40243594422430
C	6.28244464600759	3.77458214495117	-0.32590831204977
C	5.27814428638038	0.93362200000696	3.92614249637110
H	5.73728240461922	-0.99630476732188	-1.24895670404525
H	1.56710147649241	3.71340933968104	2.36926479912692
H	5.83302195928076	-3.31387767987125	-1.73656279566019
H	-0.15365962863807	5.29205977012958	1.89625520204404
H	4.73801026638839	-5.54269533089812	-1.99892953475600
H	-2.03963021123056	6.10204431590374	0.47677726383543
H	-0.02951878381868	-3.16276481761273	-0.27320413666825
H	-0.91785617947129	1.91504870753928	-3.07448496802084

H	2.82618856298999	-6.96577469834012	-1.80019544542924
H	-3.43608813944347	5.91269138903162	-1.45596041385267
H	3.35923310739374	2.60815686452798	-1.68518281349428
H	3.93663807003609	3.65608545082722	2.33550516848876
H	6.36173050722641	0.47686089622860	0.60383101661765
H	2.76414272975848	-0.51554939657197	2.11075267737474
H	-0.96580383939009	-5.42631533932380	-0.37232470963616
H	-2.83067649133671	2.75197547408865	-4.36065330884666
H	0.42777859465957	-7.36459101895011	-1.18251822919895
H	-4.13147433362345	4.75972706436319	-3.57235173443206
H	4.68606212852245	4.70584288532316	-1.47616497240505
H	5.74190886563932	3.06021461003169	3.94627729497561
H	7.71137100248830	2.56719186320135	0.78605978890182
H	4.56799672041298	-1.11331264543957	3.69851139106834
H	6.86801861015278	4.70223744651886	-0.23321538443342
H	6.08408707523875	0.66589392563431	4.62668618182977
C	-1.85872928549236	-1.73636949817484	-3.42882035298923
C	0.60694326007716	-1.31930124112449	-3.80435078371242
H	-2.64642462303458	-1.39576692435654	-2.72146849094524
H	-2.10099371143686	-1.26997192833037	-4.40946244189127
C	-1.88142101098043	-3.25935365565913	-3.54521958568102
H	-1.15498543572004	-3.63718501584276	-4.29323879946488
H	-1.64043415535727	-3.73427220390850	-2.57334334949839
H	-2.88923112921788	-3.59797441908066	-3.85919124710769
C	0.56997493040544	-0.40620875415259	-5.02997120876026
H	1.50365896389703	-1.09250101750581	-3.19707691749430
H	0.70747242502912	-2.38271505951986	-4.11270982987476
H	-0.30926542517988	-0.61278991848941	-5.67310147566631
H	0.53667799956832	0.65835829348828	-4.72361987006060
H	1.47828182502612	-0.56075494116829	-5.64676850522511
Ni	-2.34139850789433	0.26880703704755	-0.68102374978737
H	-6.51888424458884	3.48204252625458	0.54314470968858
H	-4.84562880403000	3.85234748305590	-0.04509054434515
C	-5.45851896647744	3.60961499815952	0.84291633046540
H	-5.38037498776189	4.43862163771235	1.57598294013376
N	-4.95036636755226	2.37464064311304	1.40186050308585
O	-5.67392082851325	1.83114135325495	2.45873205912865
H	-1.57732559744669	1.07181036160824	1.49536852009631
H	-5.22110986117290	3.39866308243353	3.80351820791576
C	-0.30490225168066	-1.10375989834748	2.38758652445048
C	-2.12575923952683	0.14022570468018	1.25168926370992
C	-1.52102909959758	-1.06857322086567	1.79560351497672
C	-5.16984843070949	2.29177921128147	3.72225554328584
C	0.32657704537575	-2.36915846690227	2.85913050169818
C	-3.97207793141550	1.62240957249256	0.85062112353850
C	-3.64072806732775	0.25257860040944	1.30957628036537
H	-5.83405834633008	1.83791433189056	4.48147445723200
H	-2.08457919667707	-2.00916172875178	1.69120049082083
H	-4.08320652648186	-0.08955498286873	2.26303845011947
O	-3.32773512483230	2.00716358056957	-0.16528897311171
H	-4.12516080243423	1.95022298059617	3.88727616877232
C	-4.18865864391435	-4.83705774138607	1.49444976953558
H	-4.10561153571107	-5.87958126154999	1.83802058152350
C	-3.61265613858160	-4.44503497770579	0.27469088207271
C	-4.88624986394533	-3.89139740818941	2.26626162904274
H	-3.09077064330143	-5.18623390677675	-0.35030605512431
H	-5.35405016937385	-4.19259055326344	3.21658364738643
C	-3.71415577118478	-3.11515926054024	-0.16129997365521
C	-4.99638011464318	-2.56549902090090	1.82148325971557
C	-4.39918454381082	-2.16181219276779	0.61107339278810
H	-3.27046194771665	-2.78498674893829	-1.10983541484077
H	-5.56114178861865	-1.83188333173550	2.42153623355896
C	-4.51789729425534	-0.72477525998063	0.10990732472534
O	-4.14923315032564	-0.50754853961736	-1.15577576935621
H	-5.55341908205940	-0.35915298730141	0.35810913734653
H	0.30847883429635	-0.19737216655247	2.51814958139845
F	1.54385230917254	-2.56765512894484	2.26197202589604

F	-0.41978070514015	-3.46837416375440	2.60106098019018
F	0.56731308449126	-2.34781723103736	4.19800205988039

TS (R,S)-17a'

Electronic energy: -4915.8344 Eh

Enthalpy: -4905.9798 Eh

Gibbs free energy: -4914.9962 Eh

P	-0.23563168731462	1.21780176483439	-0.51884739465496
O	-1.56668344723039	0.62450586809132	0.37245127712971
O	-0.34759378380011	0.02605820822088	-1.75494462777027
N	-0.73335597701080	2.52156086215437	-1.45163126257482
C	-2.72409529405554	-0.99139783308930	-0.92542761363606
C	-1.49509892734016	-1.82741065262952	-0.82715034193797
C	-2.72631426229548	0.25318708604082	-0.26427107976160
C	-0.28420270720367	-1.26823922624140	-1.27766783518681
C	-3.85925092114889	1.12117192175836	-0.22360666894762
C	0.96897918759835	-1.94952104726003	-1.22311181160746
C	-3.88913085471577	-1.40850750851565	-1.63858265954712
C	-1.49353199384788	-3.14363294520394	-0.26861770016777
C	-5.00848203160550	0.68410688452558	-0.96725005563874
C	0.95503231214994	-3.23144968043178	-0.57411389913949
C	-4.99756374187585	-0.56069434986314	-1.64185816742369
C	-0.26716799081367	-3.80082698630076	-0.14290143197948
C	-3.90662400505051	2.40754858196733	0.47264986945495
C	2.23557360735861	-1.41862087592800	-1.72635936834667
C	-5.06748776563749	3.24256259724737	0.28932747659857
C	3.44988238222439	-2.11732006172768	-1.39391423141271
C	-3.94475015006231	-2.68806103316273	-2.39009005926387
C	-2.72323880424026	-3.81488554437889	0.22610245948517
C	-6.16009495502570	1.53698449860503	-1.07662354301700
C	2.19360320849825	-3.90894677636276	-0.30368220415548
C	-6.17769850394921	2.77699305968131	-0.49535620903182
C	3.39421381903503	-3.35286698864809	-0.66114741868799
C	-2.89004994121873	2.89390407902593	1.34619297140663
C	2.35017978277360	-0.26713024381548	-2.55567163091729
C	-5.13049766365130	4.52058094871330	0.90768658982298
C	4.70266975560297	-1.61264722477979	-1.83516584379932
C	-5.08485431178892	-3.51542362536033	-2.28953928265793
C	-2.96090944273800	-5.16472745227495	-0.11488372801502
C	-2.88489268221412	-3.09956629141301	-3.22867489216485
C	-3.65652148183272	-3.15235546778149	1.05385439942508
C	-2.98211717350467	4.14750620486123	1.94747413800807
C	3.58866593899289	0.19088635416363	-2.99560414048551
C	-4.09891519430089	4.98095759033509	1.71751728322101
C	4.77856076221547	-0.47146803476038	-2.62402791037366
C	-5.15642201139201	-4.72751292595197	-2.98856311333677
C	-4.10700446750395	-5.82953008896530	0.34072759666914
C	-2.95683242399133	-4.31232726881318	-3.92761778880921
C	-4.80152303652275	-3.81955122964864	1.51022048402882
C	-4.08964856242135	-5.13446128567164	-3.80597862915598
C	-5.03545359189520	-5.15745810028341	1.15175176881697
H	-5.88741088558335	-0.84265753962690	-2.22454578907686
H	-0.23565814478811	-4.78105389229424	0.35604598928781
H	-7.02052270262763	1.17514696517720	-1.66033519118281
H	2.15314191885966	-4.87660382545381	0.21965782627690
H	-7.05281480304360	3.43651241689312	-0.60472544842887
H	4.34112748551363	-3.86328851947694	-0.42487794109978
H	-2.02594729618216	2.26330275037135	1.56974153314229
H	1.44488159617305	0.26252075730605	-2.86654581819290
H	-6.02688940176489	5.13763780174215	0.73783544233586
H	5.61554867056897	-2.16184423019448	-1.55497430802689
H	-5.90679836424795	-3.21819259451830	-1.62129926374070
H	-2.25219169732578	-5.68499900679057	-0.77667601789992
H	-2.00265541407188	-2.45397173546145	-3.34396462624520
H	-3.47943125070164	-2.11210885942968	1.35557355106106
H	-2.17260268892651	4.48126101934451	2.61485395189035
H	3.63451743856546	1.08343644111951	-3.63830749127015

H	-4.15998040963956	5.97382130845264	2.18861791504609
H	5.75476424344509	-0.09472869867293	-2.96552457092070
H	-6.04660126064266	-5.36631601580826	-2.88211066008937
H	-4.28235792958153	-6.87659556764669	0.04953285778988
H	-2.12260611191810	-4.61493178101141	-4.57927430127483
H	-5.51389594763135	-3.28860853161352	2.16029199392105
H	-4.14156066204662	-6.08980659881776	-4.35060341525498
H	-5.93843547657270	-5.67665494737573	1.50826381236602
C	-1.63994328335682	2.45004798322180	-2.60406963585054
C	-0.16665298871697	3.82979052965986	-1.07786864828756
H	-2.10965281144131	1.44897231153559	-2.61020769616917
H	-2.46378187845612	3.18129880720737	-2.44185277286976
C	-0.95498032891174	2.70290021976071	-3.94691661274724
H	-0.15603884731731	1.95522572621383	-4.12724208205557
H	-1.69237879420411	2.62767577733450	-4.77209839034497
H	-0.50099826300526	3.71350092795666	-3.99601709981048
C	1.30981659487600	3.99156177384756	-1.43986693009591
H	-0.77718763148629	4.60290736113673	-1.58852604347191
H	-0.30623983628337	4.00026165565223	0.01153192134192
H	1.94382171787496	3.24570759297136	-0.90333038248036
H	1.48520102172425	3.85013482132801	-2.52482019058040
H	1.68291202995306	4.99239326456019	-1.14115624889241
Ni	1.52248389234731	1.55094764505662	0.50760479233014
H	6.93828708368498	1.78908114227376	0.83456978970958
H	5.76140889621530	1.58515347639787	-0.52615083460540
C	6.30854641539565	1.06291255589054	0.27877976402456
H	6.94997380665745	0.27923694146586	-0.17503793275119
N	5.32525653713195	0.48136486543254	1.16179164628731
O	5.82875717422007	-0.12307511891778	2.31063157436870
H	1.76180669402095	-0.81261576409557	0.91762586973319
H	6.38451060976224	-1.92560138282149	3.06152958541320
C	-0.38323920870639	-0.96306583993930	2.54797009620081
C	1.74924893340941	-0.01196496801526	1.68312952605509
C	0.64694636937364	-0.08882422174747	2.63451494329184
C	5.94614688824992	-1.53974056183848	2.12154775735617
C	-1.53810510719226	-0.94796646289853	3.49199254223547
C	3.98441840367907	0.68930507175190	1.09470903872861
C	3.07998009554426	0.42126785397178	2.19496071212690
H	4.95211108389055	-2.00854769285946	1.95228888466717
H	0.65273250072948	0.62837590017044	3.47263788991627
H	3.45074047805510	-0.02423243556418	3.13308807186108
O	3.46708619062178	1.22041971240590	0.05607717182009
H	6.62169764003924	-1.78390595527178	1.27283748343643
C	6.77042943723506	3.85313184632787	3.33049398751209
H	7.79696961455370	4.22882208451942	3.46239585902411
C	6.01315641022924	4.24361903375925	2.21192745756736
C	6.20897285073254	2.98698646538566	4.28627918994033
H	6.44676522083784	4.92893703351539	1.46661487727107
H	6.79665340424248	2.68419684113917	5.16680585955166
C	4.70613153588972	3.76504583287413	2.04551421384025
C	4.90255951800386	2.50949452629163	4.11904772658478
C	4.14107855243373	2.88968766377559	2.99523990598108
H	4.09081997852408	4.05655369087838	1.18137560770316
H	4.46302358877861	1.82605901071329	4.86413791938348
C	2.76021394492450	2.36178449049990	2.77073205023369
O	2.00836126090459	2.94559816645443	1.88557678123103
H	2.27584270244972	1.99375341030480	3.71174203471936
H	-0.45671558561907	-1.70693627522135	1.74002335574424
F	-1.31586315956080	-0.19691705906439	4.59690325081861
F	-1.85278510989331	-2.20343728432150	3.90652879007943
F	-2.66964811612465	-0.44833586824255	2.90427977204702

TS (S,S)-17a'

Electronic energy: -4915.8506 Eh
Enthalpy: -4905.9768 Eh
Gibbs free energy: -4915.0104 Eh

P	-0.44444327898008	-0.45991044673198	-1.49108382649033
O	0.59793741188558	-1.18534870195251	-0.36114525864475
O	0.55315292772637	0.85030235432562	-1.87212919235662
N	-0.34859232693616	-1.27242067948688	-2.94891513544837
C	2.73824921775594	-0.19671781430703	-0.61302494496044
C	2.13150137859250	1.07490601504816	-0.12238085697339
C	1.92866469928478	-1.34882754182266	-0.68593154400241
C	1.00930447043419	1.59242557663591	-0.79434671720856
C	2.41321111263783	-2.64544082490484	-1.03797645658115
C	0.35768399673052	2.81281228622250	-0.44909532155204
C	4.11832766729992	-0.29540567744543	-0.97228336732119
C	2.66284427067243	1.79429834352498	0.99144124300981
C	3.81107870235311	-2.71232167144869	-1.36800119965402
C	0.92131409333798	3.51218820384822	0.67307456233385
C	4.61411598472328	-1.54783390261830	-1.33580640734634
C	2.05178544755705	2.99621973938299	1.34667204076354
C	1.60709523916978	-3.86841694888341	-1.10477575467451
C	-0.81903096165281	3.37268017127956	-1.11905728128183
C	2.24718091099517	-5.08763944076298	-1.53518109998204
C	-1.39863151837221	4.58038360604313	-0.58539942559808
C	5.02870971089327	0.87738865611143	-0.99302255338546
C	3.79792432738010	1.29465147898810	1.80585640417951
C	4.39955023372616	-3.95883175129954	-1.77302263365695
C	0.31114719722700	4.72043525969003	1.15242864213478
C	3.64688923388989	-5.09920620851393	-1.85815295467537
C	-0.80976013678174	5.22833318899999	0.55372935222532
C	0.21915588037474	-3.95039246810795	-0.79155648070951
C	-1.44201951687030	2.81561917470980	-2.27481744376773
C	1.49499536325745	-6.28807364722648	-1.64930844596130
C	-2.55978314284662	5.13956364649080	-1.18318309935321
C	4.63029419596388	2.11375655887767	-1.54872753264564
C	3.86086481632813	-0.04871210340882	2.23757254435333
C	6.33653296516355	0.76322954411641	-0.47230201744618
C	4.82727387959076	2.17741705435425	2.19811105351268
C	-0.49825722869056	-5.13744846962658	-0.92082069666247
C	-2.58750016949496	3.37904334255222	-2.83418736600244
C	0.13675386474312	-6.32076318627559	-1.35572003288236
C	-3.16273273019093	4.54379399431665	-2.28462096787765
C	5.50817205450408	3.20618641538803	-1.56375499502529
C	4.93358164649769	-0.49795758213406	3.01909604497657
C	7.21467711740488	1.85462689967329	-0.48969731071611
C	5.89944351838610	1.72877916200163	2.98064698480913
C	6.80192900220007	3.08330790836124	-1.02977880396685
C	5.96073146427711	0.38683538315839	3.38908307028905
H	5.66637556673535	-1.63795554654760	-1.64425762522152
H	2.41799728503059	3.54058423277109	2.22905222275461
H	5.47185496878985	-3.97007543639068	-2.02153103164188
H	0.76187580960039	5.21899491146862	2.02387774333341
H	4.10110777463299	-6.04992610142927	-2.17799003688154
H	-1.28168039582616	6.14879596735753	0.93139954794245
H	-0.31752016572406	-3.06847196775617	-0.44084639382677
H	-1.00447367140834	1.94261990027107	-2.76290922890932
H	2.01736095362105	-7.19870540959298	-1.98242467011987
H	-2.97938795282247	6.05946966367981	-0.74672777635835
H	3.62601713620716	2.21539280207111	-1.98418743317330
H	3.05122759886986	-0.74250124257299	1.97036969606512
H	6.65187461444802	-0.18522631742147	-0.01233713585387
H	4.80129252018444	3.22112916376563	1.85065240368042
H	-1.57254443761258	-5.12576019740875	-0.68310430641291
H	-3.04178963016621	2.90237172276635	-3.71594537068793
H	-0.43366965809442	-7.25655049758614	-1.45826173990617
H	-4.07093368063131	4.97945157269568	-2.72758759637883
H	5.18036934062703	4.16086315262424	-2.00318729023989

H	4.96374194616114	-1.54772351176002	3.34948716929874
H	8.22409051535635	1.74913739057493	-0.06335550386909
H	6.70035137414275	2.42982530294973	3.26155347445274
H	7.48831131974433	3.94398242575013	-1.03821575972283
H	6.80474061895731	0.03093577818727	3.99979750310795
C	0.77196286701442	-1.13629665945090	-3.89709798776820
C	-1.58282440432937	-1.93484706826141	-3.40477234424643
H	0.48214202232063	-0.43797691914649	-4.71534358873008
H	1.60773441701500	-0.64951463832885	-3.36216863068546
C	1.24141082814927	-2.46391726835811	-4.48703984764632
H	2.13206191791751	-2.28494924865225	-5.12217290041874
H	1.52443491250113	-3.18022479992149	-3.69155025997721
H	0.47071247109641	-2.94042994984087	-5.12530732423740
C	-2.35439879787383	-1.13271634979793	-4.44978498781403
H	-1.31874875651306	-2.94375159874875	-3.78651476915309
H	-2.21608218775463	-2.10899657701440	-2.51002136451708
H	-3.27843801827333	-1.67191808441838	-4.74052016704439
H	-2.65012134416794	-0.14719062159896	-4.03362760695966
H	-1.76147528835850	-0.96064481315304	-5.37123790144250
Ni	-2.30851612823138	-0.09563465012685	-0.76432445161525
H	-6.54113185480153	-4.00078292796808	-0.37575801629060
H	-5.72408965362714	-5.07835239461348	0.82626093382521
C	-5.56942448484458	-4.25801296045399	0.09544041915828
H	-4.85734799575233	-4.59210093891451	-0.68025479327313
N	-4.99708524032383	-3.09124549920224	0.73217024080176
O	-5.83060901008744	-2.44776700432334	1.64848969058954
H	-5.87413036673504	-4.05077178625187	3.02524607329659
C	-0.49348610984919	0.10126596980612	2.61744349876187
C	-2.08850914931843	-0.83729487575892	0.95942463840201
C	-1.61782352661969	0.20961629942764	1.86911172923930
C	-5.64148778960749	-2.96565099251913	2.97343922752531
C	-0.10548962194394	1.12433473695057	3.63419062936131
C	-3.87248175545124	-2.44246580286723	0.28292079842867
C	-3.58018538630864	-1.09338619370657	0.89250703551823
H	-4.60461260120182	-2.78782247907221	3.33245738349958
O	-3.16207225006206	-2.94440180696313	-0.60394446459728
H	-6.35507229429295	-2.40784840904344	3.60889867309653
C	-4.97020123930827	3.38803512476595	2.82812052432041
H	-5.06488327602646	4.21652086021574	3.54693327641781
C	-5.66417974531585	2.18400867606183	3.04297567119060
C	-4.16501649924737	3.53079188708380	1.68654479722443
H	-6.31097065167470	2.07046679472523	3.92703010170247
H	-3.62446388517420	4.47213675944785	1.50548620226489
C	-5.53511992292498	1.12655140413957	2.13068696709115
C	-4.04605752266226	2.47522341850456	0.76887851800800
C	-4.71357183652569	1.25874343727894	0.99402664680639
H	-6.08005837094646	0.18235619344692	2.29611062984171
H	-3.43161397885277	2.57501108933251	-0.13728715167660
C	-4.54000013952906	0.09901443734539	0.01549823971540
F	-0.54588361747921	0.77473596355777	4.87868753162942
F	-0.61429686958077	2.35415889833353	3.37337466419955
F	1.24367833607814	1.24879619996666	3.73744880368126
O	-4.07860350544065	0.45949074257799	-1.21152971554901
H	0.15138711174385	-0.79090835220918	2.57886427372537
H	-2.23577926931569	1.11649746062382	1.97423429448280
H	-4.03686403921618	-0.97600639084169	1.89148970866165
H	-1.47223694797864	-1.75371213010366	0.96457753625334
H	-5.49819315727876	-0.47896634687159	-0.02871859598673

TS (S,R)-17a’
Electronic energy: -4915.8280 Eh
Enthalpy: -4905.9743 Eh
Gibbs free energy: -4914.9901 Eh

P	0.34441751002864	1.08365577929938	-0.79020607589448
O	-0.96899240012527	1.36531599400881	0.26744696808456
O	-0.55422812725349	0.20850263899949	-1.95732674315801
N	0.57345335826722	2.48344479145342	-1.69516045780104

C	-2.91132931213708	0.31791413271216	-0.62833401871104
C	-2.15663965925624	-0.96641302900218	-0.68346377990793
C	-2.28066232385887	1.47766188580020	-0.12496653197138
C	-0.96069846045944	-1.00129238189743	-1.41965580409849
C	-2.96908466543290	2.71816410591652	0.07733946895513
C	-0.16258988457925	-2.16821188238229	-1.60042122407988
C	-4.27819056182741	0.38699998778715	-1.04393799396274
C	-2.61706354700669	-2.16424563833225	-0.05202091429416
C	-4.35913199424937	2.72927652151165	-0.29015447402336
C	-0.65667878826978	-3.35757122013169	-0.96564654526827
C	-4.96612872577447	1.58360056155031	-0.85551227095813
C	-1.86192323852294	-3.32547773379203	-0.22419352622027
C	-2.38253588864249	3.94041422299028	0.63515721481903
C	1.11791387440368	-2.21196675798775	-2.30287150744850
C	-3.22681602881217	5.09763450352269	0.80630351173853
C	1.86466462576885	-3.44339897173596	-2.28346307353081
C	-4.97895668108422	-0.75602873639561	-1.68376844063307
C	-3.82959223281540	-2.20785590431309	0.80338032600549
C	-5.15136124530445	3.91357949788146	-0.10791379382248
C	0.10535832462841	-4.57367541805519	-1.02420084933964
C	-4.61042516625245	5.05145887048775	0.42613119990101
C	1.32421116126508	-4.61078542890004	-1.64631520389301
C	-1.01745523410037	4.08641985862762	1.01834585957831
C	1.71125557811808	-1.10934901503680	-2.98750321742848
C	-2.69352473655499	6.30149197062882	1.34247282030616
C	3.14690251588360	-3.51018262487259	-2.88824058075636
C	-4.36575851694694	-1.52045390730391	-2.70152939499179
C	-4.08908112260940	-1.20286046748728	1.76134099125426
C	-6.30028562311897	-1.07910708145148	-1.30381526108416
C	-4.73565038393410	-3.28580218027014	0.69467971574509
C	-0.51758013147265	5.27920559784624	1.53646021633776
C	2.97872548144641	-1.20019695134547	-3.56513091988607
C	-1.35702402854593	6.40160111221564	1.70811045905073
C	3.71436350870291	-2.40185052886715	-3.50831800437564
C	-5.04859404014878	-2.58400392174078	-3.30723764886458
C	-5.22906672929832	-1.26852676032906	2.57377618686172
C	-6.98377579843399	-2.14106604295357	-1.91081781851062
C	-5.87500984447076	-3.35112825806067	1.50777317799939
C	-6.35864850422885	-2.90237145981951	-2.91151117101695
C	-6.12973537715331	-2.33948402520798	2.44812555227363
H	-6.01453160407716	1.66015132550158	-1.18022943766192
H	-2.17912348426363	-4.24880240235476	0.28341923537227
H	-6.21057520266940	3.87747862853214	-0.40563012273282
H	-0.30477157465268	-5.46857079085450	-0.53136005738047
H	-5.22356917967487	5.95471031379601	0.56931246418013
H	1.91859710809800	-5.53746783321682	-1.66414163730864
H	-0.33789209626020	3.24459163427091	0.88550685475601
H	1.14912405220778	-0.17995595740434	-3.10908744358646
H	-3.37096749919609	7.16199570485711	1.45960716751954
H	3.69221225821157	-4.46603788473924	-2.84481143908532
H	-3.34764780179123	-1.26842695653179	-3.03065656795567
H	-3.38282254336396	-0.37283337137354	1.88737380487798
H	-6.78070339080782	-0.51095231504766	-0.49317558672534
H	-4.56110248089115	-4.06208815173262	-0.06561806162527
H	0.54757324254815	5.34283921750930	1.80854973727255
H	3.39610307222992	-0.32300163525916	-4.08311390527167
H	-0.95827546523638	7.34120711556732	2.11977219309904
H	4.71472192774965	-2.46664417052442	-3.96270584043752
H	-4.55443935188158	-3.16573100758558	-4.10064158755269
H	-5.40734023194158	-0.47815030965321	3.31922165963371
H	-8.00746935523105	-2.38574182587751	-1.58817389685131
H	-6.57648114768563	-4.19235024654397	1.39665975337941
H	-6.89186499012426	-3.74115620389506	-3.38494174652688
H	-7.02632206576809	-2.38780029620137	3.08526248157417
C	-0.39910394850016	2.98092287060776	-2.68119854060134
C	1.88787060494403	3.12873870937934	-1.60609347074843
H	-0.06305257059866	2.69995070192204	-3.70619565742833

H	-1.34907862009994	2.43741644585364	-2.51851134675771
C	-0.64200538016487	4.48694472618619	-2.61202549253529
H	-1.44575263494309	4.76131529635877	-3.32423999424050
H	-0.95980177476618	4.79761617413508	-1.59805020755574
H	0.25893414196363	5.07054348834540	-2.88963306315021
C	2.73786007270680	2.98183260573459	-2.86621119102558
H	1.75440311930811	4.20148276724959	-1.34376381832232
H	2.41432276577687	2.65176948057637	-0.75187926524085
H	3.71880405516412	3.47915377133261	-2.72576598393050
H	2.92335596825106	1.90991712018831	-3.08129257658816
H	2.25554949875116	3.43714070407039	-3.75504733879707
Ni	1.99159830546759	0.09532368451170	-0.01052688369799
H	6.31457750739188	0.19964663647338	-0.58545228003100
H	7.17845622606930	-1.07010162439782	0.37108161157339
C	6.77636011890213	-0.03514204971443	0.39057529829766
H	7.60226167675196	0.68041743769382	0.58337579436342
N	5.74586957560715	0.08161639265528	1.39767005503850
O	6.12084939163652	-0.30604984755593	2.68212388053268
H	7.34935157181024	1.38182033550556	3.00789932528636
C	0.07585935723678	1.30068465447592	3.06835674476794
C	2.16173665175403	0.70455744179416	1.85413831600339
C	0.94461216965942	0.37944845418227	2.59122350434529
C	6.49932423500447	0.83143750274013	3.46663394911684
C	-1.21968860026510	0.95206030106967	3.71721128200935
C	4.41195971519703	0.19618935761092	1.14421490889936
C	3.39405512069239	-0.08559471114529	2.13179910816653
H	5.64201200733629	1.52372481451822	3.61498567523605
O	4.00621615053393	0.48952154428301	-0.03339831754745
H	6.81584983632492	0.41987322674302	4.44393282692534
C	6.57870319276076	-4.19967869468394	1.62083472494365
H	7.52596145684075	-4.76017270697266	1.65045681248145
C	5.87652715290047	-3.94178766847838	2.81186059858557
C	6.06395243033946	-3.74608670965749	0.39285751206715
H	6.27459570920833	-4.30019348550970	3.77382508032941
H	6.60723354896364	-3.95460528869119	-0.54250969628852
C	4.67118098532603	-3.22856739717538	2.77484601890478
C	4.85720577394759	-3.03434059447609	0.35607433494137
C	4.15347553392783	-2.76676111985834	1.54835328603073
H	4.11875152557569	-3.02174134156498	3.70625999812120
H	4.42985841567380	-2.67312290839121	-0.59080100188392
C	2.88201981420172	-1.98927851100350	1.48218470129097
F	-2.27047290284010	1.53205628429613	3.06175058164334
F	-1.28229862056463	1.40518283310353	4.99886634749897
F	-1.45942115028922	-0.38066703434493	3.74999321226990
O	2.29758610509432	-1.85380759799677	0.33860073038606
H	0.25663369557767	2.38196459647595	2.97011665076503
H	0.70998047480324	-0.68862655216935	2.73795229751099
H	3.66041367062441	-0.31140738044965	3.17681960085872
H	2.31797294958809	1.79255751159997	1.69311890340089
H	2.24709479207686	-2.09539293424230	2.39899800983635

(R,R)-Q’
Electronic energy: -4915.8820 Eh
Enthalpy: -4906.0005 Eh
Gibbs free energy: -4915.0399 Eh

P	-0.10590429863114	1.97391012609796	-0.81689447429452
O	-1.47033101460828	1.33351511106337	-0.05098414857516
O	0.17530706419219	0.67175377586444	-1.86195803960711
N	-0.56198644920521	3.08622828224647	-1.98666998344086
C	-2.31647113165037	-0.44003651544615	-1.36055875180140
C	-1.07032953716519	-1.17708235650691	-1.00475292026225
C	-2.50177989907891	0.84129513465497	-0.81635340304255
C	0.17842559453949	-0.60668647469243	-1.32123841841898
C	-3.66553387075632	1.63638282223229	-1.02191781931167
C	1.42235029795196	-1.28398730894750	-1.13872216203317
C	-3.32810714680992	-0.98681136290368	-2.20908919445326
C	-1.11012346246814	-2.45092397835201	-0.35590549233164

C	-4.64729345259159	1.07987775596054	-1.90975734040092
C	1.35164256724750	-2.53764823447675	-0.43936090390933
C	-4.46001685444192	-0.20930110105003	-2.46499901620223
C	0.09990783864878	-3.08185316814900	-0.06789463008561
C	-3.88588400842766	2.96135083928583	-0.44421900834481
C	2.72536551963340	-0.80591586972168	-1.60465581486863
C	-5.02452215090728	3.72044689568693	-0.89542204554262
C	3.90342731441669	-1.54402259121924	-1.22319869426141
C	-3.20486989430844	-2.32393310835340	-2.84247767286356
C	-2.38045634987154	-3.09874681819297	0.05931716292266
C	-5.79605893947255	1.85927961670210	-2.28170163248140
C	2.55833923583119	-3.22963981815809	-0.07864247822106
C	-5.96025249651410	3.13844408274126	-1.81863201799561
C	3.78675367107408	-2.73632857913213	-0.42974186604514
C	-3.06339604356183	3.55380774389893	0.55861462459520
C	2.90963534653936	0.31063519297855	-2.46826970536101
C	-5.24133013739682	5.03577100301031	-0.40277690821875
C	5.18309744412757	-1.12311026137263	-1.67378848603315
C	-4.31308814727095	-3.19926504850818	-2.85438686858067
C	-2.58673567080006	-4.46913424528575	-0.21126212208882
C	-2.00523597937554	-2.74582659855769	-3.45824902780205
C	-3.38410322431928	-2.38528704151489	0.75122121613053
C	-3.30591864346799	4.84186613843325	1.03198090927297
C	4.17341185413082	0.68583749005693	-2.91635390357944
C	-4.38871346224704	5.60258993113954	0.53790640348381
C	5.32408608630524	-0.02514279818663	-2.51432083517081
C	-4.22142873484043	-4.46539968638346	-3.44726860398826
C	-3.77190226696432	-5.10635401411620	0.17936601016889
C	-1.91349275478123	-4.01341923744859	-4.04920445481558
C	-4.56831091597844	-3.02493777826808	1.14249573702450
C	-3.01890451140536	-4.88057721432111	-4.04160092683253
C	-4.77063299202634	-4.38468405247389	0.85271721288581
H	-5.22415804276429	-0.58999165166120	-3.15915953992641
H	0.09013418246911	-4.02961853734532	0.49081926070618
H	-6.52930380012902	1.41087222489317	-2.96955715114768
H	2.46710895899269	-4.16622495913046	0.49231377250041
H	-6.82752259130194	3.74054642676319	-2.13155429631420
H	4.70832915416130	-3.26711322102843	-0.14411153003314
H	-2.23592362980226	2.98200821657721	0.98803860890866
H	2.04017253504726	0.88606716616804	-2.79715144735375
H	-6.11316152594298	5.59573916129537	-0.77619143521614
H	6.06404737371452	-1.70576079406810	-1.36115186946195
H	-5.24507966605905	-2.89364286144954	-2.35568435120079
H	-1.82050305583186	-5.02854311221371	-0.76877186588794
H	-1.14040674317727	-2.06774385573053	-3.48597677381024
H	-3.22712972897104	-1.32942154758931	1.00791794407613
H	-2.64609246754072	5.25997242511053	1.80797269148426
H	4.26932136987844	1.55266158192050	-3.58758329113832
H	-4.56870849855864	6.62265729388207	0.90995435235904
H	6.32008521064985	0.28195742526820	-2.86778148709753
H	-5.09178013820533	-5.13923330183291	-3.43145034731810
H	-3.92181695356706	-6.17135492719963	-0.05549923873228
H	-0.97126054630313	-4.32384483259721	-4.52652424365976
H	-5.33571994264510	-2.45542388117632	1.68890014023650
H	-2.94337108679648	-5.87772954037961	-4.50192915769375
H	-5.70397953293412	-4.88271944024231	1.15747781130344
C	-1.25878368880769	2.75340289702010	-3.23899148410722
C	-0.42891477355872	4.51744290200315	-1.68592592583032
H	-1.64194557528656	1.71873956168090	-3.16937430618147
H	-2.15078685748254	3.41357236950415	-3.31953268170297
C	-0.36448980880898	2.88952878256699	-4.47100805786330
H	0.48132451561640	2.17489190567591	-4.42030908894973
H	-0.94523727927800	2.67381287670036	-5.39056095387508
H	0.05266329987569	3.91228276437376	-4.57045722458758
C	0.48622211890497	4.80102255088273	-0.50150916955833
H	-0.02719830700350	5.02940371759852	-2.58671585468303
H	-1.43868617674585	4.94635980029897	-1.48925957907770

H	0.04422503764150	4.38232242881798	0.45742105457442
H	1.52695179470496	4.44086169791322	-0.67947196681515
H	0.56487420831901	5.88445716588995	-0.28640162455695
Ni	1.11256639908765	2.85122221602292	0.63472975018793
H	6.91597500829919	2.91650848969984	0.52295457168268
H	5.82261110011703	2.50969384317358	-0.86459011424635
C	6.32448082479688	2.11272611300749	0.03497524382508
H	7.00048095166120	1.28657181131199	-0.26325794842164
N	5.29196114907043	1.63206735879446	0.92755516204802
O	5.76622591297704	1.15767939546566	2.15438006488006
H	1.77637707887764	0.35967354764551	0.82587709705010
H	6.40953596166455	-0.52803921265791	3.08354031233818
C	-0.31795095339556	0.06493964615294	2.43145366239929
C	1.68951525934590	1.22383569214017	1.51255694139391
C	0.59865461556740	1.06736083576915	2.47502238066096
C	5.96378576293532	-0.26116876584096	2.10641339966876
C	-1.51761434661446	0.03704095117683	3.30249214648973
C	3.96441694052680	1.96430546250974	0.80894315981108
C	3.06561796512569	1.68184439683111	2.00454196695305
H	5.00145703483917	-0.80286989782539	1.97435223000317
H	0.50614008436182	1.81823337511196	3.27289235330629
H	3.52459449104632	0.91507590647184	2.66015782486925
O	3.54250221032716	2.52360536593623	-0.21603943280361
H	6.66209694599994	-0.54841144774494	1.29103089255030
C	1.98091708715949	2.71696645372365	6.97369847158206
H	1.74707368458073	2.63008744176857	8.04604564056393
C	1.15020784666696	3.46737404860029	6.12608750855535
C	3.11791417002681	2.08148745870196	6.44433038893354
H	0.26153175938665	3.97454005759631	6.53382670340883
H	3.78013893013390	1.49850684825431	7.10372188033043
C	1.44832457765421	3.57305414692738	4.75810714626538
C	3.41300139270850	2.18829545826849	5.07689432655589
C	2.57128306445424	2.92201271066230	4.21736356896840
H	0.81144667001887	4.15183081871539	4.07227934347886
H	4.31424477271337	1.70209659148175	4.66850095414433
C	2.89567427985922	3.06018047465194	2.72767778787197
O	1.95481838239772	3.79064680922659	2.00605381515763
H	3.89236550830456	3.57821614568477	2.69692970069396
H	-0.28877742738159	-0.72484819859923	1.66534598758234
F	-1.44069086325903	0.88101593436532	4.36081362437208
F	-1.76830229313447	-1.20933170472245	3.79149225258134
F	-2.66093955237435	0.39340118606428	2.61509731432101

(R,S)-Q’

Electronic energy: -4915.8761 Eh
Enthalpy: -4905.9975 Eh
Gibbs free energy: -4915.0349 Eh

P	0.04704632611666	1.86542904470198	-0.81561498129330
O	-1.28446882833365	1.25510091004929	0.02739371279142
O	0.06617055259017	0.68759916036056	-2.04125469223059
N	-0.42351871469639	3.17275149752417	-1.74807533170165
C	-2.27434432147734	-0.49069157246698	-1.22053473664782
C	-0.98139886023135	-1.21723115041710	-1.07640551254479
C	-2.39791645698902	0.77235960274484	-0.61633486678168
C	0.18953780775606	-0.61251642441542	-1.57144083852886
C	-3.59963472747417	1.53955447113680	-0.61488339360884
C	1.45939992002516	-1.26275182623052	-1.61630974561319
C	-3.39054094411954	-1.04047364334385	-1.92291905700660
C	-0.89779186879974	-2.50967469221572	-0.47082390790898
C	-4.69500834355784	0.97633867834104	-1.35437107011238
C	1.51810124478256	-2.54794680411528	-0.97417500620934
C	-4.56605469256707	-0.28965462984314	-1.97568435836888
C	0.35355522515669	-3.12537055122338	-0.41442460839721
C	-3.76886715673612	2.83583326417608	0.04157487919540
C	2.66192209321614	-0.73181205311985	-2.25906269059374
C	-4.99575748517324	3.55875122248641	-0.18215556810733

C	3.88858380104502	-1.47868891238015	-2.13991310092996
C	-3.33343349827976	-2.35744684549622	-2.60630650836861
C	-2.07415285558788	-3.19862039354460	0.11969601302387
C	-5.91362314134225	1.72214461750629	-1.50826226809569
C	2.77185892289166	-3.24273967602674	-0.87174763573782
C	-6.04712074574564	2.97414434583713	-0.96855008769042
C	3.91455828778280	-2.71918392172172	-1.41607986600432
C	-2.81475310362584	3.43023583165744	0.91858427693980
C	2.69850827927628	0.45512681577050	-3.04470286425961
C	-5.18278135090751	4.84037124856151	0.40272005229071
C	5.07074055016334	-1.01050039123423	-2.77256666223896
C	-4.40233184580924	-3.26955221571699	-2.46379425911587
C	-2.26516228914663	-4.57823724925290	-0.11656806929358
C	-2.23653698644957	-2.72575225760042	-3.41704326956692
C	-3.00463795215479	-2.52083103389559	0.93942749435370
C	-3.03023250390097	4.68341410136530	1.48873182273903
C	3.86966712204407	0.88684205906364	-3.66163865024682
C	-4.21195780779632	5.40830817110212	1.21950161291834
C	5.07054771601352	0.15805463212159	-3.52507712518210
C	-4.36929345375775	-4.51930042674669	-3.09643158524953
C	-3.35793081892159	-5.25815522556970	0.43702571429444
C	-2.20372972844491	-3.97623255004772	-4.04929430378410
C	-4.09466168079078	-3.20301794911236	1.49679196601009
C	-3.26654229621949	-4.88073165739854	-3.88702626192723
C	-4.27997013883290	-4.57234932770440	1.24417664763374
H	-5.42041741926372	-0.67274063083374	-2.55363814258364
H	0.45124238398585	-4.09295212311806	0.10007413839515
H	-6.73137132054466	1.26828798279117	-2.08892135476948
H	2.78801550580159	-4.21014229440637	-0.34659398509017
H	-6.97475647479740	3.55040702194712	-1.11000172813257
H	4.87241851203294	-3.25641481105508	-1.33454240188933
H	-1.90260510615499	2.88619682321611	1.17589353428377
H	1.78485850160809	1.04037663164254	-3.17909799165481
H	-6.12681888256669	5.37207263533576	0.20453295487315
H	5.99087229555494	-1.60624101670983	-2.66454361022024
H	-5.25118659210708	-3.00610320613983	-1.81501471634386
H	-1.56452517724064	-5.11328959406101	-0.77511260769442
H	-1.40747690581939	-2.01900878924717	-3.56417783309920
H	-2.87328689635692	-1.45274596961726	1.15684080949344
H	-2.26876318378207	5.10128956215420	2.16527954344121
H	3.85145386854971	1.80884186498568	-4.26282709284163
H	-4.37128859619604	6.40086684475424	1.66767319131749
H	5.99330679324570	0.50703014501971	-4.01320840008273
H	-5.20492207649571	-5.22238928137592	-2.95796062218472
H	-3.49692546166653	-6.32969107506092	0.22633433100976
H	-1.34198717414104	-4.24406769499314	-4.67993876965484
H	-4.79998845613649	-2.65707699405173	2.14190731178770
H	-3.23628815719262	-5.86474334139994	-4.37973147160640
H	-5.14011434816482	-5.10456433477348	1.67874593668888
C	-1.37998661677360	3.10862886683912	-2.86134043126920
C	0.00599852630409	4.49499873150556	-1.25814068494969
H	-1.79620302837486	2.08425347803182	-2.90192818298125
H	-2.23571738402978	3.78481489644065	-2.63898592531355
C	-0.74053184083704	3.45839844831437	-4.20398600080270
H	0.06203894409715	2.73540958917009	-4.45432307839295
H	-1.49978899748971	3.43058780942731	-5.01184358757422
H	-0.29775746133912	4.47576954127876	-4.19532019522499
C	1.52283989765112	4.65436248949167	-1.18809116250986
H	-0.40044027059489	5.24643531279109	-1.96454841659641
H	-0.46077520004191	4.71855079804527	-0.27261368099336
H	2.05561505791540	3.89559798414684	-0.54963592690091
H	1.98515138353604	4.54015762027237	-2.18900722784952
H	1.79968657455919	5.63909559508783	-0.76259663398368
Ni	1.54249263988886	2.39764011572611	0.52929604244507
H	7.10871310667454	1.42169205055275	0.43712159333815
H	5.96183228885685	1.30465279839193	-0.96231450449973
C	6.39237263676175	0.76518773697335	-0.10107684783708

H	6.91363609971936	-0.14139301927912	-0.46741118539004
N	5.29814735958273	0.40785372964205	0.77688730293062
O	5.69113424751849	-0.22941447581793	1.95575922964646
H	1.57329848958688	-0.14872222981863	0.87449005204834
H	5.98025260577912	-2.06773576874244	2.76197943832627
C	-0.42594121673572	0.06163167763828	2.56521412804080
C	1.71318602543969	0.74181705756468	1.51875860526508
C	0.65297115582253	0.88517426836189	2.51373795419323
C	5.60606258158105	-1.65134277168600	1.80732622951536
C	-1.58925867814533	0.27680450846518	3.46066482278061
C	4.06873954433795	1.00814197125744	0.72730246968153
C	3.17293515772801	0.91711252714405	1.95235134143625
H	4.55813713935622	-1.98142363950612	1.63644583307227
H	0.72983108352428	1.70825743532929	3.24227778871071
H	3.49588932998480	0.09144402093590	2.61929709886780
O	3.72663322193695	1.66766359016456	-0.27176004607217
H	6.24406507217138	-2.01468702509235	0.97247918317449
C	7.39712694934894	3.57947846750983	3.09548399217917
H	8.44961637491933	3.88774780530204	3.19431011372223
C	6.60329684243110	4.09300807591902	2.05650851904516
C	6.83567470398710	2.67797672689131	4.01666464640136
H	7.03307050552280	4.80849796161523	1.33761993942542
H	7.44853323558882	2.27767883201292	4.83945985426961
C	5.26016998235531	3.70258996161560	1.93478072059991
C	5.49368944615195	2.29028106586508	3.89223847089877
C	4.69609913931919	2.79047794790882	2.84550617690290
H	4.61998562961581	4.09521765067059	1.13200092426400
H	5.05631315305449	1.58344428298236	4.61626520308797
C	3.25848551780016	2.31836275721936	2.66308953464785
O	2.49561377942830	3.20044251661802	1.90178533028091
H	2.83309666048559	2.16719809872293	3.69147247691481
H	-0.53912505827634	-0.77979850203359	1.86445177608505
F	-1.42192316547330	1.30307617389194	4.32882977585821
F	-1.88718893887737	-0.83187772453140	4.19514318847298
F	-2.73013894097883	0.55382313380134	2.74216974162677

(S,S)-Q’
Electronic energy: -4915.8828 Eh
Enthalpy: -4906.0058 Eh
Gibbs free energy: -4915.0398 Eh

P	0.73608412162571	0.42078017720189	-1.55798848824679
O	-0.36947829960086	0.77997817342430	-0.30891228761866
O	-0.37336114106896	-0.46063292750117	-2.49315045852315
N	0.91356290683755	1.74213819510433	-2.56022347511439
C	-2.55144682506078	0.08999122442579	-1.00580565850891
C	-2.03051265441728	-1.30595482228008	-1.04073765341009
C	-1.67848118205336	1.12057697771344	-0.57887480924869
C	-0.85311947546436	-1.53649929698530	-1.76496424438860
C	-2.09504229763273	2.47416227060854	-0.37643005824064
C	-0.09897008776090	-2.74163649521332	-1.70448589632676
C	-3.90139380624645	0.41778186660386	-1.34159078676428
C	-2.62239144425262	-2.40039888789331	-0.33524478154744
C	-3.47255746512925	2.75399044977568	-0.68067333760899
C	-0.70403260370367	-3.81803316149912	-0.97466348804131
C	-4.32591704707772	1.73359623222661	-1.16291399919720
C	-1.96723843395835	-3.63397990789687	-0.36446209852207
C	-1.23566069767474	3.57059909872039	0.08480977389856
C	1.25842606438152	-2.89124609319927	-2.21533628762659
C	-1.80422907812385	4.89328860910407	0.18376836388940
C	1.96512111543146	-4.10967546837023	-1.92914907237285
C	-4.84431641052852	-0.58230496171735	-1.90265951293539
C	-3.86399737859221	-2.26746745473296	0.46609434730270
C	-3.99059080417497	4.08667034672458	-0.54034293601451
C	0.01737594245968	-5.04468479129231	-0.78462024879513
C	-3.18808078367882	5.11614116448057	-0.12880427063126
C	1.30741992777930	-5.17745908674673	-1.22858816703664
C	0.14181191977635	3.44265331365775	0.42895461584263

C	1.96095396309468	-1.89010146658721	-2.97766300409030
C	-0.99567009414861	5.99219361316995	0.58239722926954
C	3.32254735706166	-4.25230570830310	-2.33324522326256
C	-4.43914627460154	-1.48796989969113	-2.90850105894091
C	-4.04014659662568	-1.21129194892604	1.38685048470742
C	-6.18391630115815	-0.62367736495024	-1.45779212985056
C	-4.88457140582185	-3.23463707560677	0.34278338709560
C	0.91612774100443	4.53721985353657	0.80647092790875
C	3.30595086982468	-2.05584982084746	-3.33026221745856
C	0.35131616097907	5.82857315970060	0.88314135469153
C	4.00379150184486	-3.23051271931512	-2.98143591663530
C	-5.34277031994413	-2.42050320360147	-3.43570660408652
C	-5.21468462466869	-1.11647994472816	2.14487686980252
C	-7.08683415547312	-1.55556211928857	-1.98558841234977
C	-6.05931372544004	-3.13876105580209	1.10057335414199
C	-6.66839483601187	-2.46266390336108	-2.97242803513573
C	-6.23158758932388	-2.07556327117424	2.00104401491263
H	-5.35473337525738	2.00830596818508	-1.43967232992230
H	-2.38971587857155	-4.47200480487909	0.20890283771468
H	-5.05029414024398	4.25869140421606	-0.78344481636958
H	-0.47418361523265	-5.85867176799259	-0.23098169767071
H	-3.58759018938050	6.13766015187476	-0.03259773166027
H	1.87243873024322	-6.10399491139763	-1.04335583593901
H	0.62457050634334	2.46653854612951	0.39093331565601
H	1.39813439456395	-1.09212940508081	-3.47204064729985
H	-1.46506666171896	6.98668080774728	0.64332390532388
H	3.83728698835131	-5.19607454642039	-2.09430946069094
H	-3.40874143232096	-1.44910510701611	-3.28971244285347
H	-3.23983389789831	-0.47041871956092	1.52274741278588
H	-6.50708794066692	0.05798046791138	-0.65699701518242
H	-4.76540934490162	-4.05146347779159	-0.38482656600124
H	1.97993610896045	4.37262847987076	1.03607021259884
H	3.80282687741647	-1.27286556375704	-3.92233939008698
H	0.96610027259685	6.69194158570883	1.17994405400427
H	5.06364903812104	-3.34805124121284	-3.24919997110823
H	-5.01022502323006	-3.11651369045075	-4.22126355737832
H	-5.33312546712781	-0.28958191683425	2.86202371201843
H	-8.12175401463019	-1.58405354576946	-1.61159155146272
H	-6.85102113232563	-3.89374822763717	0.97812557677839
H	-7.37518902094484	-3.19984782535847	-3.38345252807288
H	-7.15513922612385	-1.99597254612744	2.59493571538377
C	-0.15133431508090	2.24159680708836	-3.45007775889865
C	2.25136919940294	2.34753499815477	-2.64625110053983
H	0.06544606360608	1.91579963921951	-4.49318283878643
H	-1.09409002395680	1.73828742259897	-3.16573480027101
C	-0.33592497669579	3.75636607880458	-3.40820809678017
H	-1.21051252527746	4.03363677900679	-4.03007575793016
H	-0.52119906483421	4.11261905705503	-2.37657403086581
H	0.54217541129376	4.29933100106012	-3.81149718608184
C	2.94775664387516	2.10143838821393	-3.98135893235026
H	2.16344145639960	3.43645207156001	-2.44214791343621
H	2.84945778048672	1.94005988022410	-1.80554292960075
H	3.95405901289342	2.56667614144385	-3.97565266610242
H	3.07413037462641	1.01438126870301	-4.16346960060024
H	2.38619241758581	2.53023644494025	-4.83644877892036
Ni	2.43491833988249	-0.66445536908763	-0.97273658954489
H	5.49726580162069	3.54920246723955	0.53852440670035
H	7.07866588499602	2.73971860800069	0.89460697224127
C	6.08720298111292	3.02031324773543	1.30828246640681
H	6.23363537895661	3.69307700278166	2.17882006746908
N	5.34273293180761	1.83569817733414	1.67712083966002
O	5.99844107399971	0.96840854336290	2.55141630192054
H	6.13219011811839	2.36662530399745	4.13018956872060
C	0.36415975202962	-1.42847435431366	1.95894154841337
C	2.27005645711984	-0.18559610405329	0.90520422056139
C	1.59958216079604	-1.39756976708331	1.39564221207087
C	5.76144883934078	1.34133340791916	3.91576479238111

C	-0.16327398176517	-2.65432774934475	2.61995167483755
C	4.20847006874680	1.40586948353863	1.02728437652671
C	3.71429941416615	0.00894272440144	1.38982194880625
H	4.68228306687063	1.27343444277694	4.17424973038577
O	3.65006207513703	2.14759483615039	0.20621009026537
H	6.33476796435530	0.61479266790274	4.52233535784366
C	4.71673644354761	-4.77051232964611	3.07896434241252
H	4.73348853590654	-5.69737042825410	3.67294489885702
C	5.18404350027000	-3.56616989780503	3.63353156217843
C	4.23322096953310	-4.77991921583587	1.76070097579311
H	5.57427892540339	-3.54976116516645	4.66342196378003
H	3.86586882321281	-5.71751043254388	1.31357156617446
C	5.15374114892743	-2.38187320576217	2.88108930536781
C	4.21172248542878	-3.59472318892996	1.00944592070971
C	4.65205498592334	-2.38060808344202	1.56490703335070
H	5.52923037357459	-1.44484094839962	3.31894821284268
H	3.83569511358783	-3.57090210961267	-0.02189919251002
C	4.59407034887922	-1.09774732812816	0.72635173437583
F	-1.51999019261892	-2.74539132308602	2.53914869739875
F	0.12328882031976	-2.66863574105928	3.95875386860597
F	0.35256958158726	-3.80464042422298	2.11095014622208
O	4.10587729435112	-1.31098061665932	-0.56587715456243
H	-0.25793892530692	-0.52660587199431	2.06626156137116
H	2.16803535515806	-2.33968957305297	1.36745567776143
H	3.74524385030593	-0.07620407949498	2.49915774494329
H	1.66443314731652	0.72029000989374	1.08738835177816
H	5.64844375347099	-0.72802746035416	0.66827681825050

(S,R)-Q’
Electronic energy: -4915.8821 Eh
Enthalpy: -4906.0093 Eh
Gibbs free energy: -4915.0395 Eh

P	0.46295962226404	0.27278628869932	-1.37676876982650
O	-0.70017667601713	0.65569140343432	-0.17981441009103
O	-0.62927224504912	-0.52851157833428	-2.39770216852351
N	0.75425305831011	1.62723258291067	-2.30199895162874
C	-2.85853241259994	0.08134356208844	-1.04321830685802
C	-2.39149660462606	-1.33229404259591	-1.05222236054717
C	-1.97644296355308	1.05650864131246	-0.51650168333005
C	-1.17583552074187	-1.59408656668562	-1.69558174749432
C	-2.36134992697595	2.41555089748206	-0.29252439270147
C	-0.43256041178151	-2.79634449715363	-1.54894223465425
C	-4.15737553274510	0.47641335618110	-1.48473220820366
C	-3.04107064002315	-2.39627968541370	-0.35315316698426
C	-3.68057293462567	2.77552051225670	-0.73835177958941
C	-1.07281807411159	-3.82828221154663	-0.78550788158749
C	-4.53173660953520	1.81052258794431	-1.32698824506122
C	-2.37374701241914	-3.62086798825242	-0.26508273709040
C	-1.51773916758614	3.44362291957485	0.32326175043086
C	0.94389345217152	-2.98088285988071	-2.01456397825341
C	-2.01182992445090	4.79740767221241	0.37855603427217
C	1.61544931950089	-4.21266364174420	-1.67197286472426
C	-5.09203719655757	-0.47889866756735	-2.13059178435602
C	-4.35929289640568	-2.23775636654525	0.31201515394883
C	-4.13134680599393	4.13621439104449	-0.63469908291631
C	-0.36142467367375	-5.04057294719377	-0.49008135222726
C	-3.32400031917538	5.11146985025704	-0.11393085526795
C	0.93450967140651	-5.21468476728314	-0.90126639288582
C	-0.23111201905279	3.20526061601837	0.88852410625638
C	1.66182876280582	-2.06106820106657	-2.86286988932294
C	-1.20540295079822	5.82944790202985	0.92988072274323
C	2.94477774884506	-4.43575896807267	-2.10385372063814
C	-4.64293281853653	-1.39939029416472	-3.10373783135532
C	-4.63443297260429	-1.15810760777422	1.18005216666048
C	-6.46369772753806	-0.46658370866563	-1.79549756008247
C	-5.36649381667000	-3.20225023967721	0.09333128251063
C	0.53714663136113	4.23466034472740	1.42800923753914

C	2.97592148384958	-2.33397559336492	-3.28310621578111
C	0.05917846563644	5.56321553369842	1.44130611761062
C	3.62897174785096	-3.51102991759601	-2.89210686517808
C	-5.53736807339036	-2.29486252982554	-3.70571249365109
C	-5.88790286920679	-1.04120694995278	1.79504345429559
C	-7.35700247785635	-1.36236221662452	-2.39709741190088
C	-6.62054472980006	-3.08368806792214	0.70802420265618
C	-6.89648202425816	-2.28473739682809	-3.35054793287681
C	-6.88806380167902	-1.99918876666169	1.55825810791367
H	-5.51225492430479	2.14249187264037	-1.69958586929828
H	-2.83159210464610	-4.43332953067446	0.31843628623470
H	-5.14221164029184	4.37557564764600	-0.99883908205718
H	-0.87375992827271	-5.81443724555972	0.10125439043682
H	-3.67001217318172	6.15490743223359	-0.05175767271979
H	1.48210365755082	-6.13660248293657	-0.65213602595609
H	0.16310019355447	2.18728523138148	0.90815311416115
H	1.12609843116300	-1.25041088880414	-3.36669456496390
H	-1.61130284476866	6.85310828322998	0.94775544932023
H	3.43462785088348	-5.37546152099166	-1.80462155303288
H	-3.58464112643026	-1.40022593169951	-3.40121560877678
H	-3.85045040642724	-0.41905643845614	1.39271430189067
H	-6.82245035461232	0.22731292114982	-1.02096508759639
H	-5.16934540724698	-4.03848857396813	-0.59450405773865
H	1.52613930235527	3.99999977697113	1.85129985705032
H	3.47637550880658	-1.61505195310684	-3.94957214575240
H	0.67322778658217	6.37378664160708	1.86226202489515
H	4.66330903608252	-3.70912888300061	-3.20894562313340
H	-5.17011587260533	-3.00289760773196	-4.46457635280019
H	-6.08262798402087	-0.19637753924769	2.47366228679490
H	-8.41894450395764	-1.35028116316596	-2.10753478726515
H	-7.39748897511862	-3.83863285246777	0.51238346746141
H	-7.59636981596043	-2.99345784905745	-3.81939012762021
H	-7.87330816167491	-1.90178559508545	2.03973962239663
C	-0.23919096629754	2.21276611724276	-3.22186719570143
C	2.11218360004772	2.18943478925579	-2.27537717033671
H	0.03761260253790	1.94690198594113	-4.26758950379630
H	-1.21034411648898	1.71944032584210	-3.03115524708335
C	-0.38621144998464	3.72656299421799	-3.09433065757288
H	-1.20718716918454	4.07077209214338	-3.75459366886990
H	-0.63416724635197	4.02213704141468	-2.05677738521286
H	0.53285433123287	4.26527325008823	-3.40025479783913
C	2.87650107770310	1.99989603827812	-3.58296865700613
H	2.05030080986038	3.26547029633906	-2.00355961991658
H	2.66944359238928	1.68693060448931	-1.45956897056229
H	3.90213501536145	2.40802031479882	-3.48105953412719
H	2.96304594445771	0.92084300777527	-3.82627352077214
H	2.39359021257864	2.51240144727253	-4.44010332893333
Ni	1.95924554078793	-1.07317696088231	-0.72824779292060
H	6.25621548352439	1.91464963463395	-0.35926979154386
H	7.47660516572627	0.95200862458911	0.56496921336730
C	6.60948246863317	1.64137388999425	0.65067787206353
H	6.91661744308185	2.55628751455756	1.19620793472952
N	5.51087260212400	0.98583539790619	1.33009823787161
O	5.82014913063749	0.53733254125736	2.61615837482950
H	5.94887738636976	2.48710208449745	3.41855129556634
C	-0.00118081750111	-1.12146669578978	2.29624076703730
C	2.08535470724092	-0.38847888334631	1.09339488062214
C	1.19117765020809	-1.38293066096430	1.69967942688712
C	5.44581308666536	1.51177104952021	3.59572058532646
C	-0.82586012002234	-2.18713432365551	2.92171646394521
C	4.43819208114250	0.43761184498434	0.66516838097786
C	3.57283110395717	-0.54359353085818	1.43535500280705
H	4.34380598326492	1.66190110680858	3.61925353374315
O	4.24073210144953	0.71867776306277	-0.52766099598433
H	5.78384141218260	1.09792968228025	4.56486575668524
C	8.25990193618204	-2.47157552222500	0.60075008008266
H	9.35177358967990	-2.57771321310339	0.50501396254282

C	7.63793863109619	-2.66750775788223	1.84592642601966
C	7.47836072966955	-2.15122279082864	-0.52234609590505
H	8.24232236168111	-2.92771957087516	2.72902796847197
H	7.95863206278634	-2.00478160939873	-1.50288073305990
C	6.24624980849144	-2.53738811776781	1.96422500706841
C	6.08593151350407	-2.02205450930189	-0.40084101351648
C	5.45880021030636	-2.20432832443064	0.84643731854867
H	5.75898673829316	-2.69060940208552	2.94089593170343
H	5.45648700357902	-1.77381077973427	-1.26667758078490
C	3.95651913739096	-1.99504405351573	0.98323465843649
F	-2.15202351411820	-2.04404803070885	2.62970051916569
F	-0.75223264497736	-2.16571081703451	4.28831752615094
F	-0.46675062488915	-3.44109156564401	2.53827692112859
O	3.28758964375873	-2.24020946457337	-0.20958842396464
H	-0.41021107073546	-0.10314236153311	2.37757364778605
H	1.52789270102802	-2.43244649383600	1.68263508830646
H	3.73709587309083	-0.41105804016252	2.52410540271835
H	1.72986211353156	0.65049004583016	1.24067454758541
H	3.61252315059494	-2.67376153836707	1.81016560915727

K•BEt$_3$

Electronic energy: -5178.7065Eh

Enthalpy: -5167.7918 Eh

Gibbs free energy: -5177.6774 Eh

P	0.45554644345195	1.54128643118990	-0.96650230859824
O	-0.71699399661638	1.27992929119558	0.25419910324899
O	0.11531528042630	0.21640190633242	-1.96077945331052
N	-0.17209299801104	2.71819520750234	-1.99321342290815
C	-2.36118800407696	-0.08490675583442	-0.76606659924717
C	-1.36266681285907	-1.19279791264667	-0.75801866348041
C	-2.03341434505889	1.12532538698052	-0.12472745190445
C	-0.10835528292657	-1.01822215564688	-1.37063147403852
C	-2.99069159809484	2.14603025236572	0.16157981634123
C	0.87863123549613	-2.04808009896107	-1.46349400999142
C	-3.66625102238947	-0.25406242092502	-1.32038686837133
C	-1.67555450592086	-2.44300947869775	-0.14043185775664
C	-4.30442654910726	1.93556187319014	-0.38691150460701
C	0.53277015529658	-3.29248871708784	-0.82838490968676
C	-4.59639607622358	0.76858535208909	-1.13114181072332
C	-0.71971666284554	-3.45545073395872	-0.19322749223636
C	-2.74160409792895	3.34769811691622	0.95997025062796
C	2.18808356718911	-1.92887737247421	-2.10968137761555
C	-3.80558145301613	4.30925212364544	1.10962331330440
C	3.10167354418885	-3.04349499533594	-2.02099146206851
C	-4.04484842389112	-1.45598975948484	-2.10461256569168
C	-2.95982482956588	-2.70347063890057	0.56063559683928
C	-5.32798423587512	2.92829775230680	-0.20758649422314
C	1.46868023101518	-4.38161135597193	-0.80199616123018
C	-5.08583859807569	4.07383641292520	0.50229793576692
C	2.70889504284629	-4.25834605620547	-1.36521536047876
C	-1.51550291780358	3.63312606657182	1.62658111401637
C	2.64088992843049	-0.78381162399369	-2.82762441035234
C	-3.59595603689325	5.49059973887861	1.87117867691850
C	4.40541645118956	-2.94963951350377	-2.57514237454039
C	-5.31116884413965	-2.05410667166071	-1.92180418266842
C	-3.65984260763321	-3.90416396699530	0.30962354291174
C	-3.16910566663764	-2.01265664695998	-3.06377722721458
C	-3.49250056547501	-1.79427839968414	1.50191619331623
C	-1.33912641738982	4.79432713958279	2.37558502146636
C	3.92348259924484	-0.72487391973243	-3.37083113860554
C	-2.38011484248565	5.73969736401734	2.49813338665363
C	4.82447638234578	-1.80106760133057	-3.23341372980250
C	-5.68572221246572	-3.18324214563030	-2.66209365630186
C	-4.86484471955804	-4.18432420544242	0.96787877218071
C	-3.54302226684433	-3.14326686388453	-3.80280470030637
C	-4.69883487976489	-2.07523148904795	2.15839320990360
C	-4.80059940813109	-3.73667777887441	-3.60146232908728

C	-5.39289101763982	-3.26740570833195	1.89100308682737
H	-5.59549694938467	0.67839009137264	-1.58295825115143
H	-0.92705278945179	-4.41553966086430	0.30271187830608
H	-6.31487040741718	2.74191896013162	-0.65815354535909
H	1.16419537272617	-5.31437803969728	-0.30332350300730
H	-5.87413327091147	4.83141930550260	0.63302339727082
H	3.43268617857354	-5.08582024348585	-1.32233591922045
H	-0.67817259746586	2.93712133935548	1.55388964540005
H	1.97317483034215	0.06811619476823	-2.97684472817246
H	-4.42785463888959	6.20680440436676	1.95958409427489
H	5.08239305821405	-3.80964092449162	-2.46162384792953
H	-5.99368492802122	-1.64855471090715	-1.15986816807578
H	-3.26937624005167	-4.60761900668514	-0.44137188355955
H	-2.19133999542892	-1.54338727911121	-3.24406527813428
H	-2.93734240535585	-0.87672508325890	1.73944329939409
H	-0.36941355018225	4.96331964685179	2.86844332011800
H	4.23377576095781	0.18155273338205	-3.90975767387669
H	-2.23341801309050	6.65914132311168	3.08503300470108
H	5.83999519118052	-1.73363380303106	-3.65167164620669
H	-6.67147005541509	-3.64340599856821	-2.49358772723956
H	-5.40286513972915	-5.11891133434823	0.74667869807959
H	-2.84832657273997	-3.56013144854947	-4.54810269521925
H	-5.09746724914601	-1.35774619858299	2.89225992163569
H	-5.09141483963541	-4.62726801558398	-4.17950612453028
H	-6.34249698375316	-3.48278199300110	2.40469913908809
C	-1.13929619760722	2.42404119434291	-3.07265842127913
C	-0.16785982145560	4.09108032469731	-1.44638295947351
H	-1.64163789203803	1.46005231244985	-2.87219104399961
H	-1.93414934650504	3.19762054006821	-3.02394423594802
C	-0.49499659839996	2.38872680073425	-4.45668347509469
H	0.28059773639294	1.59848821112640	-4.50462468906469
H	-1.26060108881134	2.16678427713795	-5.22837980841550
H	-0.01391715706079	3.35280902769436	-4.71245433284240
C	-0.08825472900101	5.20592246947258	-2.48383208299746
H	-1.08105055401853	4.24070409393208	-0.83059101123815
H	0.69319475177728	4.16478957371764	-0.74834060270632
H	0.81564211671622	5.12310486279834	-3.11377440810633
H	-0.98013748334304	5.22219380643024	-3.14167193349812
H	-0.05416033702838	6.17937577496884	-1.95501032967757
Ni	2.46059024405490	1.27708173199142	-0.11657757286334
H	-1.80487665724215	1.54938445851646	3.58433616961911
H	-0.27129378882312	2.22393082241202	4.26030375104037
C	-0.81602861313808	1.29369307652495	4.01993904881144
H	-0.96420454550798	0.70375860405475	4.94768268138302
N	-0.01377069205068	0.55421609587738	3.07127718652482
O	-0.60777901851430	-0.61451093161963	2.59604487157268
H	3.62976907261137	0.75746721164674	2.11329789059251
H	-0.78124047874104	-2.60439526351625	2.92298644182322
C	5.07447574010569	-1.40422291610985	1.41115800050046
C	3.09982684857548	0.08373369367423	1.41720783077041
C	3.80389369656610	-1.13172661670846	1.03028109599499
C	-0.20634444019927	-1.76228285958765	3.35323021714999
C	5.83058301033309	-2.60505256249037	0.95553031819180
C	1.04480832226374	1.08716331290923	2.37340121746934
C	1.68332748802625	0.18348478604643	1.36605440894329
H	0.88221883308776	-1.95722946058098	3.24138456089526
H	3.26193624742720	-1.85566074705815	0.40007296522408
H	1.10082900403259	-0.68111160032803	1.01417540537298
O	1.48624263108008	2.21793794366933	2.63772878857385
H	-0.46030622688061	-1.65312418746263	4.42960036893104
C	7.36838994859289	2.64748490988736	2.20626179101983
H	8.13636950064973	2.96578216055094	2.92781096245358
C	6.10548044201319	3.26816634726109	2.20132407569960
C	7.64110625561529	1.61205118012159	1.29850411544102
H	5.87934030930523	4.06623161535147	2.92459761471221
H	8.62286685219315	1.11446879849848	1.30452365198969
C	5.12813413818541	2.87058878183074	1.28261657316970

C	6.65887085613473	1.20456095857854	0.38381105879299
C	5.40075798571524	1.84496753953280	0.35077627513005
H	4.13276344350881	3.33491980233877	1.30087413570109
H	6.86475063266164	0.38704640323449	-0.32300852011910
C	4.41233440579318	1.36584674496209	-0.63905777375754
O	3.65354319358256	2.15687896062012	-1.38310246656092
H	4.63993840818066	0.37488619713413	-1.08879693635528
H	5.65110371299988	-0.71123711680477	2.04217956894392
F	5.03231524921531	-3.62319350697692	0.54783139490018
F	6.63392509493259	-3.08914165106327	1.93694438415674
F	6.65385893772574	-2.31167167674855	-0.10054059264283
B	3.98679534460840	3.79487911193991	-1.98270623183878
C	5.60493658650914	3.83866650026553	-2.12551473532900
C	3.09134877252683	3.78685476852002	-3.33659930610559
C	3.35751868392627	4.83459716470040	-0.87615514379662
C	3.49656171452679	3.00960942229581	-4.59187926993995
H	2.97444434755839	4.86200591156656	-3.61362085816351
H	2.07120855891045	3.46363705670253	-3.03876023383214
C	4.29025328992723	5.89756069096132	-0.28126784363965
H	2.90153570129841	4.24165366268577	-0.04927796965550
H	2.49443731198916	5.36276504683299	-1.33756589166553
C	6.35551205887750	2.73565951578543	-2.88335769669139
H	6.08083396851384	3.96829756754561	-1.12982125781430
H	5.79199997351874	4.81301311375500	-2.63895818937697
H	4.43166872100999	3.39801855000794	-5.04573439059101
H	2.71676395573092	3.05098529071391	-5.38395523448365
H	3.66908261071812	1.93652914762304	-4.37357406934236
H	6.15016479849446	1.72566122439661	-2.46858968955535
H	7.45797105001710	2.87067602362497	-2.83618243959234
H	6.07863625812610	2.69237107173306	-3.95433794169870
H	5.15058575377956	5.44978167369084	0.25770056092018
H	3.76994654740181	6.56766110057928	0.43737424057032
H	4.72052127964037	6.54657243726951	-1.07373226889467

TS (R,R)-17a•BEt_3
Electronic energy: -5178.6931 Eh
Enthalpy: -5167.7725 Eh
Gibbs free energy: -5177.6661 Eh

P	-0.05692588030257	1.08048054222057	0.63284202993538
O	1.09808103453188	0.69117696025026	-0.54642553404558
O	0.48001870912062	0.05361255798202	1.85776954430648
N	0.33268634707624	2.53184923905692	1.35508076251496
C	2.89909265157621	-0.30436477815567	0.64788107556491
C	2.00155814138165	-1.47185348111346	0.86978591248765
C	2.42876113426008	0.73580332313706	-0.17256898204040
C	0.76935871212341	-1.25872412986402	1.50854896314420
C	3.25033518744163	1.80735777980505	-0.62176928113011
C	-0.15079128987162	-2.30093008642318	1.82324295666404
C	4.22049202142490	-0.22749167112325	1.18449947730769
C	2.35025090983117	-2.78563769956241	0.43434319011309
C	4.53732048109186	1.91545386610886	0.00518028846839
C	0.19304485559580	-3.60101029060081	1.31170170570821
C	4.98667255594098	0.90224760214608	0.88629791936016
C	1.42622370648137	-3.80960271498407	0.64938157112245
C	2.86962436975606	2.77199863414462	-1.64642654413108
C	-1.38543159917359	-2.14086879973919	2.59143626739926
C	3.69770414839795	3.93412927607141	-1.83063025676407
C	-2.28940909241637	-3.25925405379512	2.67402100235251
C	4.78434922731450	-1.29225809363474	2.05165398860109
C	3.62788013512912	-3.08442868578732	-0.25969090207027
C	5.34539289912588	3.07985451401143	-0.23779155612921
C	-0.74124388459534	-4.68657624865706	1.43467741825484
C	4.91797028500160	4.07207212388084	-1.08209653306875
C	-1.94819912029486	-4.51181578822112	2.05843776740990
C	1.77225955061272	2.59036806153225	-2.53515881932828
C	-1.73774177080845	-0.96233305933324	3.30869881925368
C	3.33781904258880	4.90890628251889	-2.79962786912858

C	-3.50352733595793	-3.13730093447374	3.40003520011936
C	6.10246446576105	-1.74857643919618	1.82974387793009
C	4.12767473396670	-2.26263933161474	-1.29440807917419
C	4.03975784776873	-1.86725494393429	3.10557239459550
C	4.36891316856513	-4.22858260496616	0.10817661090418
C	1.47146991960864	3.54076316924385	-3.50842763850920
C	-2.92120899393736	-0.88068836649579	4.03891756415426
C	2.23356392701977	4.72486180382888	-3.62491504372533
C	-3.82322201366716	-1.96529757900665	4.07499199206417
C	6.65480421972836	-2.76152607408731	2.62479614237196
C	5.34114939705737	-2.56888262771061	-1.92546981854358
C	4.59290435857104	-2.88016703207560	3.90092507830944
C	5.58073100707668	-4.53608532734288	-0.52488538297654
C	5.89995964539979	-3.33596985059155	3.66017530044655
C	6.07555656893013	-3.70367879954456	-1.54122833151792
H	5.97376751867326	1.02165565503714	1.35781779407832
H	1.64197333047608	-4.81447342069136	0.25762408324779
H	6.31023635126066	3.16141784602317	0.28595470186483
H	-0.46497189670590	-5.66057870390795	1.00229743720262
H	5.52922522469439	4.97388647174457	-1.24204217536563
H	-2.66775782900123	-5.34201035987995	2.13125037235944
H	1.17249775545341	1.67572227585073	-2.48370402785506
H	-1.05995720805581	-0.10430439108377	3.30604399794018
H	3.97325830598557	5.80216690049631	-2.90610218941134
H	-4.18257480968835	-4.00378098892513	3.43161138501911
H	6.68389650398526	-1.32561718240227	0.99684398048053
H	3.55574960280447	-1.38394725525679	-1.62159360210967
H	3.02276956420334	-1.50540859705762	3.31308518450447
H	4.00450800134301	-4.86346438198452	0.92991733659613
H	0.63371775508979	3.35199619612811	-4.19539255566044
H	-3.15204500490446	0.04667049450088	4.58413016637978
H	1.97379361175238	5.48005828108782	-4.38229419945803
H	-4.76507248736314	-1.88595709345665	4.63862657751986
H	7.67819510861301	-3.11457820486138	2.42523264855213
H	5.71503103785475	-1.91507340694342	-2.72851964340734
H	3.99924212700042	-3.31300132673811	4.72077610991479
H	6.14989470495443	-5.42452737856805	-0.21099928320773
H	6.33046278603020	-4.13580748662981	4.28210707324498
H	7.03145355321744	-3.93828408927759	-2.03427283198173
C	-0.45975894959388	3.71899671237448	0.99102489075784
C	1.39437770904073	2.70482003174976	2.36069852339461
H	-1.38134472286732	3.35998466146333	0.48811564129930
H	-0.80647278615730	4.20668191295904	1.92690319055652
C	0.26748452263408	4.71392299412489	0.09228587411020
H	0.53146588064227	4.25504466467987	-0.88024841851624
H	-0.38874027734755	5.58670194474553	-0.09995235406432
H	1.20061120832937	5.09298503595492	0.55665580451939
C	0.85624933607609	2.77201080668757	3.78929119010151
H	2.11691131038375	1.87259836274886	2.26640938002713
H	1.95428034276734	3.63007683251102	2.10568931668330
H	0.12897050129274	3.59874649556086	3.91985965818241
H	0.35363043749997	1.82328697883574	4.06402513088208
H	1.68871138414839	2.94129328512996	4.50192108143106
Ni	-1.95671230628394	0.90498663081262	-0.12762719114371
H	3.64619642327162	-3.13334669539259	-4.10905712546571
H	2.69812065503129	-1.96011815008311	-5.11646332736035
C	2.66334741796342	-2.92681163897285	-4.58192186142671
H	2.42920893271100	-3.73547888706197	-5.30645025467534
N	1.63792664238452	-2.81462951015664	-3.57209235939009
O	1.50912720280543	-3.90859337099563	-2.72089505988778
H	-1.23312880077125	-0.15493542802477	-2.90769774698664
H	-0.48078470616787	-4.38225860253942	-3.23408172653258
C	-3.41441173104743	-0.47603129114937	-1.12356002892328
C	-1.06513060027236	-0.86692356233320	-2.08020676271875
C	-1.93236097033247	-0.80551754691432	-0.91267273387223
C	0.53270197986119	-4.83874960721147	-3.20663950115465
C	-4.29389447923173	-1.20822199097609	-0.11763528127882

C	0.89221660995109	-1.67508974929348	-3.33811597125716
C	-0.03195647302912	-1.74339134955985	-2.18156028557434
H	0.79865268935453	-5.22270077225159	-4.21521912657233
H	-1.74426097141952	-1.60601381922572	-0.17310816201142
H	0.15694026900611	-2.48858026243604	-1.39453730633022
O	1.00432519660295	-0.67610739863242	-4.06586099220376
H	0.54127245429192	-5.67584779263752	-2.48267863001886
C	-2.89092226571075	2.38806855784360	-5.35447060876361
H	-2.63326941131176	2.70579239084746	-6.37624498925937
C	-2.28886846379409	3.00787615768050	-4.24868052879787
C	-3.83539618297291	1.36830803543960	-5.14895676516030
H	-1.56748263361331	3.82477608421407	-4.40018570867743
H	-4.32581459076306	0.88673439303465	-6.00868760741813
C	-2.61368635934226	2.60530366065071	-2.94348406089690
C	-4.16593517916445	0.97129383067013	-3.84560620490170
C	-3.55323633017549	1.57923387777057	-2.73216871357358
H	-2.15551223298830	3.10247462692702	-2.07670922506296
H	-4.92409159076091	0.18480388920744	-3.69351645785084
C	-3.92942499998330	1.14650303449553	-1.32205023022945
O	-3.51080960346026	2.06557210063753	-0.36639187124234
H	-5.02550926899485	0.98598030769898	-1.25417777399912
H	-3.70250765165065	-0.85723513117451	-2.12222428767943
F	-4.11458344303535	-0.74754483017720	1.14131789490372
F	-4.02149017736815	-2.53864306875245	-0.10262812133727
F	-5.61009002282669	-1.08480132034267	-0.41953630746889
B	-4.40969147267995	2.80290965546491	0.80549061946915
C	-3.65447296239886	2.34850770097031	2.19766408145074
C	-4.25309006267618	4.39092630832902	0.45360539631733
C	-5.93698312704076	2.24434687464952	0.68198586745032
H	-4.09831599353081	1.40068517985211	2.57686012808017
C	-3.58838394208083	3.36064883242613	3.34599516927863
H	-2.60600250889911	2.06182602030533	1.92354154308694
C	-6.89613319678196	2.89730608983169	1.68741352390730
H	-5.96569468919427	1.14305412740860	0.84129570344267
H	-6.34207930067299	2.40920638814407	-0.34588929029672
H	-4.67882727308930	4.98300081860123	1.29673225998050
C	-4.90236385402875	4.85449271073429	-0.85589643090547
H	-3.17223053859633	4.66685869093170	0.43995460315648
H	-3.07658621356486	4.29883447911298	3.04352188489586
H	-3.04697579778449	2.96922297094680	4.23662328498681
H	-4.60223683022664	3.65598836513722	3.69197312171486
H	-7.93756424967323	2.51505099898200	1.61006610139455
H	-6.94382572471519	3.99866210354986	1.55097563509141
H	-6.56595768053223	2.72274745977595	2.73361495777488
H	-4.51249867676765	4.28382978145920	-1.72567863086838
H	-4.73461912919662	5.93214912042919	-1.07529041269880
H	-6.00234312698609	4.69803163618740	-0.84387630540285

TS (S,S)-17a•BEt$_3$
Electronic energy: -5178.6927 Eh
Enthalpy: -5167.7718 Eh
Gibbs free energy: -5177.6638 Eh

P	0.03357728266700	1.18365847271347	-0.56329085709955
O	-1.28639164304200	1.16079403714512	0.52048063673986
O	-0.52514192289305	0.02872868754834	-1.64888494544191
N	-0.09964547149691	2.52306683352864	-1.55422775925469
C	-3.11982252564428	0.41949967859784	-0.78301911146576
C	-2.49740859419867	-0.93450138940145	-0.73455605214105
C	-2.51593815160120	1.45265260958431	-0.04381644137558
C	-1.16837168014848	-1.10586323749867	-1.16464353716143
C	-3.08264902239364	2.75020915757814	0.12036096720965
C	-0.49691541016619	-2.36593755232963	-1.18397960021958
C	-4.32520980148526	0.69194360003891	-1.50077758632852
C	-3.23062416065006	-2.06462760663730	-0.25694439992737
C	-4.28472065433734	3.00037077693070	-0.62633420045099
C	-1.26249996278482	-3.47756545664286	-0.68920506508594
C	-4.86470783408291	1.97670263387861	-1.41294317008902

C	-2.59521365498176	-3.30305828609882	-0.25507045984094
C	-2.52315074676311	3.81727741077951	0.94916402189855
C	0.87013171202292	-2.60654688610645	-1.65331621637055
C	-3.12995692238548	5.12247570565529	0.89052406972928
C	1.42817436296682	-3.92781239491431	-1.49083350114847
C	-4.99058954067636	-0.32185444288245	-2.35627881294088
C	-4.61892526809872	-1.96402318343457	0.26102117988218
C	-4.87294398724104	4.31190776860179	-0.61842578720748
C	-0.66428136496440	-4.77684948260122	-0.57324988904271
C	-4.30350071354737	5.33756597238620	0.08921035075846
C	0.63973457415777	-4.98710960785709	-0.92772466440752
C	-1.42167058453011	3.65319147715688	1.83691952157575
C	1.68891891714513	-1.64137263368929	-2.30895219707836
C	-2.58167712460744	6.19296534401994	1.64761092771565
C	2.74909397337815	-4.20795608283158	-1.93155055149033
C	-6.39593950697335	-0.45705398144670	-2.32553206217726
C	-5.58803324885727	-2.90728242430022	-0.14522198012995
C	-4.25131920854135	-1.15047906369741	-3.22981610942504
C	-4.99697954409039	-0.96254463199181	1.18275330530123
C	-0.90756811530298	4.71586127024604	2.57602093619244
C	2.97259979262239	-1.95260856648022	-2.75562562912068
C	-1.47812207004175	6.00356441392913	2.47256629405077
C	3.52069108737970	-3.23699220963916	-2.55771381971366
C	-7.04354033362177	-1.40379992654032	-3.12985939105241
C	-6.90109981021569	-2.84244438352777	0.33932993184360
C	-4.89964551464280	-2.09772187656950	-4.03392240714388
C	-6.31093632506507	-0.89690252455652	1.66629863443134
C	-6.29730067656023	-2.23248247239837	-3.98327743483284
C	-7.26988068131160	-1.83234163443041	1.24233274595719
H	-5.76145238486337	2.22224508598841	-2.00128785667163
H	-3.12317515164623	-4.17583170054749	0.15545387077791
H	-5.78524549337827	4.47501040561979	-1.21240143379994
H	-1.27959169313871	-5.59365710884080	-0.16753755627650
H	-4.74722253372436	6.34514154355101	0.07294440073533
H	1.10279076235383	-5.97996199834342	-0.81698630870214
H	-0.96689692803742	2.66594180324775	1.94367839306157
H	1.30415432454899	-0.63606539653635	-2.49796256042148
H	-3.05991615585839	7.18228682750300	1.57382127043776
H	3.14147002483001	-5.22608680803925	-1.78165788485674
H	-6.98157260184503	0.16374117205285	-1.63101692975582
H	-5.31097038349643	-3.67788899128753	-0.88026227138205
H	-3.15927097222824	-1.03898571767464	-3.29048372517674
H	-4.24854726226548	-0.23908189110060	1.53600137293018
H	-0.04595408044972	4.53945130602819	3.23772013684077
H	3.56431182241351	-1.17708659636730	-3.26313240798301
H	-1.06330466232450	6.84411255380935	3.04924762182013
H	4.54093098532495	-3.46519260720823	-2.90095548667233
H	-8.13844693472163	-1.50536765480592	-3.07882413623931
H	-7.64568070840376	-3.57775678082007	-0.00235646427949
H	-4.30779398787579	-2.73198257044904	-4.71163419118106
H	-6.58629880455692	-0.11196569228359	2.38744167409376
H	-6.80393345256516	-2.98087732533328	-4.61186261042039
H	-8.30245938170790	-1.77620110965125	1.61995392302009
C	-0.95412351376730	2.57499293574974	-2.75761577882006
C	0.47046677673031	3.78580657212309	-1.05026815322631
H	-1.82365688180156	1.90592618003985	-2.61589094491254
H	-1.36457419430486	3.60552529459413	-2.81273420480646
C	-0.22155389344767	2.20532386488195	-4.04645259917919
H	-0.91385711251210	2.27883618664241	-4.91003818405706
H	0.64204422943412	2.87126036315437	-4.23692468192709
H	0.15285662992454	1.16382278098412	-3.99546212875433
C	1.27404700373267	4.58127110518403	-2.07195219851753
H	-0.35370028627831	4.41303295924887	-0.64273910912172
H	1.11848137793244	3.52839468531001	-0.18426744650666
H	2.11367756161875	3.98988431420814	-2.48047705256183
H	0.64265288465406	4.93239770368781	-2.91267094417929
H	1.69563366853056	5.47927434391278	-1.57802642489225

Ni	1.77748015132168	0.78356576344525	0.44247240792283
H	-0.71645346826405	-5.57458570833207	2.69722285270581
H	0.63725347632130	-6.30438431432381	3.64418823394002
C	0.37377730035160	-5.75118138778357	2.71829920530788
H	0.66971698697010	-6.35651647018182	1.83576197922471
N	1.01134289621368	-4.45635919902855	2.68234321121670
O	2.40185750299715	-4.47450194818358	2.60502556051974
H	-0.56019340606297	-0.88625430514770	2.07685000226768
H	2.73500437679969	-3.58479035159240	4.48770324885452
C	2.58370124827361	0.71954036173666	2.54155946383547
C	0.53713912512418	-0.85171496941328	2.18612867697420
C	1.12858942400535	0.48007545765005	2.16389693389024
C	2.99160871568751	-4.50037960041463	3.91115651925368
C	2.70794651619513	2.04742210038798	3.28201413283105
C	0.33064559050015	-3.27244243039540	2.46339520576590
C	1.16567853328999	-2.04719052496844	2.36259034230749
H	2.68159610508084	-5.40035852513477	4.48506158580386
H	0.43125600586923	1.26796930607930	2.49958542069265
H	2.25618069177680	-2.13077117178493	2.46435484539524
O	-0.90742942055139	-3.25979572339099	2.40752948766879
H	4.08295296700996	-4.52962393266875	3.73716946505056
C	5.77900320200215	-3.29606364900608	1.99780721501123
H	6.28025058397014	-4.26617046199250	2.13865329665013
C	4.87398245383299	-3.11743267243844	0.93947550405139
C	6.04743971533576	-2.23082736117165	2.87412622121963
H	4.64894936309982	-3.94726424814740	0.25394861910806
H	6.76141603248464	-2.36048222048067	3.70195612319759
C	4.25353074905420	-1.87532853123703	0.74231932053576
C	5.41790934953021	-0.99158138907686	2.68471735962219
C	4.52524358735903	-0.80390458257891	1.61177047118266
H	3.56768625028138	-1.72496701151221	-0.10335045566427
H	5.63814405047327	-0.15229044693964	3.36415763817424
C	3.90622309244373	0.56057187008905	1.38809930601480
O	3.65205426151874	0.80356713741078	0.03622032675982
H	4.57480805846286	1.34216125648626	1.79652140057572
H	2.90737910981731	-0.06505398247535	3.25148596785353
F	2.37393914864130	3.10956307338336	2.50651741039851
F	3.96377985188735	2.26028860896835	3.74803580942170
F	1.88578276424252	2.07342494548769	4.36148655423104
B	4.51585607539054	1.90526678213108	-0.83976223692691
C	6.06758764150223	1.39950932266518	-0.80285432701743
C	3.77861172921139	1.73910794062318	-2.28427039274223
C	4.28716442991373	3.39313130577836	-0.18818459972156
H	3.24024156556148	3.45900364398392	0.18715547926984
H	4.31991811447108	4.12280879729170	-1.03025969410649
C	5.24723695043127	3.90357856049152	0.89672495734822
H	6.68984649980980	2.21847440298851	-1.23614233065960
C	6.39490927406334	0.08885142972275	-1.52799496348808
H	6.41738705384150	1.33208002763602	0.25541534855494
H	2.69212519980956	1.96485964027394	-2.16684815006844
H	3.80909890239496	0.66868851490331	-2.58059679937382
C	4.36361036011705	2.58551243267339	-3.41838393127447
H	6.29509715534867	3.92776353851924	0.52987972996633
H	5.25372605601749	3.26777614993312	1.80681608646666
H	5.00329120685067	4.93300130669155	1.24090734030622
H	5.81013066163053	-0.76118852851222	-1.11902453655718
H	7.46830610834916	-0.19426686519464	-1.45643398797745
H	6.15014766567729	0.14982900545243	-2.60998864035304
H	5.42316776295067	2.31614241455660	-3.61766195834919
H	4.36168962943264	3.66899456269076	-3.16947401712153
H	3.81386970785340	2.47494316392928	-4.37933750510553

(R,R)-N
Electronic energy: -5178.7322 Eh
Enthalpy: -5167.8030 Eh
Gibbs free energy: -5177.7000 Eh

P	0.70109982587512	1.08849664182299	-1.25992320963470
O	-0.55765153999792	0.92478911564500	-0.14772060242663
O	0.12637707541925	0.02567526597282	-2.43560724402525
N	0.50844164288604	2.51317482172146	-2.11582928893241
C	-2.22865560120297	-0.46118584576878	-1.08436183980884
C	-1.19241050149624	-1.52606112558777	-1.20136575841102
C	-1.87843591884951	0.78223431006463	-0.52717361876306
C	-0.04938830466387	-1.28192495744622	-1.98461046154051
C	-2.81283502593992	1.83680004460551	-0.28391943571498
C	0.88975112789913	-2.28971475208779	-2.34986136129418
C	-3.57637813707423	-0.68710965279396	-1.50452547271151
C	-1.36409941560373	-2.81513103942671	-0.60683651151694
C	-4.16284374286209	1.57021229894283	-0.70302504285993
C	0.69920486699786	-3.56911900808144	-1.72430648162155
C	-4.50386584503748	0.33383300905367	-1.30040721859900
C	-0.40257071360798	-3.79084597453965	-0.86727642333999
C	-2.50860978070583	3.13149974409044	0.32825294912152
C	2.00218295370046	-2.11719552586691	-3.28321512693310
C	-3.56471844132376	4.10645076181646	0.44452827611596
C	2.95034546499743	-3.19285250768333	-3.42658106415565
C	-4.01665809682459	-1.94646185883182	-2.15730875901059
C	-2.50281917857954	-3.14075980188868	0.28838747423625
C	-5.18007362890086	2.57384351963765	-0.55155416129585
C	1.66335568558140	-4.61577599994048	-1.91856004639418
C	-4.89112500513196	3.79740222410535	-0.01037244591320
C	2.76362456547776	-4.42326352836545	-2.70969396256506
C	-1.23698375010808	3.50006679078477	0.84873602385536
C	2.18444623582342	-0.97635307675525	-4.11452221404804
C	-3.30392016303381	5.37562435775549	1.02785389800443
C	4.05303382900151	-3.05422635614058	-4.31034795864875
C	-5.23357089908217	-2.54937569078344	-1.76912940749023
C	-3.16138266780444	-4.38355039151249	0.15743997351603
C	-3.26168503536256	-2.55494926450822	-3.18512592293869
C	-2.93839705515355	-2.25189871136588	1.29715412961413
C	-1.00764852517066	4.74640925300787	1.42781985802239
C	3.25544874409179	-0.88218187182822	-5.00153144320734
C	-2.04298273103714	5.70221104953152	1.51375714876508
C	4.21368423488549	-1.91443477240199	-5.08971172557066
C	-5.67614713280763	-3.73189944548498	-2.37648185156332
C	-4.23081411221144	-4.72426185817142	0.99574878423157
C	-3.70362141426983	-3.73879106745419	-3.79120681674938
C	-4.00919260568412	-2.59322501381409	2.13474103355159
C	-4.90965151119400	-4.33531593232210	-3.38649436524716
C	-4.66352058463739	-3.82747015554865	1.98553670084188
H	-5.53953042201041	0.19429035562990	-1.64447343461763
H	-0.48162186584834	-4.76866014545394	-0.36964615570274
H	-6.19912016601316	2.33323641041001	-0.89117096350508
H	1.50247566527558	-5.57149708016987	-1.39700065547737
H	-5.67328813747735	4.56511127767168	0.09568630974728
H	3.51235514649657	-5.22043509296633	-2.83623725826837
H	-0.42059802225950	2.77676122810511	0.82878752144244
H	1.46464532454312	-0.15433935479066	-4.06648038063273
H	-4.13332850097696	6.09706459353786	1.09516275843246
H	4.77347368162463	-3.88404822196154	-4.37907522687939
H	-5.81823122245452	-2.10149526122552	-0.95157311817467
H	-2.85127974031909	-5.07402406236681	-0.64106705577421
H	-2.32547117676683	-2.08741612715870	-3.52153124720838
H	-2.42423602959334	-1.28964094067778	1.43477704507356
H	-0.01213269046175	4.96646699829476	1.84217508051155
H	3.35196872088015	0.01306262843623	-5.63469507337437
H	-1.85699543317937	6.68673219056667	1.96917657876584
H	5.07029379700964	-1.82298097343920	-5.77415746495334
H	-6.61974095674739	-4.19380237673459	-2.04773852235922

H	-4.73916518096243	-5.69192794770705	0.86562798918240
H	-3.10247236490524	-4.19578745892902	-4.59221025725379
H	-4.33208898550203	-1.89140291429248	2.91885506489875
H	-5.25245804618296	-5.26767103964252	-3.86087215163136
H	-5.50674845203755	-4.09001094781070	2.64263385083337
C	-0.61660858106008	2.71818795478085	-3.05133224612805
C	1.19036234433826	3.71013224966569	-1.58797902344652
H	-1.42906551949684	2.00681829804198	-2.80584303945263
H	-1.02858344404642	3.73144640695301	-2.85543364412603
C	-0.23022223599524	2.55783368278152	-4.52055079336327
H	0.11758253542023	1.52562237602246	-4.72223773947342
H	-1.11021443476711	2.75269793772218	-5.16685585336521
H	0.57504250671036	3.25792893914581	-4.81600526300291
C	1.78661985766855	4.62930882524612	-2.64903186074627
H	0.47594609269163	4.28993604422089	-0.96088773751558
H	1.98508602142631	3.35619815224900	-0.89325971861663
H	2.51410692697145	4.10510607094402	-3.29955821564353
H	1.00313554497946	5.07347842707219	-3.29472637865823
H	2.31402136405855	5.46604971021039	-2.14893258616490
Ni	2.59143369245442	0.73684266000635	-0.38640955156296
H	-2.21774818889883	4.32280372693649	4.08373542083885
H	-0.81580028905202	4.10391315554547	5.20419111683641
C	-1.70463369566260	3.61332320429314	4.76894772834200
H	-2.39971692390665	3.31774735770737	5.58078244785955
N	-1.24113642215782	2.44894485491882	4.04716846786330
O	-2.25093648059882	1.76224060316958	3.36370532036705
H	2.35792716042350	1.67914588400152	2.51029694873342
H	-3.61829171877837	0.28406173276770	3.56209119587666
C	3.08827245270195	-0.97548304444696	1.70593133065173
C	1.56539916258525	1.00083232594278	2.15891514265845
C	1.91042978123542	-0.09314565820125	1.24141931672656
C	-2.77399998630111	0.69030908362173	4.15158649842467
C	3.08128429539220	-2.32581425941646	1.00249105122193
C	0.06350988869177	2.32962364728160	3.58647684208388
C	0.31943777329031	1.19964542381572	2.66346371609370
H	-2.01506302973883	-0.10646845750951	4.31401649595408
H	1.02288111085779	-0.71192269144888	1.00570185433803
H	-0.51293009089482	0.54690086775968	2.37157422100108
O	0.93900922236596	3.12177906645588	3.96684800001720
H	-3.15407398893019	1.04668118785933	5.13361515723105
C	5.40979336846951	1.95561946684615	5.16215019371337
H	5.64052375903251	2.50124190767147	6.08990493079885
C	4.97510871270941	2.65064378655391	4.02301159663291
C	5.55239668149244	0.55847391022996	5.11006851098153
H	4.85990845214641	3.74487290049680	4.05313185144891
H	5.90102521134640	0.00527410378990	5.99588614305100
C	4.68307812478824	1.95432080250345	2.83996155405360
C	5.25290171760038	-0.13511827886583	3.92838554163853
C	4.80794760495347	0.55597162976693	2.78473190900123
H	4.34830113516614	2.49254213342798	1.94196220467075
H	5.36613157813526	-1.23114937634138	3.88999221248537
C	4.43423768079786	-0.23788242761168	1.53833905362169
O	4.31778978188637	0.57841622593906	0.37653097988403
H	5.23376482401568	-0.99154195822768	1.37139097555786
H	2.93316976964128	-1.23715954953465	2.77630979437281
F	3.21167493459335	-2.22548318131171	-0.34243624923266
F	1.92224420659256	-2.99460945871674	1.24157741959476
F	4.09209692469098	-3.11781684287600	1.44230864356610
B	5.60928452842947	0.73772994533367	-0.63215810187067
C	5.76193078438896	-0.69233892757863	-1.41529911331961
C	5.07709281616211	1.95363884267579	-1.58220143493090
C	6.94486271614000	1.10342560726015	0.24319574983197
H	6.97389299760828	0.50039967435066	1.17973011166865
C	7.21365789003765	2.57577767673977	0.57732605452465
H	7.81374203661070	0.71966621436763	-0.34084995206785
H	4.78007982505250	-1.02564077931096	-1.81884183658471
H	6.05405216572205	-1.49569236168582	-0.69747032538417

C	6.77643486602046	-0.67175815561724	-2.56652405049201
C	3.92495657972037	1.54281956825834	-2.48751627301243
H	5.90705839236991	2.32448178192212	-2.22482284277287
H	4.78704812573134	2.82387600996807	-0.94948684242366
H	6.38104067153224	3.03551919306525	1.14789195292198
H	7.33907619014022	3.18426424852345	-0.34367496206577
H	8.13352657108156	2.72045075646984	1.18566977904567
H	4.27217748123487	0.91391876467837	-3.33147699975690
H	3.30590745446168	2.35657999706587	-2.90945339571356
H	3.18793066363183	0.78168394640689	-2.04478390795400
H	6.48658901176967	0.06125237939305	-3.35065098899307
H	6.88161013892620	-1.65531916444651	-3.07450721791524
H	7.79006058338266	-0.37544376147951	-2.22045358804902

(S,S)-N

Electronic energy: -5178.7349 Eh
Enthalpy: -5167.8081 Eh
Gibbs free energy: -5177.7029 Eh

P	0.94953803771493	1.01326569762706	-1.49541069573406
O	-0.23302527542116	1.26164018796721	-0.30107488677034
O	0.07870207306878	0.00750510983061	-2.52413176996874
N	1.03822662390018	2.34758826042610	-2.50311851947252
C	-2.22808653961966	0.21199318433715	-1.04279711649884
C	-1.47618791079126	-1.07494325392079	-1.08088587067264
C	-1.57473115251825	1.37695616272541	-0.59530226363276
C	-0.35518783813254	-1.17165117496455	-1.91932813565412
C	-2.24813087550445	2.61843422190623	-0.37093792929199
C	0.31616638700137	-2.39273927214261	-2.20447965973111
C	-3.60768719975294	0.27684294684994	-1.40987742237531
C	-1.88829682856321	-2.22970032373490	-0.34268421527694
C	-3.63494644099521	2.65107875570793	-0.75191058493363
C	-0.03913495423211	-3.50153198543838	-1.36566653624393
C	-4.26897009396593	1.49580927843777	-1.26488229258683
C	-1.11887176913007	-3.38870221227573	-0.45977872747773
C	-1.64268971419800	3.82984780456797	0.18687843757176
C	1.28341642812890	-2.59221002966483	-3.27914581009551
C	-2.43341658923683	5.03430118904792	0.24437278270764
C	1.99573426210307	-3.84111612408415	-3.33293947030366
C	-4.34854553363279	-0.89437087396111	-1.94354005567443
C	-3.08224036863296	-2.23886256336396	0.53948263898215
C	-4.38220894266916	3.87476292555053	-0.65563823346505
C	0.71818671998552	-4.72029953438806	-1.44451378955788
C	-3.80073477999582	5.02532652511733	-0.19511342790755
C	1.72212453174738	-4.86898700510852	-2.36590805050963
C	-0.31765686362172	3.90700082817089	0.70157712533974
C	1.50918496933253	-1.65747793118261	-4.32926710970449
C	-1.86824966071628	6.23403575396137	0.75403934614802
C	2.93398608505429	-4.07244145371268	-4.37377371209474
C	-3.79627357865834	-1.73266530179650	-2.93778551237136
C	-3.39727497133232	-1.16206228661634	1.39612676684629
C	-5.64771365943284	-1.17806134875333	-1.46802367325793
C	-3.93552777141663	-3.36577591446644	0.53712847948096
C	0.21274142863464	5.09405638154290	1.20327167882129
C	2.41853640257145	-1.91990300119387	-5.35118685972608
C	-0.56010376990915	6.27501776316518	1.22284200015035
C	3.15234396317639	-3.12678810165856	-5.36973495883759
C	-4.51654935520348	-2.83102533990397	-3.42672026997672
C	-4.53677821309933	-1.20435727181853	2.20997665699884
C	-6.36747266706144	-2.27601286789531	-1.95725945596502
C	-5.07605999179250	-3.40807676810443	1.34962962574805
C	-5.80244933545209	-3.11088076974381	-2.93474432720090
C	-5.38621452589493	-2.32273920960797	2.18550418746456
H	-5.31970043874215	1.57841483750199	-1.58051324889043
H	-1.34908112928900	-4.25219737631574	0.18014016610180
H	-5.43587383508361	3.86309327496902	-0.97384214175741
H	0.47139801859541	-5.53015952823671	-0.74145529763926
H	-4.37502554784598	5.96287911583796	-0.13734430908115

H	2.30876409707231	-5.79960990176569	-2.41259623244874
H	0.30139049317177	3.00865533237804	0.71210266010482
H	0.95436658943413	-0.71389308798321	-4.34028572799463
H	-2.49837535277701	7.13708897537159	0.77651924163550
H	3.47881125140108	-5.02894532413095	-4.38258035738179
H	-2.79744096622983	-1.51312831576821	-3.34069426144695
H	-2.72838866907408	-0.29436573059281	1.45516103155961
H	-6.07806546750948	-0.55071722036875	-0.67314107865939
H	-3.71785185255995	-4.20314975354118	-0.14261001658955
H	1.24412311202974	5.09788780670301	1.58596573319553
H	2.56493634503234	-1.17517674680497	-6.14852502416706
H	-0.13665706165529	7.21233610244105	1.61440069845256
H	3.88034880117225	-3.32206129311527	-6.17149348655976
H	-4.07031463579711	-3.47100557132522	-4.20342806287119
H	-4.75113794092929	-0.35994600649950	2.88236125368273
H	-7.37162342760087	-2.49016460513654	-1.56037998124714
H	-5.73483019313913	-4.28952940009151	1.31919837173420
H	-6.36392180072610	-3.97793025456062	-3.31543004296096
H	-6.28451496324341	-2.35122915224050	2.82135948926800
C	0.11506499119209	2.62674992852180	-3.61938885227079
C	1.92238872625314	3.43982068484474	-2.05944296898650
H	0.73026884057258	2.76843788285647	-4.53498878397945
H	-0.48971196115637	1.71849040583247	-3.79164284562796
C	-0.79894298383983	3.83407717508189	-3.41566456145054
H	-1.44381877052185	3.95692137633107	-4.30904660103398
H	-1.45590103469387	3.70243677926079	-2.53551899267600
H	-0.23227971819589	4.77731777794759	-3.28350683600483
C	2.76724516480806	4.04892277612108	-3.17350940749389
H	1.31744812737003	4.23042985499657	-1.56098671364266
H	2.58323709773828	3.02442787054557	-1.27273290925453
H	3.47311684747772	4.78241809739639	-2.73579808081748
H	3.35807676935859	3.27698284262180	-3.70337799766101
H	2.15065148887904	4.58856020922803	-3.91996127426422
Ni	2.67840718071755	0.17630948492315	-0.59370546293251
H	-1.18964501631344	-3.93331244828903	3.56828842806545
H	-2.80384831628833	-3.11036472722815	3.51024776320198
C	-1.77831347254192	-3.07324581597874	3.93544767193426
H	-1.83923614810676	-3.12113796089887	5.04312120731054
N	-1.08955575590490	-1.88025998007347	3.50514797360131
O	-1.68271460302516	-0.67541917339743	3.88161058072724
H	2.31809011999880	-1.31197998303935	1.70118536759664
H	-1.23022493529373	-0.91974120238125	5.93015819923745
C	3.68660763612688	1.18046658722302	1.75647171222611
C	1.69738063105398	-0.40374722979092	1.68752453742985
C	2.29986244951972	0.83791253367194	1.18910507970525
C	-1.11617117373273	-0.18558540018534	5.10316608180961
C	4.17706471876665	2.55254505071214	1.32021745062525
C	-0.01974223575126	-1.87128697552960	2.62847655186987
C	0.43335920851599	-0.53513995162957	2.17421703059509
H	-0.04121303610113	0.06842696883617	4.97589673240438
H	1.61721535082597	1.70058097493395	1.27527000573746
H	-0.24867061245260	0.32291613546503	2.25106442243417
O	0.52232390278531	-2.93459567904841	2.28568670605353
H	-1.68780045372065	0.73052504165081	5.34656647954025
C	5.55836315240027	-2.51948538842672	4.76609578364337
H	5.77008464286572	-3.16556369779534	5.63195692225054
C	4.80880553306767	-3.00787269938808	3.68497475196571
C	6.04188505104495	-1.20006907992374	4.73377885551229
H	4.42707997410284	-4.04034768354079	3.69692682027402
H	6.63688054264861	-0.80816822375423	5.57328320951043
C	4.53922572034067	-2.18551423833609	2.57883109992167
C	5.77248243236223	-0.38057001342407	3.62824262361989
C	5.01254358944113	-0.86297494452966	2.54318644903545
H	3.96348644016082	-2.57783330291403	1.72977611780542
H	6.15525968504397	0.65273292443650	3.60465573580857
C	4.72357768645081	0.08459260787752	1.38516042239638
O	4.20517429909622	-0.56950180626395	0.23459330703018

H	5.68645372271618	0.57305565046215	1.12371028442216
H	3.59907920504634	1.26968313536637	2.86260719099001
F	5.29192068930315	2.91082193519225	2.00388073406647
F	3.24675912781739	3.51953056946787	1.53886603210858
F	4.49907873888341	2.61453114351064	-0.00361899561684
B	5.02447649761727	-1.12470161973264	-1.03915311034746
C	6.48426885488503	-0.40519253451857	-1.06115627105155
C	4.97153388020088	-2.75061135284015	-0.86753047599245
C	4.02152570310023	-0.64695434356687	-2.27690664800667
H	7.03224579870473	-0.64681732385642	-0.11972555954186
H	6.33886662112938	0.70066232989458	-1.03614715751723
C	7.40378057997527	-0.74886046785599	-2.24348115672035
C	4.25365806180737	0.68328494768625	-2.99741568506294
H	2.88848971516840	-0.79152761510521	-2.05470794908149
H	4.03078467560400	-1.46513618644565	-3.02901147231625
H	5.46622323916851	-3.04066627101251	0.08639873645890
C	5.56482793900908	-3.59821049832201	-2.00099023727718
H	3.89833055357241	-3.03279553000879	-0.73586785546168
H	7.75852528345667	-1.79752587535446	-2.19748248948538
H	8.30905720834250	-0.10478273790316	-2.28389377657648
H	6.88763470302633	-0.63867666376439	-3.22096205837337
H	4.35847949207674	1.53925150591763	-2.29979175839332
H	3.44118721821875	0.92845520775196	-3.71308880045198
H	5.20039269299542	0.64010990903163	-3.57358093298820
H	6.67360386017085	-3.57319828152611	-2.00046479021303
H	5.24073001266754	-3.24489583809579	-3.00314494970519
H	5.26944208584652	-4.66853632848478	-1.93531751397696

(R,R)-M

Electronic energy: -6815.5734 Eh
Enthalpy: -6800.5742 Eh
Gibbs free energy: -6814.2236 Eh

P	0.78578794066878	0.36333680117574	-1.17738063277903
O	1.40884278150687	-1.18527003796841	-0.82376079704110
O	2.24120794712968	1.00798333535881	-1.82914632166687
N	-0.09345600388543	0.15755010616813	-2.58636575957651
C	3.79248927875327	-1.08808707071131	-0.94257060169889
C	3.80115032453403	0.21797096857774	-0.22447913750451
C	2.58679819434399	-1.75338756870480	-1.24888299201187
C	3.14646734083472	1.30634172553333	-0.82970593148140
C	2.54731773448311	-3.05893276723590	-1.84911472715475
C	3.40314933796472	2.66320034080576	-0.47920400179657
C	5.04148020658150	-1.67877363067992	-1.32133973710660
C	4.57979628010069	0.44529700145950	0.95681560116824
C	3.82536021073778	-3.66727017446197	-2.10426734383771
C	4.16307703205615	2.86383320786935	0.72099338966183
C	5.02548933764933	-2.95803273971337	-1.86809037121480
C	4.69513243639602	1.75648509690760	1.42175828009096
C	1.33778575696536	-3.82233681487099	-2.17486713001518
C	2.96971277344791	3.82283319682944	-1.25399802118494
C	1.48250971522730	-5.17749763466914	-2.65175033198705
C	3.19674072534325	5.13592717979244	-0.70815877003862
C	6.34546555409947	-0.98242585149318	-1.16263958039362
C	5.24169090080824	-0.65156366529956	1.70583031913929
C	3.91165241442602	-5.00863680332081	-2.61038244255016
C	4.33901815934962	4.19334197392526	1.23621942621308
C	2.78462289782486	-5.74471734539827	-2.85334401653223
C	3.85170003268241	5.28321404104595	0.56306184369421
C	0.00862496688219	-3.33198278818631	-2.04648766889007
C	2.39230587759888	3.74128177795342	-2.55351602708428
C	0.33643560607278	-5.96742016318034	-2.93478151320945
C	2.80867529551910	6.28535511056238	-1.44888642274993
C	6.51912184433183	0.36234852874532	-1.56046362070551
C	6.49927812402160	-0.43952914279494	2.31493615186382
C	7.45860231768447	-1.67690655571890	-0.63978263364397
C	4.63955845199505	-1.92087658838139	1.85097395729046
C	-1.09759525909865	-4.12534222915672	-2.34433831191803

C	2.04647252321814	4.88531467933245	-3.26807277665710
C	-0.94578430172482	-5.45654695840910	-2.78198417186515
C	2.24394166972191	6.16938062928923	-2.71389071078924
C	7.76157212369746	0.99524886978503	-1.42082923727852
C	7.13068271096468	-1.45859067476699	3.04016155840987
C	8.70195760165412	-1.04507753349587	-0.50243449302022
C	5.26832105234973	-2.94161016016445	2.57543078088904
C	8.85731700779792	0.29659787039889	-0.88622543205173
C	6.52006137912353	-2.71652639662191	3.17208589394260
H	5.97464366287957	-3.43397310009506	-2.15607378469521
H	5.21603570941039	1.94889785967524	2.37104440704733
H	4.91301434111423	-5.43175424768314	-2.78337499513329
H	4.87520931878297	4.31397387996388	2.18988632458339
H	2.85419721868286	-6.78029887411407	-3.22095004996691
H	3.98606712905956	6.29850397160046	0.96737322369311
H	-0.16242765126671	-2.31750730978536	-1.68187244529449
H	2.22647456747576	2.75703757397517	-3.00208666780774
H	0.49131646545655	-7.00057586927855	-3.28326297707640
H	2.98533316144508	7.27747808502001	-1.00414157397633
H	5.67290859104129	0.91421879069871	-1.99386279055592
H	7.00644919388163	0.52784161138128	2.18345818318034
H	7.33369521938191	-2.71692016435131	-0.30253607403206
H	3.65604249889902	-2.10101123672697	1.39899903291816
H	-2.10626775045608	-3.71215269217835	-2.21075107993765
H	1.60276835204919	4.78332326868589	-4.27002286809934
H	-1.82860614200272	-6.07747298822532	-2.99658742927489
H	1.94883923349894	7.06522804789335	-3.27917662865475
H	7.87585542560579	2.04328391994330	-1.73796286466193
H	8.11637638030628	-1.27295825899827	3.49380097208313
H	9.55197053651766	-1.60095857132136	-0.07765365532935
H	4.77070106769424	-3.91834505042174	2.68090431262950
H	9.83118630266863	0.79714747297191	-0.77217608583205
H	7.01738447178110	-3.51860365007205	3.73877689477699
C	-1.34690281998780	0.92217361787147	-2.70223602972626
C	0.52463018923038	-0.37021425231662	-3.81428549439199
H	-2.15566358317261	0.23593861508247	-3.02879328643847
H	-1.62177816681647	1.24323337876751	-1.67395915441821
C	-1.26447793933256	2.13110353859630	-3.62744786221292
H	-0.45964939128443	2.82201053610759	-3.30524474853750
H	-1.07305687935798	1.83646229029930	-4.67926331416422
H	-2.22062448217515	2.68735332731958	-3.58935348573247
C	-0.44713643294491	-1.13642718939588	-4.70835948033525
H	0.99218482040512	0.46136090909869	-4.38974961029716
H	1.35456590698865	-1.03968325065393	-3.51240896624179
H	-1.24565418714939	-0.48612943768319	-5.11722947577314
H	0.10823153668113	-1.56176615990259	-5.56794907626084
H	-0.92305616250800	-1.97346132190679	-4.16314507795228
H	-2.97113417593871	4.68207587238032	6.04362721417412
H	-2.34906594662871	2.24042870316543	6.10043977822697
H	-1.52960916929319	-2.51451892436596	5.77254049449476
H	-1.94175716767868	-0.92768785872422	5.05307614357262
C	-3.13577880442067	4.04647962302396	5.16027744836497
C	-2.78603646430061	2.67861852485036	5.19033201614084
H	-0.31293710509142	-1.19573279499944	5.75234428033447
C	-1.12933471582159	-1.67040078296931	5.17448650105867
C	-3.70179920006160	4.57910482368126	4.00718965371896
C	-2.97542870261160	1.87283685816917	4.07080512992896
H	-4.00217415071791	5.63799425150868	3.96926271809087
H	-2.67704432953106	0.82165468988723	4.11234969025144
H	3.17096748627504	-0.56246506635139	4.15377745230253
H	1.85668219813252	-2.01211193691929	2.62161511741485
C	-0.64536371216531	-2.17723146804147	3.81603229763213
C	-3.91848501223545	3.77687682328622	2.85517447534000
H	-1.49660639126947	-2.63295863523896	3.27031693819257
N	-0.03011162057395	-1.14011685334571	2.97204114496775
C	2.07639437931546	-0.66555425822077	4.29713947354551
C	-3.52088270206116	2.39212921964745	2.86307908766274

H	-8.18738011081193	-0.02235093197104	1.77950485283170
C	1.43780439973964	-1.03975821089447	2.96438068783753
H	1.68160376800221	0.29188854210643	4.68278972149559
H	0.10121283115689	-2.99115055185559	3.94423146121165
O	-2.36822525214016	-0.30506238923960	2.35164285916433
H	-5.84260880747936	-0.83940129326310	1.94591874483941
C	-7.85283532433370	-0.85015167844360	1.13547465998105
C	-4.54255581674010	4.34143216561858	1.69077344598951
C	-6.53075283718610	-1.30593690677354	1.22655743163213
H	-4.81546475705603	5.40796480323787	1.70930863485151
H	1.91771338937848	-1.45187630296763	5.06251225458397
C	-3.72464864426790	1.60008302663980	1.65184811154865
H	-9.78531598973657	-1.08526544584215	0.15925766843240
C	-3.26387423528714	0.26342785111225	1.45500210602744
C	-8.74858145577191	-1.44815973768500	0.23305136049048
H	1.70816432556384	-0.29088605948054	2.18758723828913
P	-0.81403884524894	-0.45229861171169	1.66289638711432
C	-4.81343860268553	3.57076608353486	0.59158629042904
C	-4.43795077247348	2.18423095919133	0.54989588129050
C	-6.07651987408410	-2.36800526466326	0.41278557348203
C	-8.31307390190682	-2.51393762247573	-0.57075840621643
C	-3.70438940092937	-0.54885020714316	0.39094236166749
C	-4.68429334053016	-2.88096566021896	0.50273743112781
C	-3.53767054874188	-2.02571469077517	0.51607195025911
O	-1.19285632659953	-1.72447402737205	0.57600823622783
C	-4.47886184089302	-4.25655925870535	0.59348025146518
C	-2.25478972260213	-2.58772079953302	0.66884545484863
C	-6.99030298677334	-2.96831101059131	-0.48111500351229
H	-5.29297451268473	4.00249763553078	-0.29776507732708
H	-5.34902959238060	-4.92992886157892	0.59894904791385
C	-3.19217053484500	-4.82812928774178	0.73347896548154
C	-2.02062709281227	-3.99705417686355	0.79366854025409
H	-9.00434360000553	-2.98594302179703	-1.28586279208231
C	-4.72306752661389	1.41684684761359	-0.60301768150288
Ni	-0.04131224602397	1.20893726330650	0.59733509287349
C	-3.07623706116002	-6.25700297307586	0.82955653649251
C	-4.42149560826245	0.05759089584118	-0.68922653917533
C	-0.71337833496062	-4.63899845020781	0.95550972923585
H	-3.99876095116186	-6.85549935456038	0.77768704138894
C	0.52825972280448	-3.94518556398744	0.99999765329141
C	-1.85654409460611	-6.85871170734755	0.98330251083870
H	0.53919586035048	-2.86206965283116	0.87389812916341
C	-0.65472445012687	-6.07704469696120	1.05150727107993
H	-6.64398778796976	-3.77881601395919	-1.14041548418393
C	1.74099178789776	-4.61506537624213	1.14690305869281
H	-1.77355073078249	-7.95430953342252	1.05605088807368
C	0.59560073151883	-6.73565662599013	1.20019175387486
H	-5.19473893225226	1.92385732288781	-1.45681120606997
C	1.78613162116829	-6.02100485737821	1.25107801195903
H	2.67565267747622	-4.03587348528390	1.15071449158804
H	0.59892578489788	-7.83496525933553	1.26794352917437
H	2.74959028686259	-6.54216488472216	1.35762231911696
H	-2.95782863428519	-1.81391953108130	-2.04058659437622
C	-4.81499399686057	-0.69868798268793	-1.90269063944010
C	-3.94393691288805	-1.64078828514468	-2.49271781117687
H	-6.76721205220581	0.24488601048596	-2.05544549637408
C	-6.06451648880214	-0.46416617783657	-2.51760234864295
C	-4.31297742752905	-2.33399867883150	-3.65334566269259
C	-6.43564998978631	-1.15701682559091	-3.67779566934406
H	-3.61466781014774	-3.05867420570669	-4.10039234537083
C	-5.56354342847688	-2.09816180211690	-4.24883425791759
H	-7.42077287522969	-0.96975409799558	-4.13197664351239
H	-5.85594033746842	-2.64531451980237	-5.15820710553947
H	-3.92624354632098	5.96233846059417	-3.56200531494894
H	-2.68984956830920	6.64947392919273	-4.69526285427238
C	-3.07065263519870	6.65585810607811	-3.65127040242428
H	-3.40675616233466	7.68401328154325	-3.39809535222434

H	-1.34351710939580	2.77492008791535	2.21763084318153
N	-2.05721619637313	6.19347782209656	-2.73417443826955
O	-0.86142919420874	6.90879940279538	-2.76309957741522
H	-2.04259572161559	3.26974459778259	-0.07547534420150
H	0.11807432412754	8.45446632256268	-1.88095352019253
C	-0.30446796737144	2.67333690046225	1.86961695996057
C	-1.03816503575020	3.68968019551484	-0.26327313430485
C	0.03958583789349	3.21895394385759	0.58945433454144
C	-0.84241482560075	7.92068510697837	-1.74904257461475
C	0.65016123343758	2.75364258899979	3.00999961548044
C	-2.14498225295802	5.01946897495865	-2.00369988649364
C	-0.94247935517076	4.63975144832267	-1.23480882638269
H	-1.68198672743298	8.63919373115695	-1.87202410454807
H	1.04701323108883	3.62727836688758	0.41254102600118
H	0.01198819548173	5.13821818292520	-1.45084166695783
O	-3.20394201017916	4.36385164815616	-2.01623125515761
H	-0.87880080660440	7.47247120462586	-0.73238516696175
F	0.78371765858004	4.02250313739478	3.49275918691987
F	1.91168829810449	2.33791430116620	2.69030670728074
F	0.23743149025613	1.99198092553583	4.06406220210512

(S,R)-M

Electronic energy: -6815.5686 Eh
Enthalpy: -6800.5649 Eh
Gibbs free energy: -6814.2190 Eh

H	-4.95864948989534	-2.36758433474457	-6.02597027698390
H	-5.48681528581628	-0.67119916334252	-4.24666338825591
H	-6.13431292894389	2.24187873047219	0.05683194045744
H	-5.25100599217930	1.37795948438340	-1.24712065237788
C	-4.44602902470562	-2.40809261711149	-5.05302063412910
C	-4.73080225840976	-1.44865315146384	-4.05659676881877
H	-5.22685855608052	3.16673691706145	-1.18066148038841
C	-5.21354654404992	2.24780092938908	-0.56122061771209
C	-3.53162262780905	-3.42015307821278	-4.78071145259329
C	-4.06180952985228	-1.47319607344404	-2.83360989985501
H	-3.32218262510557	-4.19970956537103	-5.52994174964801
H	-4.28896380900388	-0.71177802982463	-2.07873744457822
H	-1.31454944129670	4.38309432501140	0.76584021710310
H	-1.39136103170207	3.35934912196991	-1.56514388759016
C	-3.97886656808388	2.17507120764954	0.33728356784295
C	-2.87499959741832	-3.49927117096498	-3.52419010869469
H	-3.99515057520131	1.23455605864396	0.91782491732507
N	-2.71505863790564	2.23885566633114	-0.41130168629744
C	-2.10666217213756	4.64600476390669	0.03989813159831
C	-3.09615894447892	-2.47564279876505	-2.53744424223844
H	-5.74072752002285	-4.66076475354307	1.42177278809326
C	-2.32379658402459	3.53593262074282	-0.98535870614005
H	-1.80722734497030	5.57767112563686	-0.47994079587023
H	-3.99162275383320	2.99817432971266	1.08330782763043
O	-2.83857886348603	-0.28773379179263	-0.52251859326158
H	-4.58704707813694	-2.46504074638936	1.21310202497465
C	-5.22792785579149	-4.23010103741783	2.29559769035313
C	-2.03526535670375	-4.62287416353537	-3.21428421524872
C	-4.57403379139127	-2.99636278717029	2.17530033478654
H	-1.85782854792287	-5.37814427374858	-3.99541753099684
H	-3.03332261244574	4.87161445383151	0.60556188031033
C	-2.39973394593846	-2.57308853163123	-1.25991522352603
H	-5.74319776769079	-5.88458163515449	3.61435295772743
C	-2.38185972178660	-1.56770622158421	-0.25071583459139
C	-5.23467254802366	-4.91236543906311	3.52396417034346
H	-3.07546312621630	3.85288768072830	-1.73547585618456
P	-1.66327907502552	0.94845372690176	-0.60212854052555
C	-1.51717580849459	-4.78596617118544	-1.95656859647253
C	-1.71071739647180	-3.79168663816676	-0.93722125769645
C	-3.91102961367998	-2.42166263326253	3.28266118806399
C	-4.59137543913633	-4.34446993989057	4.63540515117889
C	-1.99107721947736	-1.83186858707571	1.07148434466298

C	-3.20171172627331	-1.12062718801350	3.18257979503183
C	-2.31177159618318	-0.81664468564185	2.10968035822401
O	-0.91225960011236	0.66898558812311	0.92094458427999
C	-3.39153549583127	-0.15035224091176	4.16478561399589
C	-1.68293753605338	0.43895418350073	2.04271309381163
C	-3.93699042527683	-3.11095693990805	4.51514962657594
H	-0.92732722810454	-5.67881943708258	-1.70415908010977
H	-4.10718431330612	-0.33774098774354	4.97937297609789
C	-2.69031556521224	1.07815479795534	4.15918285518434
C	-1.77125361570425	1.40114102241815	3.09863365633132
H	-4.58505801946220	-4.87305472159006	5.60103011465533
C	-1.21442550429473	-4.01008888227942	0.36868325050121
Ni	-0.37540394796272	0.79923303553434	-2.31552969168057
C	-2.91583954124464	2.00866763174779	5.23081894032215
C	-1.39078422717020	-3.08565498596360	1.40070145565879
C	-0.98671837295760	2.63103264400946	3.20557035759915
H	-3.65390507971983	1.73891903331240	6.00170466031100
C	0.06354134108888	2.98681027013295	2.31293651333983
C	-2.22708320475299	3.18913096554196	5.30009027965903
H	0.27981596709680	2.33878694997502	1.45764002330944
C	-1.23016687423913	3.51405652770991	4.31936178470656
H	-3.40149427503014	-2.68751819432983	5.37826529378919
C	0.83004677450144	4.13470800230180	2.51096052358396
H	-2.40004764502118	3.89610811979252	6.12634061227924
C	-0.45590700078973	4.69471317912903	4.47653386670757
H	-0.66907123501127	-4.94313124839382	0.56665648855750
C	0.56893962760753	5.00461078811422	3.59076251895391
H	1.64139568008584	4.36510650495050	1.80795842277404
H	-0.67491883041884	5.35078294773834	5.33360857918649
H	1.17394441297278	5.91335881793215	3.72979656115440
H	-1.61395888766561	-5.48741145118613	2.63158086154358
C	-0.95543240658594	-3.42057441888312	2.77983868139161
C	-1.12650639050421	-4.73497266977446	3.26936525631416
H	-0.16795060592590	-1.44872254324624	3.26739639465168
C	-0.35876772152663	-2.46493778054964	3.63322389238622
C	-0.72706643709934	-5.07990349960586	4.56789012360627
C	0.03153079624285	-2.80438233011786	4.93525521268283
H	-0.88218202932937	-6.10778305303975	4.93058616426686
C	-0.15256781247479	-4.11337060576014	5.41035685532956
H	0.50339929931921	-2.04089473210789	5.57244339649965
H	0.15317228526065	-4.38036856850806	6.43376861284897
H	-6.23804904148990	1.74345475668909	-4.08324468639281
H	-7.14163172580279	2.85392702123844	-5.17089901351127
C	-6.67151618078191	2.75399625791369	-4.16853960219457
H	-7.45826962080076	2.86358292007167	-3.39331726271420
H	0.15541558596802	1.42359406260826	-4.81012648273281
N	-5.64690951354539	3.75761979689706	-3.98407479437543
O	-6.15535763495295	5.04270115926051	-4.16773212407331
H	-1.73941640858525	2.97084908343882	-4.19049170494419
H	-6.74557057489524	5.26116270282365	-2.15405050641421
C	-0.34493876675871	0.60471396420986	-4.26517023546467
C	-2.36211491681160	2.08076406728850	-3.98684322937080
C	-1.69613525352025	0.80262424998024	-3.82915673189888
C	-6.14594773567411	5.77453380263143	-2.93848659085784
C	0.06241636474527	-0.73394850654438	-4.77734094011084
C	-4.26198083619923	3.64118294553247	-4.03159783614660
C	-3.70807364550895	2.27602073592589	-3.90404634562436
H	-5.10773191569881	5.93697935121056	-2.58069066594442
H	-2.34998824774244	-0.07939446039955	-3.72425464424491
H	-4.36516024325155	1.41639528202633	-3.71857971251211
O	-3.54991852754096	4.65587003279309	-4.09595187210275
H	-6.61181163617144	6.75054813045918	-3.17490800744945
C	1.98406713883686	-5.67879480956529	0.12205389712740
H	1.88589181392433	-6.77328502487389	0.19407667313126
C	1.96407823760279	-5.07372539221923	-1.17873356051822
C	1.83996901875641	-5.90079463316127	-2.32707238615769
H	1.77021635541327	-6.98986884487858	-2.17704469138080

C	1.80572268171996	-5.36062495836250	-3.60718710802821
H	1.70940559802947	-6.01425081172570	-4.48757939697135
C	1.89285520437430	-3.96147831327608	-3.75958271168096
H	1.85617723531718	-3.50714039010852	-4.76038486236851
C	2.02019096863214	-3.12953085477468	-2.64908484910289
H	2.08665633755119	-2.05460267263534	-2.81143461825715
C	2.06474470365492	-3.64046003279410	-1.32231115871406
C	2.24273053648671	-2.82053273839790	-0.11947545091356
C	2.27802626966403	-3.48673173412956	1.15573527952128
C	2.44880036373382	-2.75361977096955	2.35057289898523
H	2.35578010780080	-3.28891465795822	3.30515299766173
C	2.70616912897437	-1.38782815050014	2.34871401499530
C	2.83562723185806	-0.71946801719702	1.09050814569881
C	3.53955987771495	0.59569354048407	1.03174848057334
C	4.84504139497638	0.74717963277571	1.59798121085465
C	5.60421832733349	-0.37921871769167	2.20165411504499
C	6.24996314706308	-0.20904586895614	3.44571570610824
C	6.99617101705225	-1.24973236143401	4.01631901349388
H	7.48060174981744	-1.10136360283441	4.99369923640611
C	7.11132475137932	-2.48099324286745	3.35132485185578
H	7.69246803957257	-3.30119687010237	3.80019721682912
C	6.48303363884875	-2.65883130516061	2.10683204661587
H	6.57534617230730	-3.61678563781330	1.57238197565876
H	6.13423156286478	0.74377808077166	3.98415989805548
C	5.73761328159954	-1.61843317416048	1.53633914102677
H	5.25800802131395	-1.76445853551300	0.55741504495051
C	5.45107760887909	2.00243292546508	1.52585861224801
H	6.46719548573621	2.12967011618586	1.92855147832583
C	4.84187526641442	3.11401402392133	0.89688706657504
C	3.54077291738121	2.98864908984126	0.29970539320739
C	2.94553480444558	4.15222585266208	-0.36221275082109
C	1.63581825343221	4.18915021513729	-0.92534581637784
H	0.99106090104920	3.30953732683844	-0.85611740572627
C	1.14390013108920	5.32463155181009	-1.56498288246936
H	0.13231702964829	5.30062585958030	-1.99752344681773
C	1.92599872178252	6.49479890581618	-1.66679938992905
C	3.19512909498935	6.50859774759420	-1.10032821193699
H	3.81878128593564	7.41510200211024	-1.14808097581783
H	1.53204549245281	7.38597467992704	-2.17832890354066
C	3.72197140907473	5.36516024828936	-0.44128582459382
C	5.02516040451676	5.43472408253313	0.15980765234664
H	5.58324984488342	6.38097384565766	0.08644008347655
C	5.55705122656212	4.35722520908223	0.81609624554611
H	6.55177023212448	4.41539169456510	1.28386780573199
C	2.94613949526216	1.69542945422589	0.39333178698228
O	1.71390021470892	1.45980805538866	-0.16647581215664
P	1.65618052453665	0.68622328221997	-1.68880541565298
O	2.53103605326538	-0.71446784455243	-1.28518755256351
N	2.87023285556256	1.32832259123191	-2.66531690339821
C	2.50075708111301	2.43757567691119	-3.55169036795451
H	2.96770109374071	3.37448754539999	-3.17341294695903
C	2.85977357276222	2.23874757597490	-5.02258301170034
H	2.44375657773842	1.29228728332085	-5.41818540877265
H	3.95717870657897	2.22165693076213	-5.17754171758563
H	2.45636058300148	3.08149842947696	-5.61988731595210
H	1.40601051661693	2.58933223469090	-3.44033295439272
C	4.30673753046487	1.01911208307151	-2.53879924441074
C	4.79202646447984	-0.11100144877021	-3.44815963652347
H	4.27848063641007	-1.05983599465213	-3.20030904026746
H	5.88195254319902	-0.26396675867652	-3.30745786640572
H	4.61221148921882	0.10733767209361	-4.51830973476427
H	4.85970706123876	1.95999665287227	-2.75103178092816
H	4.53623332591532	0.76443394241125	-1.48696596290338
C	2.77019855673436	-0.65236903003113	3.63343337089675
C	2.10913584328649	0.58877241875087	3.76295726584077
H	1.59101476209232	1.01537479260793	2.89439314749549
C	2.07475025528509	1.26404435856080	4.98977690167060

H	1.54138997421020	2.22471000278067	5.06242574864857
C	2.70764244526945	0.70777395311701	6.11443185110469
C	3.36865710151405	-0.52766109488481	5.99956696240429
H	3.87275637457789	-0.96636640777799	6.87461376210184
H	2.68500626271854	1.23521871136981	7.08051704021568
C	3.39779388562862	-1.20349956344761	4.77162202330631
H	3.93526015530038	-2.15928017576404	4.68253428219969
C	2.47653573134468	-1.40268225929035	-0.09257571413972
C	2.12046714712789	-4.91060238332422	1.24631622826845
H	2.12571345506319	-5.36767954015140	2.24737016482062
F	1.42554291725961	-0.87707710330829	-4.81009106077680
F	-0.36023335792778	-0.96293143612927	-6.05331201446647
F	-0.42570734053717	-1.75540199642001	-4.02333667361853

[Ni(benzaldehyde)(stilbene)(substrate)]
Electronic energy: -3526.3961 Eh
Enthalpy: -3519.7804 Eh
Gibbs free energy: -3525.7584 Eh

Ni	0.21977025196060	-0.12052034983824	-0.04339671065190
H	-6.96334459922655	3.34141479304084	-1.41264125797637
H	-6.64094432467324	1.58640723982926	-1.68718248699484
C	-6.21882124334133	2.60329111429343	-1.77579734903406
H	-5.98762461121143	2.81745421135939	-2.84180022435371
H	-0.97484876129999	-1.26825889703472	1.95171505245129
N	-5.02000094902331	2.64401763511755	-0.96937880126301
O	-4.32868871442097	3.85712975695383	-1.01166284365175
H	-2.76957103747285	-0.33421481384788	0.50137543356138
H	-4.50929418543434	4.29474702841589	1.04346391992064
C	-0.39897324436019	-0.33449881002524	1.83121619246221
C	-2.33087905833739	0.67440952729375	0.60083727346812
C	-1.03405903127052	0.79201012459433	1.24433092646952
C	-4.75459816911077	4.72312648878140	0.04702394763103
C	0.61695594391196	-0.13307481108241	2.91118217896170
C	-4.36342961462016	1.50828209878350	-0.51996116083179
C	-3.04416792337552	1.72868898055766	0.11144687082825
H	-4.19492359124567	5.66646381211758	-0.10104804700441
H	-2.64690627327651	2.75186111871623	0.17275472870352
O	-4.90032402351692	0.39329057846569	-0.63322084659351
H	-5.84482713466710	4.93381287908435	-0.00732536662616
F	0.03929597662278	0.22268892182376	4.09257500856862
F	1.52380831325501	0.83347981067423	2.61729391949670
F	1.31580866940620	-1.27939245389981	3.16759571938887
H	-0.68274875833968	1.80576210155540	1.50219554074235
C	2.88791152102777	2.04825905366006	-0.22186180067633
C	3.81546951702729	2.78324771124850	0.52388284499561
C	3.41089026324025	3.93308419440019	1.22736769336671
C	2.07089921872779	4.35372839149677	1.17070891179408
C	1.13954110657451	3.62172777034537	0.42235670178143
C	1.53573256990009	2.45614405758221	-0.27035550960563
C	0.52652441837756	1.65989801616908	-1.00160422329261
O	0.84244133808383	0.63379916084389	-1.72198796139938
H	4.86514808748655	2.45342389381669	0.56495342135665
H	4.14303875361073	4.50449245689181	1.81836629327130
H	1.75323175152844	5.25815117980285	1.71194585389888
H	0.08907665991585	3.95184546262250	0.36838161128122
H	3.19244571695288	1.14523487061608	-0.76976784125675
H	-0.47024788056910	2.14497083018262	-1.15037270882845
C	2.74842827010775	-1.59903907698919	-0.27630412373881
C	-1.04190539000095	-2.49699481695132	-0.71529629450000
C	1.35160091538136	-1.96207143208379	0.01231661832820
C	0.32664661054777	-2.02059650063100	-0.93675357129656
C	3.64317196493374	-1.37136283231870	0.79319990403597
C	-2.03637217157367	-2.15116653229744	-1.66017829104744
C	3.25173103547110	-1.43575820821948	-1.58987064981735
C	-1.45267538074827	-3.22969578054997	0.42549146509700
C	4.96200239332655	-0.95667838560415	0.56597689906985
C	-3.38447751740129	-2.45464032973910	-1.44395780270643

C	4.56873431749565	-1.02822333228238	-1.80821003582663
C	-2.80144395526132	-3.52918765207670	0.63316072208981
C	5.45755456652223	-0.76196996799853	-0.73898022348992
C	-3.80653166028938	-3.12854260299018	-0.28022198033895
H	1.17374066310066	-2.36343284841445	1.02152264542521
H	0.56324456043653	-1.76624353259698	-1.98185832237627
H	3.28572676776221	-1.48978492349759	1.82609987241879
H	-1.74215872880032	-1.59138760171383	-2.56215105609472
H	2.59996378479038	-1.62118248179034	-2.45519934901955
H	-0.70397127783288	-3.56681278528297	1.15850908568762
H	5.60826351800969	-0.77590210982158	1.43565202461866
H	-4.12090337016970	-2.12657985845837	-2.18799480278908
H	4.91399530587297	-0.91054529545401	-2.84686756783226
H	-3.08123603852840	-4.08619004783003	1.54067695311543
C	6.89183493934176	-0.29333836372733	-1.03035038679109
C	-5.28622027704590	-3.39142245296864	0.03607059526945
C	6.84702242141847	0.97827001462795	-1.90905241592169
C	7.64171227304305	-1.41463233316223	-1.78698581419828
C	7.66671563219527	0.03344861310793	0.25806983334935
C	-5.65410236826065	-2.59694262925371	1.31285462979963
C	-5.51082340783151	-4.90115998657302	0.27571230746055
C	-6.21125078290456	-2.92619470831890	-1.10249986167882
H	8.68838723057177	0.38045855724129	0.00307632042018
H	7.76939050291996	-0.85360013764228	0.91508069351785
H	7.17332853267516	0.83846835119668	0.84029306313139
H	7.14181589944387	-1.66655317254538	-2.74365362730204
H	7.69266578072144	-2.33889615823090	-1.17600585579586
H	8.67967801412613	-1.09729216425240	-2.01958213251044
H	7.87644704590492	1.33326813178733	-2.12350740861138
H	6.29963238266220	1.79712909245190	-1.39897711875426
H	6.34727836732177	0.79153552452332	-2.88007450149495
H	-5.50109285290531	-1.51168422331464	1.14621547041442
H	-5.03773251281178	-2.90775986818231	2.18049474594845
H	-6.71933624017242	-2.76509769376172	1.57693073504000
H	-5.99676748843139	-3.45941059893748	-2.05132029078002
H	-6.11426392229947	-1.83671057949316	-1.27822444407112
H	-7.26637133573690	-3.13537779639330	-0.83269550939157
H	-5.25691032259993	-5.49050418381168	-0.62927229427842
H	-6.57495966167072	-5.09463185376006	0.52421149537973
H	-4.89662097224422	-5.28478749332351	1.11489585217696

[Ni(L1)(stilbene)(substrate)]
Electronic energy: -5426.0586 Eh
Enthalpy: -5414.3137 Eh
Gibbs free energy: -5424.9099 Eh

H	6.13740603942179	-3.47870995839180	-1.25552349032023
H	4.48633625608189	-2.17212349036081	-2.65535117752031
H	1.42817496206147	0.79313077284332	-4.90082389902170
H	0.95179373614299	-0.89186812292126	-5.26206386474049
C	5.08680653577477	-3.38834048839718	-0.94076681434828
C	4.17049357187689	-2.64316250058989	-1.71420944006785
H	1.99349352648353	-0.53653914270177	-3.84258211455890
C	1.13252733450789	-0.17131451453543	-4.43857792777181
C	4.64267159081172	-4.01900791590315	0.21592134518138
C	2.84811001862356	-2.49521281453340	-1.30479760106380
H	5.33351647231207	-4.62506089559791	0.82283586011751
H	2.16662976746578	-1.90096648096253	-1.91936218586985
H	-0.65364965911416	3.96938585073460	-3.85484954270398
H	0.16576059937290	2.88977802598545	-1.83609209968348
C	-0.12220881371549	-0.03131397545421	-3.57450953586558
C	3.28775694064815	-3.92093557388345	0.63287206359560
H	-0.41544339656559	-1.01589676286179	-3.17155446699157
N	0.05612740788854	0.89625387597933	-2.44954226781094
C	-0.78253464038277	2.87152700393913	-3.77768762542360
C	2.36121402401863	-3.11373577653270	-0.11926362636467
H	-1.37081606210444	-6.60954139853840	-1.86850114013592
C	0.23644094675435	2.31929953388614	-2.78308677273017

H	-0.64941360728142	2.45028963048624	-4.79434445092154
H	-0.97806112390581	0.31606729315722	-4.19227350282120
O	0.35449113458349	-1.21341661586081	-1.13425824803059
H	-1.27301870652918	-4.12242985542287	-1.86059910234885
C	-2.29556705435131	-6.02908942074548	-1.72764878422787
C	2.83767958213405	-4.64910792478944	1.78652800643255
C	-2.23855565206673	-4.62874375073364	-1.71867829499751
H	3.57403156605969	-5.24065619516698	2.35232766478361
H	-1.82024242422820	2.67282696419211	-3.44884285634218
C	0.97651045747731	-3.02443423969743	0.34244354217150
H	-3.56704712683061	-7.78968393523647	-1.56569935444539
C	-0.01965275404152	-2.16301566995515	-0.21263338518680
C	-3.52500953883159	-6.68960641531284	-1.56417421228871
H	1.26336886973810	2.49378625137659	-3.18174760957381
P	0.17924231891196	0.46231336579984	-0.81858417358370
C	1.51663273340050	-4.64541750585617	2.14931147618500
C	0.55010700491248	-3.86402591599163	1.42734489333248
C	-3.41143030625472	-3.86045128982430	-1.54232997162318
C	-4.69941176147226	-5.93702824393138	-1.40246353187766
C	-1.39319972638939	-2.31130893242253	0.07104460479563
C	-3.37786071385018	-2.37572930345354	-1.52774644093042
C	-2.39639607505784	-1.63404835511529	-0.80107781811313
O	-1.40416391481431	0.40387227004814	-0.16970300397921
C	-4.33160618918357	-1.66069155905873	-2.25425768478248
C	-2.40333078853468	-0.23059046413138	-0.86443733815646
C	-4.64219653616780	-4.53694806861262	-1.39220818645135
H	1.16574411767981	-5.23988944439727	3.00674530252078
H	-5.07204739646094	-2.21016330225787	-2.85479720755137
C	-4.37000333157203	-0.24619117574850	-2.28051308805700
C	-3.39812666928015	0.52444746647081	-1.55314874961249
H	-5.66711524503955	-6.44424604254416	-1.26756954406180
C	-0.81104240588604	-3.90189015884018	1.80892314874731
Ni	1.23926767319164	1.67367102782831	0.57505917777894
C	-5.37062474709730	0.41927797701378	-3.06890456498677
C	-1.79764876760547	-3.18607845224927	1.12866807628434
C	-3.48716211861694	1.98430387371878	-1.57070307460567
H	-6.09270622652920	-0.20049205533949	-3.62239495824737
C	-2.65907211365774	2.85192633428867	-0.80358200757443
C	-5.41733298466158	1.78597694512481	-3.14232279808566
H	-1.92336250992878	2.43101510587313	-0.11435381098967
C	-4.49341612321048	2.59902404671992	-2.40097256760336
H	-5.55980790583722	-3.95226295500876	-1.22731482008157
C	-2.77718072137484	4.23739533064638	-0.88844435282240
H	-6.17655578459778	2.28976760176150	-3.76072780808720
C	-4.59037428714505	4.01441255253401	-2.46996373618164
H	-1.09272465852302	-4.52342882408257	2.67174599867760
C	-3.73834795123523	4.83130996328495	-1.73408826926589
H	-2.10750184481171	4.86384930996770	-0.28172693262293
H	-5.36488820027428	4.45332909011539	-3.11846031779305
H	-3.82139034123450	5.92683734656532	-1.80107423084070
H	-3.07767186333311	-5.49296909681887	1.77683697444983
C	-3.21735876241941	-3.33629433222036	1.53909949739077
C	-3.72767386162797	-4.61039746494667	1.87317535269949
H	-3.70299711342504	-1.22312509168525	1.39008591467083
C	-4.08334869150395	-2.22399795619209	1.63076061090142
C	-5.05981415474090	-4.76837459772371	2.27909777451695
C	-5.41517038485614	-2.38021136958465	2.03672481331047
H	-5.43979394102216	-5.77280258060531	2.52118559346760
C	-5.91135643477751	-3.65449721801627	2.35973382443987
H	-6.06896771275173	-1.49633233378278	2.10694952624646
H	-6.95879403164006	-3.77835622851261	2.67487328865100
H	6.75489735602752	-1.91701865648346	-3.18646050956841
H	7.71074065786352	-0.67715276758701	-2.28746412012122
C	7.04002432148039	-0.84240769295372	-3.14936979133618
H	7.57319286614754	-0.58049866426353	-4.08568766931037
H	3.01899289430058	3.28431655849914	1.64193940089986
N	5.88789822657618	0.01167831098208	-2.96637032130532

O	4.90640718622819	-0.11569667940009	-3.95778159144026
H	4.31912236063067	1.54648633695208	0.40951505130748
H	6.09180333185077	0.66205361180174	-5.52497231500257
C	2.31608378374075	3.35530525864691	0.79414304035582
C	3.77787931532741	1.79191341021332	-0.52156350355605
C	2.64199800976932	2.69302675512547	-0.42920837851166
C	5.11267945440751	0.81798071493990	-5.02196082715826
C	1.58798432292564	4.65427755663279	0.76618242062009
C	5.48475891553355	0.47006258864760	-1.71448881427082
C	4.23887642219480	1.25804888470945	-1.68943543015738
H	4.29728243604844	0.62257690766457	-5.74456924717966
H	2.26932675538562	3.10558868112131	-1.37958155130137
H	3.70820188955122	1.45442731298973	-2.63219334202213
O	6.21043648105561	0.27440436612486	-0.72564333223604
H	5.04591001337553	1.86596922568240	-4.65758555953787
F	1.13978632257716	5.01431913310008	2.00570002048692
F	0.49691200904921	4.64616129148204	-0.06087663262850
F	2.36692732348467	5.69044689588707	0.34009014647213
C	-1.34509227835643	2.19061619481588	2.58959944795654
C	2.17394628480592	0.51013495798195	2.68538162215430
C	0.09970664581714	1.95110602007151	2.62240638803965
C	0.73545059941712	0.72141423391099	2.44642819863143
C	-2.30514631301862	1.14974351919981	2.56745962416996
C	2.97818460626296	1.37537984933279	3.47026606498365
C	-1.83079259204011	3.51802582328264	2.59626349554027
C	2.81641513602683	-0.58817453975129	2.07080889485931
C	-3.66947466546944	1.43497718814963	2.51121676544296
C	4.35413224390058	1.16626998527744	3.58111538670775
C	-3.20237377912298	3.79543130584417	2.53380655649336
C	4.19721782868840	-0.78840867783708	2.18092688307511
C	-4.15732248061195	2.76196079898026	2.46965591580919
C	5.00511001968369	0.08851817783969	2.93020200060206
H	0.70522566811347	2.80672727480787	2.95768554117302
H	0.13438880135483	-0.18110745081822	2.24646554359399
H	-1.97714731391913	0.10085329004683	2.59908284088805
H	2.51509353635946	2.21844071079505	4.00504883058110
H	-1.11061786317506	4.34896194573196	2.62251557812669
H	2.21449673220603	-1.28587681007533	1.47662961719254
H	-4.38100595230922	0.59597568682668	2.49822513620366
H	4.94087358947973	1.86234428569012	4.20041281447740
H	-3.52238100818986	4.84606359401515	2.51882068343564
H	4.64018546830145	-1.64267908172928	1.65341351728509
C	-5.66669936456561	3.01601778829042	2.34056703569678
C	6.52548667968713	-0.08487605459229	3.05936997418024
C	-6.16370989957594	2.39559757557629	1.01335875579738
C	-6.00199073796759	4.51737212452479	2.32916670123100
H	-7.09848220254713	4.65560500950921	2.23896589163054
H	-5.52783871386797	5.03665596078418	1.47150767942360
H	-5.67531252089115	5.02011028452902	3.26205823235996
H	-5.66033574598402	2.86377020982542	0.14449390863248
H	-7.25808162054700	2.54608252332978	0.90395908925335
H	-5.96436246991628	1.30567026124782	0.96787113044856
C	-6.40313842416447	2.35464458460015	3.52796649930644
H	-7.49692809012558	2.52433846782568	3.44559894329685
H	-6.06071810386830	2.77817940451294	4.49430686693797
H	-6.23447071976247	1.25938806464221	3.55876457167640
C	7.22190399788539	1.16982024250378	2.48049456164908
C	7.03070207253884	-1.31803291281682	2.28858327218487
C	6.89497535810837	-0.24575512205448	4.55158073346474
H	6.90904109042140	2.09311669200044	3.00881700015667
H	6.98257367586977	1.27859650091707	1.40409736575558
H	8.32331245835533	1.07816114491078	2.58656225915068
H	6.81594498555394	-1.23078531570440	1.20545715664793
H	6.57396929561791	-2.25562466809206	2.66672386038993
H	8.12929807431785	-1.40789775292651	2.41194985895508
H	6.59484279201248	0.63675395830235	5.15176829888855
H	7.99252915961455	-0.36866941313402	4.66222664134411

H	6.40334534917941	-1.13865936721629	4.98976958441754

(R,R)-K.1•BEt$_3$
Electronic energy: -5178.7043 Eh
Enthalpy: -5167.7967 Eh
Gibbs free energy: -5177.6746 Eh

P	0.45859055319774	1.58408084094282	-0.46919622933221
O	-0.91302579549037	1.37066212708219	0.53569945252348
O	0.25984444519140	0.27579755474713	-1.52545388452173
N	0.08280748382708	2.77444620846695	-1.59981293637676
C	-2.38562252379018	-0.00536537028175	-0.70810349322875
C	-1.40890213158904	-1.11719951670080	-0.54569899805715
C	-2.14266837077122	1.23444981080656	-0.09403008001575
C	-0.11404330162284	-0.97911753076000	-1.07590001952703
C	-3.08775159533749	2.30670601633804	-0.11323076073231
C	0.76934133162006	-2.08796757408180	-1.25241042114171
C	-3.57258593101331	-0.20033580822842	-1.47842598280146
C	-1.81104874814927	-2.36023025283497	0.02945069651024
C	-4.25010400118179	2.10184927092712	-0.93627816335654
C	0.35137668907946	-3.32193871120473	-0.64013457348154
C	-4.46323580568317	0.86632231470076	-1.58889822670174
C	-0.90841724020039	-3.42218605547828	-0.00616602434378
C	-2.95624617798669	3.57397707195365	0.60182861699377
C	2.02389621282803	-2.06320121031424	-2.00262692897852
C	-3.89577701643036	4.63020876694768	0.32268319209560
C	2.87441646490909	-3.22565948594222	-1.95980165953234
C	-3.87628239714868	-1.47230712472037	-2.18442846474962
C	-3.12374366365743	-2.53288280986690	0.69715174578919
C	-5.18559204638884	3.17278959841628	-1.14553178103846
C	1.23737204404191	-4.45351520430542	-0.62974262407110
C	-4.99637400651100	4.40071895081506	-0.57060439798706
C	2.46433074993868	-4.39587978204660	-1.23499925691595
C	-1.96670803211367	3.82644586934167	1.59218747643809
C	2.43837812549813	-0.98303023673504	-2.83034695957037
C	-3.75618363550535	5.89166875899181	0.96077644122126
C	4.10296118369395	-3.23323503313179	-2.67132021573329
C	-5.16565424138353	-2.03785447702750	-2.07573193971470
C	-3.86245439391748	-3.72090998710869	0.50834432454507
C	-2.91815116892192	-2.12698247142390	-2.99006497149428
C	-3.65184928748897	-1.53994071932753	1.55204530351610
C	-1.86214584114110	5.06919210091960	2.21515247808858
C	3.63245296034772	-1.03110143356923	-3.54601438608166
C	-2.74574149194023	6.12026029084944	1.88693306726748
C	4.48569342605241	-2.15143310547984	-3.45546288334205
C	-5.48617170271084	-3.22917161440813	-2.74077752317791
C	-5.09761088839614	-3.90827136757605	1.14269500882158
C	-3.23812608730729	-3.31952630455943	-3.65333833709299
C	-4.88906194871588	-1.72536678796310	2.18416848442147
C	-4.52140838045509	-3.87813553424438	-3.52789289377803
C	-5.61931655756768	-2.90858699832660	1.97990382992356
H	-5.35261548469917	0.76011877163336	-2.22768483832656
H	-1.16839976886750	-4.36721509452780	0.49167220755203
H	-6.05004976038876	2.98380987006489	-1.80034800470912
H	0.90309059032914	-5.36722140510578	-0.11480632129976
H	-5.70078398763189	5.22575114924974	-0.75836533650710
H	3.14476435177197	-5.26117889099360	-1.21082026600062
H	-1.27994717494408	3.02017125347303	1.86862900119225
H	1.80424176714567	-0.09632326608109	-2.91683333388429
H	-4.48015812585041	6.68420008366949	0.71468787120199
H	4.73952767521030	-4.12940290831013	-2.60451141441827
H	-5.91188182362844	-1.55086409100873	-1.42973862022869
H	-3.47675684355957	-4.49082697086125	-0.17669134278079
H	-1.91622298462029	-1.69050539597928	-3.10950361312986
H	-3.08003703407170	-0.62100424618568	1.73978105673791
H	-1.08131371141169	5.23757325434692	2.97156935477738
H	3.91256297668821	-0.17862363074826	-4.18313467502204
H	-2.64603836703857	7.10182808608673	2.37427241755459

H	5.43635272461072	-2.17303773628426	-4.00950594854395
H	-6.49290625326211	-3.66099126347524	-2.63181570469733
H	-5.66459208607939	-4.83610413353306	0.97058059013586
H	-2.47908305009269	-3.81345171570789	-4.27921168623134
H	-5.28252037390461	-0.93970132306964	2.84728989129631
H	-4.76935057904724	-4.81693786144217	-4.04663232167164
H	-6.59332497115220	-3.05062995344448	2.47294517521585
C	-0.78154644138264	2.52953598946093	-2.77475744927877
C	0.17779799831262	4.16689109416961	-1.10953569718140
H	-1.35377992280005	1.59607856434027	-2.62603936864853
H	-1.53552242508766	3.34412508209870	-2.81311668497412
C	0.00209679983203	2.44181966500125	-4.08278956097834
H	0.71819167237623	1.59649215301153	-4.05237153776123
H	-0.69100042357660	2.27298981420152	-4.93238619891742
H	0.57693910178076	3.36632202187755	-4.28342503905888
C	0.46556664753722	5.21287621246750	-2.18236823038769
H	-0.76983849547618	4.44336650564922	-0.59739094111011
H	0.97388680328364	4.18686906997409	-0.33633813788716
H	1.42152962441108	5.02951296898403	-2.70976101307755
H	-0.34781922518916	5.26830351140908	-2.93241395728543
H	0.53289381883330	6.20672091915925	-1.69681372841585
Ni	2.32502480167221	1.55337955437901	0.77292759858533
H	-3.58945744830420	-2.56852737381705	4.49064280730534
H	-2.68397533097567	-1.38525877534417	5.52279712224670
C	-2.62990508413654	-2.36338065712782	5.01115730839738
H	-2.44405411197846	-3.15888255281420	5.76349686717414
N	-1.54998761010271	-2.28054765770949	4.05768945096353
O	-1.38274110263694	-3.40186395595852	3.24859020538739
H	1.28600637767991	0.39555698963196	3.37132035515137
H	-0.37500424507940	-5.15694321253045	3.09990262173776
C	3.44837169269425	0.15142687808562	1.66613448790878
C	1.15920892248031	-0.34540742427030	2.56435943317205
C	2.13189295864598	-0.35262135750870	1.48043041631615
C	-0.39912105232451	-4.29157920420589	3.78973896865810
C	4.60815734499687	-0.42780760067550	0.91602084485715
C	-0.83006245489202	-1.12892168900163	3.79188521605781
C	0.12788716215858	-1.22276083468226	2.66349881519942
H	0.60478326662564	-3.81461685707636	3.81985841334929
H	1.95464366109141	-1.06171806577553	0.65508930804952
H	-0.01983419146968	-2.01198795735890	1.91423120280960
O	-0.98955596361251	-0.10919050139726	4.48065856005642
H	-0.67829220051647	-4.64187211623328	4.80723714816032
C	0.96028393544358	2.54163970205015	5.43435922576595
H	0.32849249934961	2.38923215566655	6.32153544259129
C	0.41930711595723	3.14242652920878	4.28446310344327
C	2.30489259260404	2.13448306909007	5.44641969924811
H	-0.63252013739208	3.46222696478991	4.28664685955971
H	2.73847701467951	1.68476675162352	6.35226529205450
C	1.20494519484495	3.31989072585736	3.14007502041626
C	3.09296942441431	2.29190824012045	4.30078790418819
C	2.54525612856061	2.86571251230968	3.12750916301519
H	0.79987564602326	3.80860655783741	2.24298691306870
H	4.14683683340886	1.97196955564406	4.30595234462616
C	3.36805450407054	2.90068570646157	1.91619544034592
O	3.00464950008210	3.61455509449362	0.86855297114800
H	4.43914222763932	2.65592762967593	2.01460734004255
H	3.75295197390560	0.43956696401431	2.68488903773625
F	4.31035576746779	-0.78978372560304	-0.35329916109366
F	5.07595045465511	-1.54664331842505	1.54184836100204
F	5.65788793174281	0.43834931672793	0.85905274250649
B	4.02318782305000	4.28818299873817	-0.10953722713399
C	5.56234585026286	4.05034815041230	0.40032728091593
C	3.77535377716342	3.63414633221619	-1.58901898691087
C	3.54021631843862	5.85876051344762	-0.03249304927609
C	4.06742541445976	2.14205814716187	-1.71261633277108
H	4.41039288748321	4.19292476109985	-2.31262419525315
H	2.73150961266410	3.81209013739431	-1.91967275470284

H	6.21416949205736	4.35726213778751	-0.44972248914664
H	5.78642663828194	2.96508435595152	0.51626996377699
C	6.02457836585622	4.80937407610796	1.65431531628424
H	3.62183325004894	6.22119150685515	1.01859742973444
H	2.45644743163600	5.92556235435980	-0.27718397576199
C	4.32430095677507	6.80138107259871	-0.95271439904064
H	5.95728267051121	5.90787014725506	1.50987964958672
H	7.07630326272908	4.58300047055256	1.93562163136530
H	5.40388885607704	4.58357888389787	2.54976495274001
H	5.12252716258174	1.88308222968450	-1.49213225278530
H	3.82176385173595	1.72575878403184	-2.71152403601921
H	3.45307899528543	1.53858700242680	-0.99055454077537
H	5.41190060423388	6.78496965866011	-0.72634171641987
H	3.99349538862213	7.86013011186991	-0.87111497110794
H	4.22027384270402	6.51163878086806	-2.01987799223624

(S,S)-K.1•BEt$_3$

Electronic energy: -5178.7140 Eh
Enthalpy: -5167.8080 Eh
Gibbs free energy: -5177.6848 Eh

P	0.99583703972831	0.71564090804659	-1.12550522466987
O	-0.22657316056004	1.24417608844238	-0.04144352163070
O	0.26326767150570	-0.65598621726198	-1.77137062644747
N	0.90227963971387	1.68952268578796	-2.48644831936669
C	-2.21110955582980	0.25797940440936	-0.86088731563531
C	-1.66334138732227	-1.06261619717004	-0.43822150848473
C	-1.50899590031917	1.42793748604345	-0.52519456815565
C	-0.44250572664348	-1.53029815742249	-0.95516022238461
C	-2.08082866078635	2.73481351427908	-0.61187803452159
C	0.02636766348280	-2.86574003377660	-0.76606162958080
C	-3.49417291884330	0.35544429119125	-1.48096208612824
C	-2.41506295717284	-1.88856886017891	0.45265421974676
C	-3.38603990703847	2.80113894883081	-1.21639581061231
C	-0.73641190142894	-3.66362846084053	0.15654642353921
C	-4.04031605201257	1.62661801307240	-1.65545634115372
C	-1.91742881073114	-3.15682453606485	0.74251398995179
C	-1.46785509395789	3.97300685455153	-0.13019517659523
C	1.15926171950499	-3.48416323394093	-1.45556787640471
C	-2.14891150337821	5.22167161039071	-0.36686193001961
C	1.54745255576175	-4.82149500726217	-1.07946372247259
C	-4.24156818055325	-0.83748908662238	-1.95108209516909
C	-3.67547392965476	-1.44093589953178	1.09735155617057
C	-4.02114947972212	4.07257852999597	-1.42720266499993
C	-0.29416963747583	-4.98367279270536	0.50911654920362
C	-3.41915328749578	5.23828319174199	-1.03660906640563
C	0.81796917120200	-5.53274439297872	-0.06772026098100
C	-0.24843002037745	4.03177091629907	0.60077960850516
C	1.87795839120424	-2.87800739460923	-2.52489415132926
C	-1.57828888499747	6.44050412333120	0.08834454303183
C	2.64292054211927	-5.45369249387924	-1.72527003726675
C	-3.59482824355932	-1.88654132632318	-2.64228318351686
C	-3.80129708739686	-0.16112685240799	1.68183821227063
C	-5.63332639682022	-0.93334754862064	-1.73062759274961
C	-4.77563903398191	-2.32205661931925	1.17144950997467
C	0.28356131884979	5.23858777445882	1.05053941870235
C	2.93050198082947	-3.53410905198261	-3.16009600764142
C	-0.37655898741361	6.45800026247212	0.78718639975023
C	3.33472657858825	-4.82260685818790	-2.75203314839321
C	-4.31715259526342	-3.00242872002244	-3.08609694021910
C	-4.99206515595920	0.22552790350009	2.31121954054457
C	-6.35616452626473	-2.04778826455186	-2.17694168674553
C	-5.96679119216943	-1.93770347461503	1.80267685269724
C	-5.69982750824248	-3.08999506141902	-2.85168155631815
C	-6.08221989781290	-0.65979097090708	2.37244422312034
H	-5.00827961050400	1.73221377714487	-2.16731104224984
H	-2.45390421133924	-3.79073830706089	1.46273956964517
H	-5.00671130349525	4.08337620498767	-1.91728089713317

H	-0.87674618389033	-5.54161937396827	1.25751174883045
H	-3.90862636969965	6.20956116508289	-1.20782175719307
H	1.15648873374040	-6.54320711447661	0.20900558582179
H	0.28923292348445	3.10691939939076	0.81613497228939
H	1.60504578522395	-1.87545830388576	-2.86361252482887
H	-2.12015163767727	7.37732597722009	-0.11570558864583
H	2.92521792288718	-6.46723292464432	-1.39993135932958
H	-2.51694412100032	-1.81818666004979	-2.84725270490315
H	-2.95072449717051	0.53444218580099	1.65908802633874
H	-6.14412212217149	-0.13846863213684	-1.16660389332660
H	-4.70372342030850	-3.31153354596172	0.69508257167200
H	1.23039049165698	5.22761835466585	1.60954501415464
H	3.45911591151169	-3.02872920245850	-3.98118732684787
H	0.05110936074049	7.40963524310734	1.13759093894923
H	4.18091179154167	-5.32247895983682	-3.24693544611096
H	-3.79556447495229	-3.80802578221781	-3.62524032936287
H	-5.06782848626019	1.22723254139556	2.76192538494605
H	-7.43801079783218	-2.10959893609848	-1.98289364493572
H	-6.81612010129522	-2.63730221678409	1.83670742626919
H	-6.26519392211408	-3.96899286649359	-3.19748885216490
H	-7.01867380266898	-0.35299221959810	2.86318617692400
C	0.19378009018980	1.37978188242469	-3.73774288484185
C	1.45891353252738	3.04552412461974	-2.31214264958462
H	0.91895595569516	1.51947682289709	-4.56845019186411
H	-0.05570625068436	0.30372499656806	-3.72582903188741
C	-1.06598187579299	2.20433576867205	-3.99070944559772
H	-1.46393559141103	1.96624045081830	-4.99803058924384
H	-1.85550966639577	1.97789179444334	-3.25086811923407
H	-0.86879522384369	3.29484375104894	-3.95930028568594
C	2.17566533178421	3.60087478747782	-3.53786849271746
H	0.65110440826133	3.74513308024946	-2.00088868940158
H	2.16689228024106	2.99994316977289	-1.45711360445769
H	2.65037313186481	4.56499125917080	-3.26827212791588
H	2.96824002093998	2.91928719412674	-3.89925996812024
H	1.47656469338661	3.79987017729584	-4.37404608528873
Ni	2.90240211932346	0.42509147493632	0.01115368874036
H	-0.22957879218889	-4.65544169298865	4.29340260232874
H	-1.90179794126026	-4.56111537292397	3.60808947923533
C	-1.16276691248314	-4.06430691842431	4.27381254123545
H	-1.58470874437653	-4.00706076999936	5.29835922737211
N	-0.82378157284553	-2.75021497397862	3.78062331416976
O	-1.89784356941529	-1.87515549659771	3.63650124868391
H	2.60778476451383	-1.27276220938597	2.50704731854737
H	-2.29702549788381	-1.73019782041516	5.70610864247657
C	3.34601866694369	1.23760669611127	1.78853804404020
C	1.74931300577155	-0.60842825024900	2.31049221658347
C	2.02332350880953	0.73123245142752	1.81483221333505
C	-2.09983541553138	-1.09160384806938	4.81782276514884
C	3.61430689906513	2.71106401854490	1.78155799674685
C	0.38211715314555	-2.45585695977927	3.16793285291951
C	0.50916973609200	-1.09034684135996	2.59598975336347
H	-1.22738547719722	-0.43237403902843	5.01931821608350
H	1.16884747876237	1.42045080841675	1.73831109111031
H	-0.39275514007712	-0.48137564394945	2.44295402202873
O	1.30182247859027	-3.28685747746215	3.16490750394319
H	-2.99397908457049	-0.47543079215873	4.60457433915431
C	4.73354298128483	-3.81608426883071	2.56139796455112
H	4.72190398778951	-4.68334087365989	3.23854193668165
C	4.02285302342486	-3.86070013770078	1.34989131842644
C	5.45715024012792	-2.66261669548137	2.90743671795819
H	3.45291565124922	-4.76046899033402	1.07915755405487
H	6.02779047805067	-2.62893059869415	3.84797781637123
C	4.01923858621013	-2.75802180899709	0.48963628039571
C	5.45767624599243	-1.55383860904244	2.05187312592724
C	4.72825229336765	-1.58613792131342	0.83973693688839
H	3.46808514351209	-2.78724922605699	-0.45912851986393
H	6.04028718076560	-0.65558385462268	2.31188004555593

C	4.70916043434135	-0.38493348408842	-0.00208567037411
O	4.21036690285234	-0.44744912328775	-1.24184807472013
H	5.49206282643864	0.37334792857961	0.18083414281528
H	4.13826604091497	0.68427884157924	2.31788190662522
F	2.83687566809309	3.39330638271395	0.89157039959984
F	4.90605725035860	2.97871592921722	1.47328042150936
F	3.37832168742101	3.27489311486426	2.99867464931017
B	4.91051012871810	0.33129931053833	-2.37821922451955
C	6.36036231516043	-0.42055529447045	-2.56180897434953
C	5.04849981059771	1.91369271336828	-1.92167464498372
C	3.91820084972522	0.15313280482173	-3.66216978058278
C	4.47323862031294	0.71247158869245	-4.97642074851793
H	2.94097265539335	0.63373113020223	-3.43843938261518
H	3.68301280573829	-0.92345127447896	-3.80917782999831
H	6.95860972790063	-0.29913745463017	-1.62800217575914
H	6.93529911950778	0.13762044093809	-3.33652420211999
C	6.31071548301958	-1.90642808086979	-2.93374819538691
C	6.35922642572295	2.43111404535070	-1.31560755583412
H	4.20565891279646	2.11734207225628	-1.20804874653398
H	4.81449206318811	2.55380578825491	-2.80030114577288
H	4.75753811502500	1.78382405978855	-4.88400353076567
H	3.75377505383010	0.64536134451890	-5.82209809928088
H	5.39333082671019	0.17500894101130	-5.29058979041742
H	5.74112584089854	-2.49452349899416	-2.18273753063466
H	7.31823218356670	-2.37017671619483	-3.01590755624224
H	5.80247228325387	-2.06780471252606	-3.90824379526223
H	6.65737509662748	1.88990337173419	-0.39223216513601
H	6.31397933805969	3.50843500087924	-1.04577362373060
H	7.20080303098894	2.30765284614542	-2.02939100469225

NMR Spectra

^{1}H NMR (400 MHz, CD_2Cl_2; top), ^{13}C NMR (101 MHz, CD_2Cl_2; middle) and ^{19}F NMR (282 MHz, CD_2Cl_2) of compound **12**

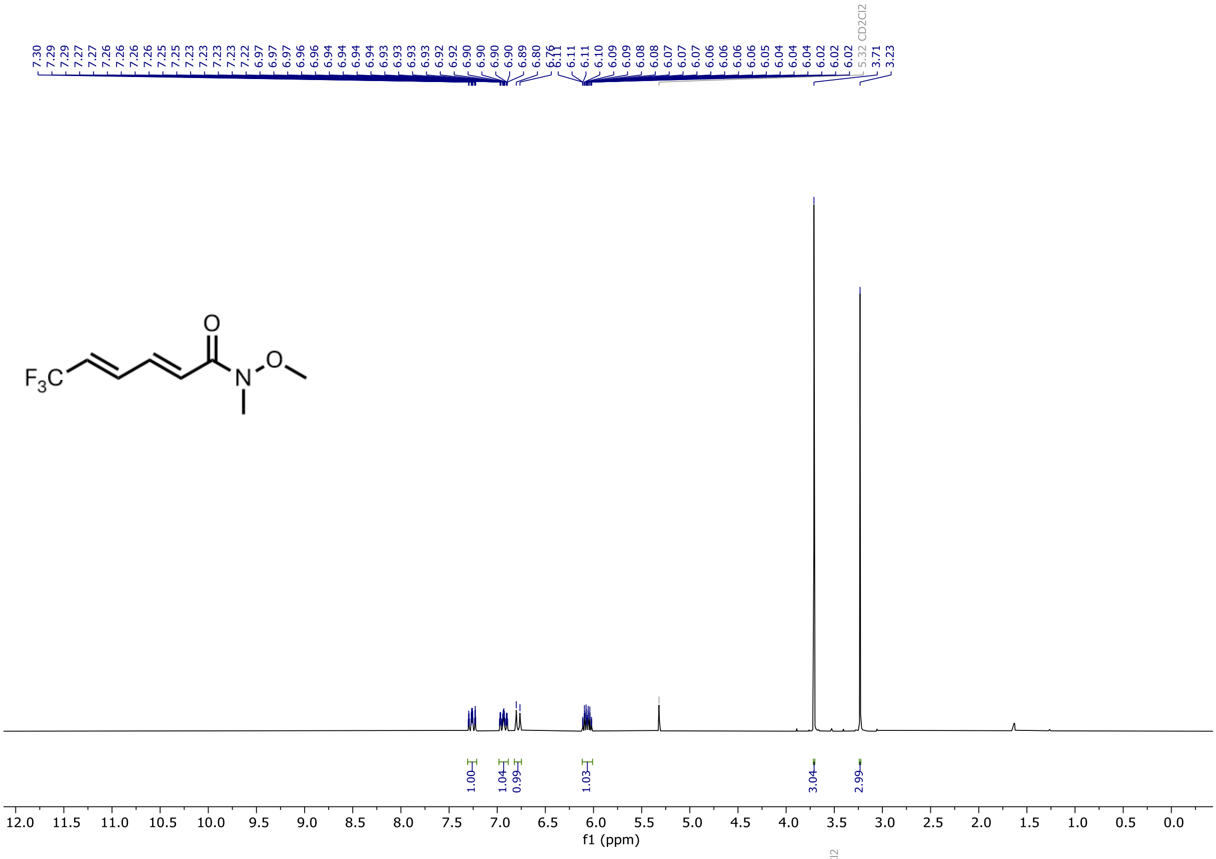

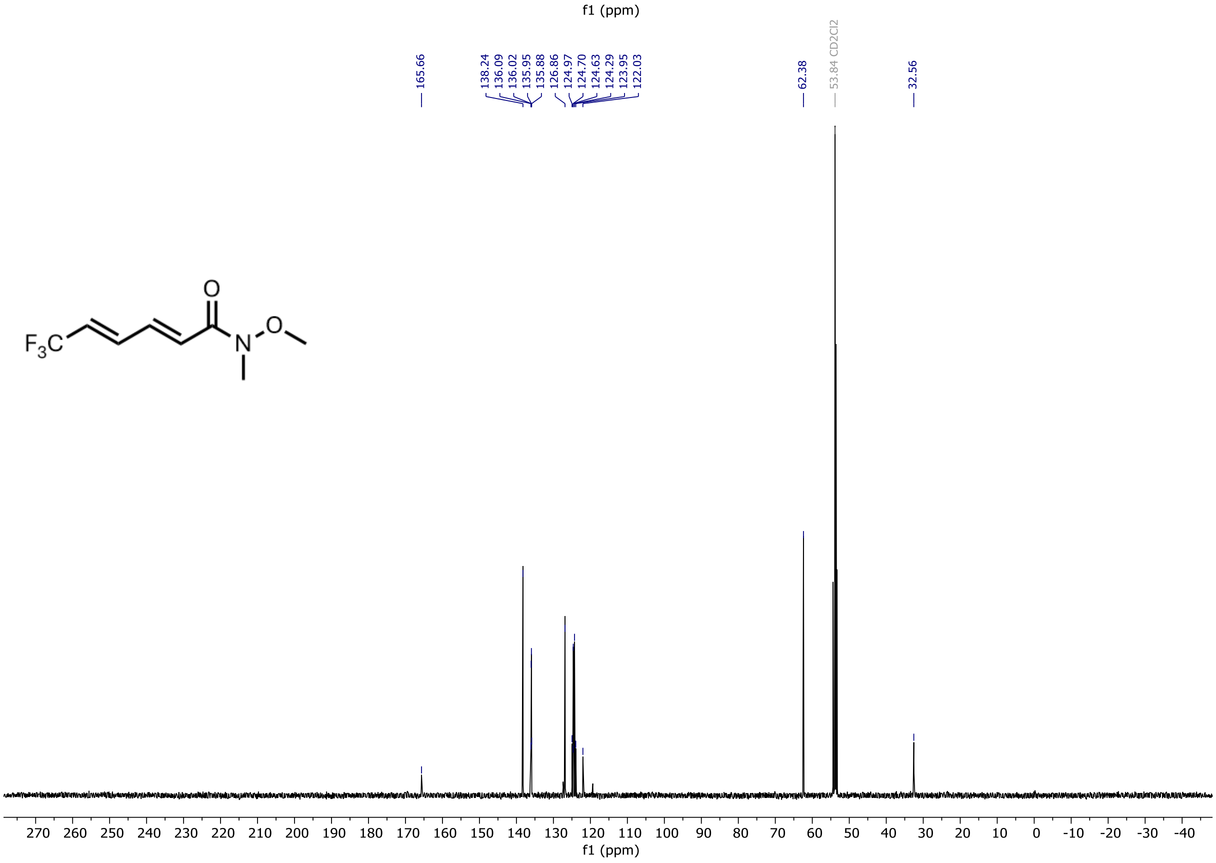

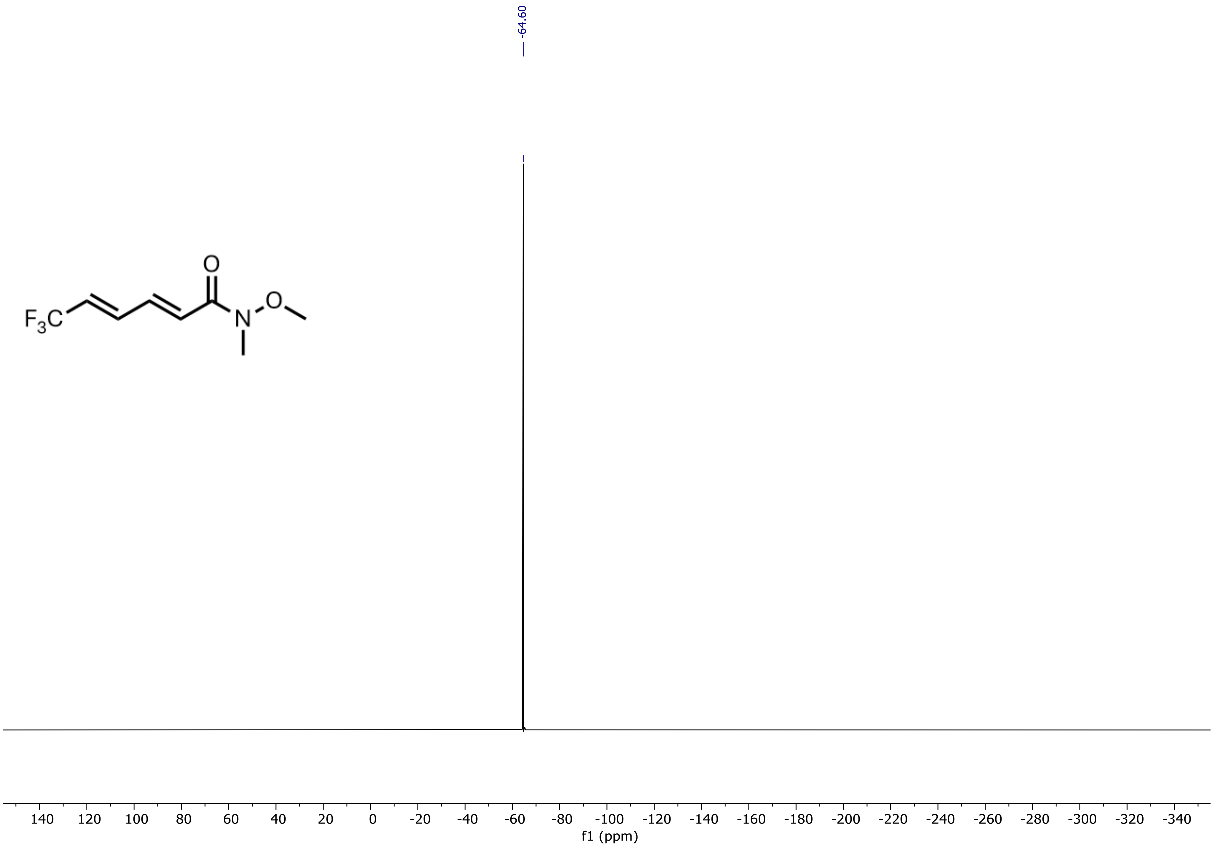

-64.60
F3C
O
N
O
140
120
100
80
60
40
20
0
-20
-40
-60
-80
-100
-120
-140
-160
-180
-200
-220
-240
-260
-280
-300
-320
-340
f1 (ppm)

^{1}H NMR (400 MHz, CD_2Cl_2; top), ^{13}C NMR (101 MHz, CD_2Cl_2; middle) and ^{19}F NMR (282 MHz, CD_2Cl_2) of compound **15**

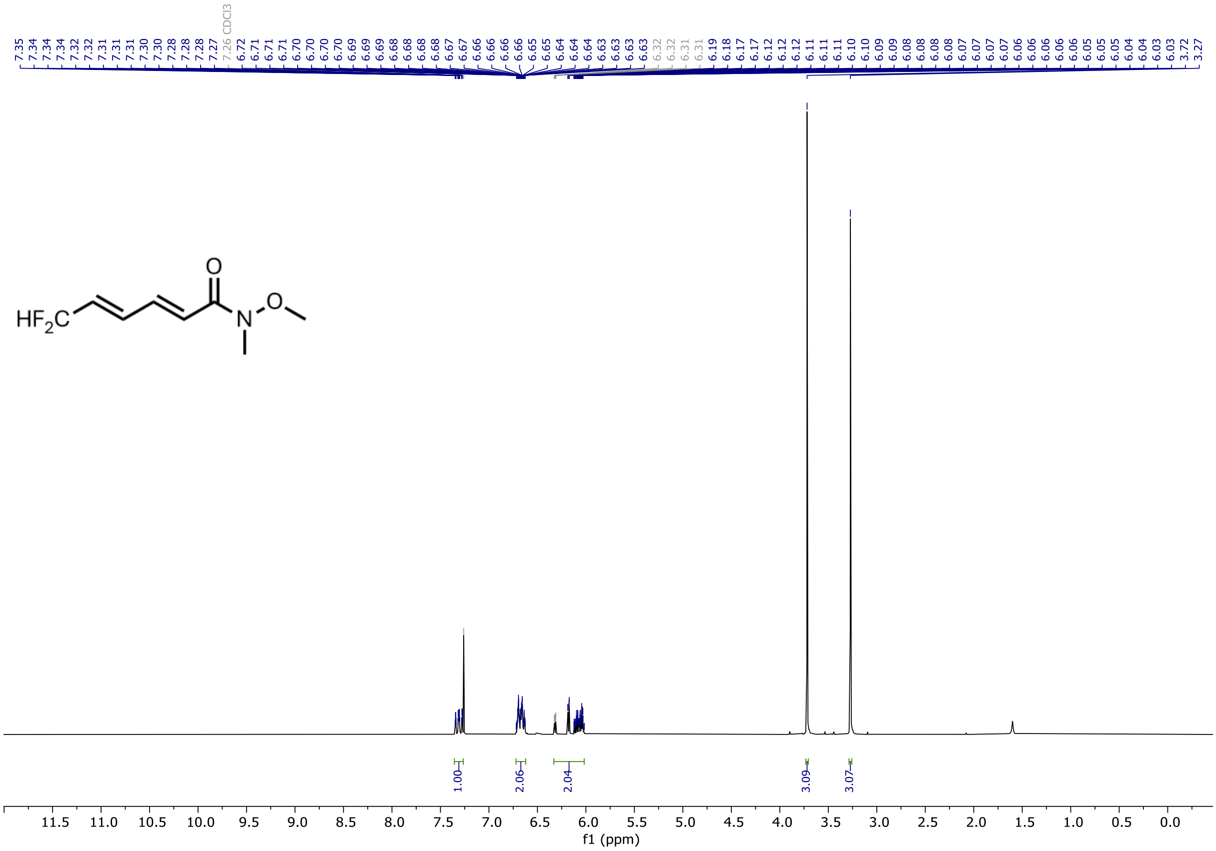

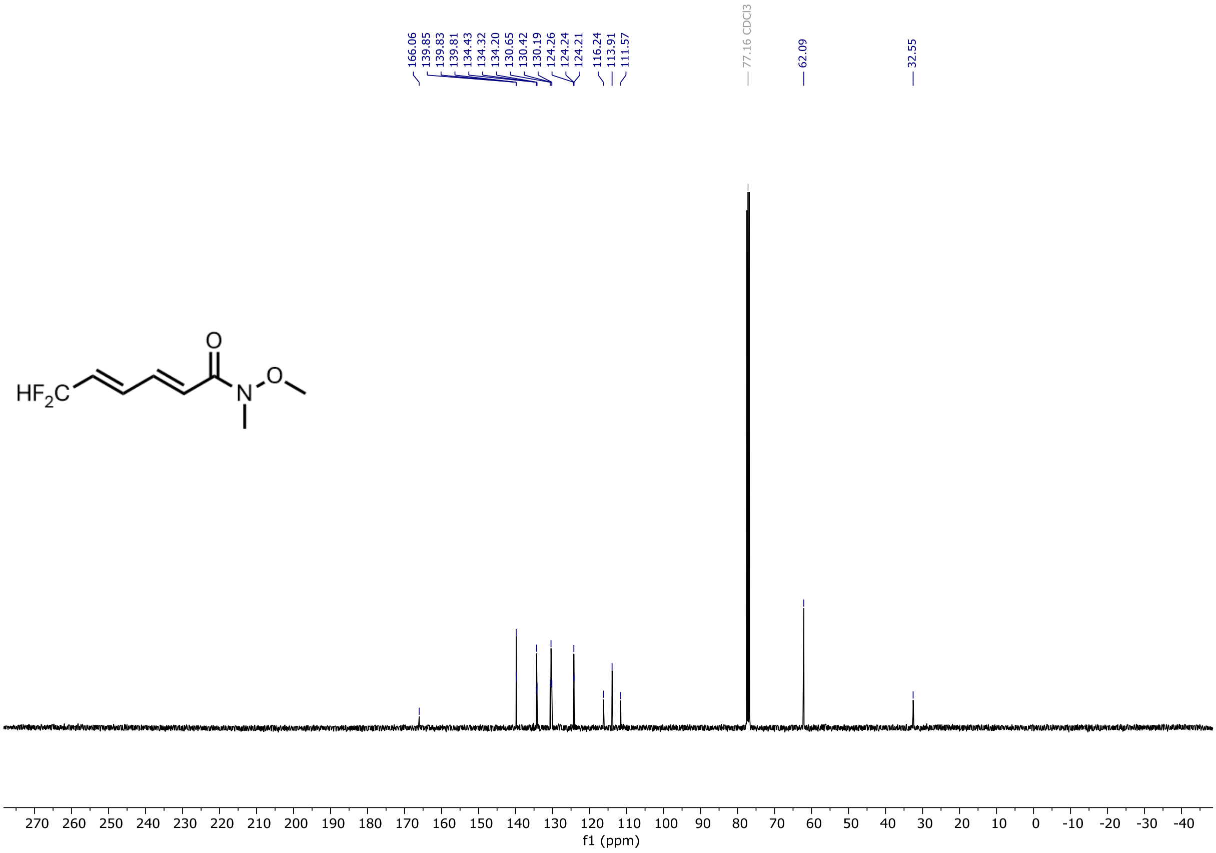

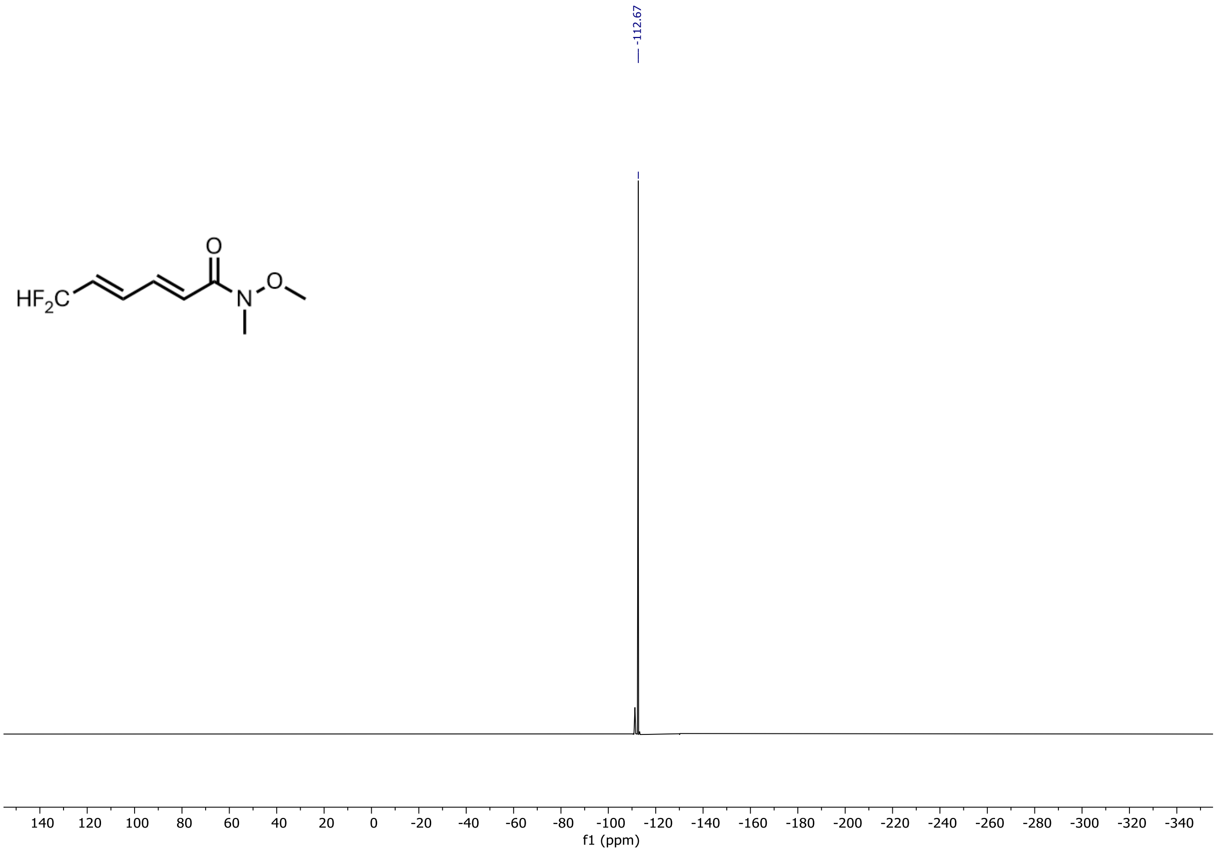
-112.67
HF2C
O
N
O
f1 (ppm)

^{1}H NMR (400 MHz, CD_2Cl_2; top), ^{13}C NMR (101 MHz, CD_2Cl_2; middle) and ^{19}F NMR (282 MHz, CD_2Cl_2) of compound **17a**

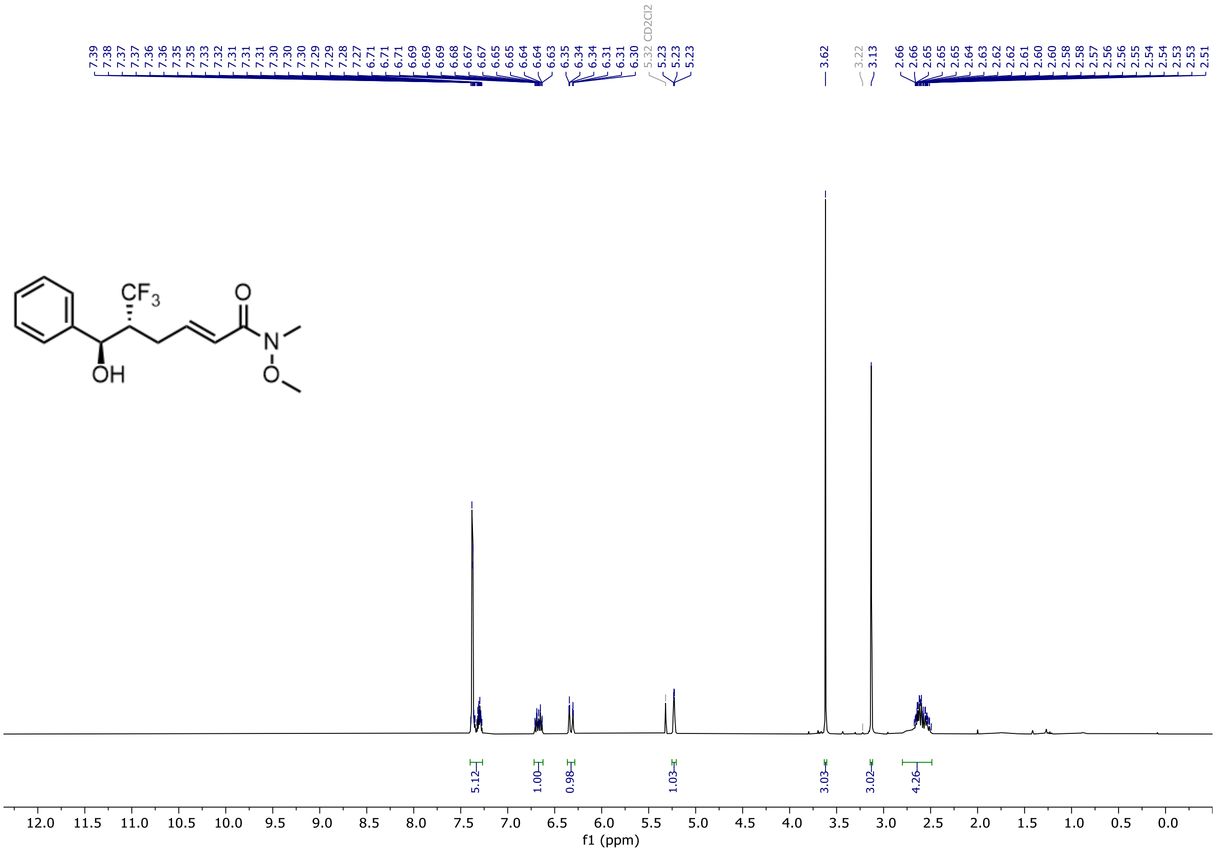

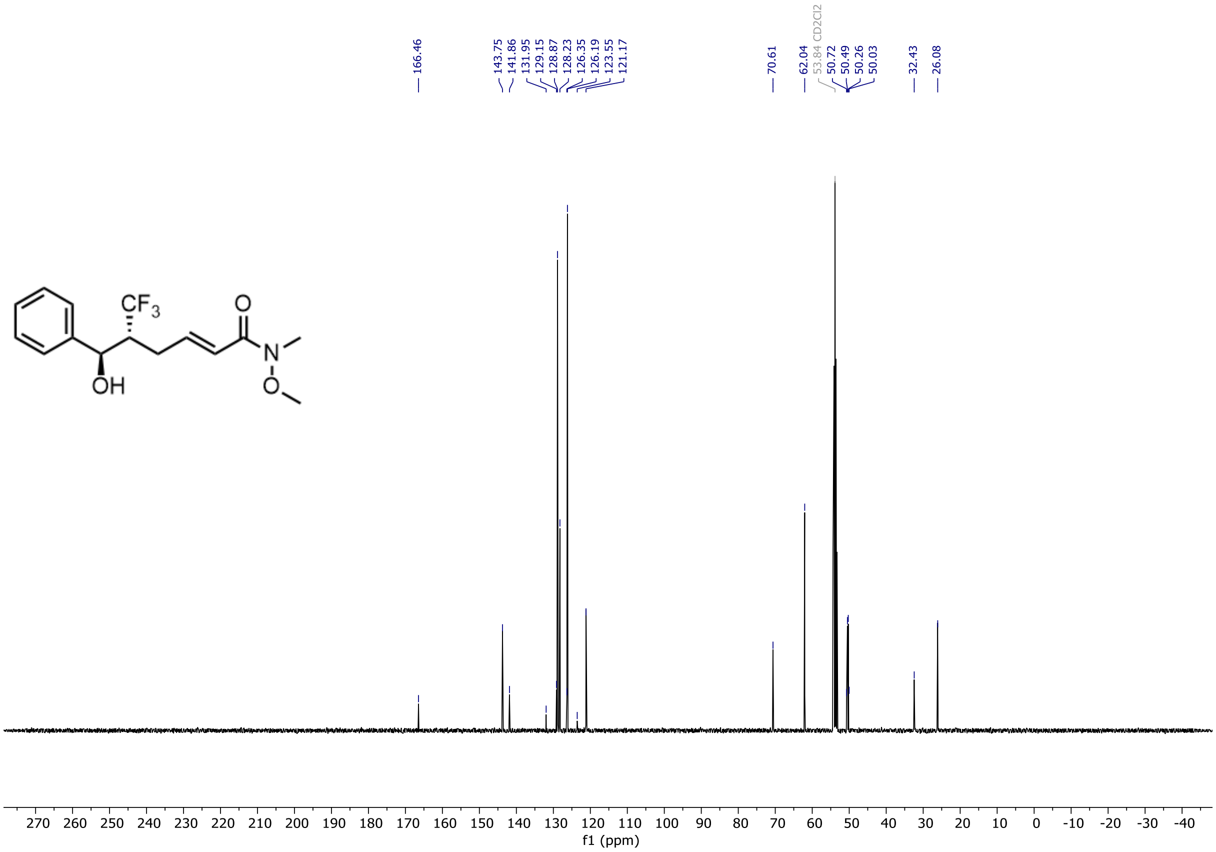

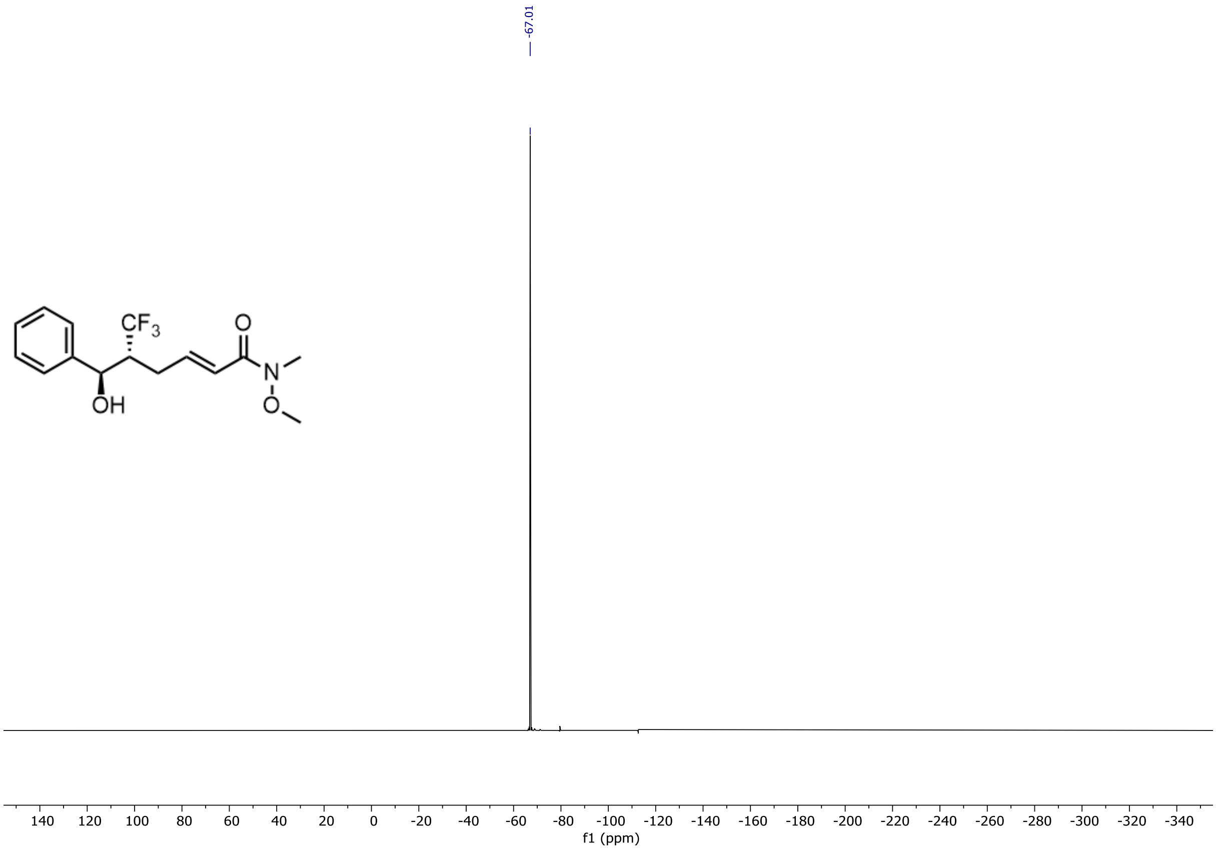
-67.01
CF3
O
N
O
OH
140 120 100 80 60 40 20 0 -20 -40 -60 -80 -100 -120 -140 -160 -180 -200 -220 -240 -260 -280 -300 -320 -340
f1 (ppm)

^{1}H NMR (400 MHz, CD_2Cl_2; top), ^{13}C NMR (101 MHz, CD_2Cl_2; middle) and ^{19}F NMR (282 MHz, CD_2Cl_2) of compound **17b**

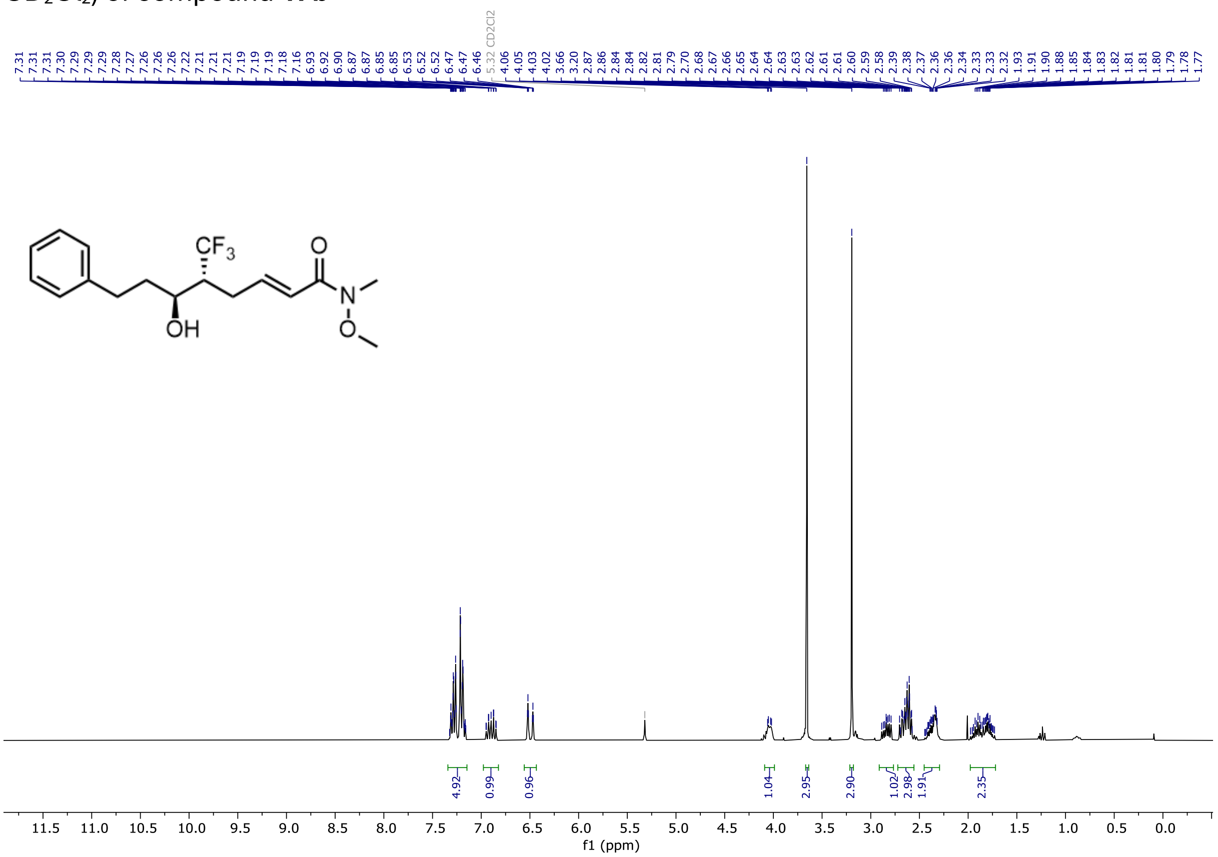

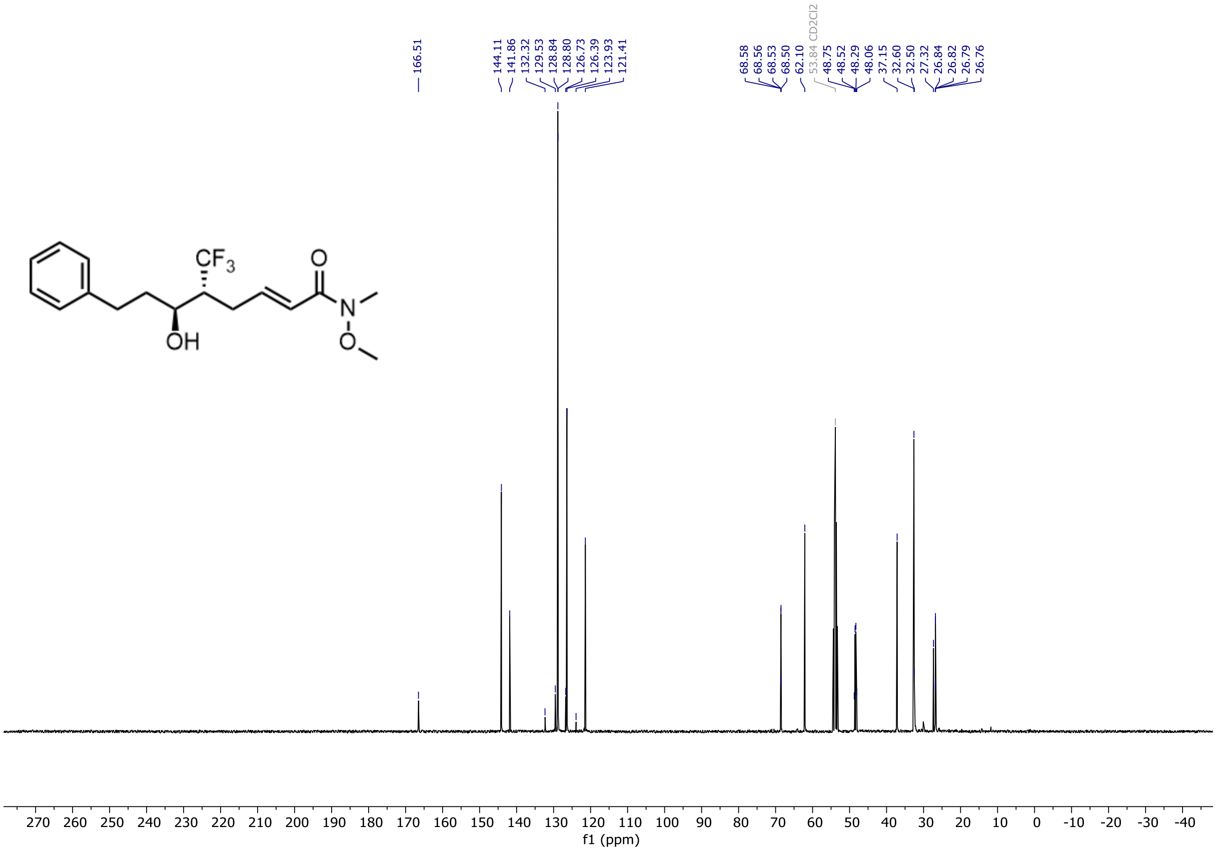

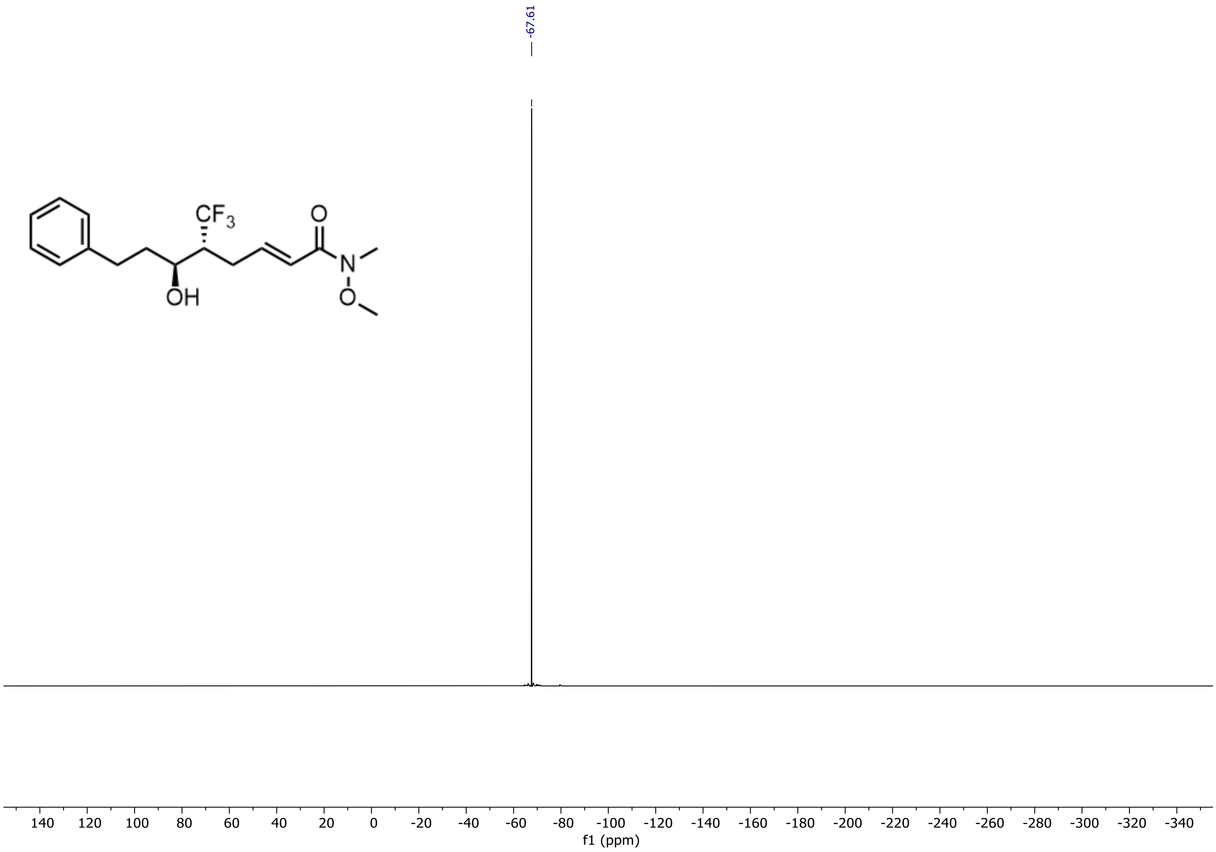
-67.61
140 120 100 80 60 40 20 0 -20 -40 -60 -80 -100 -120 -140 -160 -180 -200 -220 -240 -260 -280 -300 -320 -340
f1 (ppm)

^{1}H NMR (400 MHz, CD_2Cl_2; top), ^{13}C NMR (101 MHz, CD_2Cl_2; middle) and ^{19}F NMR (282 MHz, CD_2Cl_2) of compound **17c**

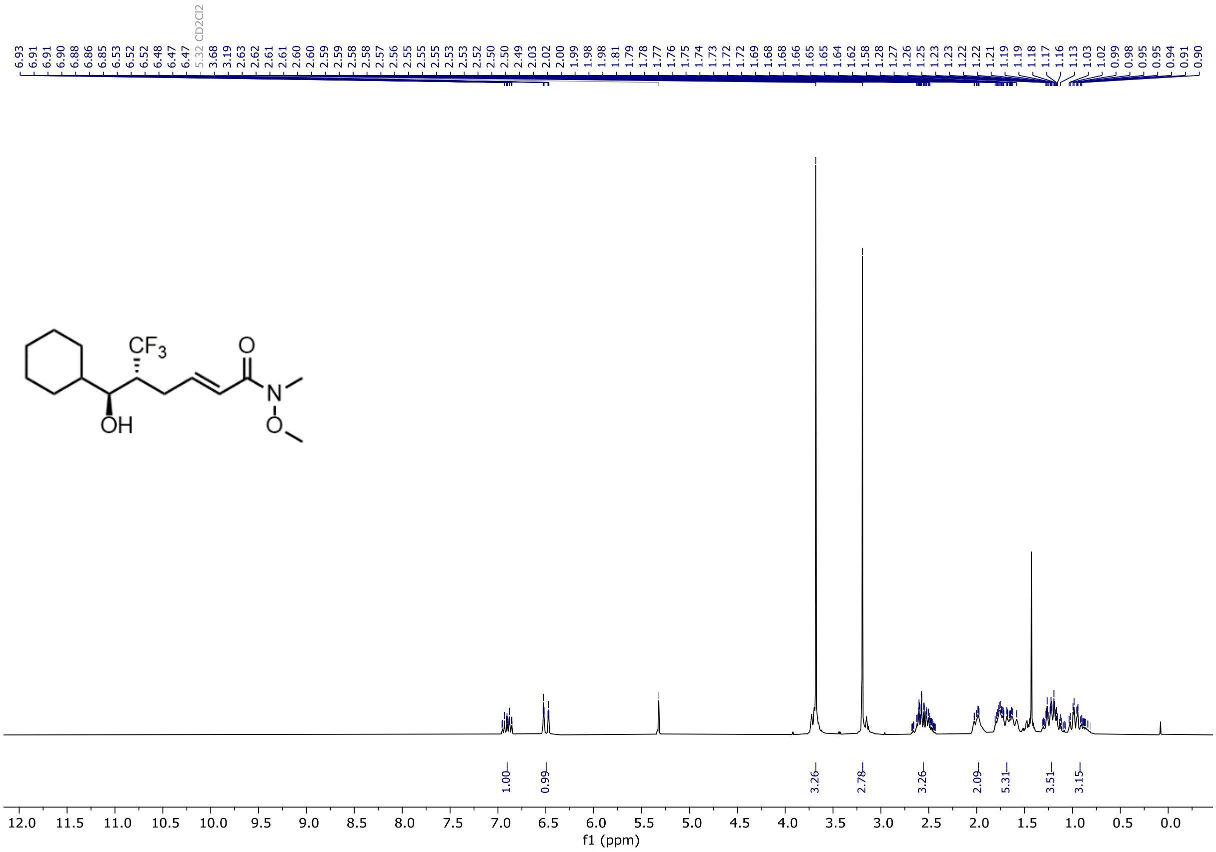

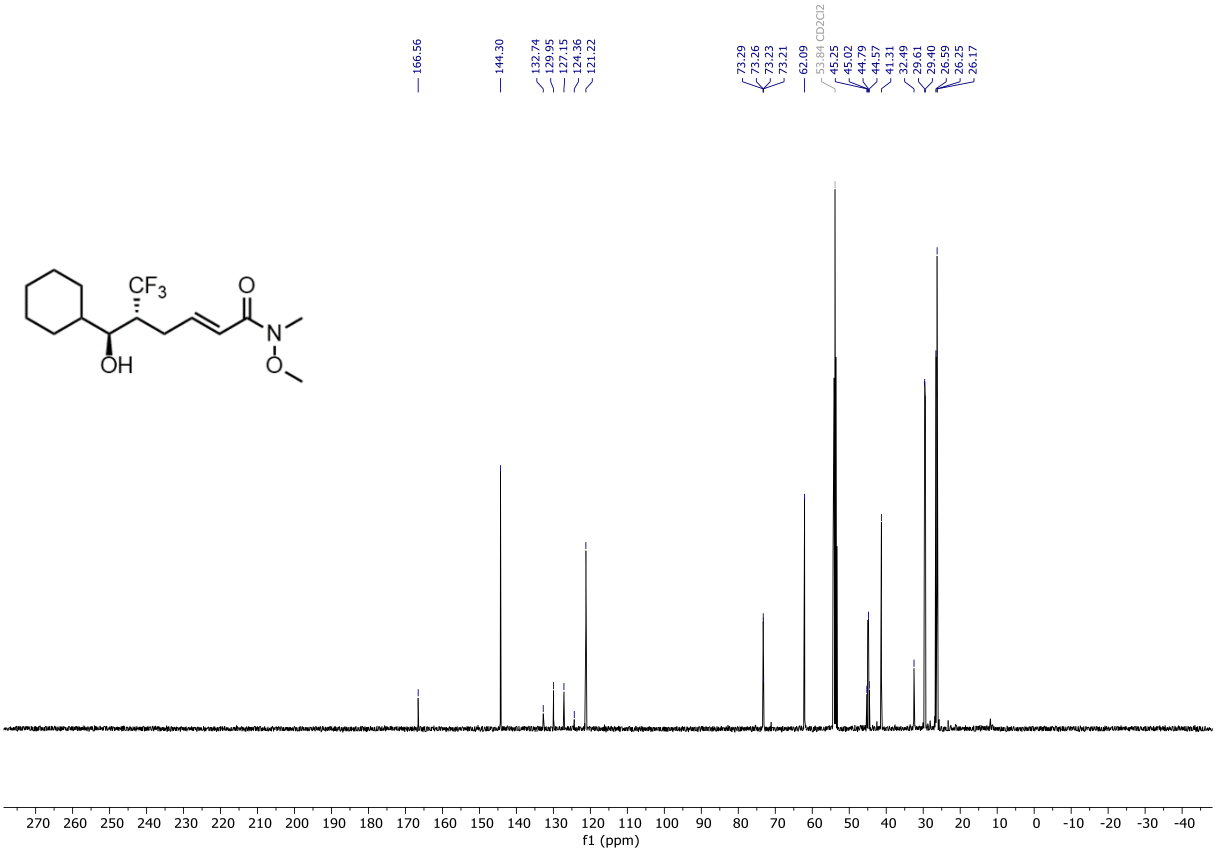

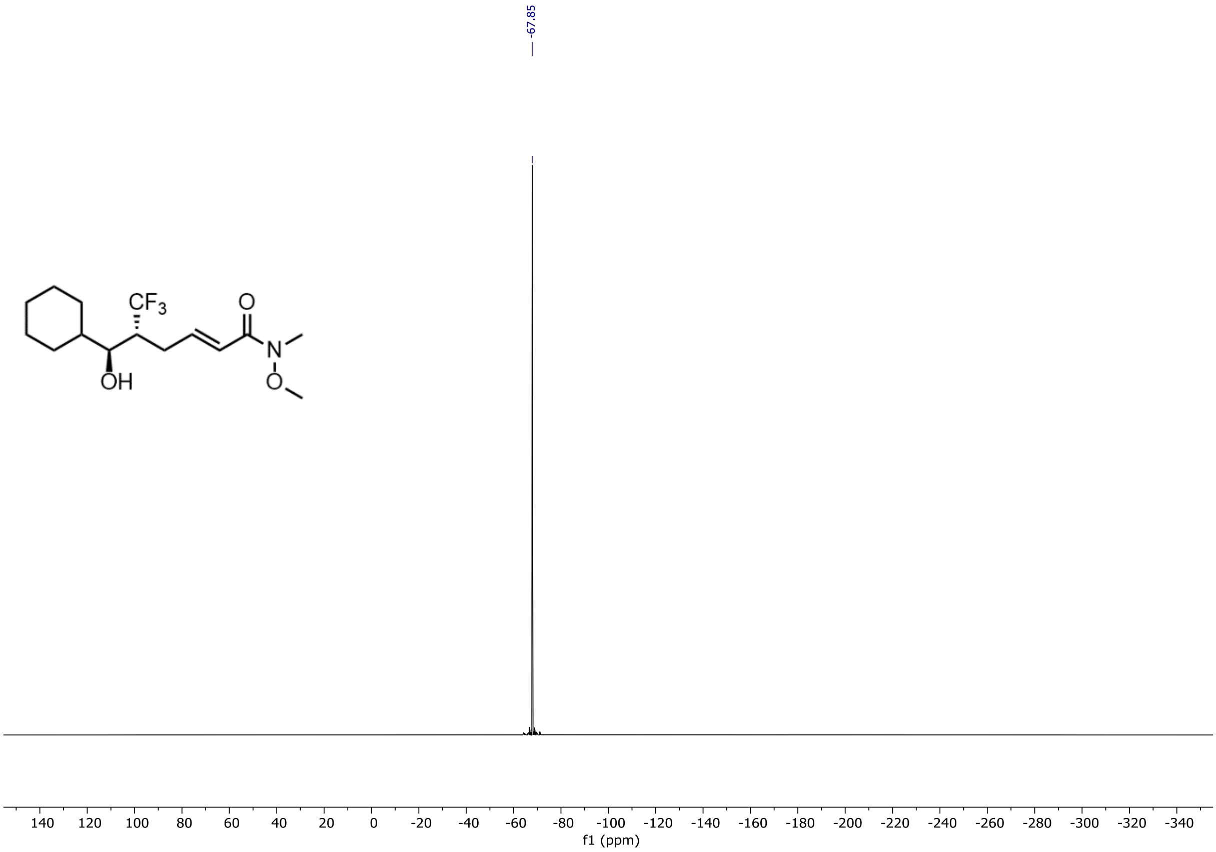
-67.85
CF3
O
N
OH
O
140 120 100 80 60 40 20 0 -20 -40 -60 -80 -100 -120 -140 -160 -180 -200 -220 -240 -260 -280 -300 -320 -340
f1 (ppm)

^{1}H NMR (400 MHz, CD_2Cl_2; top), ^{13}C NMR (101 MHz, CD_2Cl_2; middle) and ^{19}F NMR (282 MHz, CD_2Cl_2) of compound **17d**

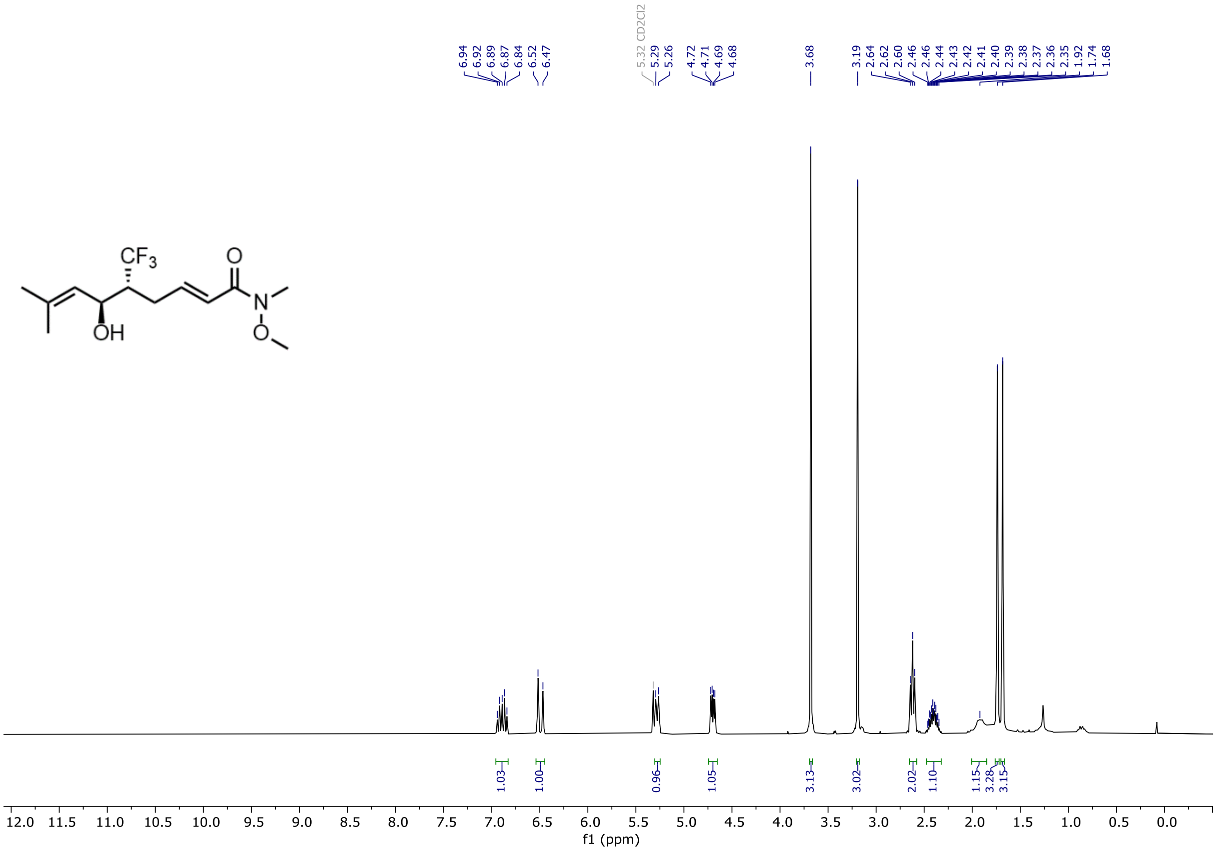

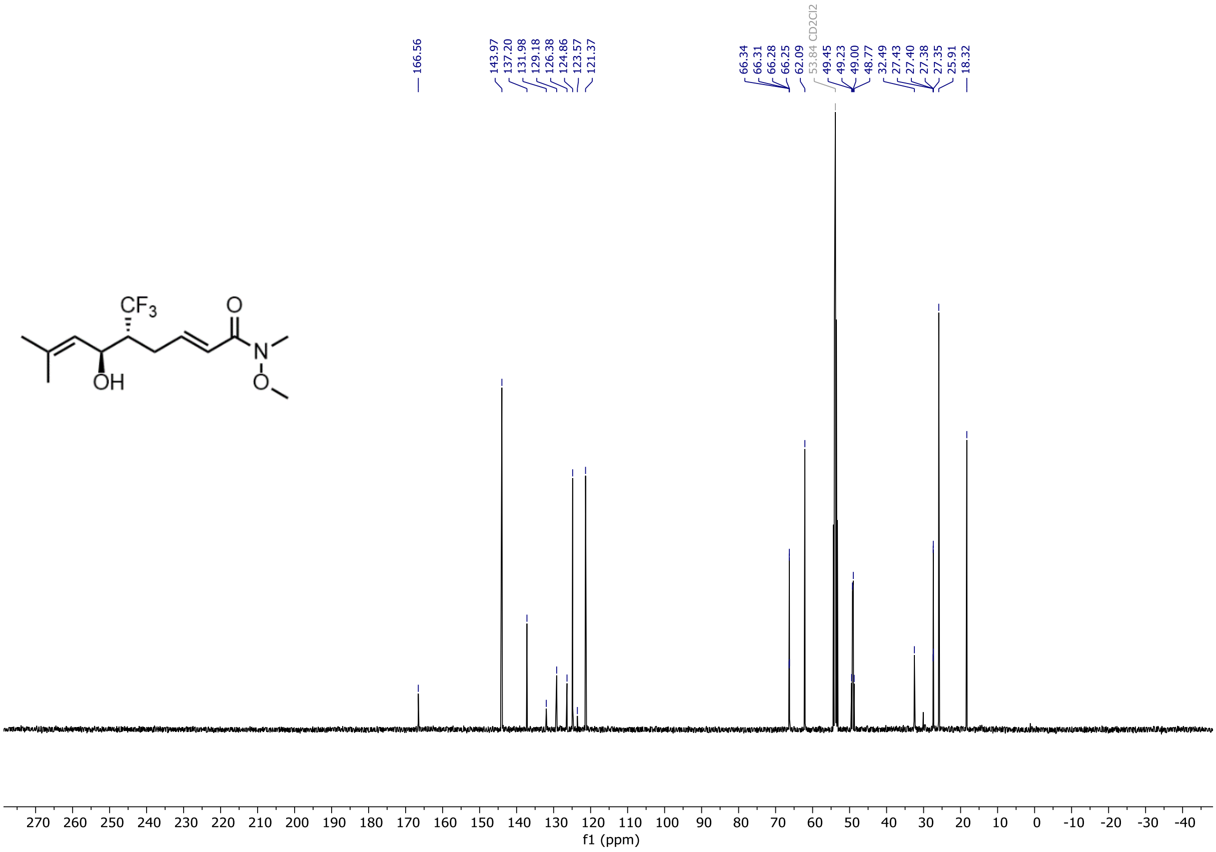

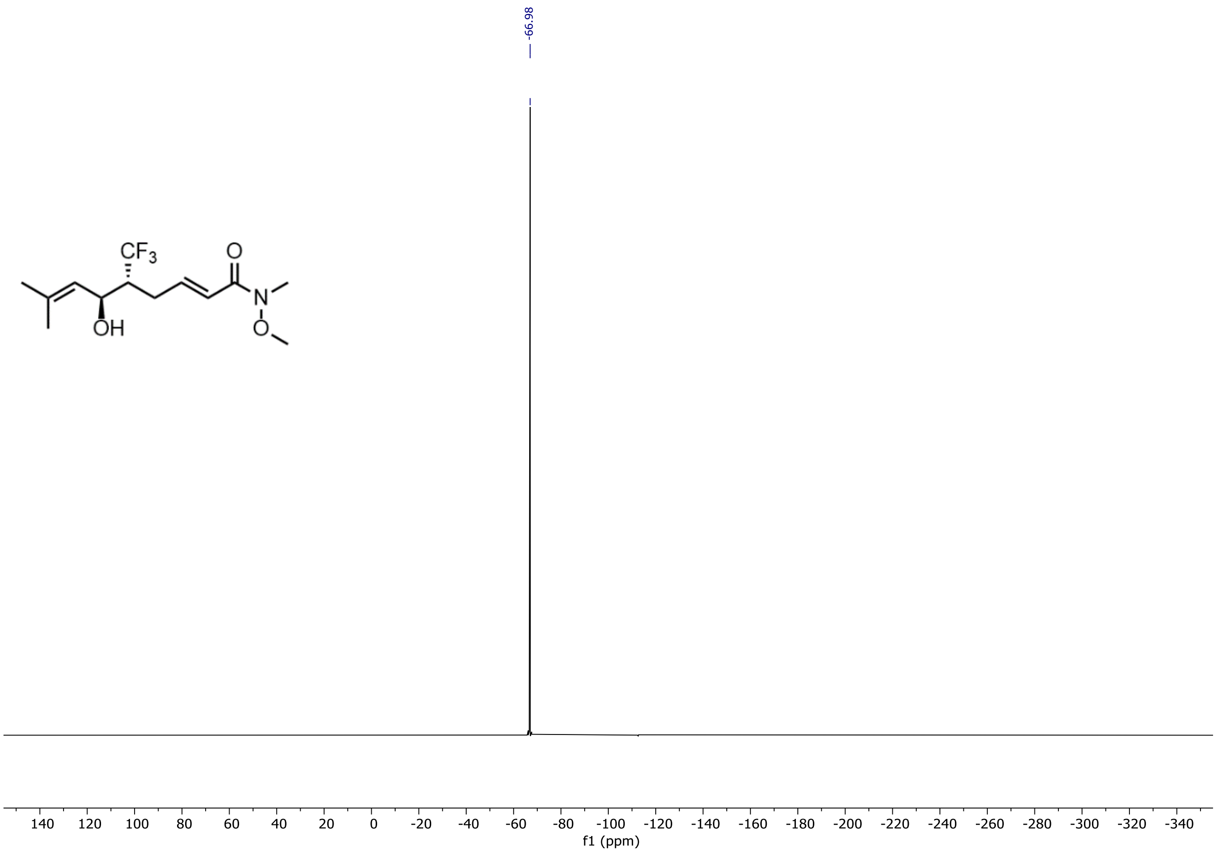
-66.98
CF3
O
N
OH
O
140 120 100 80 60 40 20 0 -20 -40 -60 -80 -100 -120 -140 -160 -180 -200 -220 -240 -260 -280 -300 -320 -340
f1 (ppm)

^{1}H NMR (400 MHz, CD_2Cl_2; top), ^{13}C NMR (101 MHz, CD_2Cl_2; middle) and ^{19}F NMR (282 MHz, CD_2Cl_2) of compound **18**

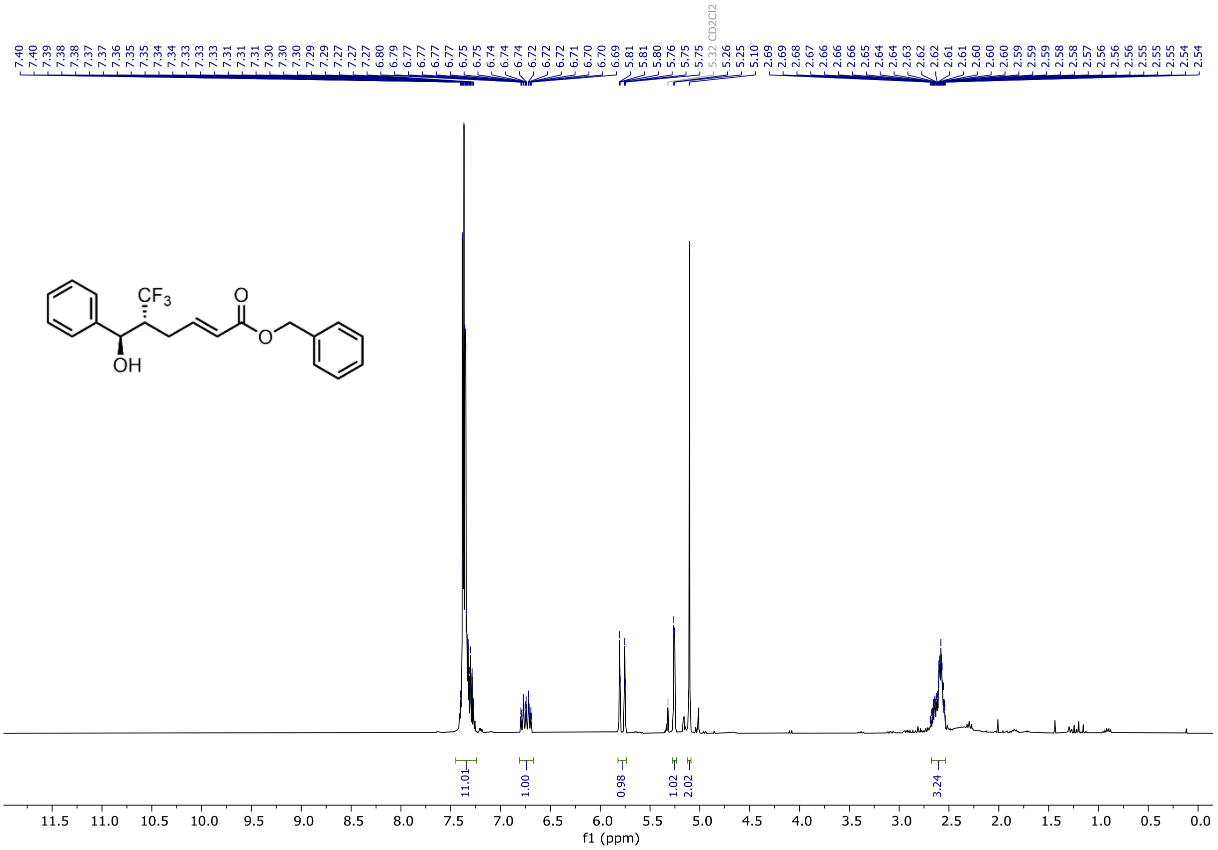

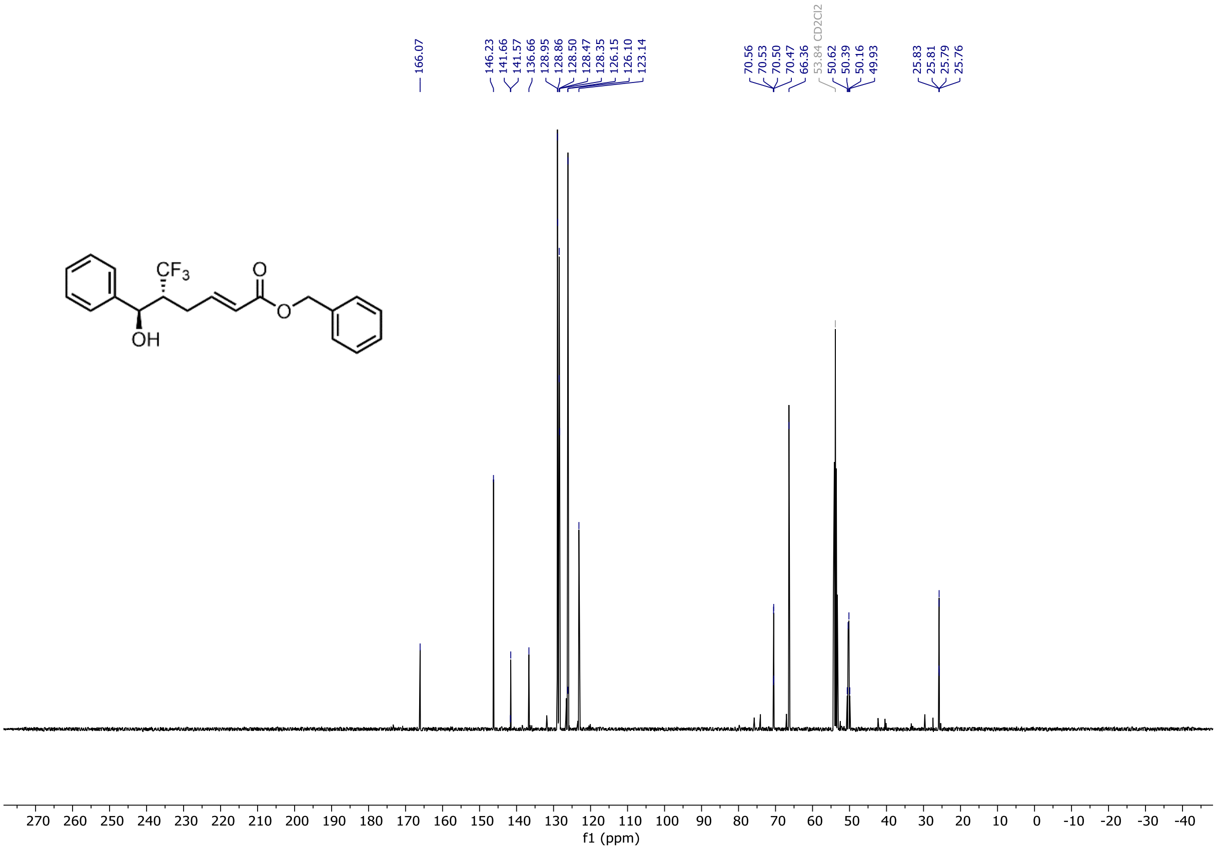

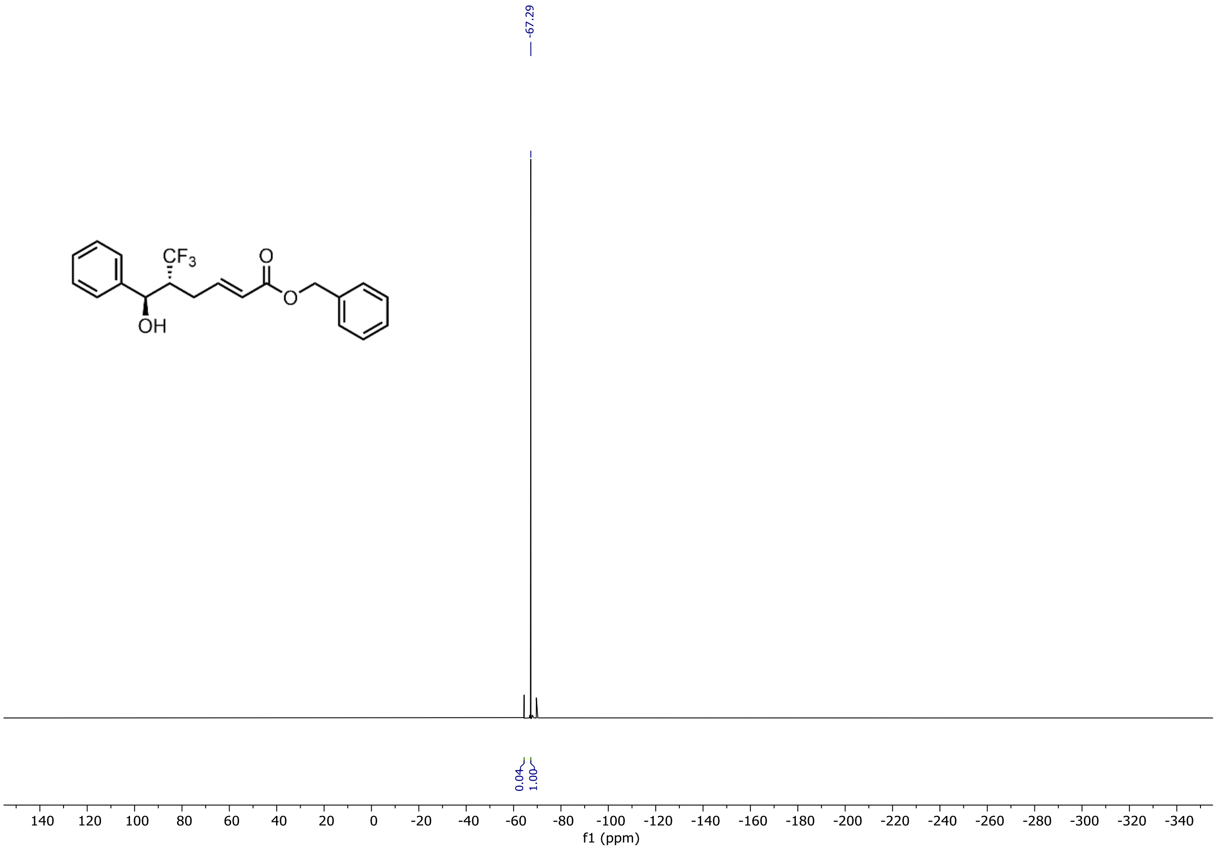

-67.29
CF3
O
O
OH
0.04
1.00
140 120 100 80 60 40 20 0 -20 -40 -60 -80 -100 -120 -140 -160 -180 -200 -220 -240 -260 -280 -300 -320 -340
f1 (ppm)

^{1}H NMR (400 MHz, CD_2Cl_2; top), ^{13}C NMR (101 MHz, CD_2Cl_2; middle) and ^{19}F NMR (282 MHz, CD_2Cl_2) of compound **19a**

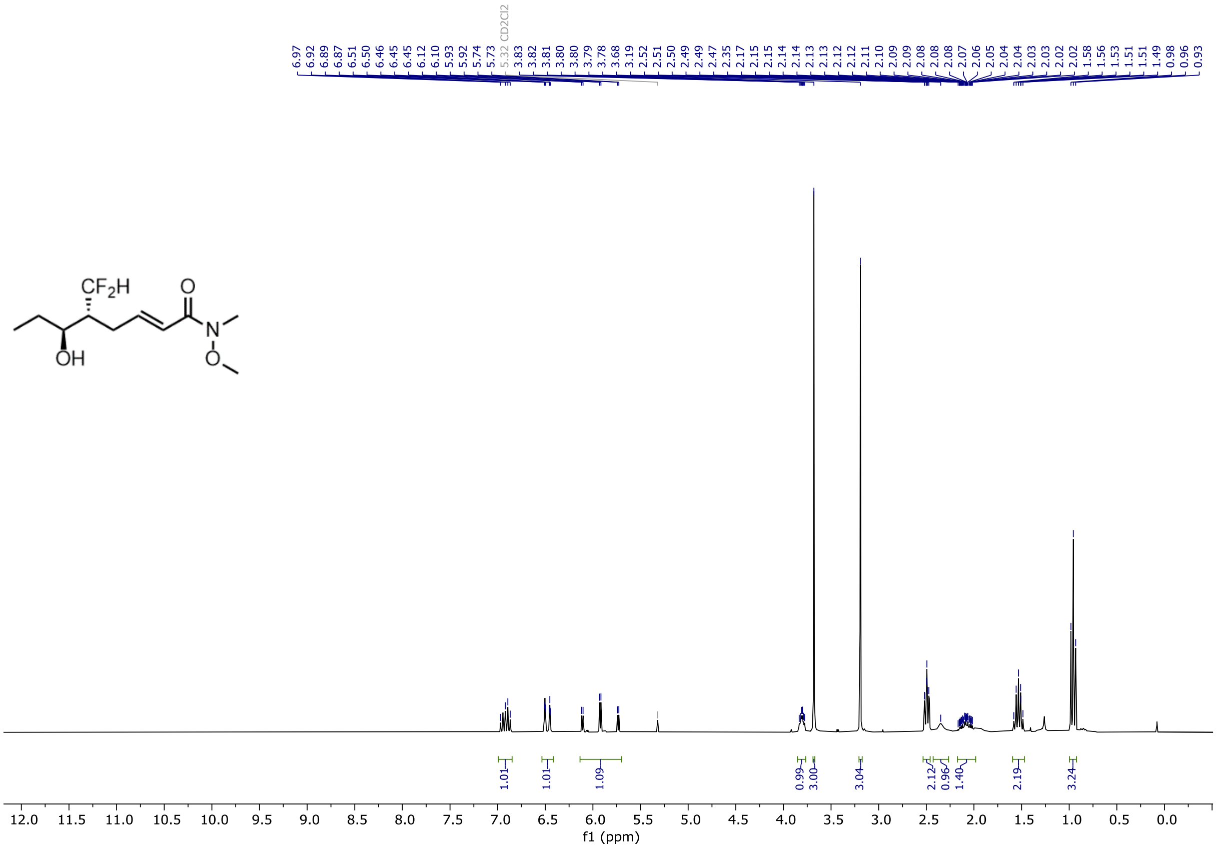

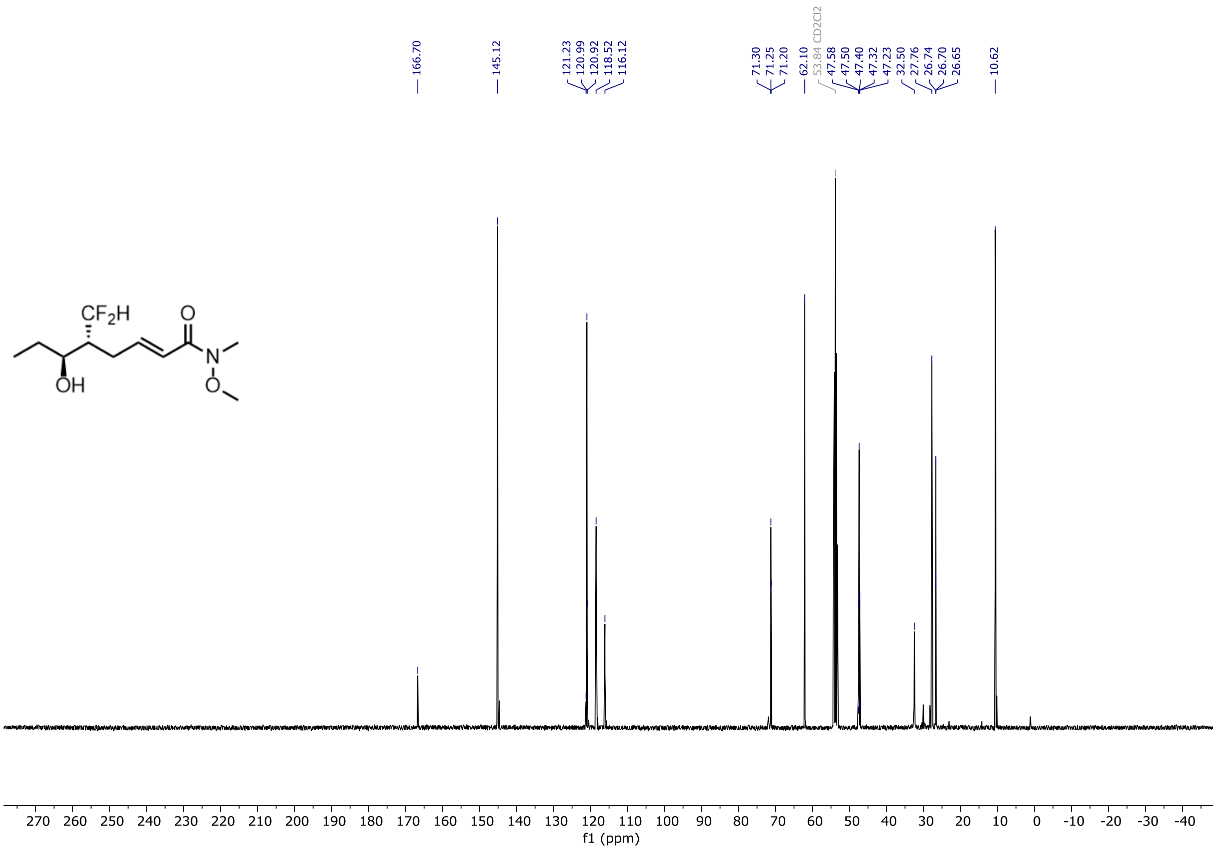

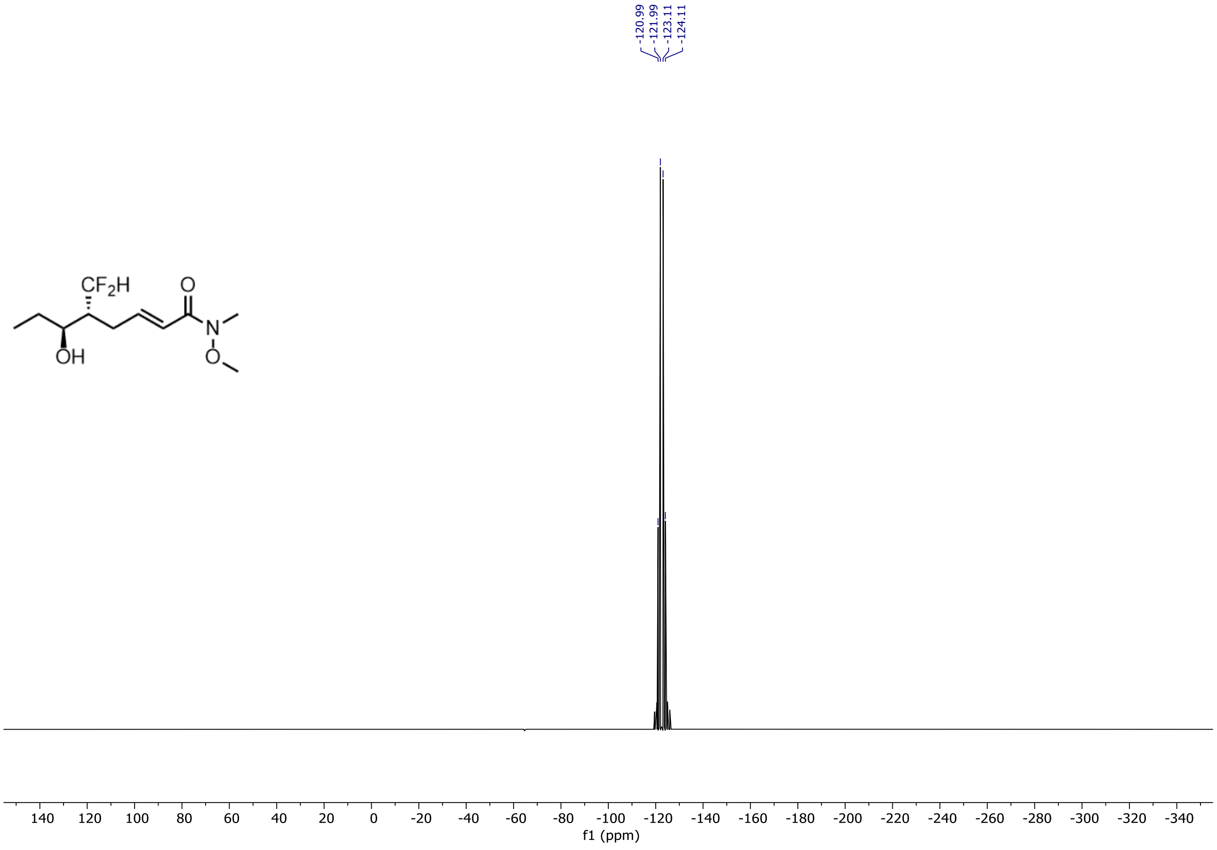
-120.99
-121.99
-123.11
-124.11
CF2H
O
N
OH
O
140 120 100 80 60 40 20 0 -20 -40 -60 -80 -100 -120 -140 -160 -180 -200 -220 -240 -260 -280 -300 -320 -340
f1 (ppm)

^{1}H NMR (400 MHz, $CDCl_3$; top), ^{13}C NMR (101 MHz, $CDCl_3$; middle) and ^{19}F NMR (282 MHz, $CDCl_3$) of compound **19b**

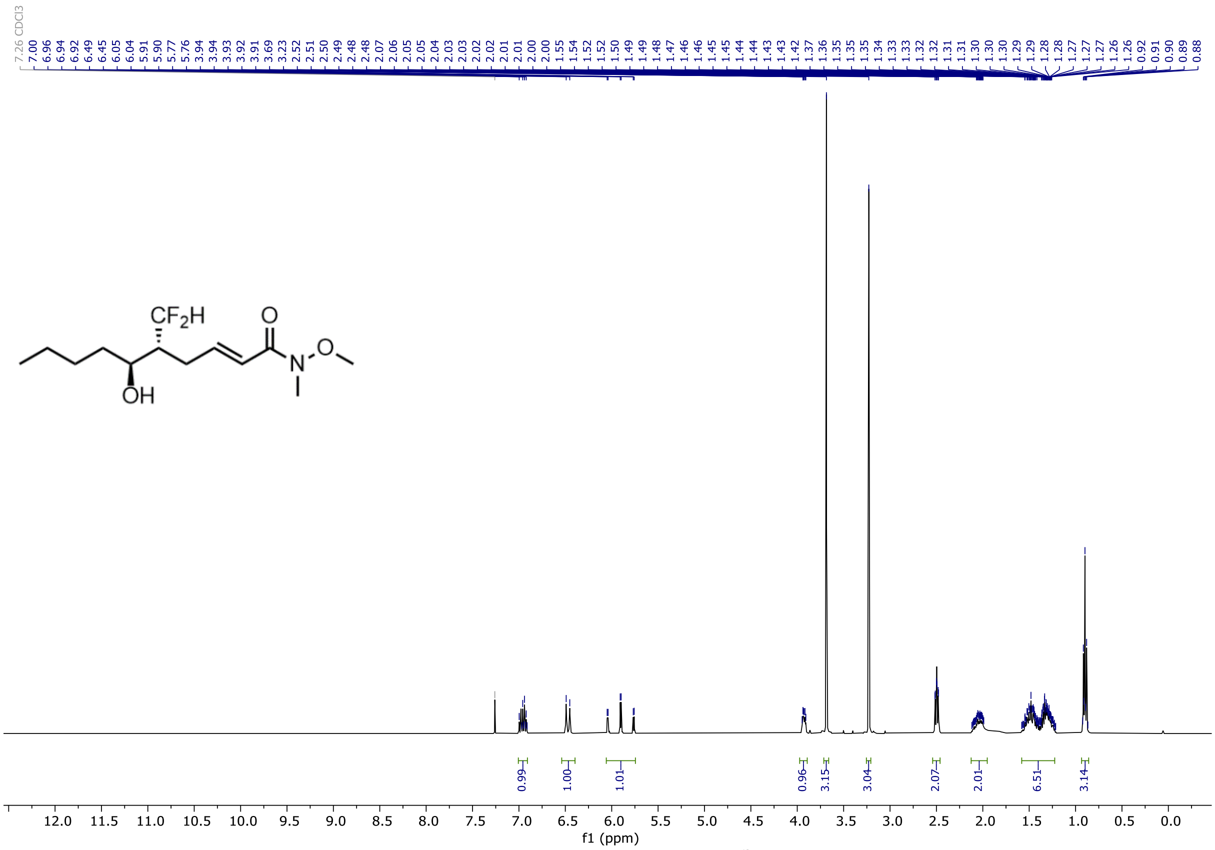

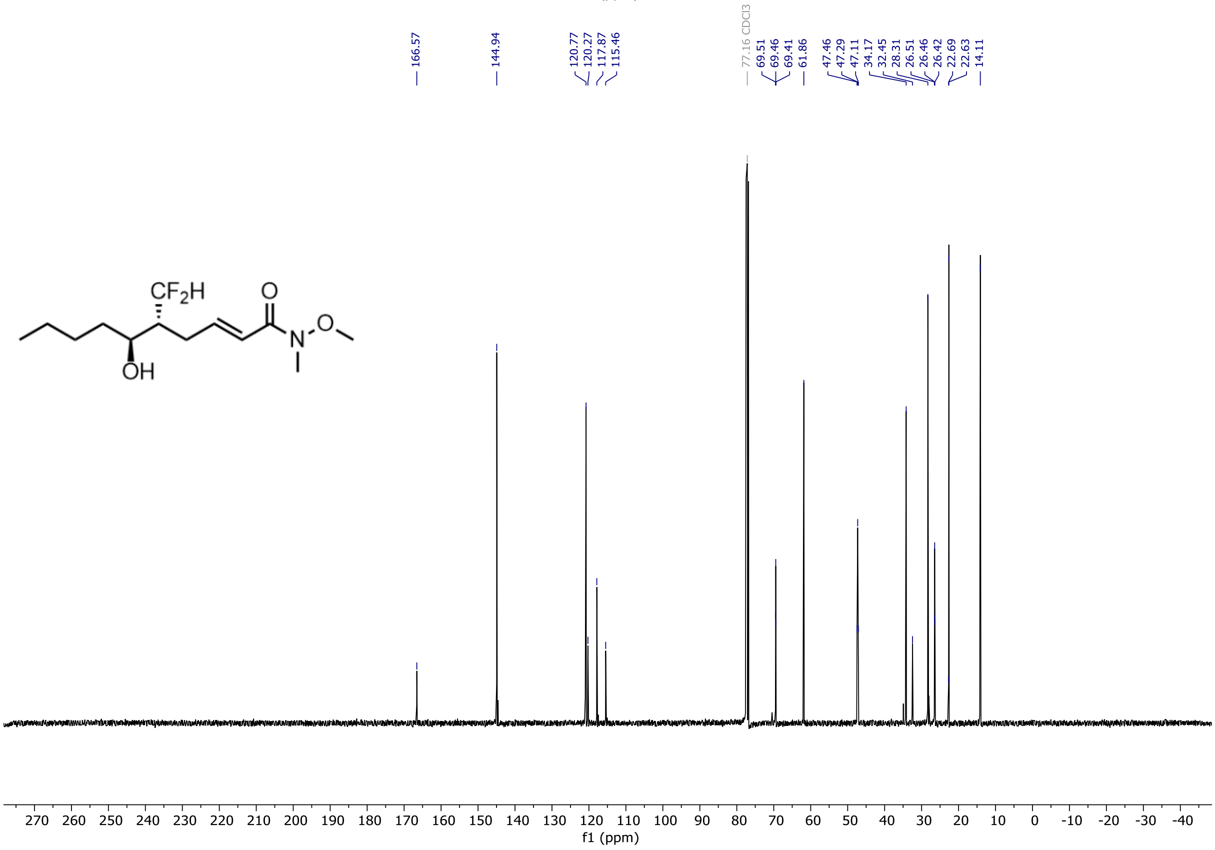

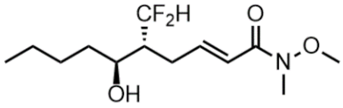

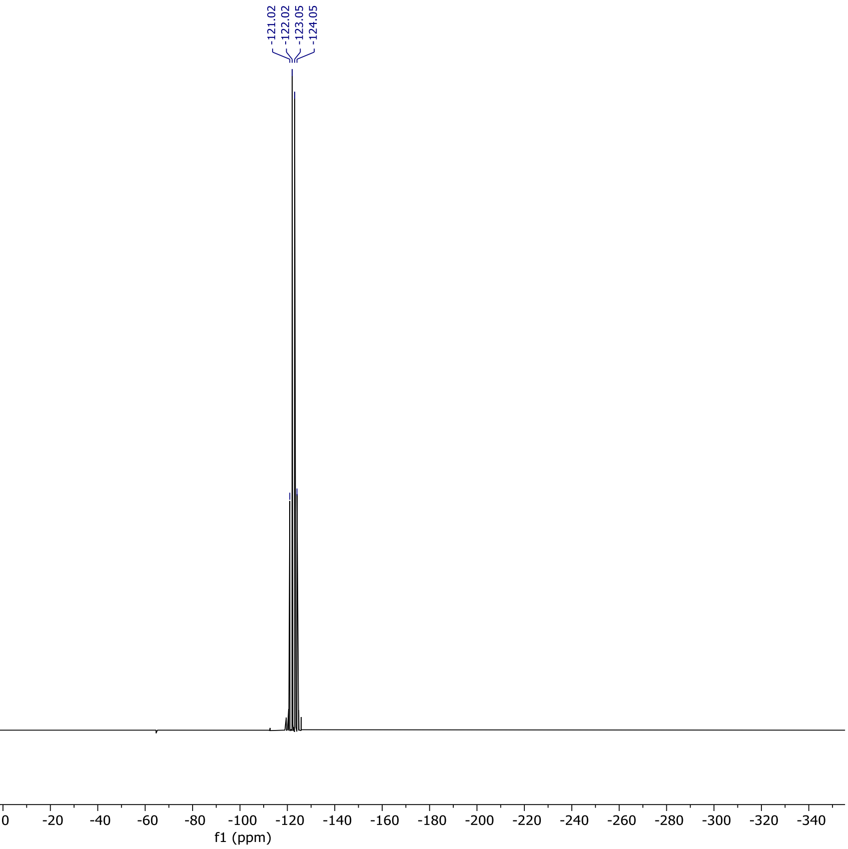
-121.02
-122.02
-123.05
-124.05
140 120 100 80 60 40 20 0 -20 -40 -60 -80 -100 -120 -140 -160 -180 -200 -220 -240 -260 -280 -300 -320 -340
f1 (ppm)

^{1}H NMR (400 MHz, CD_2Cl_2; top), ^{13}C NMR (101 MHz, CD_2Cl_2; middle) and ^{19}F NMR (282 MHz, CD_2Cl_2) of compound **19c**

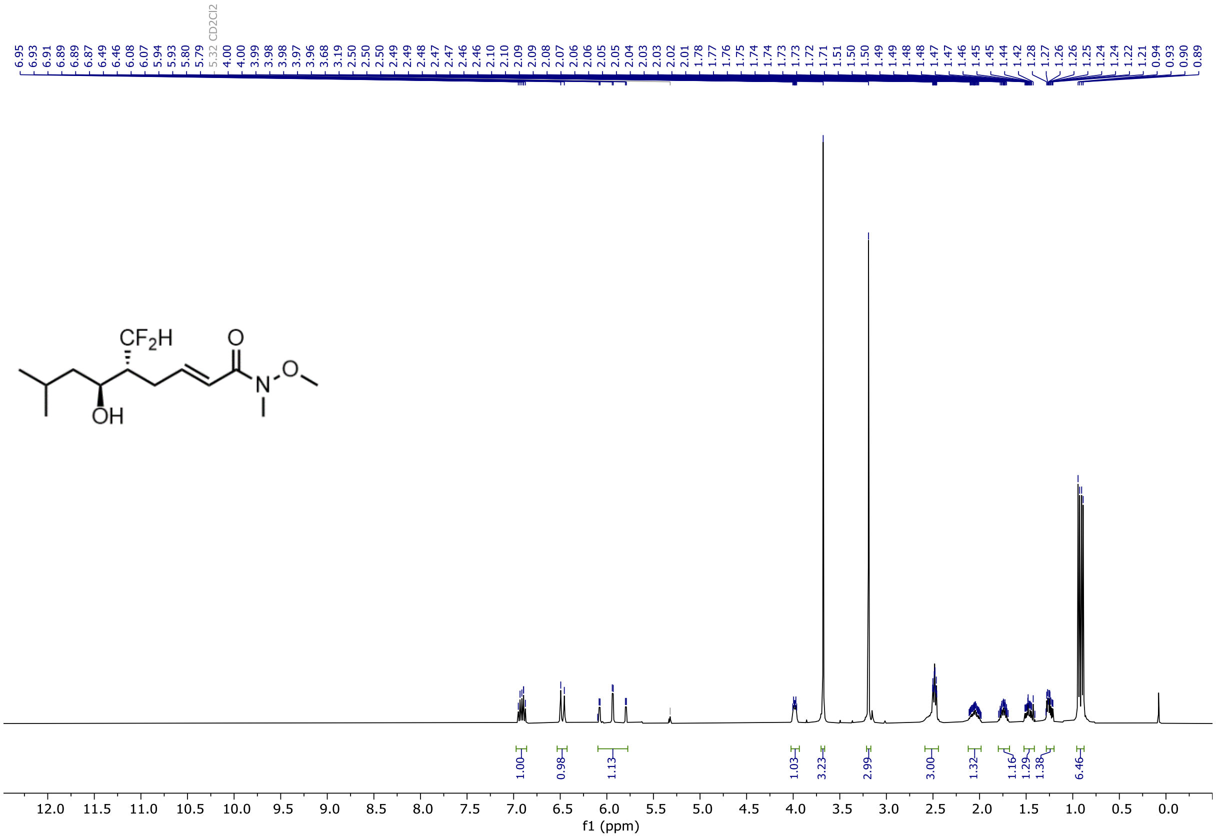

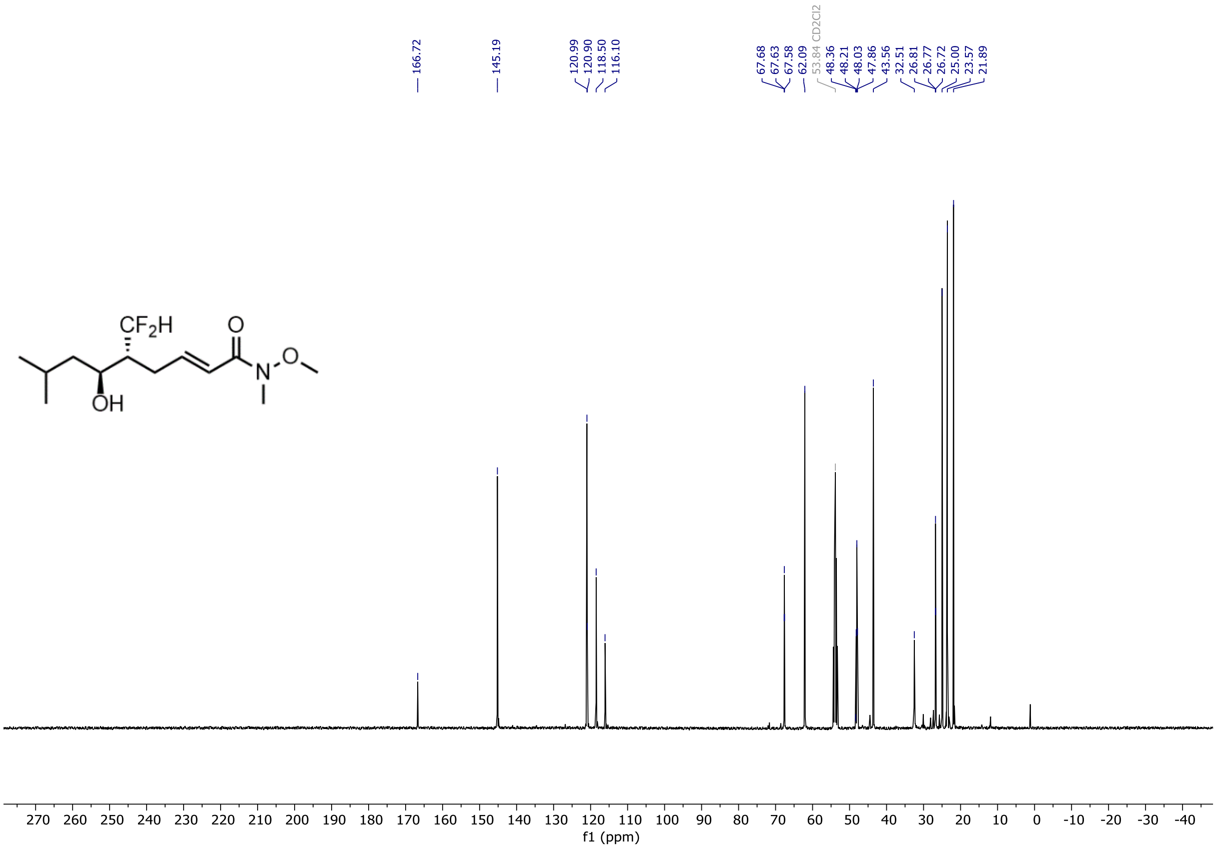

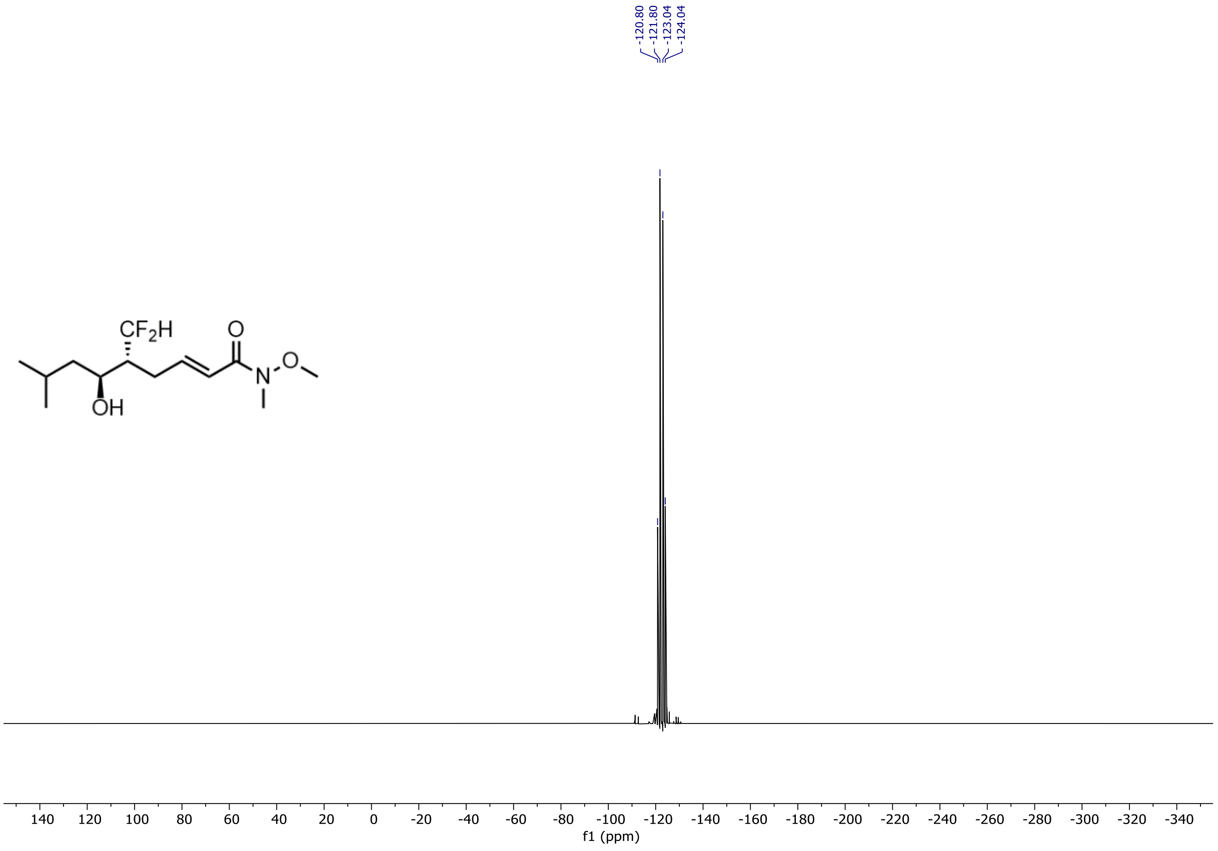
-120.80
-121.80
-123.04
-124.04
CF2H
O
N
O
OH
140 120 100 80 60 40 20 0 -20 -40 -60 -80 -100 -120 -140 -160 -180 -200 -220 -240 -260 -280 -300 -320 -340
f1 (ppm)

^{1}H NMR (400 MHz, CD_2Cl_2; top), ^{13}C NMR (101 MHz, CD_2Cl_2; middle) and ^{19}F NMR (282 MHz, CD_2Cl_2) of compound **19d**

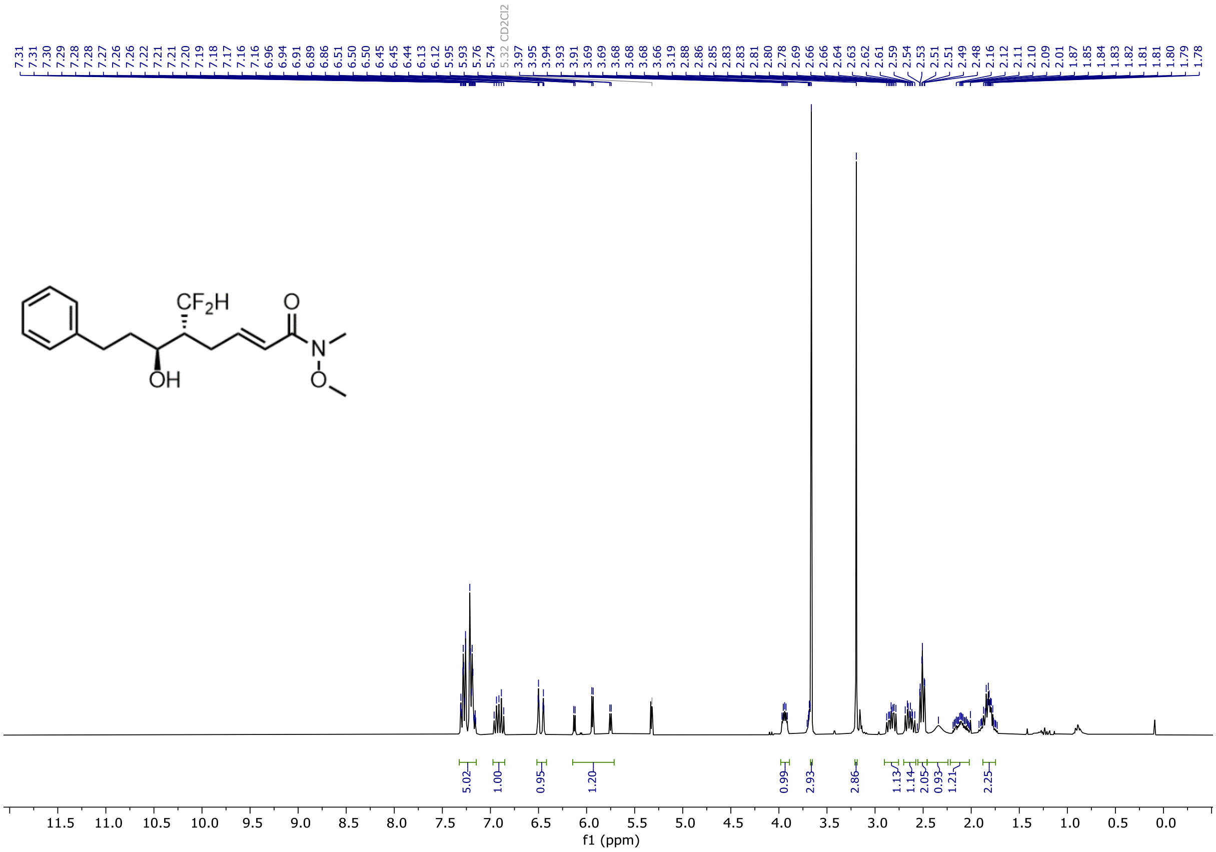

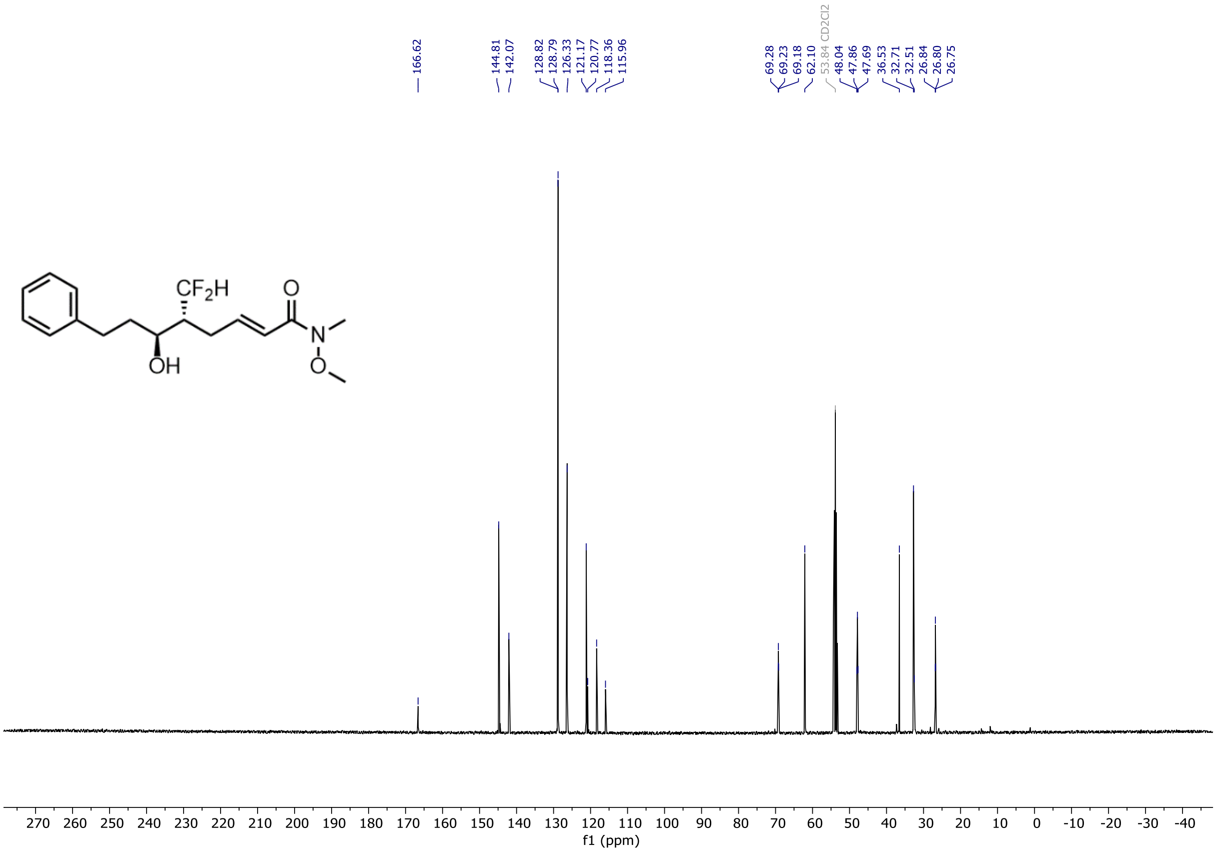

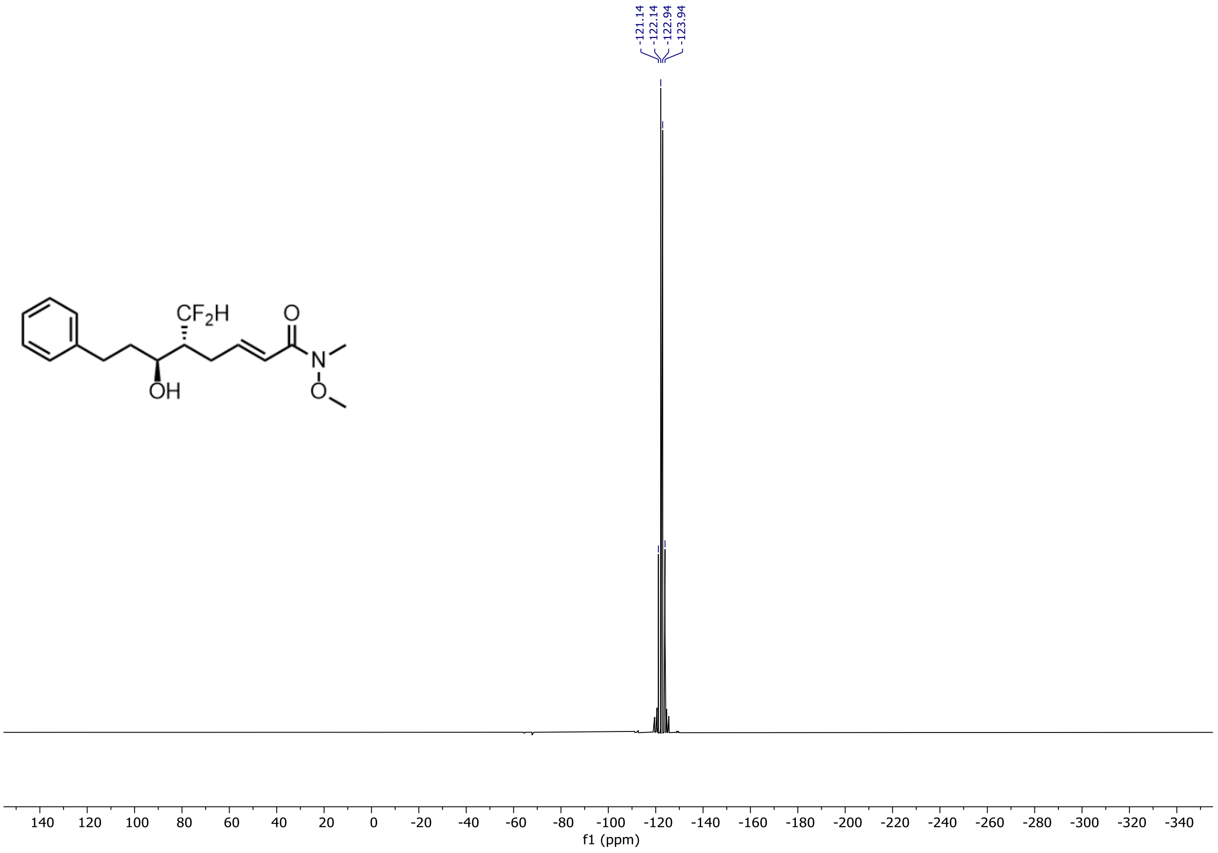
-121.14
-122.14
-122.94
-123.94
140 120 100 80 60 40 20 0 -20 -40 -60 -80 -100 -120 -140 -160 -180 -200 -220 -240 -260 -280 -300 -320 -340
f1 (ppm)

^{1}H NMR (400 MHz, CD_2Cl_2; top), ^{13}C NMR (101 MHz, CD_2Cl_2; middle) and ^{19}F NMR (282 MHz, CD_2Cl_2) of compound **19e**

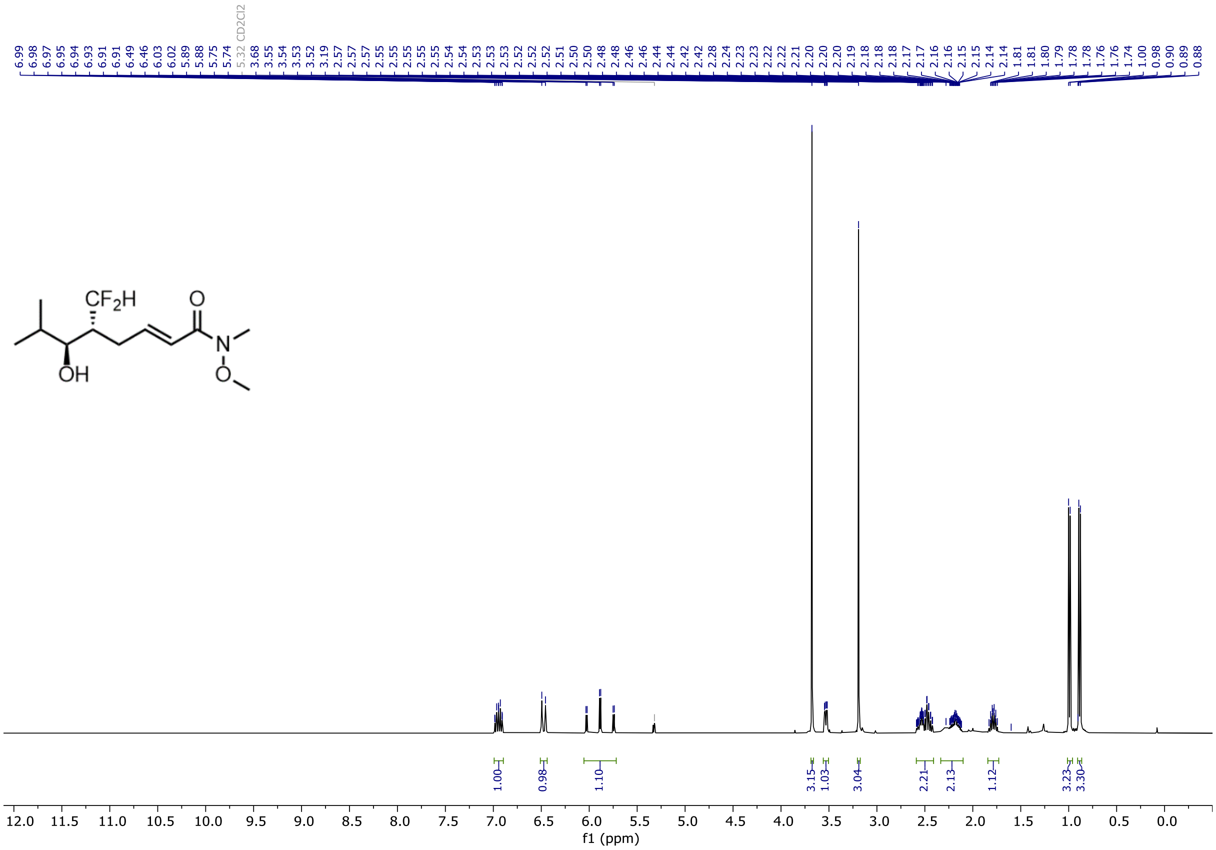

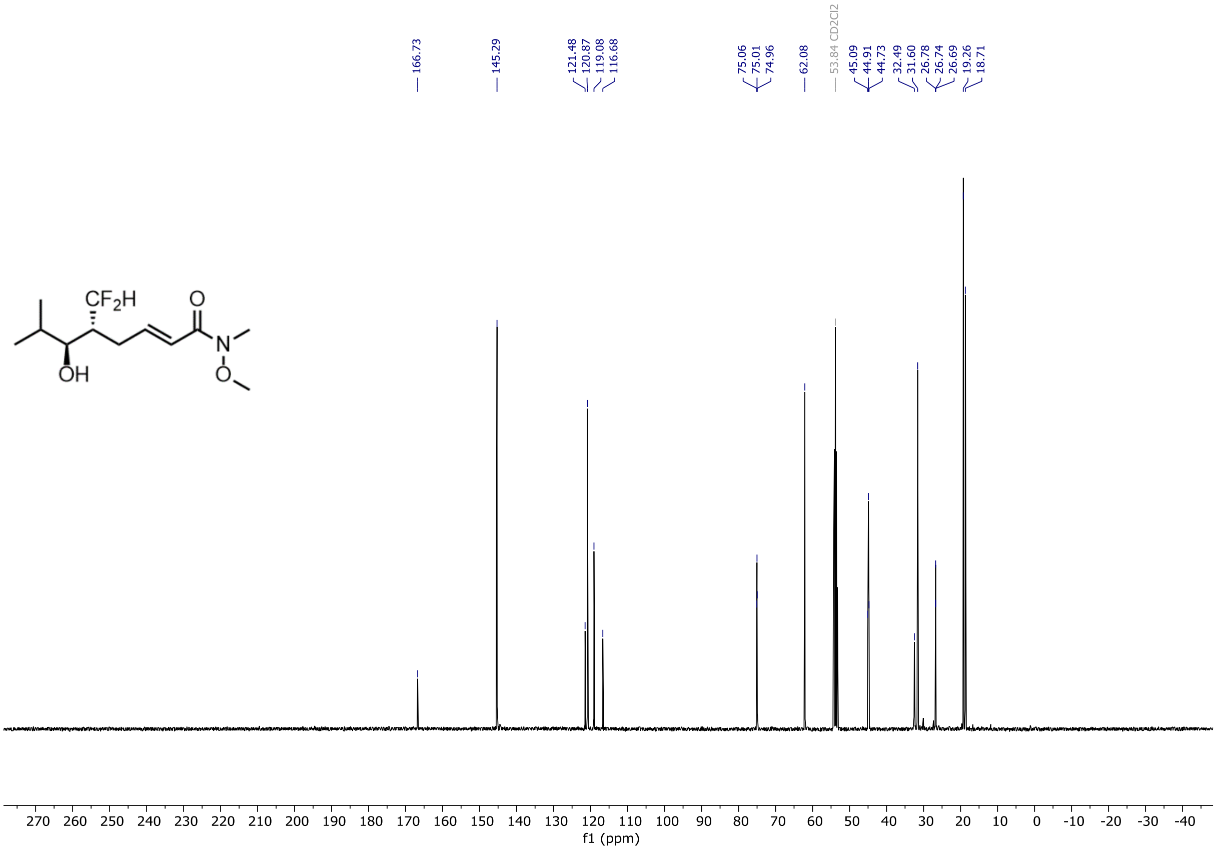

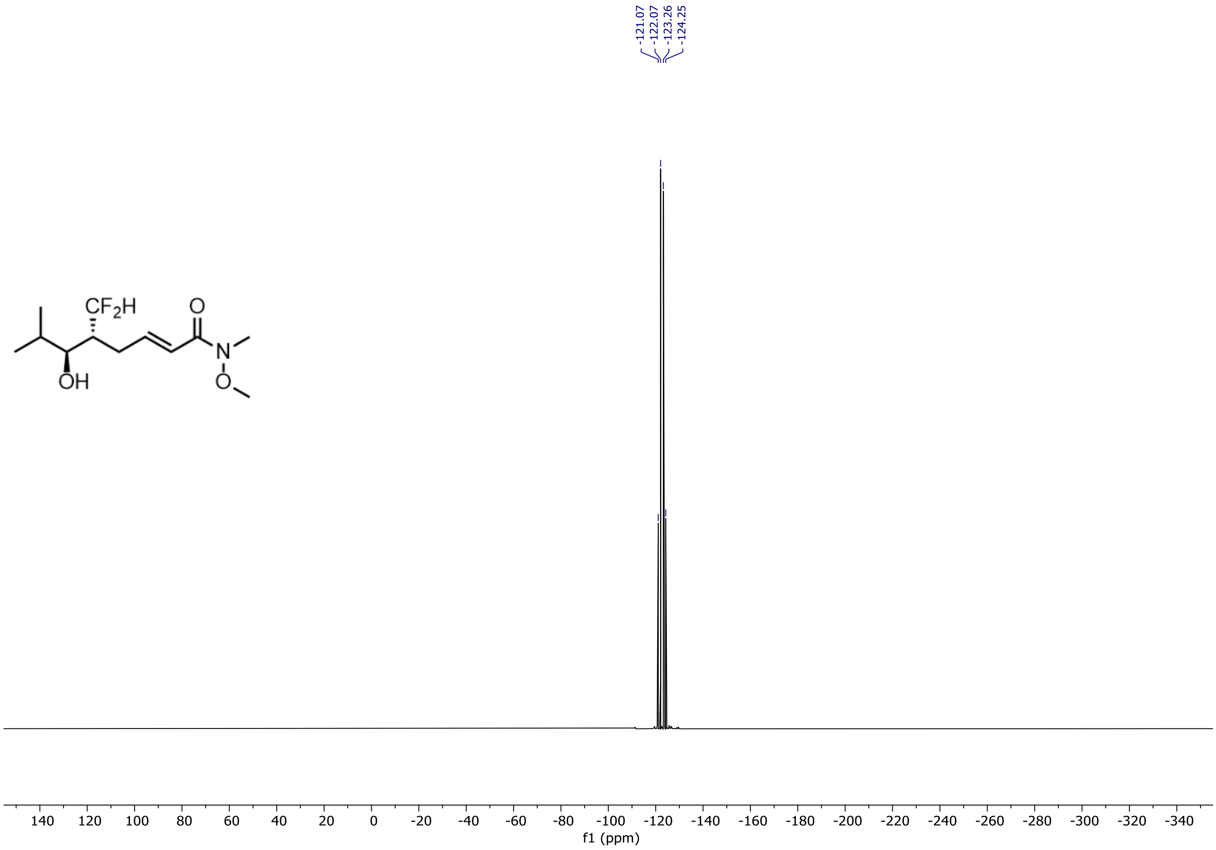
-121.07
-122.07
-123.26
-124.25
140
120
100
80
60
40
20
0
-20
-40
-60
-80
-100
-120
-140
-160
-180
-200
-220
-240
-260
-280
-300
-320
-340
f1 (ppm)

^{1}H NMR (400 MHz, CD_2Cl_2; top), ^{13}C NMR (101 MHz, CD_2Cl_2; middle) and ^{19}F NMR (282 MHz, CD_2Cl_2) of compound **19f**

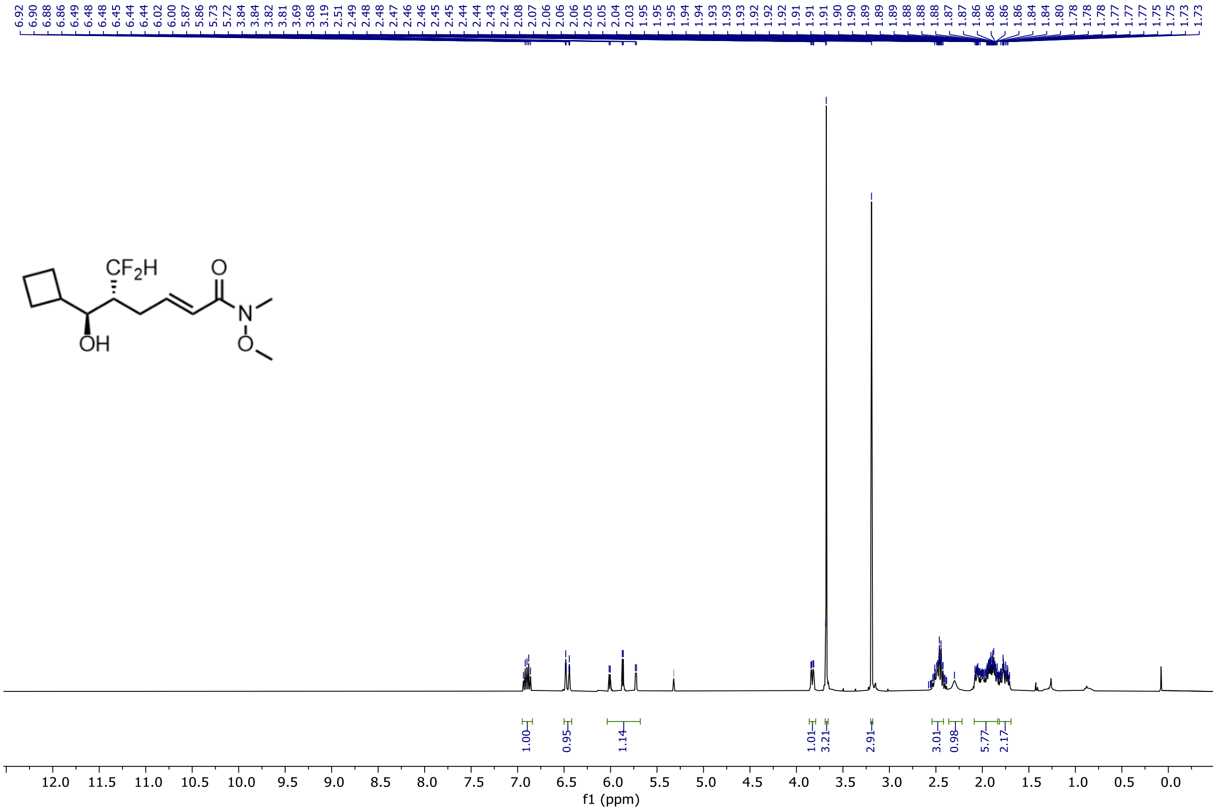

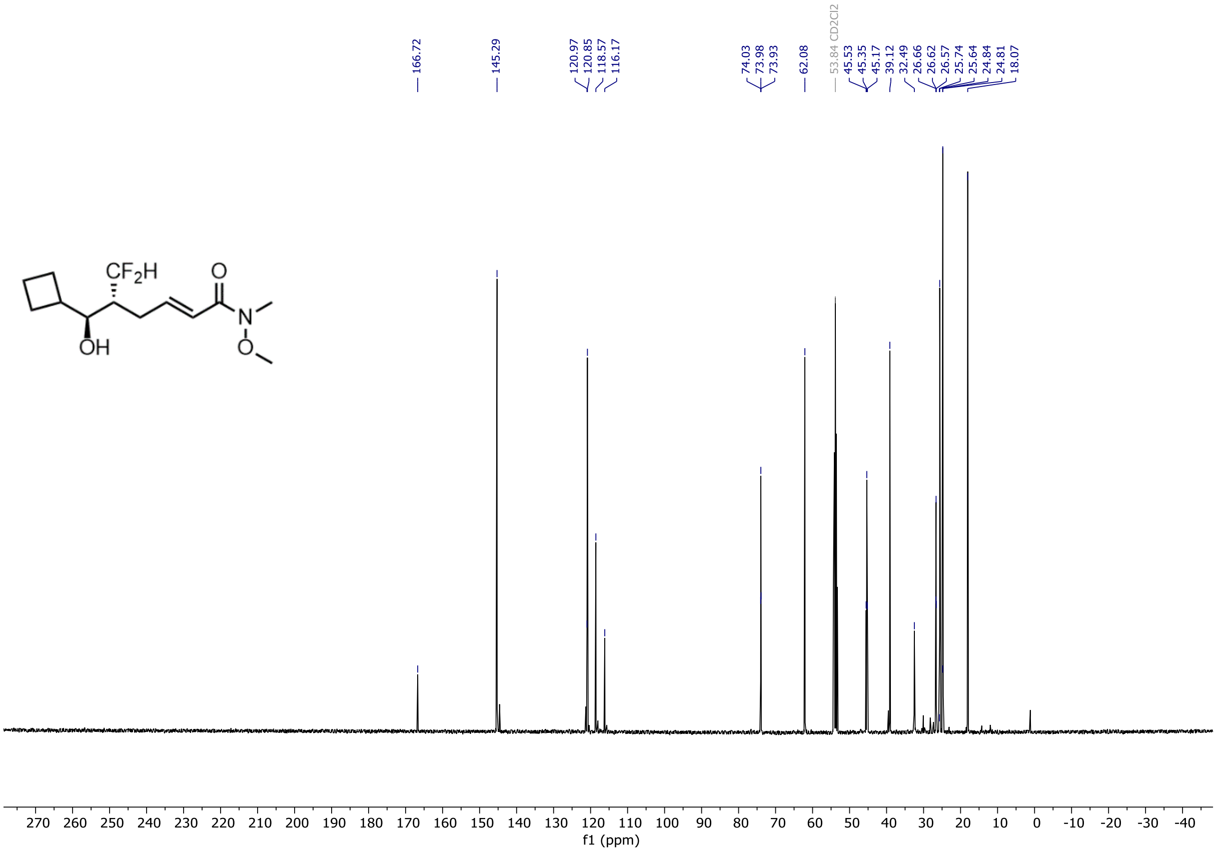

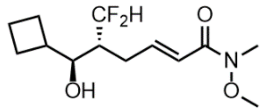
CF2H
O
N
O
OH

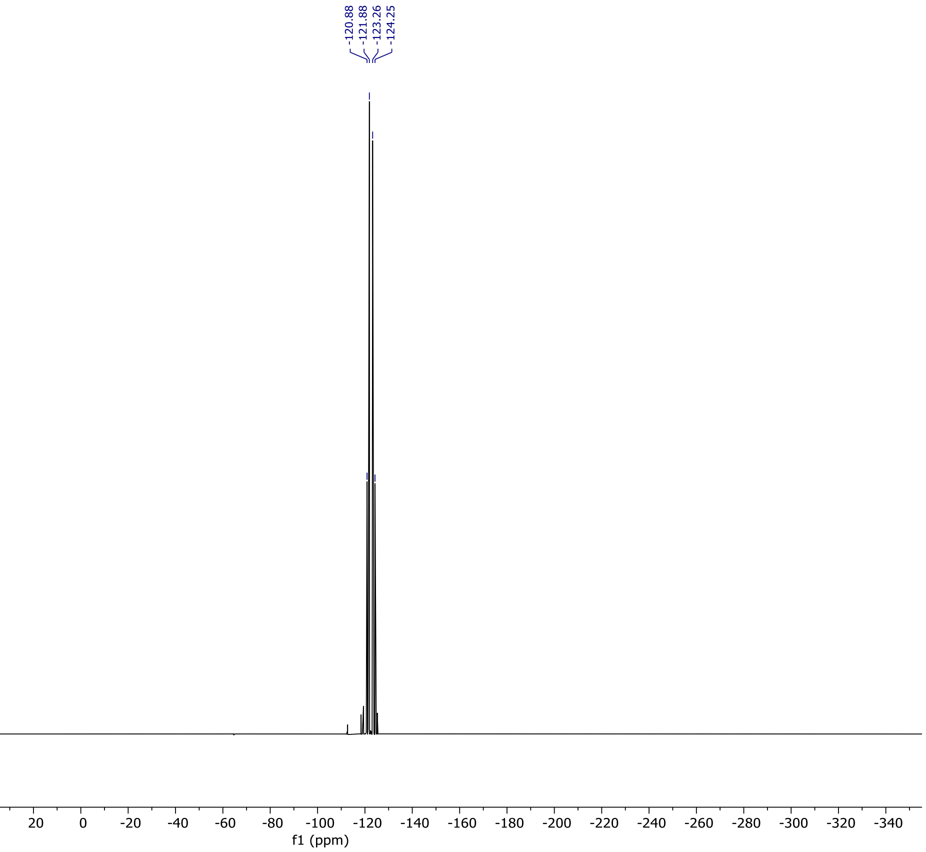
-120.88
-121.88
-123.26
-124.25
140 120 100 80 60 40 20 0 -20 -40 -60 -80 -100 -120 -140 -160 -180 -200 -220 -240 -260 -280 -300 -320 -340
f1 (ppm)

^{1}H NMR (400 MHz, CD_2Cl_2; top), ^{13}C NMR (101 MHz, CD_2Cl_2; middle) and ^{19}F NMR (282 MHz, CD_2Cl_2) of compound **19g**

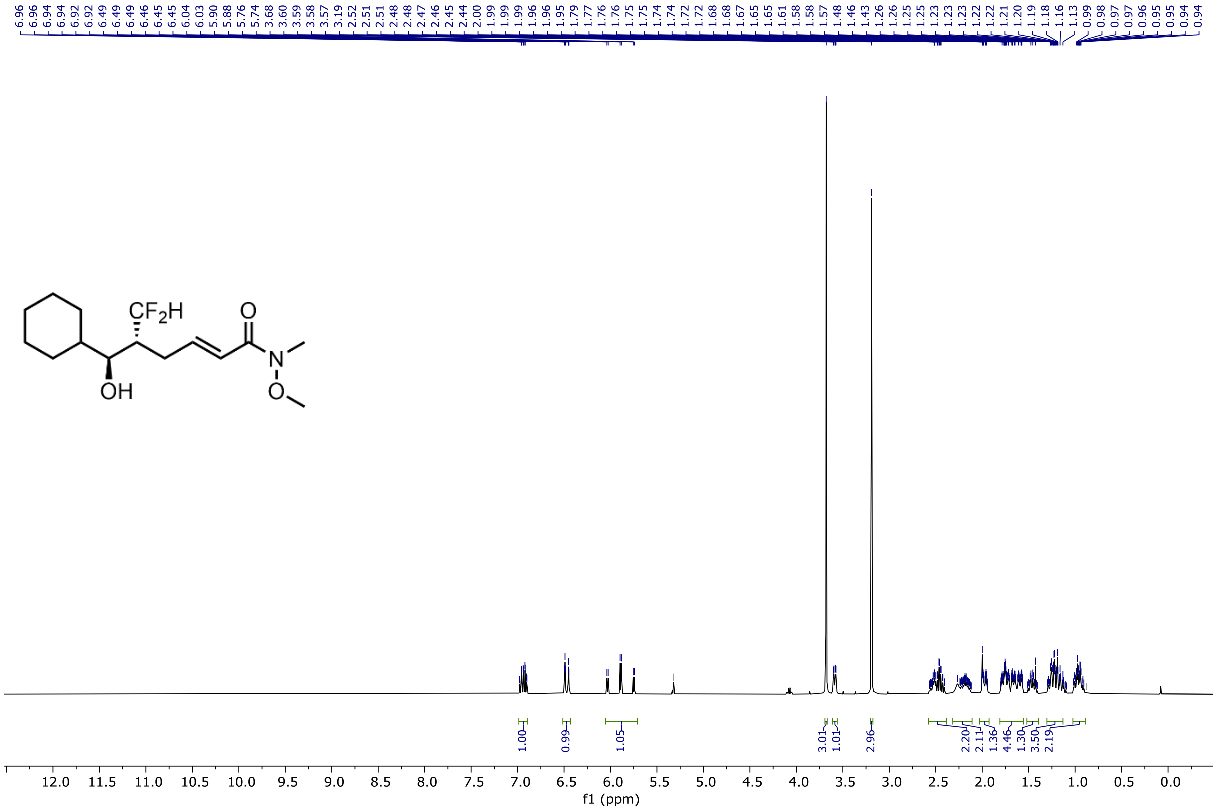

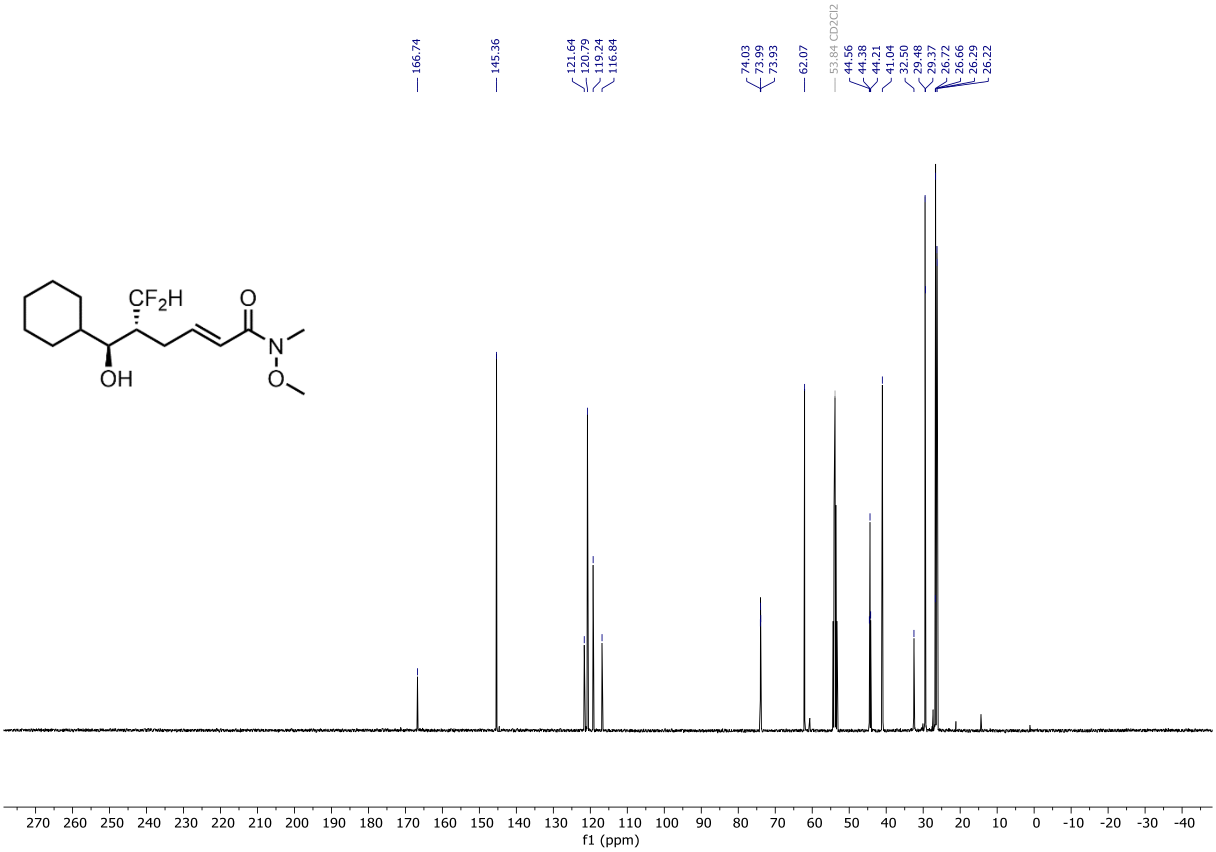

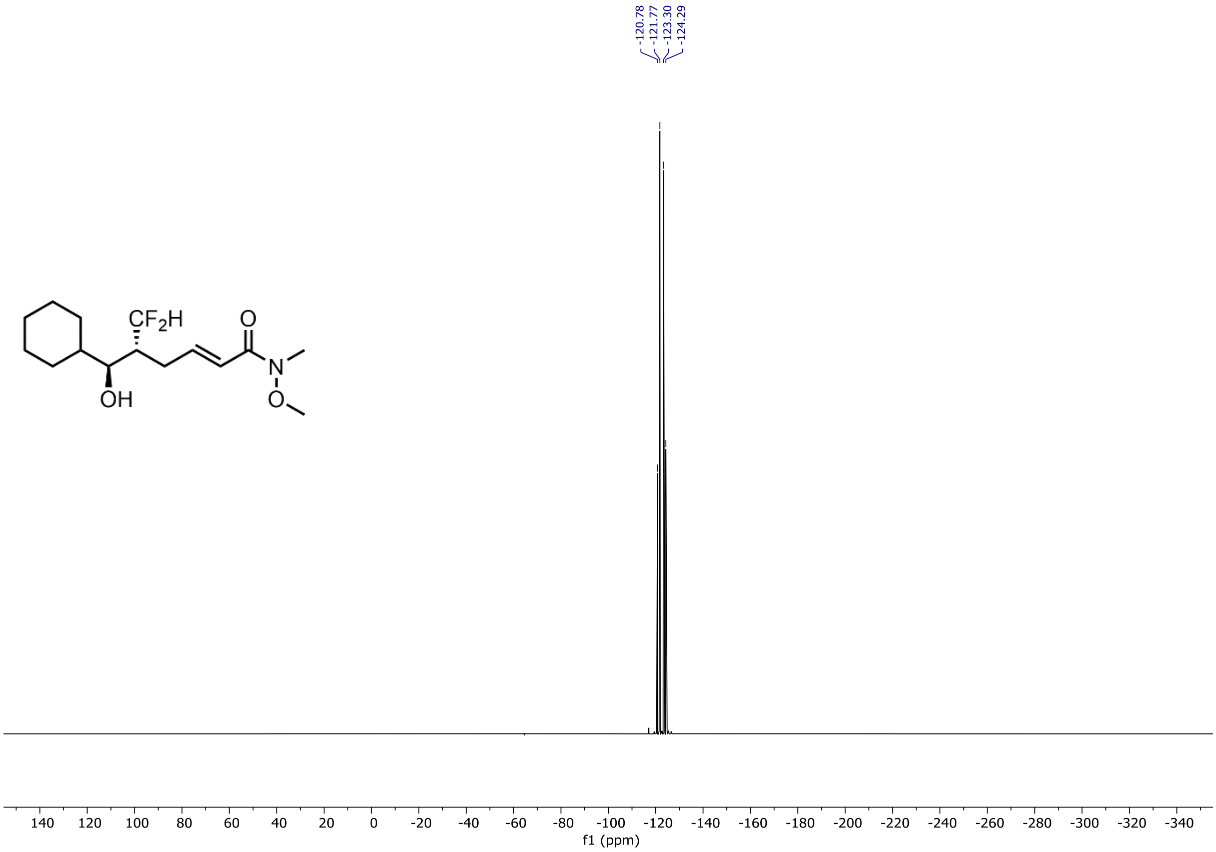
CF2H
O
N
O
OH
-120.78
-121.77
-123.30
-124.29
140 120 100 80 60 40 20 0 -20 -40 -60 -80 -100 -120 -140 -160 -180 -200 -220 -240 -260 -280 -300 -320 -340
f1 (ppm)

^{1}H NMR (400 MHz, CD_2Cl_2; top), ^{13}C NMR (101 MHz, CD_2Cl_2; middle) and ^{19}F NMR (282 MHz, CD_2Cl_2) of compound **19h**

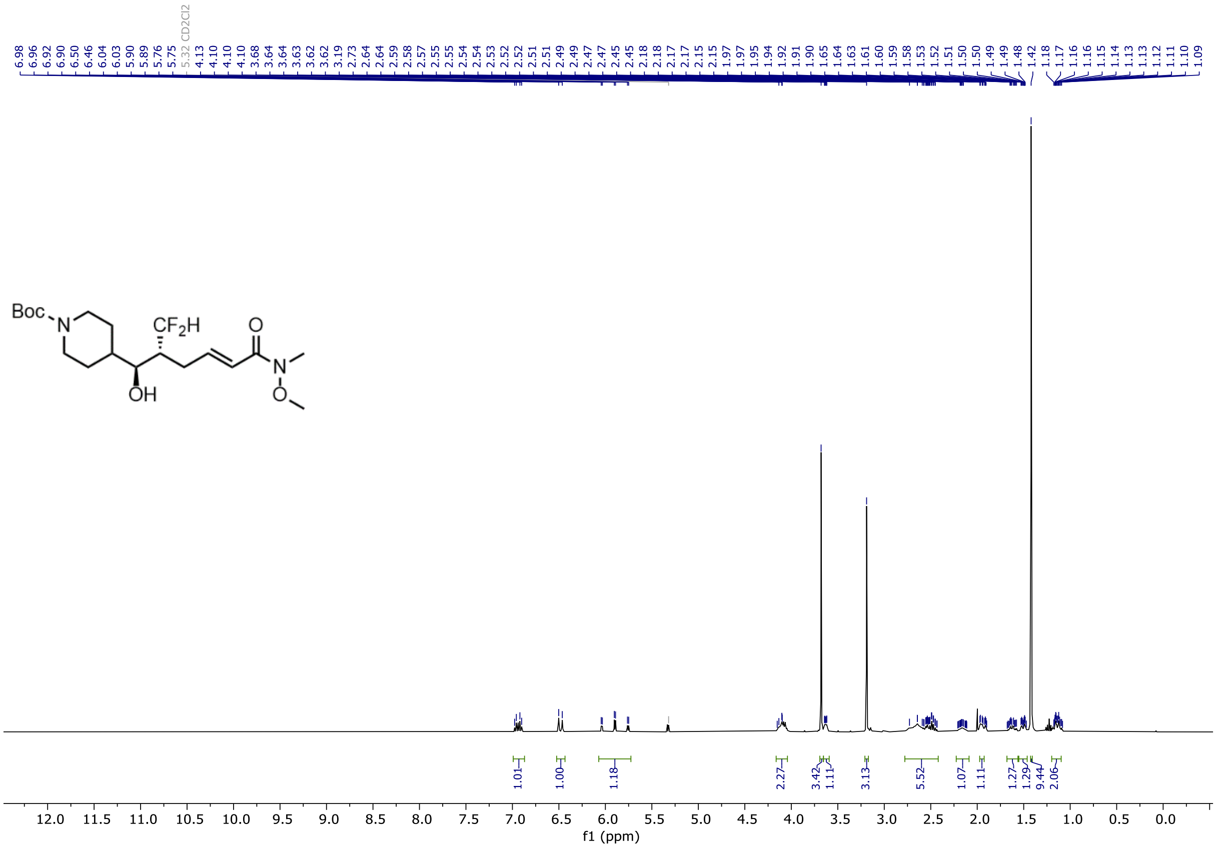

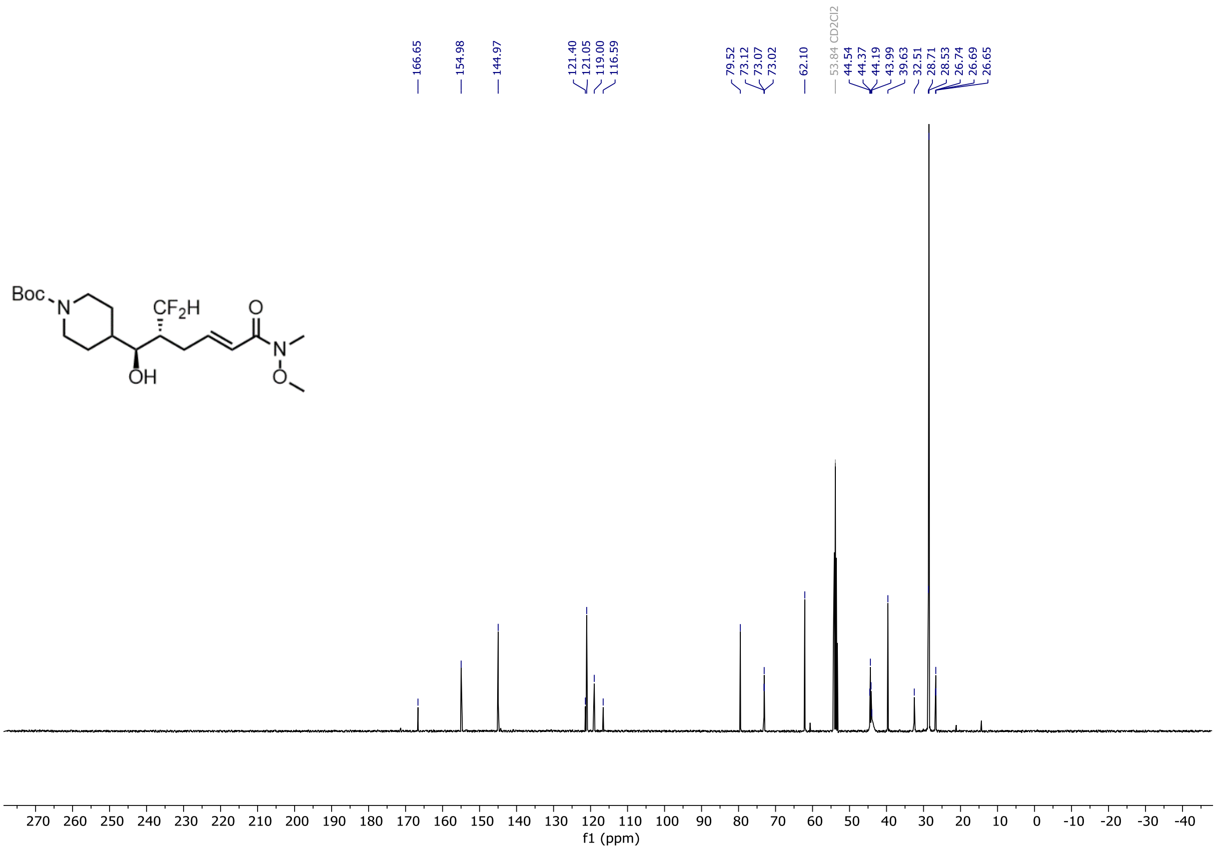

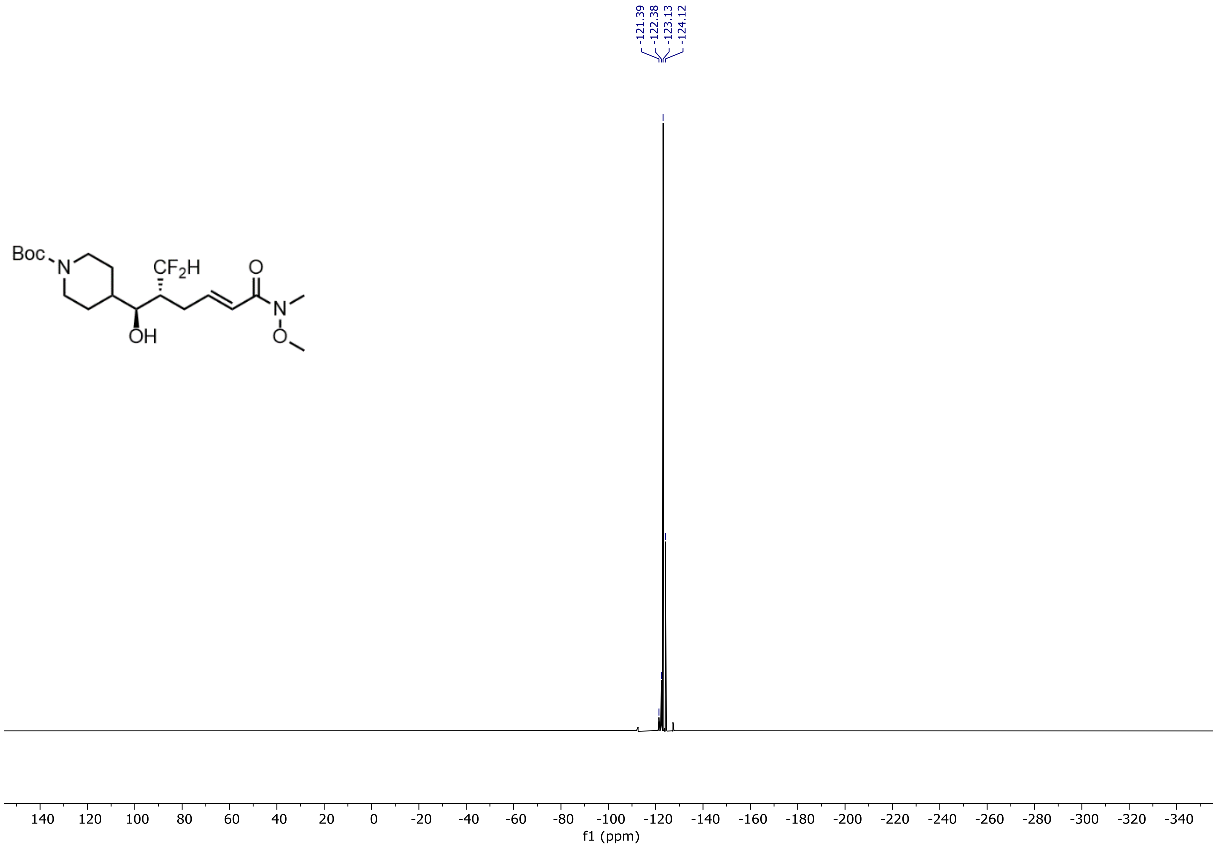
-121.39
-122.38
-123.13
-124.12
Boc
N
CF2H
O
N
OH
O
140 120 100 80 60 40 20 0 -20 -40 -60 -80 -100 -120 -140 -160 -180 -200 -220 -240 -260 -280 -300 -320 -340
f1 (ppm)

^{1}H NMR (400 MHz, CD_2Cl_2; top), ^{13}C NMR (101 MHz, CD_2Cl_2; middle) and ^{19}F NMR (282 MHz, CD_2Cl_2) of compound **19i**

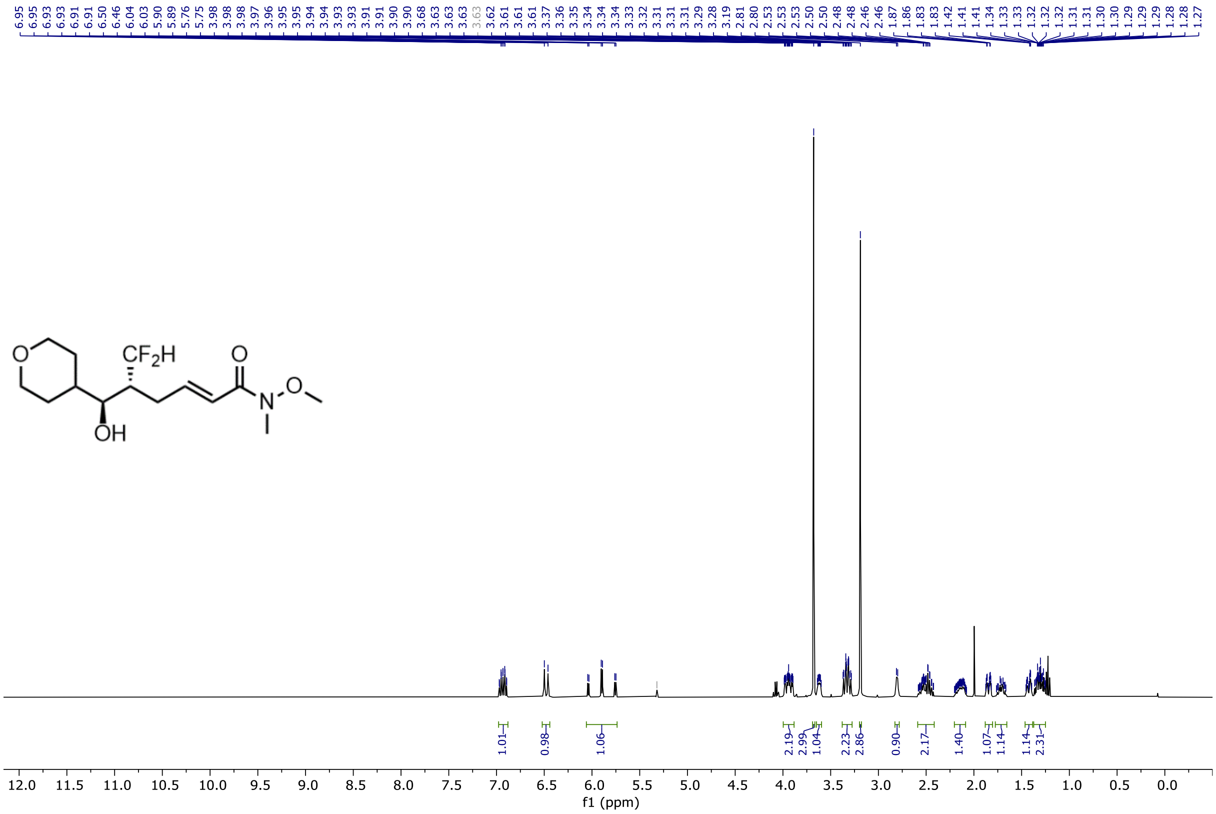

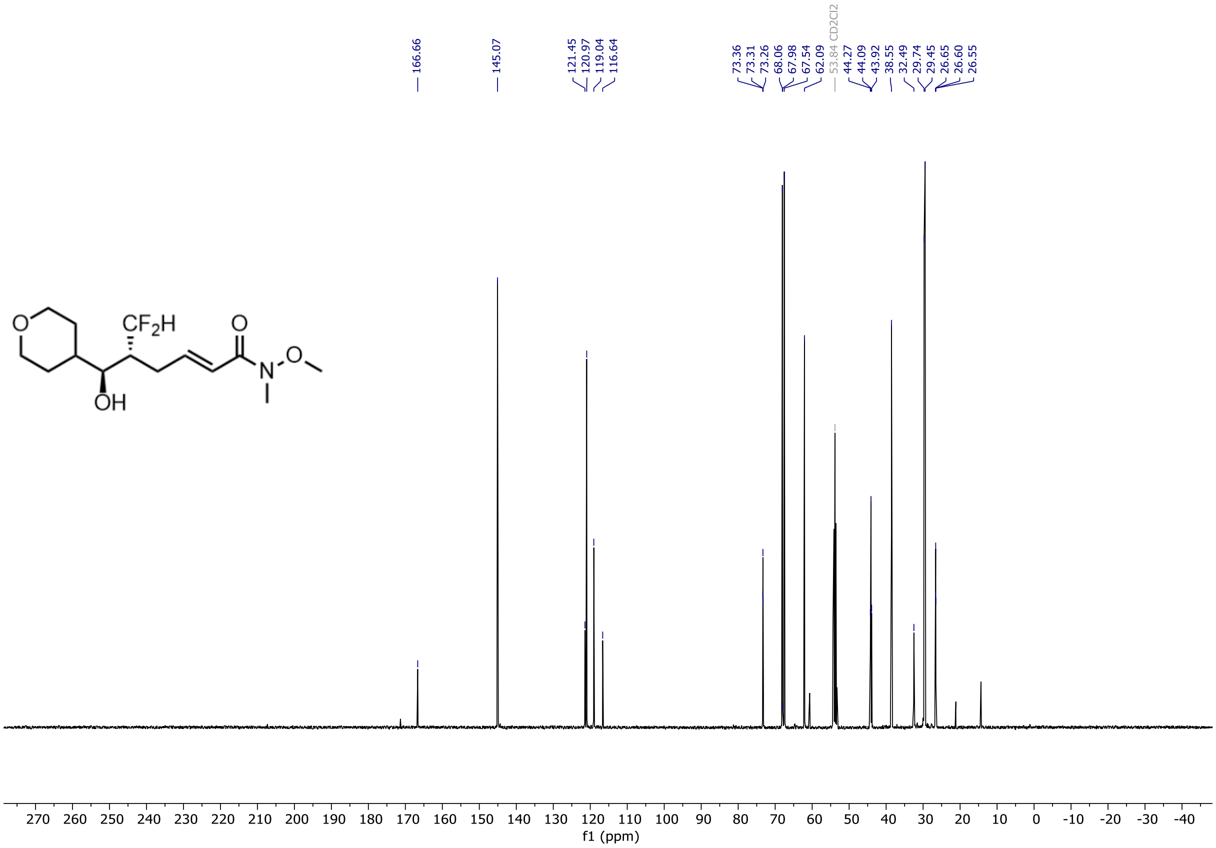

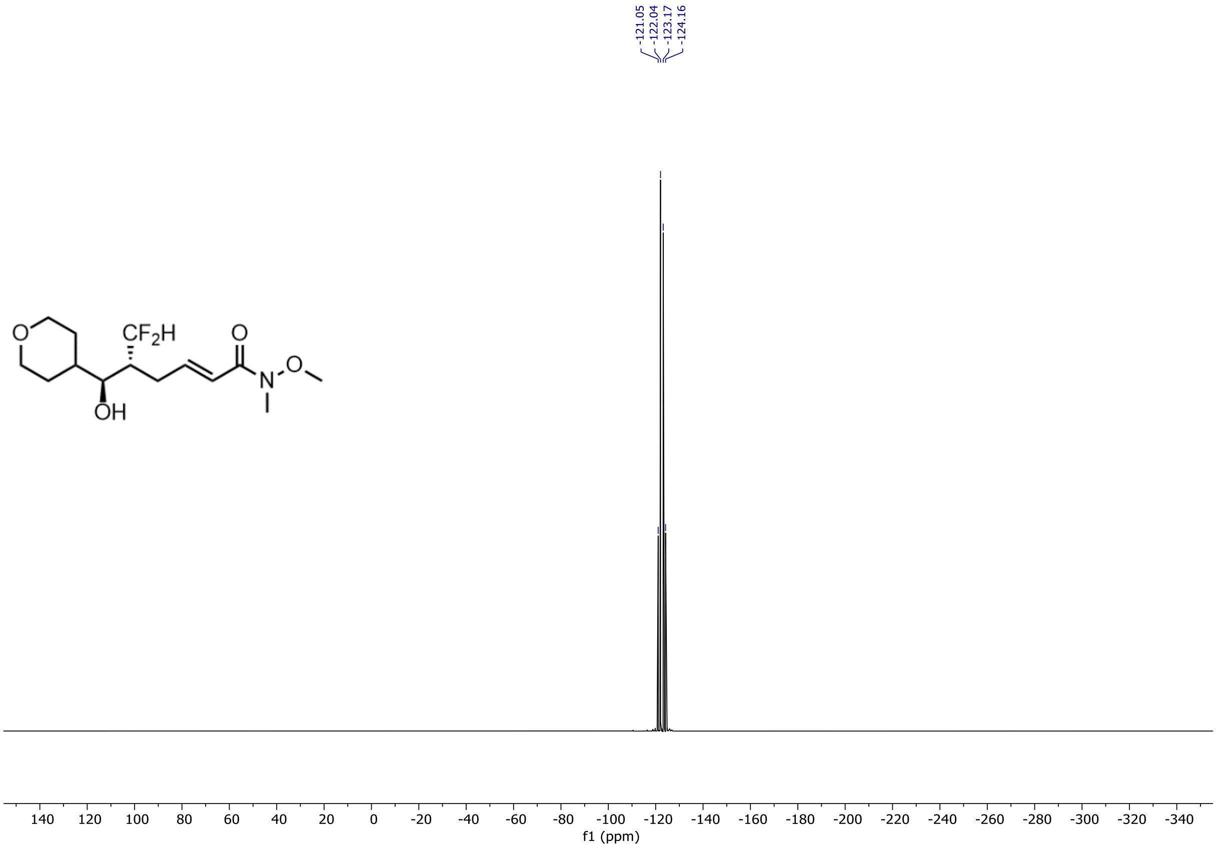

-121.05
-122.04
-123.17
-124.16
140
120
100
80
60
40
20
0
-20
-40
-60
-80
-100
-120
-140
-160
-180
-200
-220
-240
-260
-280
-300
-320
-340
f1 (ppm)

^{1}H NMR (400 MHz, CD_2Cl_2; top), ^{13}C NMR (101 MHz, CD_2Cl_2; middle) and ^{19}F NMR (282 MHz, CD_2Cl_2) of compound **19j**

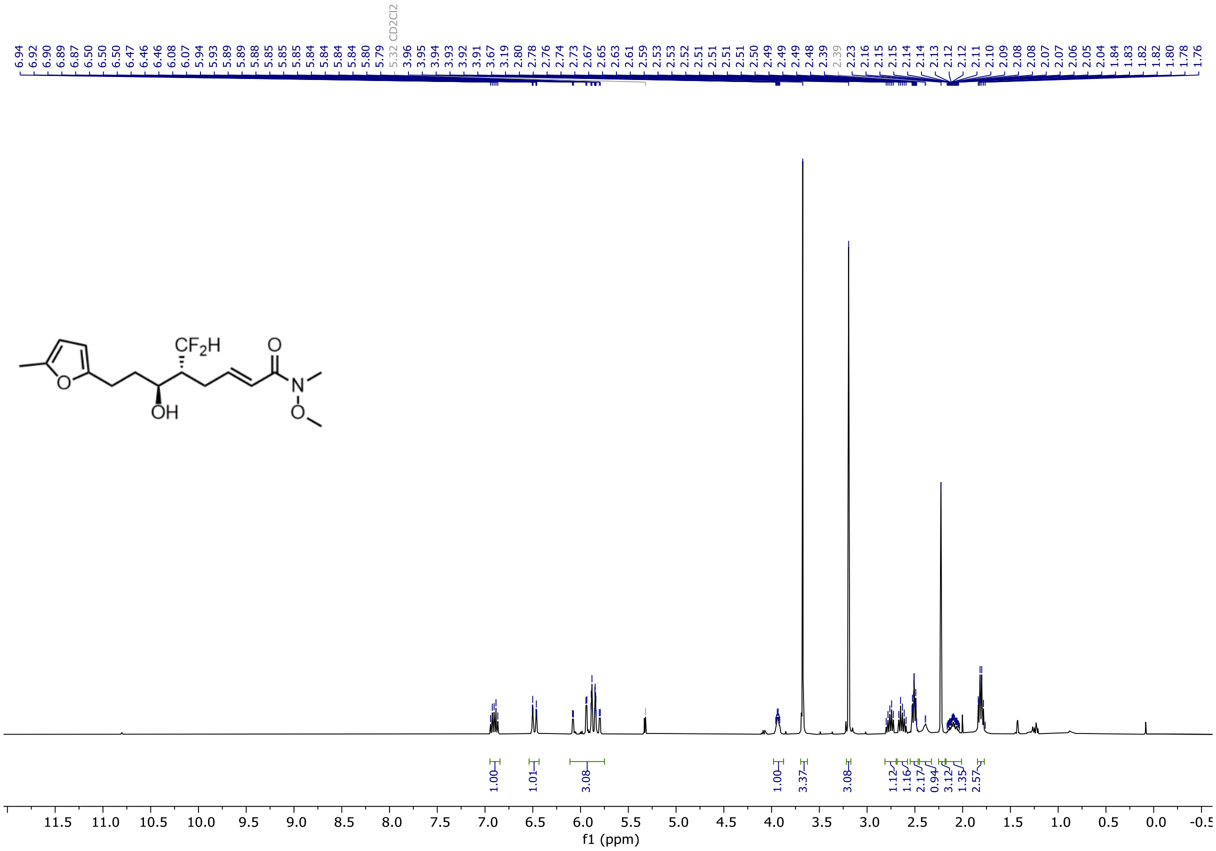

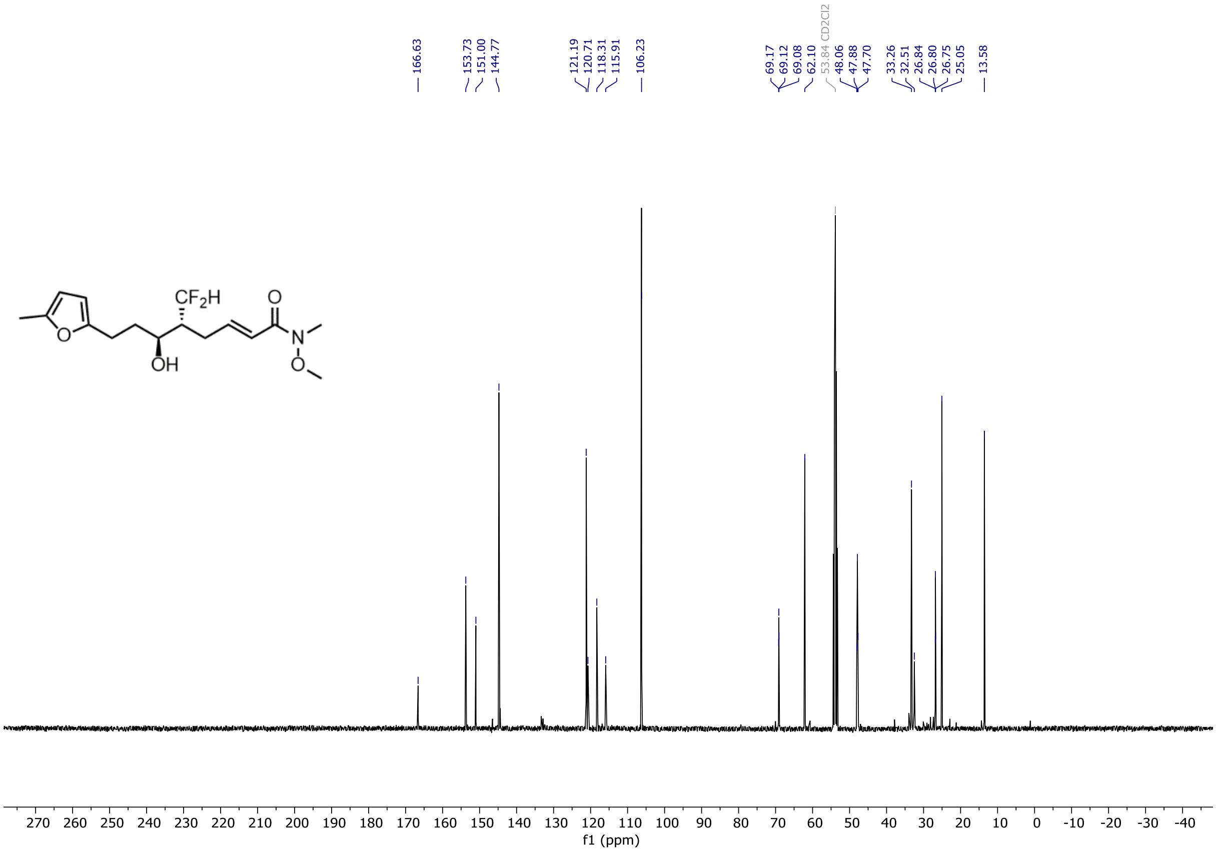

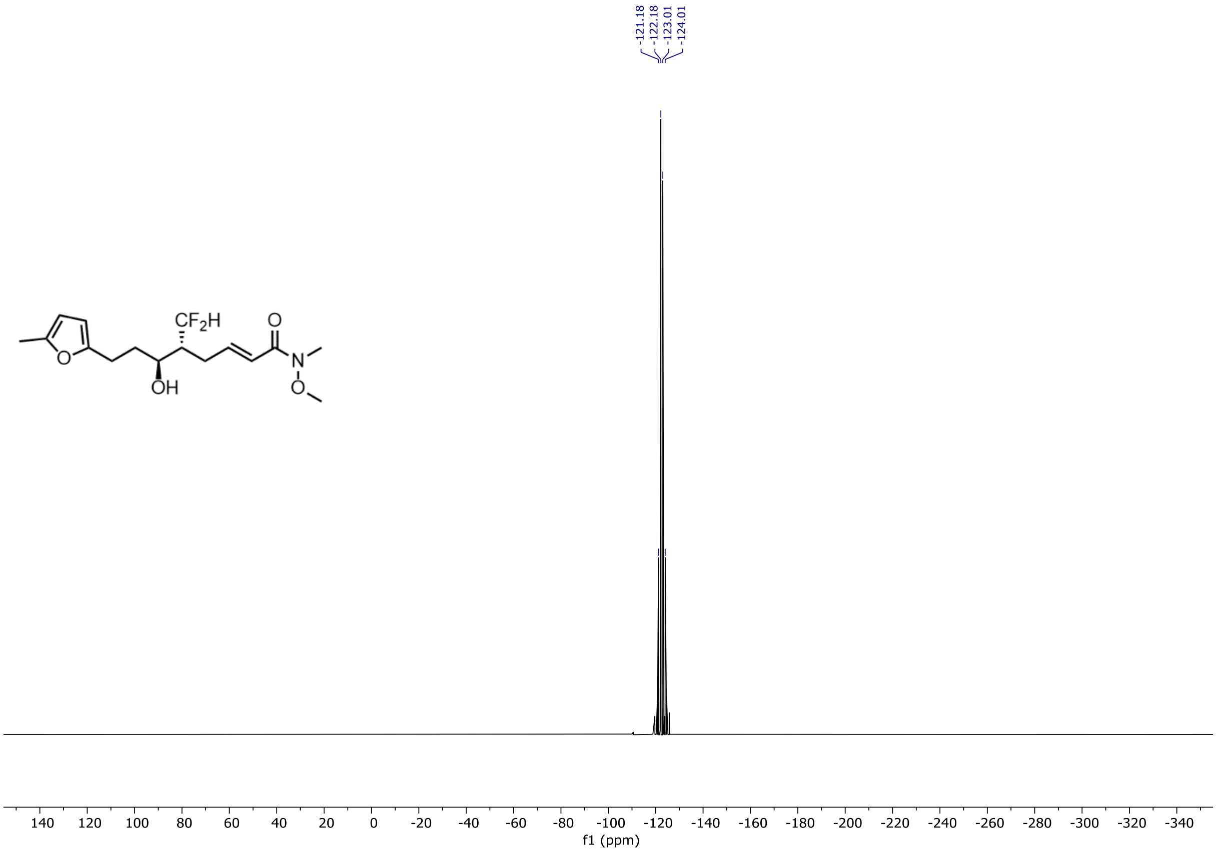
CF2H
O
N
O
OH
O
-121.18
-122.18
-123.01
-124.01
140 120 100 80 60 40 20 0 -20 -40 -60 -80 -100 -120 -140 -160 -180 -200 -220 -240 -260 -280 -300 -320 -340
f1 (ppm)

^{1}H NMR (400 MHz, CD_2Cl_2; top), ^{13}C NMR (101 MHz, CD_2Cl_2; middle) and ^{19}F NMR (282 MHz, CD_2Cl_2) of compound **19k**

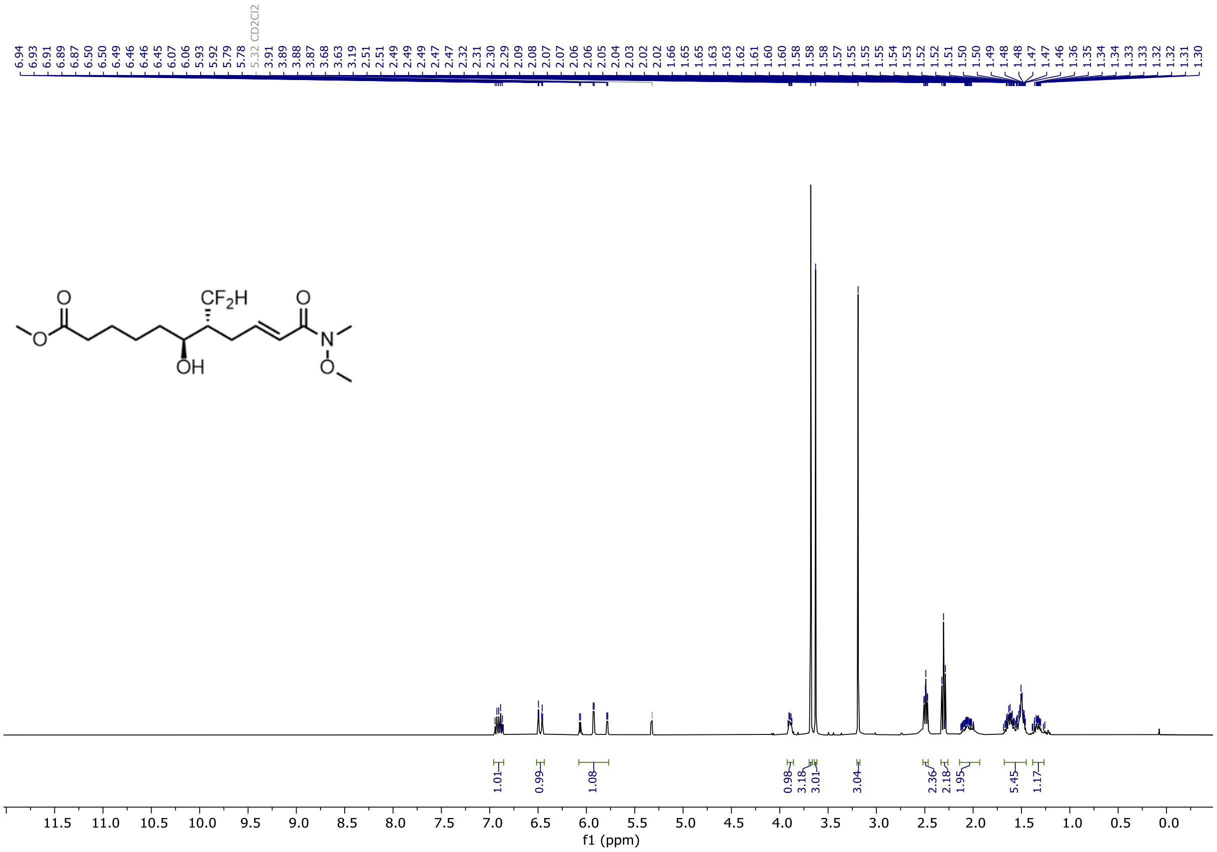

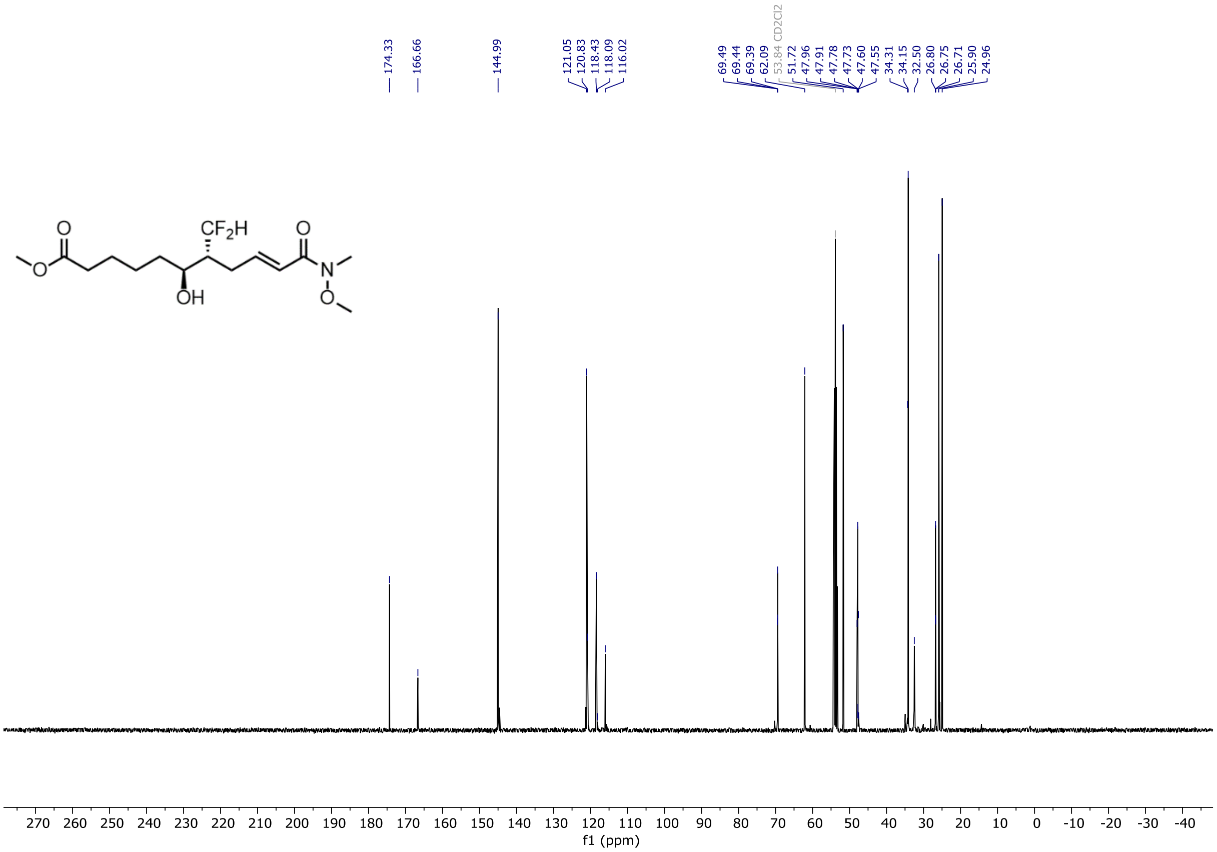

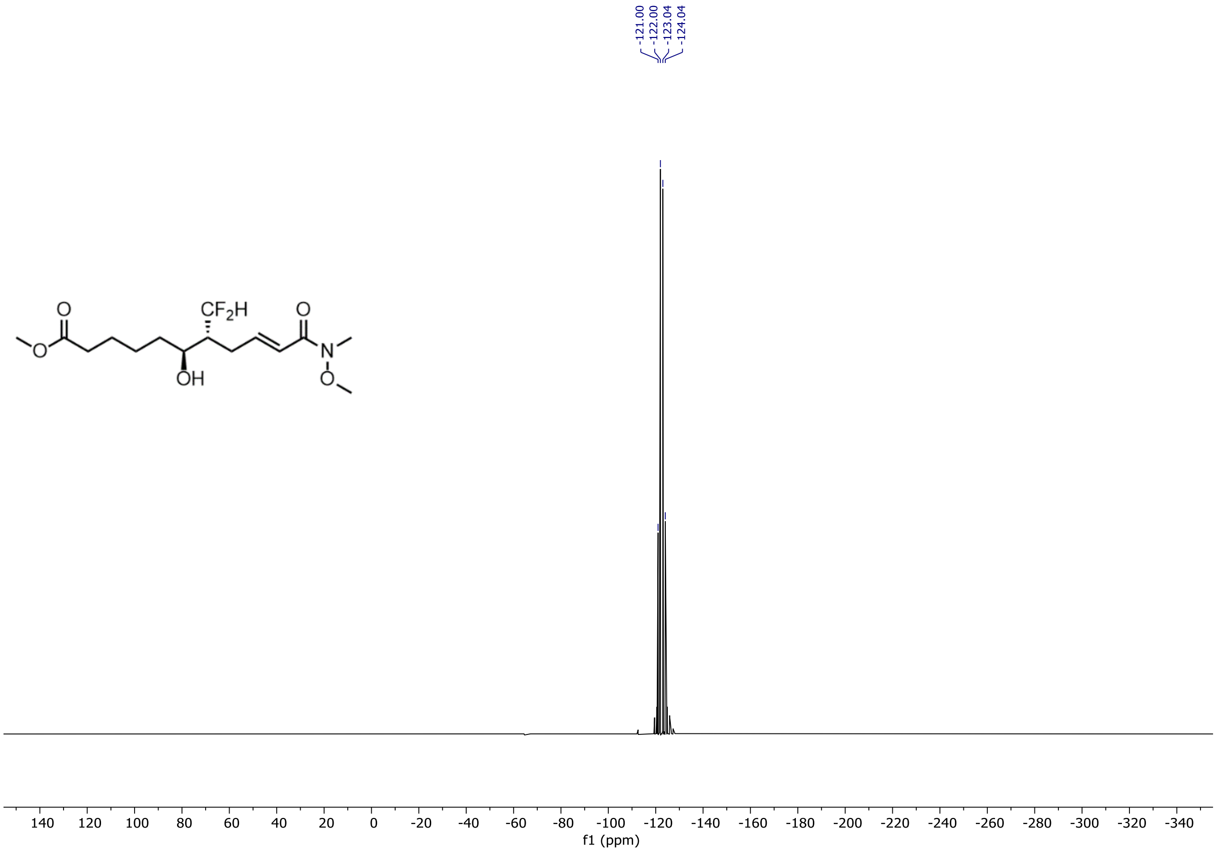
-121.00
-122.00
-123.04
-124.04
140
120
100
80
60
40
20
0
-20
-40
-60
-80
-100
-120
-140
-160
-180
-200
-220
-240
-260
-280
-300
-320
-340
f1 (ppm)

^{1}H NMR (400 MHz, CD_2Cl_2; top), ^{13}C NMR (101 MHz, CD_2Cl_2; middle) and ^{19}F NMR (282 MHz, CD_2Cl_2) of compound **19l**

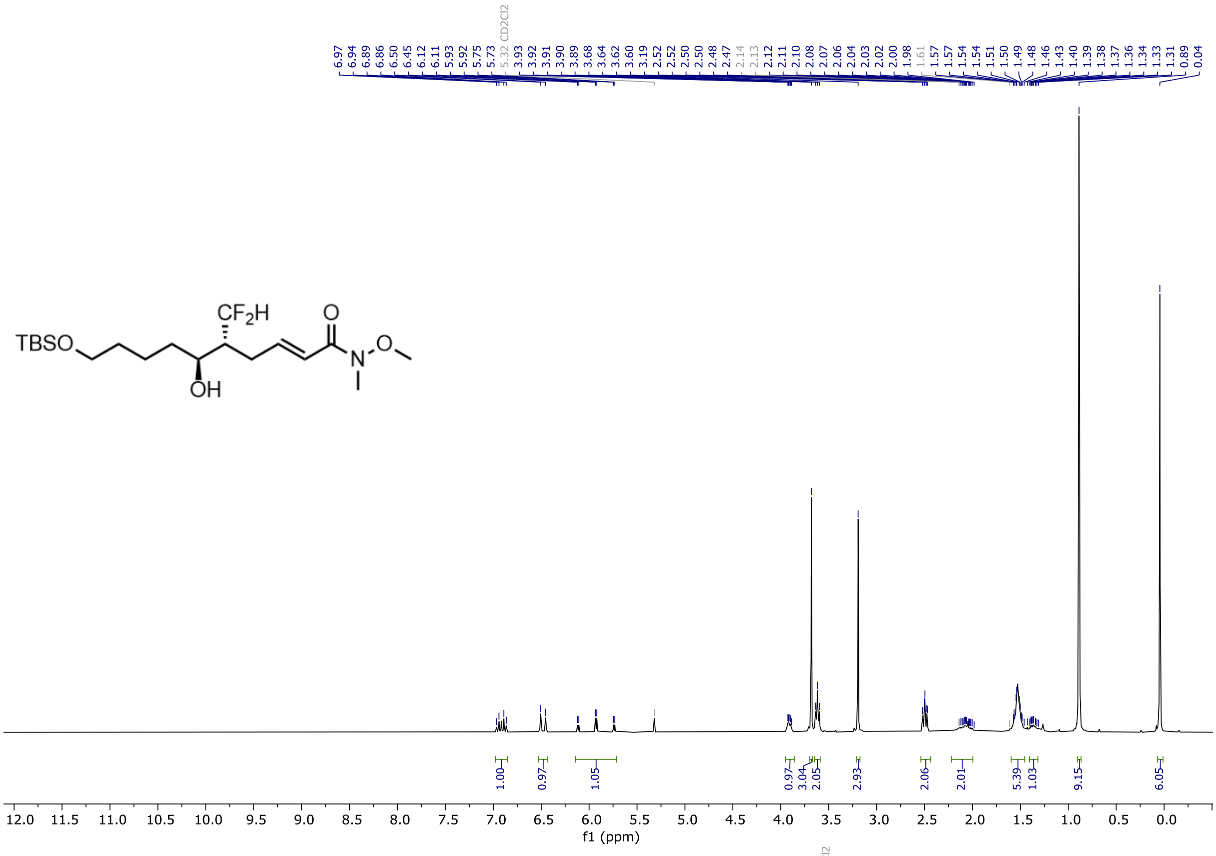

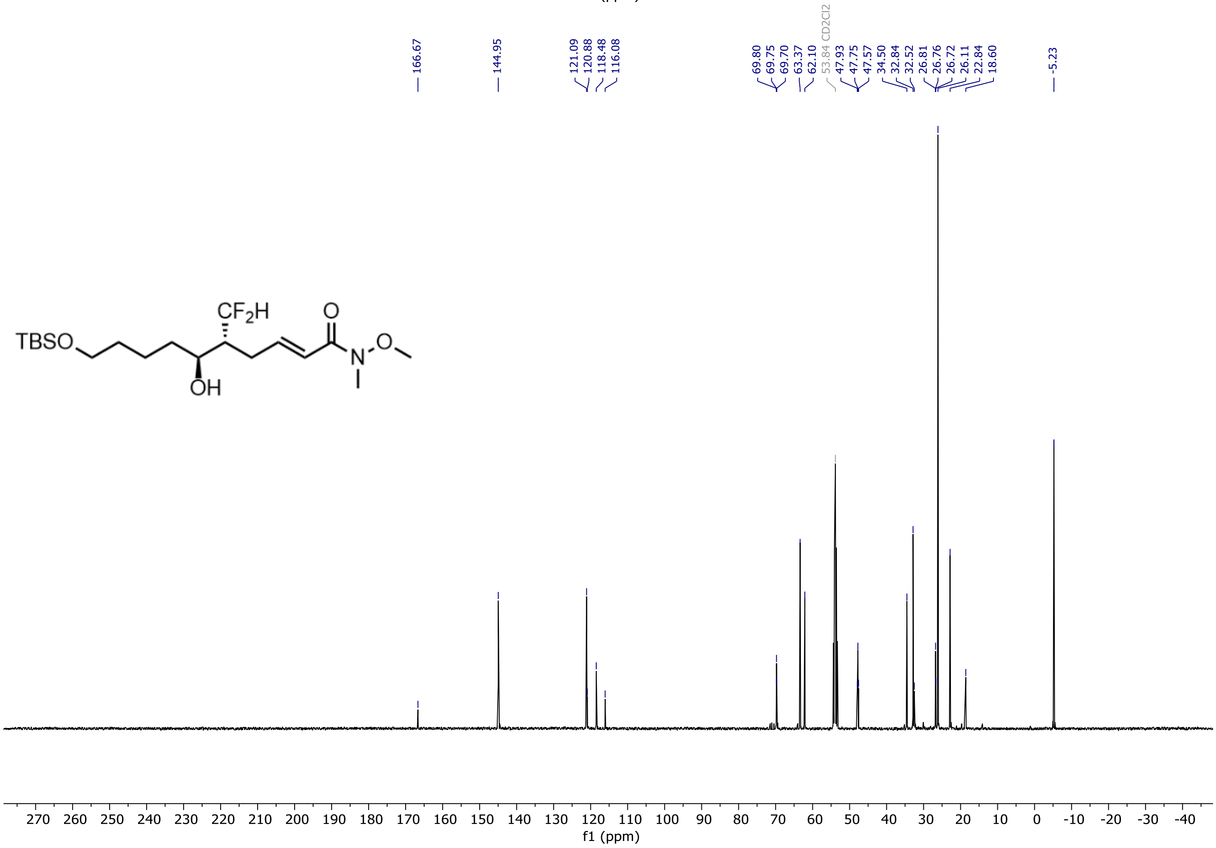

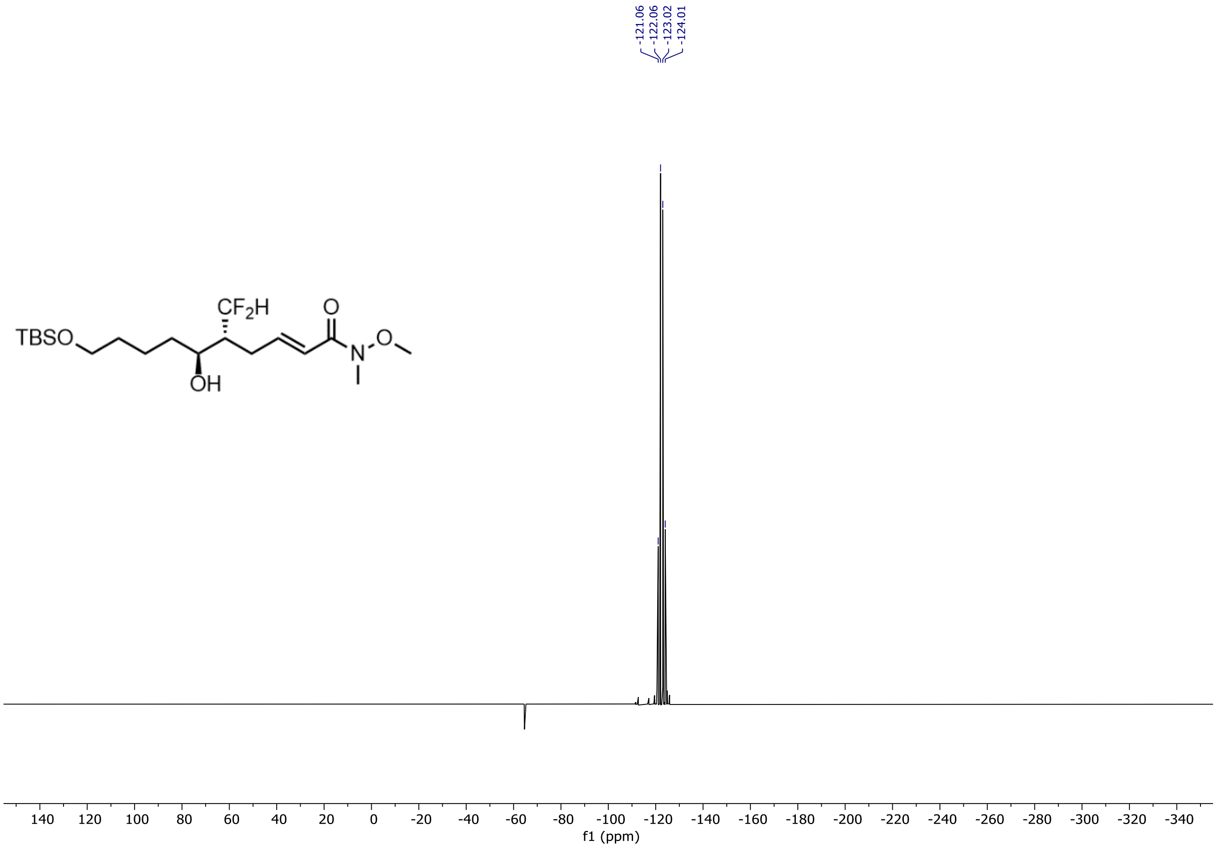
-121.06
-122.06
-123.02
-124.01
TBSO
CF2H
O
N
O
OH
140 120 100 80 60 40 20 0 -20 -40 -60 -80 -100 -120 -140 -160 -180 -200 -220 -240 -260 -280 -300 -320 -340
f1 (ppm)

^{1}H NMR (400 MHz, CD_2Cl_2; top), ^{13}C NMR (101 MHz, CD_2Cl_2; middle) and ^{19}F NMR (282 MHz, CD_2Cl_2) of compound **19m**

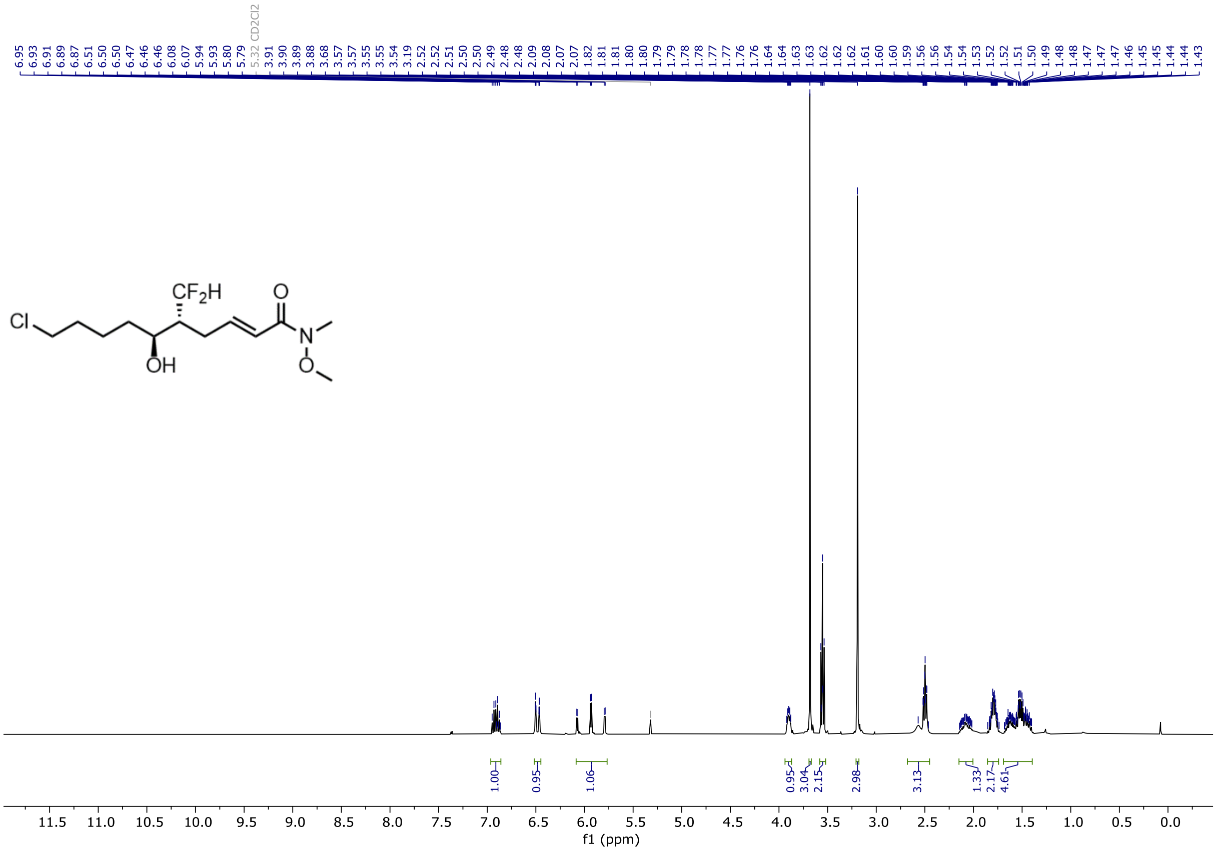

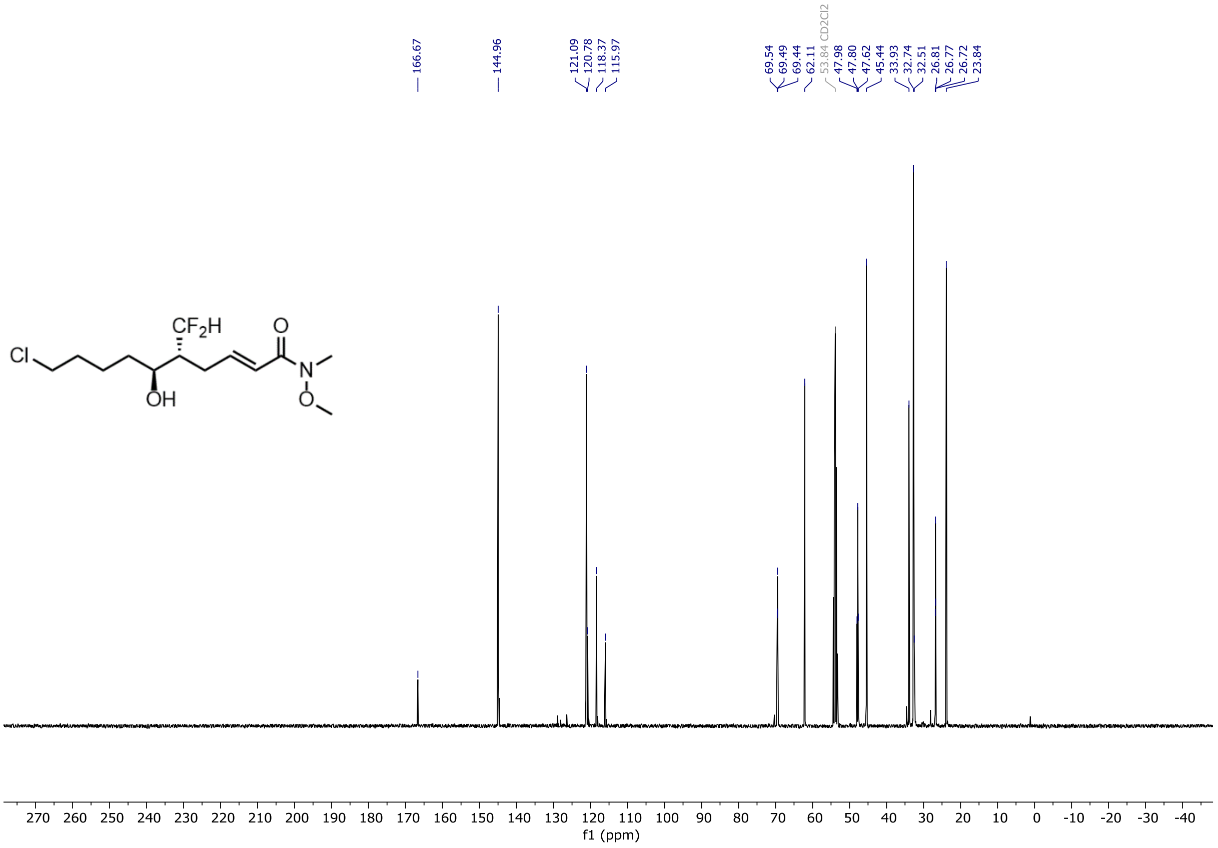

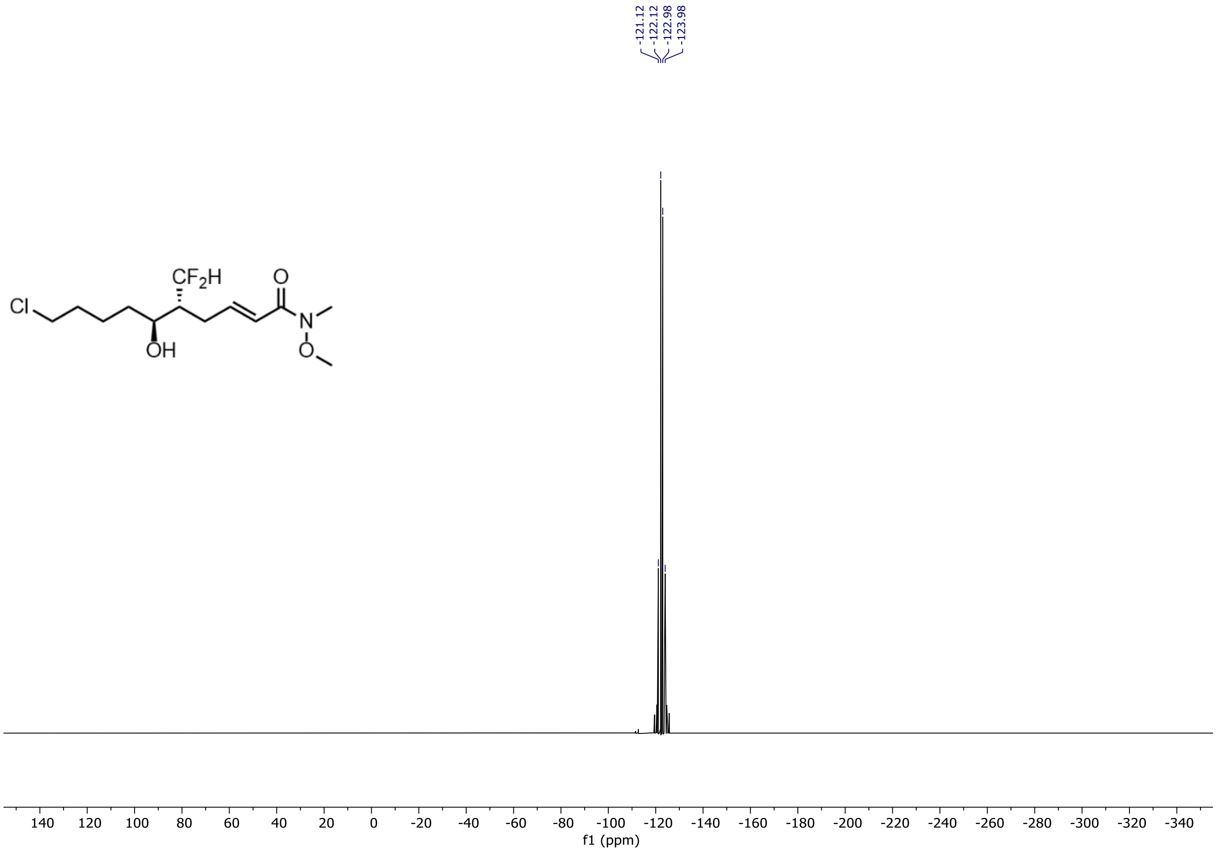
-121.12
-122.12
-122.98
-123.98
CF2H
Cl
OH
O
N
O
140 120 100 80 60 40 20 0 -20 -40 -60 -80 -100 -120 -140 -160 -180 -200 -220 -240 -260 -280 -300 -320 -340
f1 (ppm)

^{1}H NMR (400 MHz, CD_2Cl_2; top), ^{13}C NMR (101 MHz, CD_2Cl_2; middle) and ^{19}F NMR (282 MHz, CD_2Cl_2) of compound **19n**

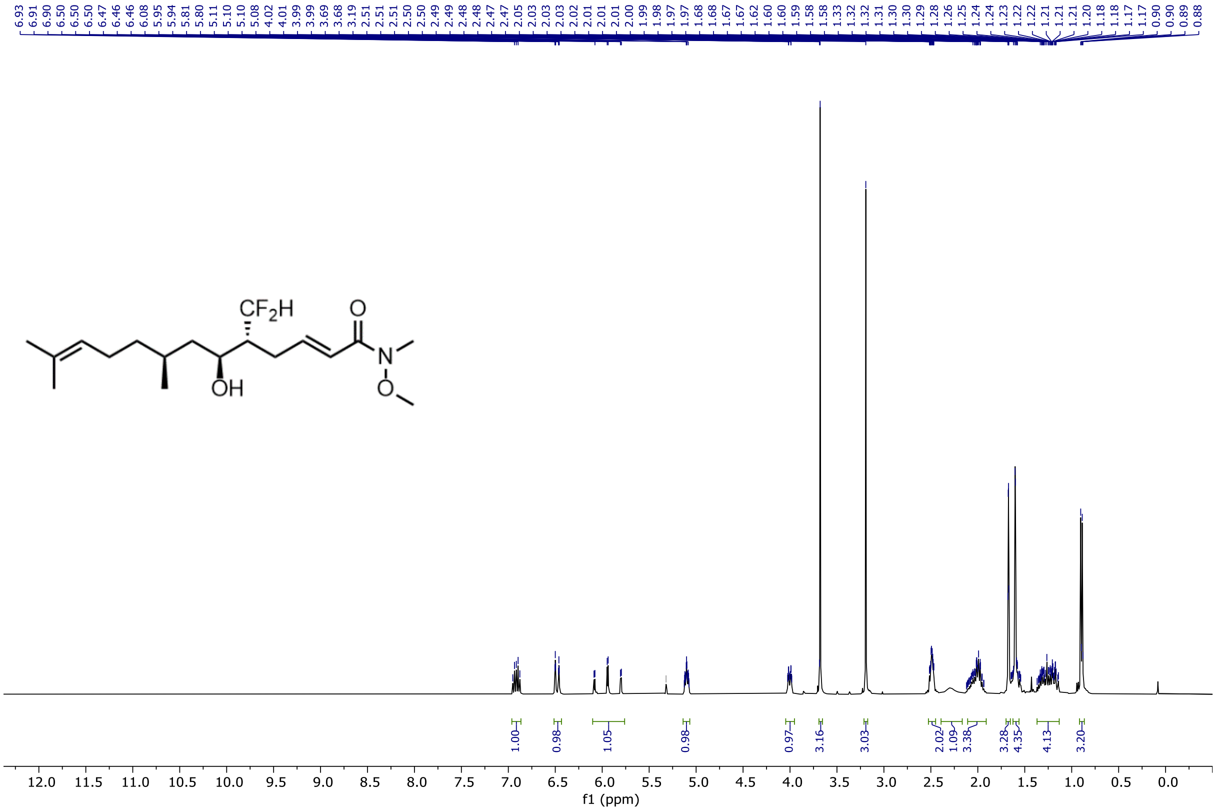

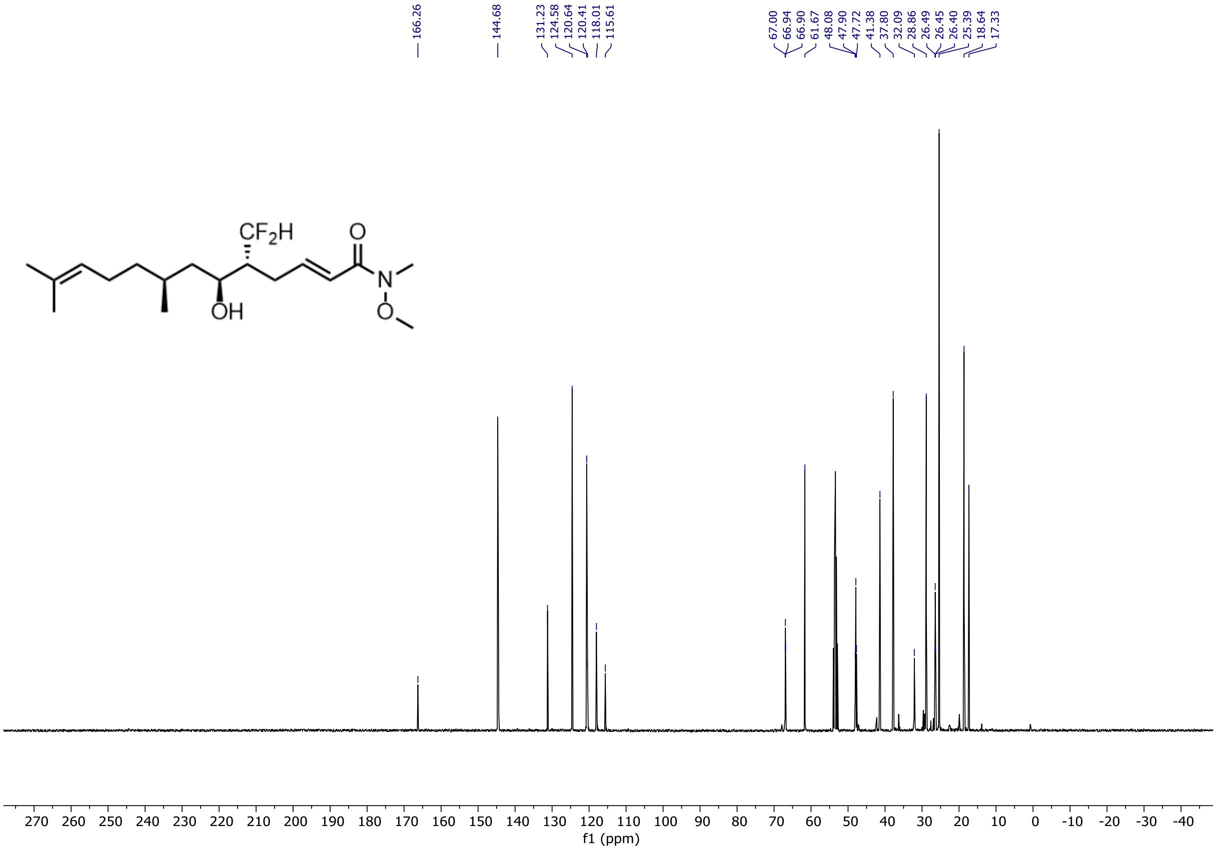

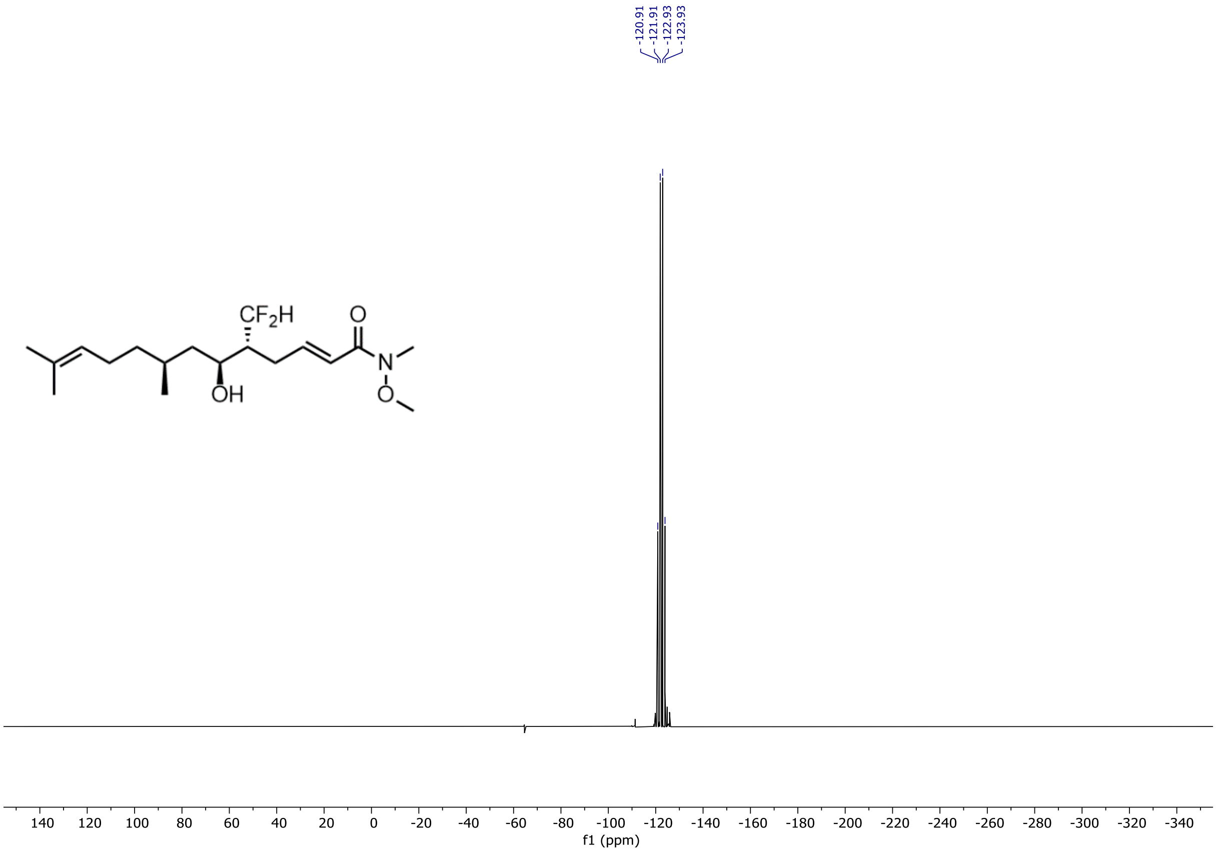
-120.91
-121.91
-122.93
-123.93
140
120
100
80
60
40
20
0
-20
-40
-60
-80
-100
-120
-140
-160
-180
-200
-220
-240
-260
-280
-300
-320
-340
f1 (ppm)

^{1}H NMR (400 MHz, CD_2Cl_2; top), ^{13}C NMR (101 MHz, CD_2Cl_2; middle) and ^{19}F NMR (282 MHz, CD_2Cl_2) of compound **19o**

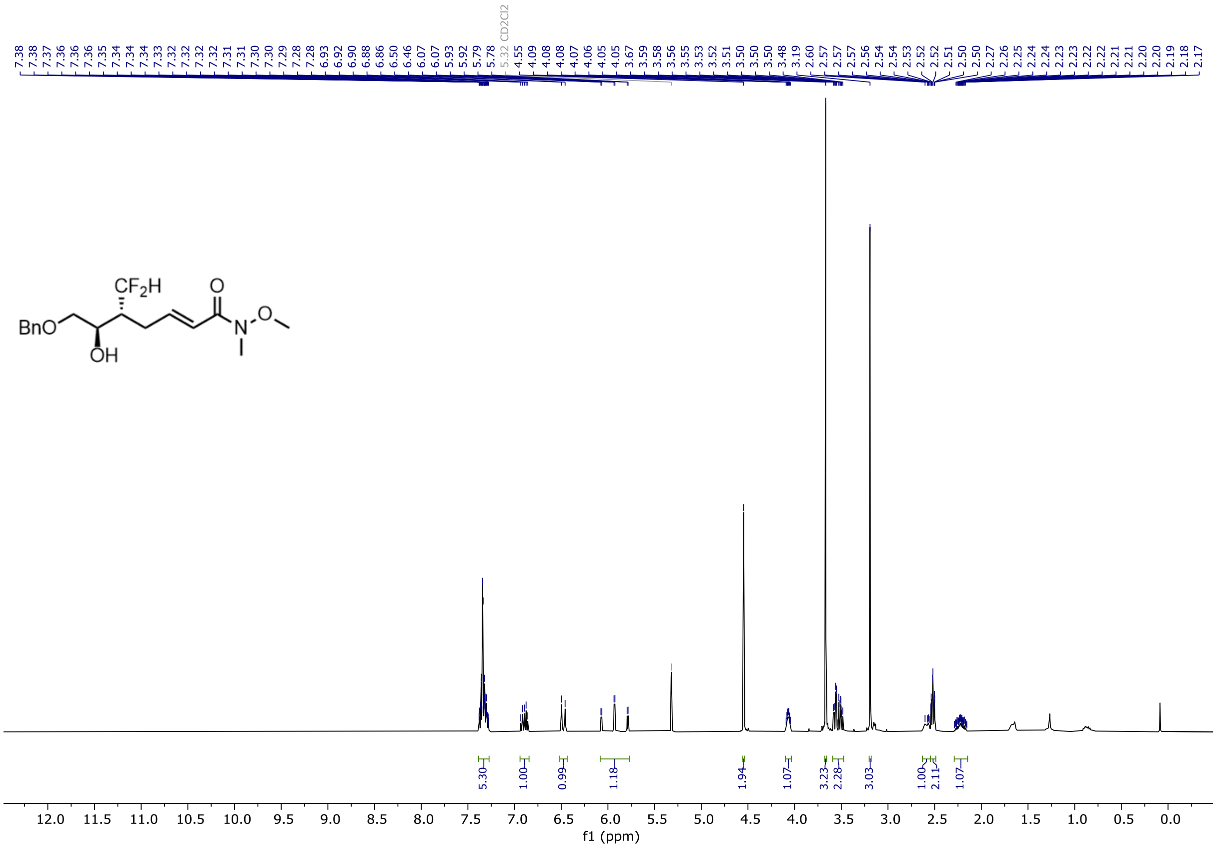

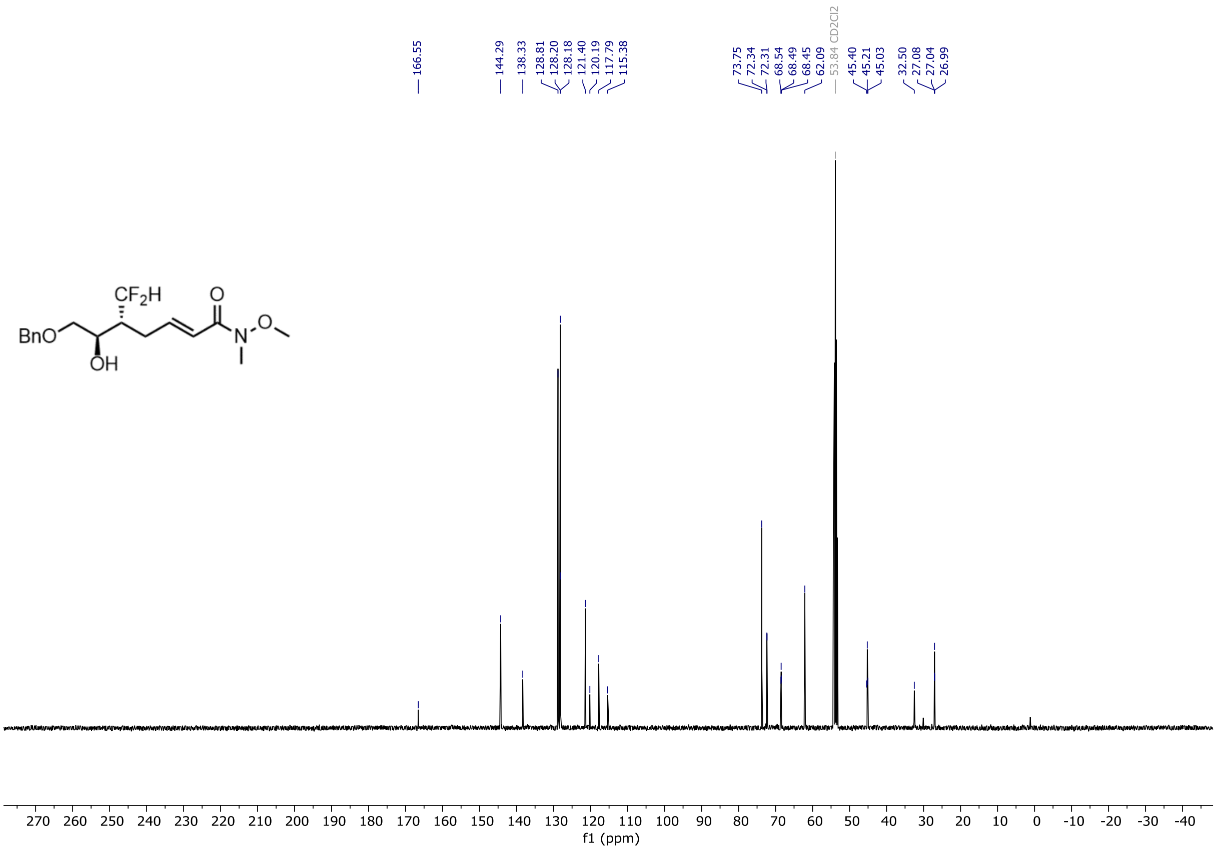

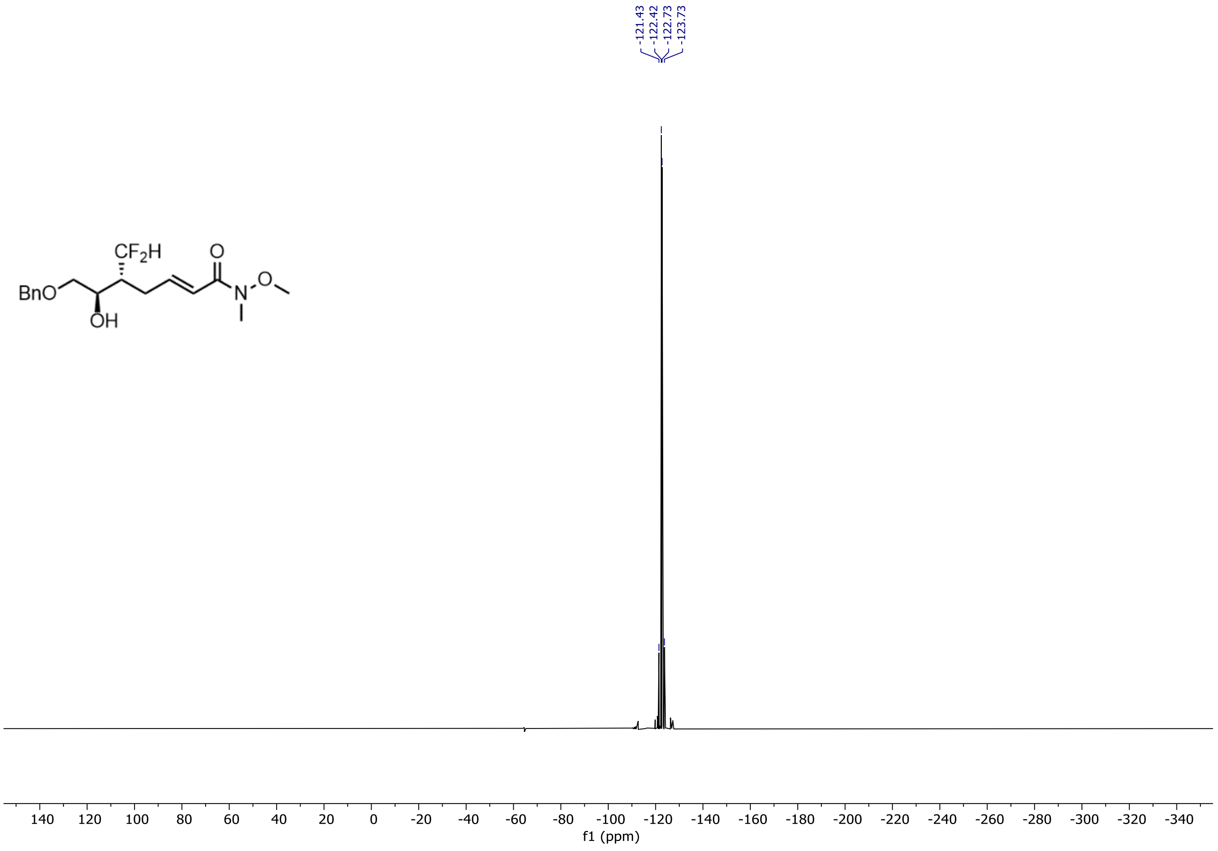
-121.43
-122.42
-122.73
-123.73
CF2H
O
BnO
N
O
OH
140 120 100 80 60 40 20 0 -20 -40 -60 -80 -100 -120 -140 -160 -180 -200 -220 -240 -260 -280 -300 -320 -340
f1 (ppm)

^{1}H NMR (400 MHz, CD_2Cl_2; top), ^{13}C NMR (101 MHz, CD_2Cl_2; middle) and ^{19}F NMR (282 MHz, CD_2Cl_2) of compound **19p (dr ≈ 10:1)**

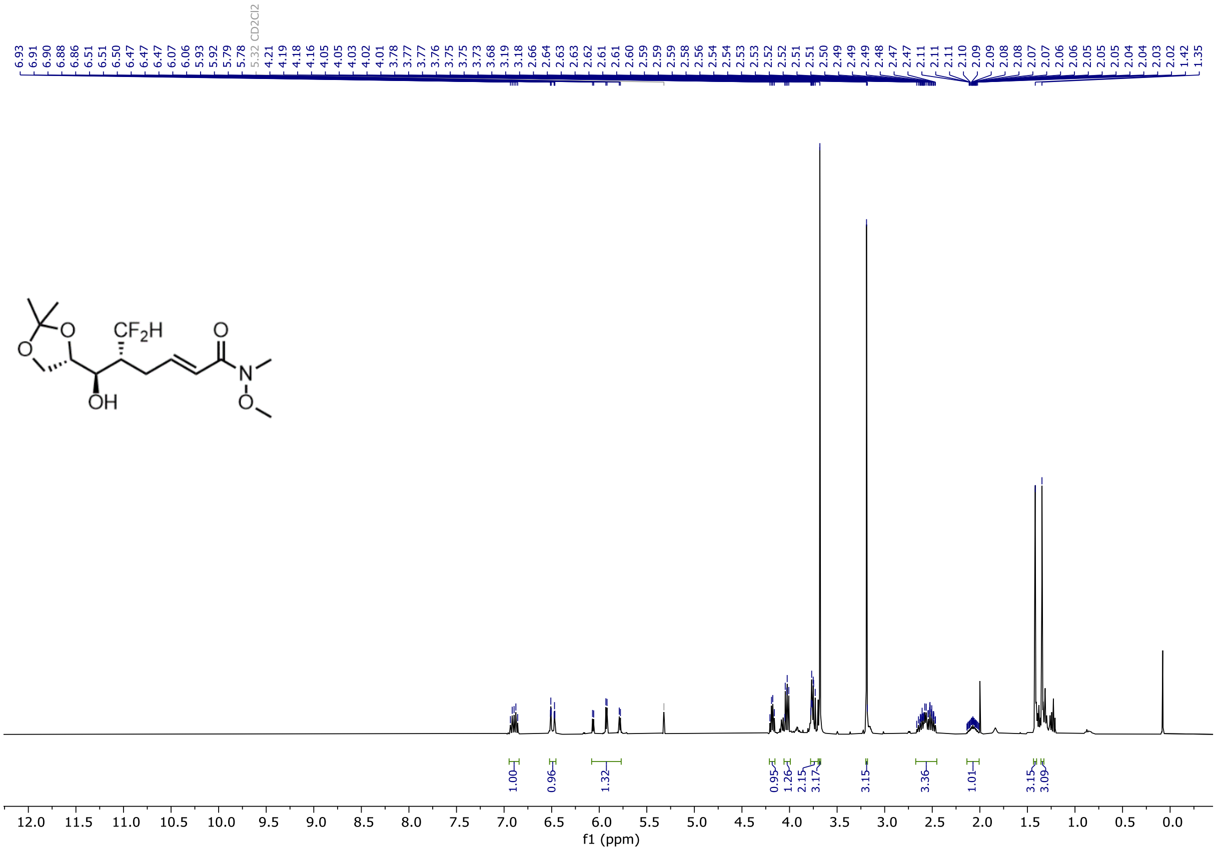

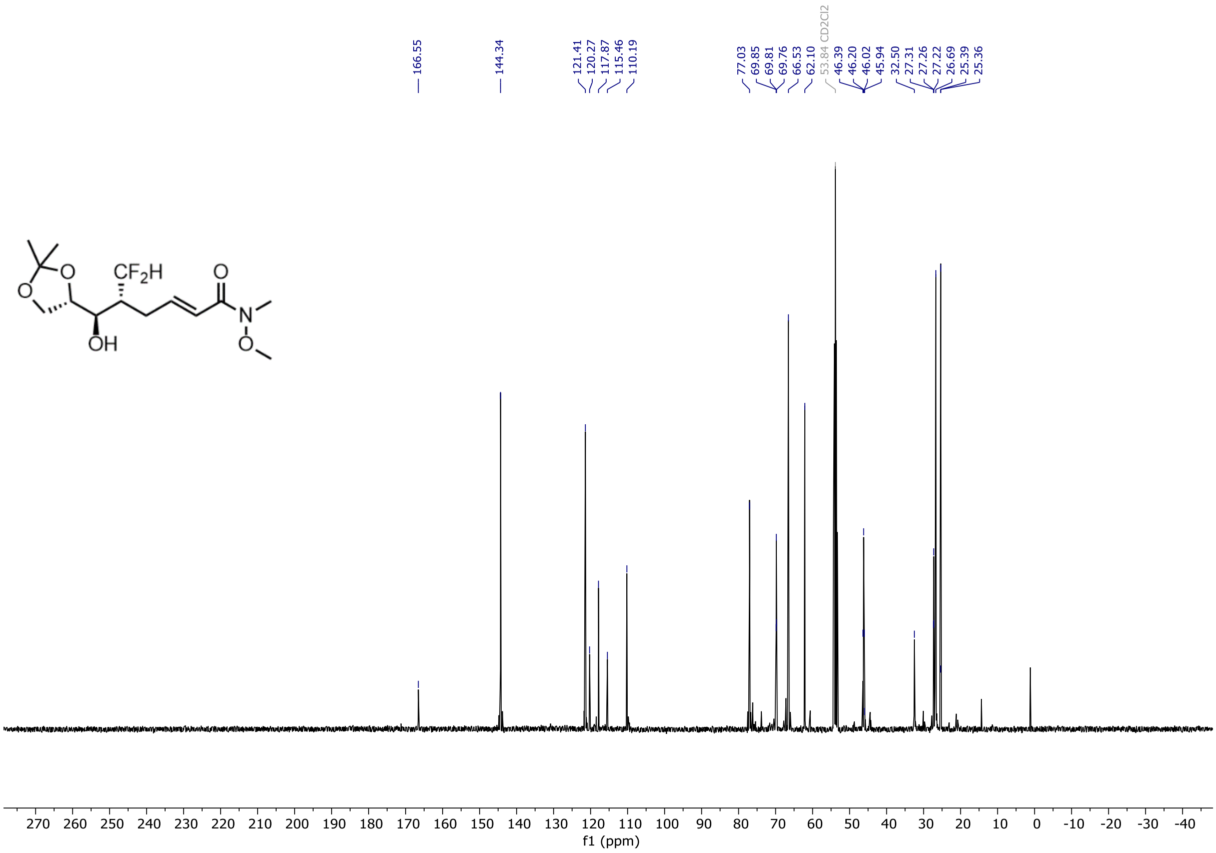

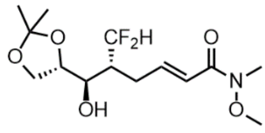

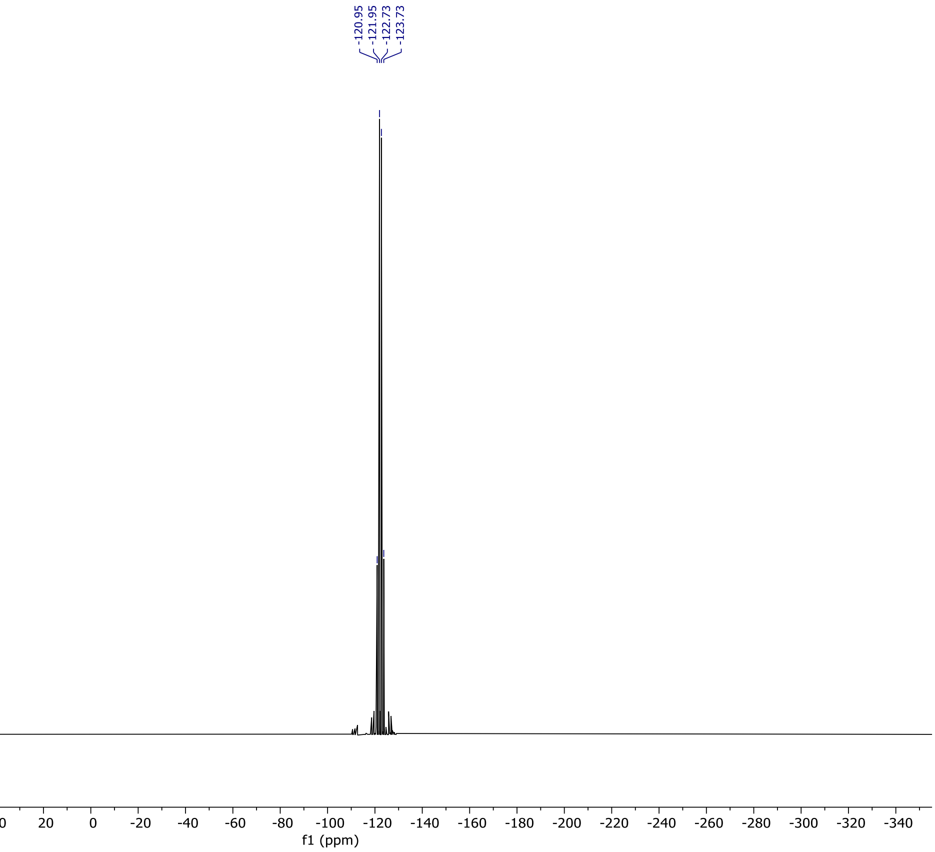

-120.95
-121.95
-122.73
-123.73
140 120 100 80 60 40 20 0 -20 -40 -60 -80 -100 -120 -140 -160 -180 -200 -220 -240 -260 -280 -300 -320 -340
f1 (ppm)

^{1}H NMR (400 MHz, CD_2Cl_2; top), ^{13}C NMR (101 MHz, CD_2Cl_2; middle) and ^{19}F NMR (282 MHz, CD_2Cl_2) of compound **19q (dr ≈ 4:1)**

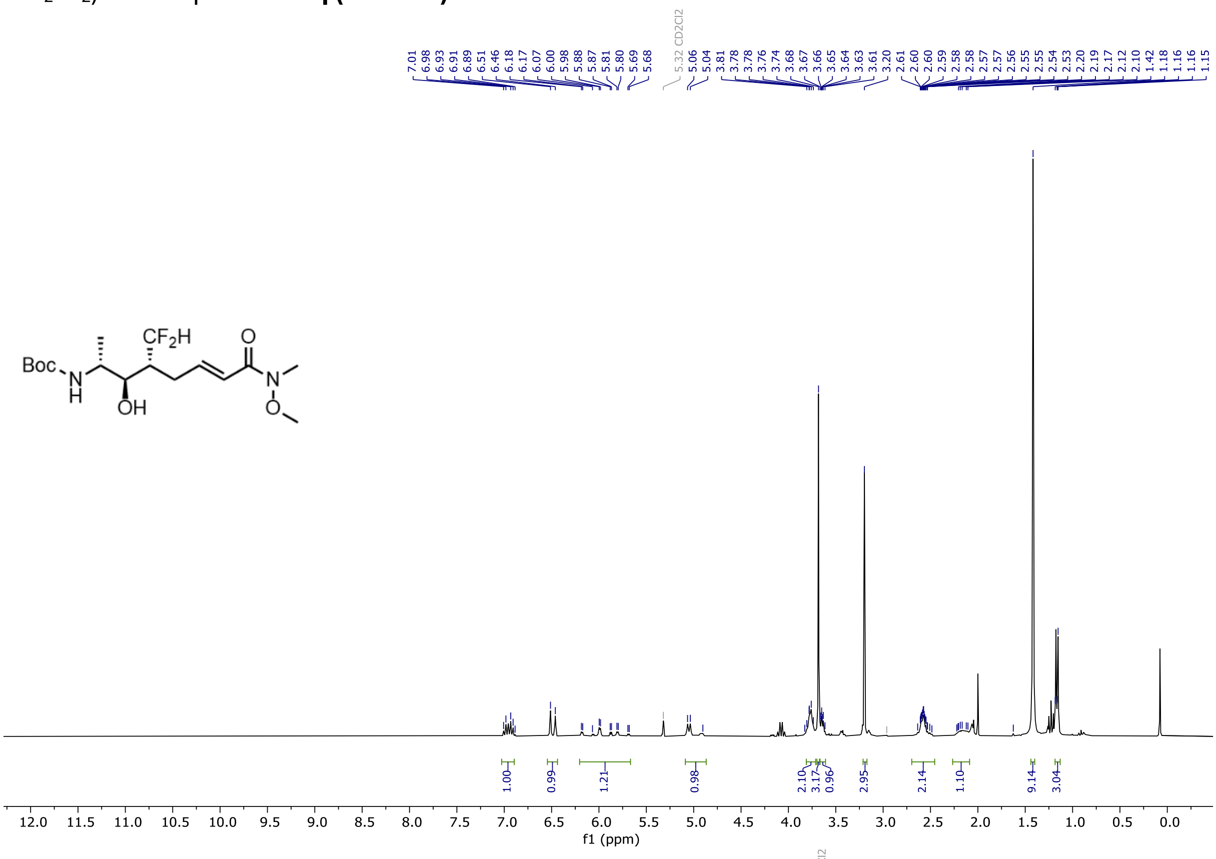

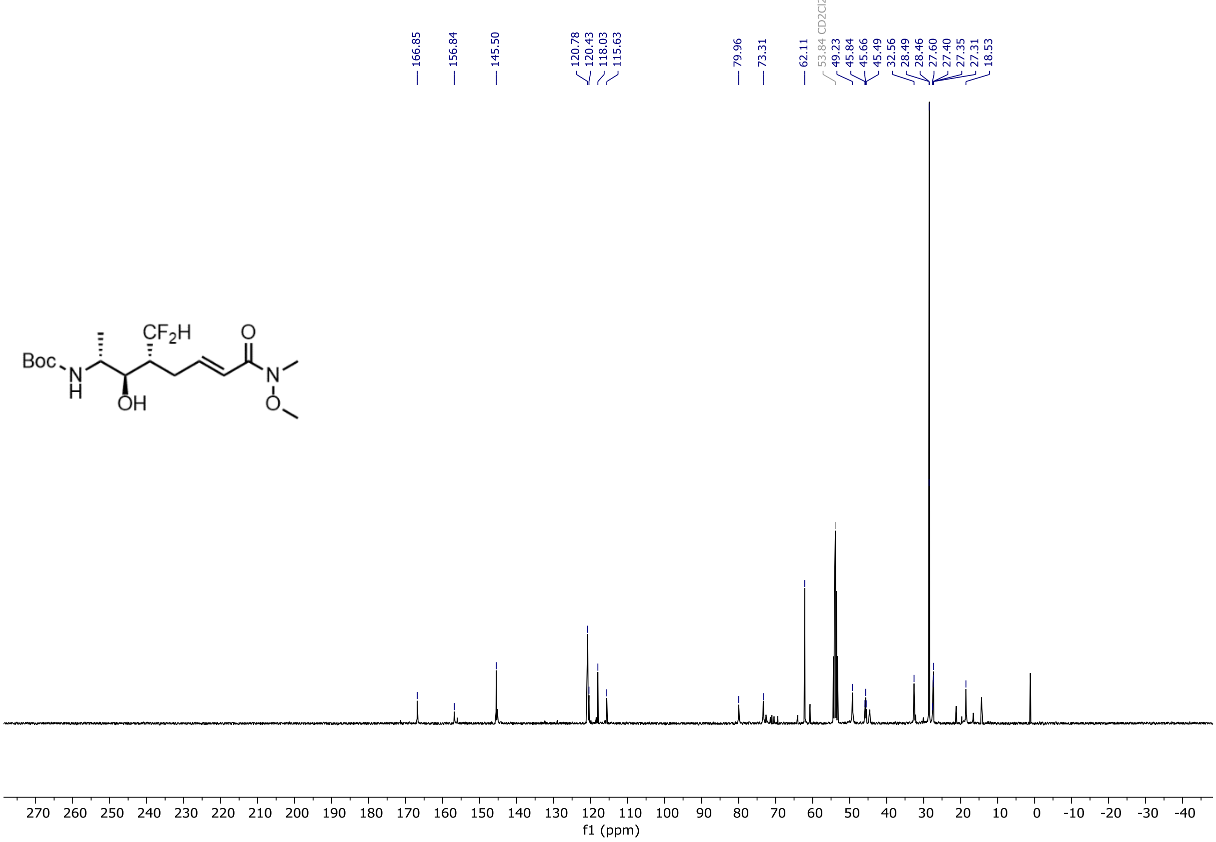

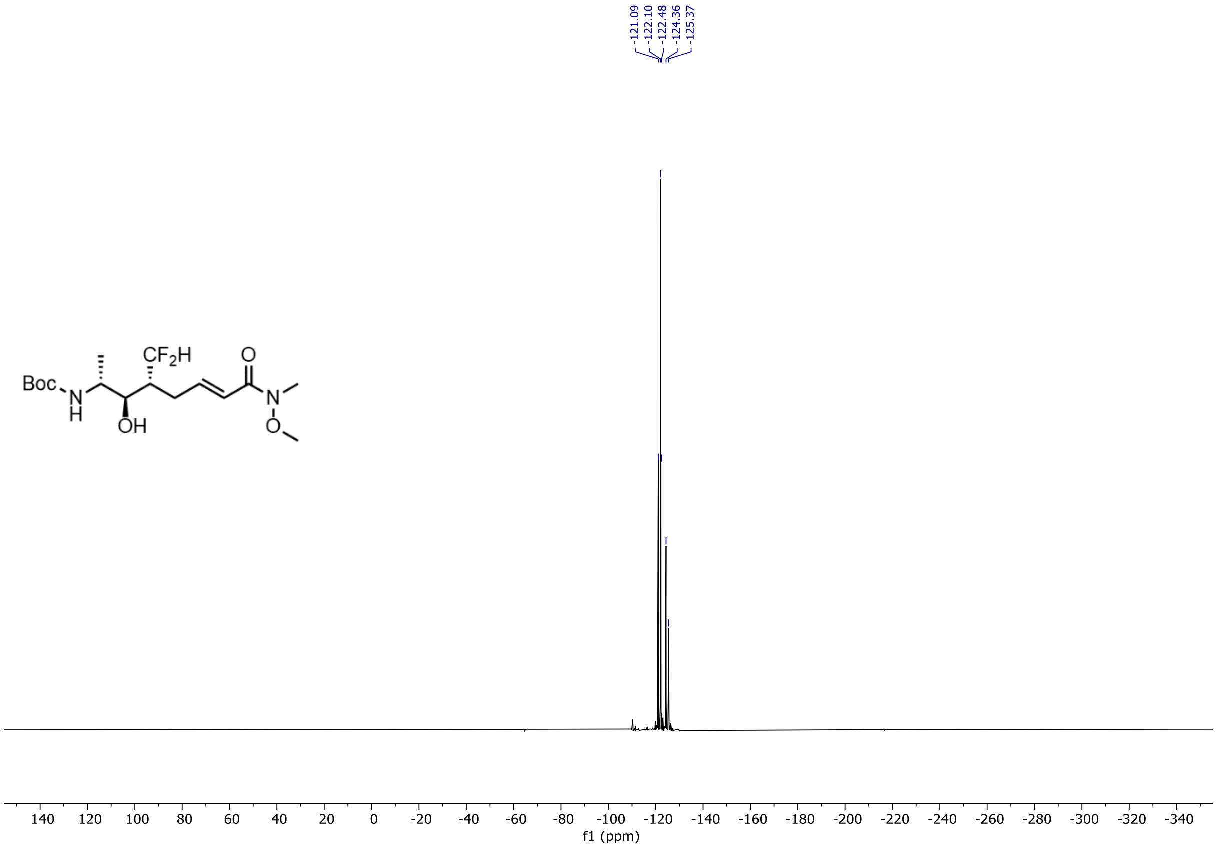

-121.09
-122.10
-122.48
-124.36
-125.37
Boc
N
H
CF2H
OH
O
N
O
140 120 100 80 60 40 20 0 -20 -40 -60 -80 -100 -120 -140 -160 -180 -200 -220 -240 -260 -280 -300 -320 -340
f1 (ppm)

^{1}H NMR (400 MHz, CD_2Cl_2; top), ^{13}C NMR (101 MHz, $CDCl_3$; middle) and ^{19}F NMR (282 MHz, CD_2Cl_2) of compound **20a**

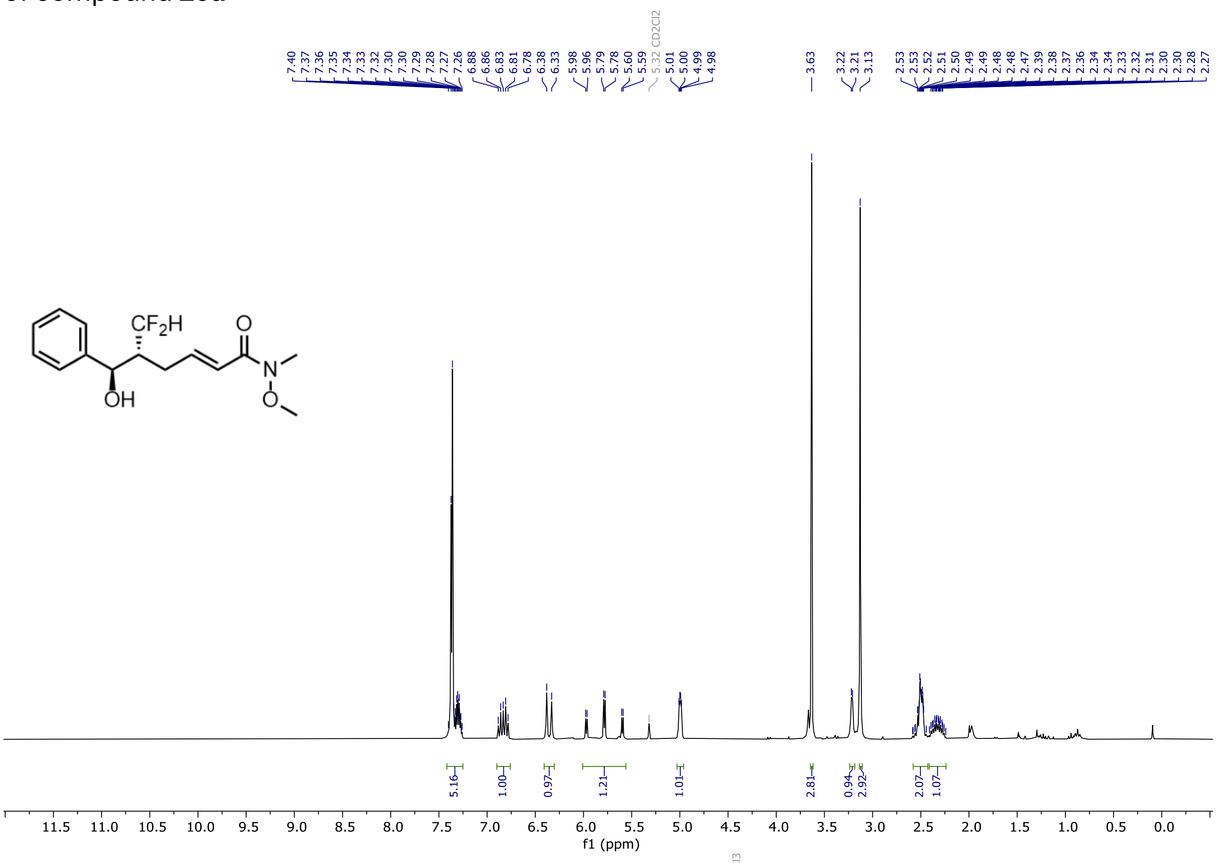

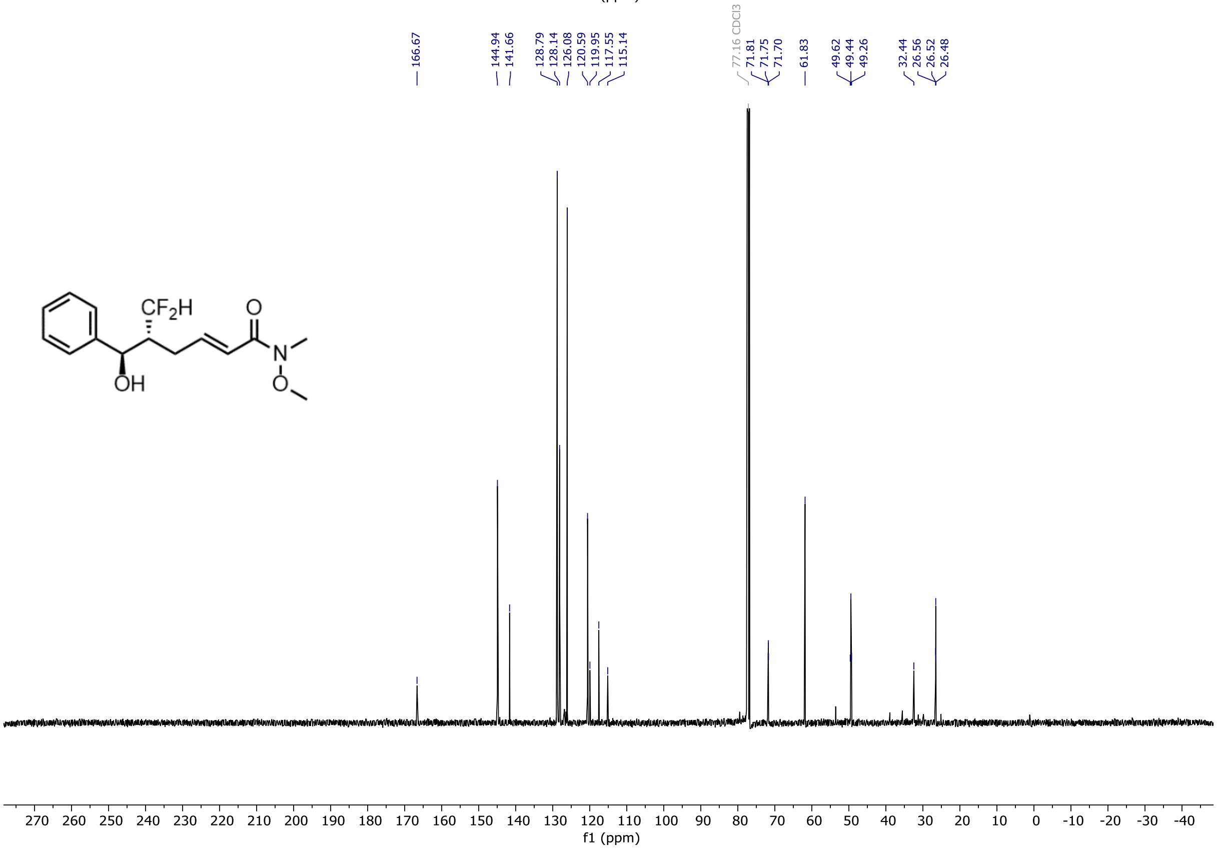

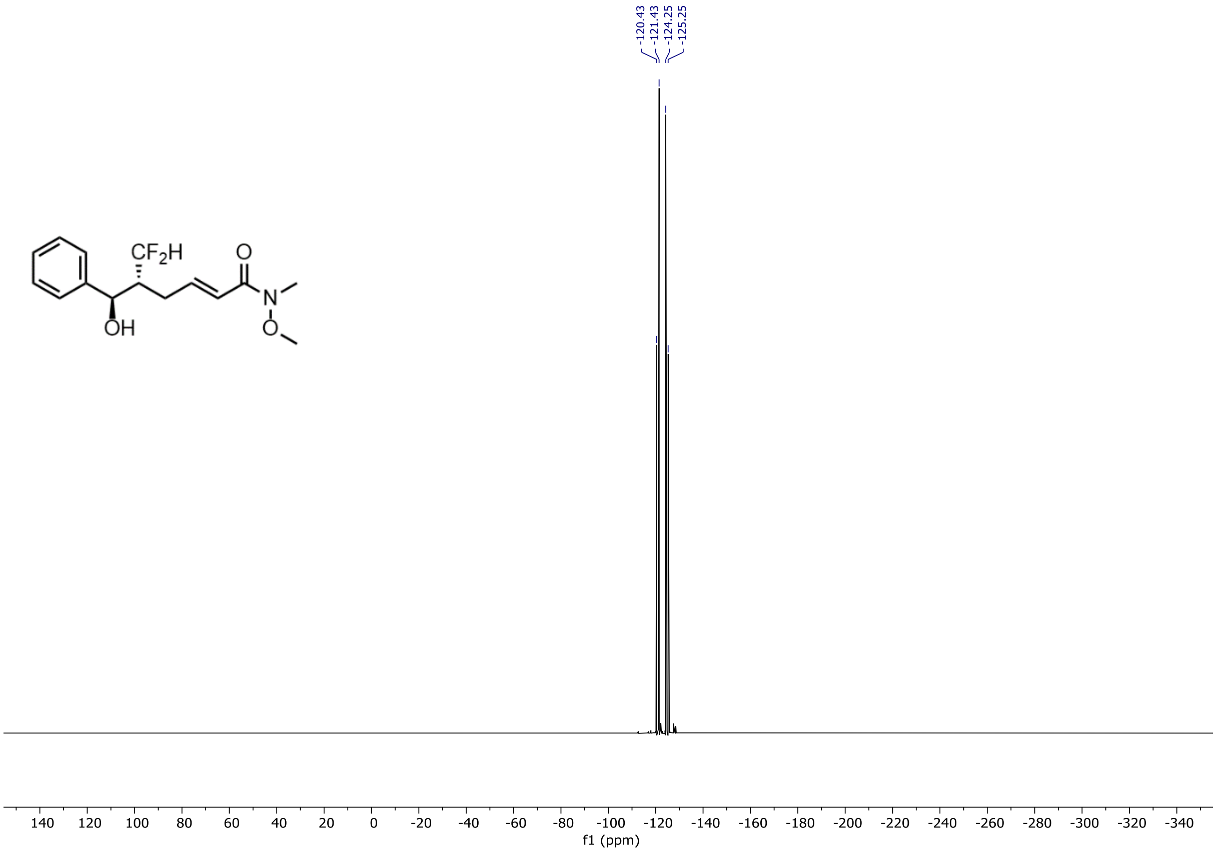

-120.43
-121.43
-124.25
-125.25
CF2H
O
N
O
OH
140 120 100 80 60 40 20 0 -20 -40 -60 -80 -100 -120 -140 -160 -180 -200 -220 -240 -260 -280 -300 -320 -340
f1 (ppm)

^{1}H NMR (400 MHz, CD_2Cl_2; top), ^{13}C NMR (101 MHz, CD_2Cl_2; middle) and ^{19}F NMR (282 MHz, CD_2Cl_2) of compound **20b**

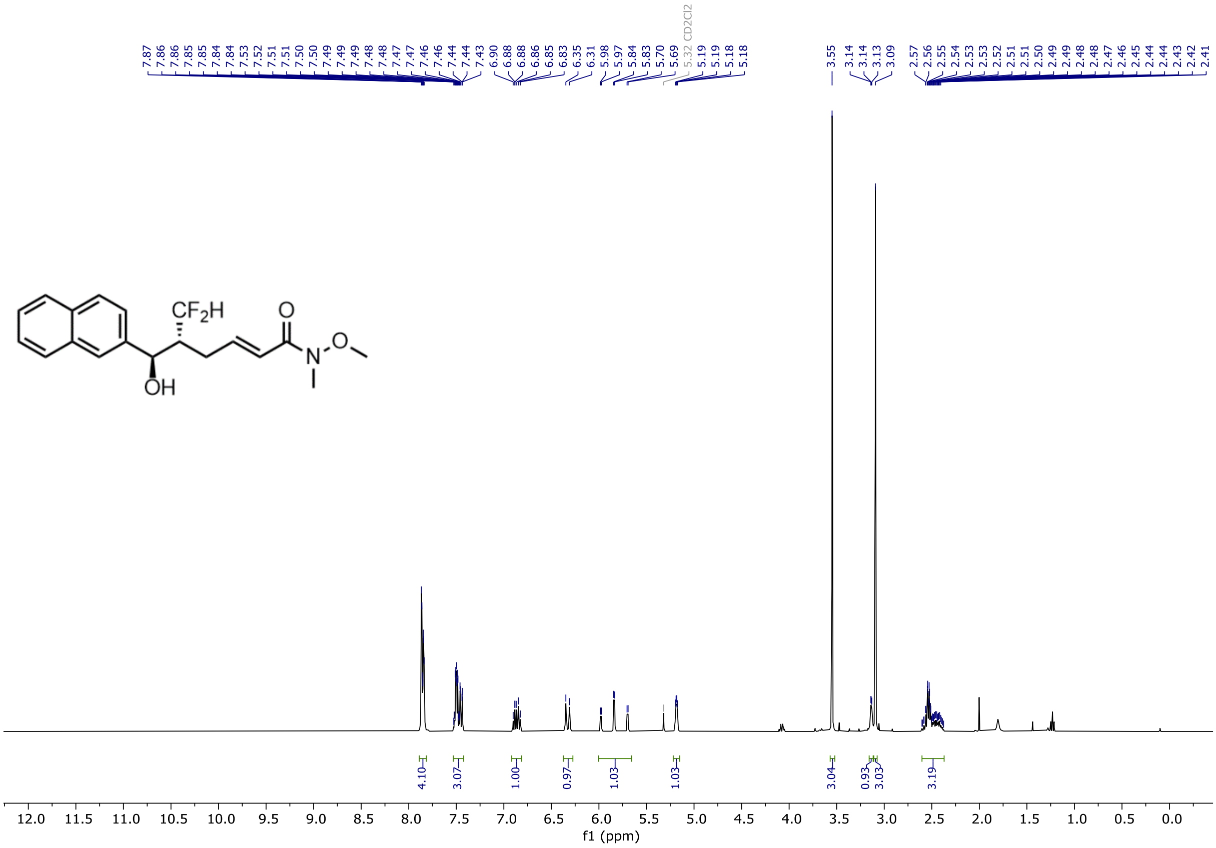

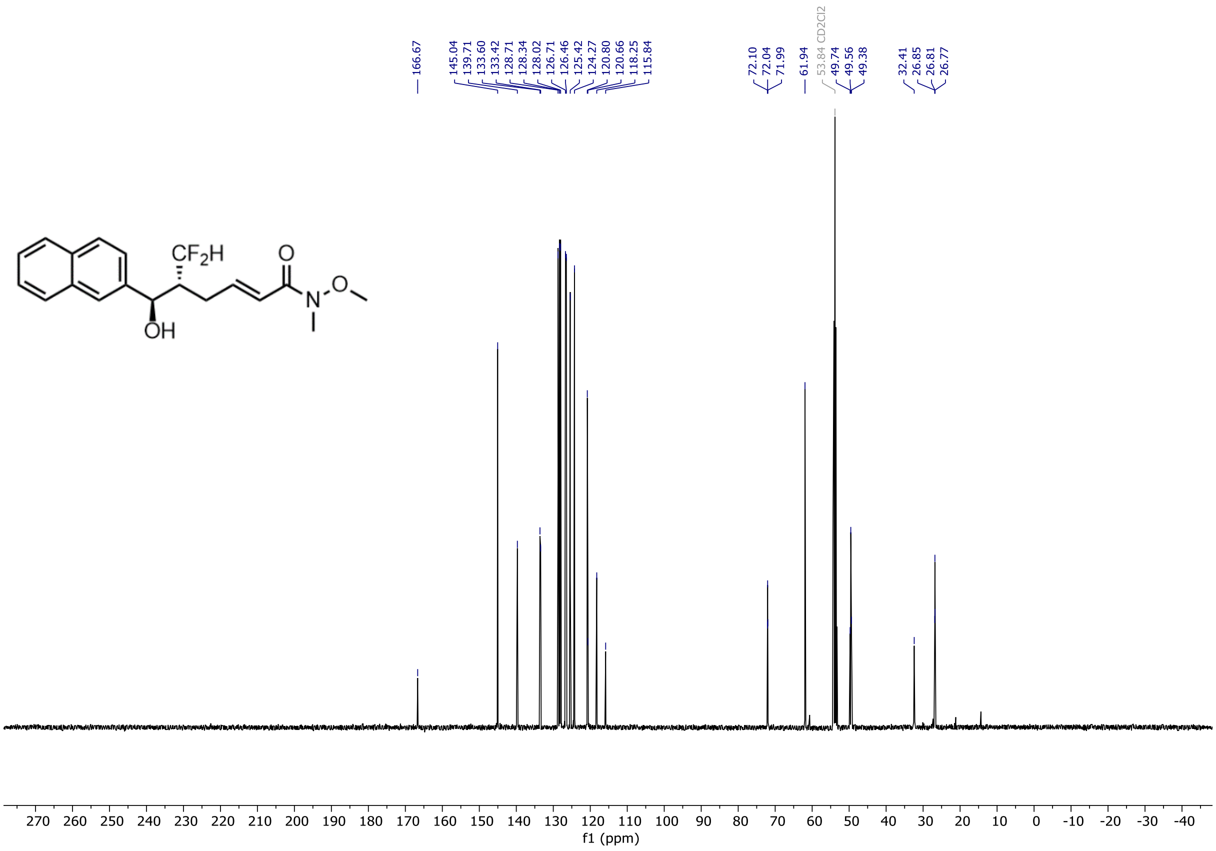

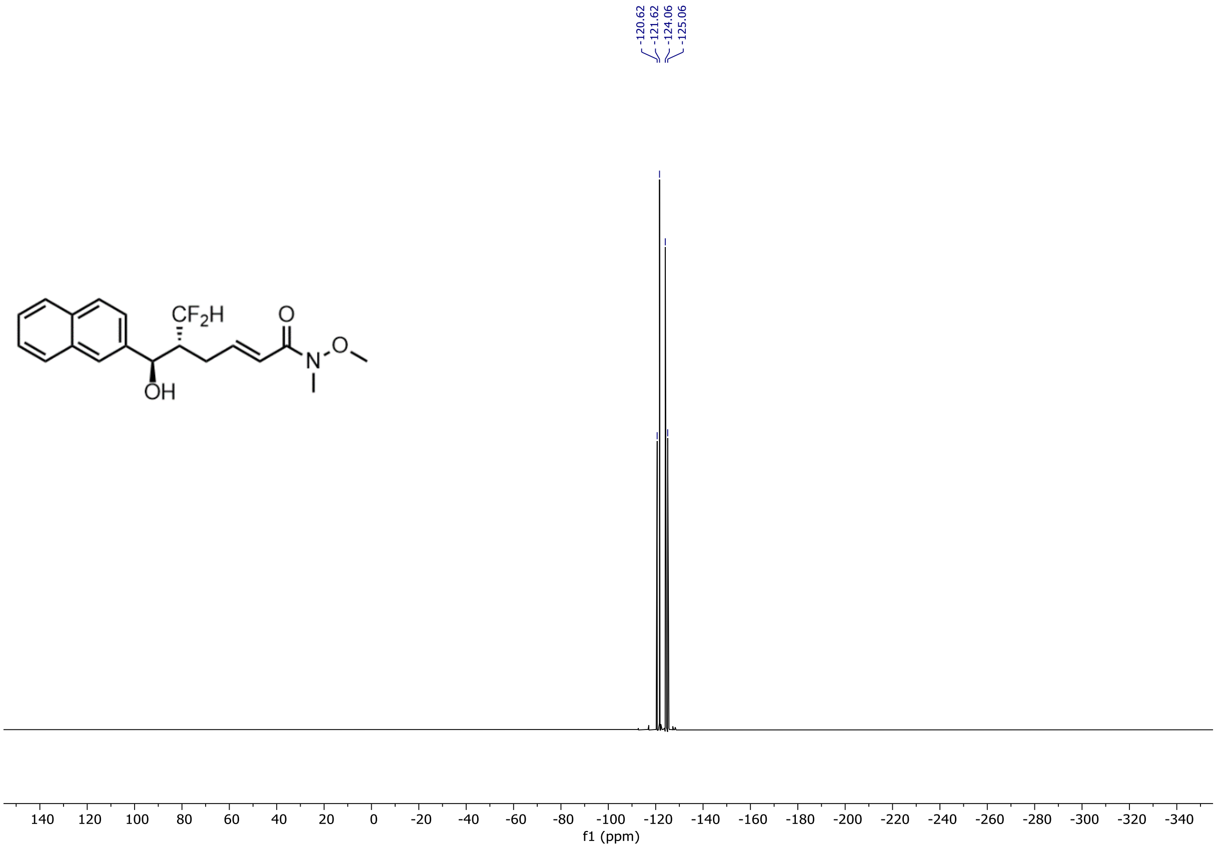
-120.62
-121.62
-124.06
-125.06
CF2H
O
N
O
OH
140 120 100 80 60 40 20 0 -20 -40 -60 -80 -100 -120 -140 -160 -180 -200 -220 -240 -260 -280 -300 -320 -340
f1 (ppm)

^1H NMR (400 MHz, CD_2Cl_2; top), ^{13}C NMR (101 MHz, CD_2Cl_2; middle) and ^{19}F NMR (282 MHz, CD_2Cl_2) of compound **20c** [Note: due to high viscosity of the isolated compound, residual cyclohexane (≈ 5 %) is visible in the ^1H NMR spectrum].

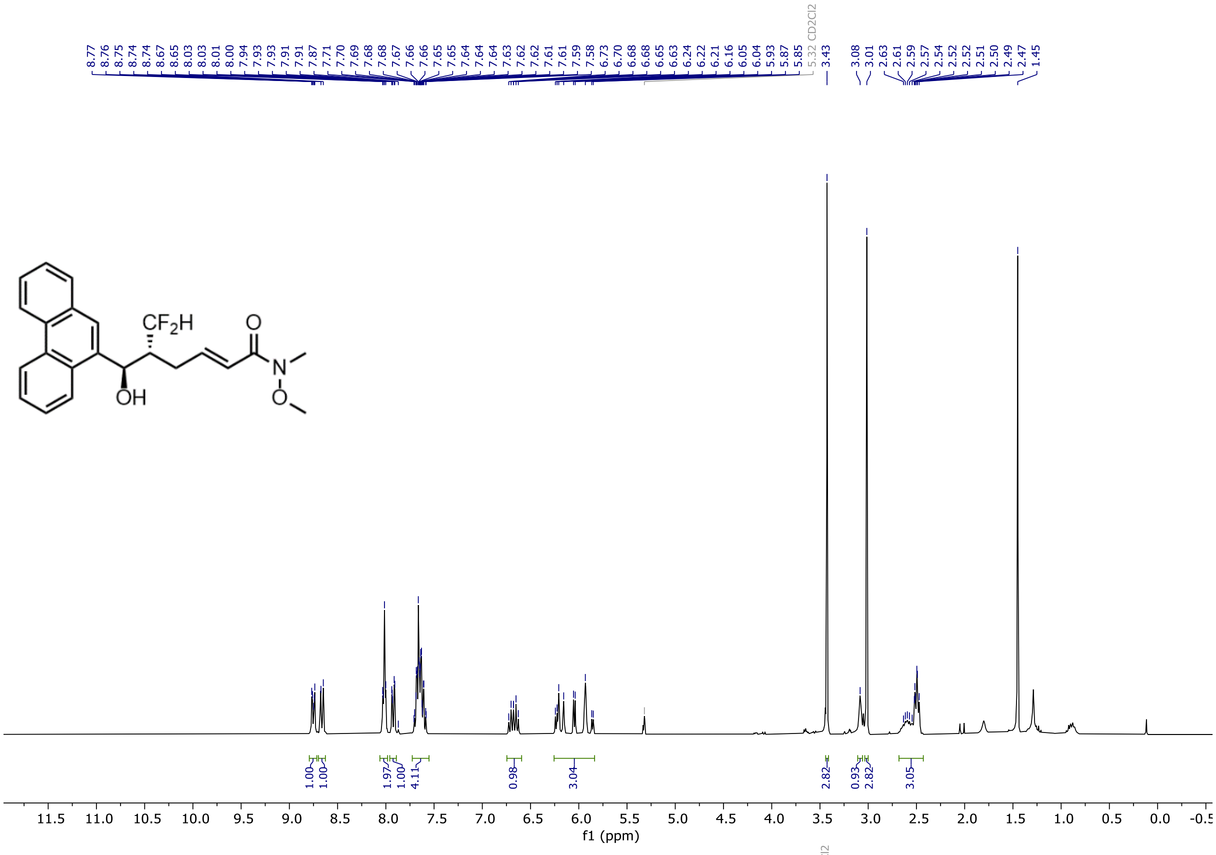

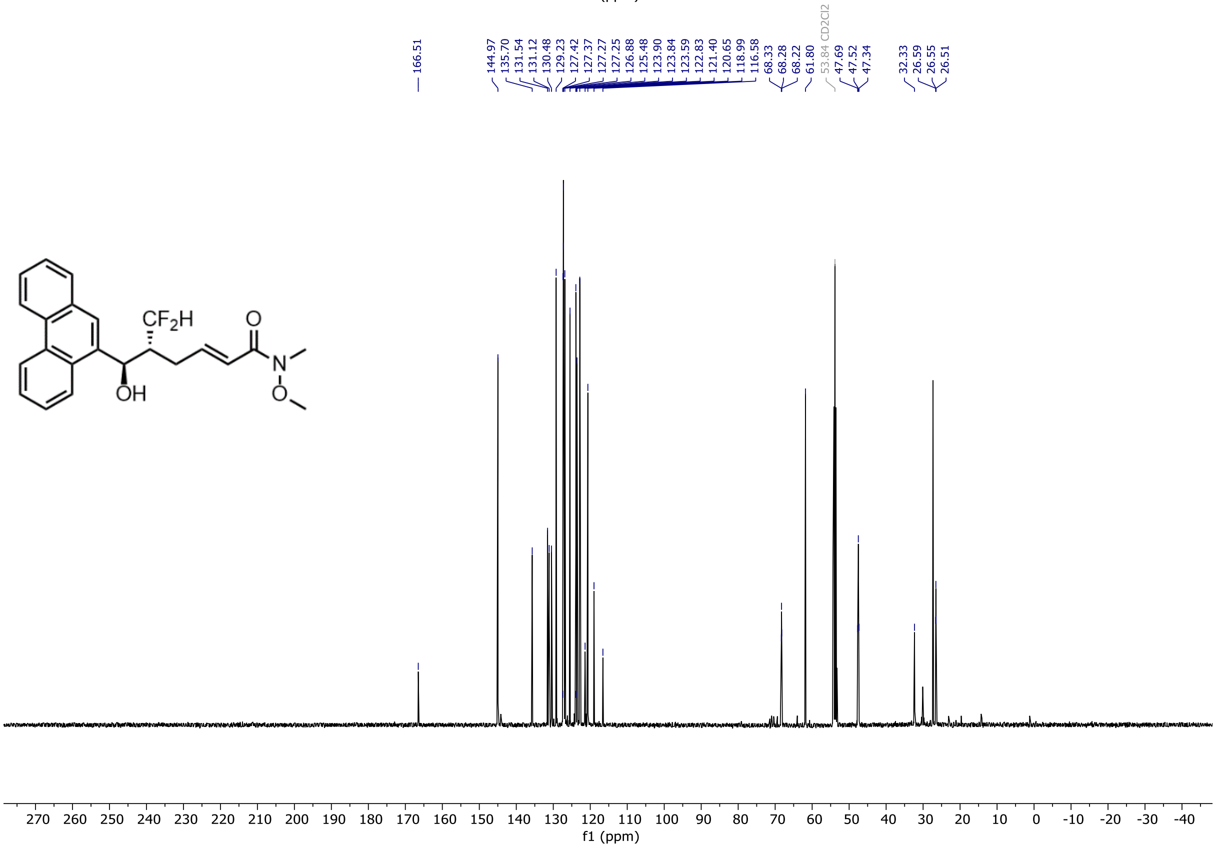

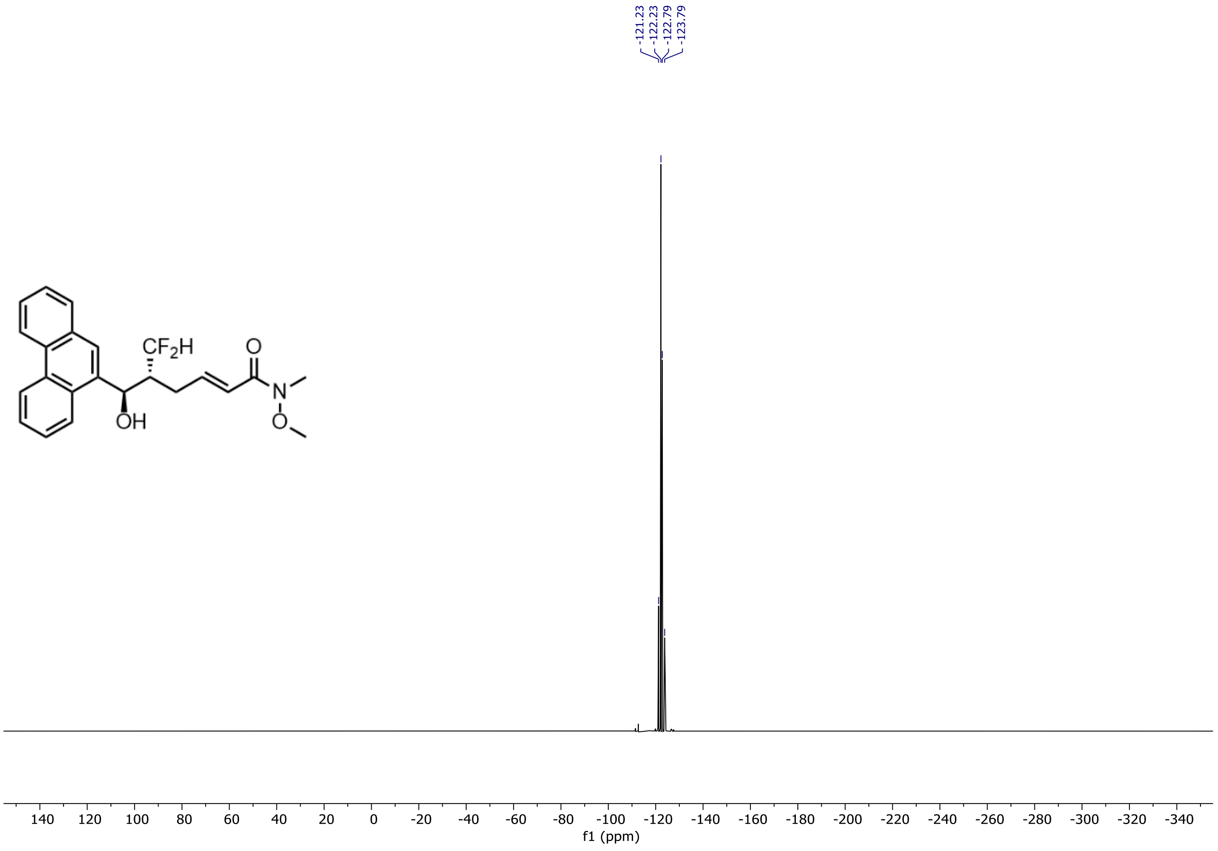
-121.23
-122.23
-122.79
-123.79
CF2H
O
N
O
OH
140
120
100
80
60
40
20
0
-20
-40
-60
-80
-100
-120
-140
-160
-180
-200
-220
-240
-260
-280
-300
-320
-340
f1 (ppm)

^{1}H NMR (400 MHz, CD_2Cl_2; top), ^{13}C NMR (101 MHz, CD_2Cl_2; middle) and ^{19}F NMR (282 MHz, CD_2Cl_2) of compound **20d** [Note: due to high viscosity of the isolated compound, residual cyclohexane (≈ 5 %) is visible in the ^{1}H NMR spectrum]

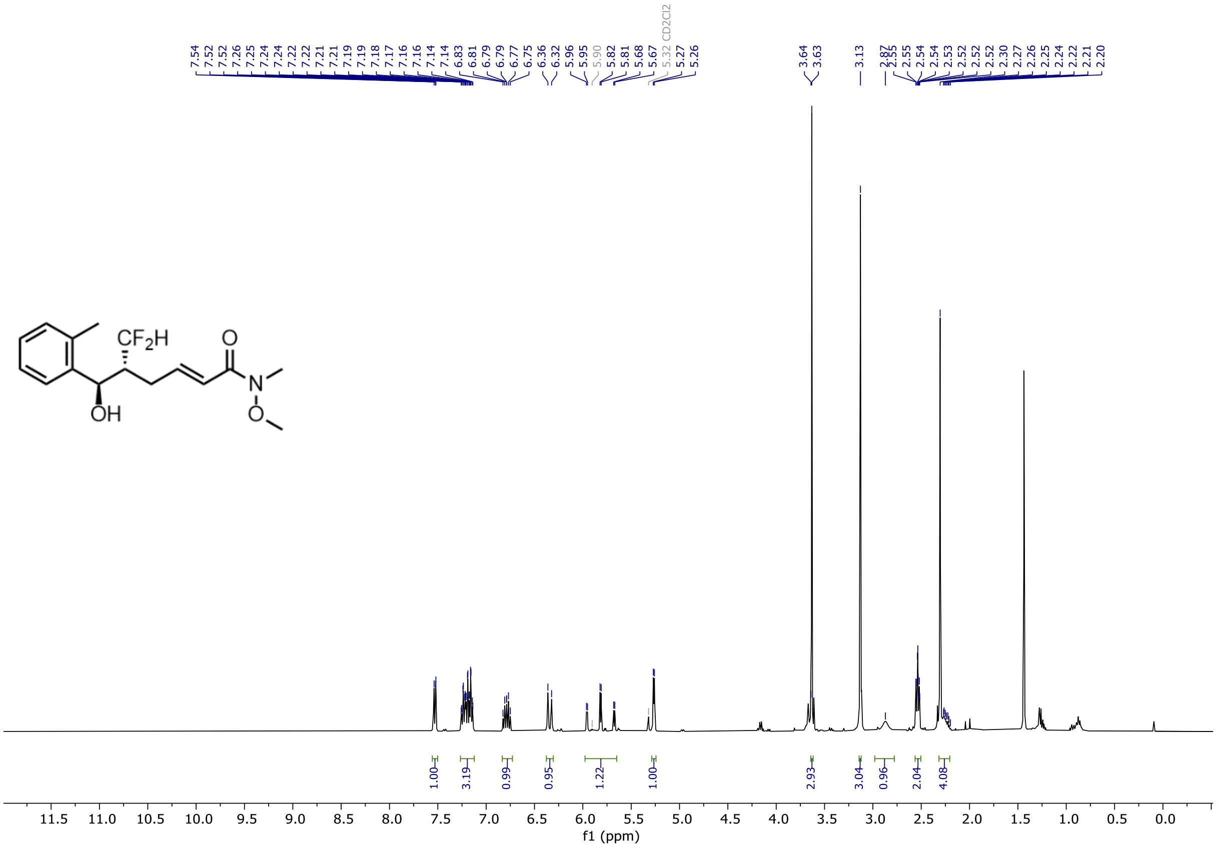

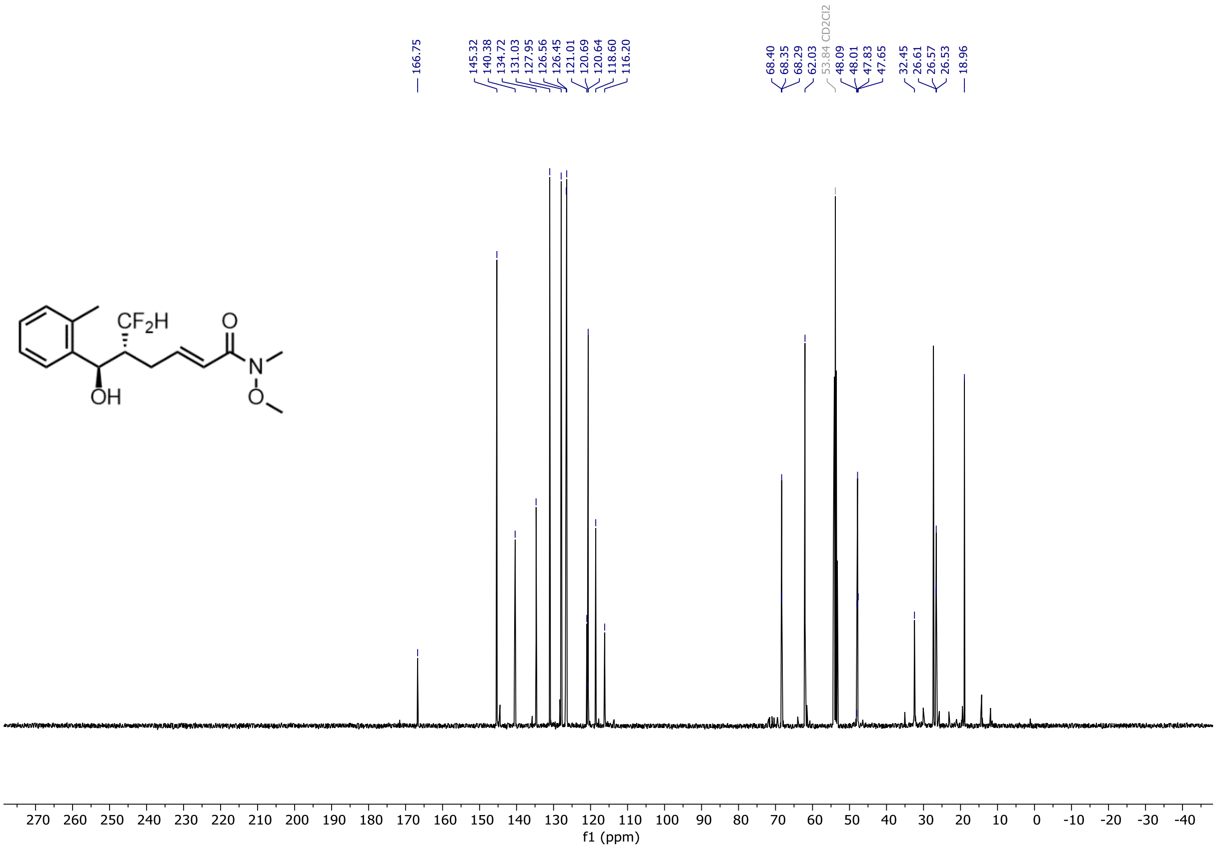

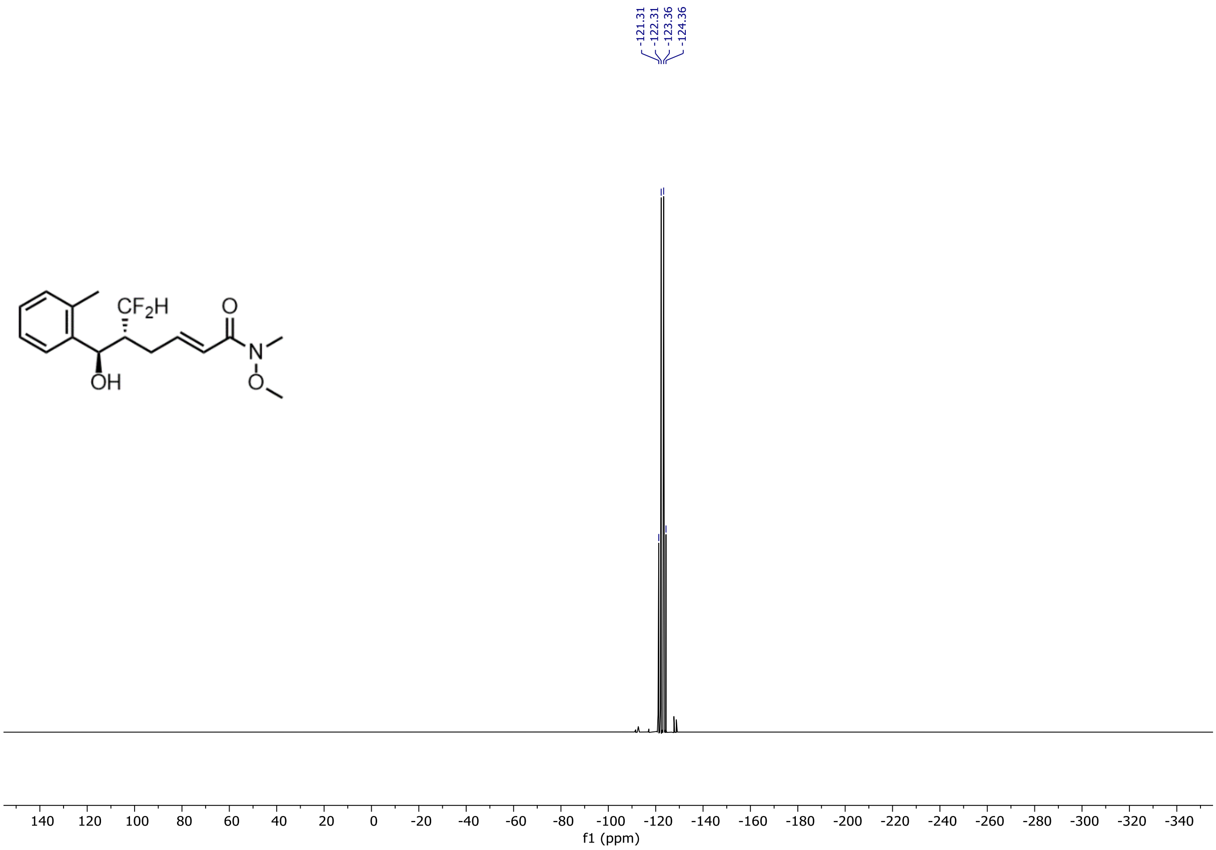
CF2H
O
N
O
OH
-121.31
-122.31
-123.36
-124.36
140 120 100 80 60 40 20 0 -20 -40 -60 -80 -100 -120 -140 -160 -180 -200 -220 -240 -260 -280 -300 -320 -340
f1 (ppm)

^{1}H NMR (400 MHz, CD_2Cl_2; top), ^{13}C NMR (101 MHz, CD_2Cl_2; middle) and ^{19}F NMR (282 MHz, CD_2Cl_2) of compound **20e**

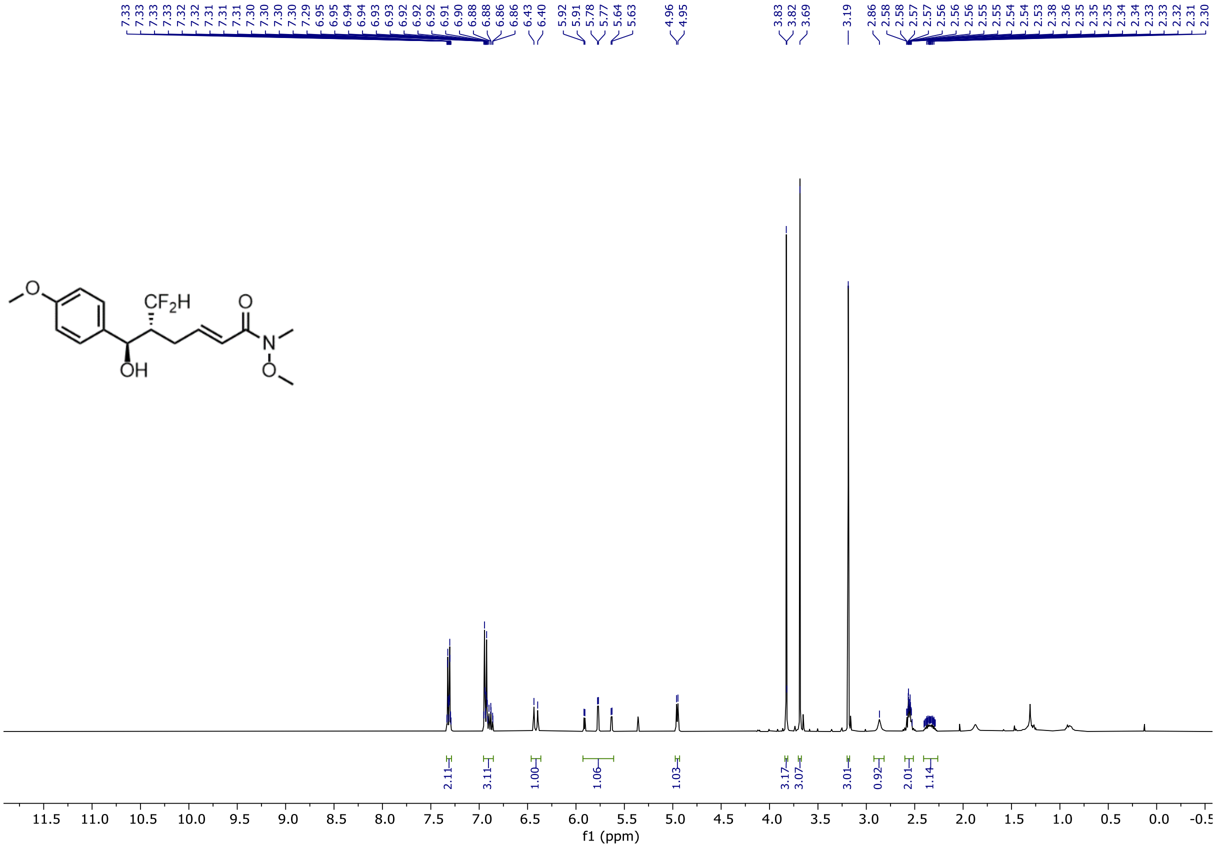

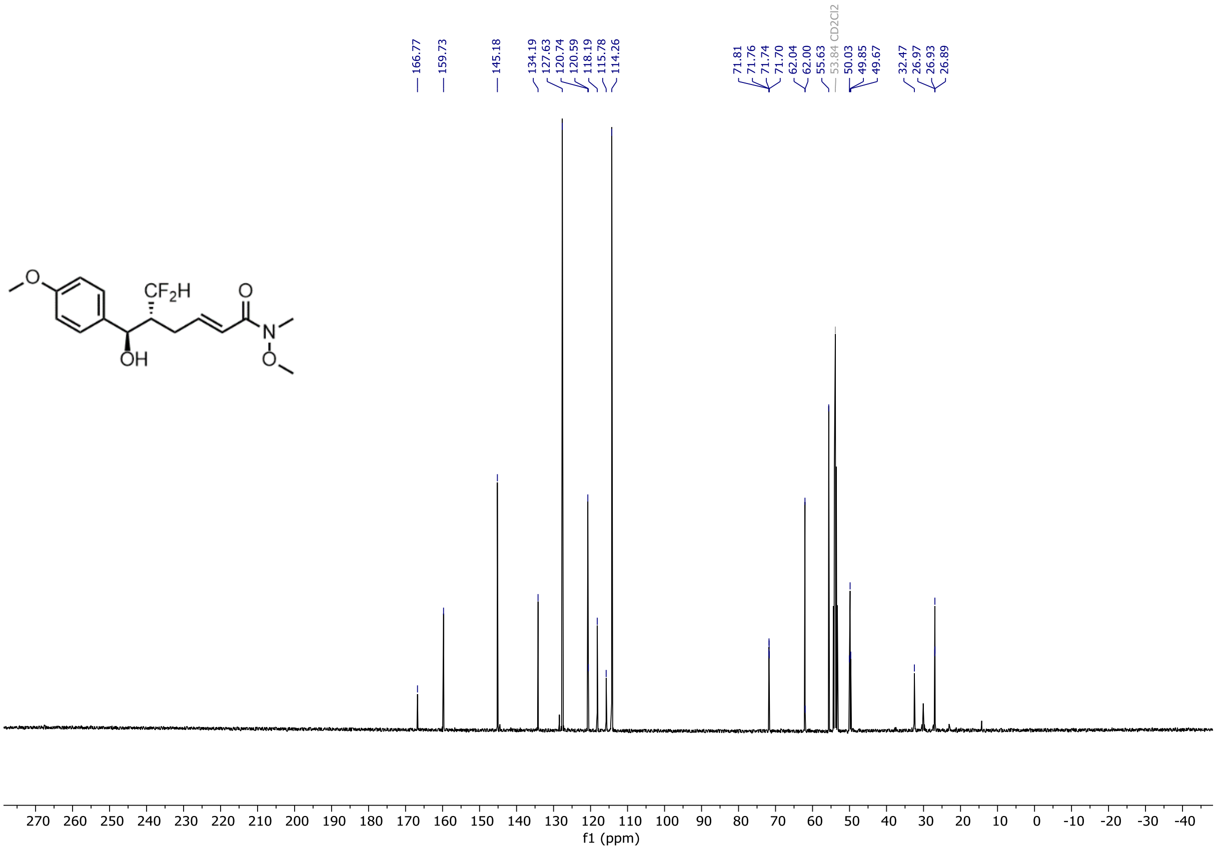

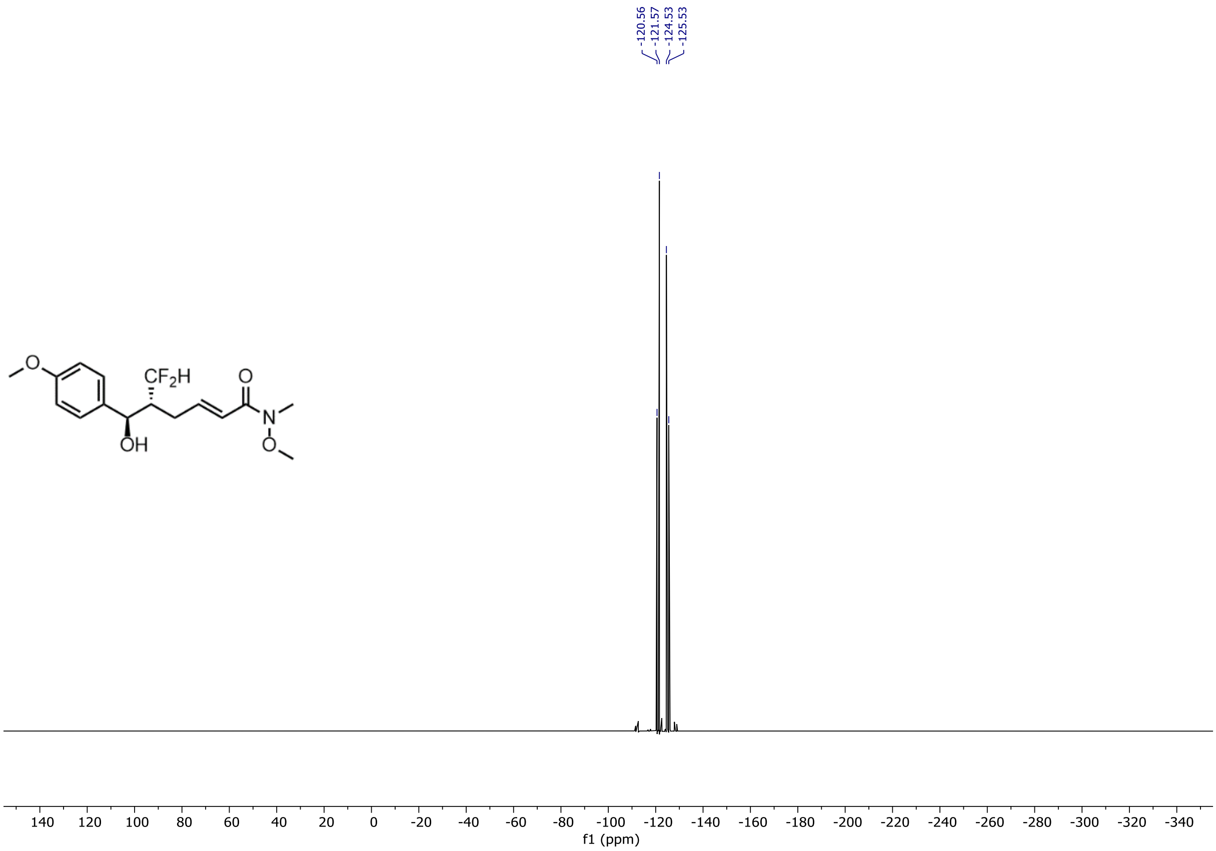

CF2H
O
N
O
OH
-120.56
-121.57
-124.53
-125.53
140 120 100 80 60 40 20 0 -20 -40 -60 -80 -100 -120 -140 -160 -180 -200 -220 -240 -260 -280 -300 -320 -340
f1 (ppm)

^{1}H NMR (400 MHz, CD_2Cl_2; top), ^{13}C NMR (101 MHz, CD_2Cl_2; middle) and ^{19}F NMR (282 MHz, CD_2Cl_2) of compound **20f**

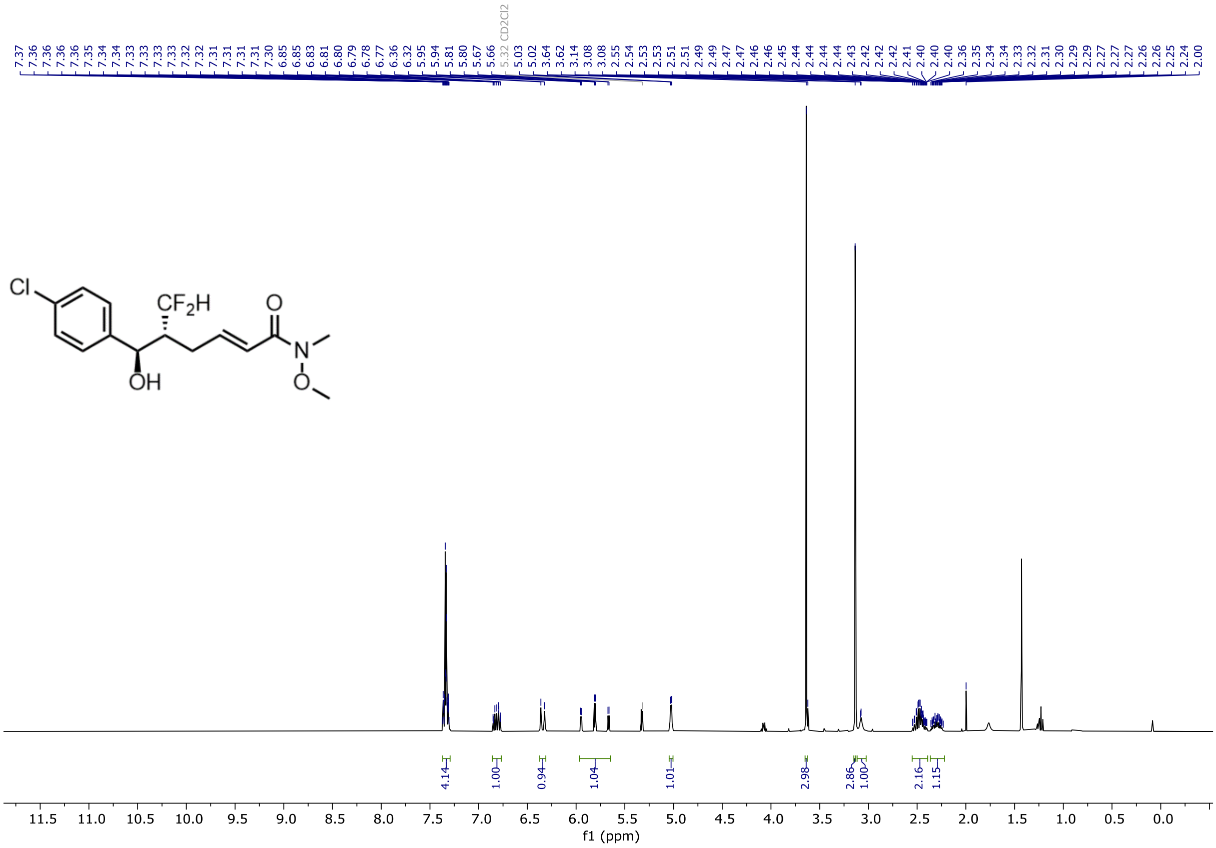

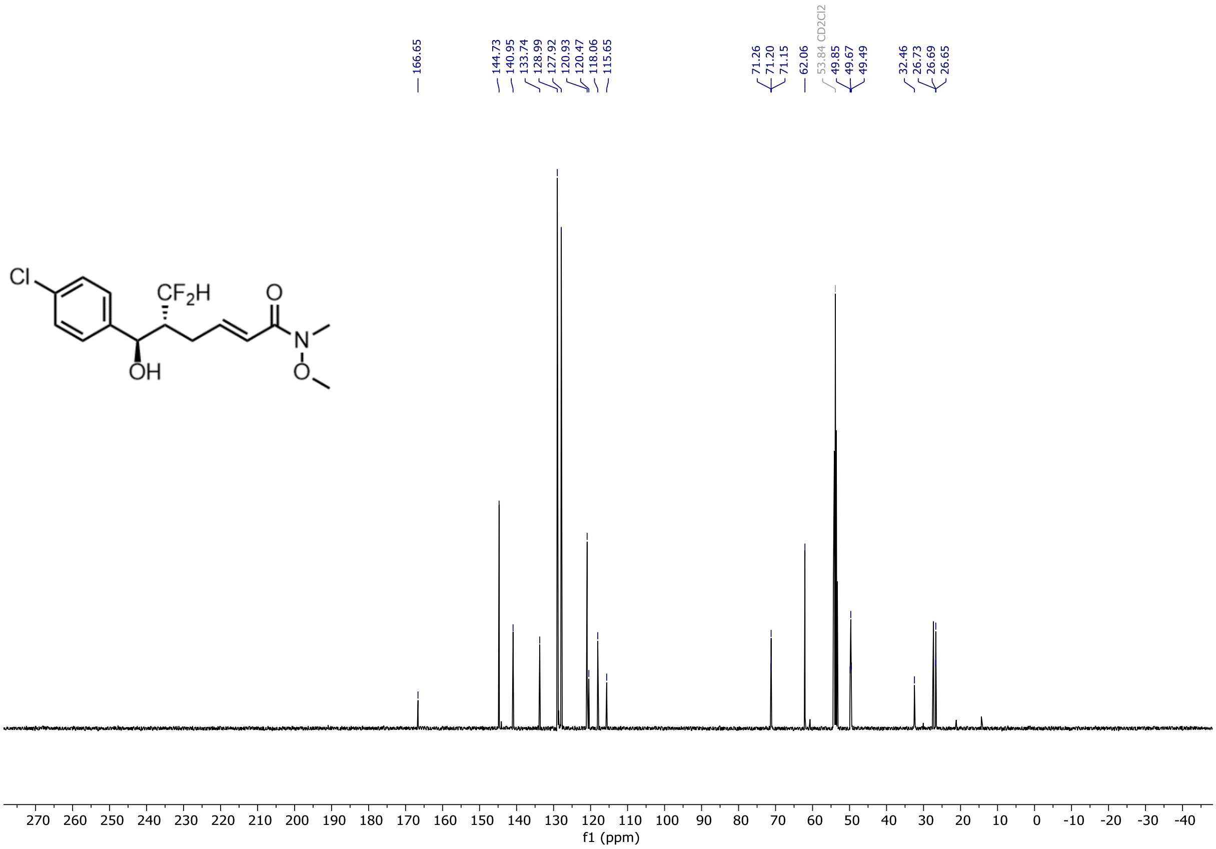

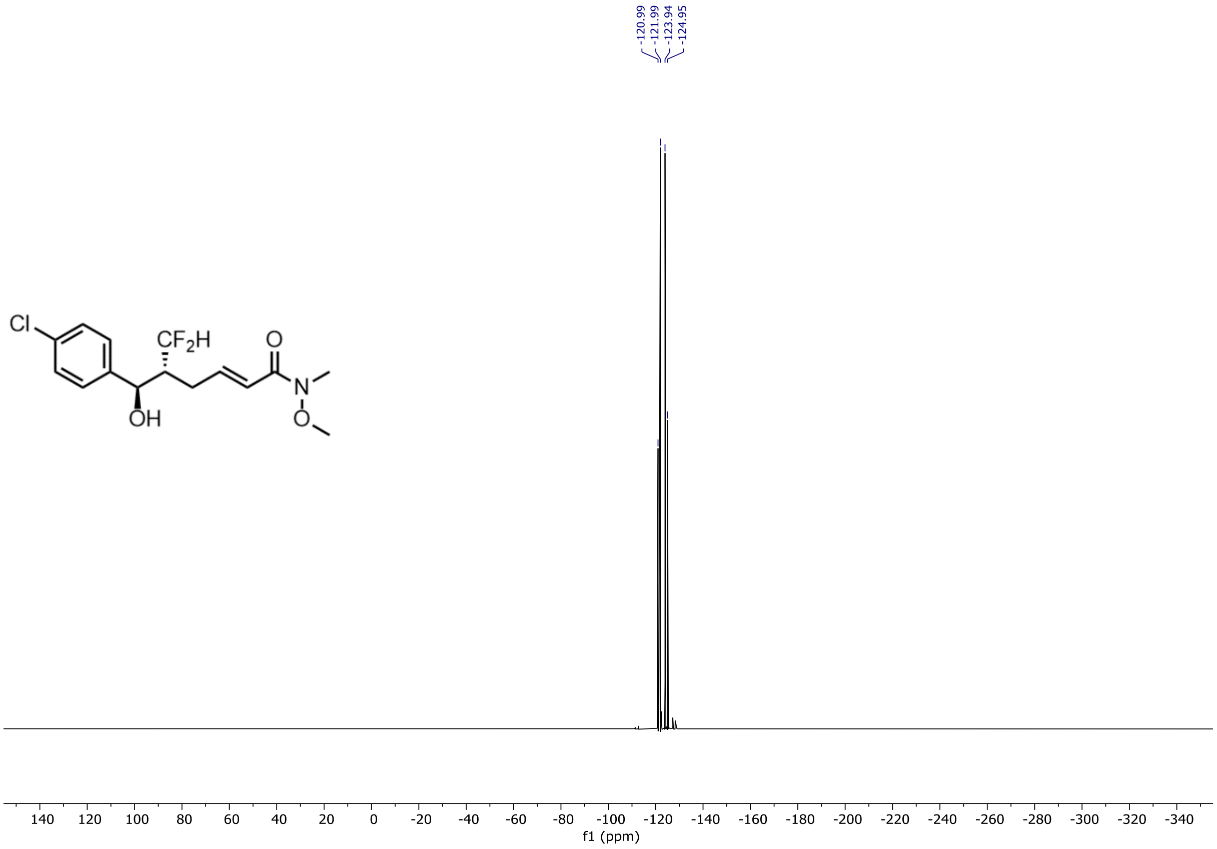
-120.99
-121.99
-123.94
-124.95
Cl
CF2H
O
N
OH
O
140 120 100 80 60 40 20 0 -20 -40 -60 -80 -100 -120 -140 -160 -180 -200 -220 -240 -260 -280 -300 -320 -340
f1 (ppm)

^{1}H NMR (400 MHz, CD_2Cl_2; top), ^{13}C NMR (101 MHz, CD_2Cl_2; middle) and ^{19}F NMR (282 MHz, CD_2Cl_2) of compound **20g**

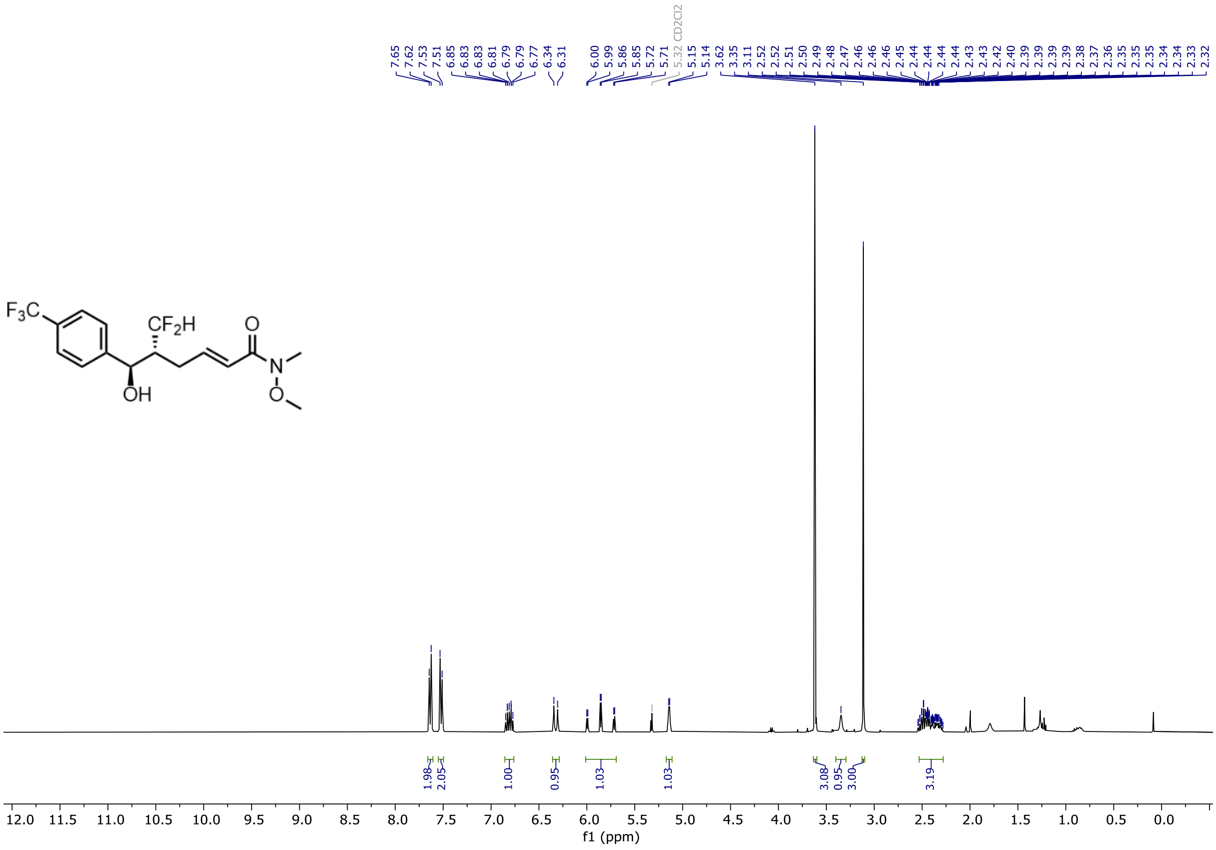

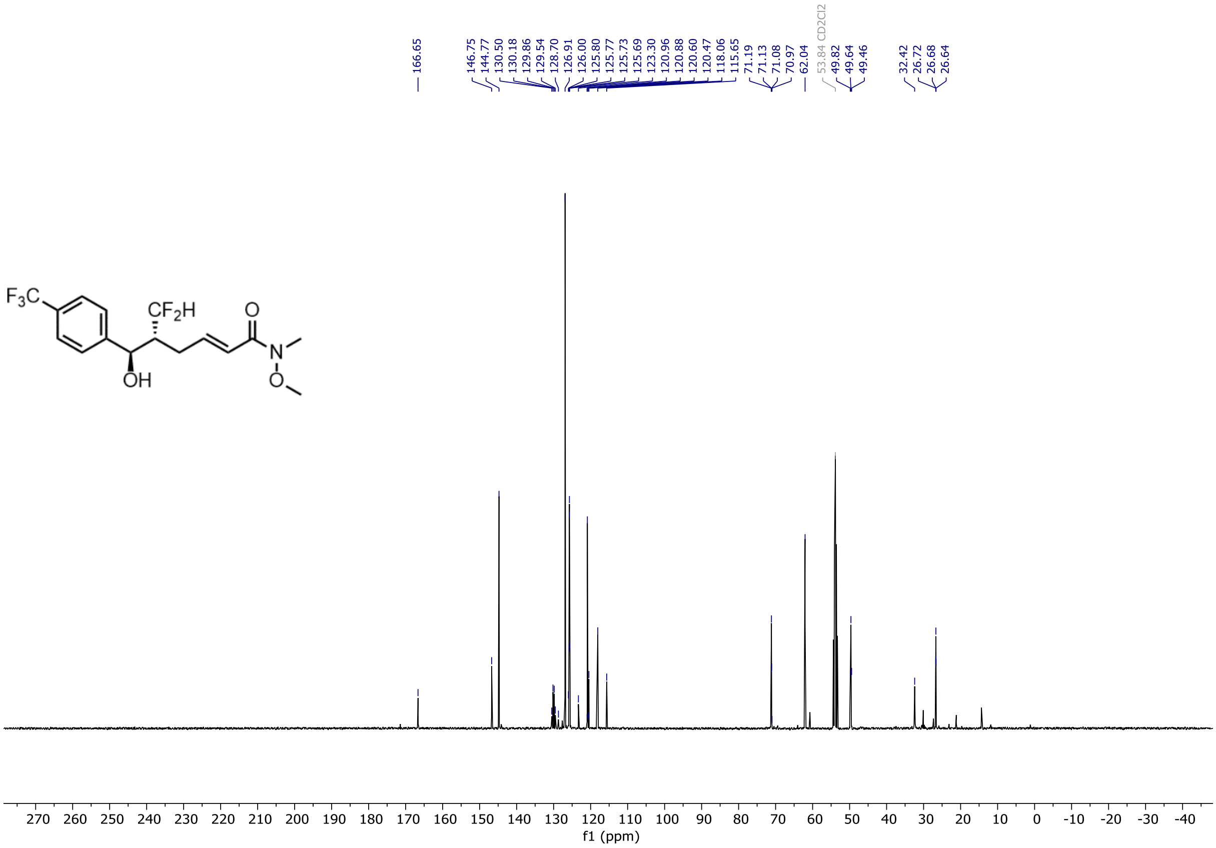

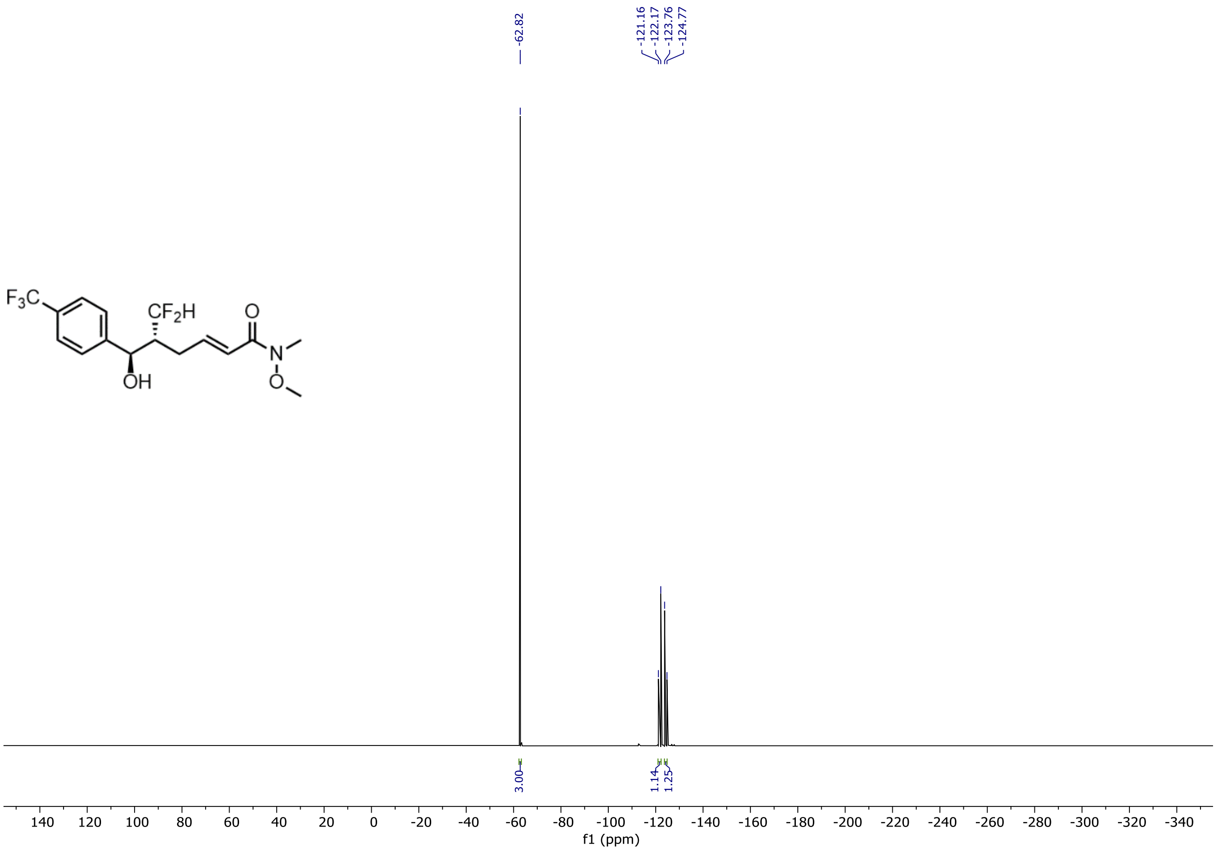

-62.82
-121.16
-122.17
-123.76
-124.77
3.00
1.14
1.25
140 120 100 80 60 40 20 0 -20 -40 -60 -80 -100 -120 -140 -160 -180 -200 -220 -240 -260 -280 -300 -320 -340
f1 (ppm)

^{1}H NMR (400 MHz, CD_2Cl_2; top), ^{13}C NMR (101 MHz, CD_2Cl_2; middle) and ^{19}F NMR (282 MHz, CD_2Cl_2) of compound **20h** [Note: due to high viscosity of the isolated compound, residual cyclohexane (≈ 5 %) is visible in the ^{1}H NMR spectrum]

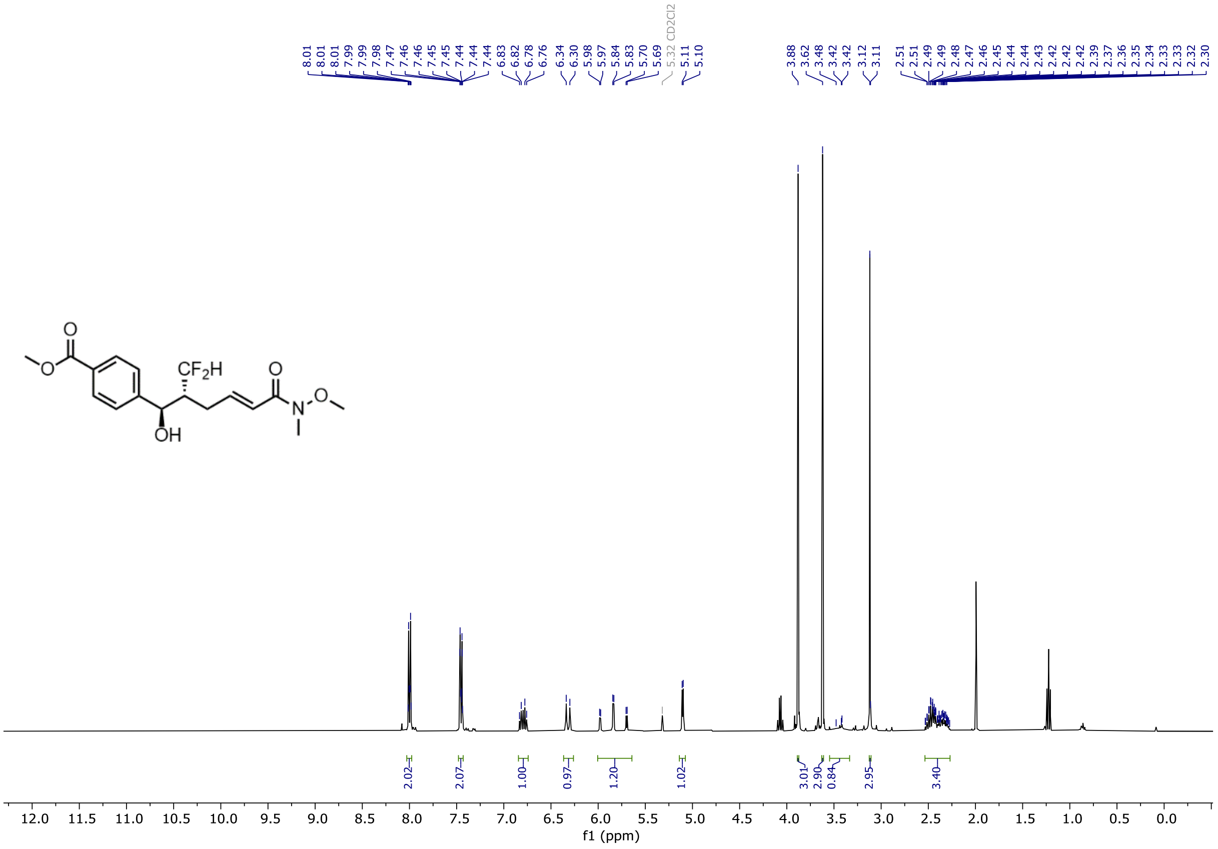

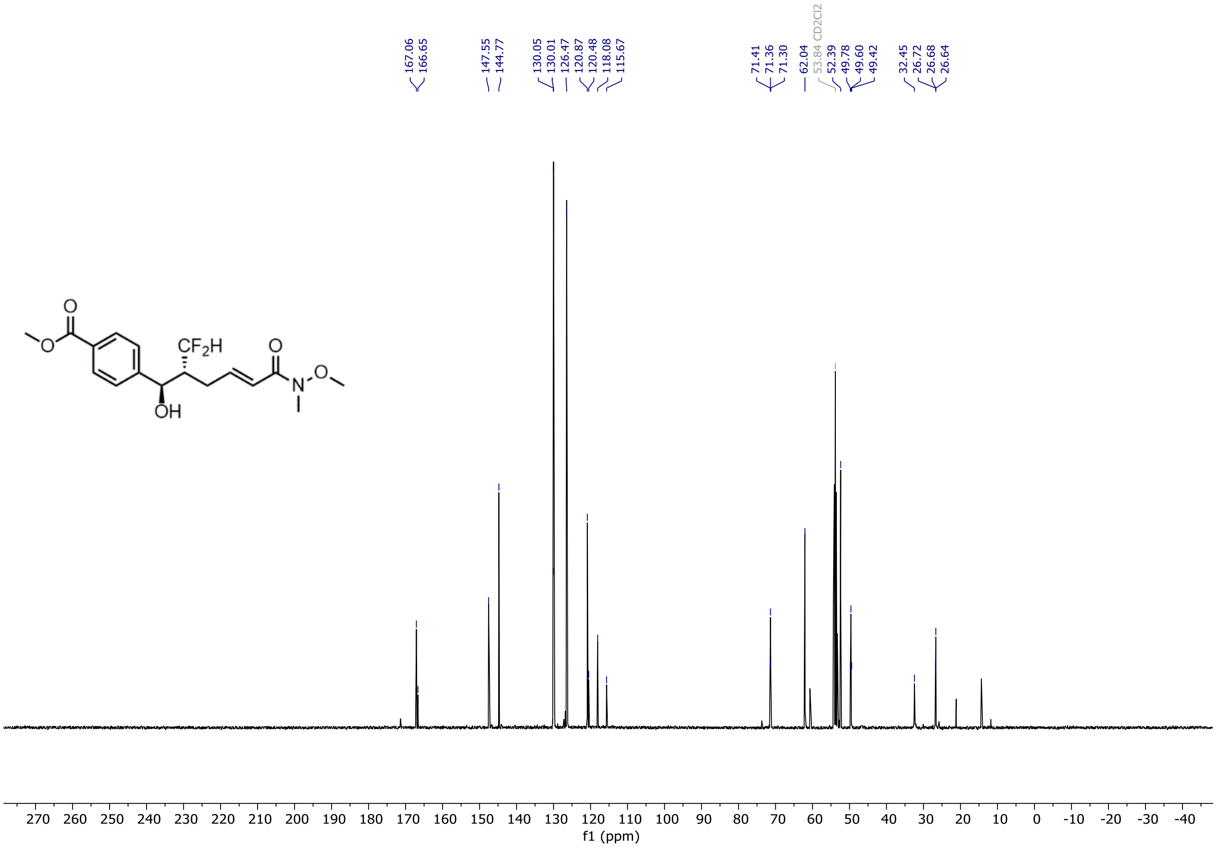

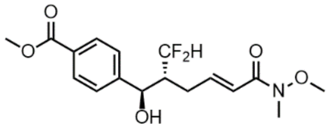

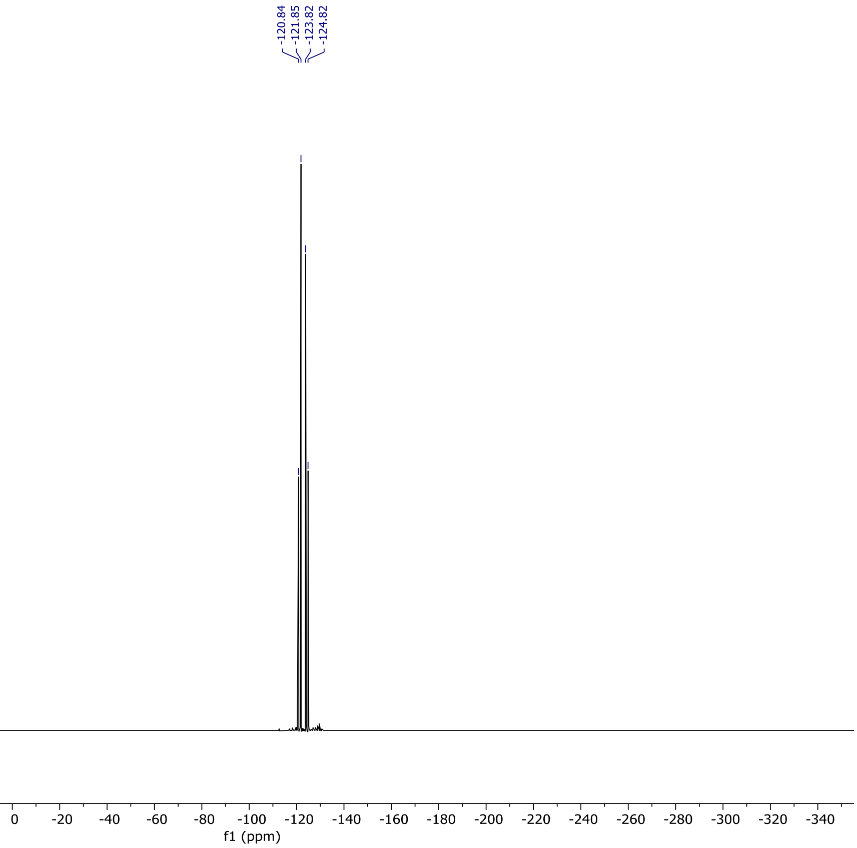

-120.84
-121.85
-123.82
-124.82
140 120 100 80 60 40 20 0 -20 -40 -60 -80 -100 -120 -140 -160 -180 -200 -220 -240 -260 -280 -300 -320 -340
f1 (ppm)

^{1}H NMR (400 MHz, CD_2Cl_2; top), ^{13}C NMR (101 MHz, CD_2Cl_2; middle) and ^{19}F NMR (282 MHz, CD_2Cl_2) of compound **20i** [Note: due to high viscosity of the isolated compound, residual cyclohexane (≈ 5 %) is visible in the ^{1}H NMR spectrum]

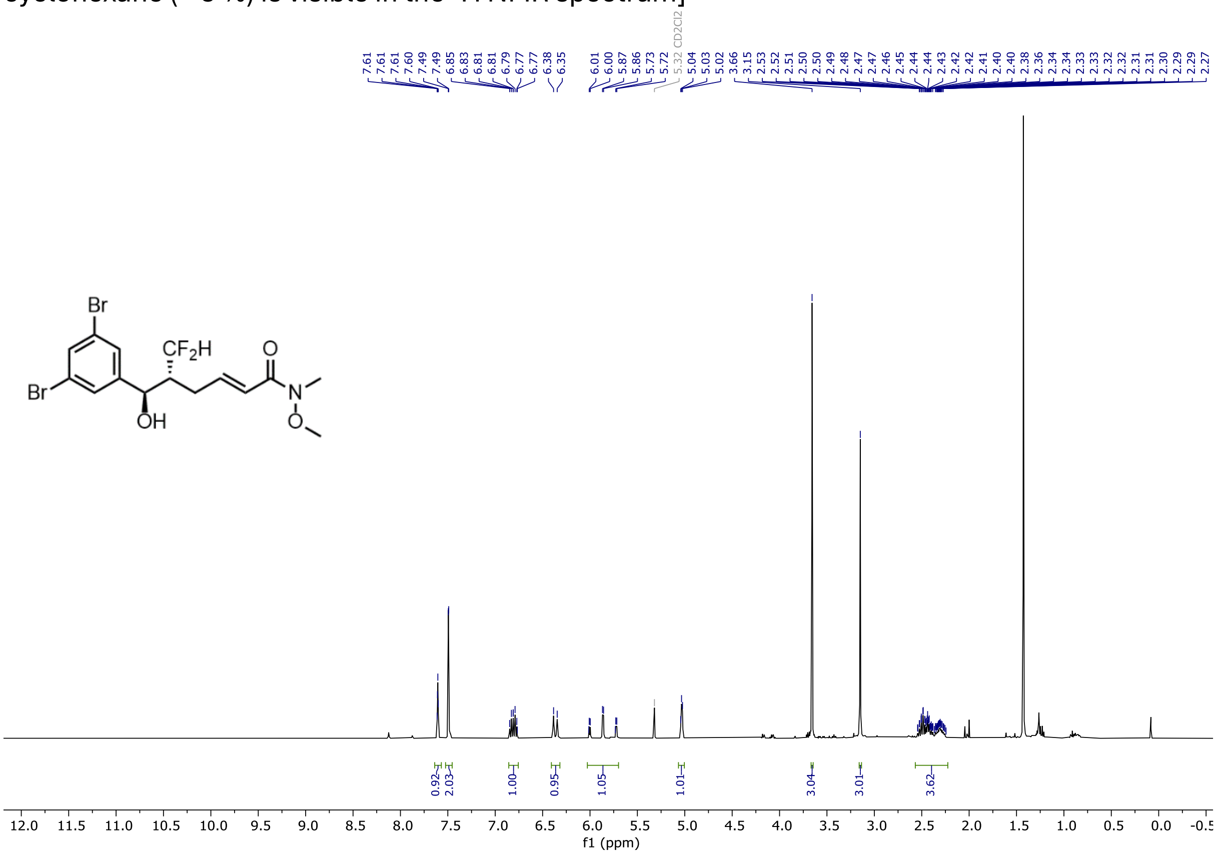

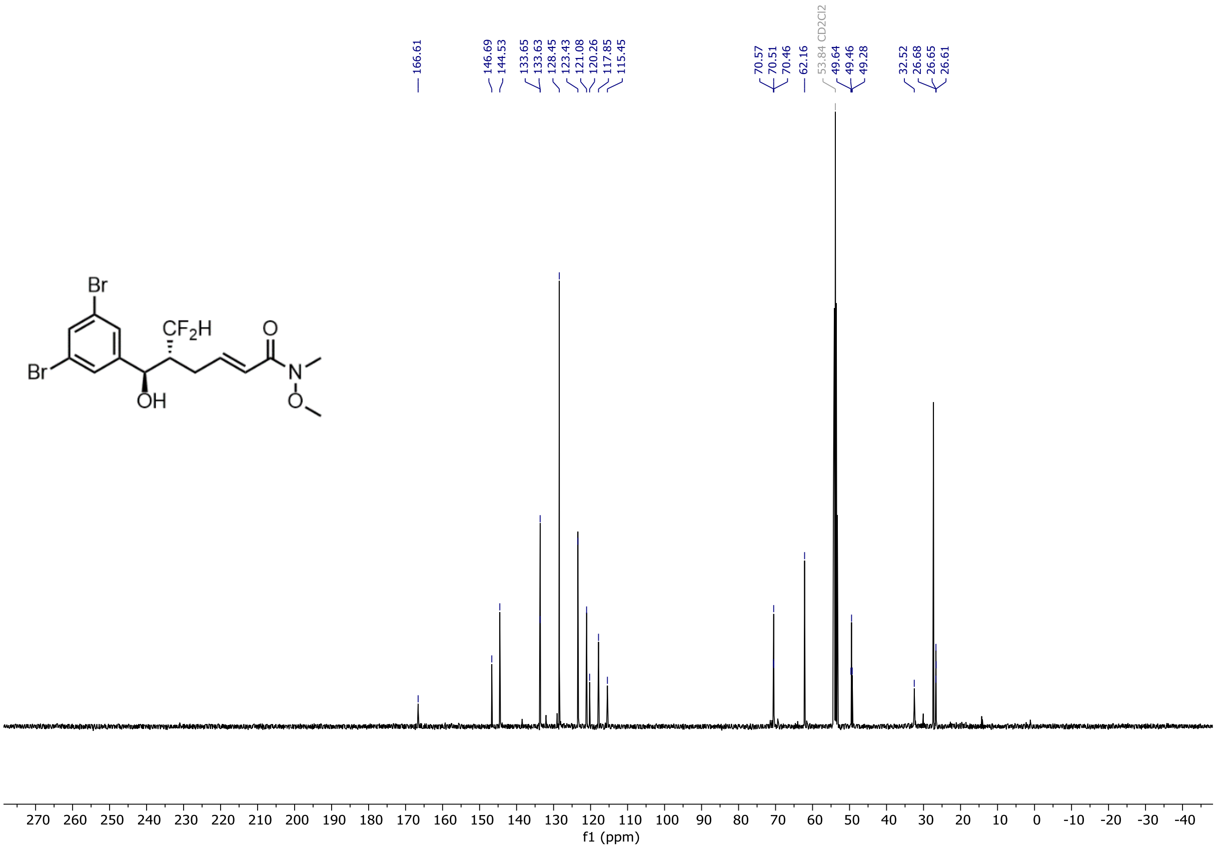

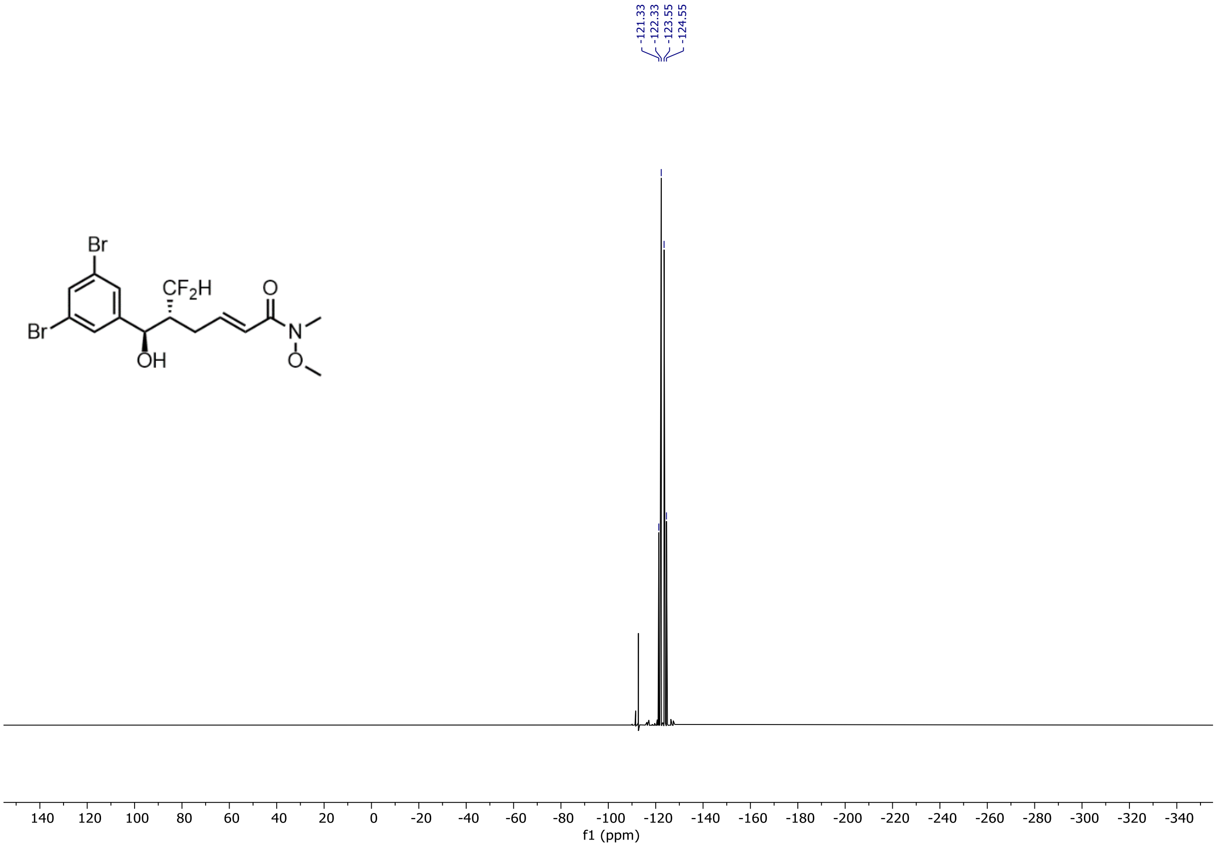
-121.33
-122.33
-123.55
-124.55
Br
CF2H
O
Br
N
OH
O
140 120 100 80 60 40 20 0 -20 -40 -60 -80 -100 -120 -140 -160 -180 -200 -220 -240 -260 -280 -300 -320 -340
f1 (ppm)

^{1}H NMR (400 MHz, CD_2Cl_2; top), ^{13}C NMR (101 MHz, CD_2Cl_2; middle) and ^{19}F NMR (282 MHz, CD_2Cl_2) of compound **20j**

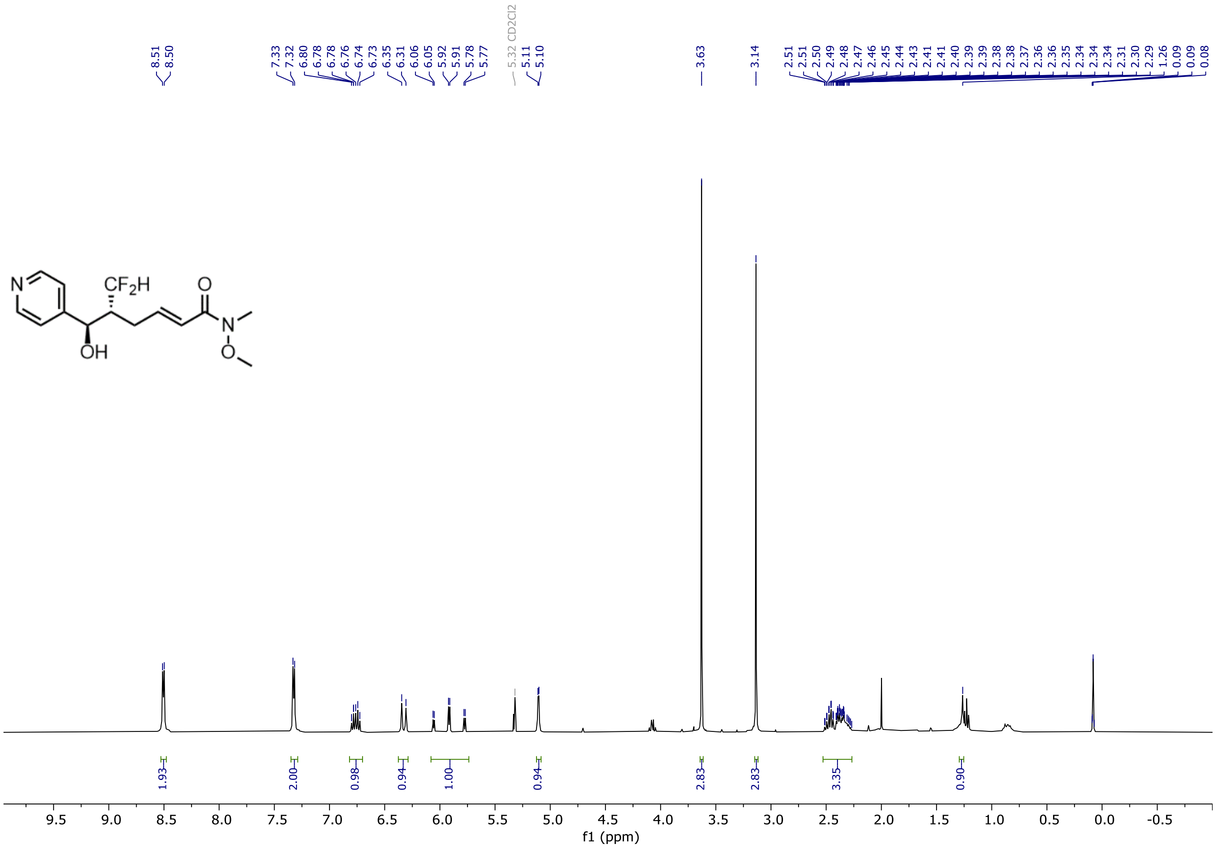

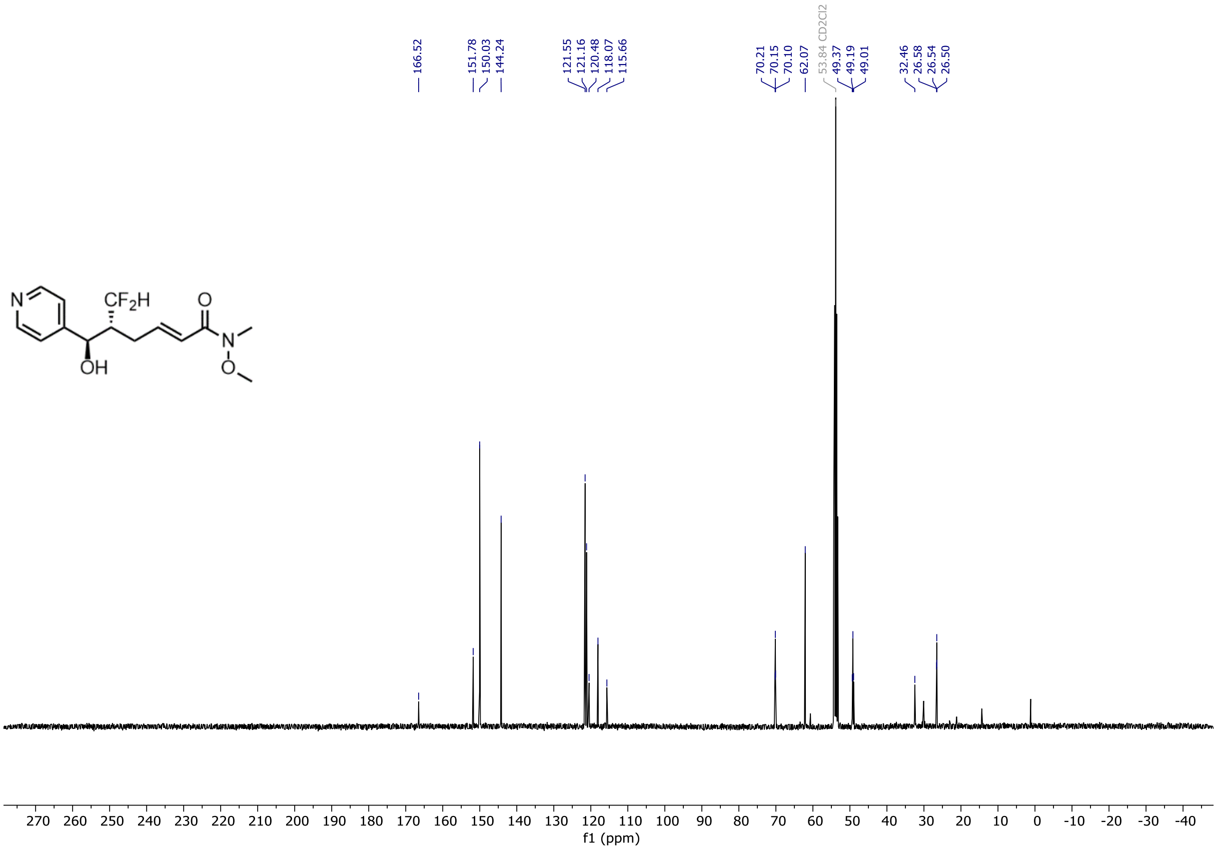

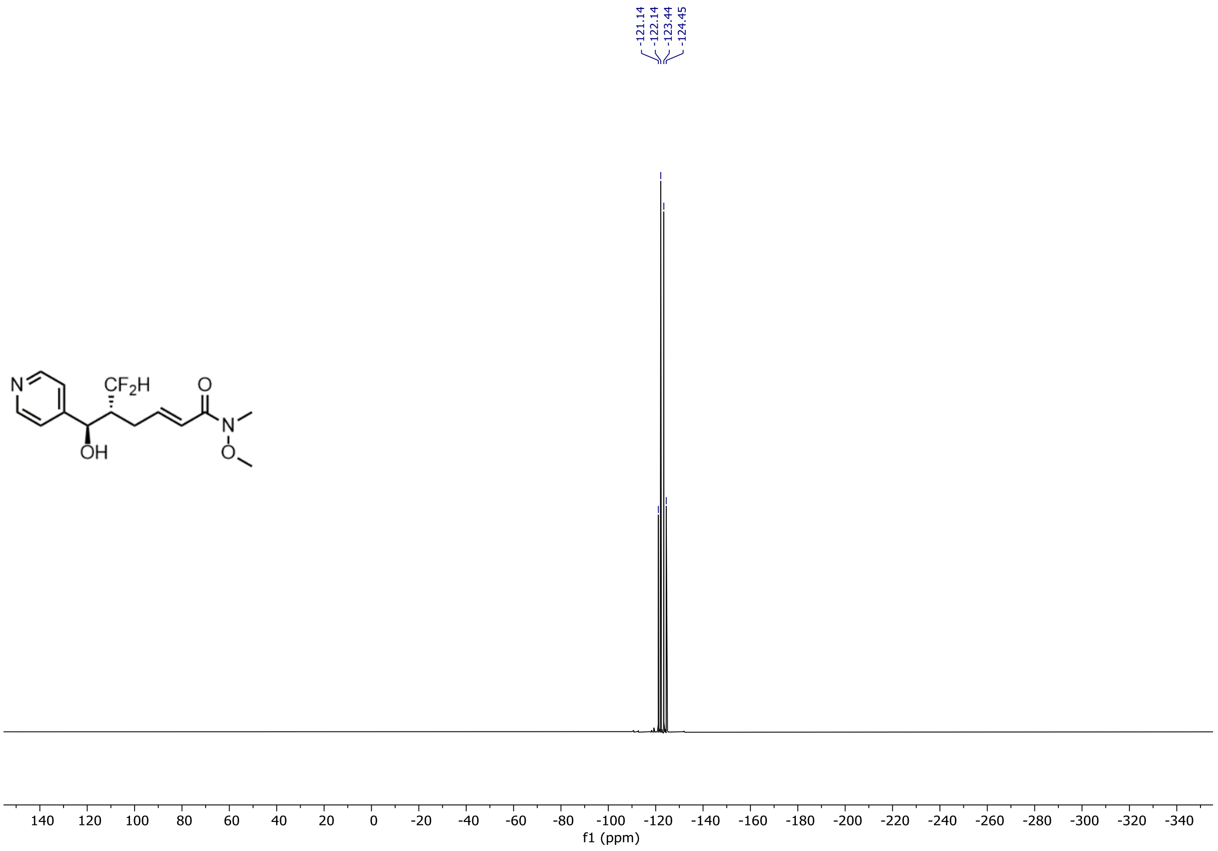
-121.14
-122.14
-123.44
-124.45
CF2H
O
N
OH
140 120 100 80 60 40 20 0 -20 -40 -60 -80 -100 -120 -140 -160 -180 -200 -220 -240 -260 -280 -300 -320 -340
f1 (ppm)

^{1}H NMR (400 MHz, CD_2Cl_2; top), ^{13}C NMR (101 MHz, CD_2Cl_2; middle) and ^{19}F NMR (282 MHz, CD_2Cl_2) of compound **20k**

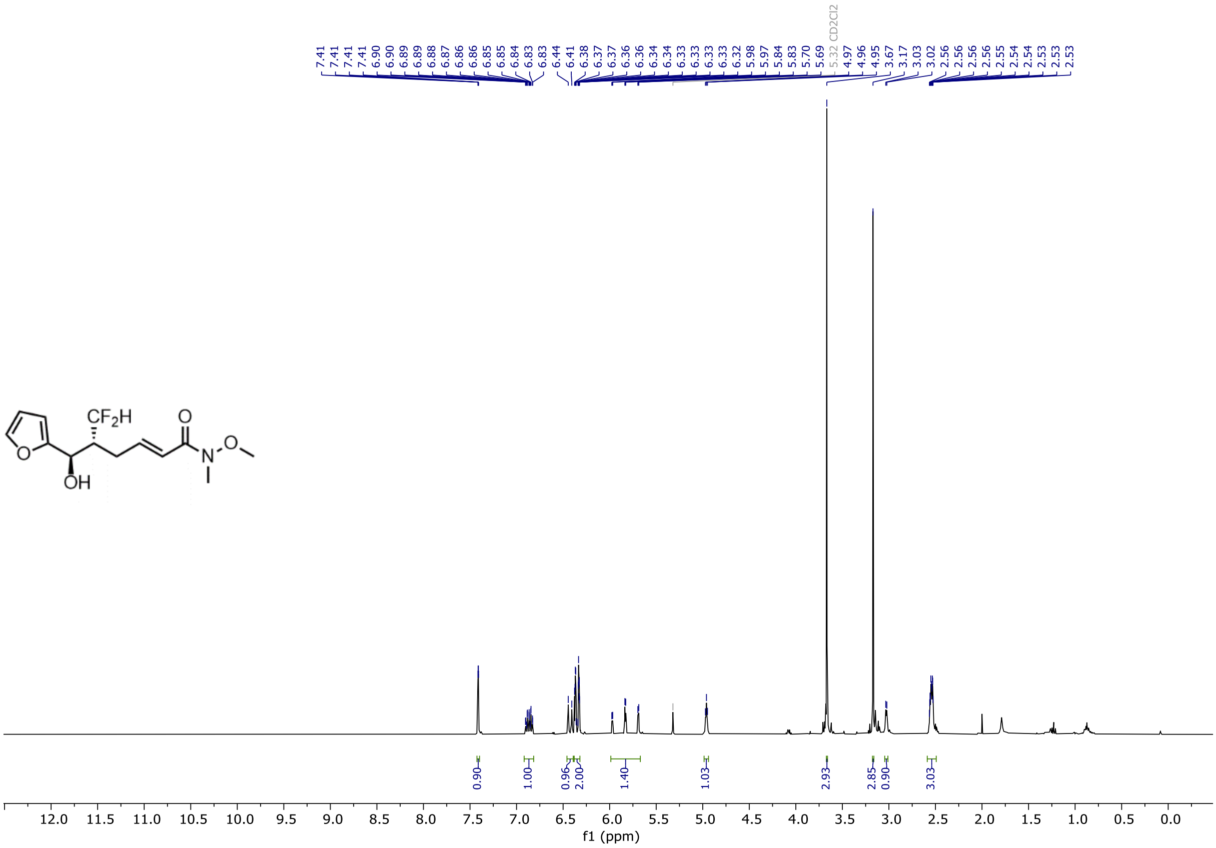

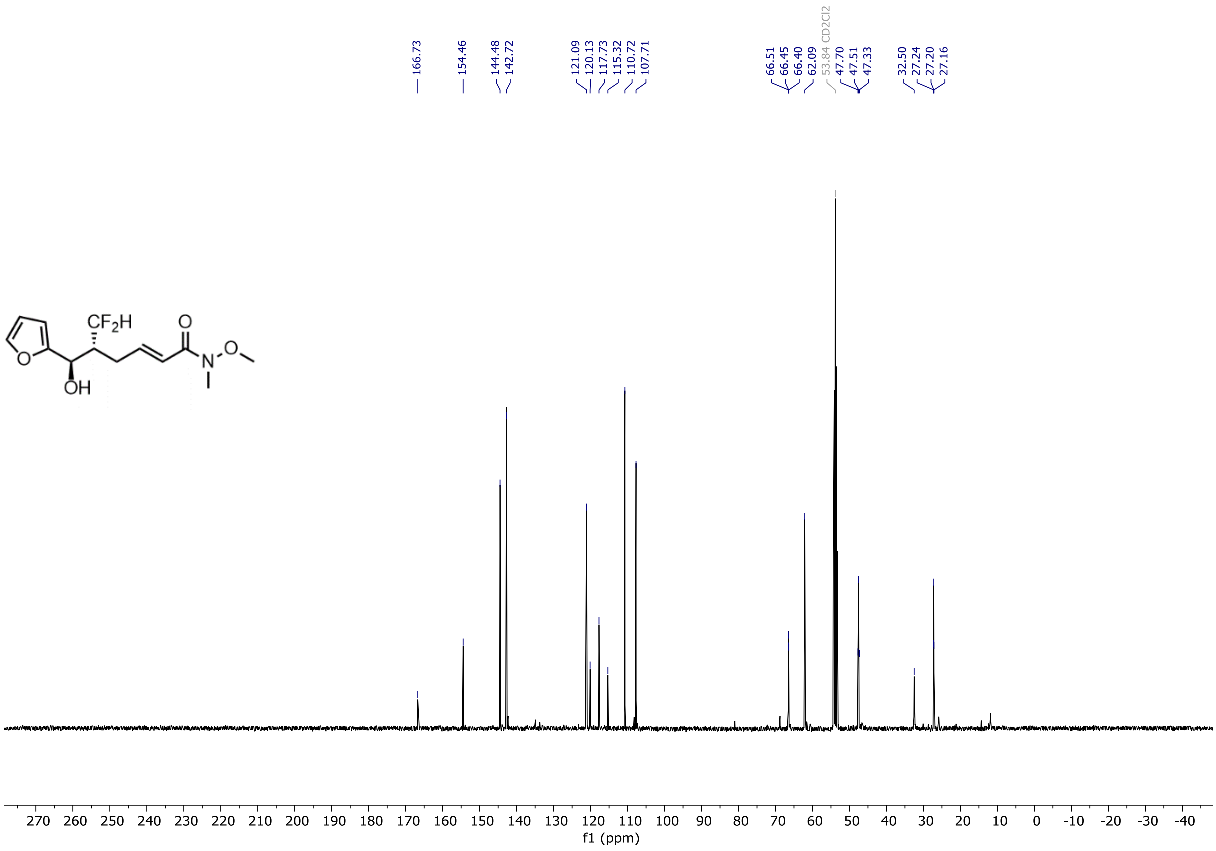

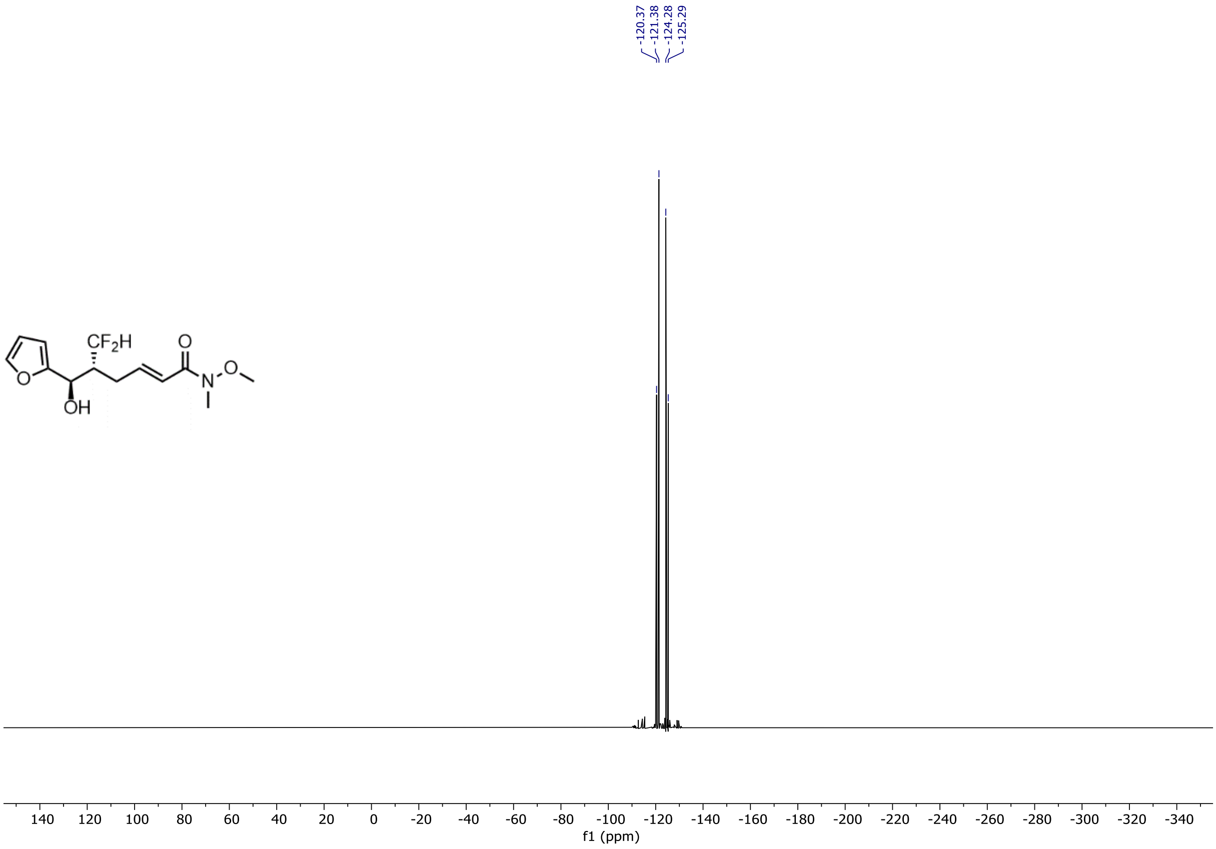

CF2H
O
N
O
O
OH
-120.37
-121.38
-124.28
-125.29
140 120 100 80 60 40 20 0 -20 -40 -60 -80 -100 -120 -140 -160 -180 -200 -220 -240 -260 -280 -300 -320 -340
f1 (ppm)

^{1}H NMR (400 MHz, CD_2Cl_2; top), ^{13}C NMR (101 MHz, CD_2Cl_2; middle) and ^{19}F NMR (282 MHz, CD_2Cl_2) of compound **20l**

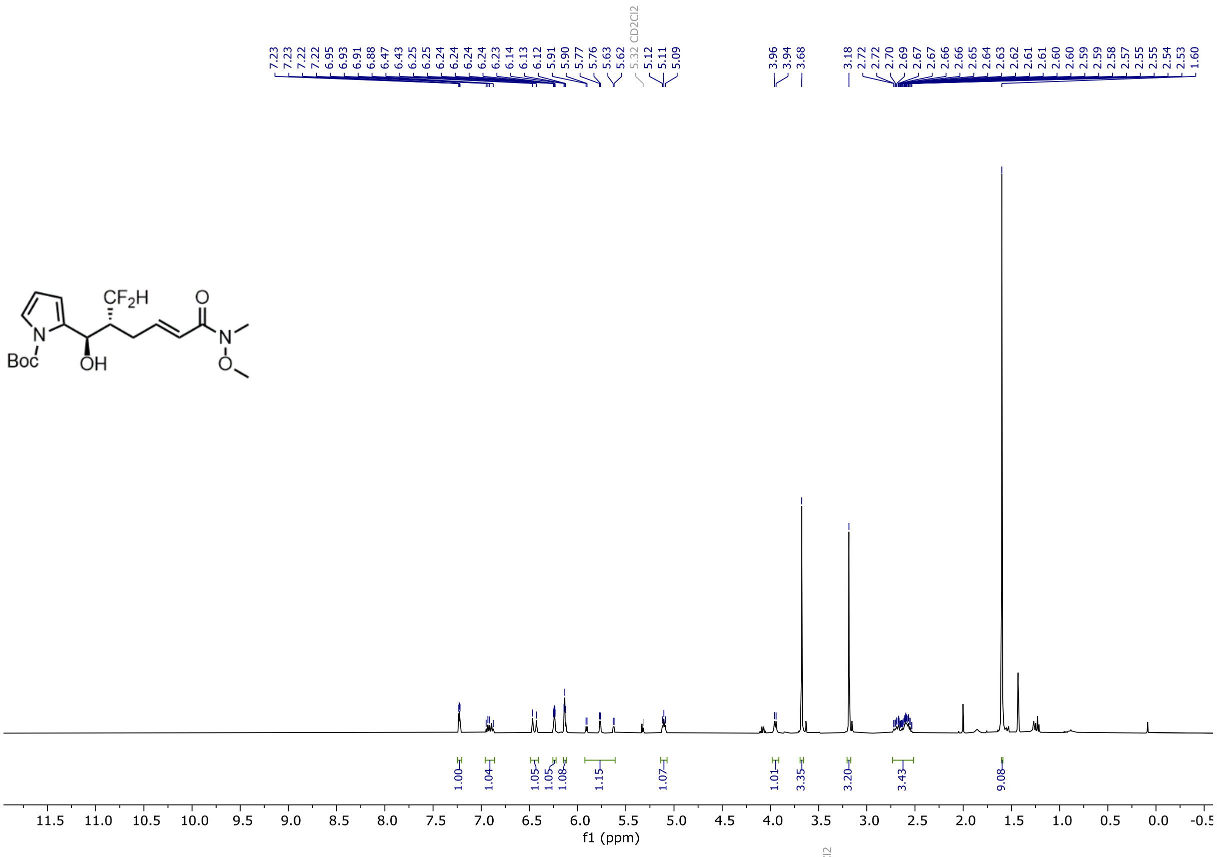

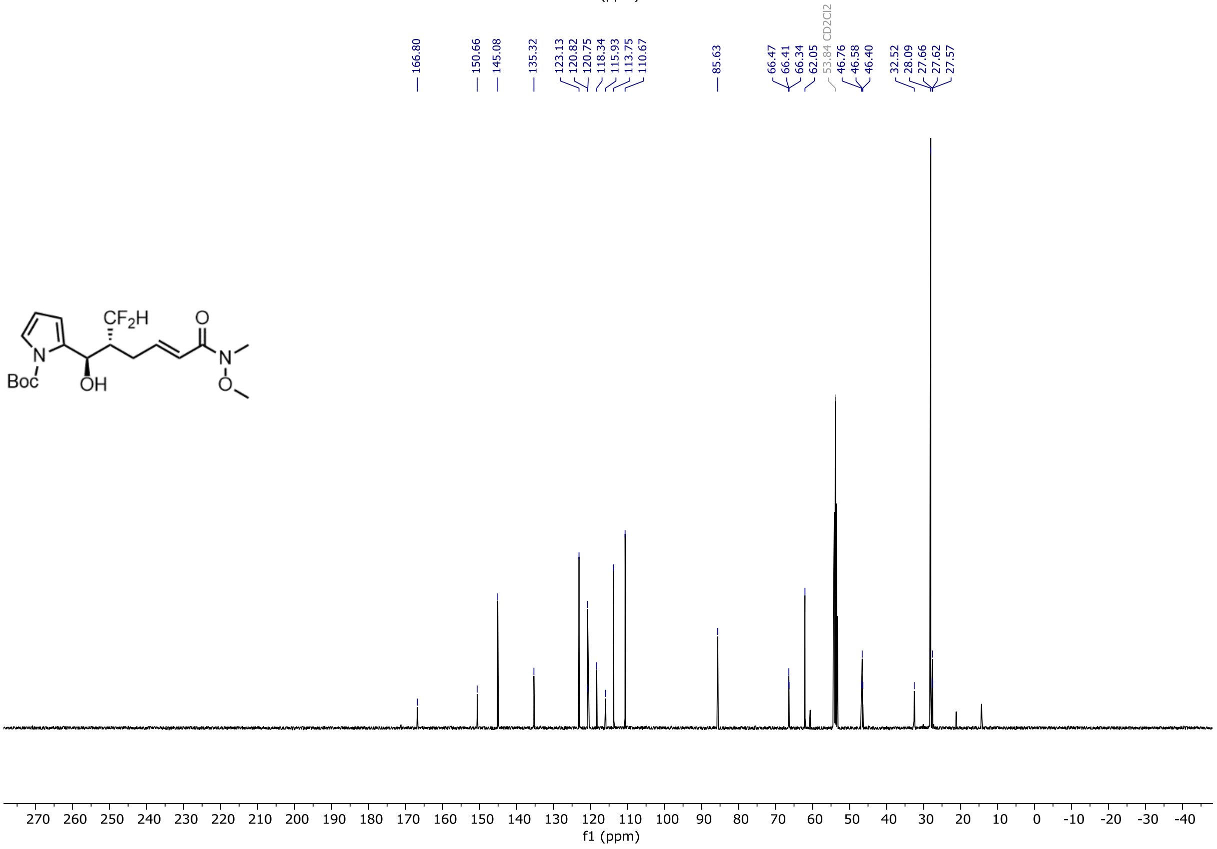

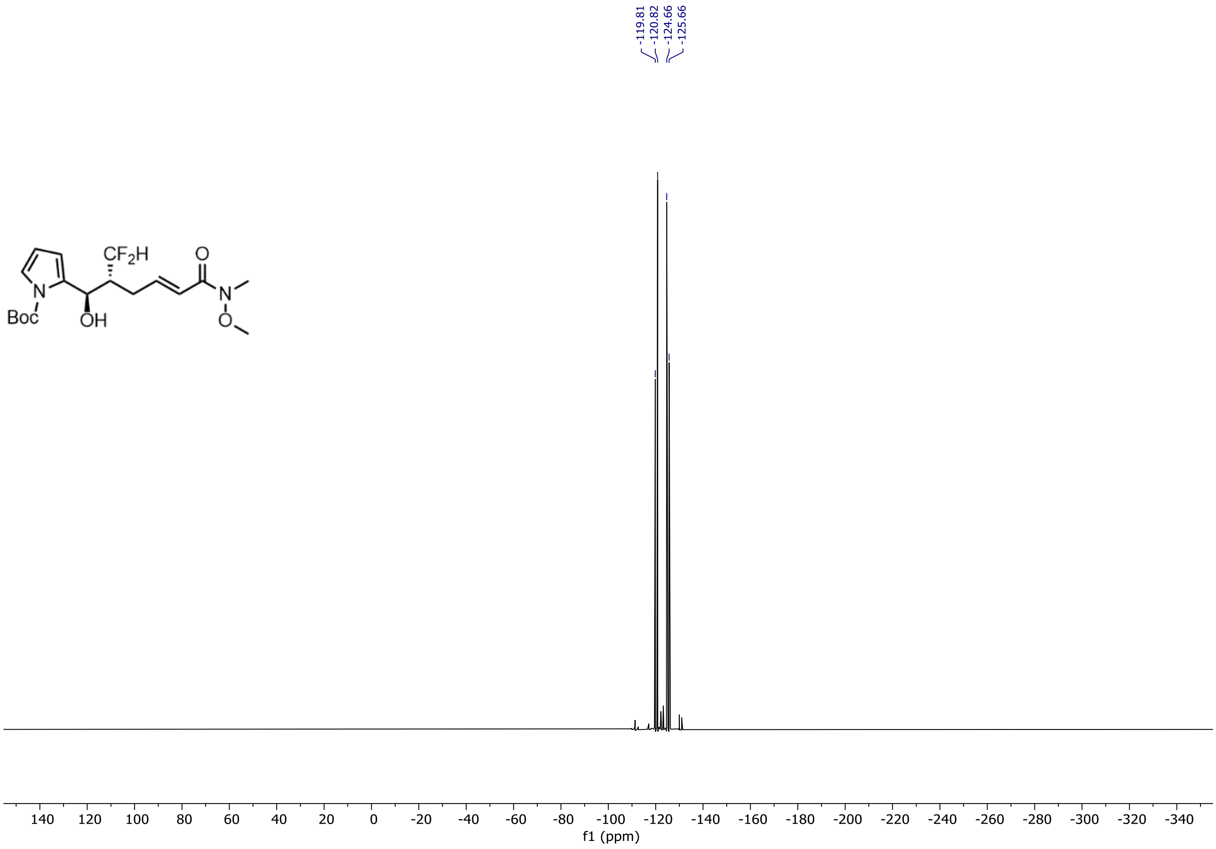

-119.81
-120.82
-124.66
-125.66
CF2H
O
N
Boc
OH
N
O
140 120 100 80 60 40 20 0 -20 -40 -60 -80 -100 -120 -140 -160 -180 -200 -220 -240 -260 -280 -300 -320 -340
f1 (ppm)

^{1}H NMR (400 MHz, CD_2Cl_2; top), ^{13}C NMR (101 MHz, CD_2Cl_2; middle) and ^{19}F NMR (282 MHz, CD_2Cl_2) of compound **20m**

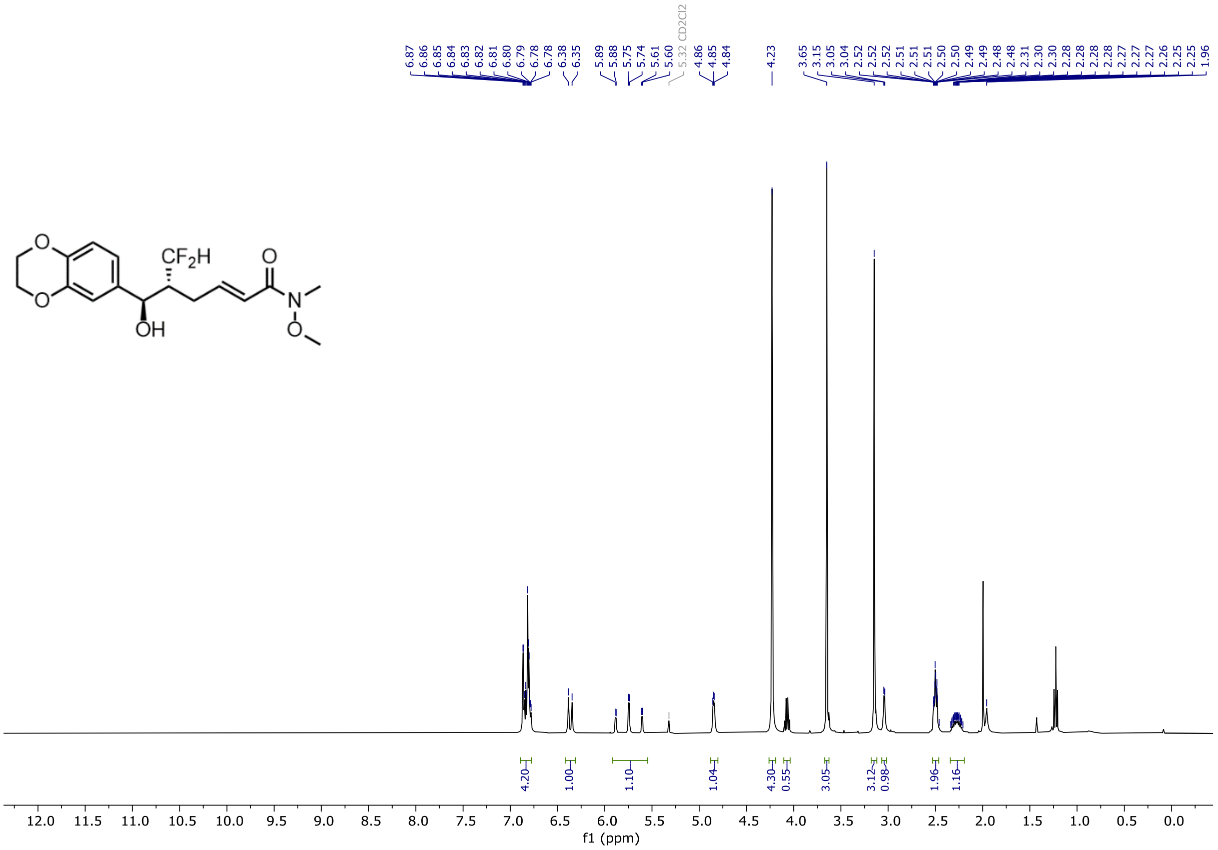

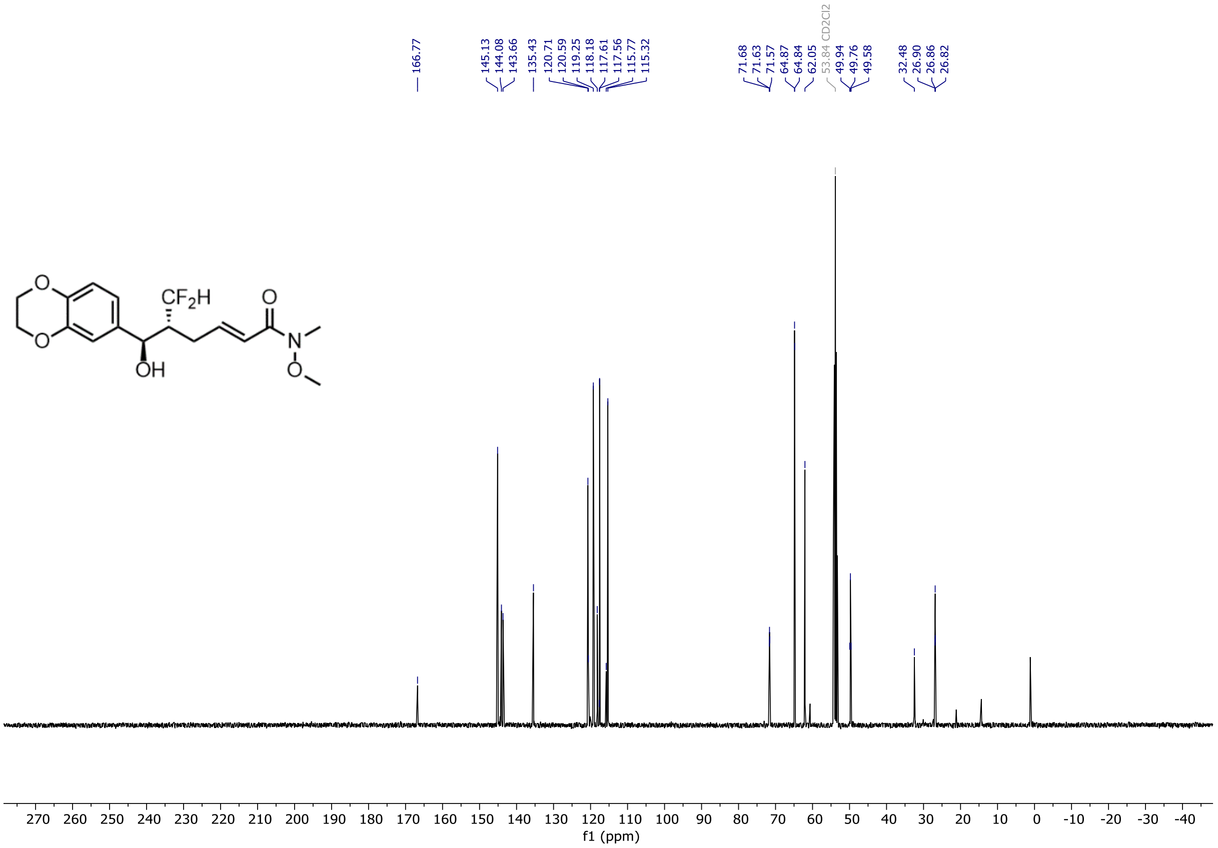

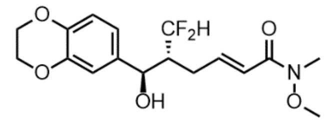

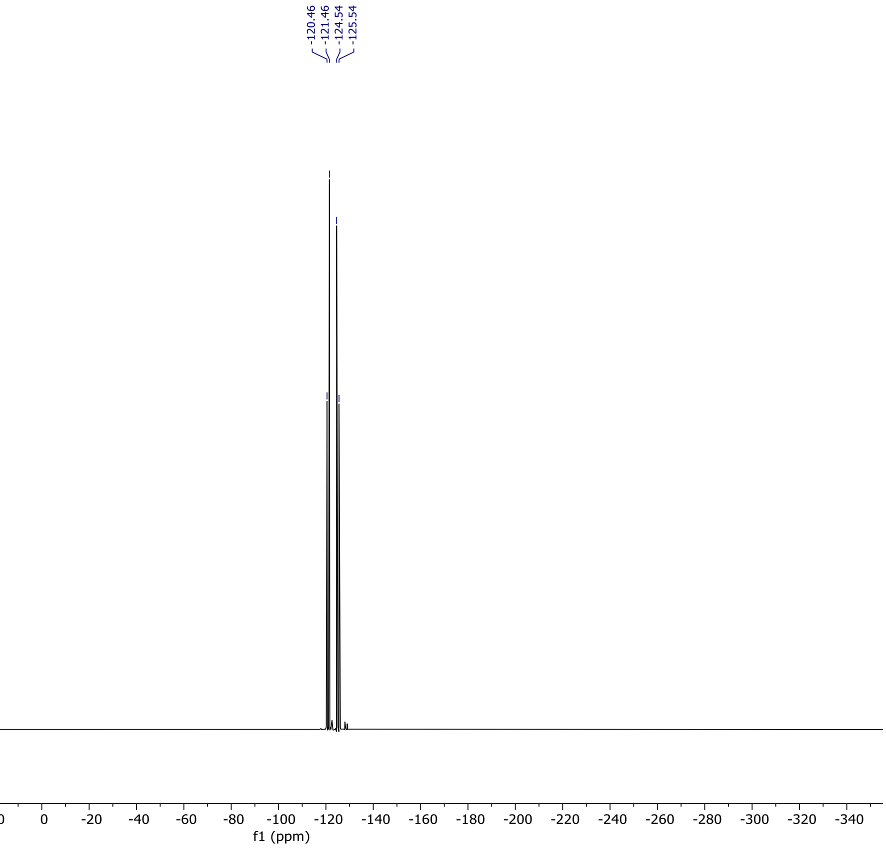
-120.46
-121.46
-124.54
-125.54
140
120
100
80
60
40
20
0
-20
-40
-60
-80
-100
-120
-140
-160
-180
-200
-220
-240
-260
-280
-300
-320
-340
f1 (ppm)

^{1}H NMR (400 MHz, CD_2Cl_2; top), ^{13}C NMR (101 MHz, CD_2Cl_2; middle) and ^{19}F NMR (282 MHz, CD_2Cl_2) of compound **20n** [Note: due to high viscosity of the isolated compound, residual cyclohexane (≈ 5 %) is visible in the ^{1}H NMR spectrum]

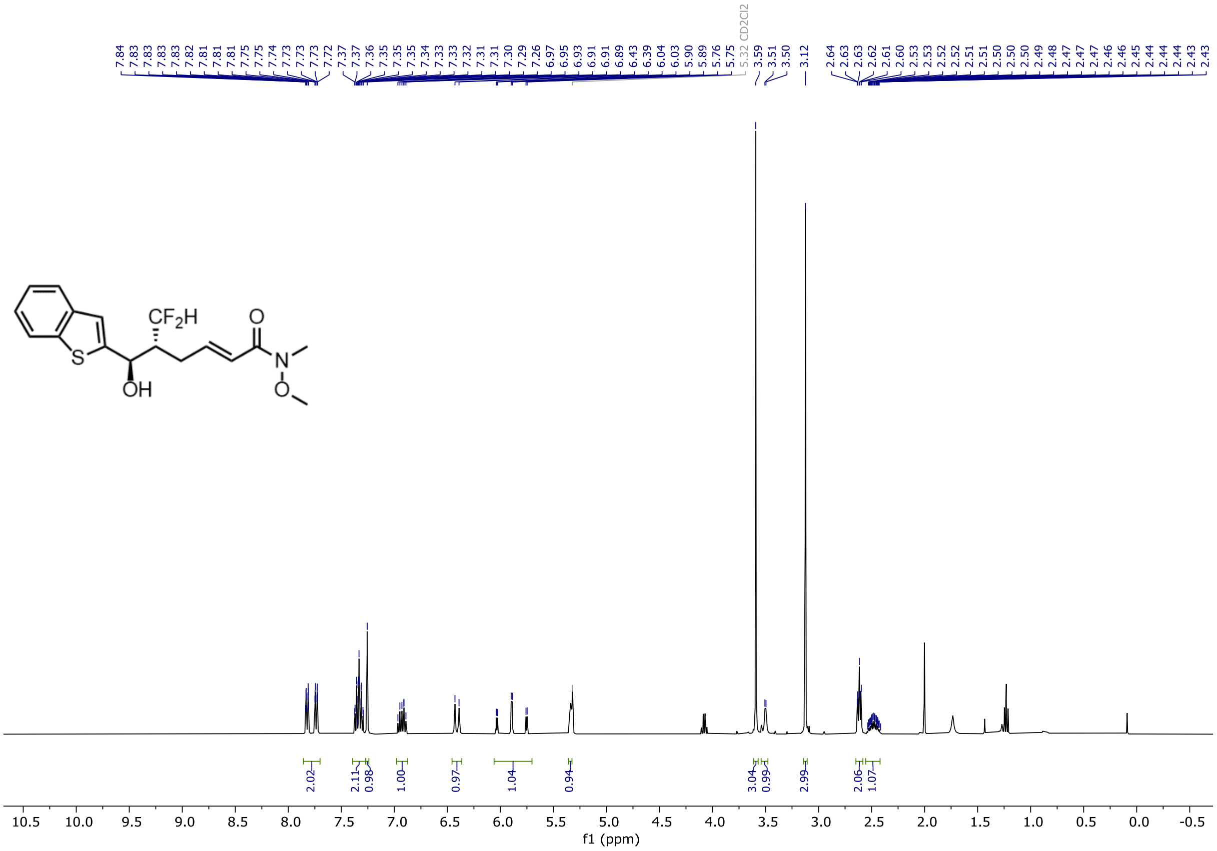

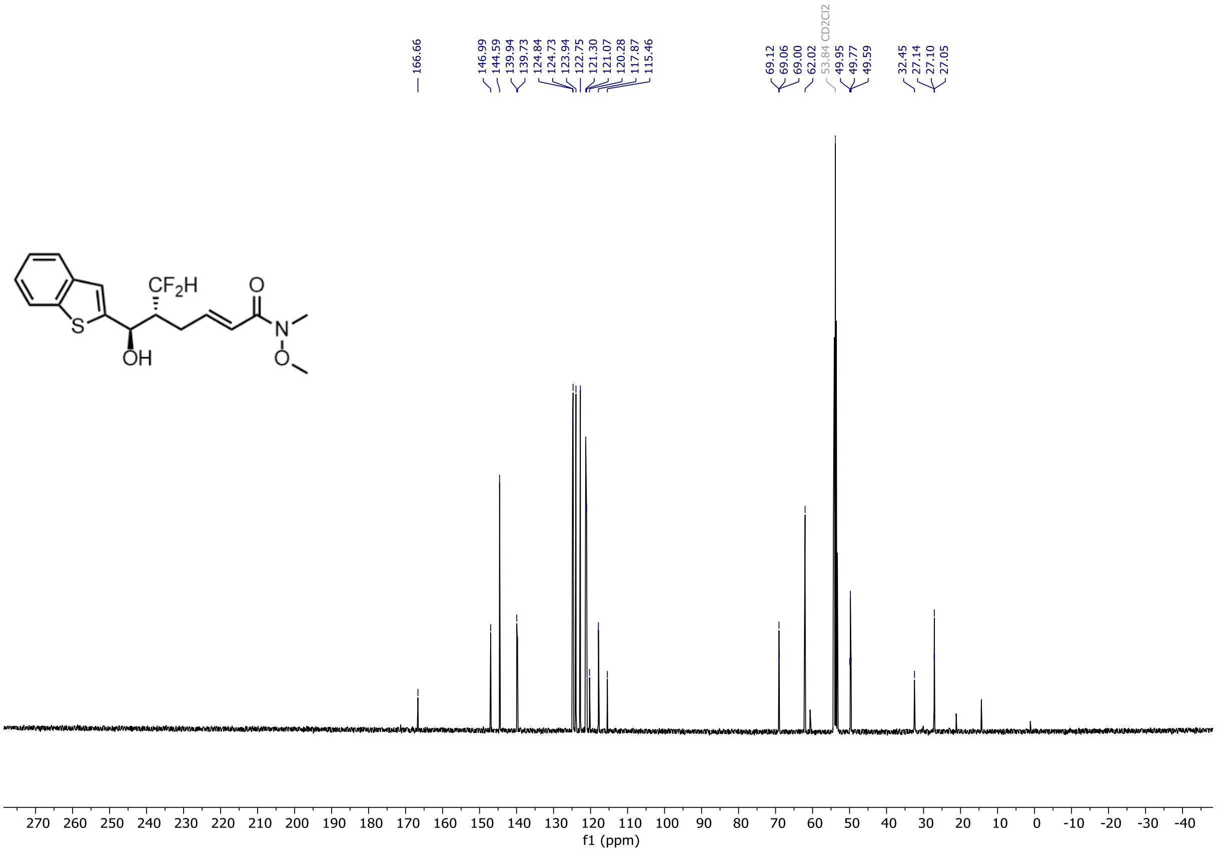

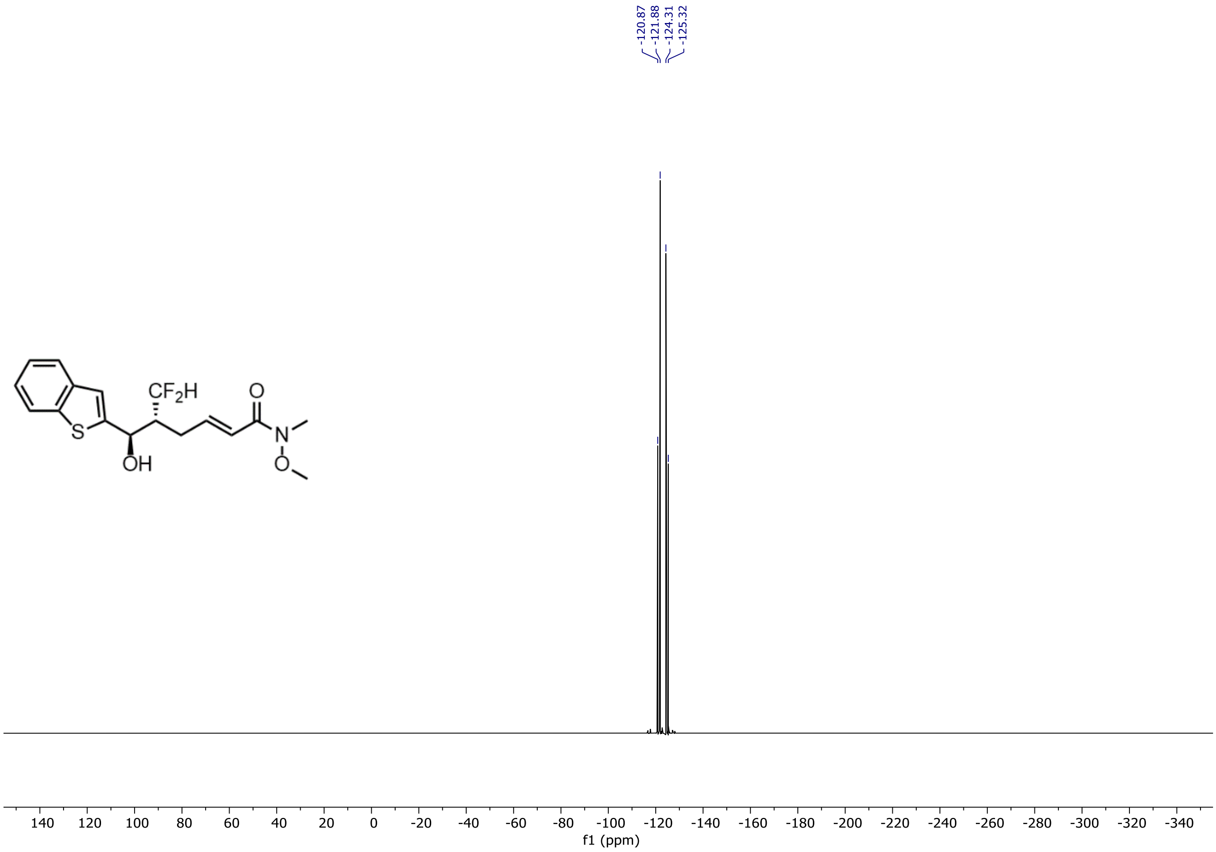

-120.87
-121.88
-124.31
-125.32
140
120
100
80
60
40
20
0
-20
-40
-60
-80
-100
-120
-140
-160
-180
-200
-220
-240
-260
-280
-300
-320
-340
f1 (ppm)

^{1}H NMR (400 MHz, CD_2Cl_2; top), ^{13}C NMR (101 MHz, CD_2Cl_2; middle) and ^{19}F NMR (282 MHz, CD_2Cl_2) of compound **21a**

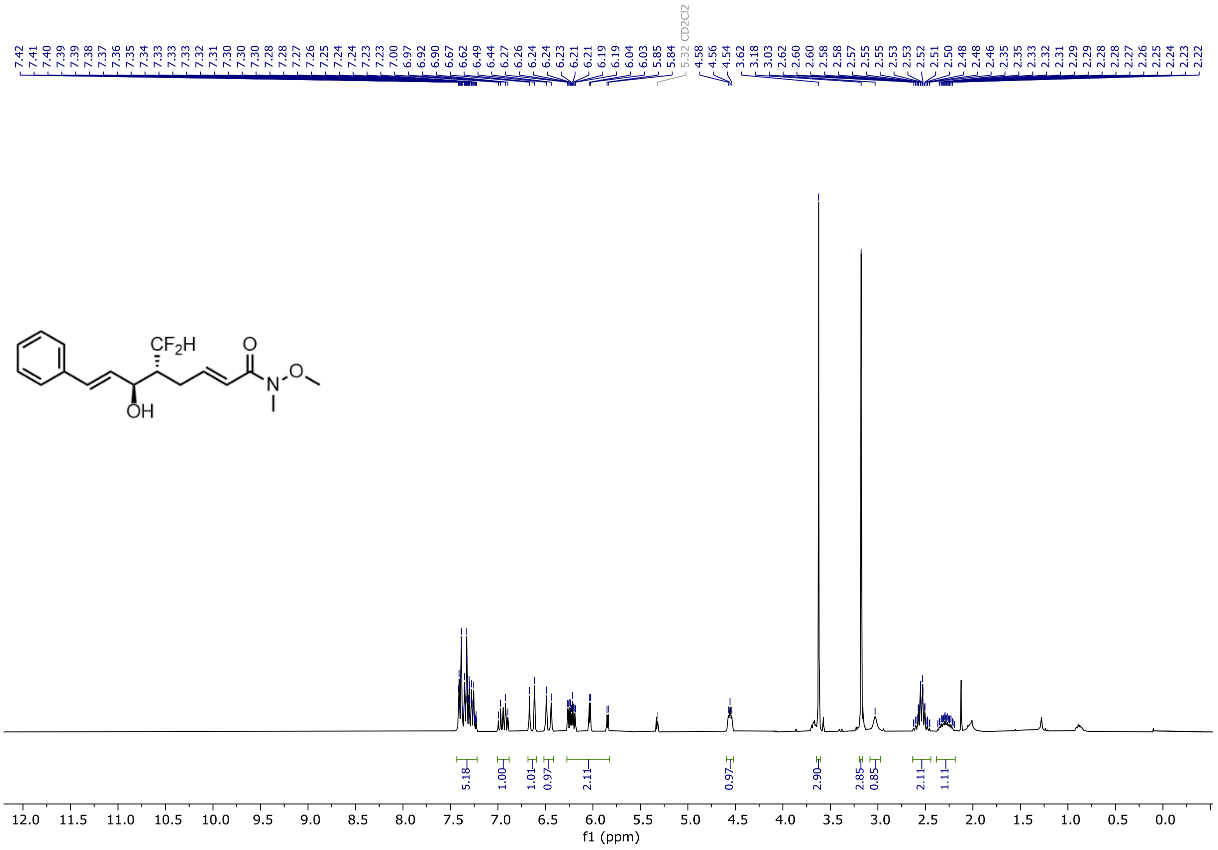

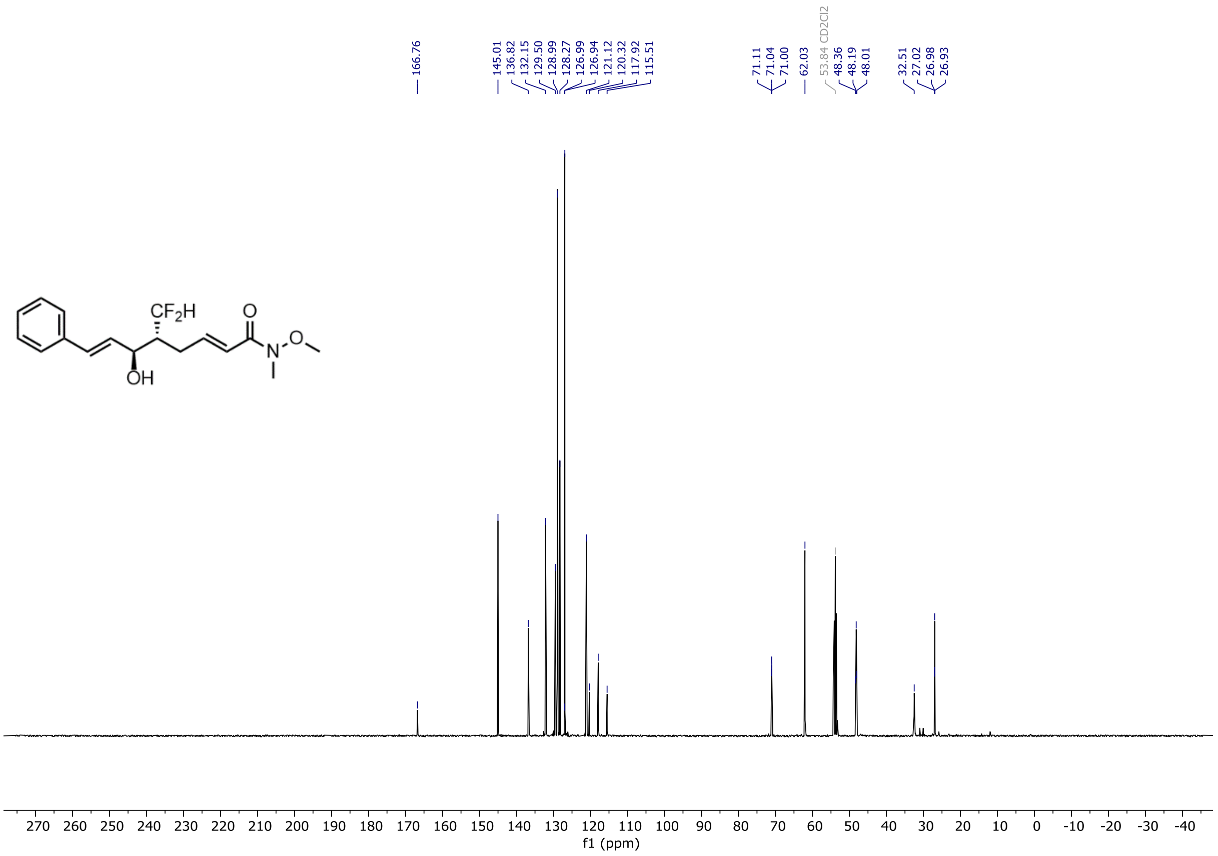

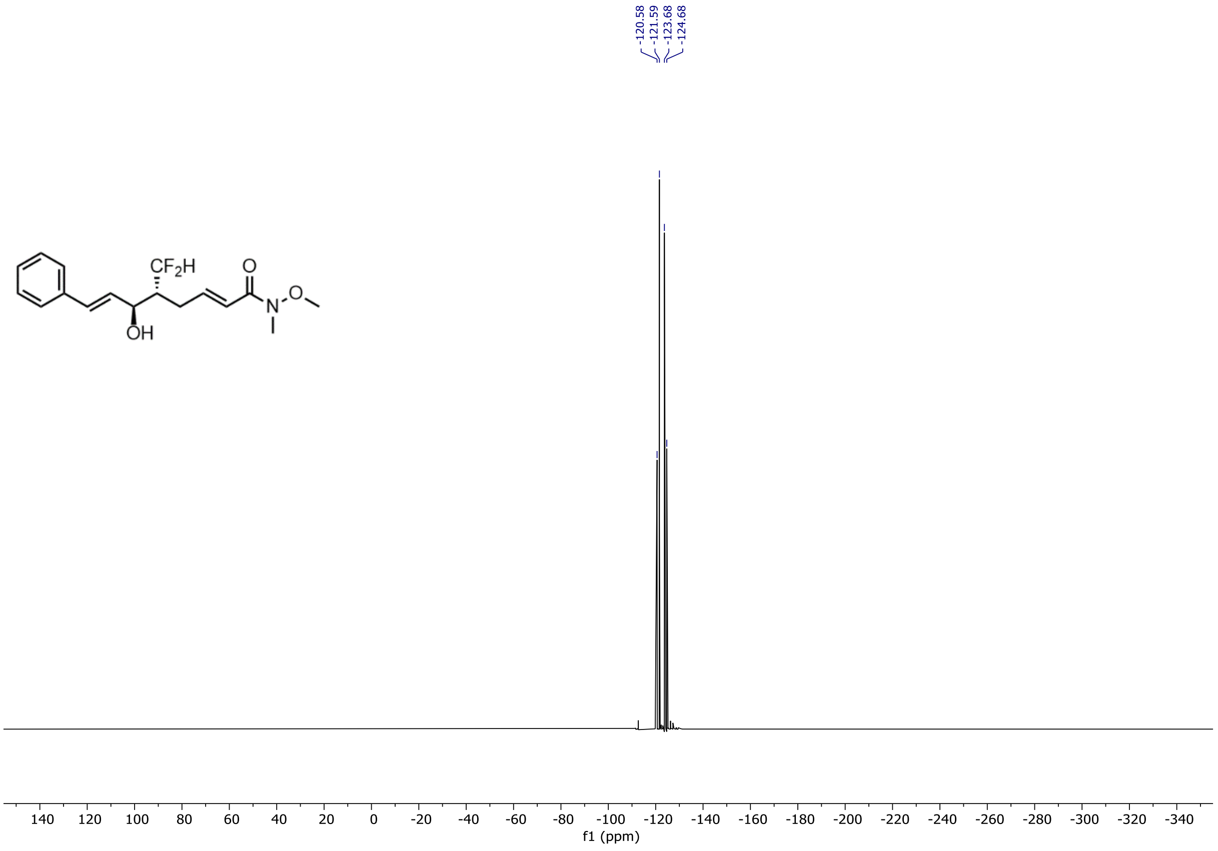
-120.58
-121.59
-123.68
-124.68
CF2H
O
N
O
OH
140 120 100 80 60 40 20 0 -20 -40 -60 -80 -100 -120 -140 -160 -180 -200 -220 -240 -260 -280 -300 -320 -340
f1 (ppm)

^{1}H NMR (400 MHz, CD_2Cl_2; top), ^{13}C NMR (101 MHz, CD_2Cl_2; middle) and ^{19}F NMR (282 MHz, CD_2Cl_2) of compound **21b**

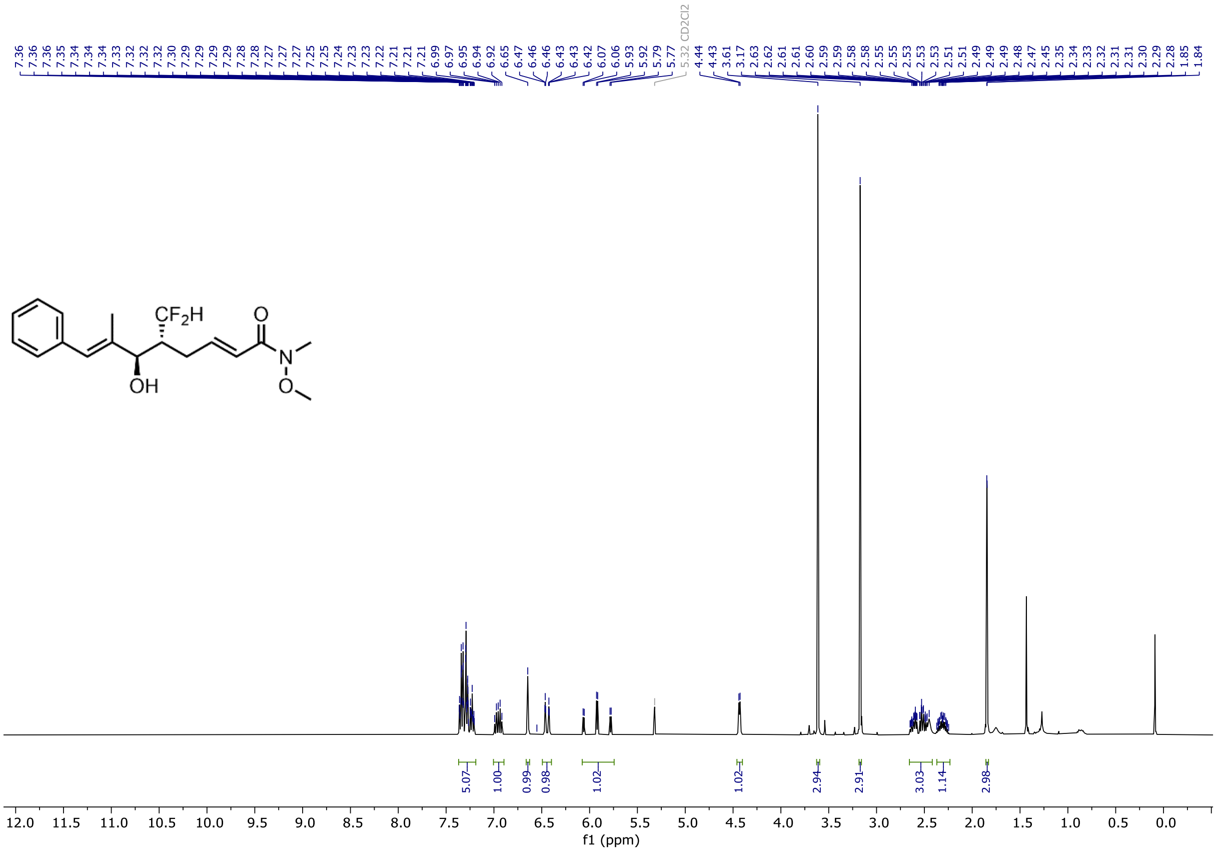

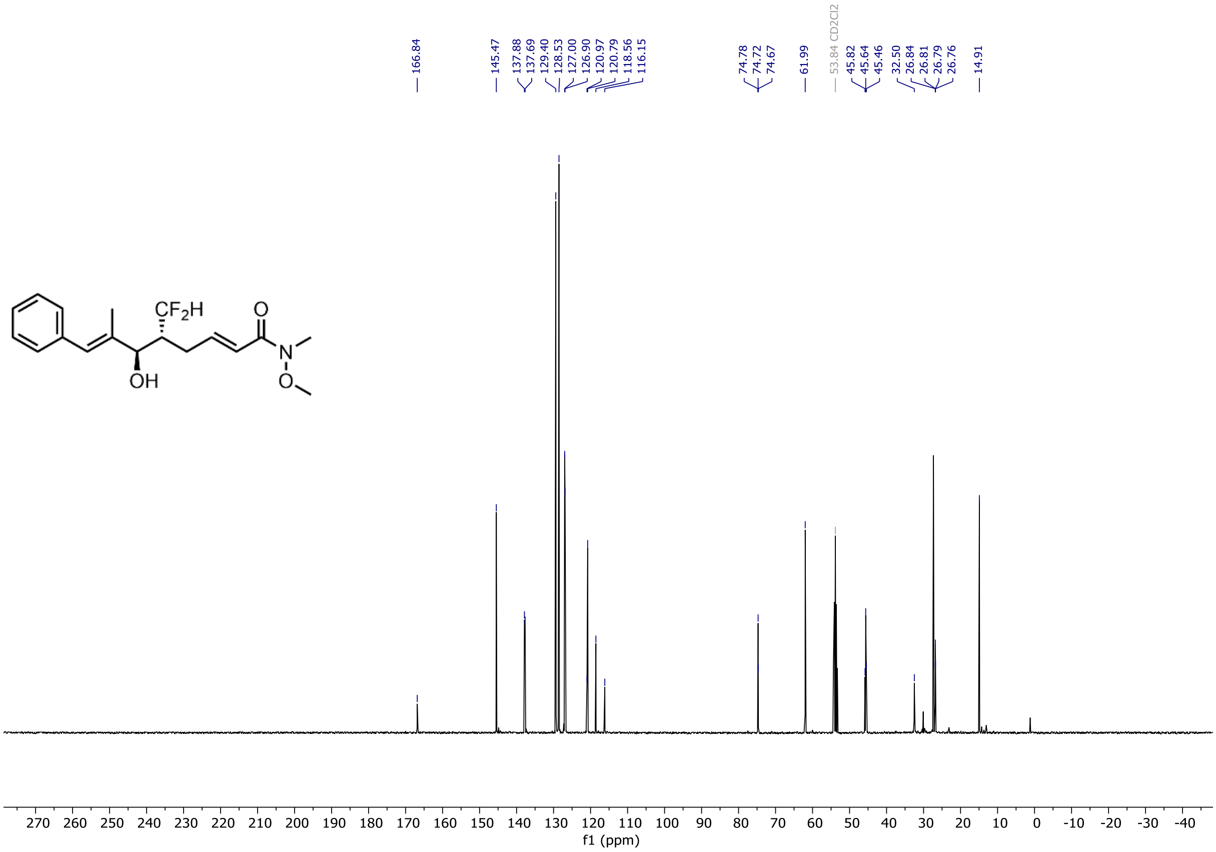

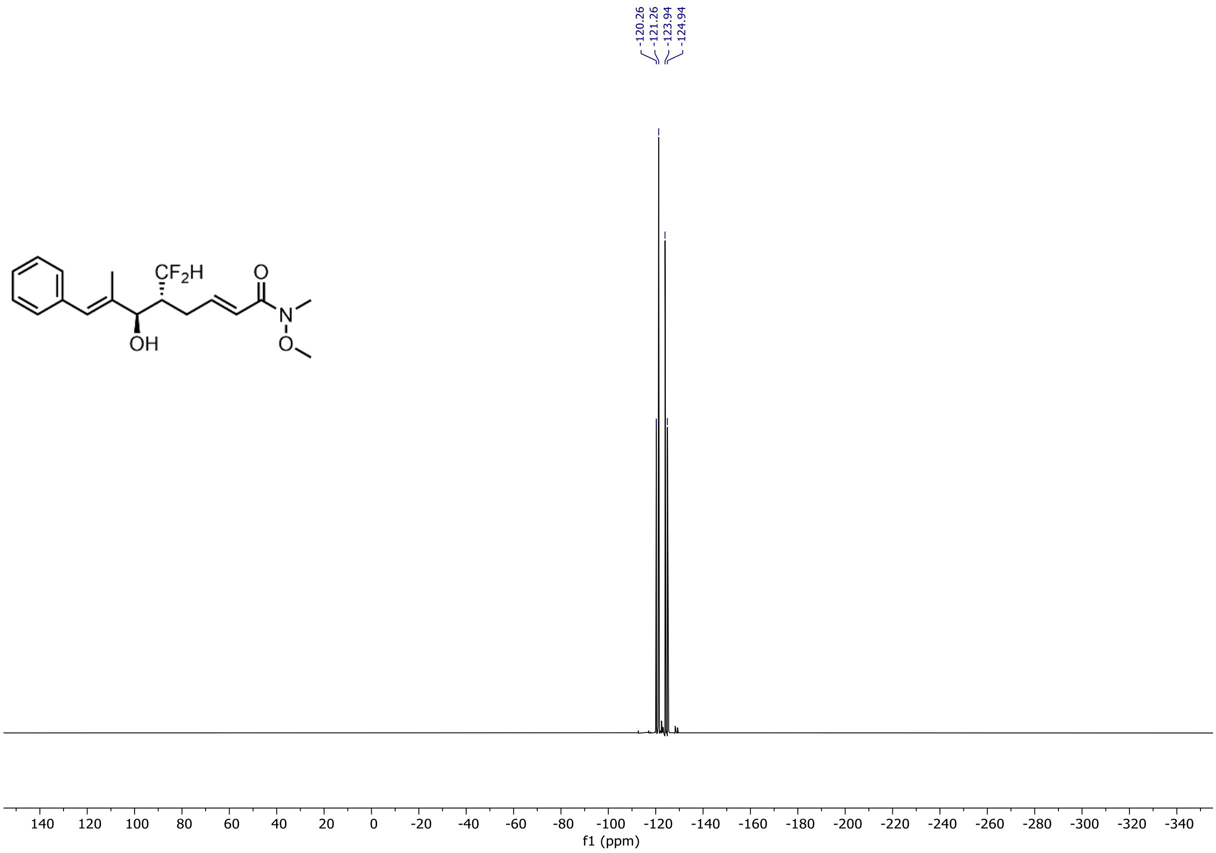
-120.26
-121.26
-123.94
-124.94
CF_2H
O
N
O
OH
140 120 100 80 60 40 20 0 -20 -40 -60 -80 -100 -120 -140 -160 -180 -200 -220 -240 -260 -280 -300 -320 -340
f1 (ppm)

^{1}H NMR (400 MHz, CD_2Cl_2; top), ^{13}C NMR (101 MHz, CD_2Cl_2; middle) and ^{19}F NMR (282 MHz, CD_2Cl_2) of compound **21c**

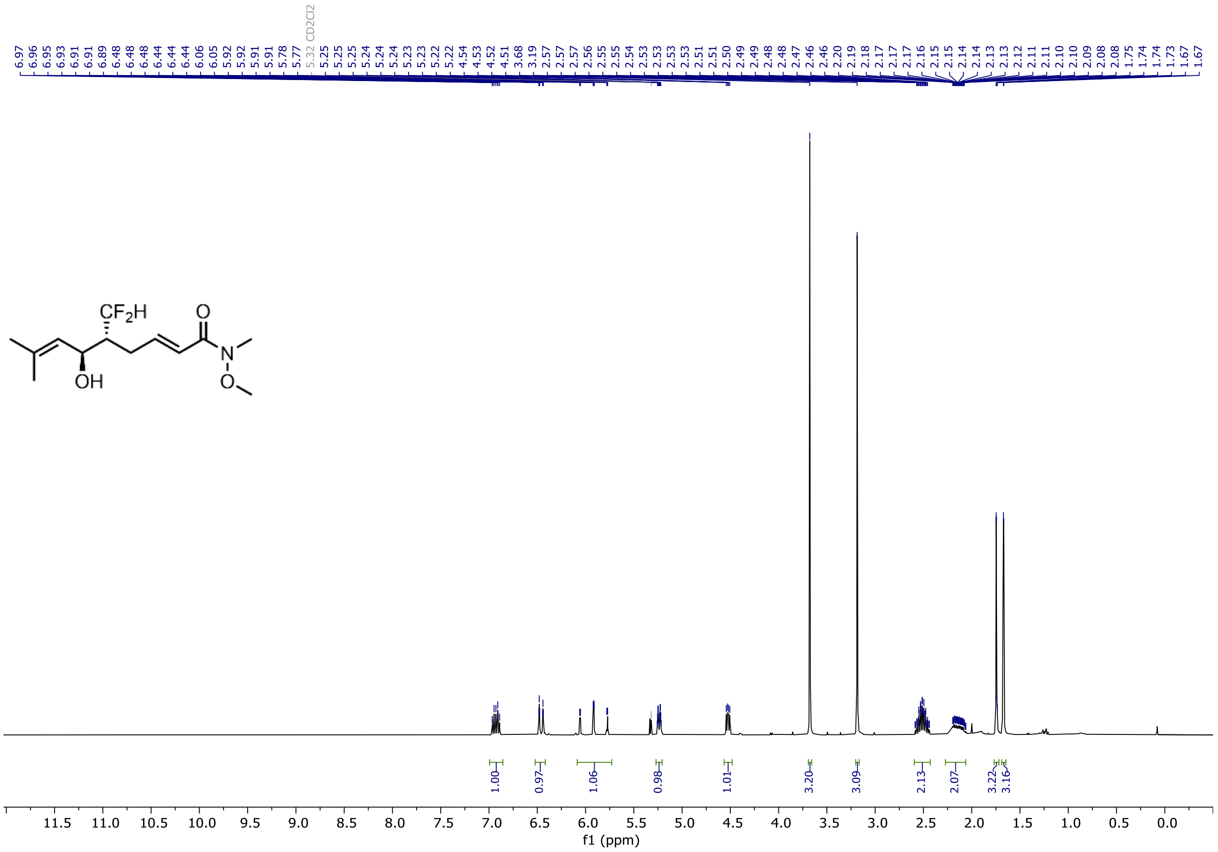

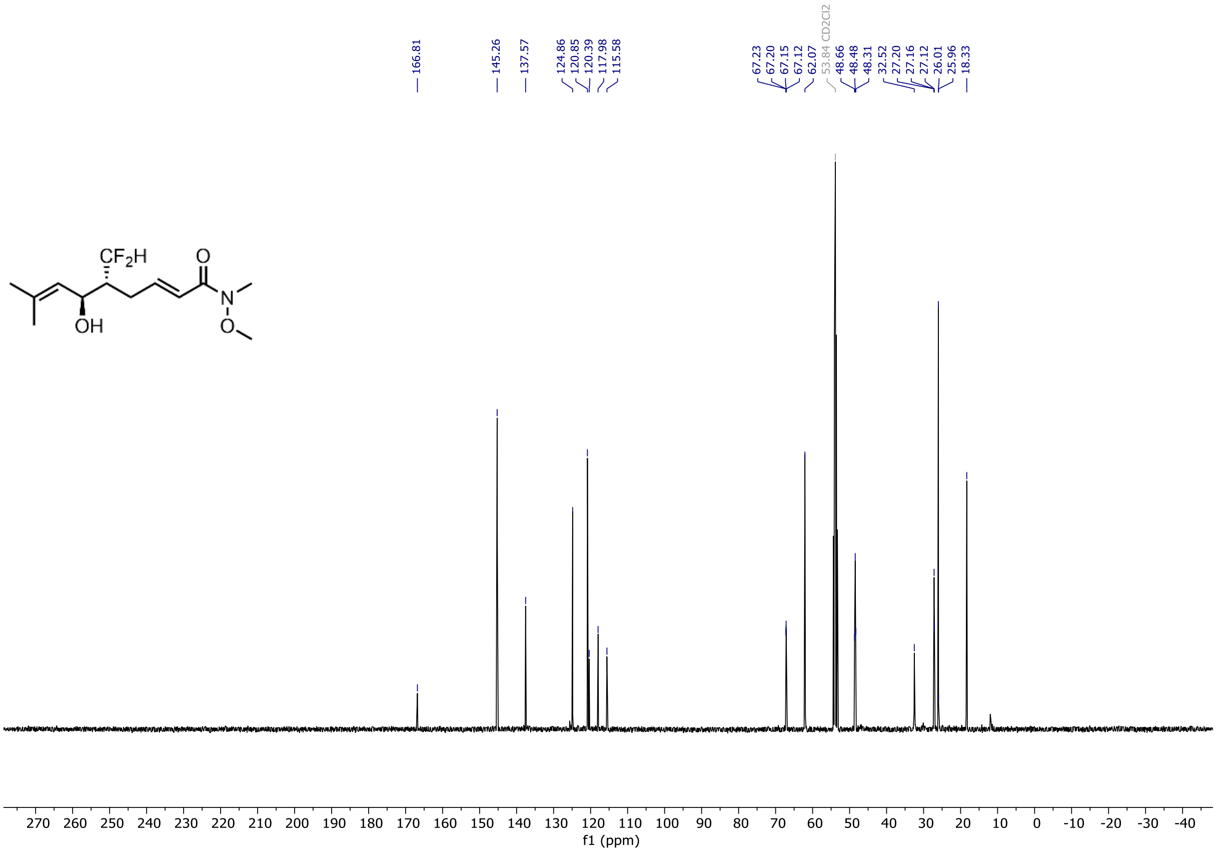

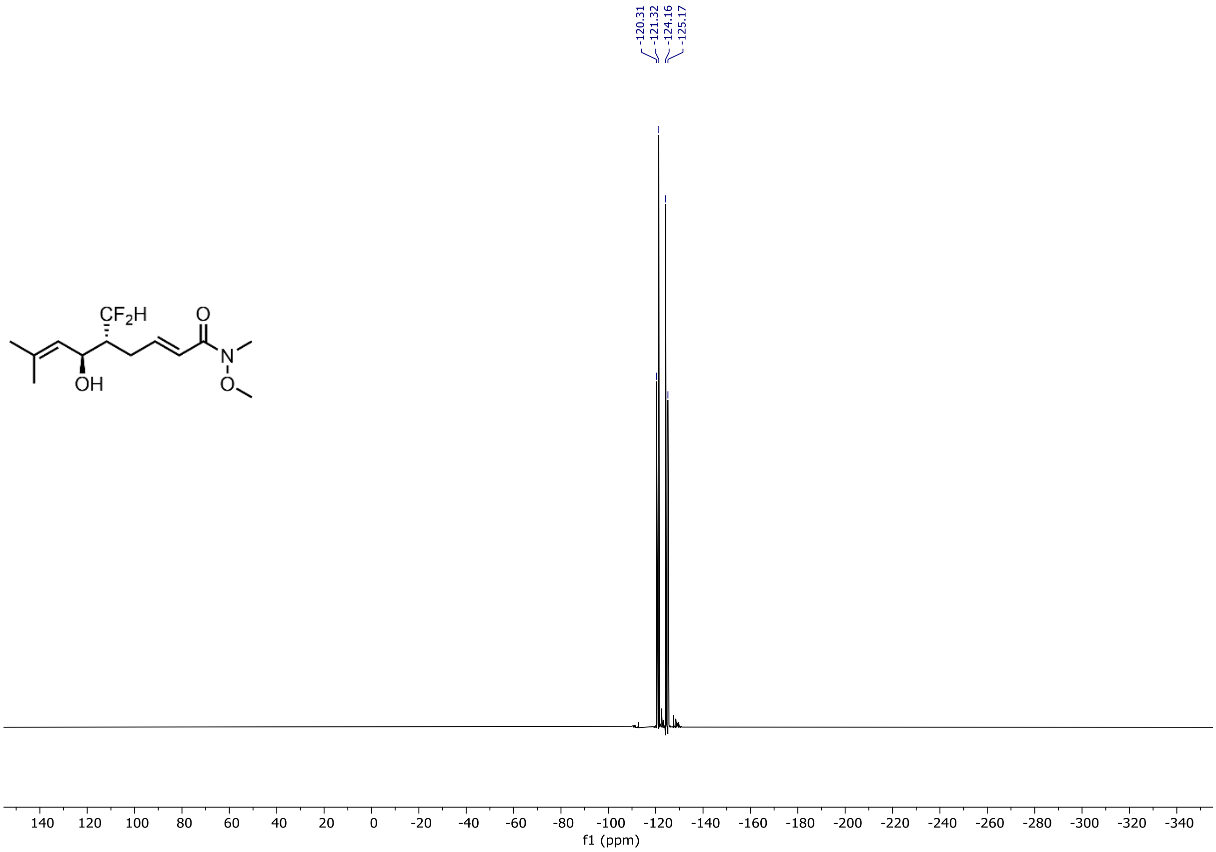
CF2H
O
N
O
OH
-120.31
-121.32
-124.16
-125.17
140 120 100 80 60 40 20 0 -20 -40 -60 -80 -100 -120 -140 -160 -180 -200 -220 -240 -260 -280 -300 -320 -340
f1 (ppm)

^{1}H NMR (400 MHz, CD_2Cl_2; top), ^{13}C NMR (101 MHz, CD_2Cl_2; middle) and ^{19}F NMR (282 MHz, CD_2Cl_2) of compound **21d**

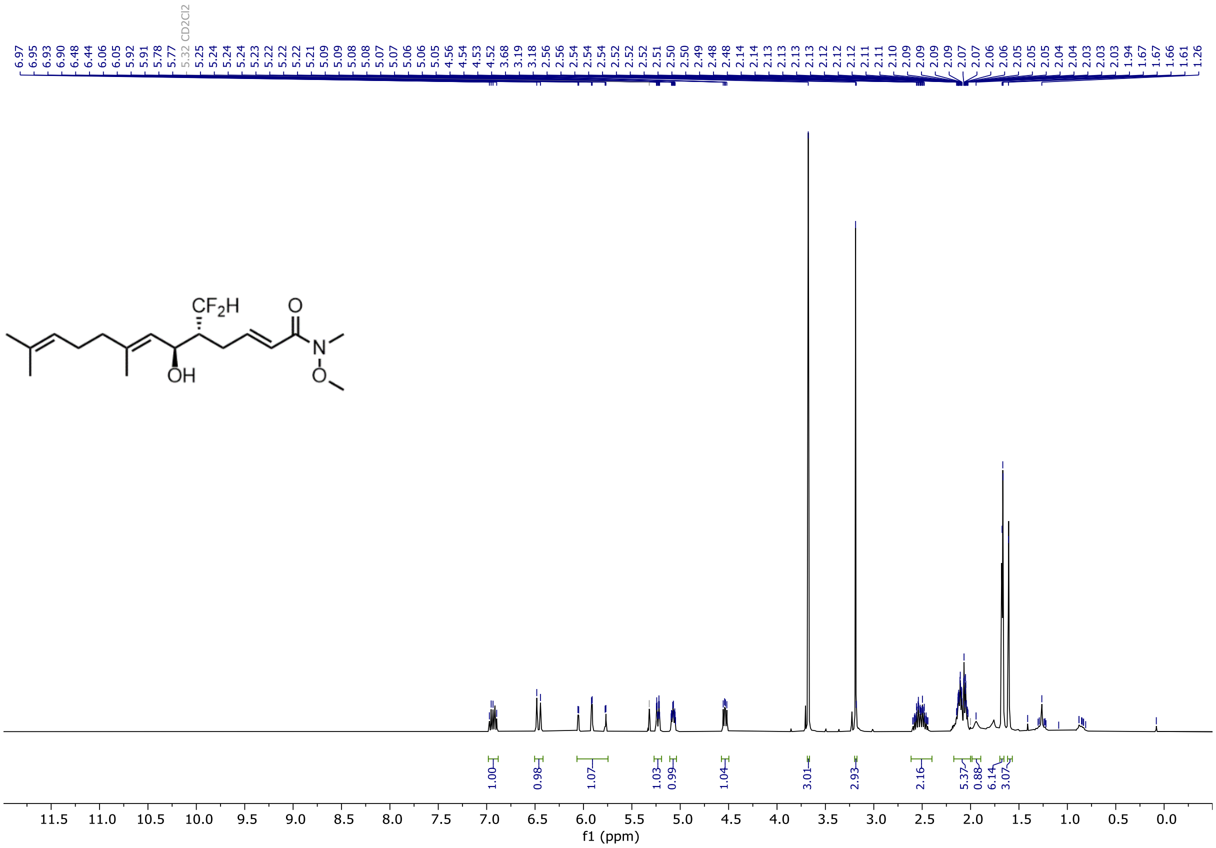

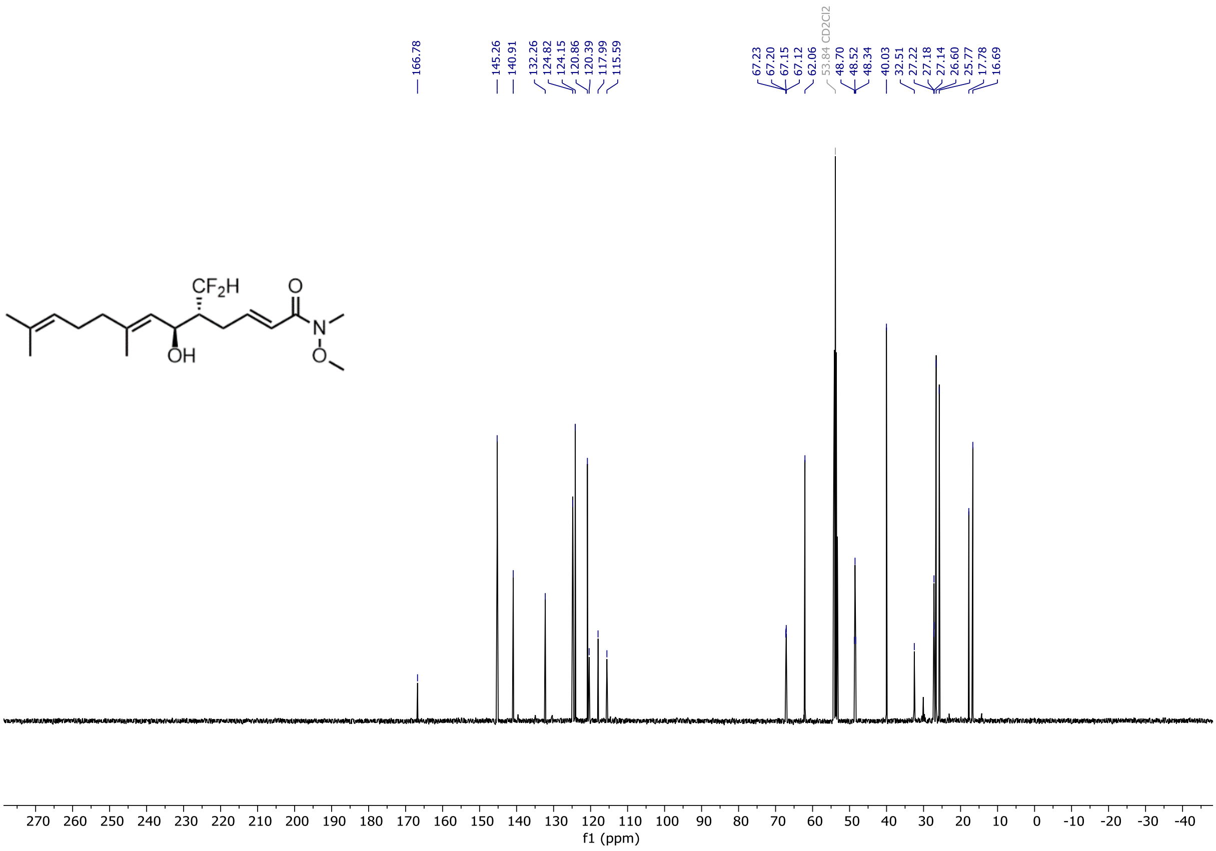

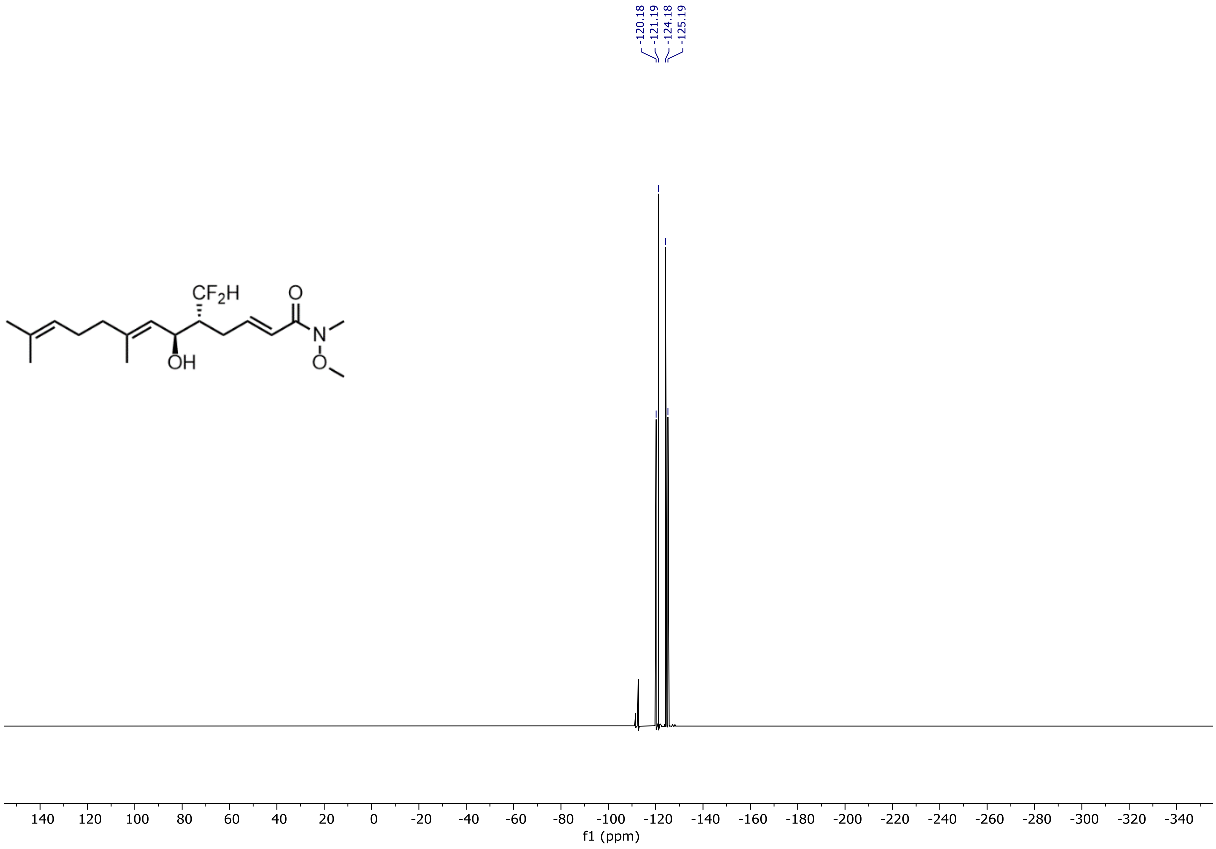
CF2H
O
N
O
OH
-120.18
-121.19
-124.18
-125.19
140 120 100 80 60 40 20 0 -20 -40 -60 -80 -100 -120 -140 -160 -180 -200 -220 -240 -260 -280 -300 -320 -340
f1 (ppm)

^{1}H NMR (400 MHz, CD_2Cl_2; top), ^{13}C NMR (101 MHz, CD_2Cl_2; middle) and ^{19}F NMR (282 MHz, CD_2Cl_2) of compound **21e (dr ≈ 4:1)** [Note: due to high viscosity of the isolated compound, residual cyclohexane (≈ 6 %) is visible in the ^{1}H NMR spectrum]

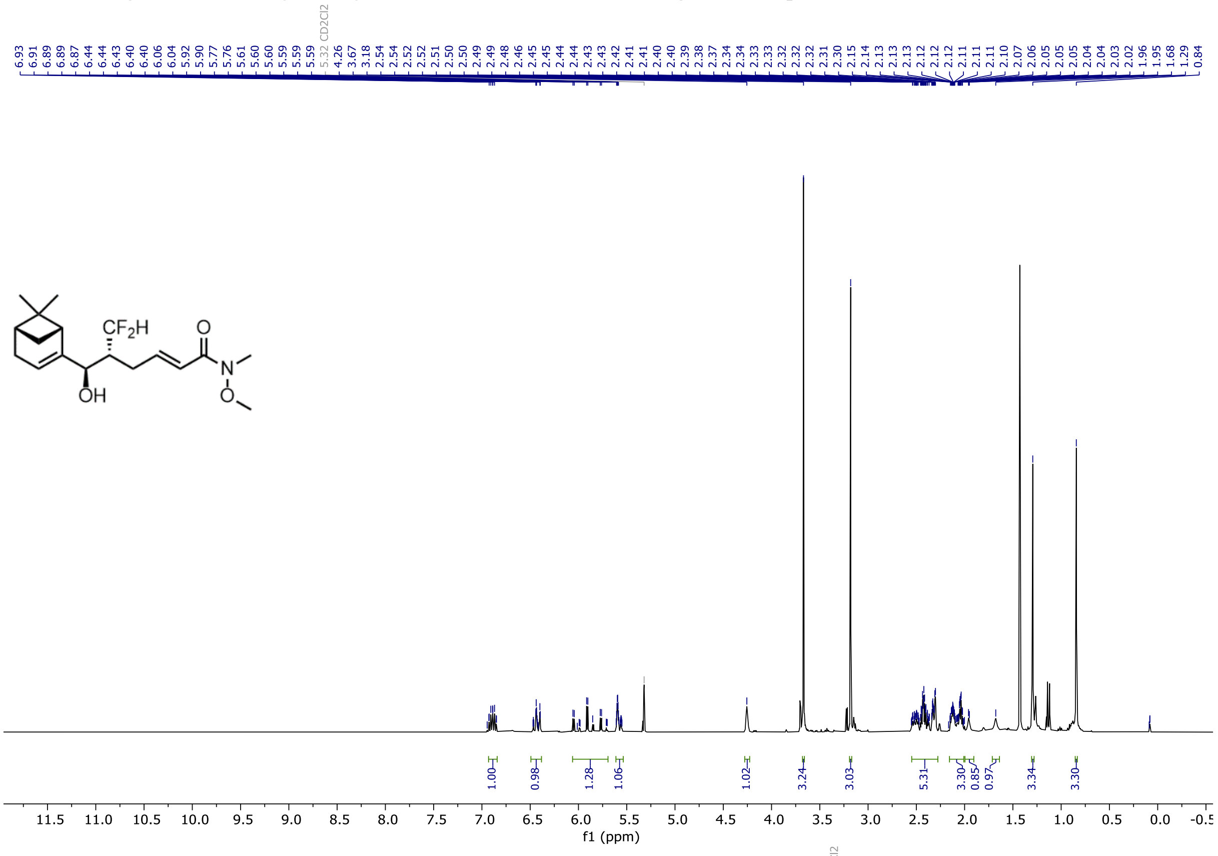

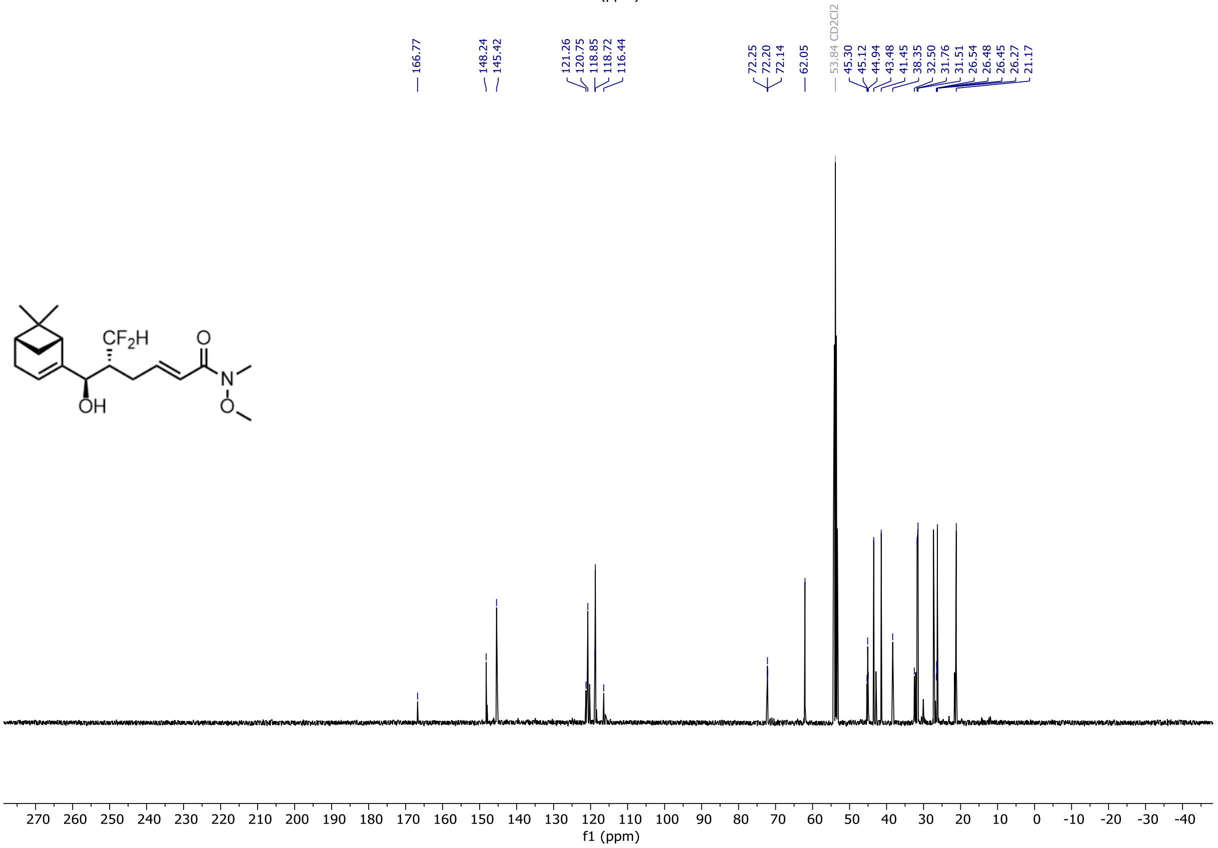

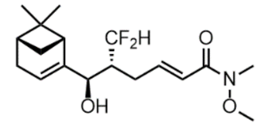

CF2H
O
N
O
OH

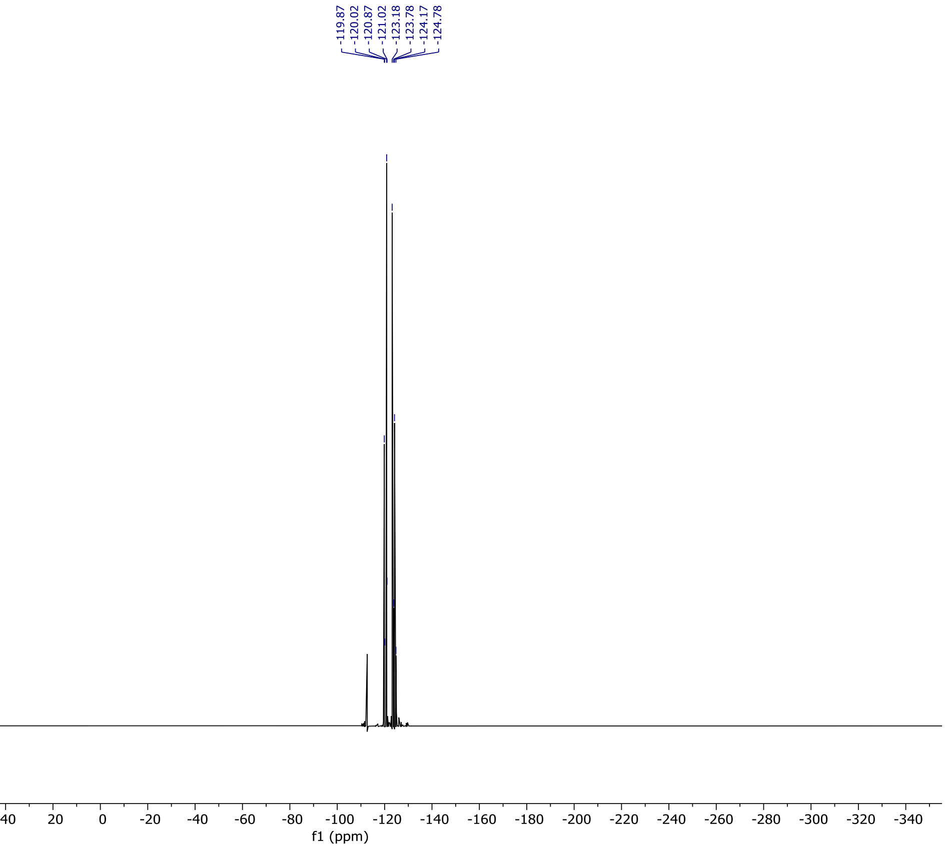

-119.87
-120.02
-120.87
-121.02
-123.18
-123.78
-124.17
-124.78
140 120 100 80 60 40 20 0 -20 -40 -60 -80 -100 -120 -140 -160 -180 -200 -220 -240 -260 -280 -300 -320 -340
f1 (ppm)

^{1}H NMR (400 MHz, CD_2Cl_2; top), ^{13}C NMR (101 MHz, CD_2Cl_2; middle) and ^{19}F NMR (282 MHz, CD_2Cl_2) of compound **21f (dr ≈ 4:1)**

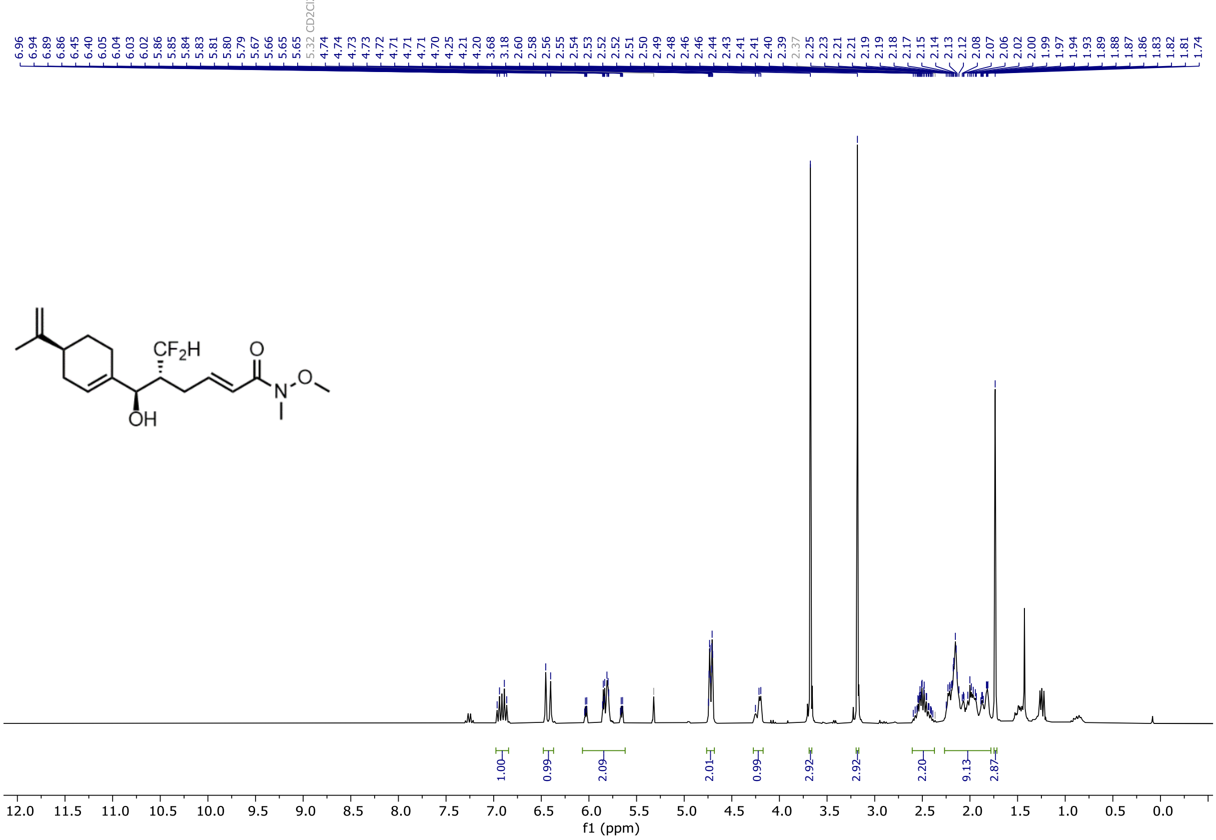

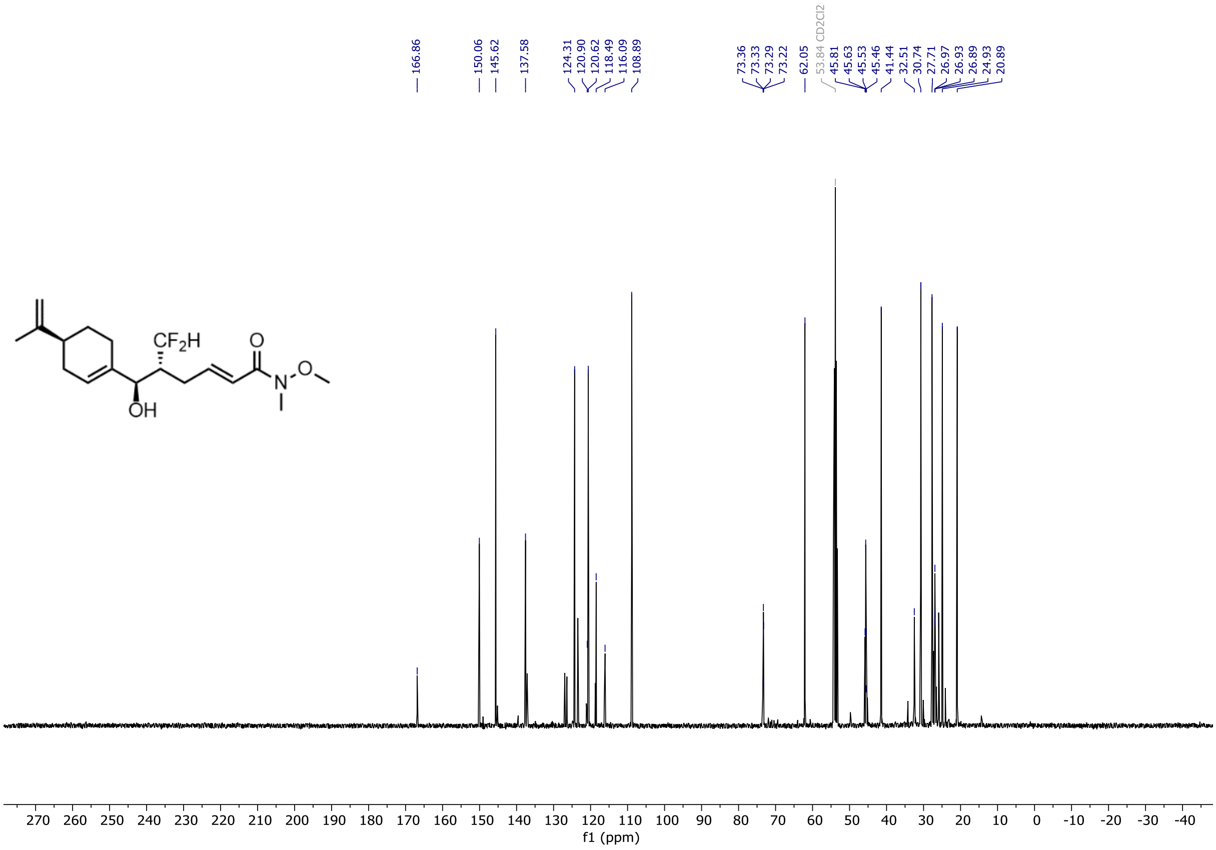

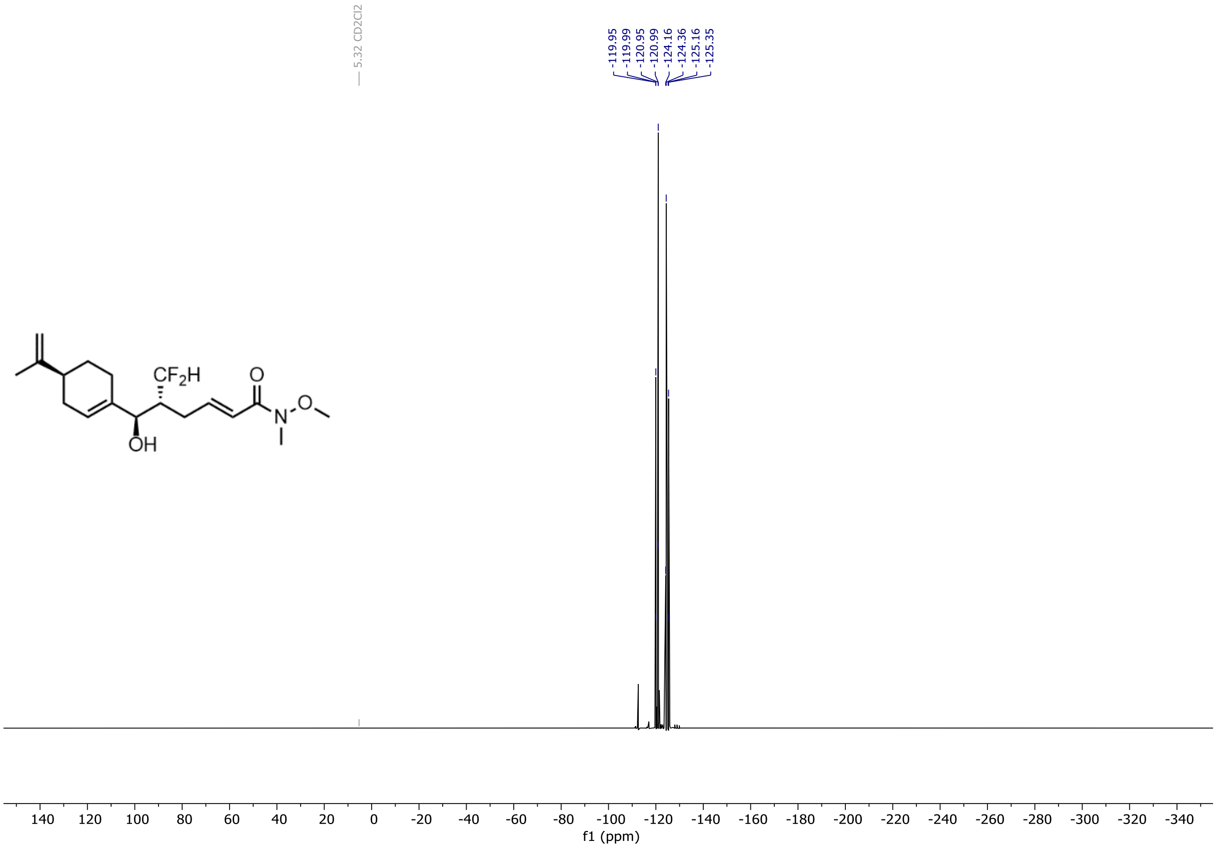
5.32 CD2Cl2
-119.95
-119.99
-120.95
-120.99
-124.16
-124.36
-125.16
-125.35
CF2H
O
N
O
OH
140 120 100 80 60 40 20 0 -20 -40 -60 -80 -100 -120 -140 -160 -180 -200 -220 -240 -260 -280 -300 -320 -340
f1 (ppm)

^{1}H NMR (400 MHz, $CDCl_3$; top), ^{13}C NMR (101 MHz, CD_2Cl_2; middle) and ^{19}F NMR (282 MHz, $CDCl_3$) of compound **S2**

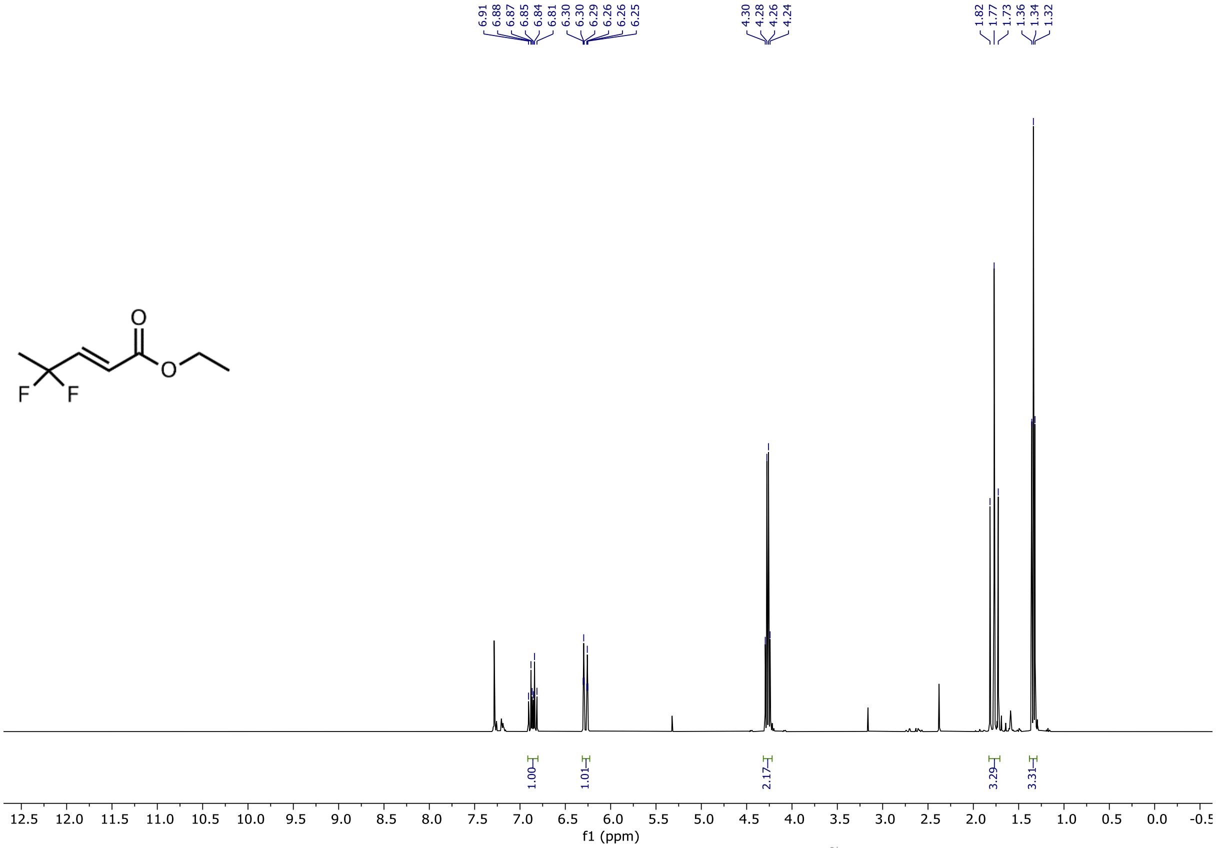

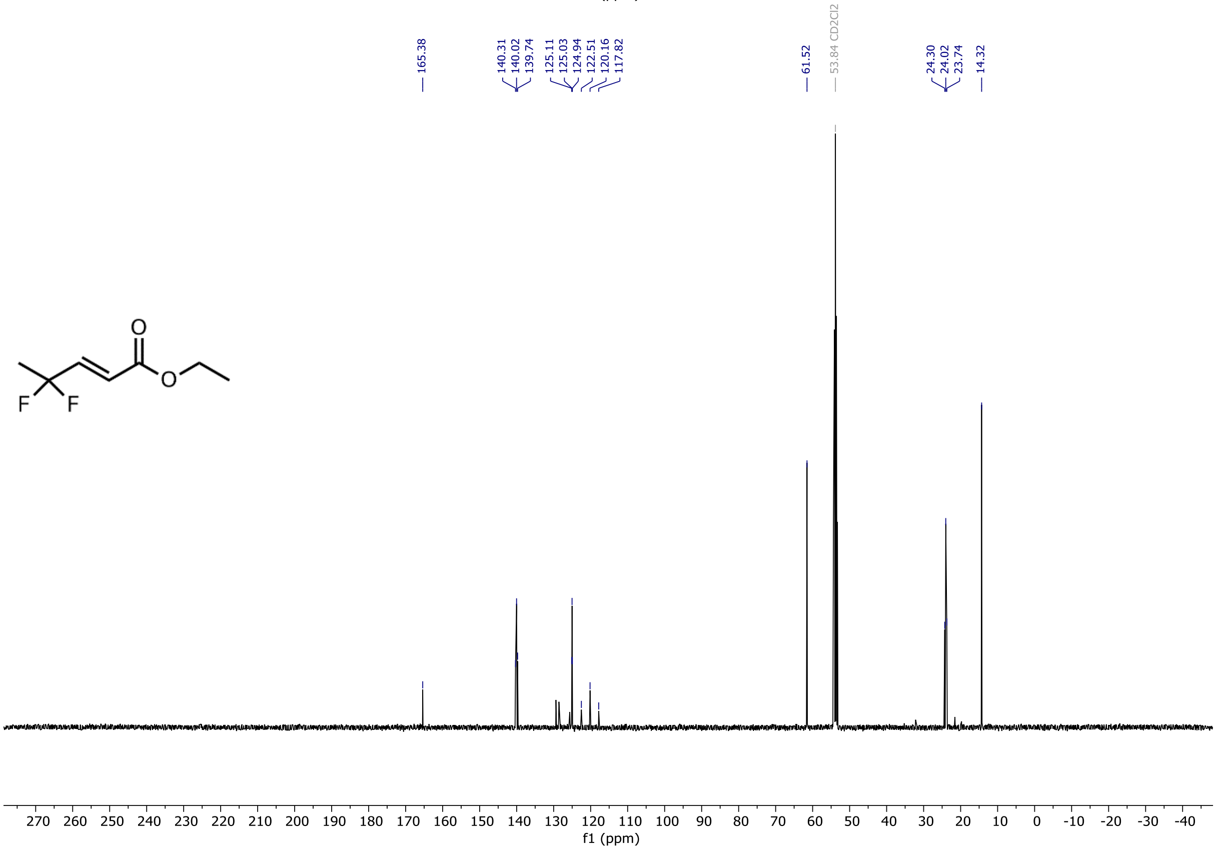

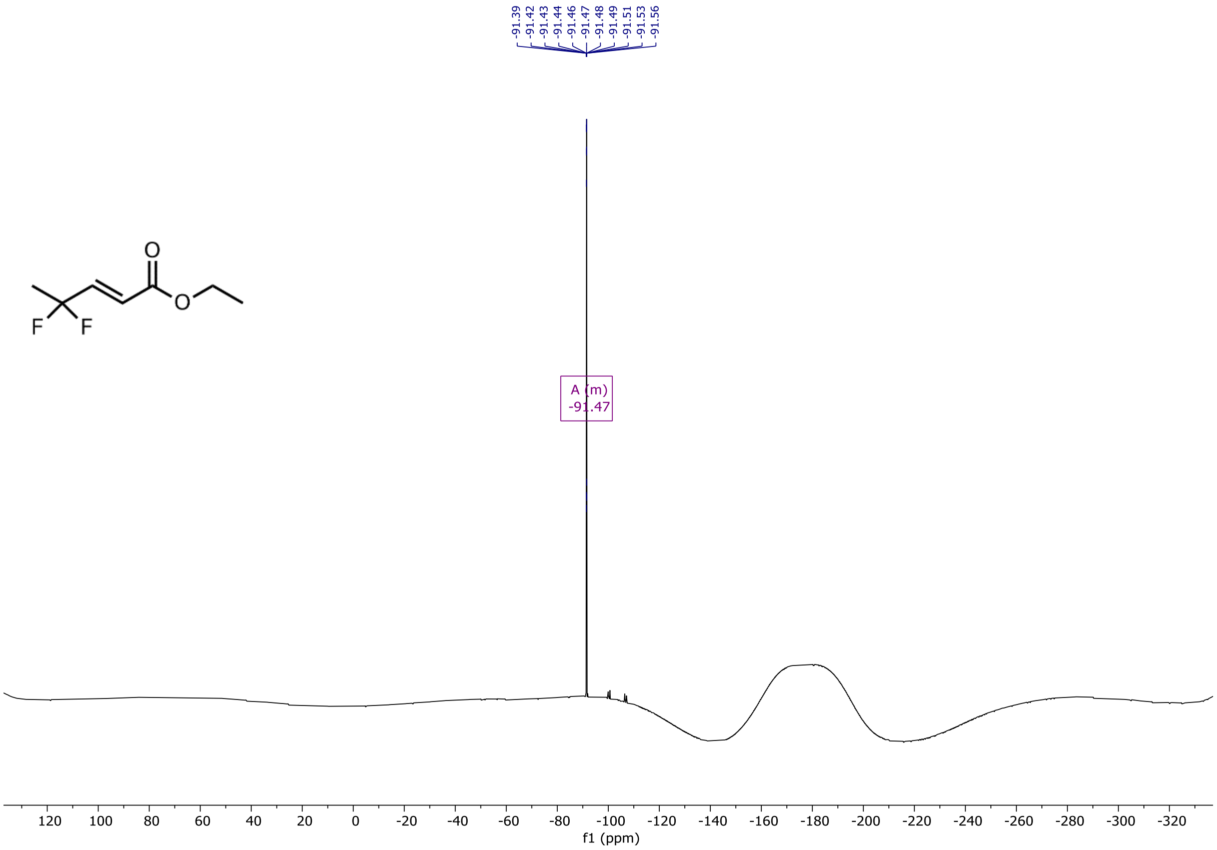
-91.39
-91.42
-91.43
-91.44
-91.46
-91.47
-91.48
-91.49
-91.51
-91.53
-91.56
A (m)
-91.47
120 100 80 60 40 20 0 -20 -40 -60 -80 -100 -120 -140 -160 -180 -200 -220 -240 -260 -280 -300 -320
f1 (ppm)

^{1}H NMR (400 MHz, CD_2Cl_2; top), ^{13}C NMR (101 MHz, CD_2Cl_2; middle) and ^{19}F NMR (282 MHz, CD_2Cl_2) of compound **22**

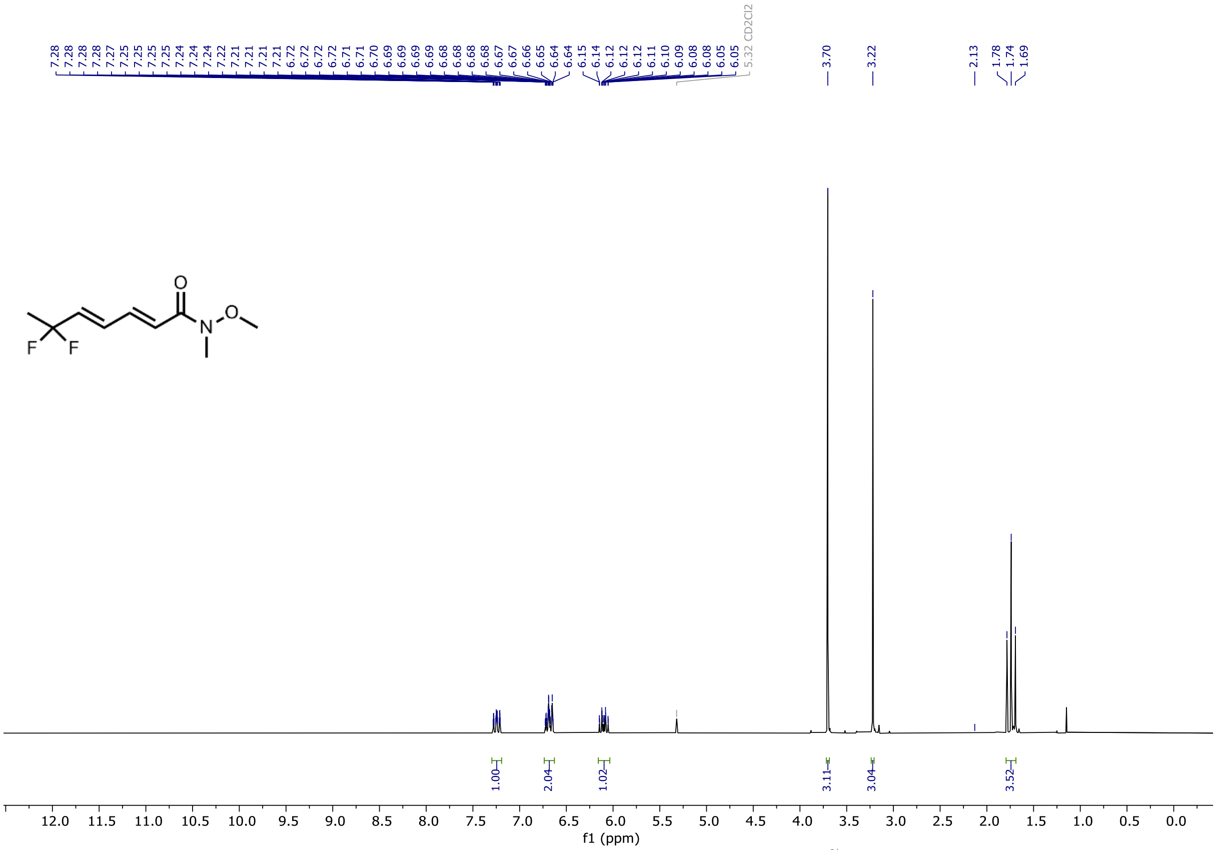

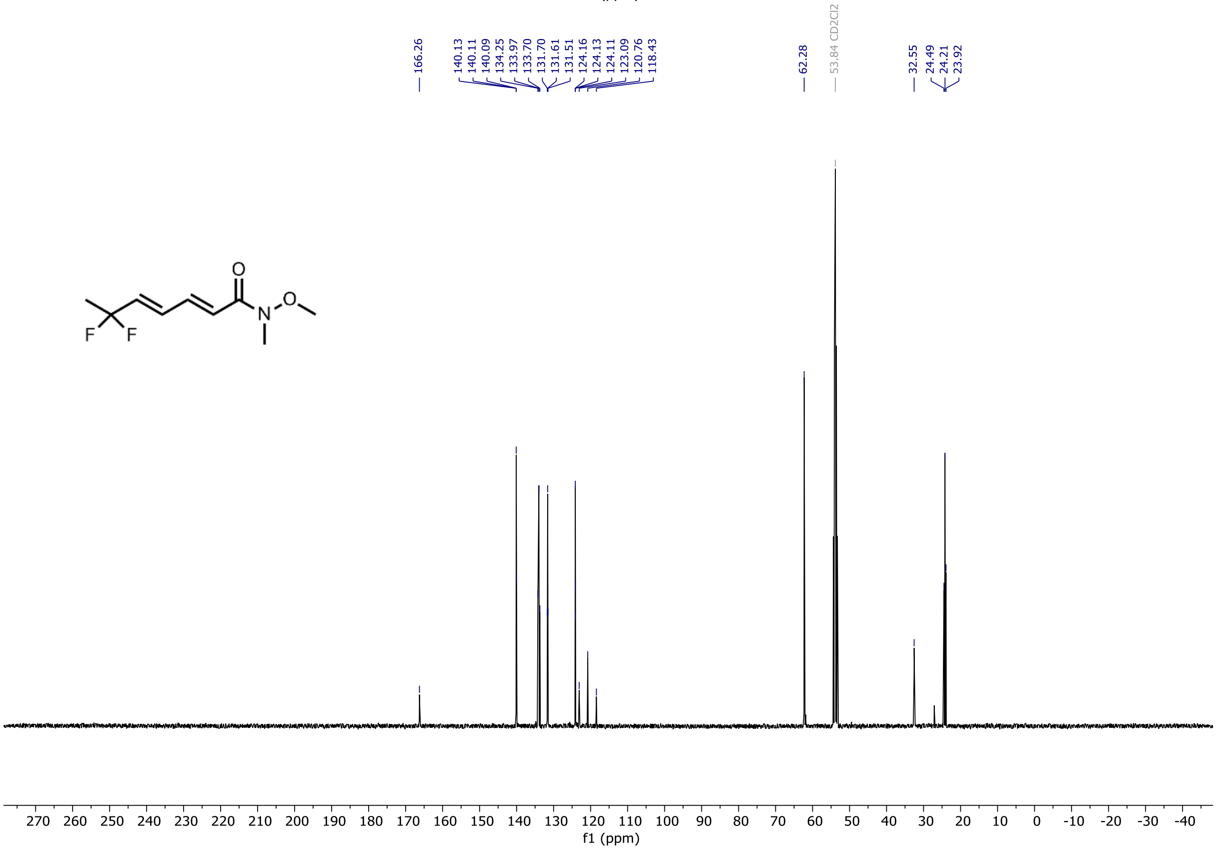

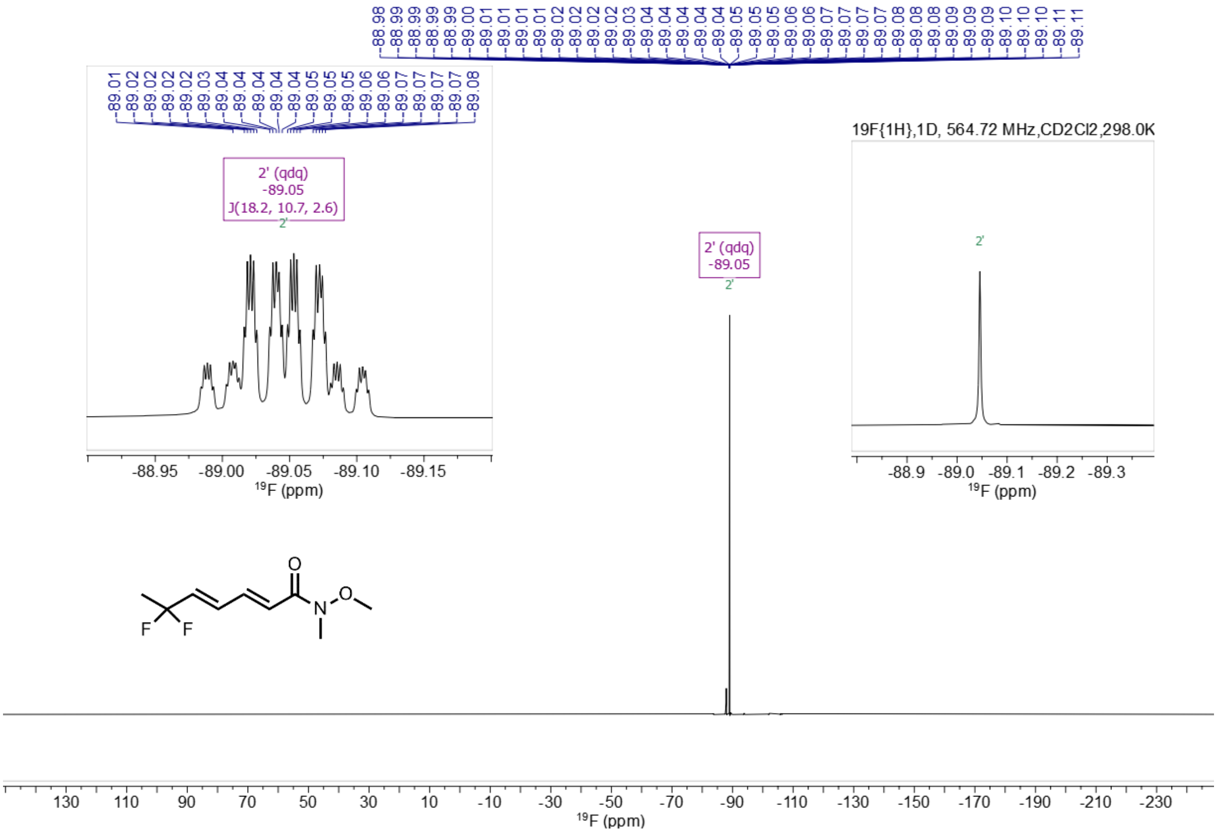

19F{1H},1D, 564.72 MHz,CD2Cl2,298.0K
2' (qdq)
-89.05
J(18.2, 10.7, 2.6)
2' (qdq)
-89.05
-88.95 -89.00 -89.05 -89.10 -89.15
-88.9 -89.0 -89.1 -89.2 -89.3
130 110 90 70 50 30 10 -10 -30 -50 -70 -90 -110 -130 -150 -170 -190 -210 -230
^{19}F (ppm)

^{1}H NMR (400 MHz, CD_2Cl_2; top), ^{13}C NMR (101 MHz, CD_2Cl_2; middle) and ^{19}F NMR (282 MHz, CD_2Cl_2) of compound **23a**

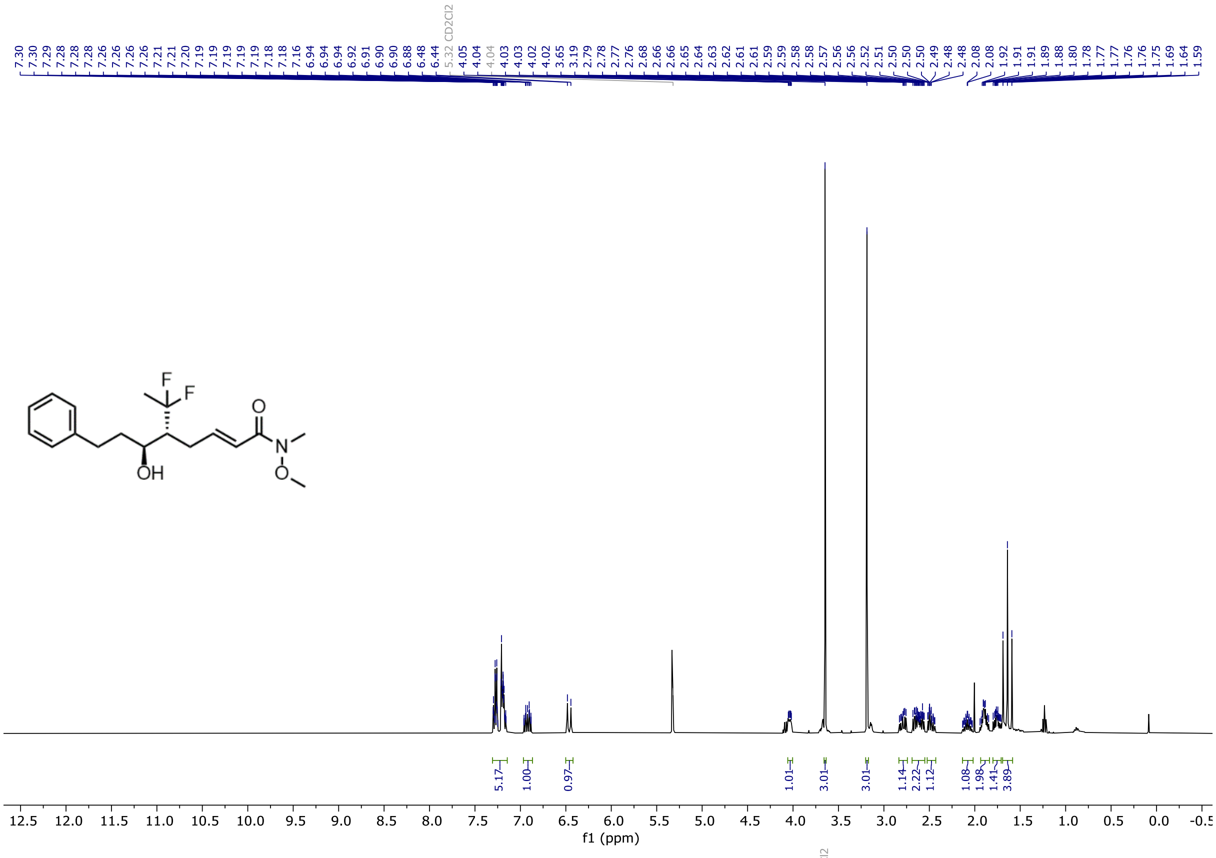

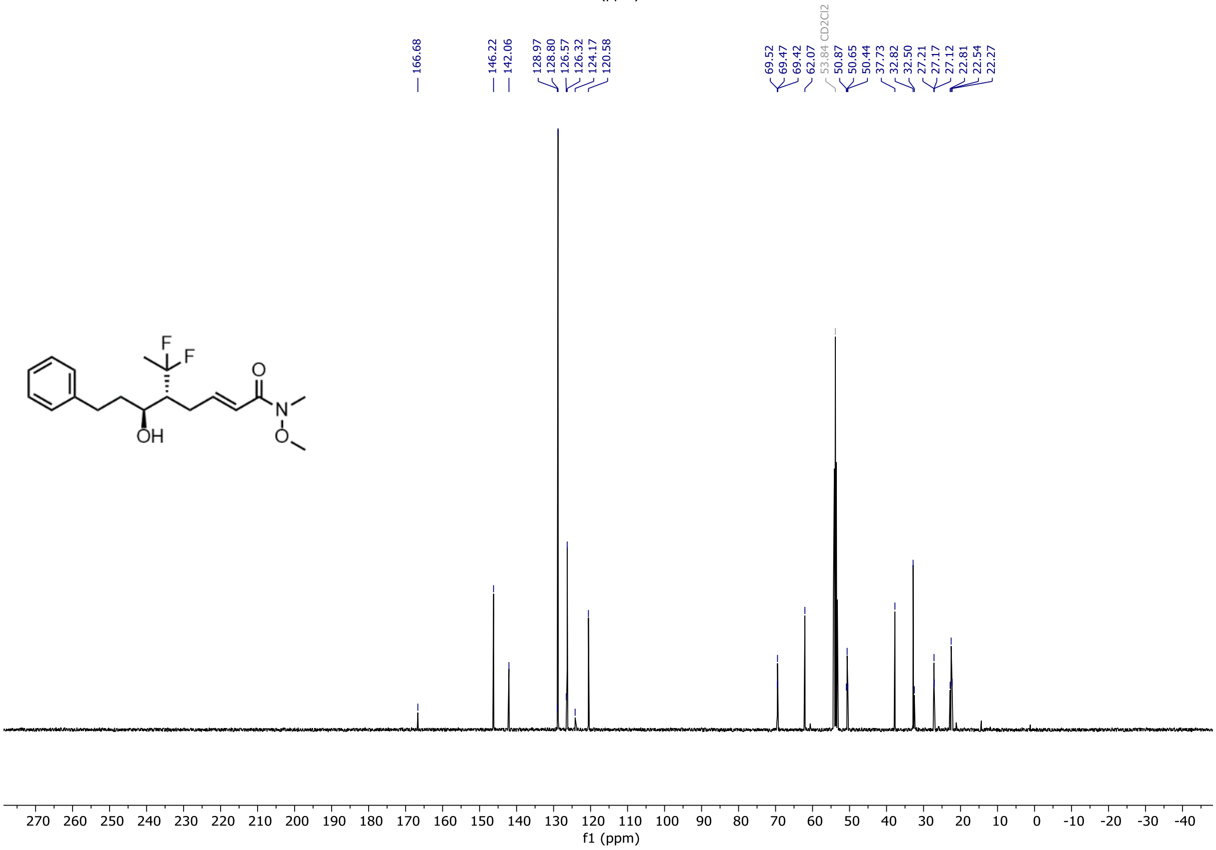

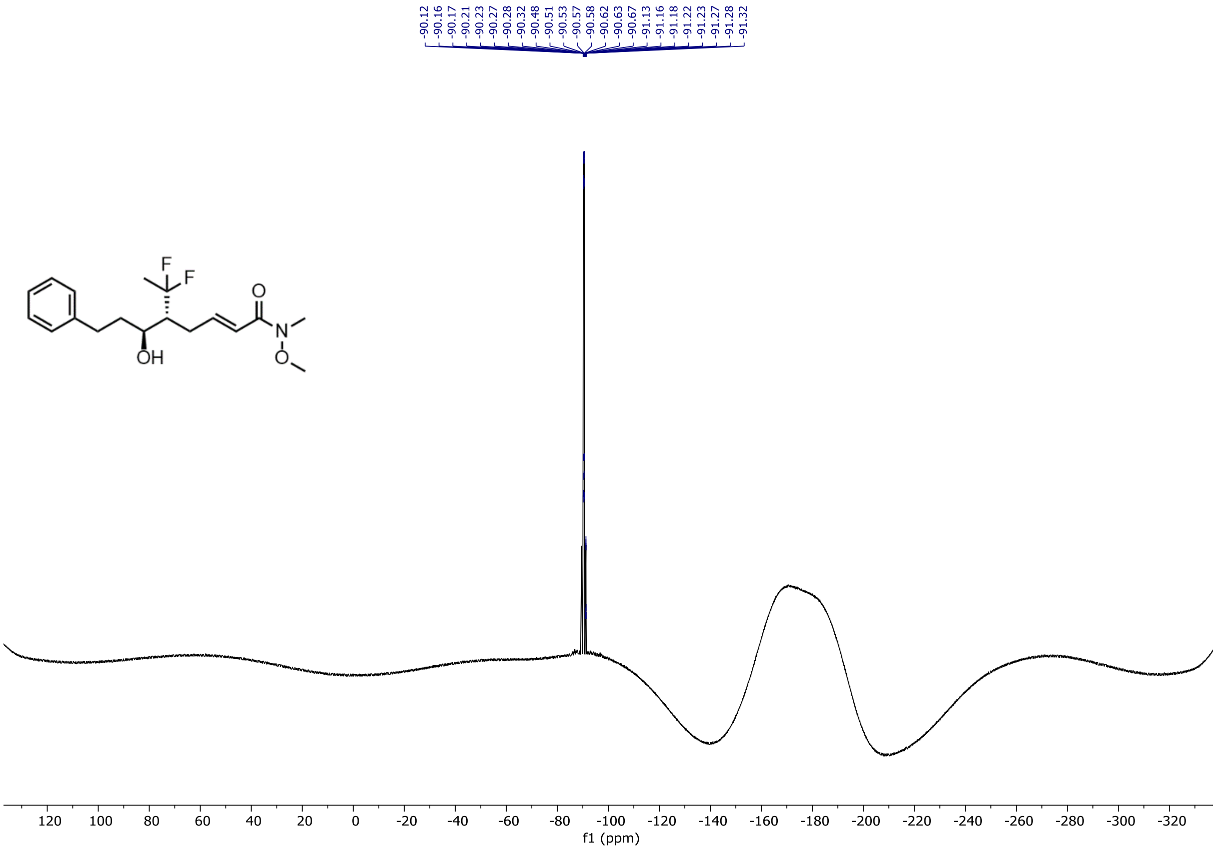
-90.12
-90.16
-90.17
-90.21
-90.23
-90.27
-90.28
-90.32
-90.48
-90.51
-90.53
-90.57
-90.58
-90.62
-90.63
-90.67
-91.13
-91.16
-91.18
-91.22
-91.23
-91.27
-91.28
-91.32
F
F
O
N
O
OH
120 100 80 60 40 20 0 -20 -40 -60 -80 -100 -120 -140 -160 -180 -200 -220 -240 -260 -280 -300 -320
f1 (ppm)

^{1}H NMR (400 MHz, CD_2Cl_2; top), ^{13}C NMR (101 MHz, CD_2Cl_2; middle) and ^{19}F NMR (282 MHz, CD_2Cl_2) of compound **23b**

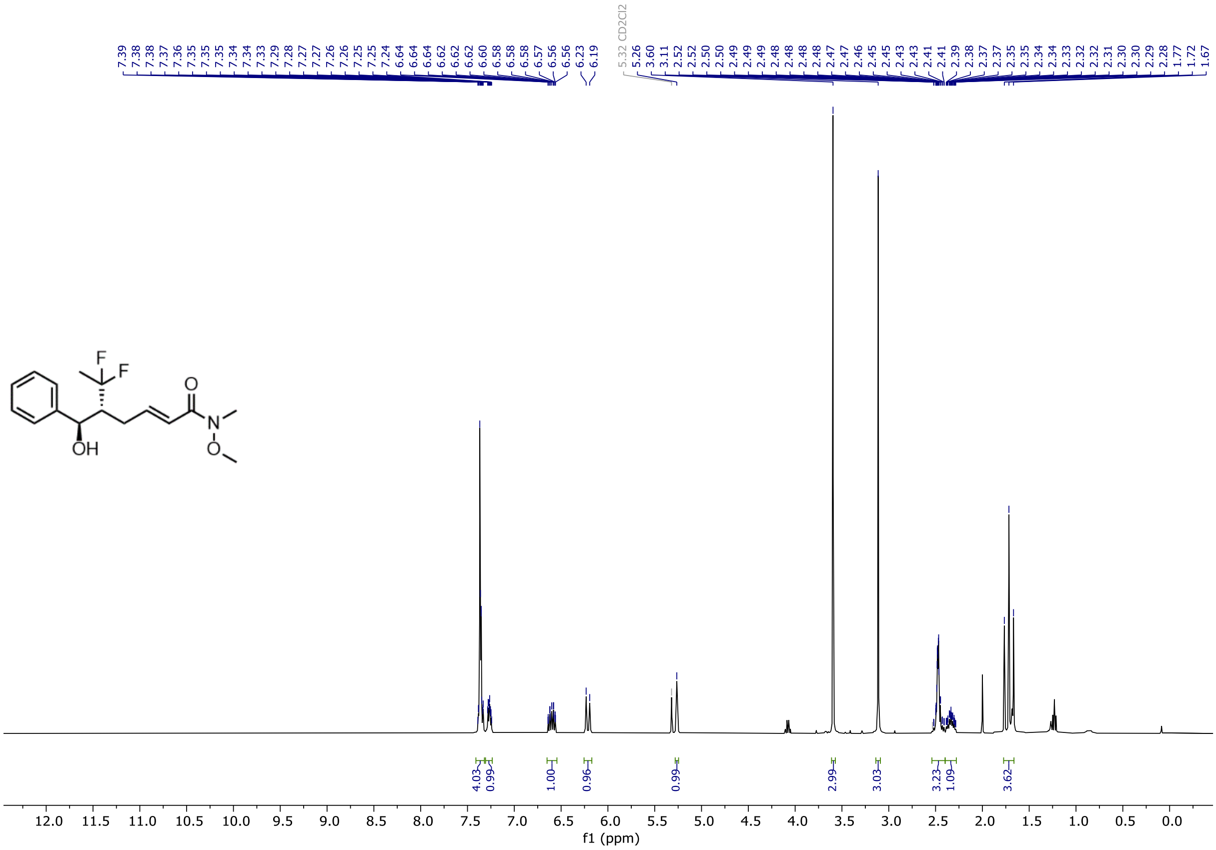

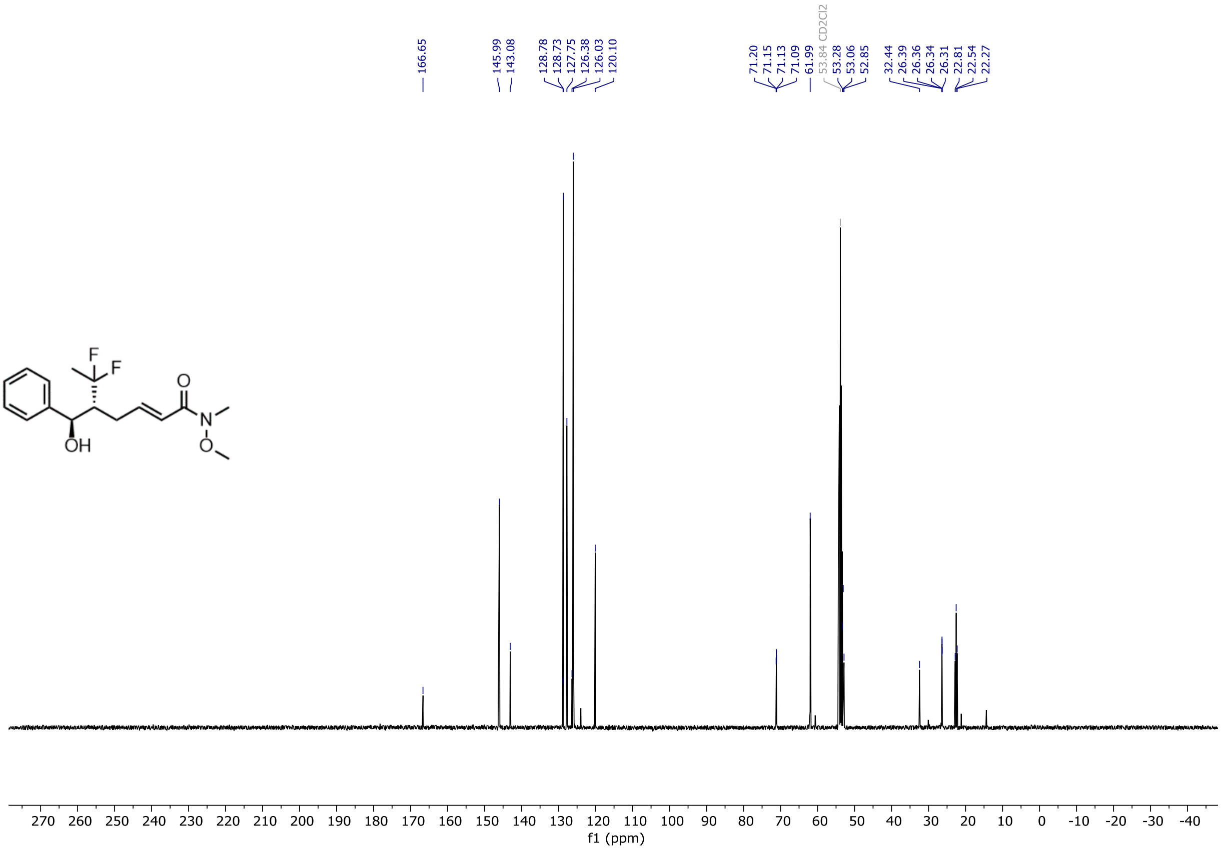

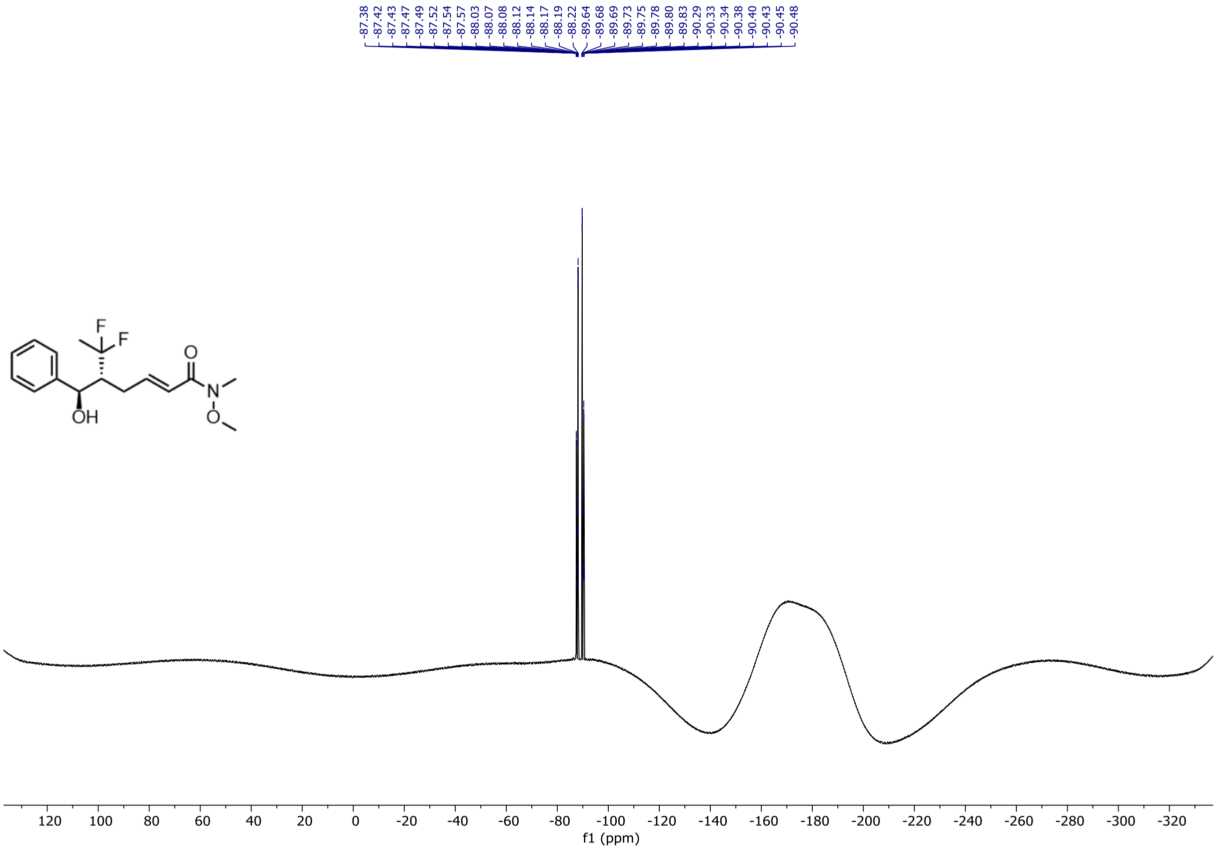

-87.38
-87.42
-87.43
-87.47
-87.49
-87.52
-87.54
-87.57
-88.03
-88.07
-88.08
-88.12
-88.14
-88.17
-88.19
-88.22
-89.64
-89.68
-89.69
-89.73
-89.75
-89.78
-89.80
-89.83
-90.29
-90.33
-90.34
-90.38
-90.40
-90.43
-90.45
-90.48
F
F
O
N
O
OH
120
100
80
60
40
20
0
-20
-40
-60
-80
-100
-120
-140
-160
-180
-200
-220
-240
-260
-280
-300
-320
f1 (ppm)

^{1}H NMR (400 MHz, $CDCl_3$; top), ^{13}C NMR (101 MHz, $CDCl_3$; middle) and ^{19}F NMR (282 MHz, $CDCl_3$) of compound **23c**

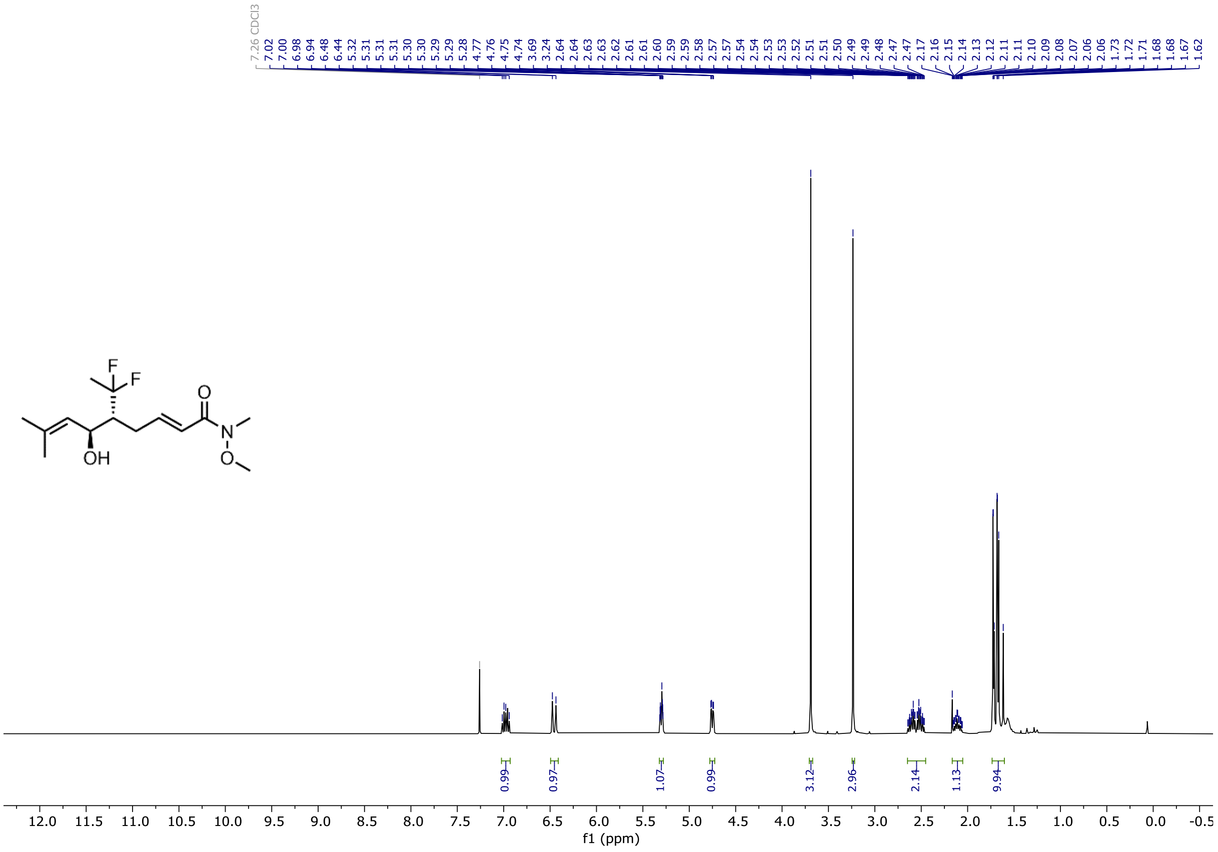

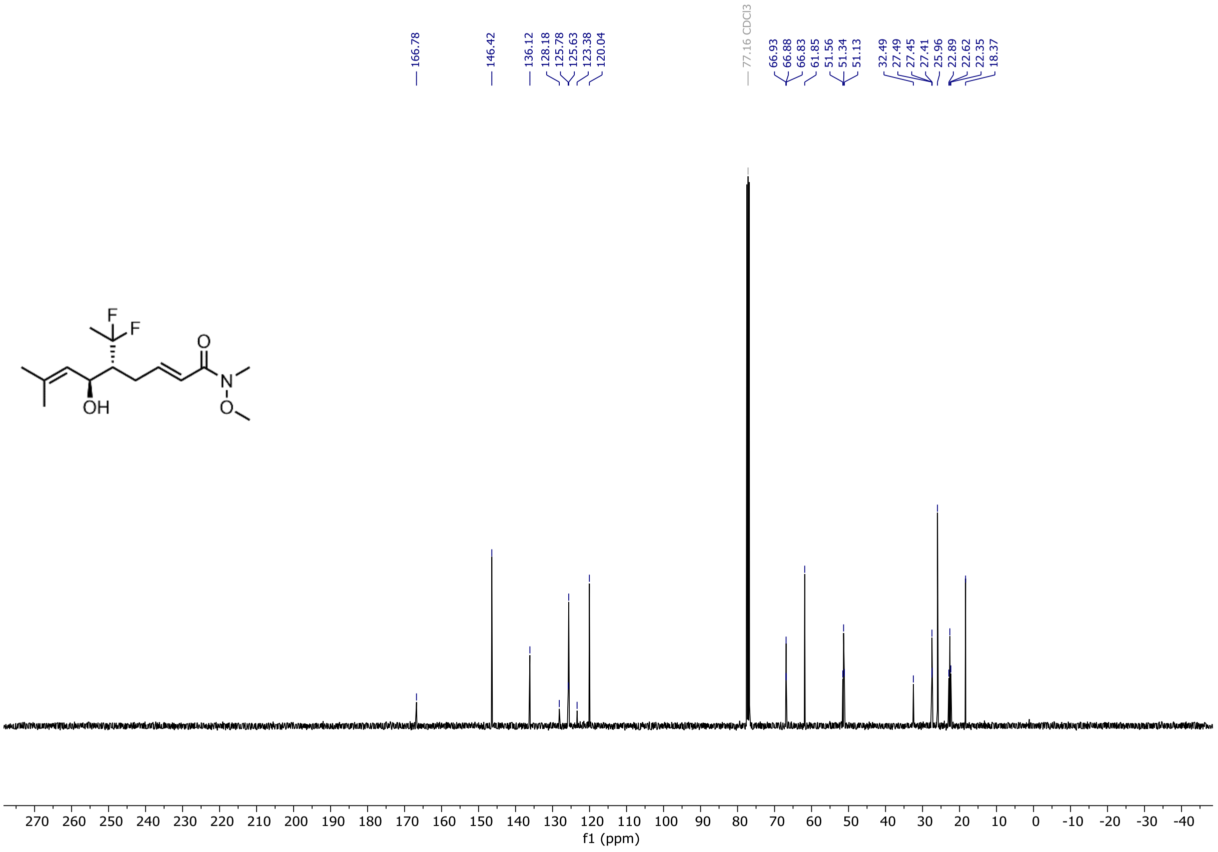

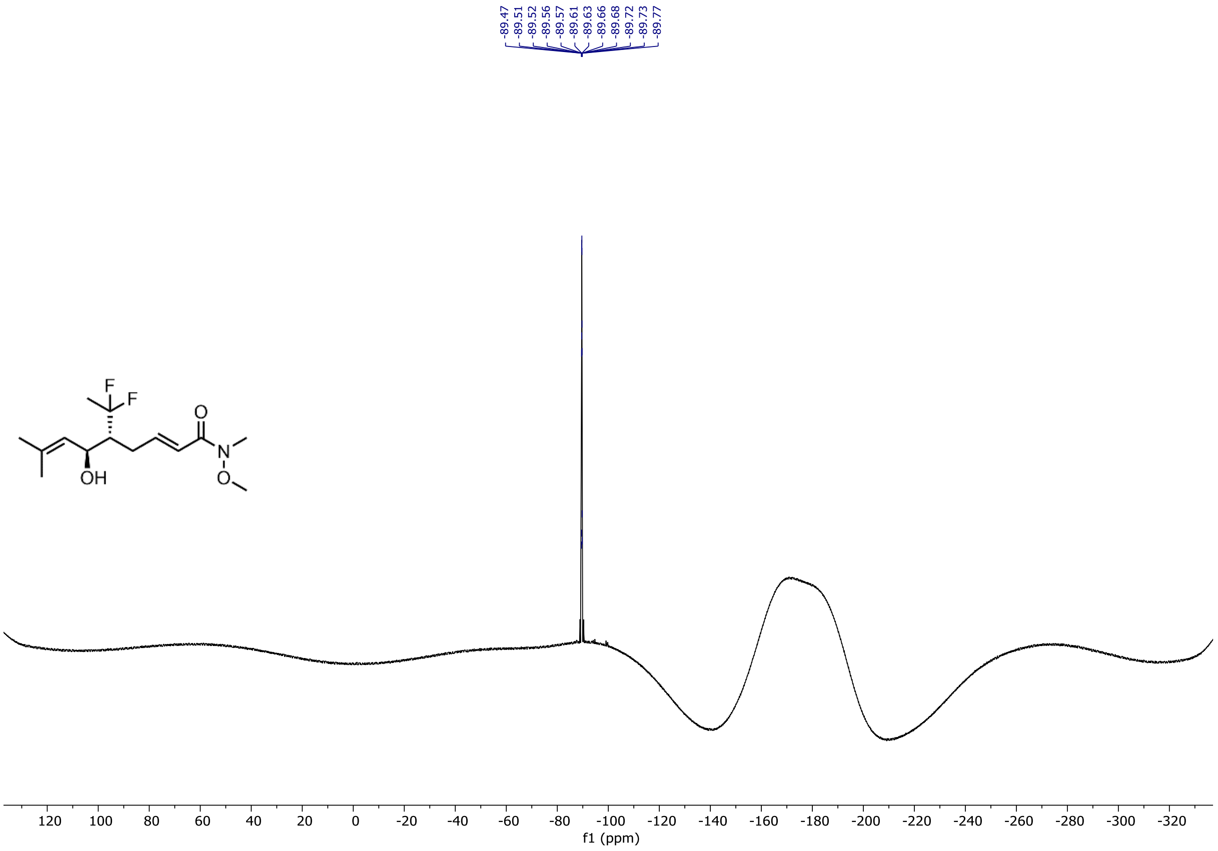
-89.47
-89.51
-89.52
-89.56
-89.57
-89.61
-89.63
-89.66
-89.68
-89.72
-89.73
-89.77
F
F
O
N
O
OH
120 100 80 60 40 20 0 -20 -40 -60 -80 -100 -120 -140 -160 -180 -200 -220 -240 -260 -280 -300 -320
f1 (ppm)

^{1}H NMR (400 MHz, CD_2Cl_2; top), ^{13}C NMR (101 MHz, CD_2Cl_2; middle) and ^{19}F NMR (282 MHz, CD_2Cl_2) of compound **24a**

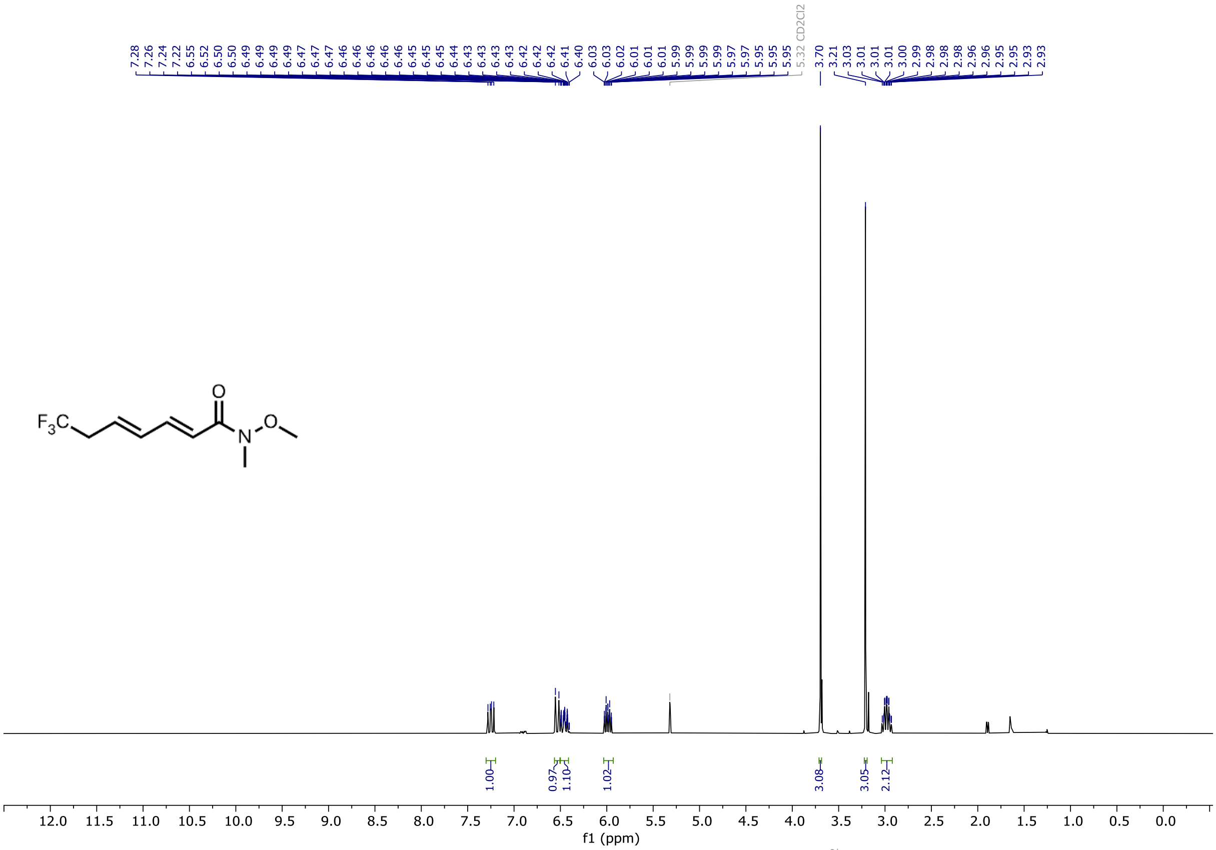

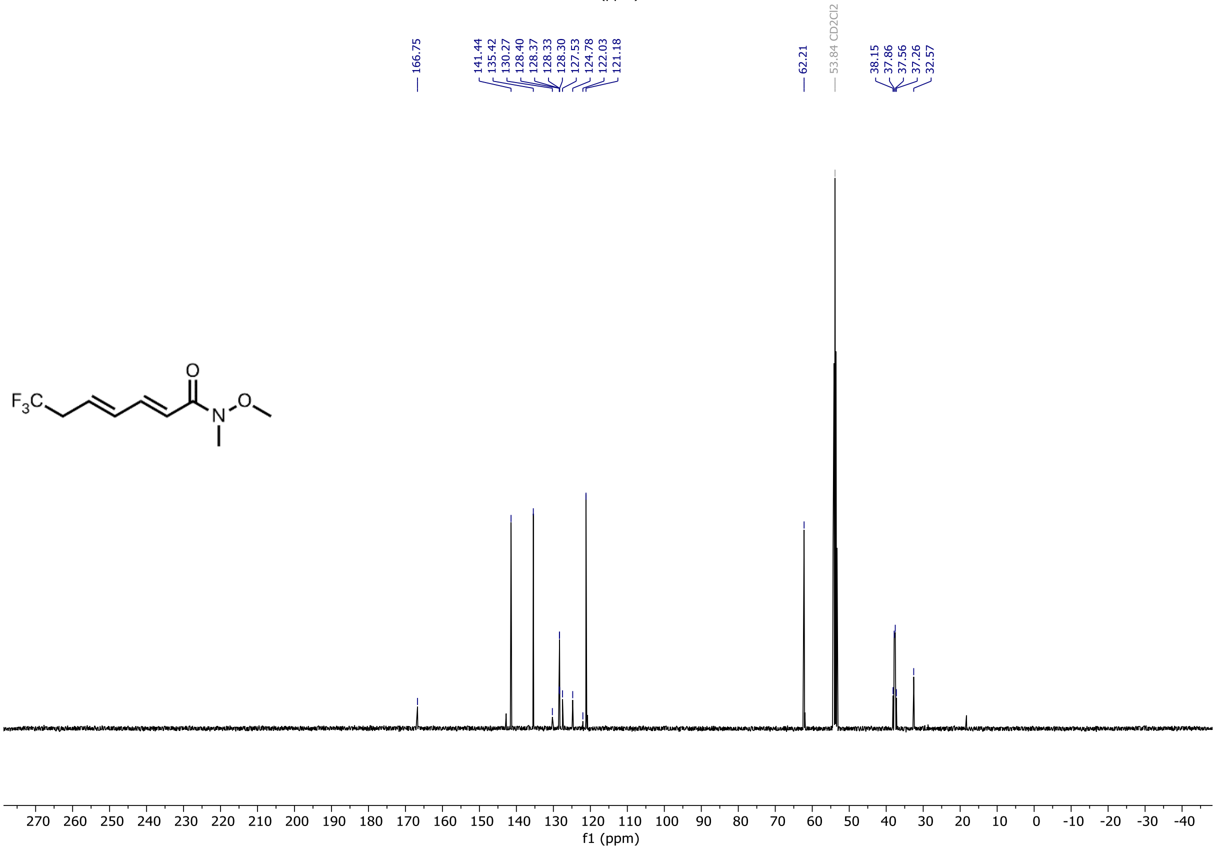

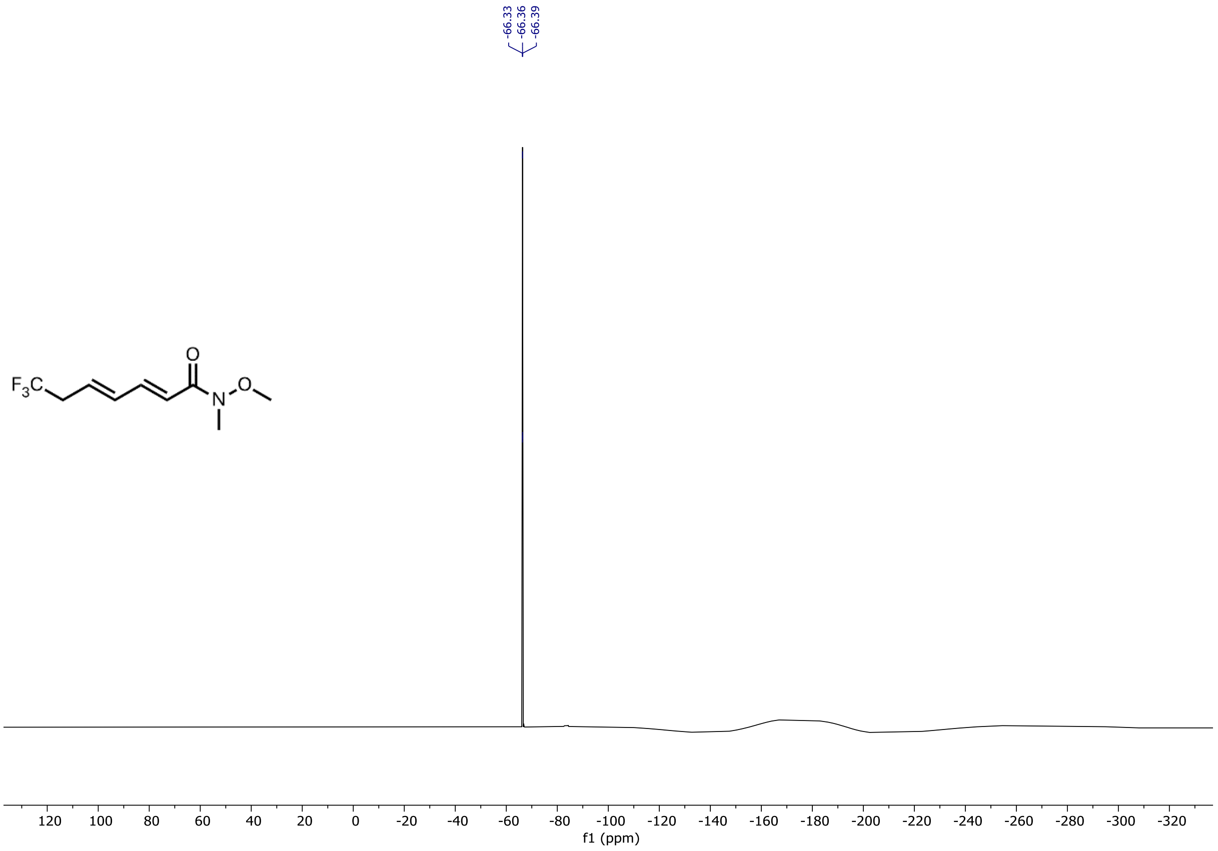
F3C
O
N
O
-66.33
-66.36
-66.39
120 100 80 60 40 20 0 -20 -40 -60 -80 -100 -120 -140 -160 -180 -200 -220 -240 -260 -280 -300 -320
f1 (ppm)

^{1}H NMR (400 MHz, $CDCl_3$; top), ^{13}C NMR (101 MHz, $CDCl_3$; middle) and ^{19}F NMR (282 MHz, $CDCl_3$) of compound **S4**

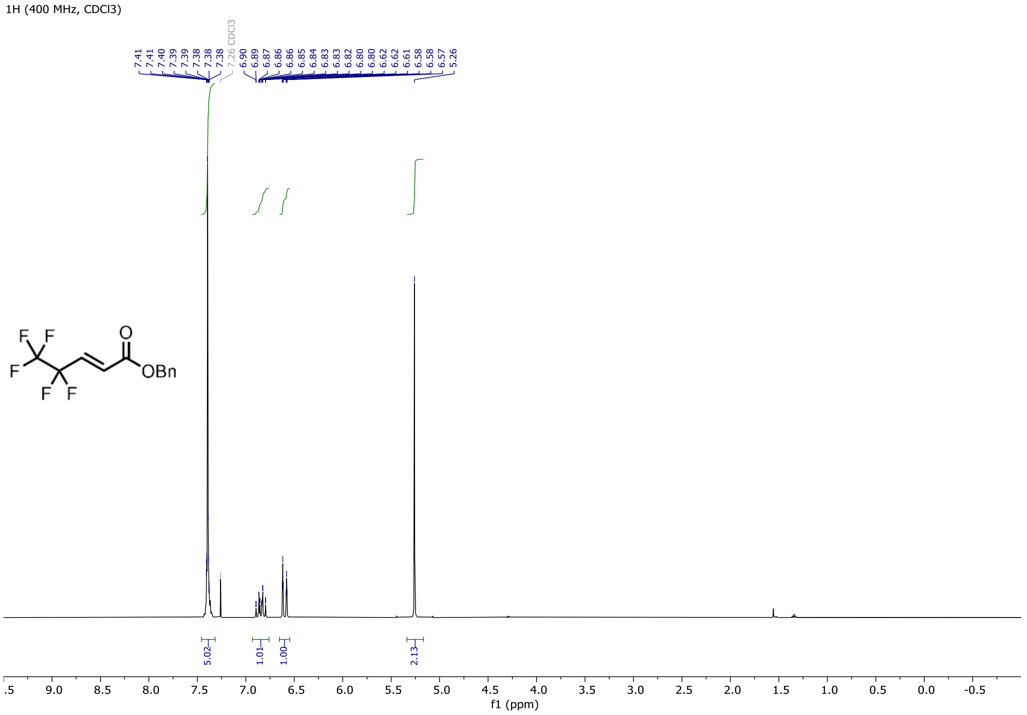

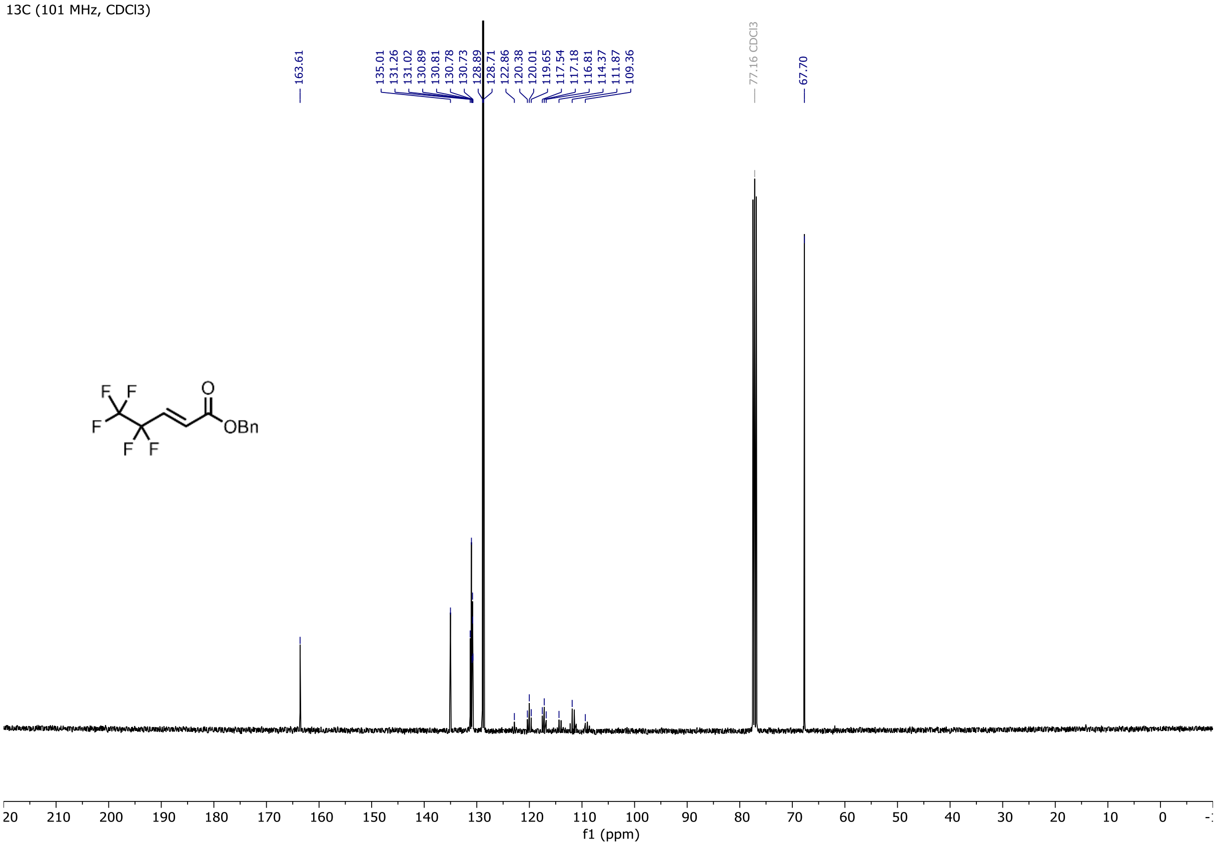

19F (282 MHz, CDCl3)

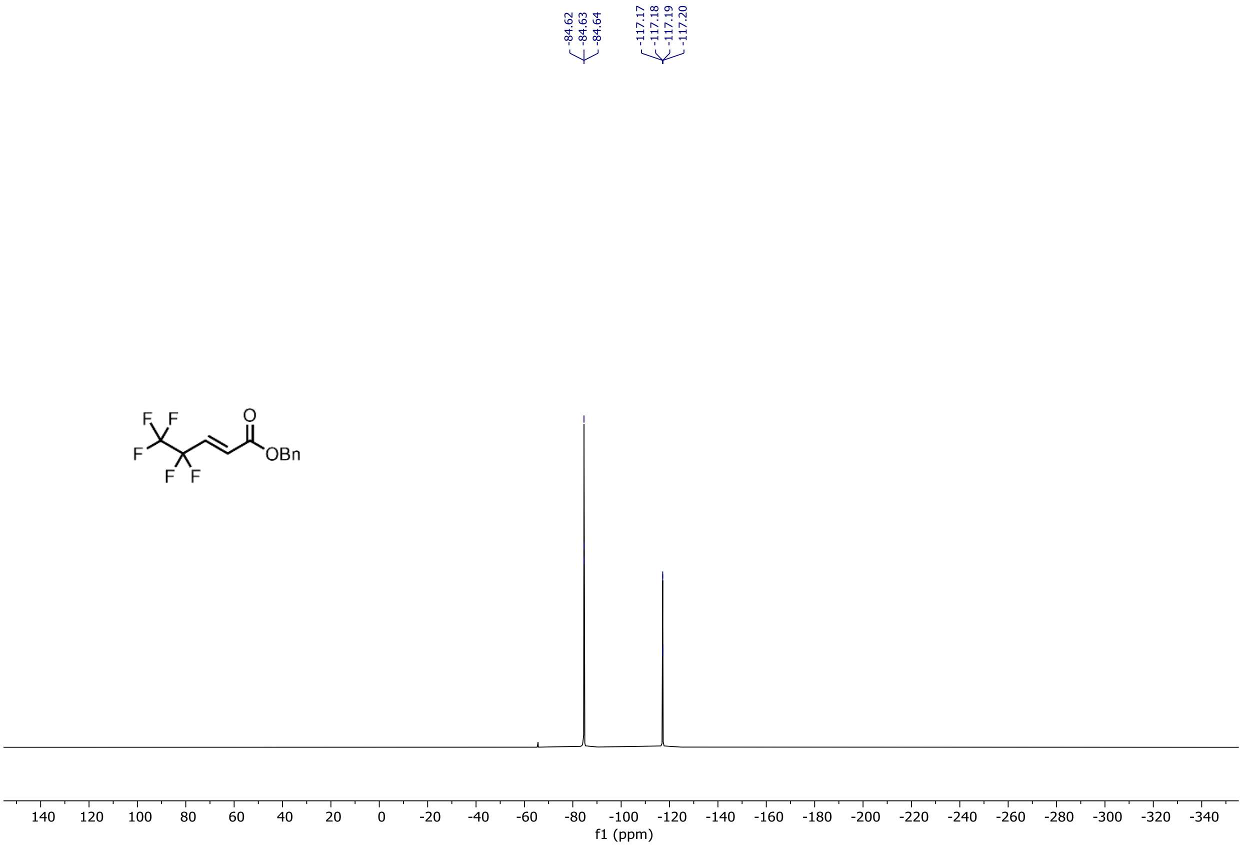

^1H NMR (400 MHz, CD_2Cl_2; top), ^{13}C NMR (101 MHz, CD_2Cl_2; middle) and ^{19}F NMR (282 MHz, CD_2Cl_2) of compound **24b**

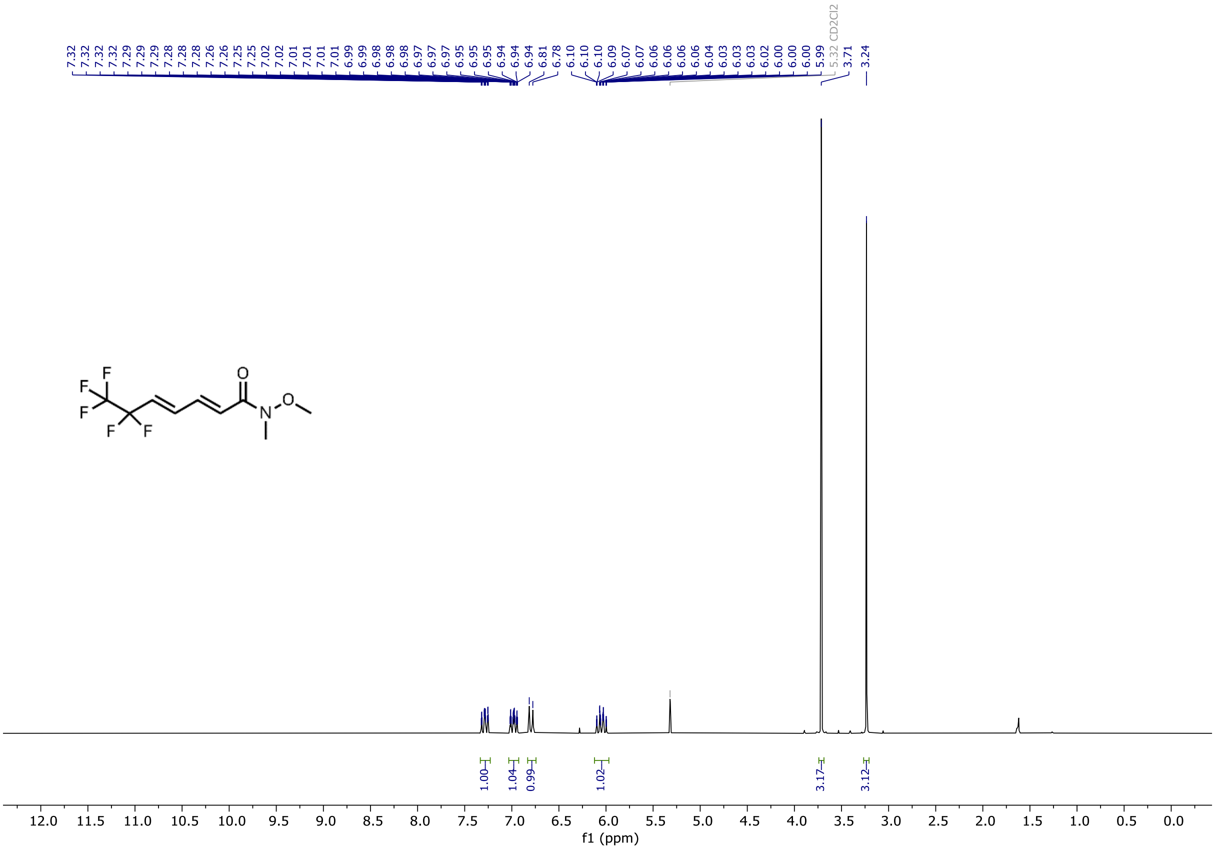

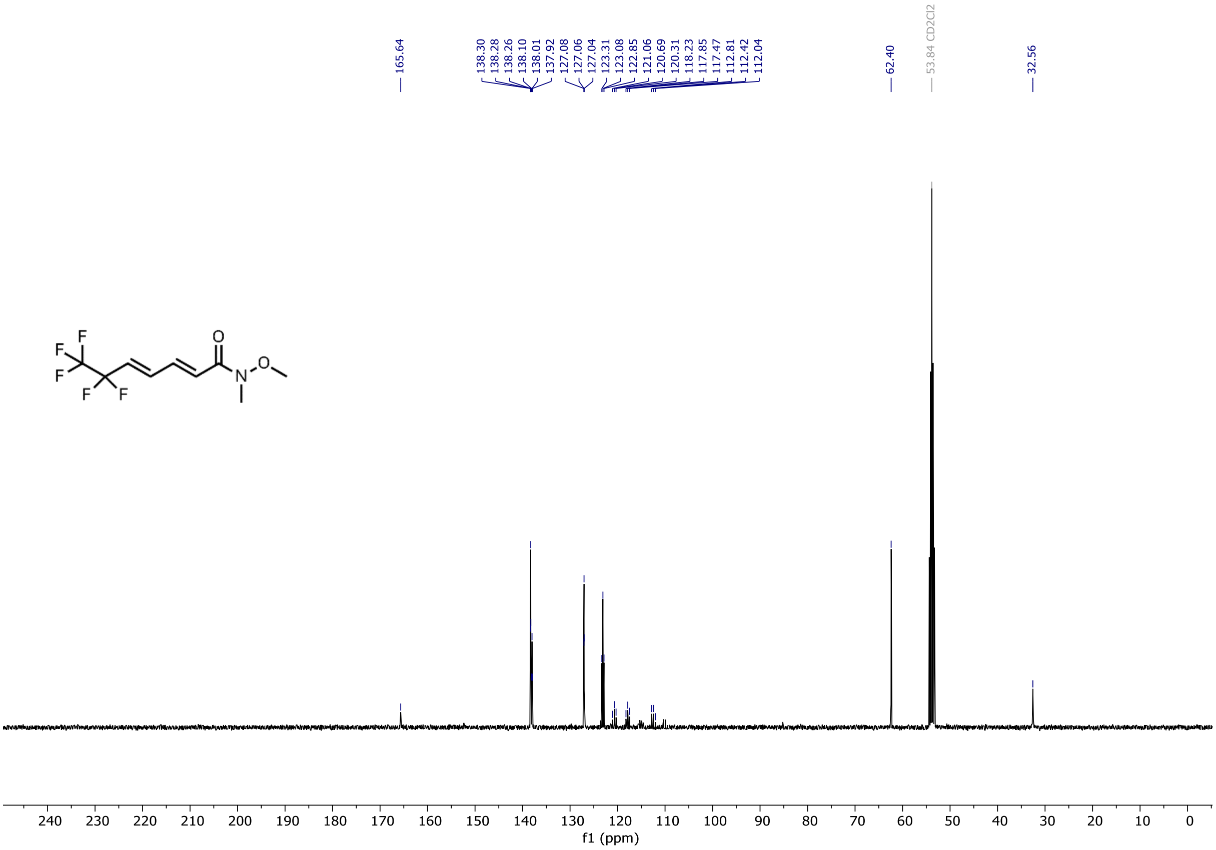

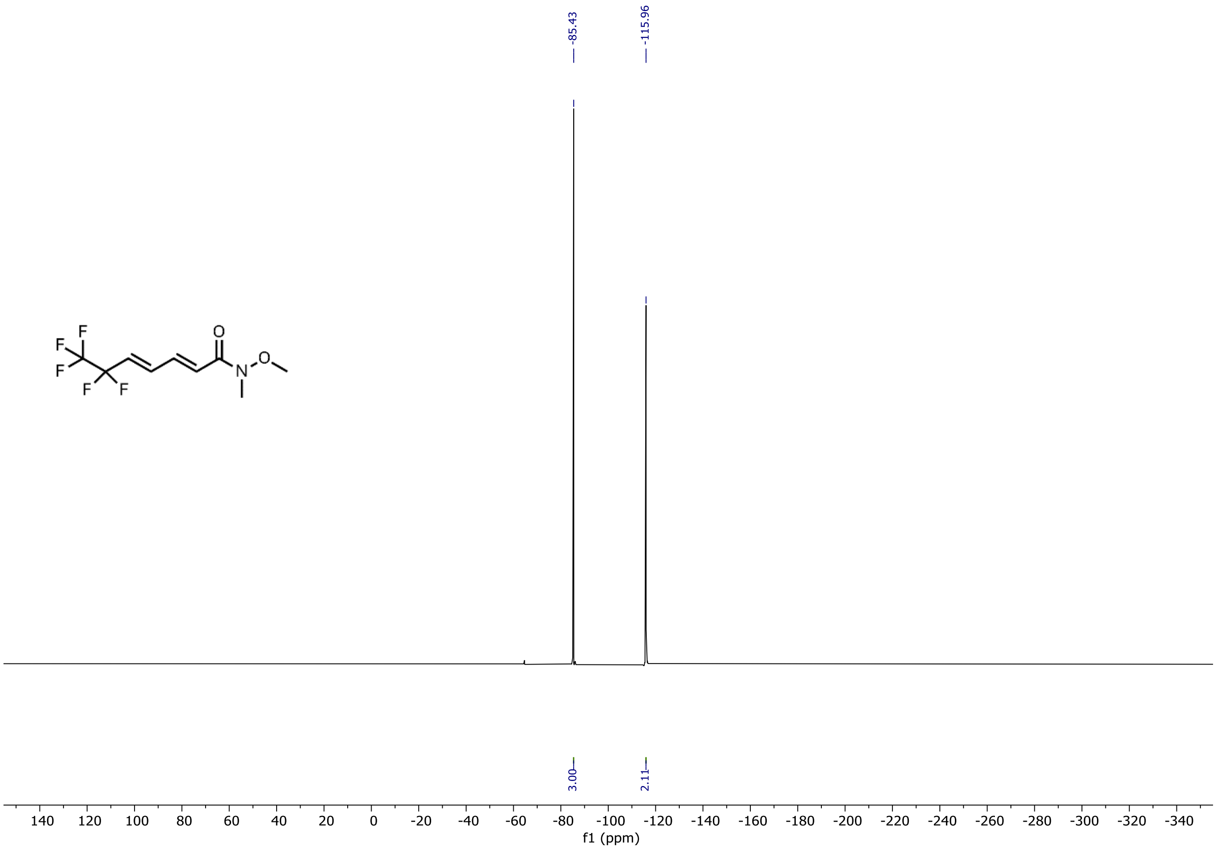
-85.43
-115.96
F
F
F
F
F
O
N
O
3.00
2.11
140 120 100 80 60 40 20 0 -20 -40 -60 -80 -100 -120 -140 -160 -180 -200 -220 -240 -260 -280 -300 -320 -340
f1 (ppm)

^{1}H NMR (400 MHz, CD_2Cl_2; top), ^{13}C NMR (101 MHz, CD_2Cl_2; middle) and ^{19}F NMR (282 MHz, CD_2Cl_2) of compound **25a**

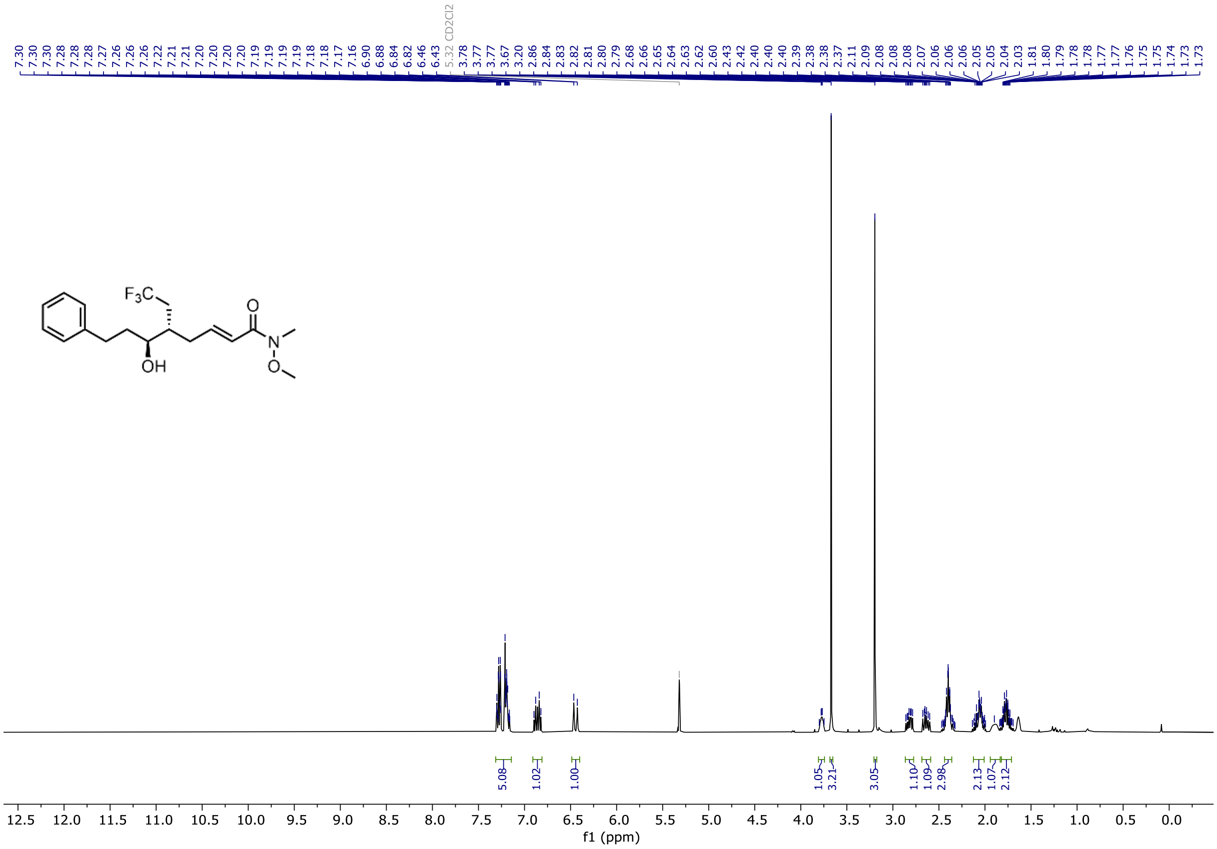

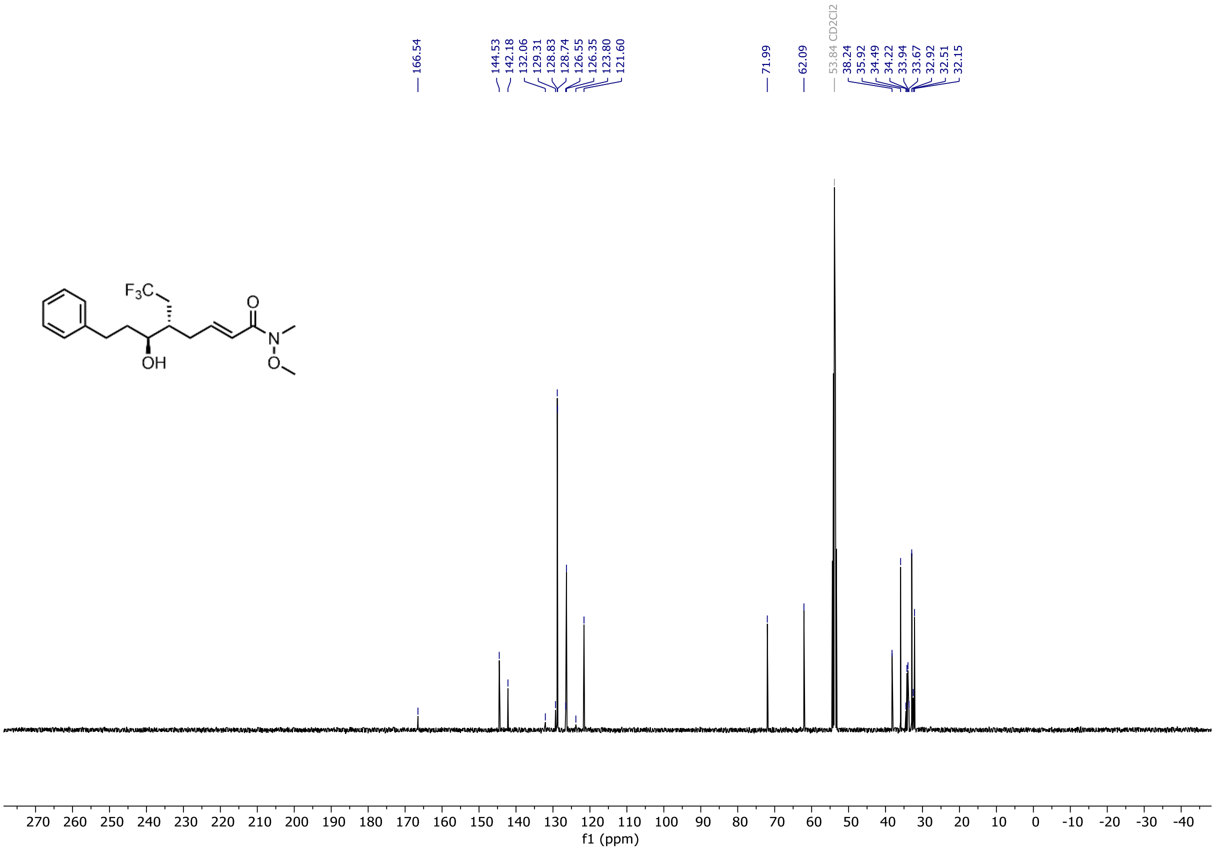

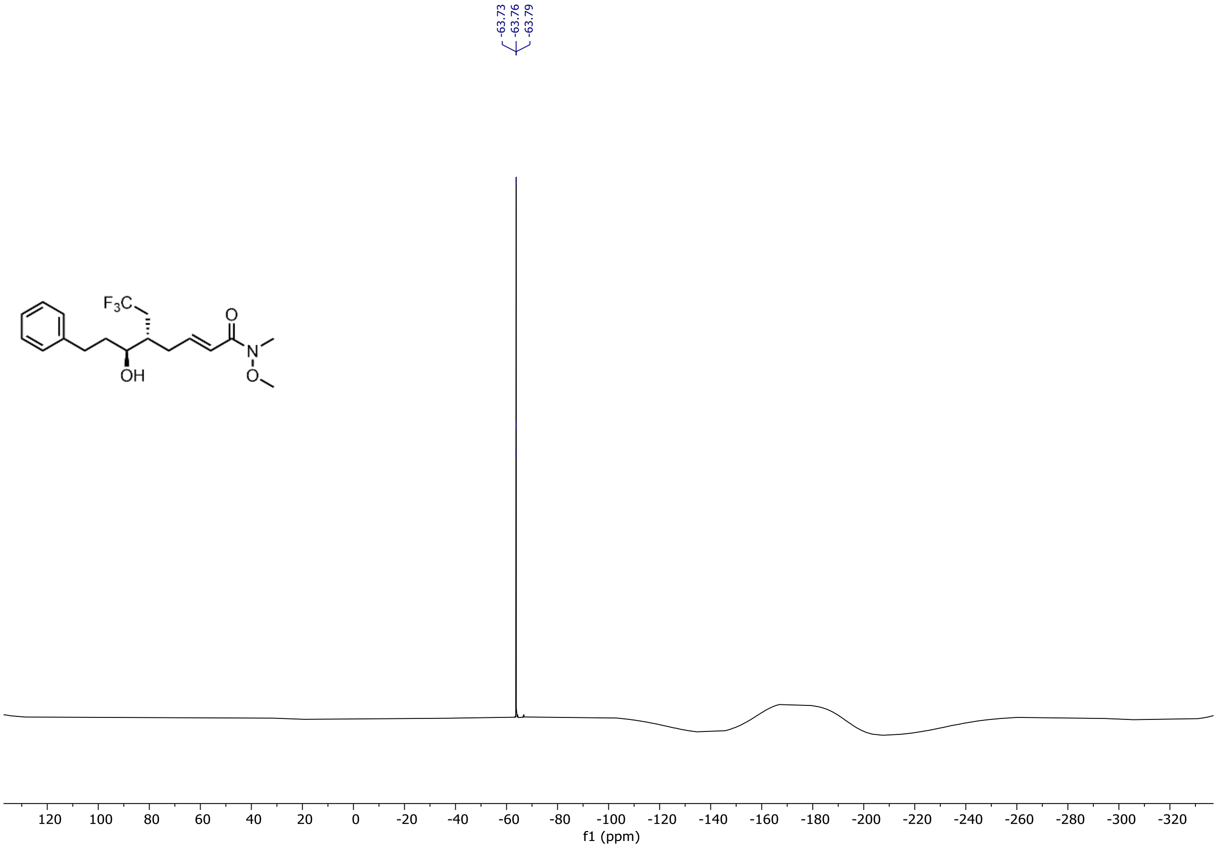
-63.73
-63.76
-63.79
F3C
O
N
O
OH
120 100 80 60 40 20 0 -20 -40 -60 -80 -100 -120 -140 -160 -180 -200 -220 -240 -260 -280 -300 -320
f1 (ppm)

^{1}H NMR (400 MHz, CD_2Cl_2; top), ^{13}C NMR (101 MHz, CD_2Cl_2; middle) and ^{19}F NMR (282 MHz, CD_2Cl_2) of compound **25b**

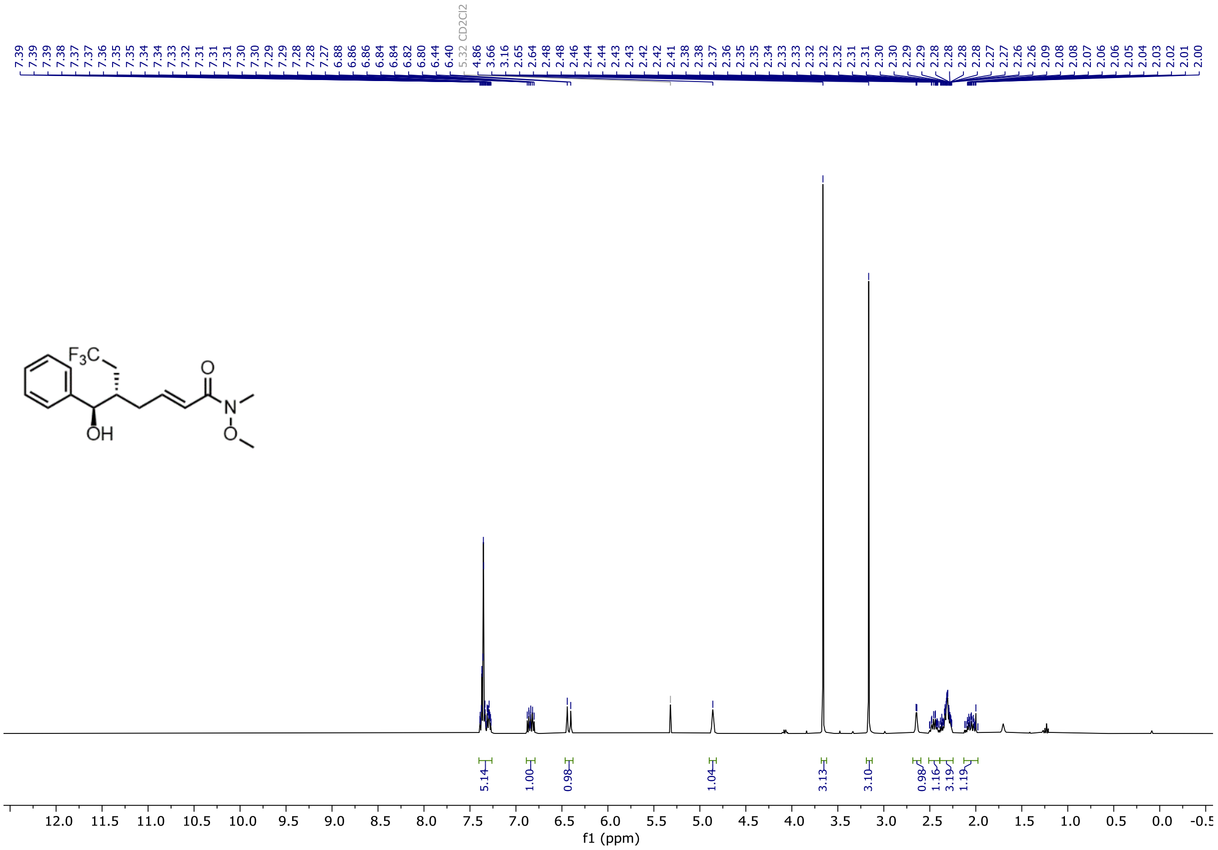

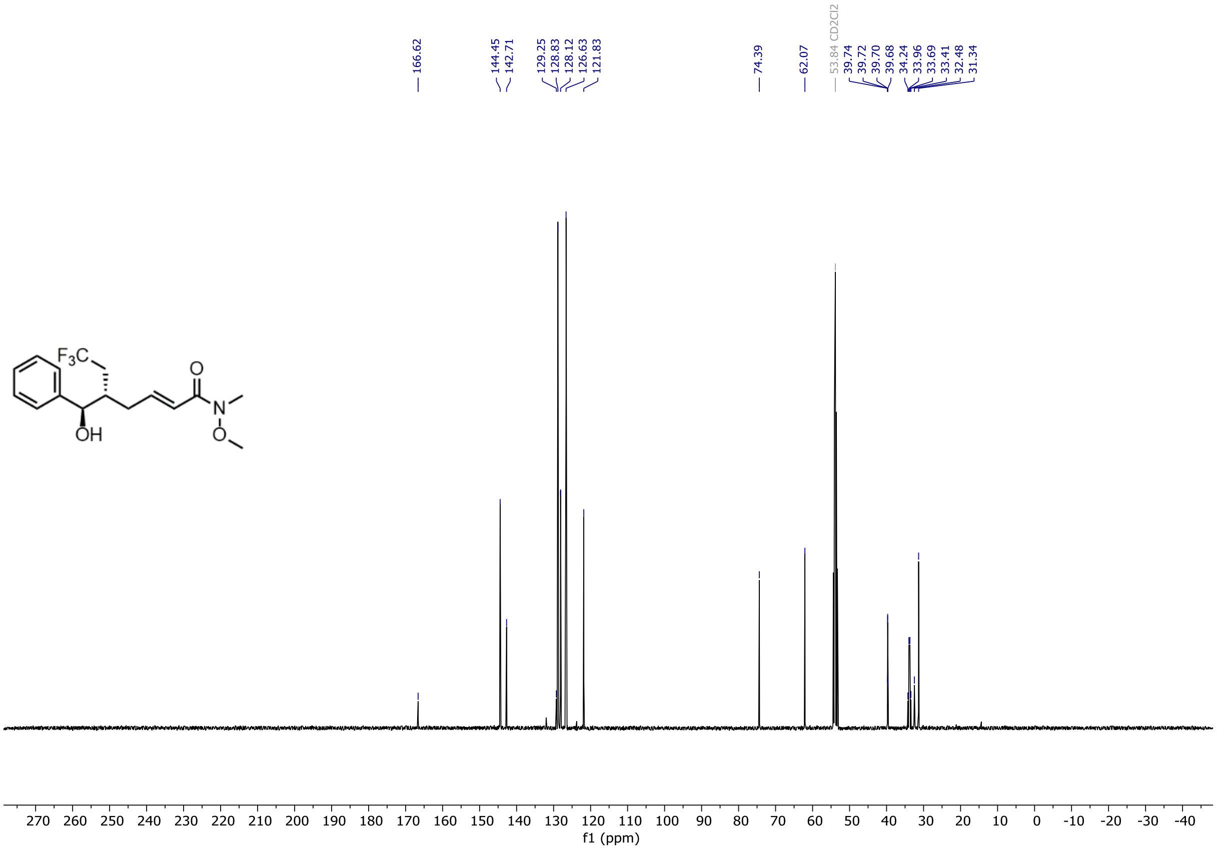

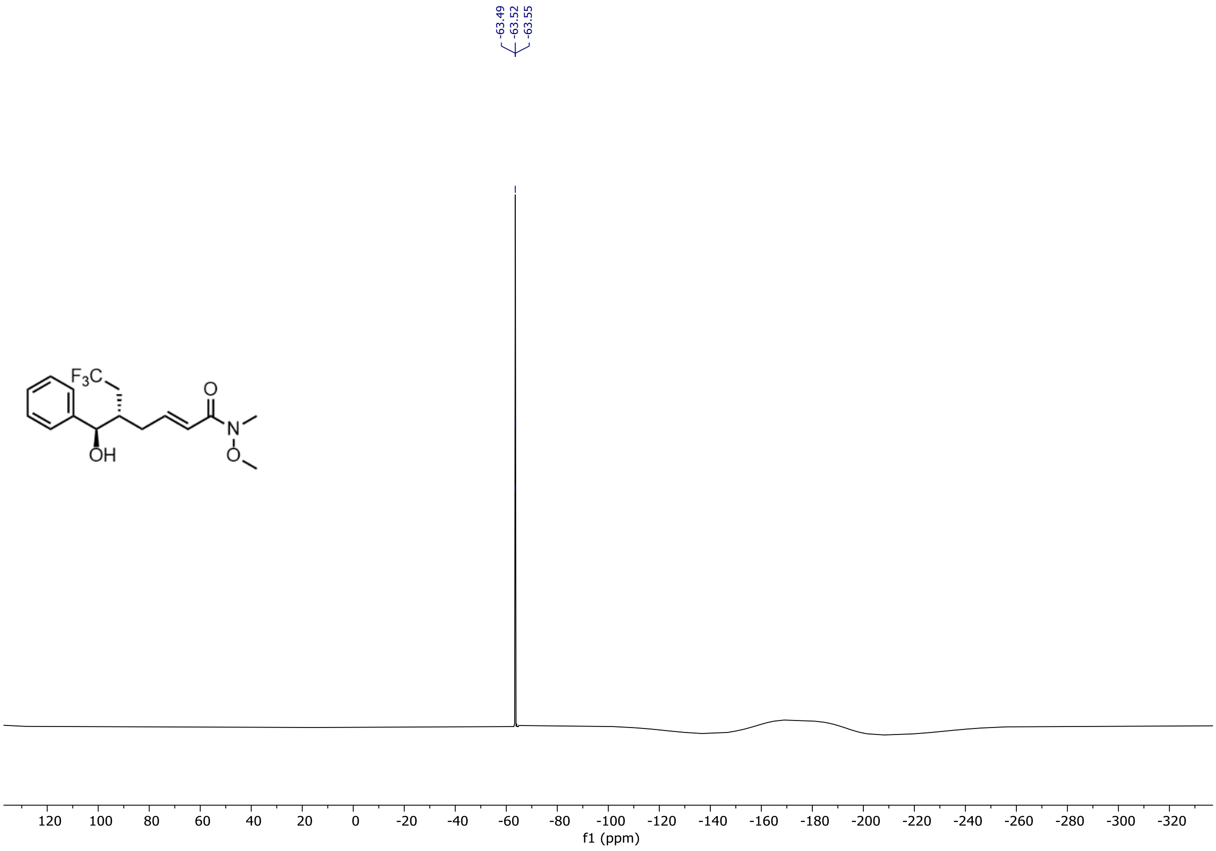
-63.49
-63.52
-63.55
F3C
O
N
O
OH
120 100 80 60 40 20 0 -20 -40 -60 -80 -100 -120 -140 -160 -180 -200 -220 -240 -260 -280 -300 -320
f1 (ppm)

^{1}H NMR (400 MHz, $CDCl_3$; top), ^{13}C NMR (101 MHz, $CDCl_3$; middle) and ^{19}F NMR (282 MHz, $CDCl_3$) of compound **25c**

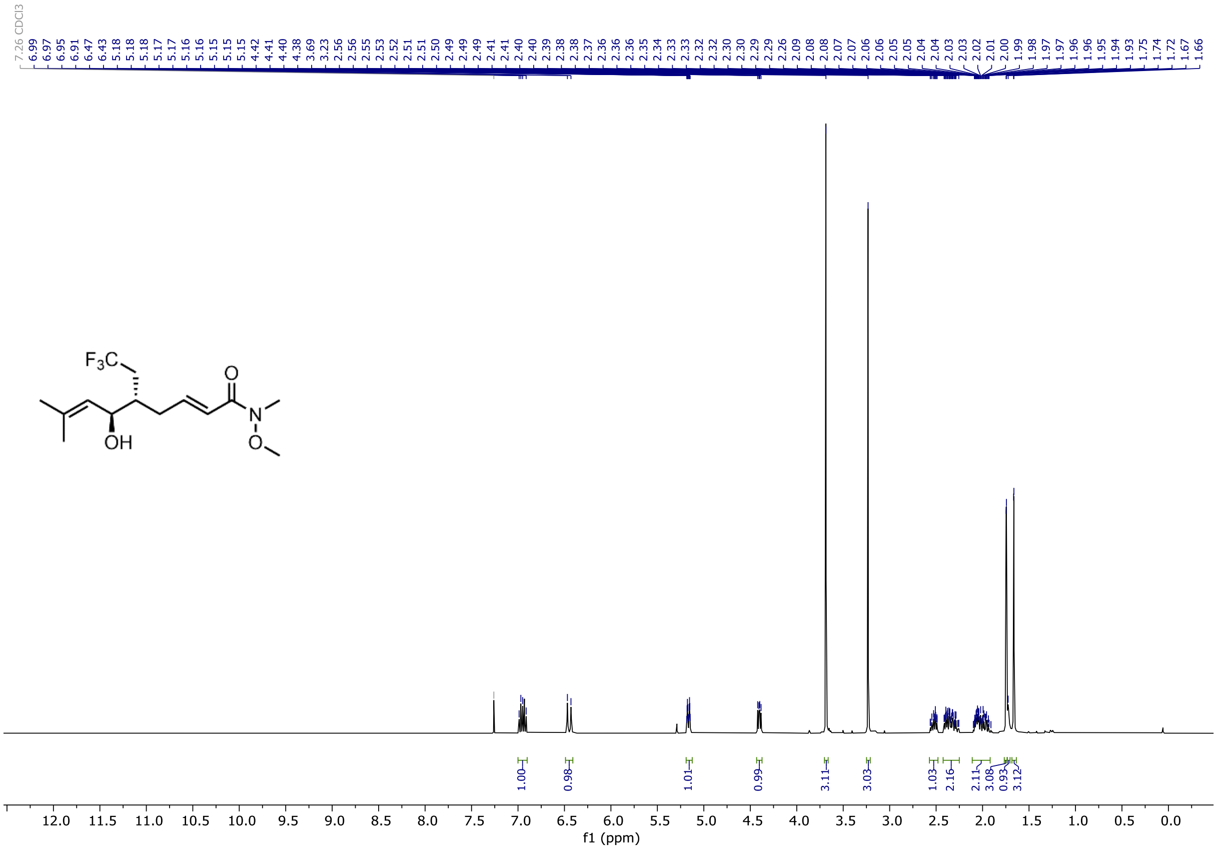

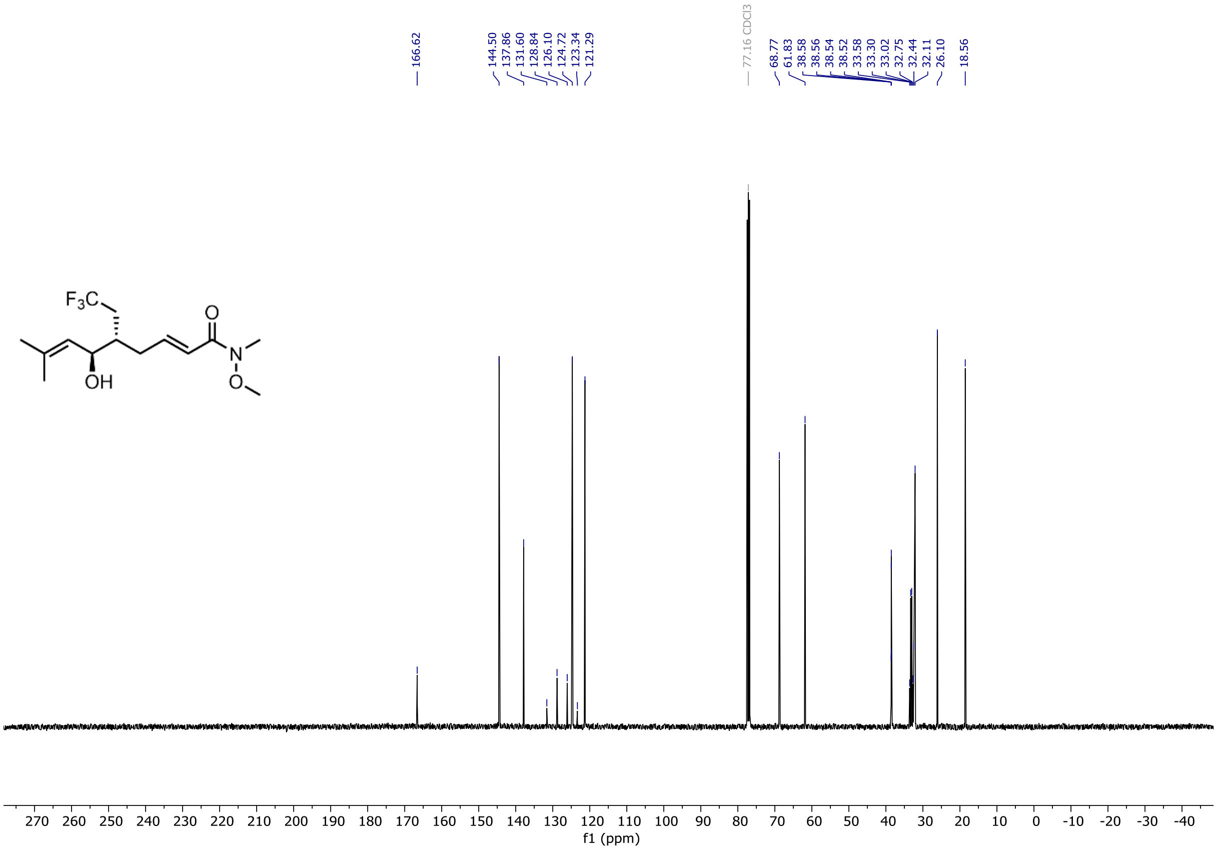

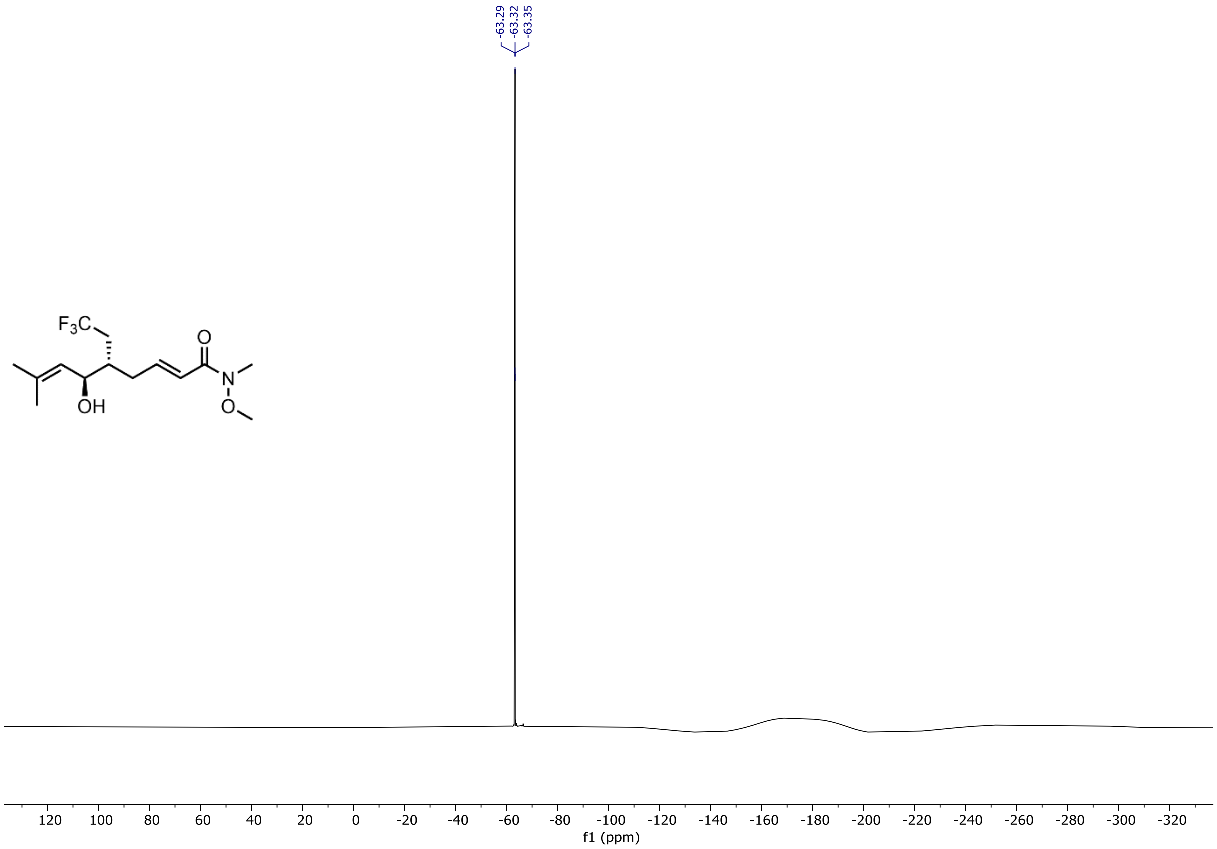
F3C
O
N
O
OH
-63.29
-63.32
-63.35
120 100 80 60 40 20 0 -20 -40 -60 -80 -100 -120 -140 -160 -180 -200 -220 -240 -260 -280 -300 -320
f1 (ppm)

^{1}H NMR (400 MHz, CD_2Cl_2; top), ^{13}C NMR (101 MHz, CD_2Cl_2; middle) and ^{19}F NMR (282 MHz, CD_2Cl_2) of compound **26**

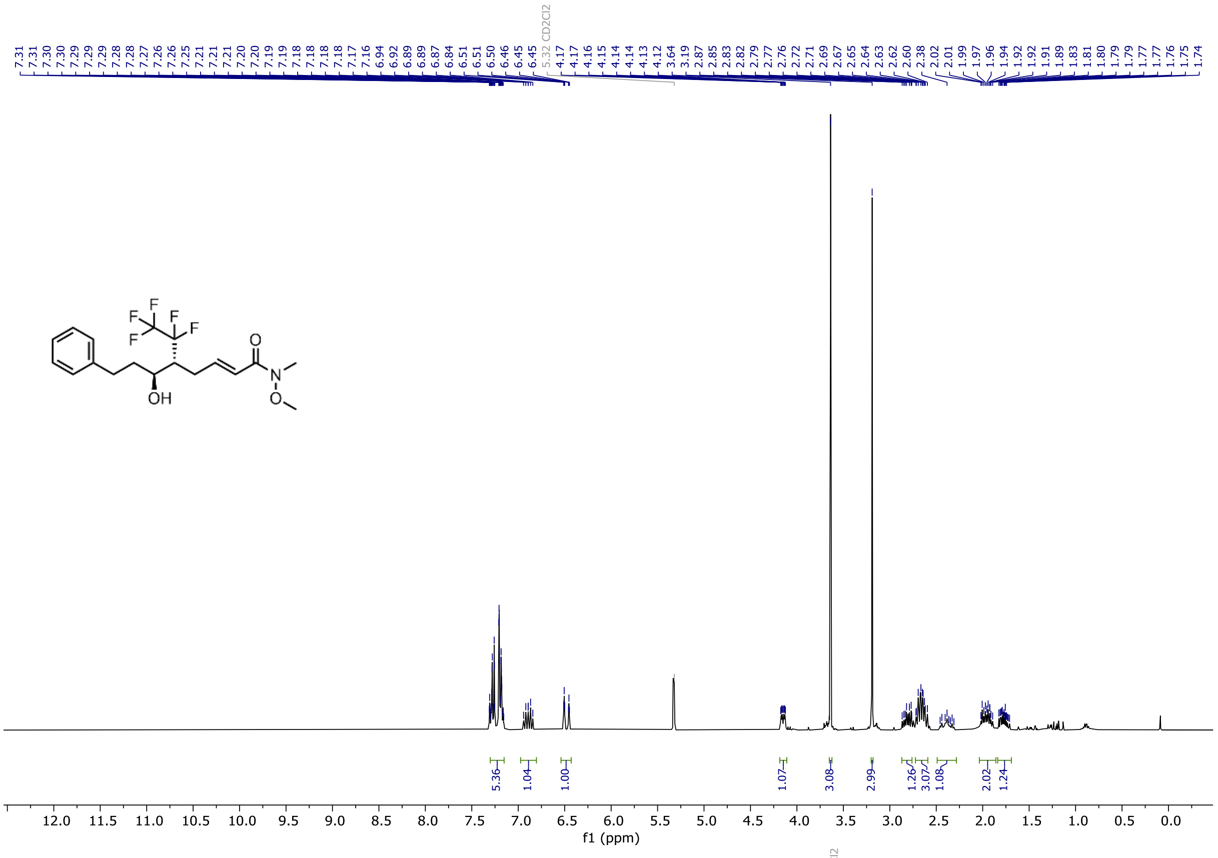

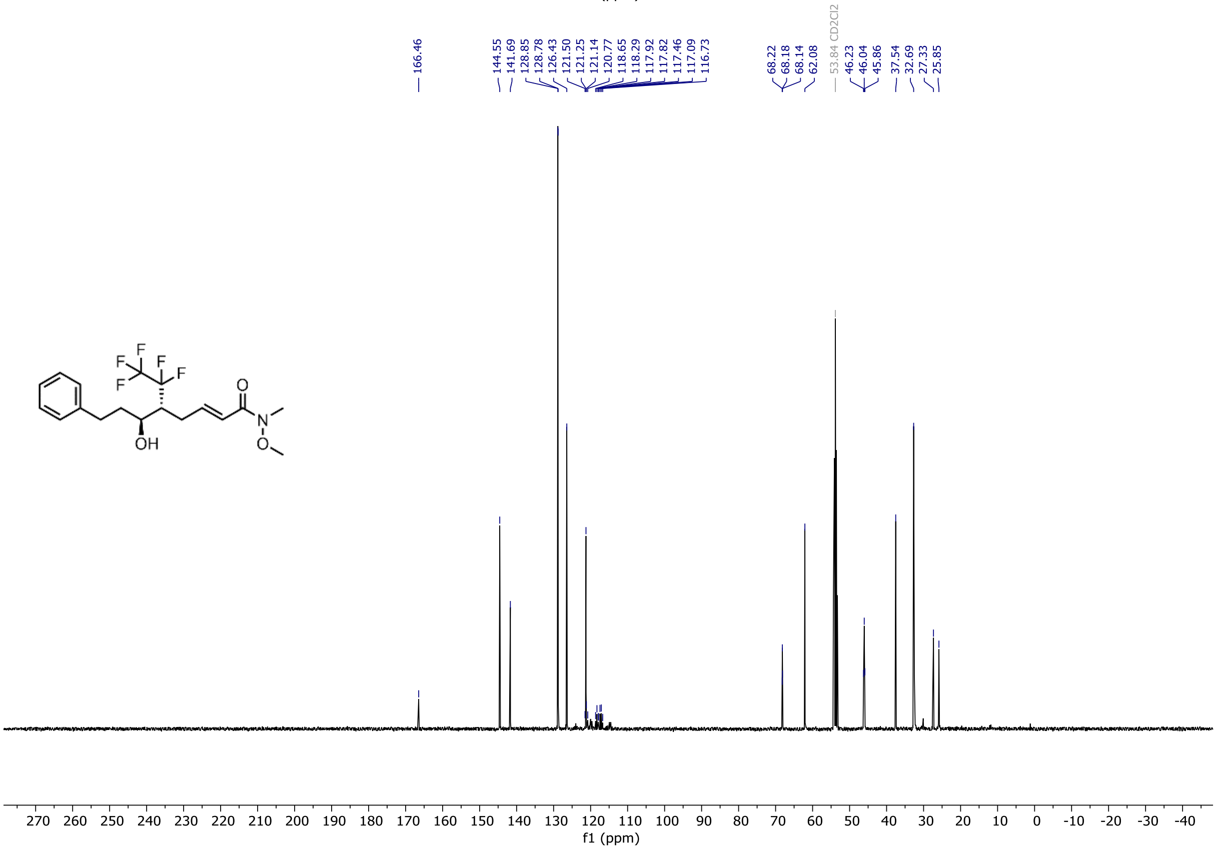

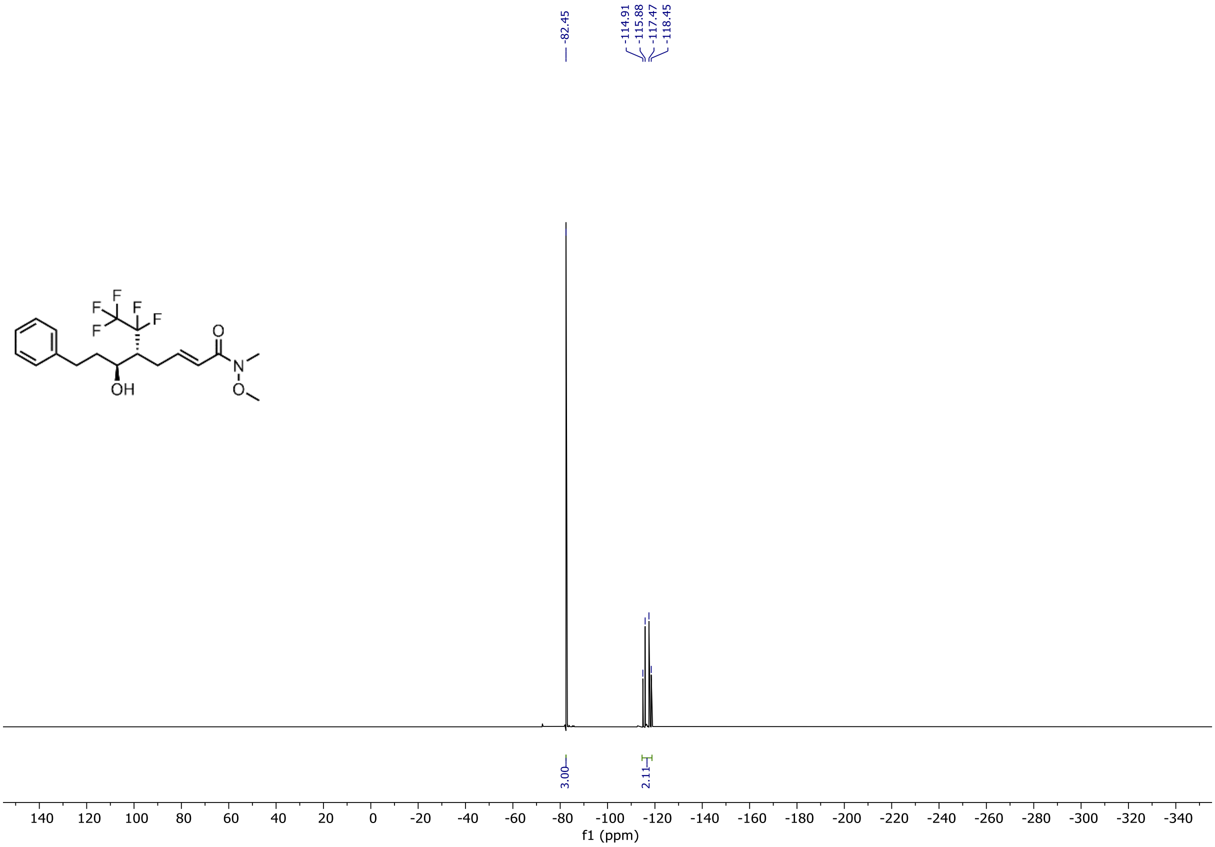
-82.45
-114.91
-115.88
-117.47
-118.45
3.00
2.11
140 120 100 80 60 40 20 0 -20 -40 -60 -80 -100 -120 -140 -160 -180 -200 -220 -240 -260 -280 -300 -320 -340
f1 (ppm)

^{1}H NMR (400 MHz, $CDCl_3$; top), ^{13}C NMR (101 MHz, $CDCl_3$; middle) and ^{19}F NMR (282 MHz, $CDCl_3$) of the mixture comprising compound **27a (blue)** and **27b (red)**

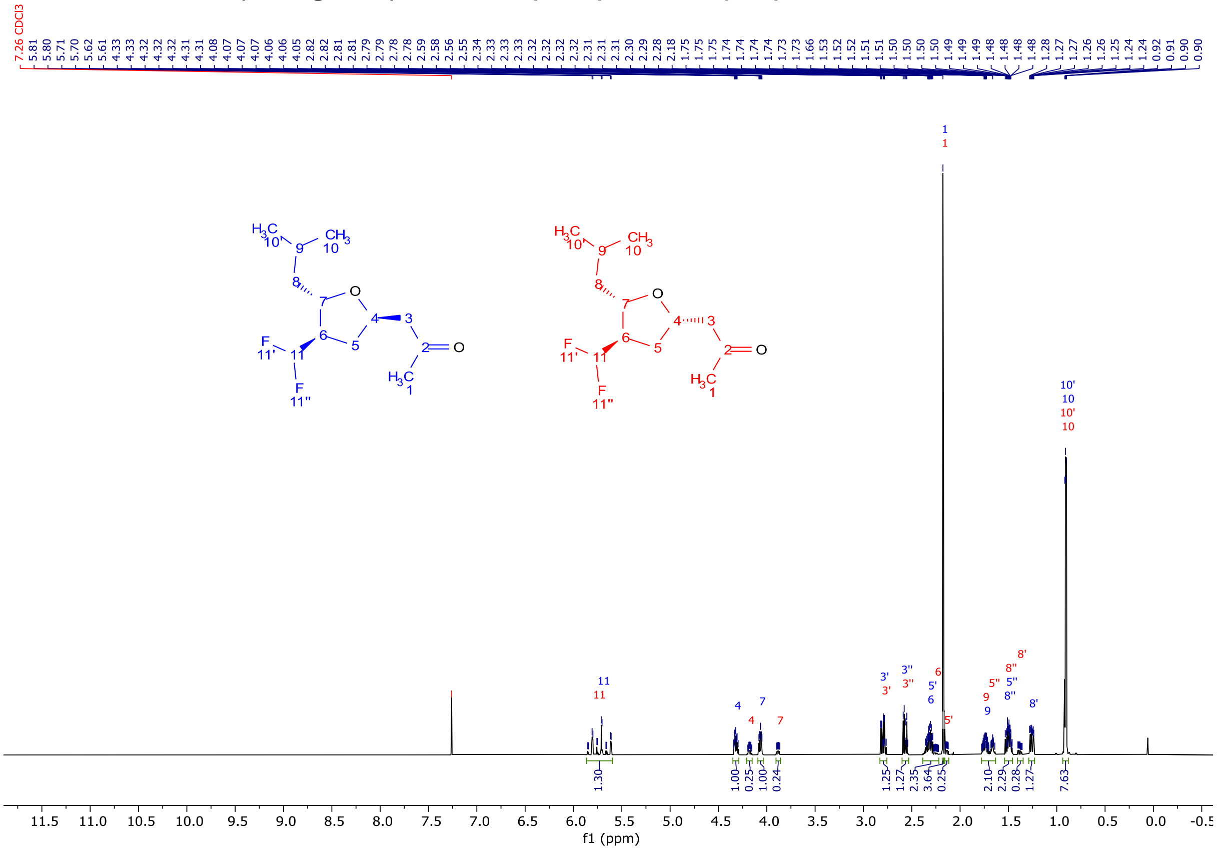

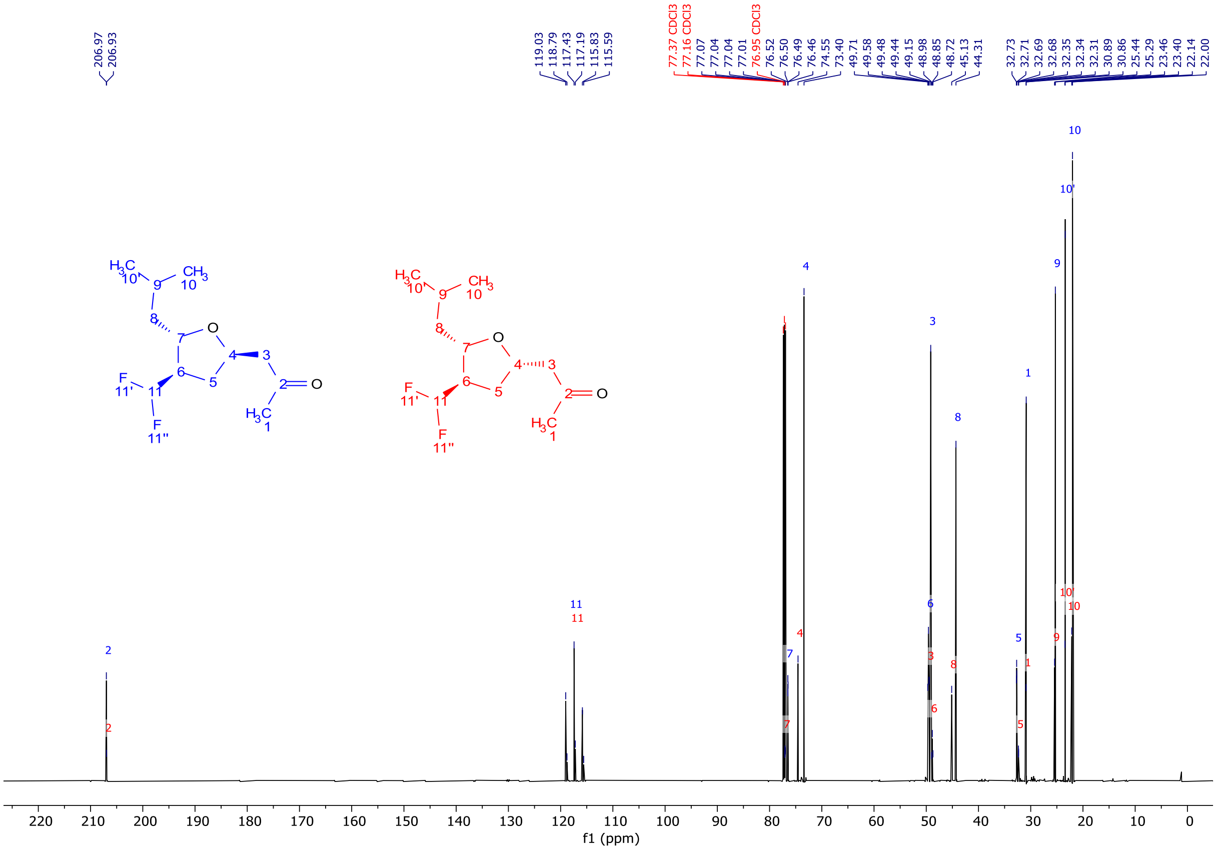

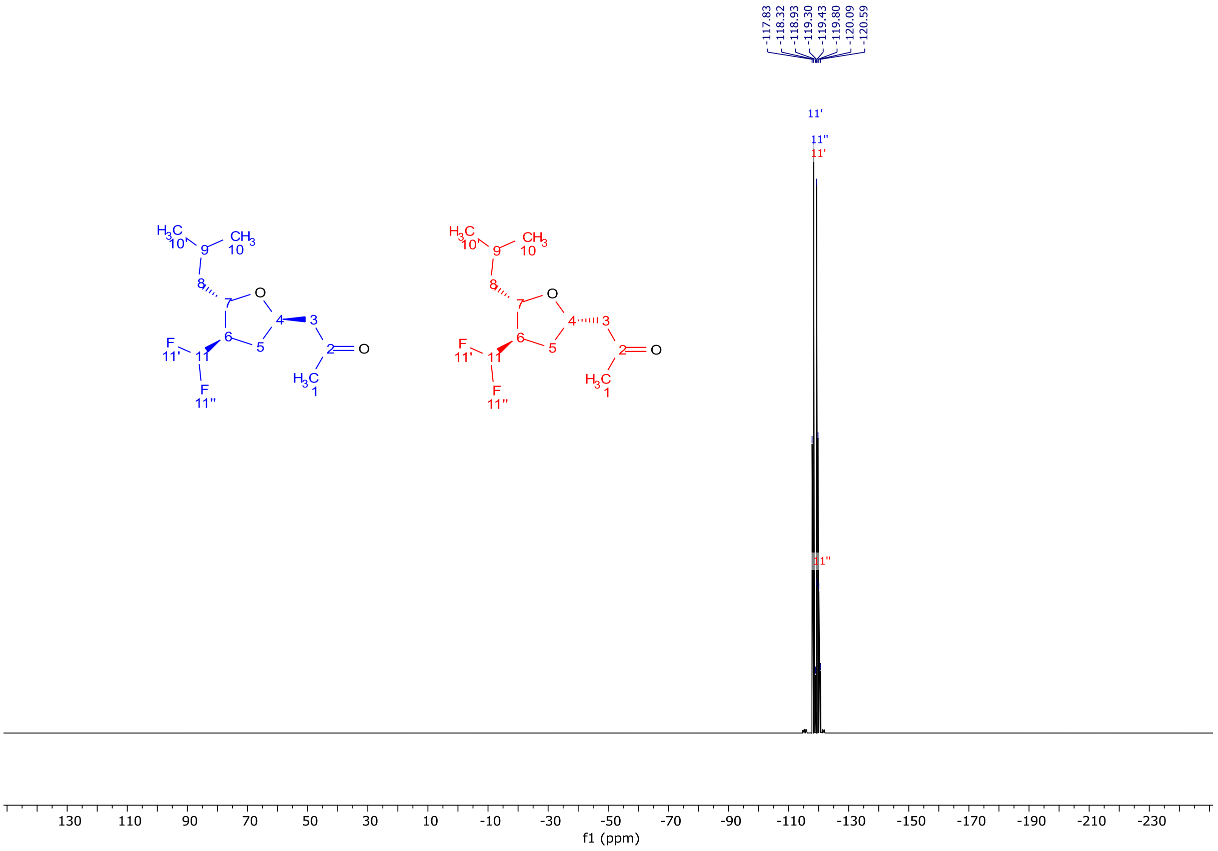

-117.83
-118.32
-118.93
-119.30
-119.43
-119.80
-120.09
-120.59
130 110 90 70 50 30 10 -10 -30 -50 -70 -90 -110 -130 -150 -170 -190 -210 -230
f1 (ppm)

^{1}H NMR (400 MHz, $CDCl_3$; top), ^{13}C NMR (101 MHz, $CDCl_3$; middle) and ^{19}F NMR (282 MHz, $CDCl_3$) of compound **28**

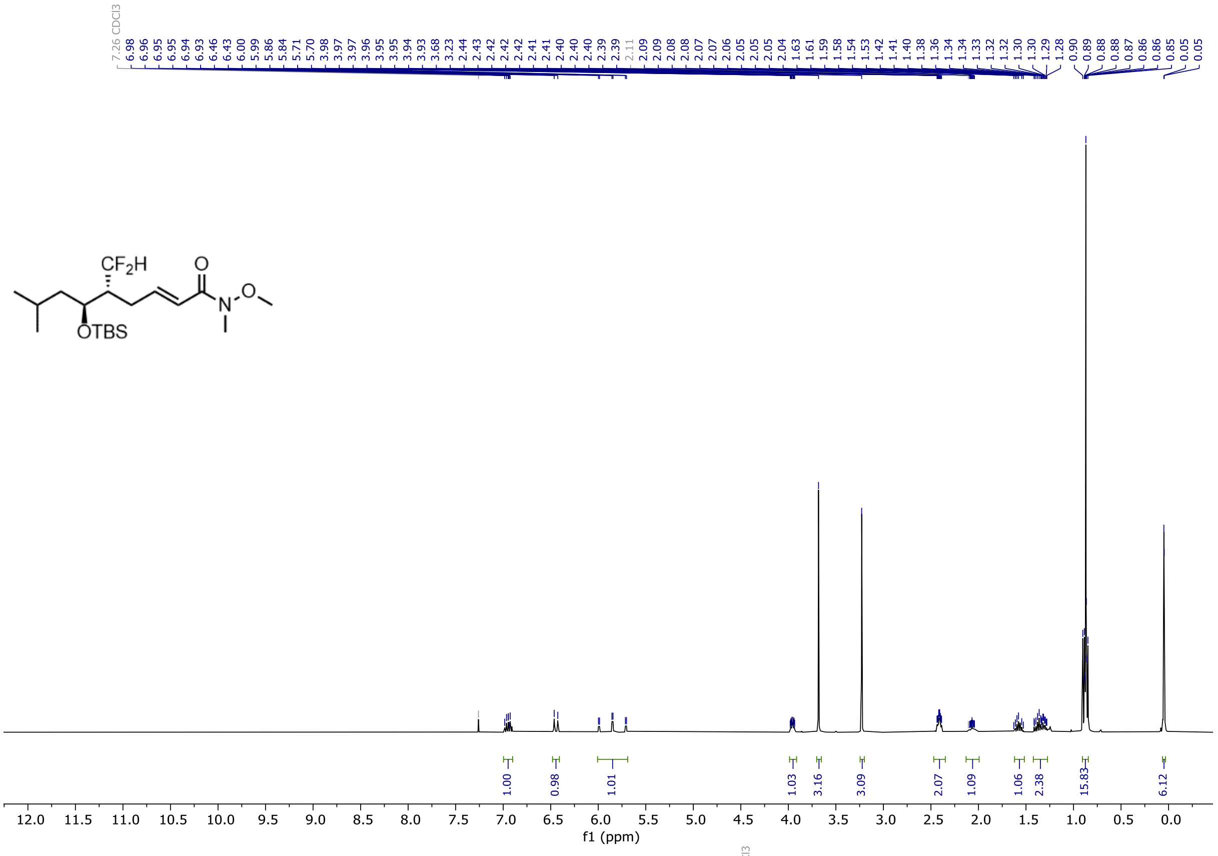

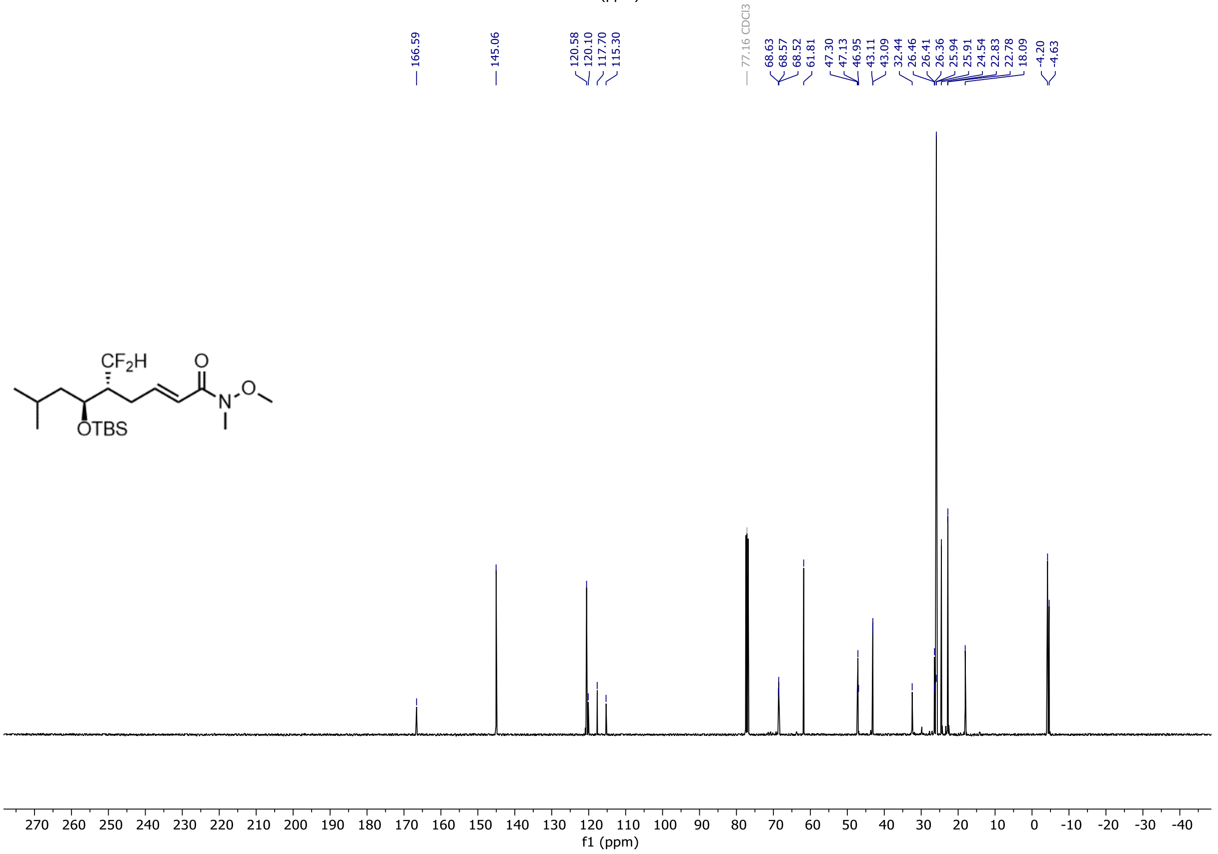

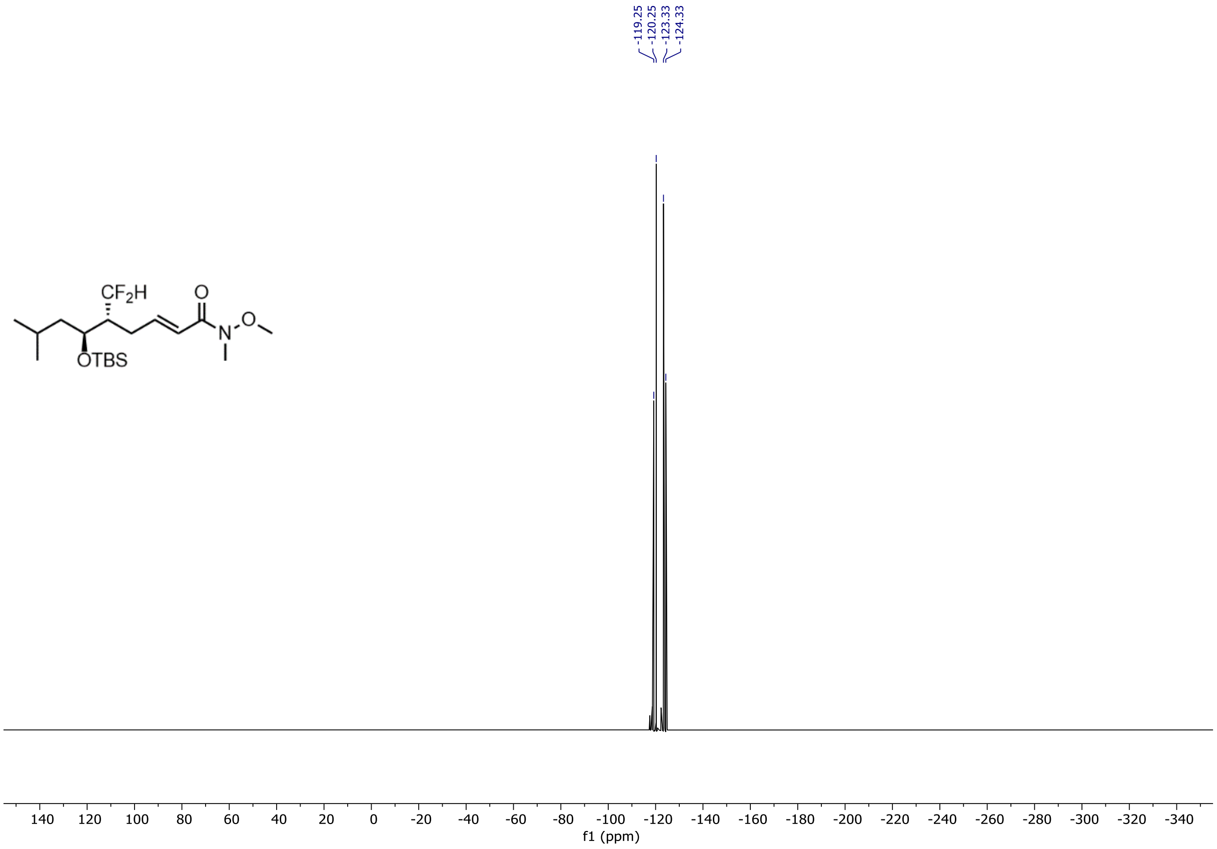
CF2H
O
N
O
OTBS
-119.25
-120.25
-123.33
-124.33
140 120 100 80 60 40 20 0 -20 -40 -60 -80 -100 -120 -140 -160 -180 -200 -220 -240 -260 -280 -300 -320 -340
f1 (ppm)

^{1}H NMR (400 MHz, $CDCl_3$; top), ^{13}C NMR (101 MHz, $CDCl_3$; middle) and ^{19}F NMR (282 MHz, $CDCl_3$) of compound **29**

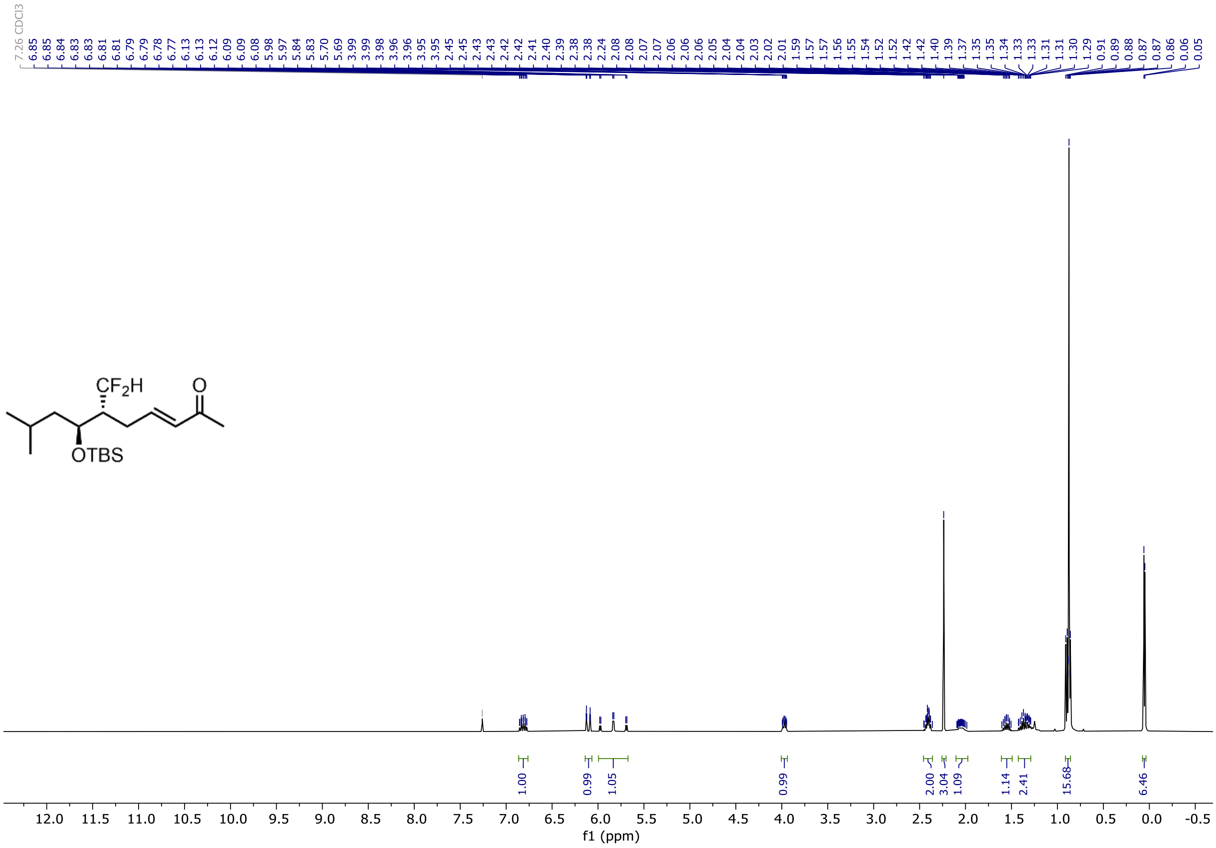

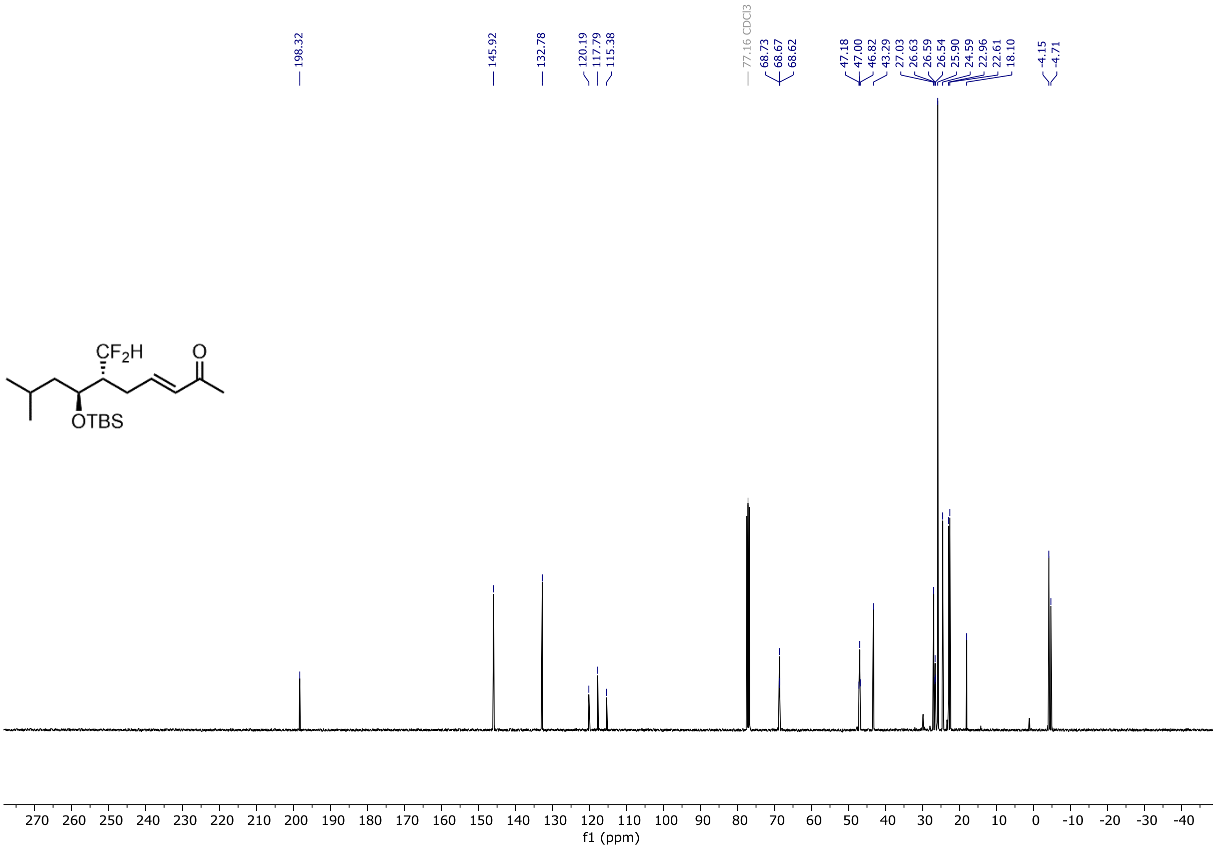

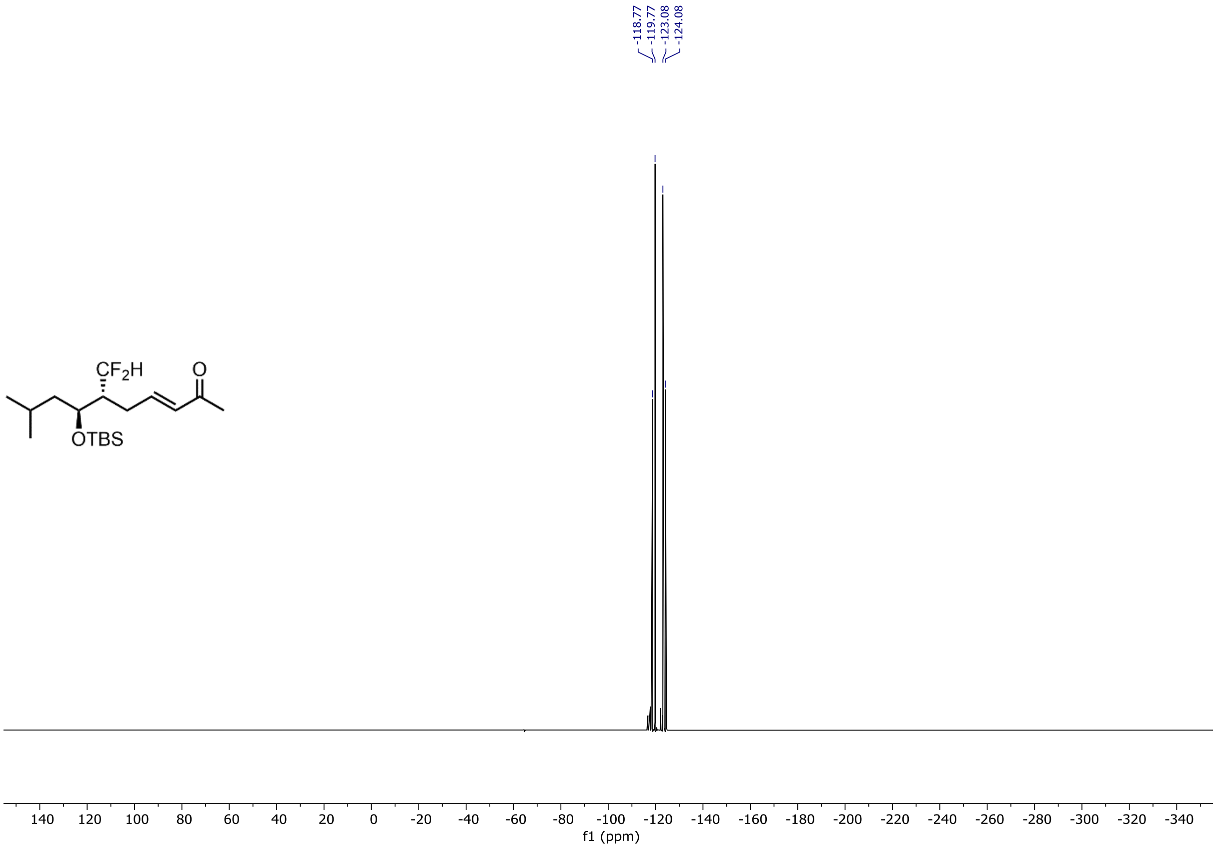
-118.77
-119.77
-123.08
-124.08
CF2H
O
OTBS
140 120 100 80 60 40 20 0 -20 -40 -60 -80 -100 -120 -140 -160 -180 -200 -220 -240 -260 -280 -300 -320 -340
f1 (ppm)

^{1}H NMR (400 MHz, $CDCl_3$; top), ^{13}C NMR (101 MHz, $CDCl_3$; middle) and ^{19}F NMR (282 MHz, $CDCl_3$) of compound **30**

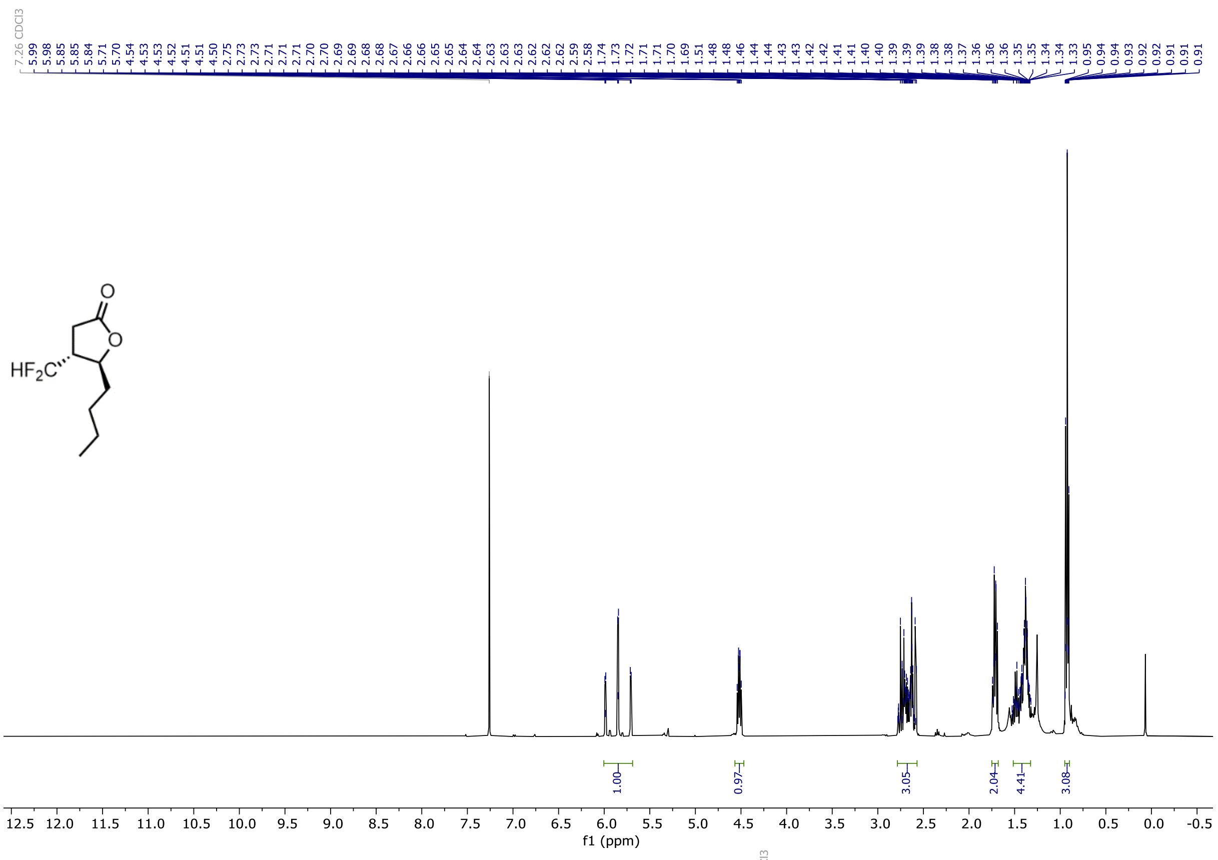

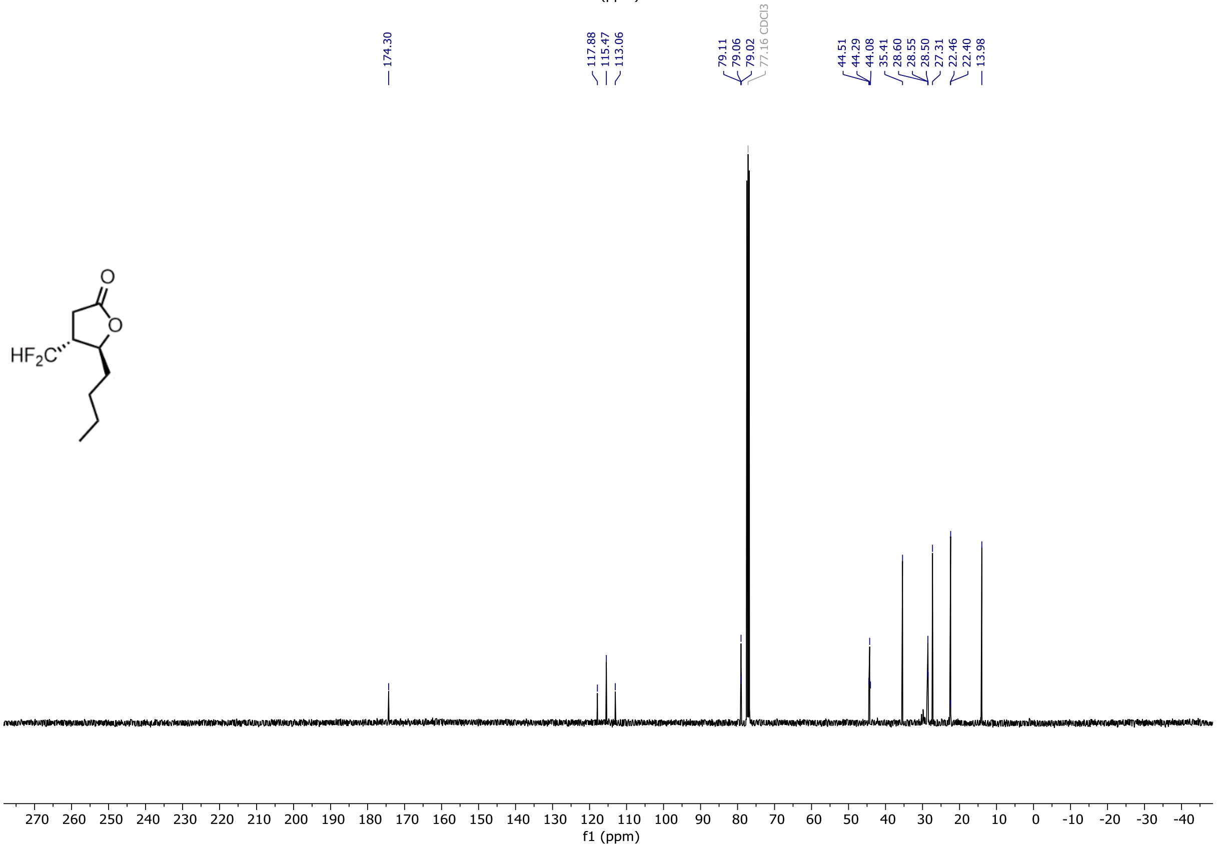

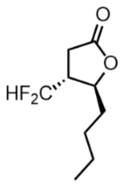
HF2C
O
O

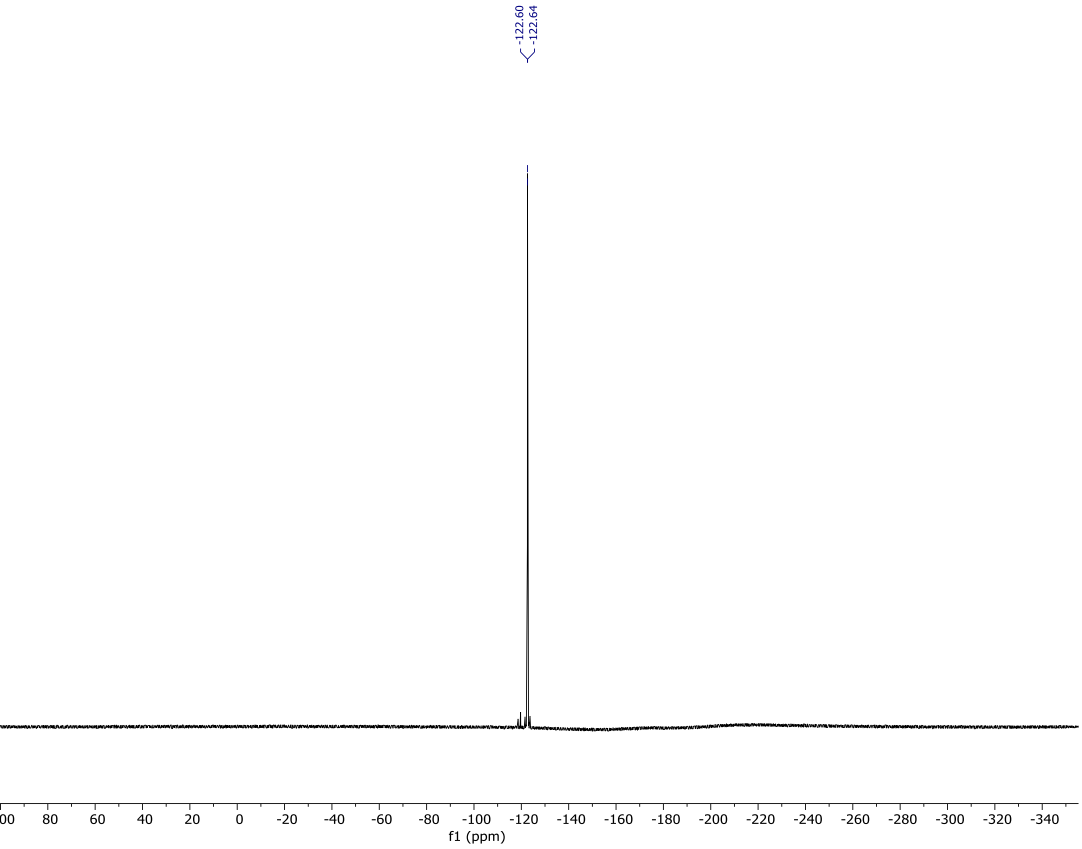
-122.60
-122.64
140 120 100 80 60 40 20 0 -20 -40 -60 -80 -100 -120 -140 -160 -180 -200 -220 -240 -260 -280 -300 -320 -340
f1 (ppm)

^{1}H NMR (400 MHz, $CDCl_3$; top), ^{13}C NMR (101 MHz, $CDCl_3$; middle) and ^{19}F NMR (282 MHz, $CDCl_3$) of compound **31** (1:1 mixture of anomers)

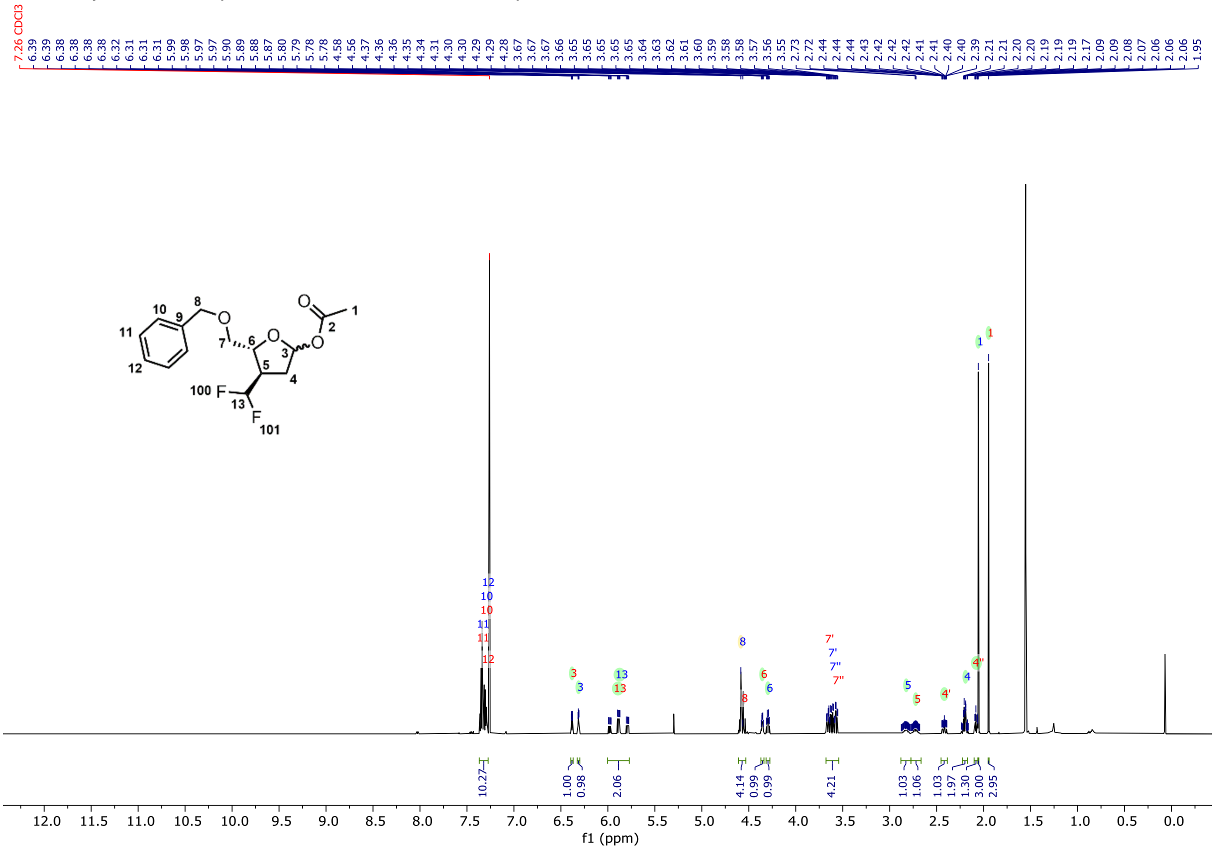

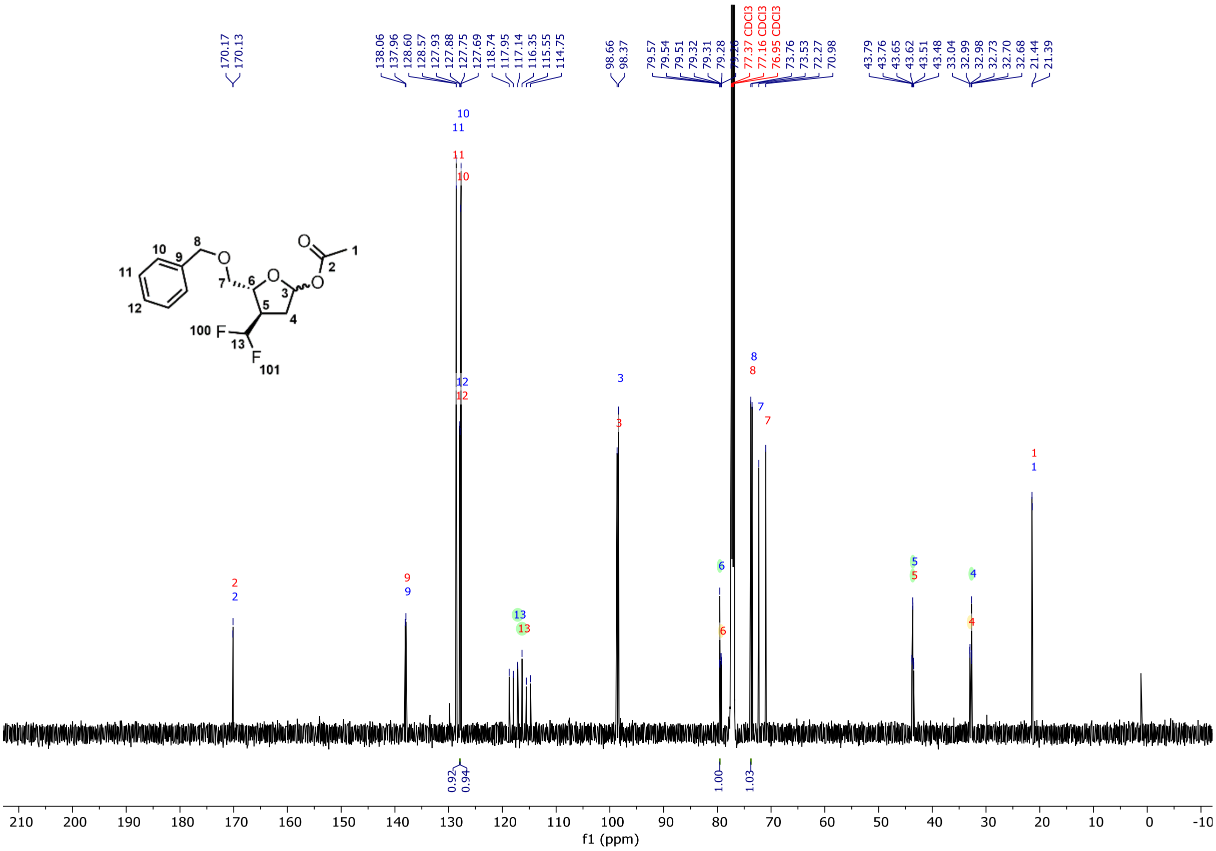

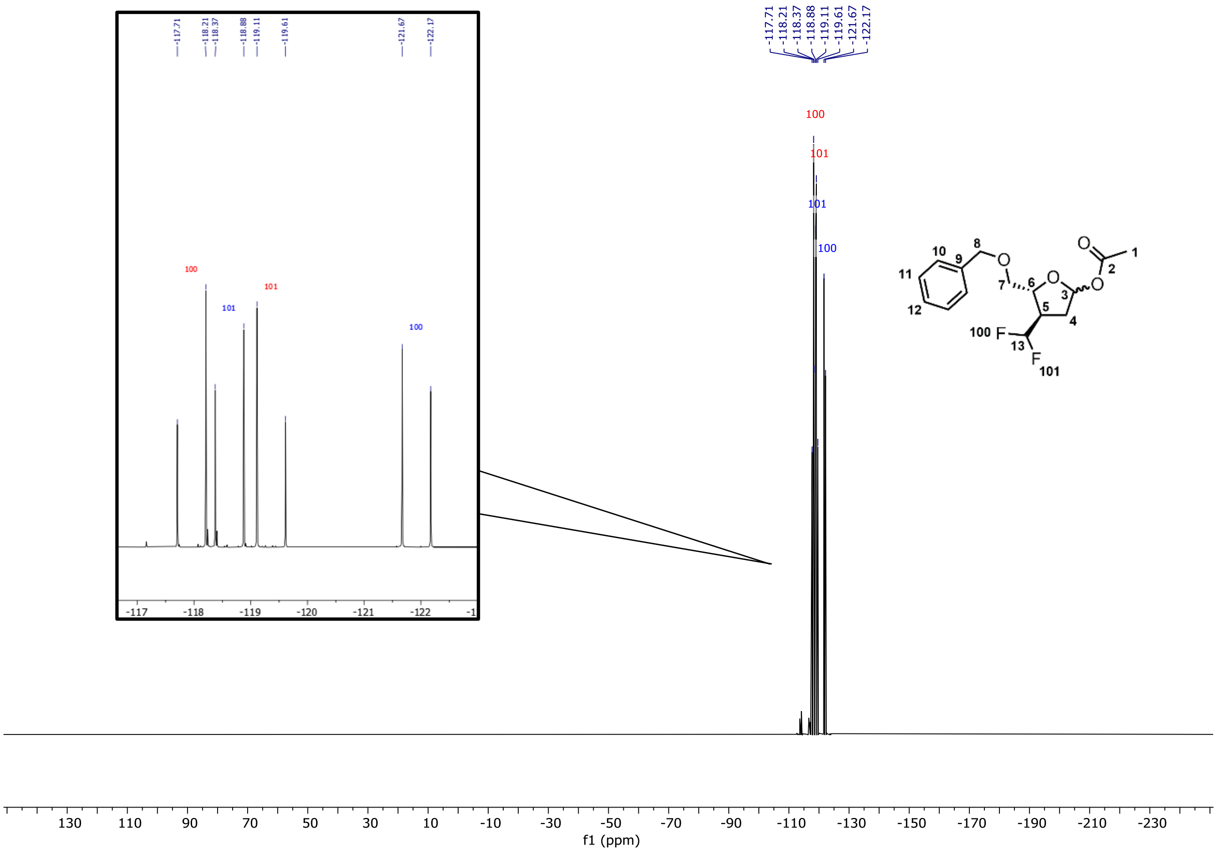
-117.71
-118.21
-118.37
-118.88
-119.11
-119.61
-121.67
-122.17
100
101
101
100
f1 (ppm)

References

1. Wilson, M. S.; Woo, J. C. S.; Dake, G. R., A Synthetic Approach toward Nitiol: Construction of Two 1,22-Dihydroxynitianes. *J. Org. Chem.* **2006,** *71* (11), 4237-4245.
2. Feutrill, J. T.; Lilly, M. J.; Rizzacasa, M. A., Total Synthesis of (+)-Crocacin D. *Org. Lett.* **2002,** *4* (4), 525-527.
3. Earle, M. J.; Abdur-Rashid, A.; Priestley, N. D., Large Scale Synthesis of Cyclodiphospho-d-glycerate. *J. Org. Chem.* **1996,** *61* (16), 5697-5700.
4. Ding, Z.; Osminski, W. E. G.; Ren, H.; Wulff, W. D., Scalable Syntheses of the Vaulted Biaryl Ligands VAPOL and VANOL via the Cycloaddition/Electrocyclization Cascade. *Prg. Process Res. Dev.* **2011,** *15* (5), 1089-1107.
5. Davies, T. Q.; Murphy, J. J.; Dousset, M.; Fürstner, A., Nickel-Catalyzed Enantioselective Synthesis of Pre-Differentiated Homoallylic syn- or anti-1,2-Diols from Aldehydes and Dienol Ethers. *J. Am. Chem. Soc.* **2021,** *143* (34), 13489-13494.
6. Ogilvie, F. B.; Jenkins, J. M.; Verkade, J. G., 31P-31P spin-spin coupling in complexes containing two phosphorus ligands. *J. Am. Chem. Soc.* **1970,** *92* (7), 1916-1923.
7. Kruck, M.; Munoz, M. P.; Bishop, H. L.; Frost, C. G.; Chapman, C. J.; Kociok-Köhn, G.; Butts, C. P.; Lloyd-Jones, G. C., BINOL-3,3′-Triflone N,N-Dimethyl Phosphoramidites: Through-Space 19F,31P Spin–Spin Coupling with a Remarkable Dependency on Temperature and Solvent Internal Pressure. *Chem. Eur. J.* **2008,** *14* (26), 7808-7812.
8. Neese, F., Software Update: The ORCA Program System—Version 6.0. *WIREs Computational Molecular Science* **2025,** *15* (2), e70019.
9. Pracht, P.; Bohle, F.; Grimme, S., Automated exploration of the low-energy chemical space with fast quantum chemical methods. *Physical Chemistry Chemical Physics* **2020,** *22* (14), 7169-7192.
10. Bannwarth, C.; Ehlert, S.; Grimme, S., GFN2-xTB – An Accurate and Broadly Parametrized Self-Consistent Tight-Binding Quantum Chemical Method with Multipole Electrostatics and Density-Dependent Dispersion Corrections. *J. Chem. Theory Comput.* **2019,** *15*, 1652.
11. Spicher, S.; Grimme, S., Robust Atomistic Modeling of Materials, Organometallic, and Biochemical Systems. *Angew. Chem. Int. Ed.* **2020,** *59* (36), 15665-15673.
12. Perdew, J. P.; Burke, K.; Ernzerhof, M., Generalized Gradient Approximation Made Simple. *Physical Review Letters* **1996,** *77* (18), 3865-3868.
13. Caldeweyher, E.; Ehlert, S.; Hansen, A.; Neugebauer, H.; Spicher, S.; Bannwarth, C.; Grimme, S., A generally applicable atomic-charge dependent London dispersion correction. *The Journal of Chemical Physics* **2019,** *150* (15), 154122.

14. Weigend, F.; Ahlrichs, R., Balanced Basis Sets of Split Valence, Triple Zeta Valence and Quadruple Zeta Valence Quality for H to Rn: Design and Assessment of Accuracy. *Phys. Chem. Chem. Phys.* **2005,** *7*, 3297-3305.
15. Cossi, M.; Rega, N.; Scalmani, G.; Barone, V., Energies, structures, and electronic properties of molecules in solution with the C-PCM solvation model. *Journal of Computational Chemistry* **2003,** *24* (6), 669-681.
16. Chai, J.-D.; Head-Gordon, M., Long-range corrected hybrid density functionals with damped atom–atom dispersion corrections. *Physical Chemistry Chemical Physics* **2008,** *10* (44), 6615-6620.
17. Helmich-Paris, B.; de Souza, B.; Neese, F.; Izsák, R., An improved chain of spheres for exchange algorithm. *The Journal of Chemical Physics* **2021,** *155* (10), 104109.
18. Regni, G.; Baldinelli, L.; Bistoni, G., A Quantum Chemical Method for Dissecting London Dispersion Energy into Atomic Building Blocks. *ACS Cent. Sci.* **2025,** *11* (6), 890-898.
19. Baldinelli, L.; De Angelis, F.; Bistoni, G., Unraveling Atomic Contributions to the London Dispersion Energy: Insights into Molecular Recognition and Reactivity. *J. Chem. Theory Comput.* **2024,** *20* (5), 1923-1931.
